# Reaction Products Revised and Mechanisms Revisited with Machine Learning-Augmented Computational NMR

**DOI:** 10.1021/acs.joc.5c02863

**Published:** 2026-03-16

**Authors:** Srinivas Beduru, Enoch Asimbisa, Ivan M. Novitskiy, Andrei G. Kutateladze

**Affiliations:** Department of Chemistry and Biochemistry, 2927 University of Denver, Denver, Colorado 80208, United States

## Abstract

DU8ML, a fast and accurate machine learning-augmented DFT method for computing NMR spectra, has proven effective for high-throughput revision of misassigned natural products. In this paper, we continue with another important underexplored challenge: revision of incorrect product structures of reported organic reactions, often necessitating correction of reaction mechanisms.

## Graphical abstract

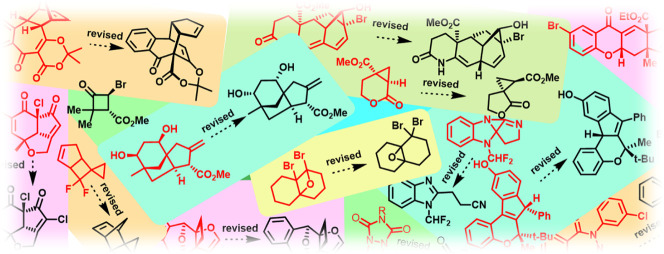

## Introduction

This paper represents the second installment in our ongoing efforts focused on organic structure elucidation and revision outside the realm of natural products. Such corrections frequently carry significant implications for physical organic chemistry and reaction mechanisms. We consider this work both important and timely, as it addresses errors before mechanistic interpretations based on incorrect structures find their way into graduate-level textbooks. We generally focus on recent work, but a revision in a newer paper often triggers the need to revisit an older one, which in turn prompts another, and so on–leading us down the rabbit hole of analyzing seasoned research.

For noncrystalline materials, NMR remains unquestionably the most informative technique for structure elucidation, with extraordinary progress achieved in the development of new pulse sequences and multidimensional experiments.^1^ This experimental work is greatly supported by increasingly user-friendly computational NMR tools, ranging from high-level ab initio and DFT methods^2^ to Computer-Aided Structure Elucidation (CASE) systems. The latter typically employ fast neural network algorithms, still lacking in the accuracy department, but progressively incorporating DFT to improve reliability.^3^ Such computational tools have become commonplace in the field of natural products, but also are increasingly used to aid in structure elucidation of complex synthetic organic molecules.^4^

Our machine learning-augmented DFT method for computational NMR, DU8ML,^5^ allows for fast and accurate computations of nuclear spin-coupling constants and chemical shifts, facilitating the structure elucidation work. Our approach is to ″label″ the substructure fragments, responsible for major deviations of the DFT calculated values, with the appropriate SMARTS strings^6^ and train “the machine” on a large set of reliable experimental nuclear spin coupling constants and chemical shifts to recognize the discrepancies and correct for them. About a decade ago, we expanded on Bally and Rablen’s idea^7^ of scaling Fermi contacts and developed a fast and accurate method for computing nuclear spin–spin coupling constants based on a substructure-aware scaling.^8^ Later we applied a similar methodology for calculations of chemical shifts.^9^ At the time, similar ideas about empirical corrections for systematic errors in calculations were articulated by Gonnella and others.^10^ Most notably, for molecules containing heavy atoms, e.g. halogens, this pragmatic approach circumvented the need to deploy expensive spin–orbit coupling, SOC, calculations. All in all, DU8ML together with its predecessor DU8+ accounted for hundreds of revised structures.^11^

Three years ago, we first applied DU8ML for organic structure revisions which had implications for proposed mechanisms, either suggesting a small modification, or revising the mechanism altogether.^12^ We now continue this work with the second installment of structure revisions which may have ramifications for organic reaction mechanisms.

To simplify navigation across referenced papers, we employ a two-part compound numbering scheme, **XX­{YY}**, where **XX** denotes the sequential numbering in this paper while **YY** corresponds to the original literature numbering.

## Results and Discussion

There are several challenges for structure elucidation work that remain most common in literature. Two of them seem particularly ubiquitous: (i) misassignment of stereoconfiguration in diastereomers, especially, the cases of quaternary stereogenic centers, where the difference in ^13^C chemical shifts for two epimers could be subtle, and (ii) errors in stereoconfiguration or atom connectivity in molecules containing heavy atoms, for example such heavy halogens as bromine or iodine. Calculation of chemical shifts in these cases requires evaluation of “difficult” contributions such as spin–orbit coupling, which are CPU-demanding and take time and resources. All these become particularly acute for “hydrogen-challenged” compounds, where sparsity of hydrogen atoms renders ^1^H NMR largely uninformative. Proton sparsity is a challenge in itself, as it prevents utilization of proton spin–spin coupling constants, possessing a wealth of stereochemical information, and limits the information provided by such requisite 2D NMR methods as HSQC and HMBC. In these cases, computational NMR provides a critically important tool for structure validation and revisions.

### Stereochemistry Challenges

Novel radical cyclization cascade was developed to access the cedrane and clovane sesquiterpene congeners,^13^ Figure 1. Cp_2_TilCl-catalyzed reaction of precursor **5­{12b}** produced the correctly assigned diastereomers **6­{14}** and **7­{15}**. However, **3­{12a}** (possessing a shorter tether) gave purported product **4­{13}**, which disagreed with the DU8ML computed data. We revised it to bis-*endo* isomer **8­{13**-**rev}**, as shown in Figure 1. Stereochemistry in this reaction is set at the **1­{6}** → **2­{7a}** conjugate addition, which necessitated the revision of an earlier intermediate, allyl cyclohexanone **2­{7a}**. While **2­{7a}** and **9­{7a**-**rev}** exhibited very small differences in the computed ^13^C shifts, **9­{7a**-**rev}** still matched better. Correction of its structure also helped to rationalize the formation of **8­{13**-**rev}** for which this revision was clearly required.

**Figure 1:**
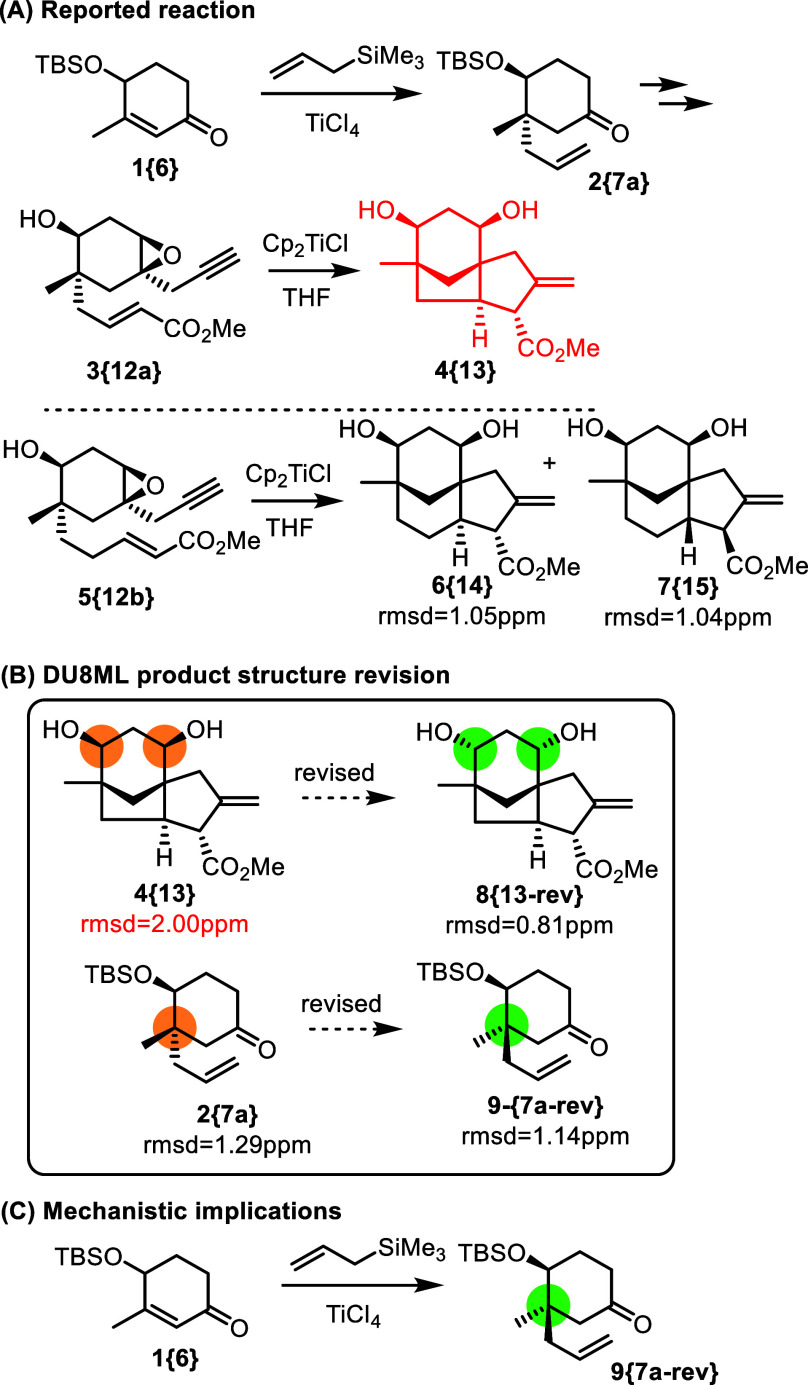
Access to the cedrane and clovane cores.

Our decision-making process relies primarily on RMSDs between experimental and calculated ^13^C chemical shifts. We use a symbolic cutoff of 1.6 ppm as the guiding criterion. In practice, this means that we recommend a structural revision when the revised structure yields an RMSD <1.6 ppm, while all alternative candidates exhibit RMSD >1.6 ppm. A clear example of this scenario is provided by structures **4­{13}** and **8­{13**-**rev}**. More ambiguous cases arise when both RMSD values fall below the 1.6 ppm threshold. These situations require additional scrutiny, including detailed analysis of individual ^13^C chemical shift deviations and proton spin–spin coupling constants. Structures **2­{7a}** and **9­{7a**-**rev}** illustrate such cases involving nearly indistinguishable diastereomers.^14^

Another example of subtle differences between stereoisomers is shown in Figure 2.^15^ Two diastereomeric heteropropellanes **10­{3aa}** and **11­{3aa** ^′^ **}** possessing a ketone moiety were subjected to carbonyl reduction with different reagents, Pd/C/H_2_ or LiAlH_4_. The reactions were diastereoselective, but the hydroxy groups resulting from the ketone reduction was assumed to have an opposite stereoconfiguration in reference to the oxa-bridge. While DU8ML produced an acceptable rmsd = 1.20 ppm for product **13­{4a** ^′^ **}**, one of the bridgehead carbons connected to the oxa-bridge was off by 4.9 ppm (red arrow, Figure 2B). The epimer of **13­{4a** ^′^ **}**, i.e. structure **14­{4a′**-**rev}** did not show such individual atom discrepancies between computed and experimental data, yielding a superior rmsd of 0.97 ppm. We therefore revised structure **13­{4a** ^′^ **}** to **14­{4a′**-**rev}**. This revision implies that the hydride attack in **11­{3aa** ^′^ **}** occurs from the same face of the molecule, which has the oxa-bridge. It is plausible that Li^+^ precoordination to the oxa-bridge affects the positioning of the aluminum hydride counterion and is responsible for this stereochemical outcome, i.e. the hydride delivery from the same face of the molecule.

**Figure 2:**
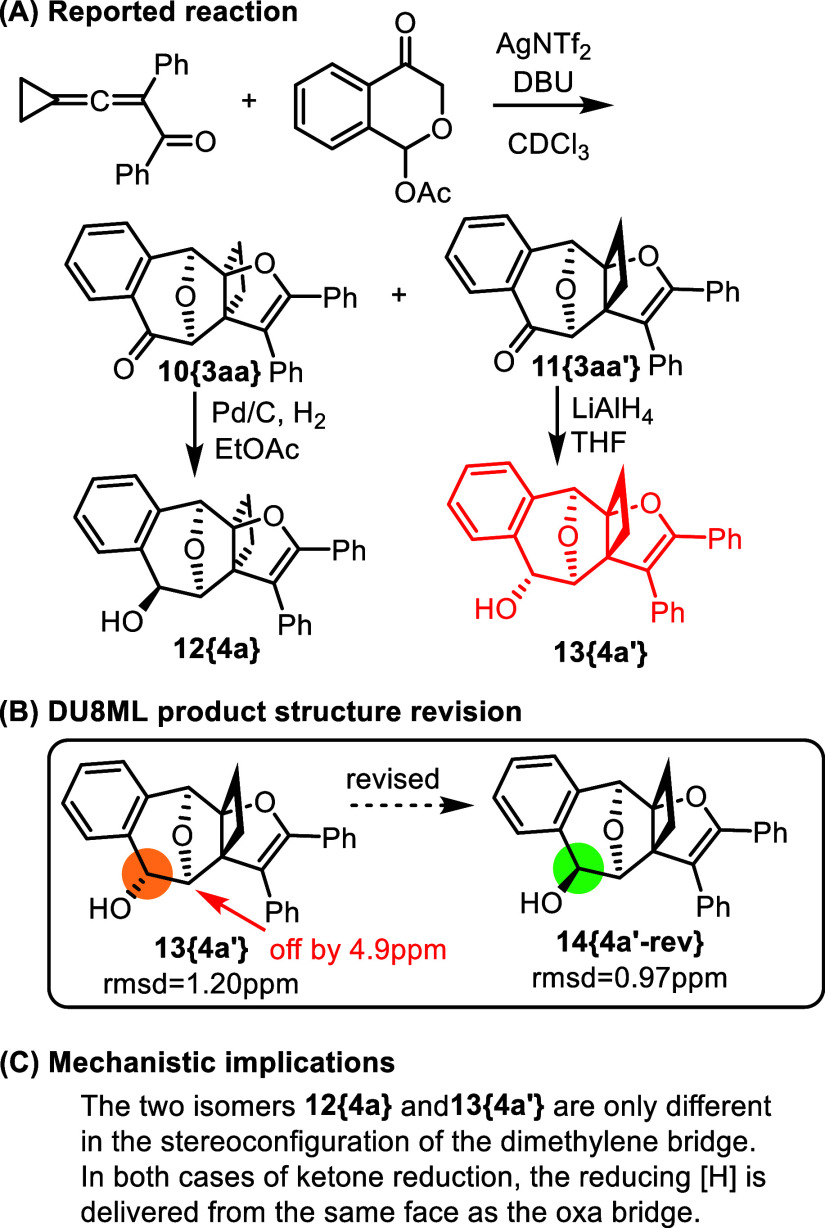
Reduction of heteropropellanes.

Another difficult case of stereochemical assignment was on display in an NHC-catalyzed [2 + 2] cycloadditions of ketones to ketenes yielding β-lactones, Figure 3.^16^ The stereochemical outcome was difficult to assess due to the lack of informative *J*-coupling constants in proton spectra. Luckily, there was an X-ray for compound **19­{3l}** clarifying the “*trans*” stereoconfiguration. This served as a good reference point to test DU8ML’s performance and accuracy, which was determined to be fully adequate (rmsd = 0.97 ppm for **19­{3l}**). The incorrect *cis*-structure expectedly gave inferior rmsd of 1.66 ppm. As Figure 3 shows, the β-lactones bearing R = Me were assigned the correct (*trans*) stereoconfiguration. However, it appears that all products with bulkier R = Et or R = Pr required revision to *cis*-configuration, as shown. Notice that for consistency we use authors’ label “*trans*” to indicate relative stereochemistry of the two aryl group. In the systematic E/Z-nomenclature such trans configuration corresponds to Z-isomer.

**Figure 3:**
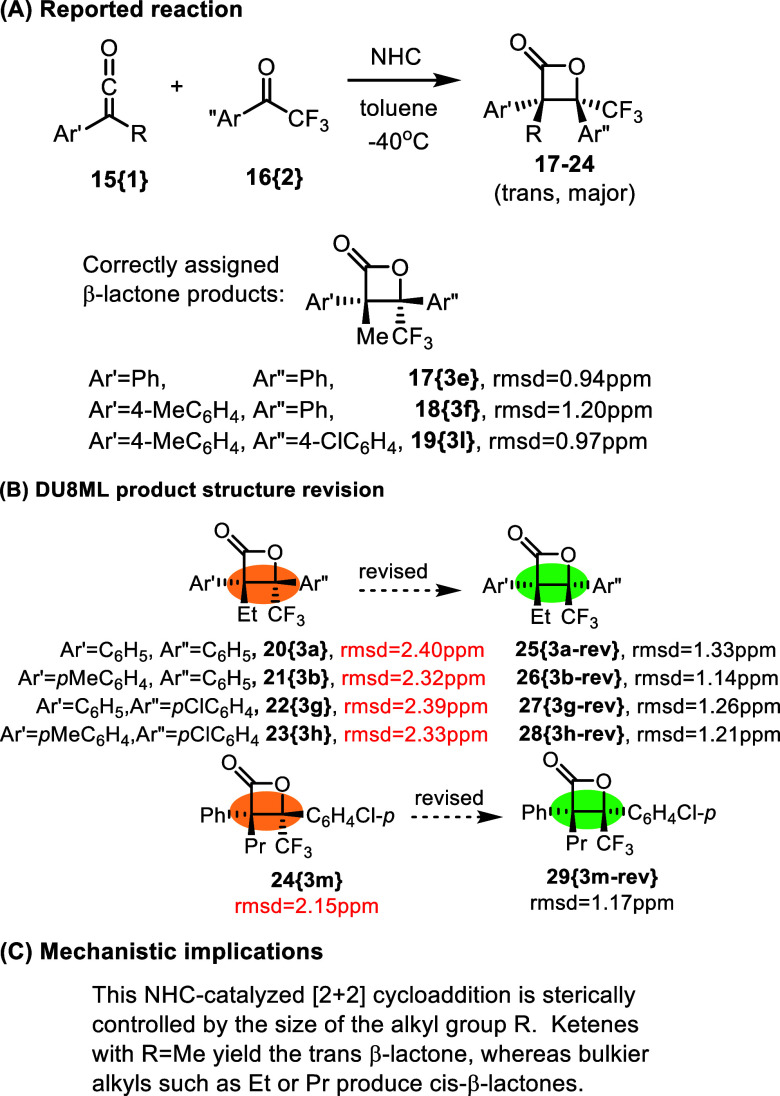
NHC-catalyzed access to β-lactones.

3-Carboxycyclobutanones obtained via a [2 + 2] annulation strategy, were subsequently introduced into secondary reactions, such as bromination with pyridinium tribromide (Figure 4), Bayer-Villiger, and other reactions.^17^ ^13^C chemical shifts calculated for *cis*-product of bromination, **31­{27}**, did not agree with experimental data. We revised this α-bromide to its *trans*-isomer **32­{27**-**rev}**. The monobrominated product of this reaction has two vicinal protons belonging to the BrC­(H)–C­(H)­COOMe moiety. Normally, this vicinal *J*_HH_ would be used for differentiation between the *cis*- and *trans*-form. In this case, this proton spin coupling constant does not help in stereochemical assignment of bromide **31­{27}** because for both forms the calculated constants are very similar, *J*_HH(cis)_ = *8*.*47 Hz* and *J*_HH(trans)_ = *8*.*43 Hz* whereas the experimental *J*_HH(exp)_ for the product is reported at 7.9 Hz. Calculated ^13^C chemical shifts for the *trans*-isomer better match the experimental data than *cis*-isomer, imparting confidence in our revision of the product to *trans*-isomer **32­{27**-**rev}**.

**Figure 4:**
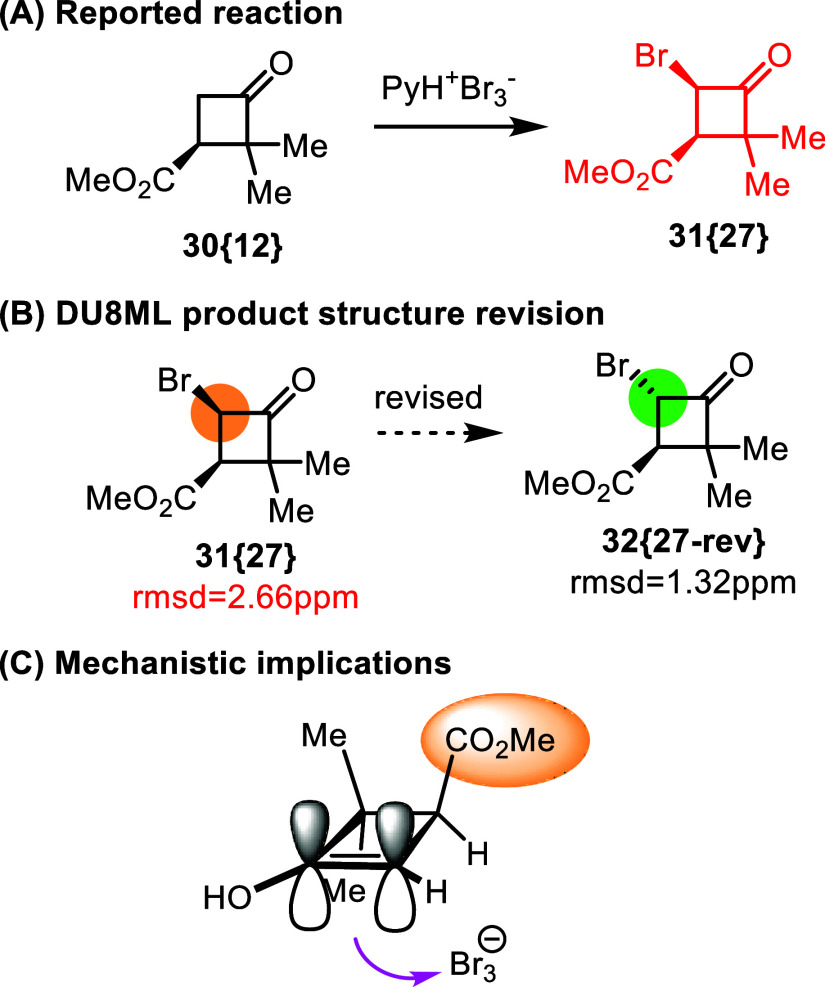
α-Bromination of cyclobutanone **30­{12}** with pyridinium tribromide.

It is likely that bromination of **30­{12}** occurs via transient enol form, and that the mildly electrophilic brominating species, the tribromide Br_3_^–^, approaches from the less sterically hindered face, Figure 4C.

Cyclopentanol-fused vinyl housanes **34­{2n}** (major) and **35­{2n** ^′^ **}** (minor) were recently accessed via a sensitized photolysis of cyclopropenyl precursors **33­{1}**.^18^ The majority of other photoproducts, including the major product **34­{2n}**, were correctly assigned as possessing the endohydroxy stereoconfiguration, Figure 5. However, the minor diastereomer **35­{2n** ^′^ **}** needed revision to bis-endo product **36­{2n′**-**rev}**, i.e. it has the same endoconfiguration of the hydroxy group as in other products; yet, it is the *C*-vinyl *epimer* of the major product **34­{2n}**. This observation may necessitate invoking a hydrogen bond from the OH group to the cyclopropenyl moiety, “anchoring” a particular conformation of the hydroxyalkyl “tether” in the transition state leading to the *endo*–OH products.

**Figure 5:**
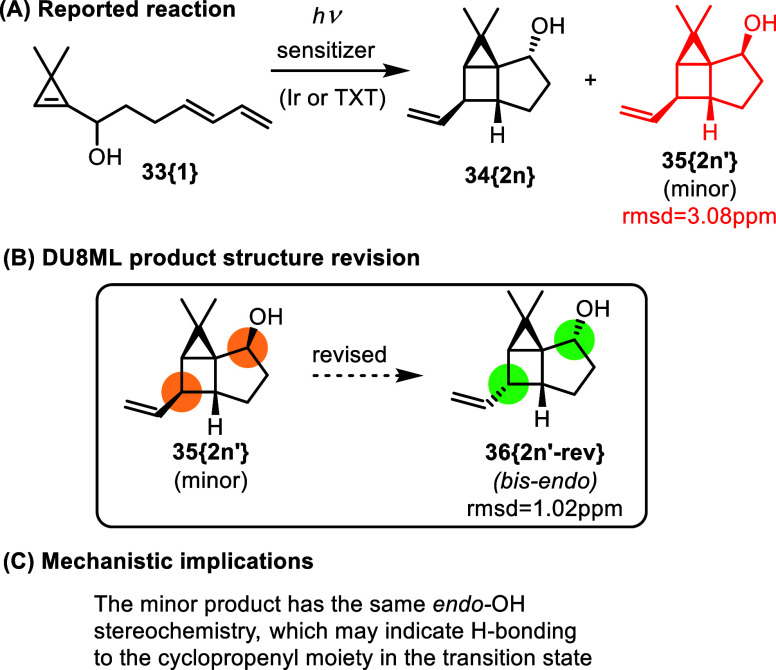
Synthesis of housanes via sensitized irradiation.

An epic divergent synthetic study toward crinipellins facilitated by a HAT-initiated Dowd-Beckwith rearrangement produced a number of structure elucidation challenges, given that many of the model reactions carried out by the authors dealt with synthesis of triquinanes **38­{13}** and **39­{14}**, Figure 6, and related compounds.^19^ Triquinanes, as we demonstrated in prior work, very often cause problems in structure elucidation of natural products.^20^ Yet, the majority of triquinanes **38­{13}**/**39­{14}** and their precursors **37­{12}** were characterized correctly attesting to the thorough structure elucidation work by the authors. We found only two subtle cases of misassignment, which are particularly difficult to identify: (i) triquinane **41­{14j}** needed revision (epimerization) of the CHMe stereogenic center, and (ii) precursor **43­{12m}** required revision of the ethylidene group to its Z-isomer **44­{12m**-**rev}**.

**Figure 6:**
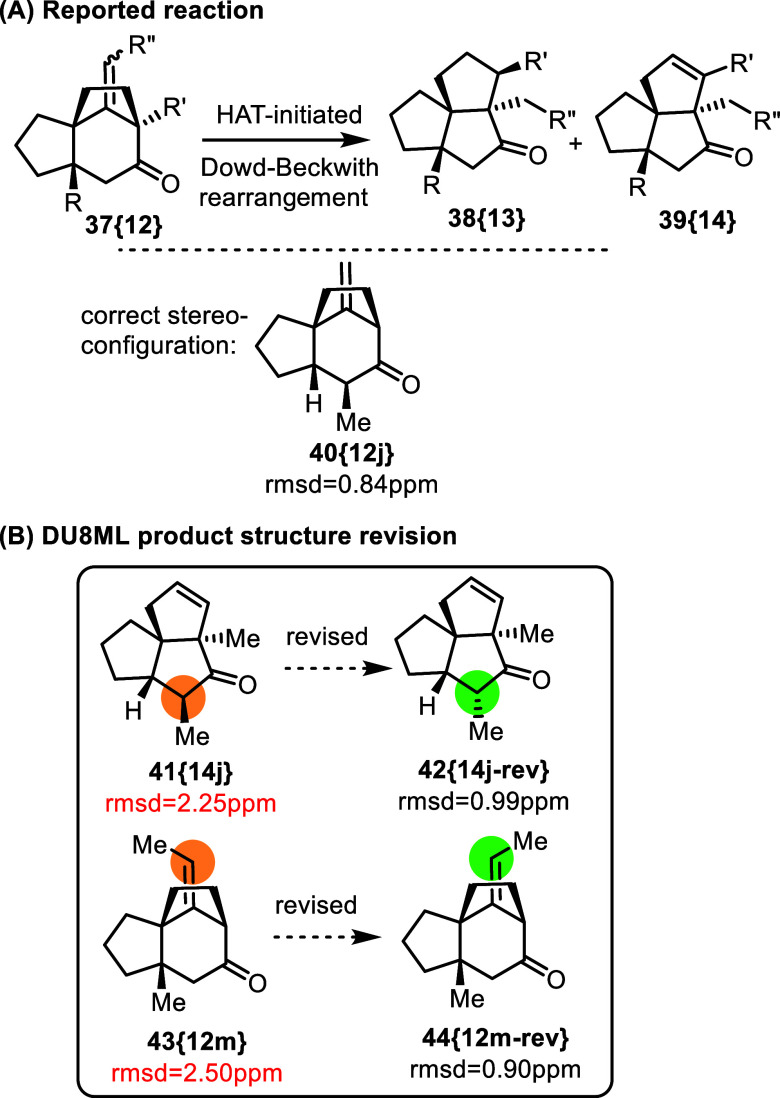
HAT-initiated Dowd-Beckwith rearrangement toward synthesis of crinipellins.

Luckily, the two revisions did not affect any major mechanistic conclusions in this study. Since the structure of **40­{12j}**–i.e. precursor of **41­{14j}**–was established correctly, it appears that the HAT-initiated Dowd-Beckwith rearrangement of **40­{12j}** is accompanied by epimerization of the α-carbonyl carbon (this was the only case of an α-Me-substituted carbonyl in the series). Due to insufficient signal dispersion in the proton spectrum of **43­{12m}**, NOESY determination of the configuration of ethylidene is challenging, and the revision to Z-isomer **37­{12**-**rev}** is not surprising. It is also immaterial for subsequent stereochemistry considerations, as this ethylidene group becomes *ethyl* as a result of HAT-initiated Dowd-Beckwith rearrangement.

### Chemo- and Regioselectivity Challenges

A recent report on *N*-heterocyclic carbene-catalyzed asymmetric cyclization described expeditious access to complex tricyclic diketones as shown in Figure 7.^21^ The cyclization has a broad scope, and the structures were assigned correctly for the majority of products. However, one of the products, **46­{4m}**, derived from the shown cyclohexane-fused substrate needed revision. DU8ML gave unacceptably poor match for the ^13^C chemical shifts of the originally proposed structure, which required revision to **47­{4m**-**rev}**. The mechanistic implications include an alternative mode of the initial attack of the zwitterionic intermediate on the triply- (*green*)–not doubly substituted (*orange*) alkene of the cyclohexadienone moiety. It could plausibly indicate that electronic effects override the unfavorable steric effect.

**Figure 7:**
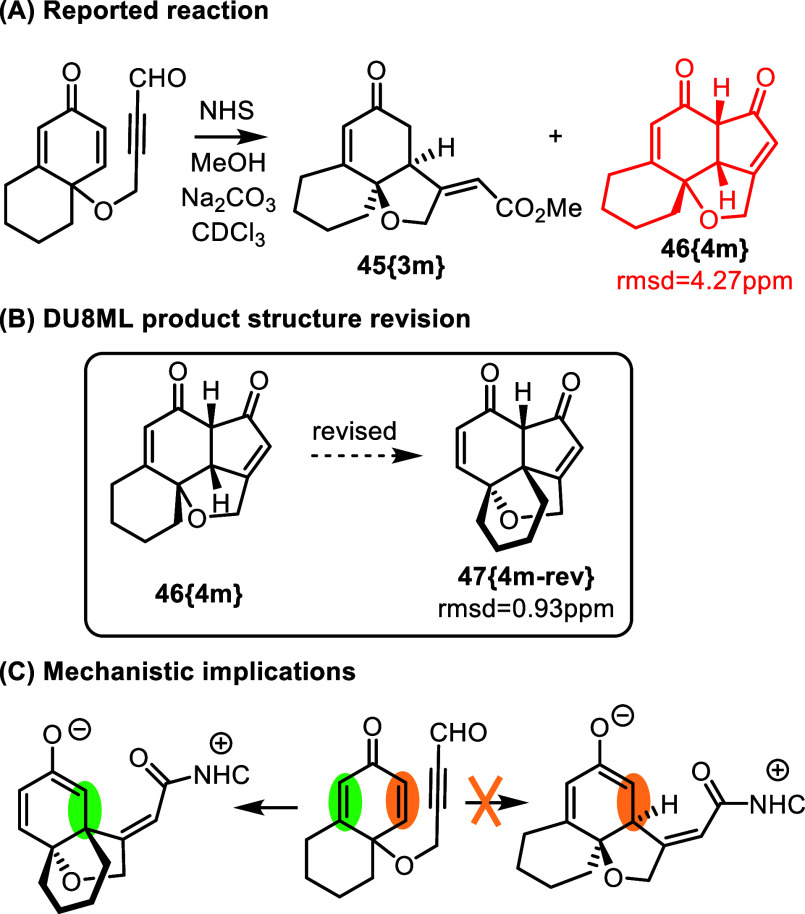
NHC-catalyzed asymmetric cyclization.

Previous report from the same group concerns halogenation of similar fused enones,^22^ Figure 8. One observation which motivated us to check structure assignment was the reported alkenic CH = CH moiety with *J*_HH_ = 10.4 Hz. DU8ML computations also disagreed with the originally assigned structures **48­{4}** and **49­{5}**. We revised them to isomers **50­{4**-**rev}** (crmsd = 0.98 ppm)^23^ and **51­{5**-**rev}** (rmsd = 1.06 ppm). The calculated alkenic *J*_HH(calc)_ = 10.4 Hz closely matched the experimental constant. Mechanistically, this makes sense, as the more strained (and substituted) cyclopentenone moiety is expected to be more reactive.

**Figure 8:**
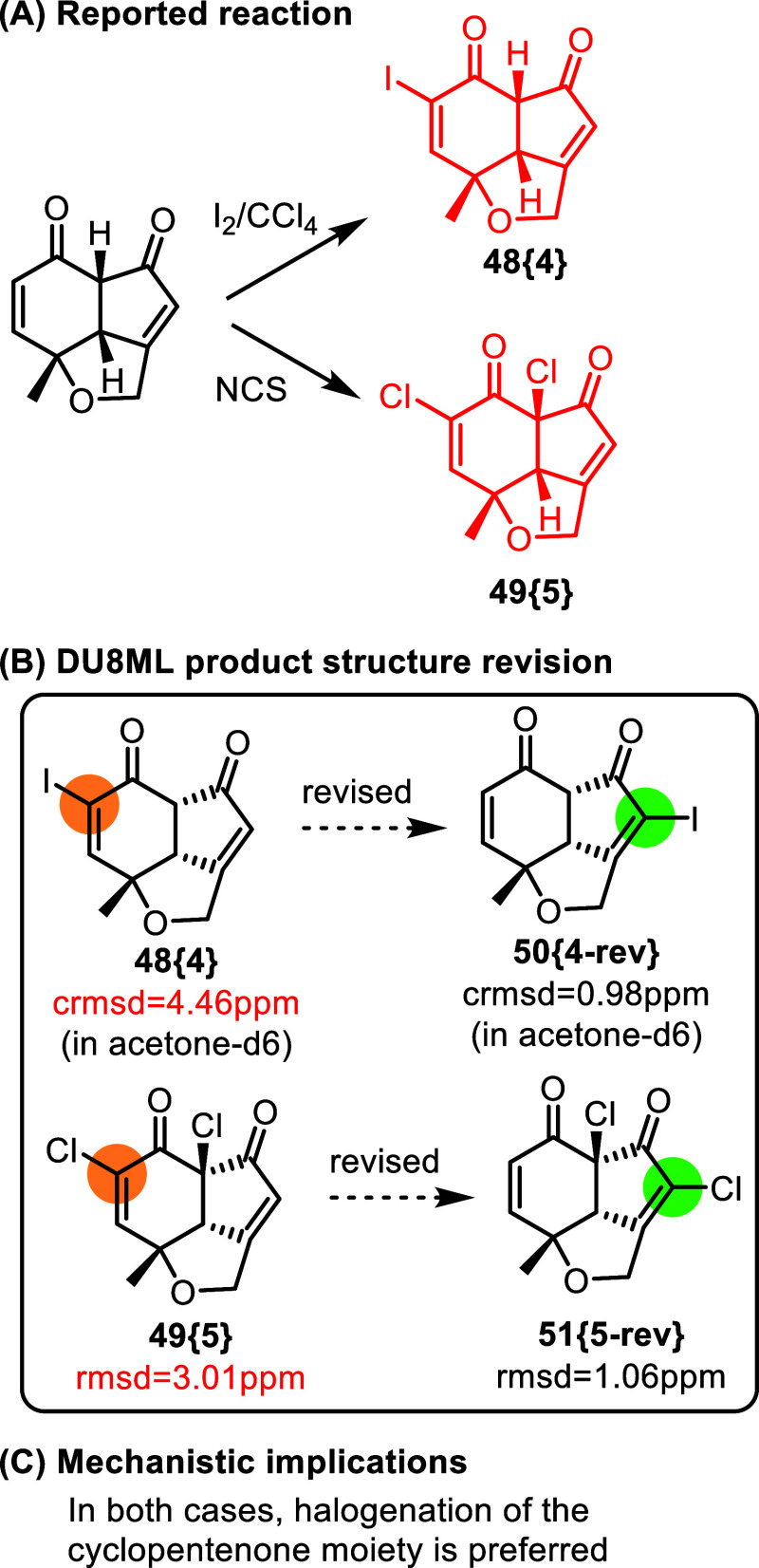
Halogenation of fused enones.

A new synthetic route to the vast collection of 1,3,4-benzotriazepinones **53­{3}** was developed via a base-catalyzed reaction of isatoic anhydrides **52­{1}** with precursors of azomethine imine ylides, i.e. hydrazonyl chlorides, Figure 9.^24^ This extensive study was justified as targeting privileged scaffolds. The authors note that as it was with benzodiazepinones, benzotriazepinones were also suspected of “extremely intriguing pharmacological activities.”

**Figure 9:**
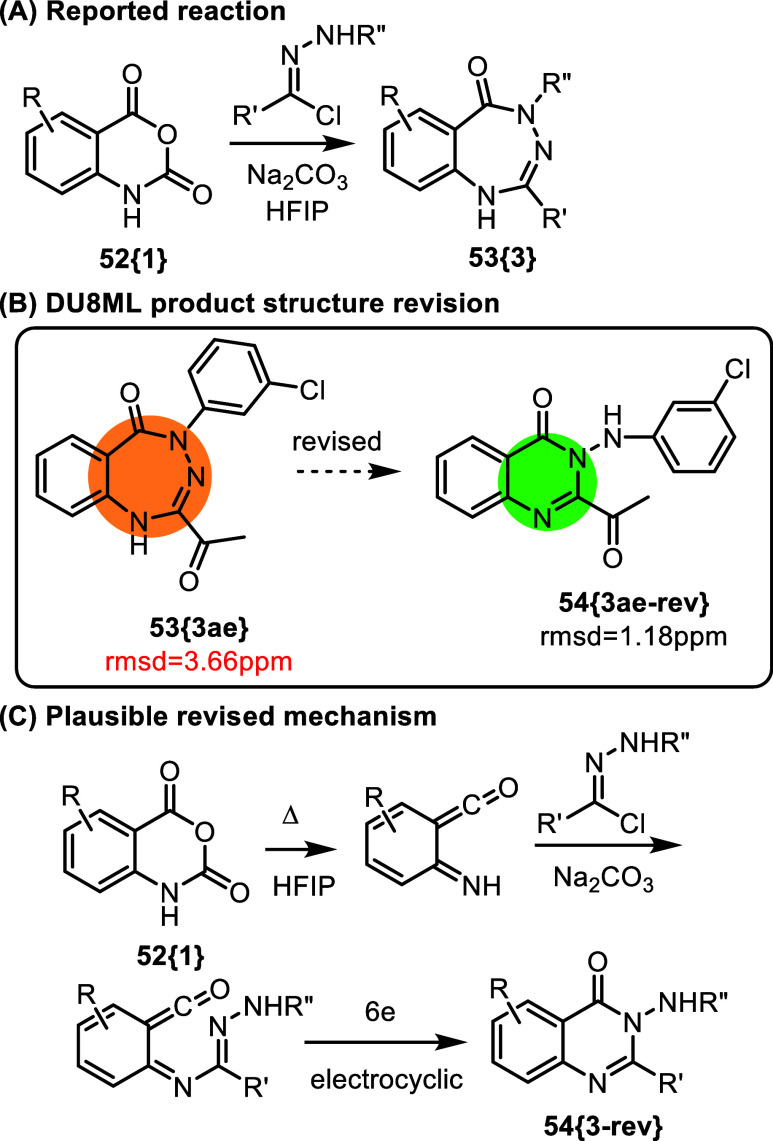
Synthesis of putative benzotriazepinones.

The authors exemplified the broad scope of this reaction by synthesizing 35 benzotriazepinones and demonstrated the potential utility of this reaction with subsequent transformations such as Suzuki cross-coupling. However, DU8ML calculations were in poor agreement with the proposed triazepinone structures. We found that all products needed revision to *N*-aminoquinazolinones **54­{3**-**rev}**.

The most informative ^13^C peak in the series belongs to the carbonyl of the purported triazepinone core. It appears at approximately 160 ppm in all products **53­{3}**, and is consistent with the quinazolinone structures **54­{3**-**rev}**. For the originally proposed benzotriazepinones this value is calculated to be 6–7 ppm greater. Figure 9C shows a plausible revision of the mechanism, where extrusion of CO_2_ from isatoic acid produces an azaxylylene which reacts with hydrazonyl chloride to yield transient amidine. The amidine then cyclizes into the final aminoquinazolinone **54­{3**-**rev}**. The order of the first two steps could be reversed, i.e. the *N*-imidoylation of isatoic anhydride could happen *before* extrusion of CO_2_. Also, the cyclization step does not need to be a concerted 6e electrocyclic process. Another noteworthy observation: quinazolinones **54­{3**-**rev}** lie more than 7 kcal/mol lower in energy than the corresponding benzotriazepinones **53­{3}**. We also cannot completely rule out the formation of **53­{3}** as transient intermediates which undergo isomerization to **54­{3**-**rev}** in HFIP at 80 °C.

Complex fused heterocycles often present challenge in distinguishing the correct regiochemistry of the ring fusion, mostly because proton spin systems in the molecule are interrupted/separated by the fusion juncture lacking protons. An instructive example for this is compound **58­{4ap}** obtained via a palladium catalyzed cascade cyclization shown in Figure 10.^25^

**Figure 10:**
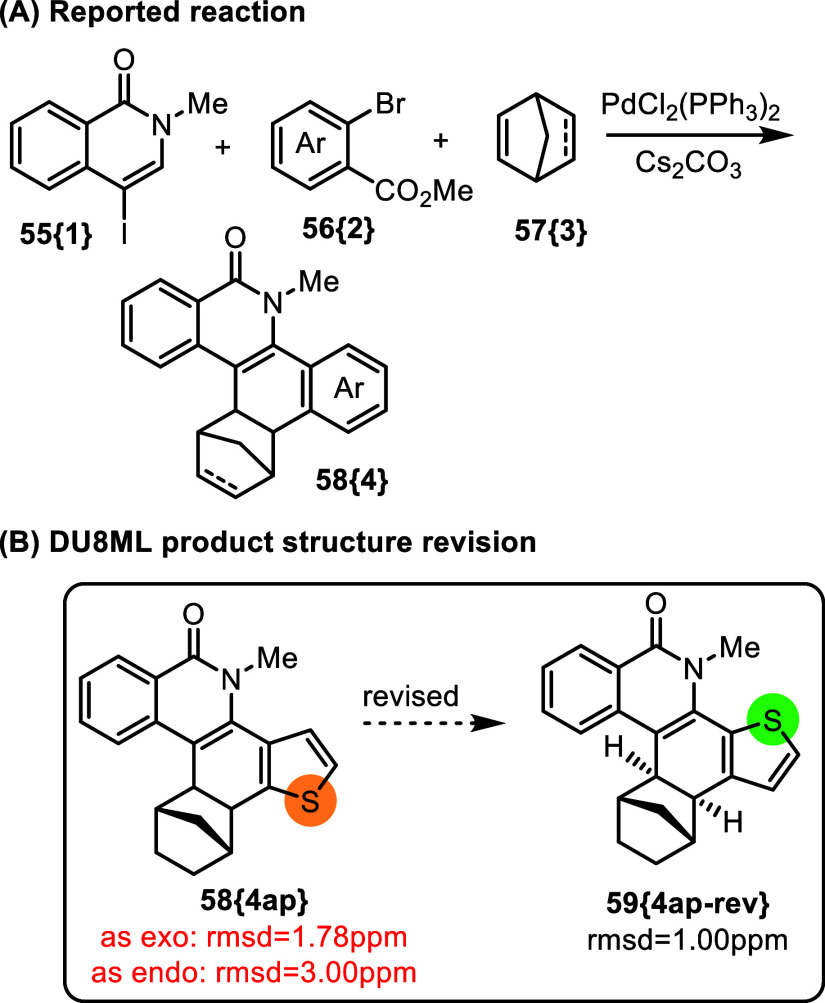
Access to complex fused isoquinolinones.

The product **58­{4ap}** clearly needed revision to its regio-isomer **59­{4ap**-**rev}**. Notice that the stereochemistry of norbornane fusion is not articulated in this case, so we had to consider both the exo- and the endo-diastereoisomers. We do not believe that this revision requires any reassessment of the reaction mechanism. Most likely a wrong isomer of bromothiophenecarboxylate was used as the starting material in this case.

A similar “problem” of the alternative thiophene fusion was on display in the report on isocoumarin synthesis via Rh­(III)-catalyzed CH activation/annulation cascade, Figure 11.^26^ As the case above, this likely reflects a misidentification of the starting material, without any mechanistic implications. Also notice that in this series, **63­{3ra**-**rev}** is a known compound with matching NMR spectra,^27^ supporting our computational revision.

**Figure 11:**
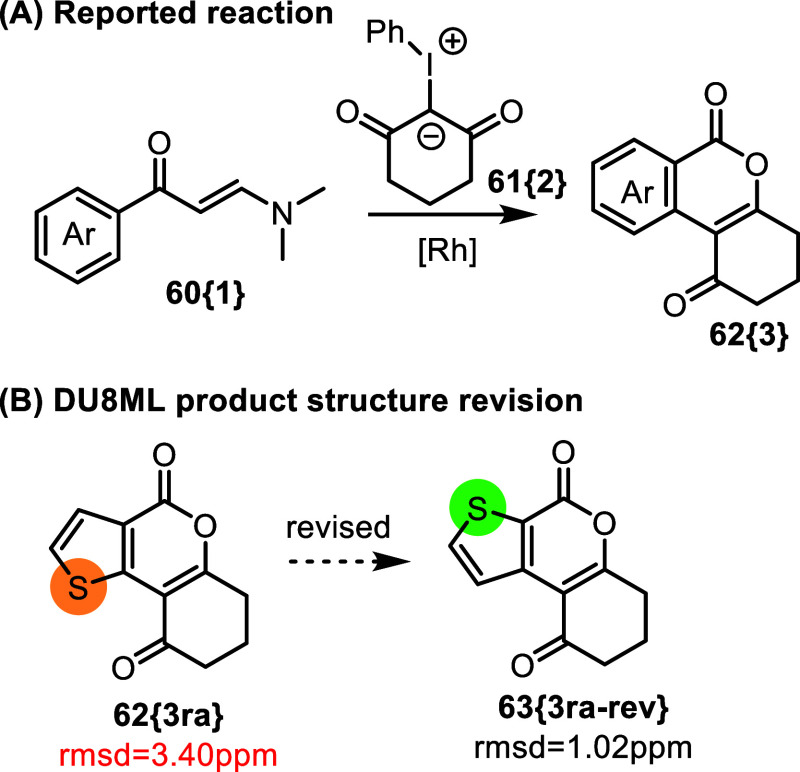
Isocoumarins via Rh­(III) catalyzed CH activation/annulation cascade.

Yet another example of a problematic *upside down* thiophene fusion was likely due to a similar confusion about the starting material. Figure 12 shows access to functionalized cinnolines and their heteroanalogs via copper-catalyzed tandem C–N bond formation in *ortho* bromovinyl-bromoaromatic starting materials.^28^ The majority of the products were in perfect agreement with DU8ML calculations. However, thiophene-containing **67­{11t}** needed revision to its “regio” isomer **68­{11t**-**rev}**. Closer look at the precursor **64­{6t}** revealed that it too requires revision to **69­{6t**-**rev}**. In other words, our revision of **67­{11t}** does not necessitate any mechanistic reconsideration.

**Figure 12:**
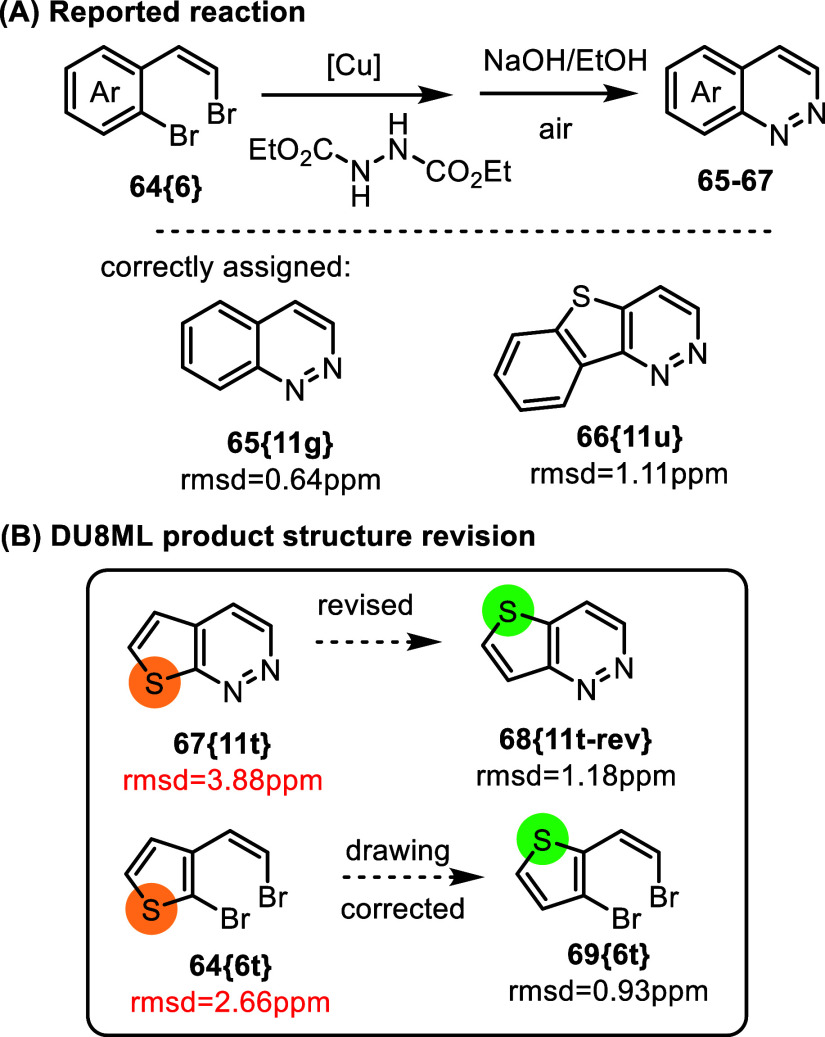
Synthesis of functionalized cinnolines via copper-catalyzed tandem C–N bond formation.

Similar revision necessitated by DU8ML computations is presented in Figure 13. It shows that proton-sparse heterocycles challenge affects not only thiophenes but also fused indoles.

**Figure 13:**
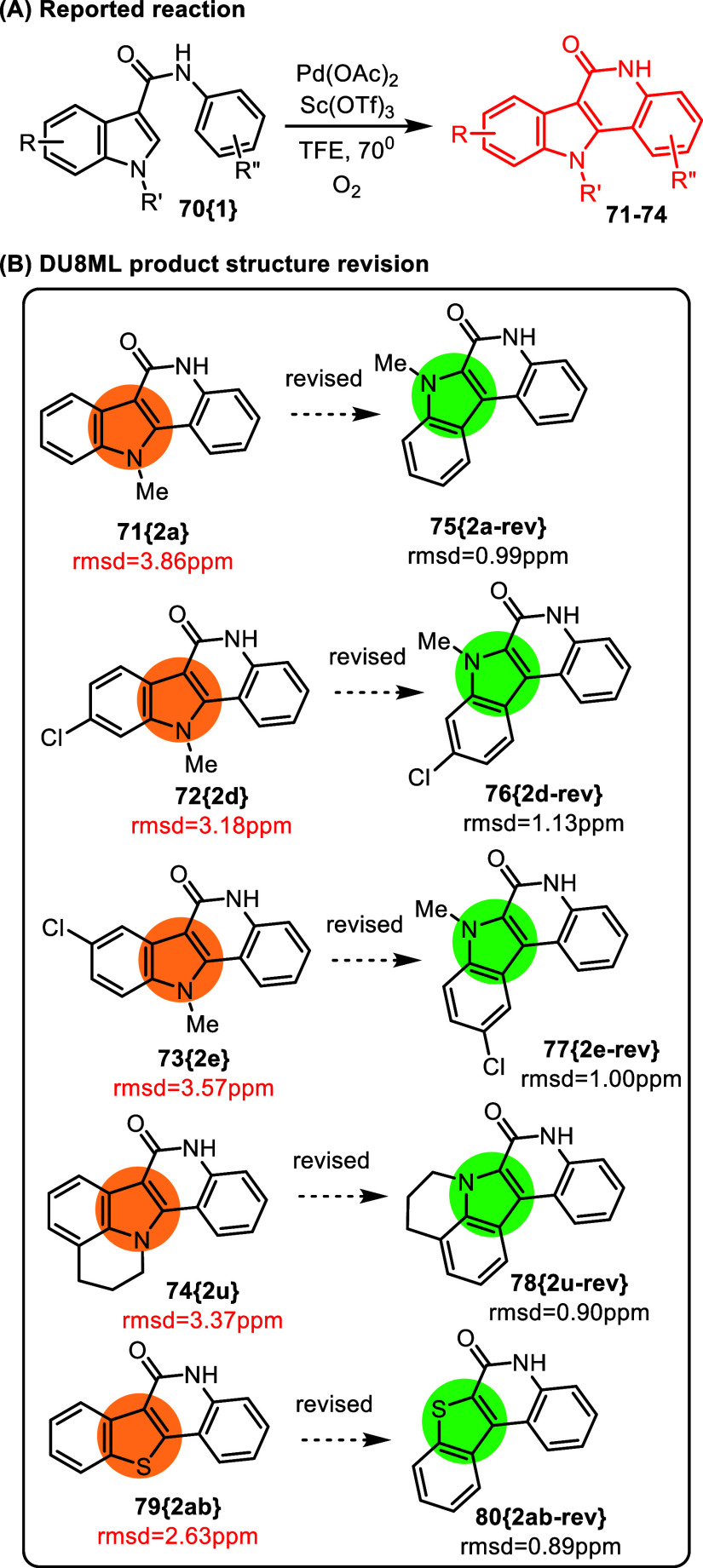
Pd­(II)/Lewis acid catalyzed annulation of indolecarboxamides with dioxygen via dual C–H activation.

This intramolecular annulation of indole-3-carboxamides **70­{1}** purportedly yielded indoloquinolinones **71**–**74** as exemplified using a rather broad range of substrates.^29^ Further, the reaction scope was extended to benzothiophenes **79­{2ab}**. The results of DU8ML computations were not in agreement with the experimental data. All 5,11-dihydro-6*H*-indolo­[3,2-*c*]­quinolin-6-one structures **71**–**74­{2}** needed revision to 5,11-dihydro-6*H*-indolo­[2,3-*c*]­quinolin-6-ones **75**–**78**. The same applies to benzohiophene **79­{2ab}**. One of the revised structures is a known compound: **75­{2a**-**rev}** and **80­{2ab**-**rev}**.^30^ A review of the literature also revealed an opportunity to compare the two sets of regioisomers. It was demonstrated that under similar conditions, both indole-3-carboxanilides and indole-2-carboxanilides form indolo­[3,2-*c*]­quinolin-6-ones or indolo­[2,3-*c*]­quinolin-6-ones, respectively.^31^ In that study, *N*-methyl substituted quinolinones were synthesized, offering a not-quite-exact comparison for compounds **71**–**74­{2}**, possessing unsubstituted quinoline nitrogen. Still, as shown in Figure 14, pairwise comparison of the aromatic ^13^C chemical shifts (including indole’s NMe) for **71­{2a}** and **81­{17}**/**82­{18}** provided additional experimental support that **71­{2a}** requires revision to **75­{2a**-**rev}**.

**Figure 14:**
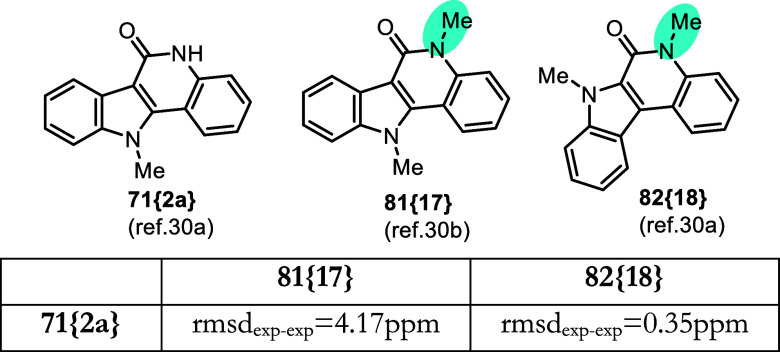
Comparison of experimental ^13^C data for indolo-quinolinone products **71­{2a}** and **81­{17}**/**82­{18}**.

Functionalized benzotropones were accessed via a one-pot phthalide ring-opening/intramolecular aldol condensation cascade and introduced into subsequent transformations.^32^ Figure 15 shows FeCl_3_-catalyzed [4 + 2] cycloaddition of one of these benzotropones, **83­{3aa}**, with cyclopentadiene yielding polycycle **84­{8}**.

**Figure 15:**
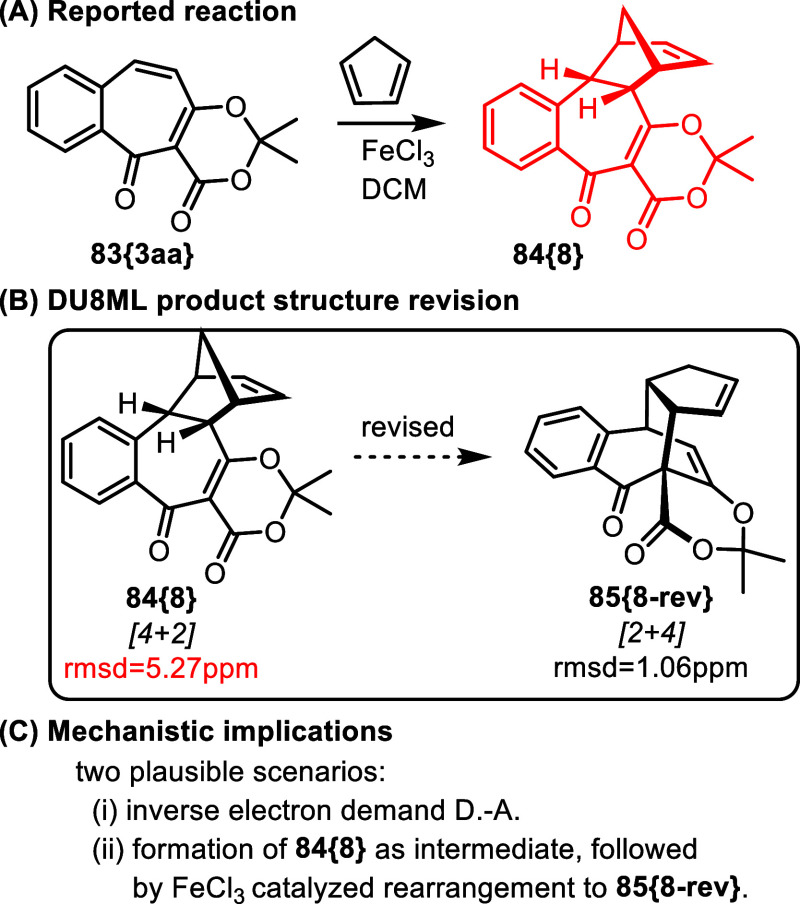
Access to functionalized benzotropones and their subsequent reactions.

^13^C values calculated with DU8ML disagreed with experimental data for **84­{8}**, which we eventually revised to [2 + 4] product **85­{8**-**rev}**. In this formal 6e-cycloaddition the 4π component is the benzotropone itself, whereas cyclopentadiene acts as a 2π component. It is possible that **84­{8}** is formed as a transient intermediate which in the presence of FeCl_3_ rearranges into **85­{8**-**rev}**. The latter is more than 11 kcal/mol downhill of the former, according to B3LYP/6–31G­(d). It is also conceivable that **85­{8**-**rev}** is formed as a result of a concerted inverse electron demand Diels–Alder reaction.

The revised (**85­{8**-**rev}**) and original (**84­{8}**) structures are formally related via allylic 1,3-shift. Another example of this allylic regio-isomerism, with an added stereochemical challenge, is shown in Figure 15. A series of indeno­[2,1-*c*]­chromenes **86**–**88** were accessed via bismuth­(III)-catalyzed cascade reaction of propargylic *para*-quinone methides and 2-vinylphenol.^33^ However, the majority of products reported in this series required revision to allylic isomers **90­{3a**-**rev}**. As shown in Figure 16B, DU8ML computations for the revised structures gave an excellent match with experimental data. Yet, a few products, for example **86­{3k}** shown in Figure 16A, do have the structures originally proposed by the authors. As Figure 16C shows, the last step in the mechanism proposed by the authors involves “diastereoselective isomerization” to products **86**–**88**. The DFT energies seem to be in keeping with this direction of isomerization as the final **86**–**88** structures are slightly downhill. Still, even with this thermodynamic bias, it appears that for the majority of isolated products, the last isomerization step does not occur and the reaction stops at structures 90–**91**.

**Figure 16:**
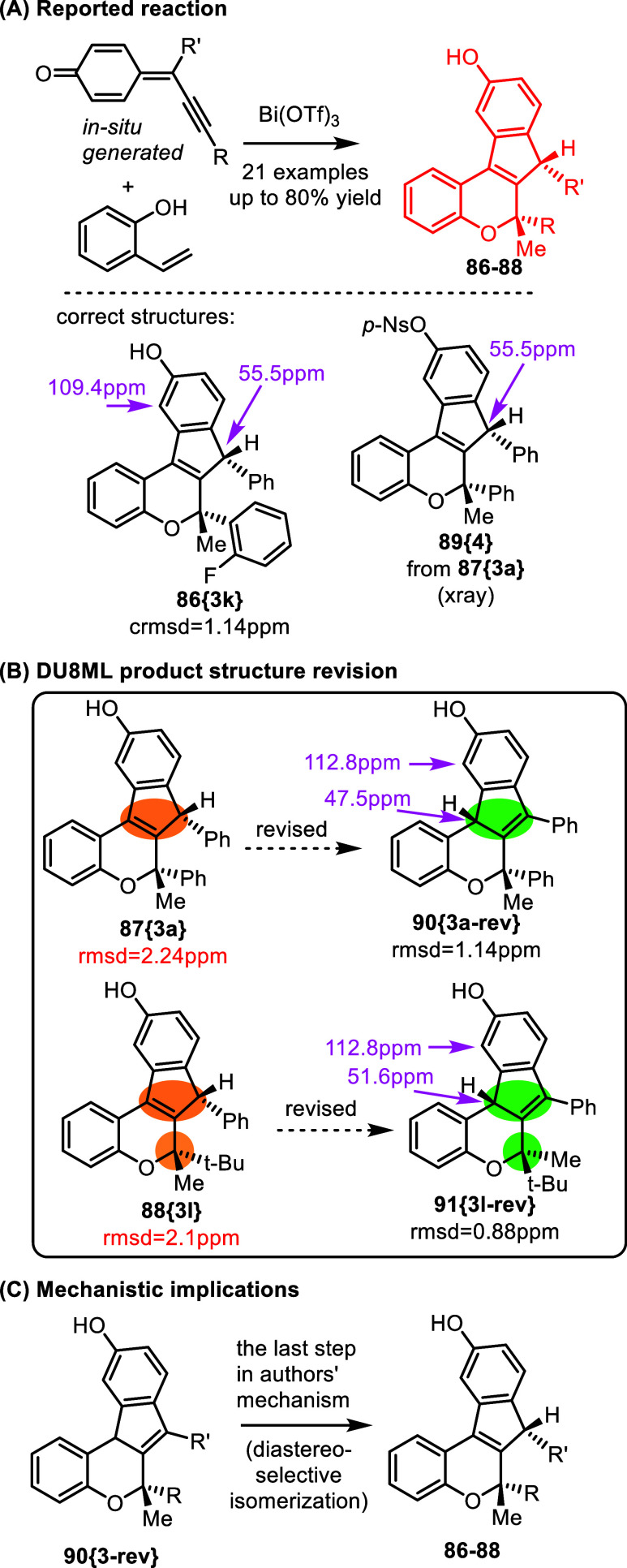
Bismuth­(III) catalyzed synthesis of indeno­[2,1-*c*]­chromenes.

A special case of allylic isomerism, which has pronounced mechanistic ramifications, is the [4 + 2] vs [2 + 2] dichotomy in cycloadditions of fluorinated alkenes. Examples of difluorinated methylenecyclopropanes cycloadditions with cyclopentadiene (CPD) are shown in Figure 17.^34^ The authors studied both gem-difluoro methylenecyclopropanes, possessing the CF2 = moiety (as in **95­{2}**) or the CF2 group in the cyclopropane ring (**92­{1}**). It was concluded that difluoromethylenecyclopropane **95­{2}** reacted with CPD via a [2 + 2] cycloaddition, which was rationalized with extended Hückel calculations. Nonetheless, according to DU8ML, product **96­{13}** needed revision into norbornene **97­{13**-**rev}**. Mechanistically, this result confirms that reactivity of fluoroalkenes do not conform to simple first-order symmetry rules, and in order to predict the outcome of cycloaddition reactions with fluorinated alkenes one is advised to use caution (and computations at a higher level of theory).

**Figure 17:**
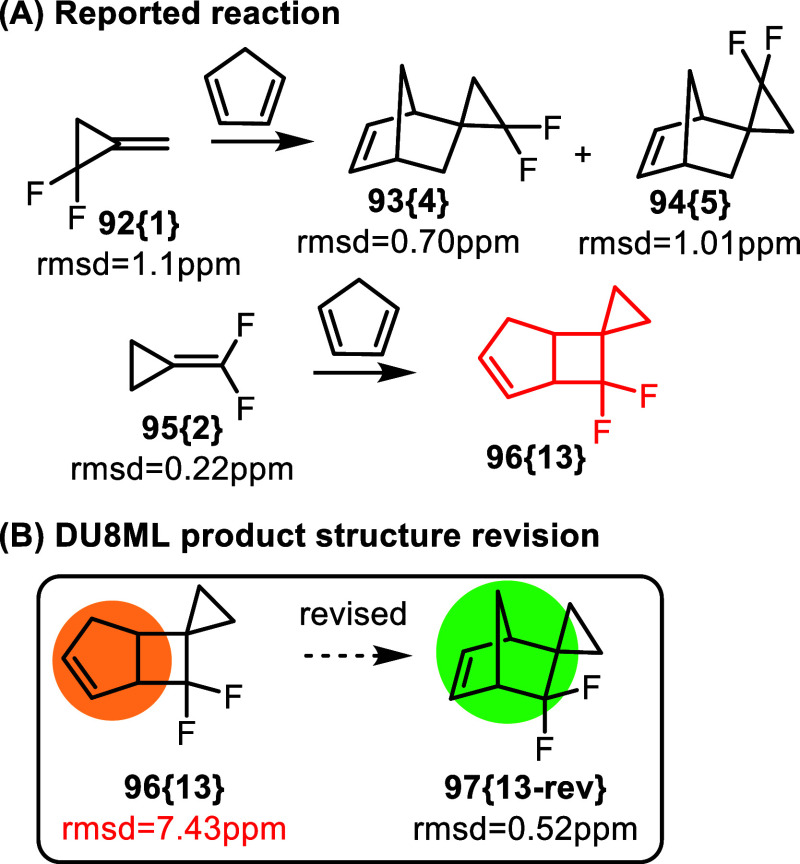
[4 + 2] vs [2 + 2] cycloadditions of fluorinated methylenecyclopropanes.

In a recent report on asymmetric organocatalyzed cascade reactions, primary targets, acrylates **98­{4}**, were further synthetically elaborated into photorearranged cyclopentenes 99-100, Figure 18.^35^ However, these photoproducts **99**–**100** were not in agreement with DU8ML computations. One obvious discrepancy was the presence of alkenic CH = CH moiety as implied by the ^1^H NMR possessing the vicinal spin coupling constant, ^3^ *J*_HH_ = *11* Hz, not compatible with the cyclopentene ring, but rather pointing to a larger alicycle. Revision to bicyclo[3.2.2]­nonadienes **101­{5a**-**rev}** and **102­{5g**-**rev}**, improved the ^13^C match (rmsd’s 5.74 ppm → 1.38 and 5.48 ppm → 1.43 ppm respectively) and satisfied the vicinal proton spin coupling constant for the alkenic CH = CH moiety, calculated at ^3^ *J*_Calc_ = 10.9 Hz.

**Figure 18:**
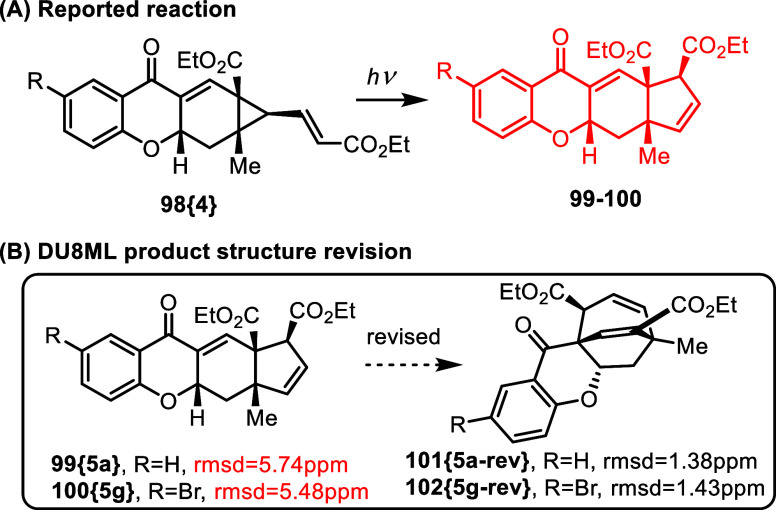
Photorearrangement of **99**–**100**.

Visible-light-mediated regio-convergent [2 + 2]/[3 + 2] cycloadditions were developed to access aza-β-lactams and γ-fused lactam derivatives,^36^ Figure 19. The reaction involves the Wolff rearrangement, with the transient ketene undergoing [2 + 2] cycloaddition to azoester **103­{6}**. Structure of aza-β-lactam **106­{7a}** was elucidated with single crystal X-ray diffractometry. The rest of the products were assigned the same regio-isomeric structure, i.e. the one where azalactam’s carbonyl group is attached to the carboxylate bearing nitrogen atom. Yet, DU8ML pointed to discrepancies in computed and experimental data for several products, for example, **107­{7c}**, **108­{7e}**, **109­{7j}**, which required revision to the opposite regio-isomers. The same revision was needed for the para-nitro product **106­{7a}**, despite the availability of X-ray structure for its purported regio-isomer. The major regio-isomer purified and characterized by NMR has the structure of **110­{7a**-**rev}**. It is likely to be a rare case where the minor regio-isomer **106­{7a}** was accidently crystallized and subjected to single crystal X-ray diffractometry.

A recent study of the photo-Fries rearrangement in *N*-acetyl indoles and other benzo-fused heterocycles revealed a variety of substrate-dependent outcomes, i.e. acetyl migrations,^37^ Figures 20.

**Figure 19:**
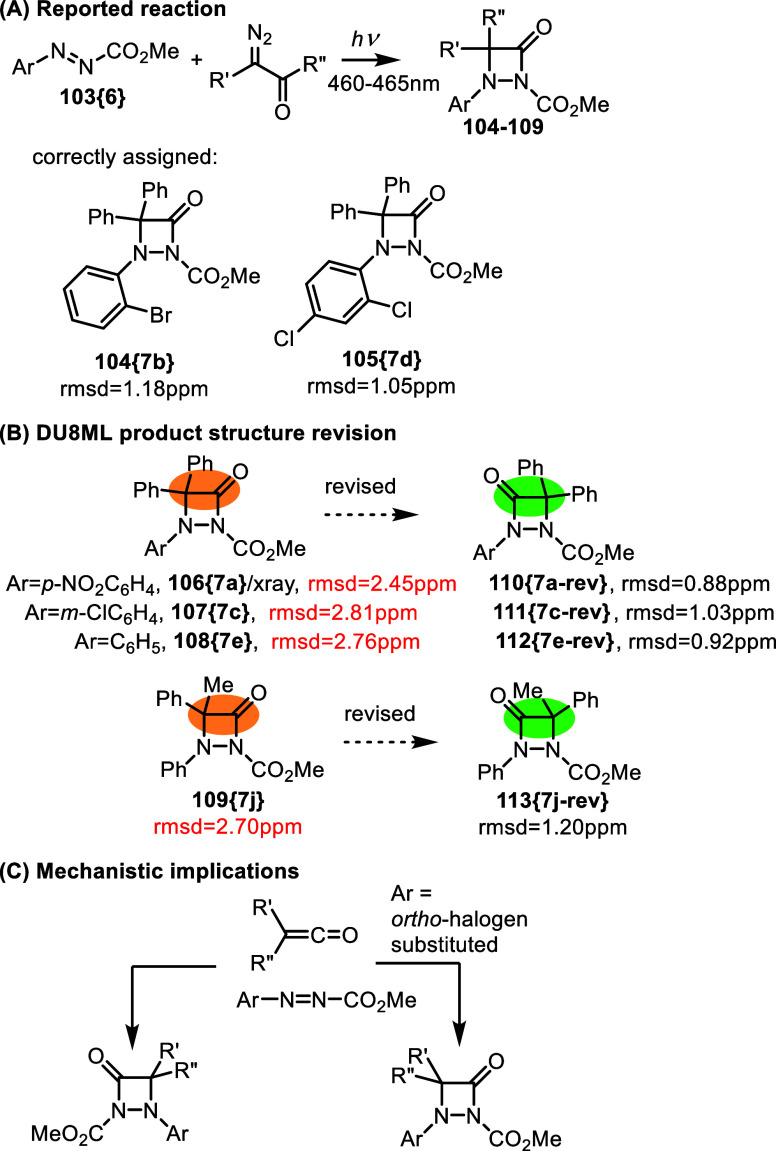
Access to aza-β-lactams.

**Figure 20:**
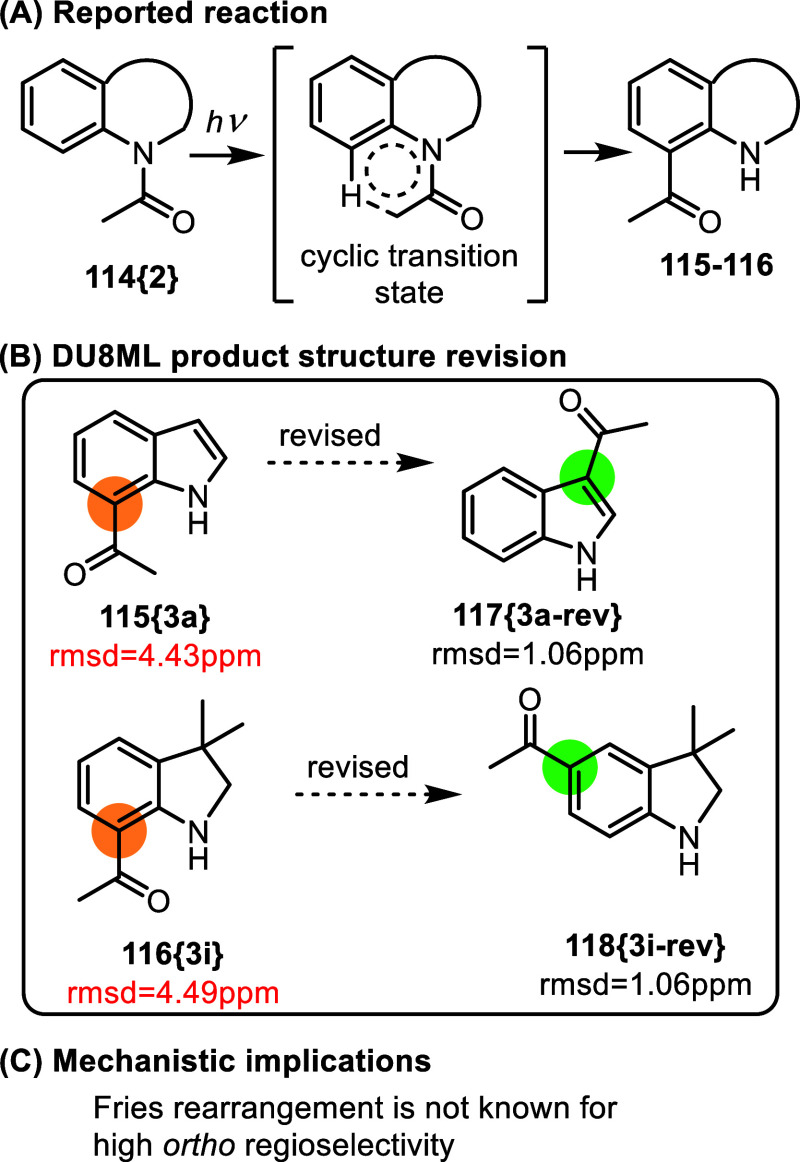
Photo-Fries rearrangement.

Besides the unorthodox depiction of the cyclic transition state (reproduced in Figure 20A copying authors’ style), many products had correct structure assignments. However, several of the purported products of acetyl migration to position 7 of indole (or equivalent in other heterocyclic substrates) needed revision. Because of impurities in spectra, we were able to revise only products **115­{3a}** and **116­{3i}** with sufficient confidence (additionally, **117­{3a**-**rev}** is a known compound with matching NMR spectra^38^). At least two other products clearly needed correction, but poor quality of the spectra prevented us from proposing their revised structures.

Diastereomer interconversion via photoinduced electrocyclic ring opening/closure in derivatives of androstenediones offered facile access to analogues of abiraterone.^39^ The fact that abiraterone and its congeners have been used in cancer treatments added to the motivation for this study. Given the low dispersion of signals in proton spectra, the structure elucidation work was challenging, so it was not unexpected that some of the structures needed revision, Figure 21.

**Figure 21:**
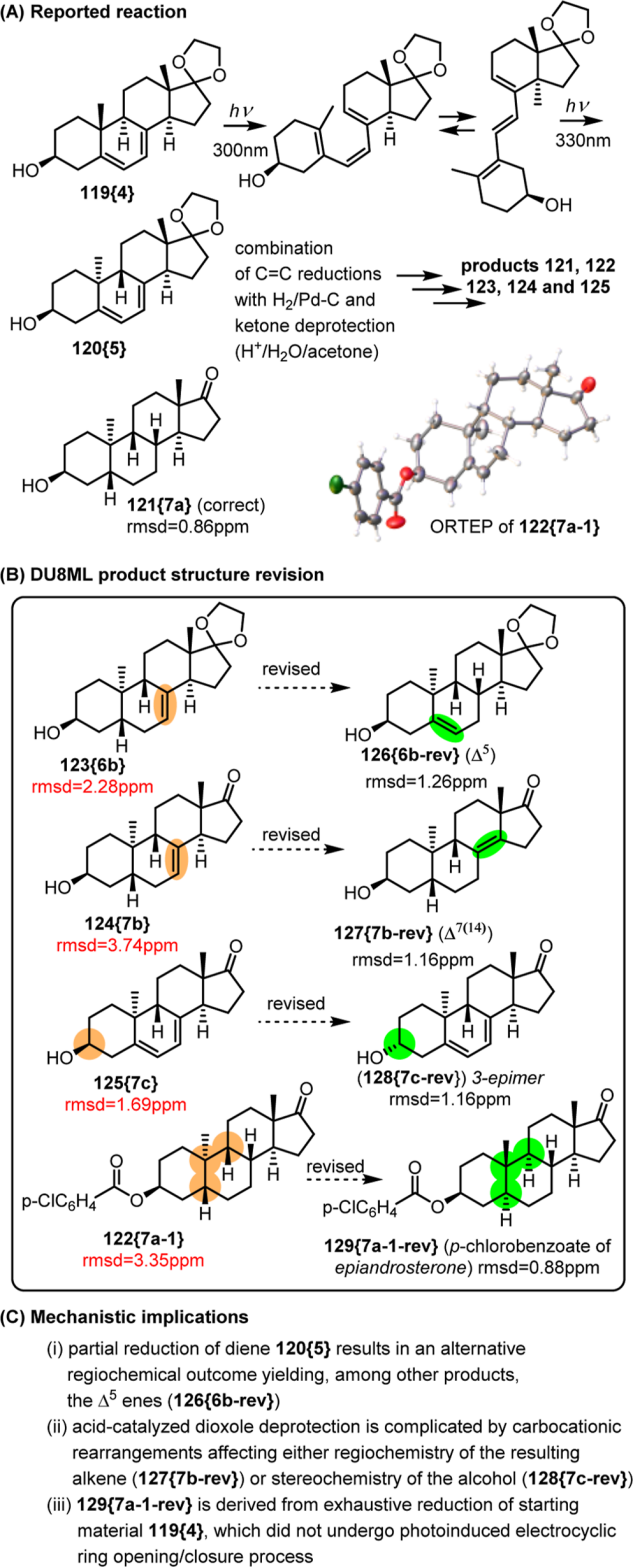
Photoassisted access to abiraterone derivatives.

The structure of the product of exhaustive hydrogenation/deprotection, **121­{7a}**, was determined correctly by the authors, rmsd­(δ_13C_) = 0.86 ppm. The product of partial hydrogenation, **123­{6b}**, required revision to Δ^5^ ene **126­{6b**-**rev}**. Putative **123­{6b}** was further deprotected with TsOH in aqueous acetone yielding **124­{7b}** which also needed revision to **127­{7b**-**rev}**. Given the revised structure of **126­{6b**-**rev}**, acid-catalyzed deprotection of it caused carbocationic rearrangements with the alkenic moiety migrating from Δ^5^ to Δ^7(14)^. Related problem occurred with diene **125­{7c}**, where homoallylic carbocation’s involvement plausibly facilitated epimerization of alcohol at C3, yielding 3-epimer **128­{7c**-**rev}**.

While DU8ML confirmed the structure of **121­{7a}** as correct, there was a problem with its *p*-chlorobenzoate, **122­{7a**-**1}**. According to NMR, this *p*-chlorobenzoate has a fully saturated steroidal core, but the ORTEP structure clearly shows the Δ^5^ ene. This likely reflects a trivial mix-up of sorts. Yet, it is important to note that according to DU8ML analysis, the saturated structure of **122­{7a**-**1}** as drawn by the authors, needs revision to **129­{7a**-**1**-**rev}**, i.e. the derivative of epiandrosterone. Compound **129­{7a**-**1**-**rev}** was not reported in the literature. However, ^13^C NMR data for a similar derivative–the unsubstituted benzoate of epiandrosterone–is available.^40^ Its 18 aliphatic ^13^C peaks match perfectly with peaks reported for putative “**122­{7a**-**1}**,” rmsd_exp‑exp_ = 0.21 ppm. Mechanistically, this means that **122­{7a**-**1}** is not derived from reduction of **120­{5}**, but rather resulted from the reduction of starting **119­{4}**, which did not undergo the title process of photoinduced electrocyclic ring opening/closure.

Finally, it is evident that instead of fully saturated steroid, it was *p*-chlorobenzoate of **126­{6b**-**rev}** that was mistakenly synthesized/isolated and subjected to X-ray diffractometry. This misidentification and the X-ray structure further confirm the validity of our DU8ML-driven revision of **123­{6b}** to **126­{6b**-**rev}**.

This challenge of a misplaced double bond seems to be rather common in literature. For example, an extensive work toward synthesis of aconitine analogs dealt with challenges of structure elucidation in rather complex polycyclic intermediates, Figure 22.^41^ The ultimate “analogue,” **131­{26}**, appears to be a more severe case of a misplaced double bond, as the revision necessitated deconjugation of the 1,3-diene moiety in favor of the enamide functional group in the revised structure **132­{26**-**rev}**. DU8ML calculations revealed significant general discrepancies between the computed and experimental ^13^C data (rmsd = 6.03 ppm) for the proposed structure, but also pointed to most severe deviations: the calculated value for the enamide = CH in **132­{26**-**rev}** was within 0.5 ppm of the experimental, but the same carbon in the original structure **131­{26}** was off by 14.2 ppm.

**Figure 22:**
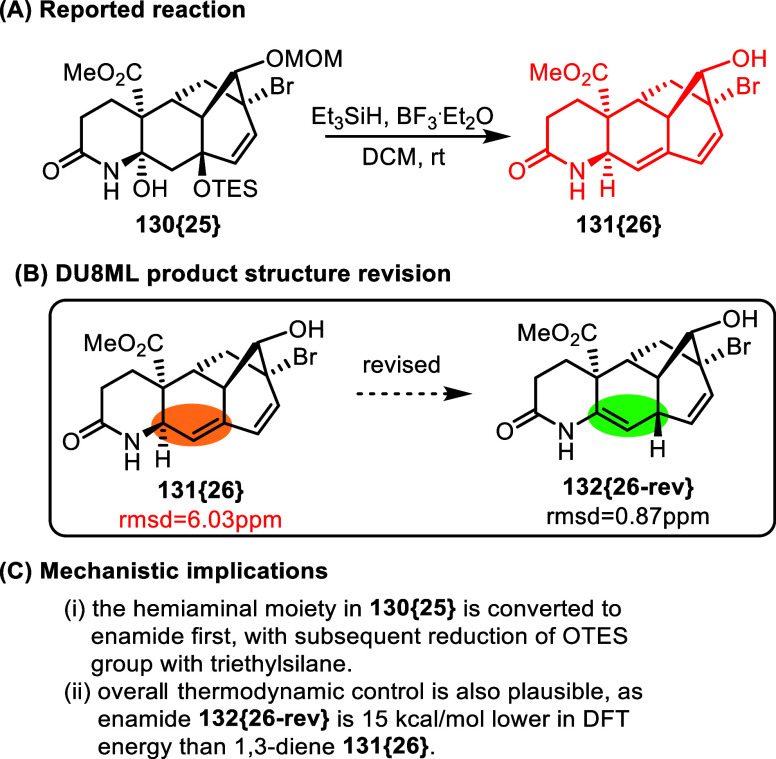
Synthesis of aconitine analogs.

Figure 23 illustrates another common challenge with structure elucidation in aroyl-substituted five-membered heterocycles, which lack protons where it matters, and therefore prone to misassignment. The authors reported a new three-component reaction to access fluorescent 1,4-benzothiazin-2-ones as shown,^42^ and proposed a plausible mechanism, which starts with an electrophilic attack of elemental sulfur on anisidine. Almost 20 products were synthesized and their photophysical properties, including fluorescence quantum yields, were explored both experimentally and with the help of time dependent (TD) DFT. Yet, our DU8ML computations rejected all originally proposed structures, which gave poor matches between the experimental and computed ^13^C chemical shift values (rmsd >5.6 ppm). After a few guesses on potential mechanisms, we arrived at the conclusion that all product structures need revision to 2-aroylbenzothiazoles. Figure 23 shows a subset of revised products containing a variety of Ar-groups, from substituted phenyl, to thiophene, furan, or pyridine–all exhibiting an excellent match of computed and experimental chemical shifts.

**Figure 23:**
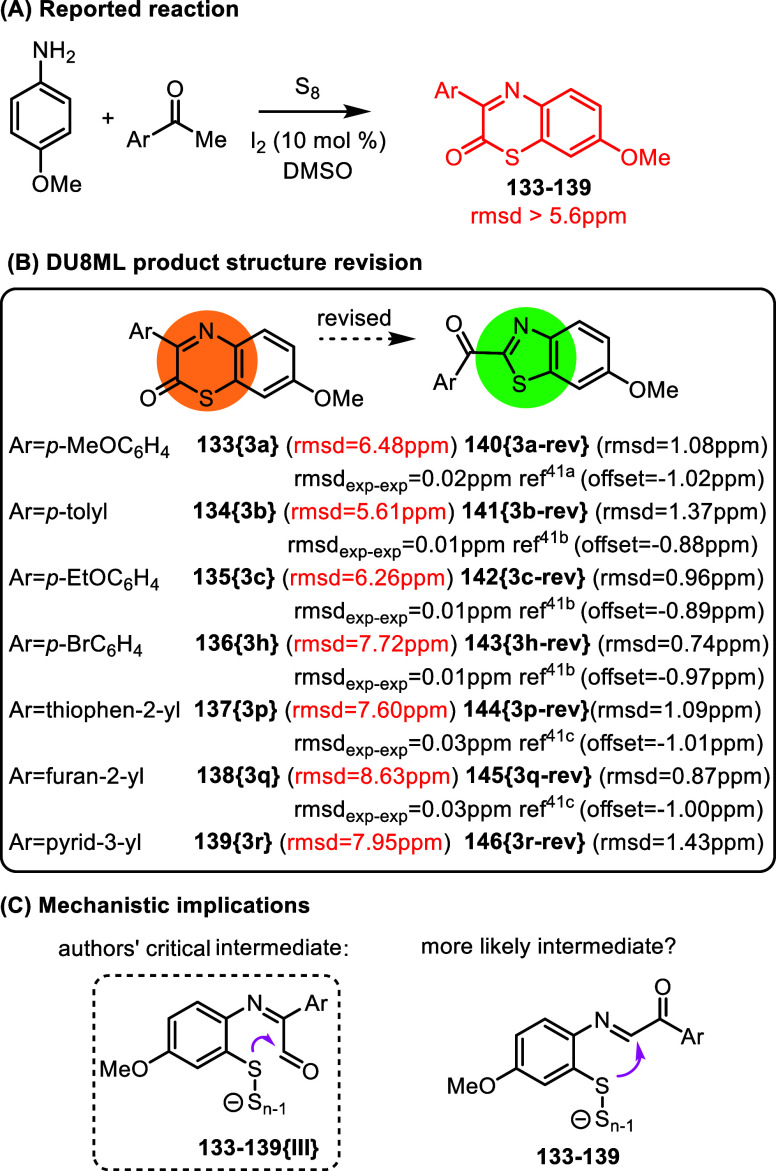
Synthesis of heterocyclic fluorophores.

Mechanism: the authors propose a key intermediate, **133**–**139­{III}**, plausibly arising from the Swern oxidation of the α-iodinated acetophenone in its imine form. However, it is conceivable that the Swern oxidation could occur earlier in the reaction. In that scenario, an alternative imine intermediate, **133**–**139**, might be operational, leading to revised thiazoles **140**–**146**.

As in the above cases, this product revision was driven computationally with the help of DU8ML. In this particular case, search of the literature revealed that most of these 2-aroyl-benzothiazoles are known compounds, giving additional support to our in silico revisions.^43^ For the previously described aroyl-benzothiazoles we calculated the rmsd_exp‑exp_ values, which are added to Figure 23B. One minor problem, which we also address in more detail later in this paper, is that the referencing of NMR spectra is often inconsistent across different research groups. To properly compare two experimental data sets and calculate accurate rmsd_exp‑exp_ values we needed to introduce a systematic offset which, in this series, amounted to approximately –1 ppm. The listed rmsd_exp‑exp_ values are based on the data corrected with such systematic offsets (also listed in Figure 23B). With this correction, rmsd_exp‑exp_ values were within the range of 0.01–0.03 ppm, imparting confidence that our DU8ML-driven in silico revisions are indeed fully warranted.

We also identified instances of “reversed confusion,” where six-membered thiazines were mistaken for five-membered thiazoles in similarly proton-deficient heterocycles. For example, a phosphine-catalyzed synthesis of sulfur heterocycles from arylpropiolates–as dielectrophilic synthons–and 1,4-dinucleophiles yielded *six*-membered oxathianes or thiomorpholines, for example **148­{1b}** and **149­{1f}**, Figure 24, via the shown vinylphosphonium intermediate.^44^ However, aromatic 1,3-dinucleophiles, for example 2-mercaptoimidazoles or benzoimidazoles, elicited a different reactivity. DU8ML helped recognize this dichotomy and we revised products **150­{1g},** **151­{1h}** and **153­{1i}** to six-membered thiazinones **154­{1g**-**rev}**, **155­{1h**-**rev}**, and **156­{1i**-**rev}**. Two of these, **154­{1g**-**rev}** and **155­{1h**-**rev}**, are known compounds.^45^

**Figure 24:**
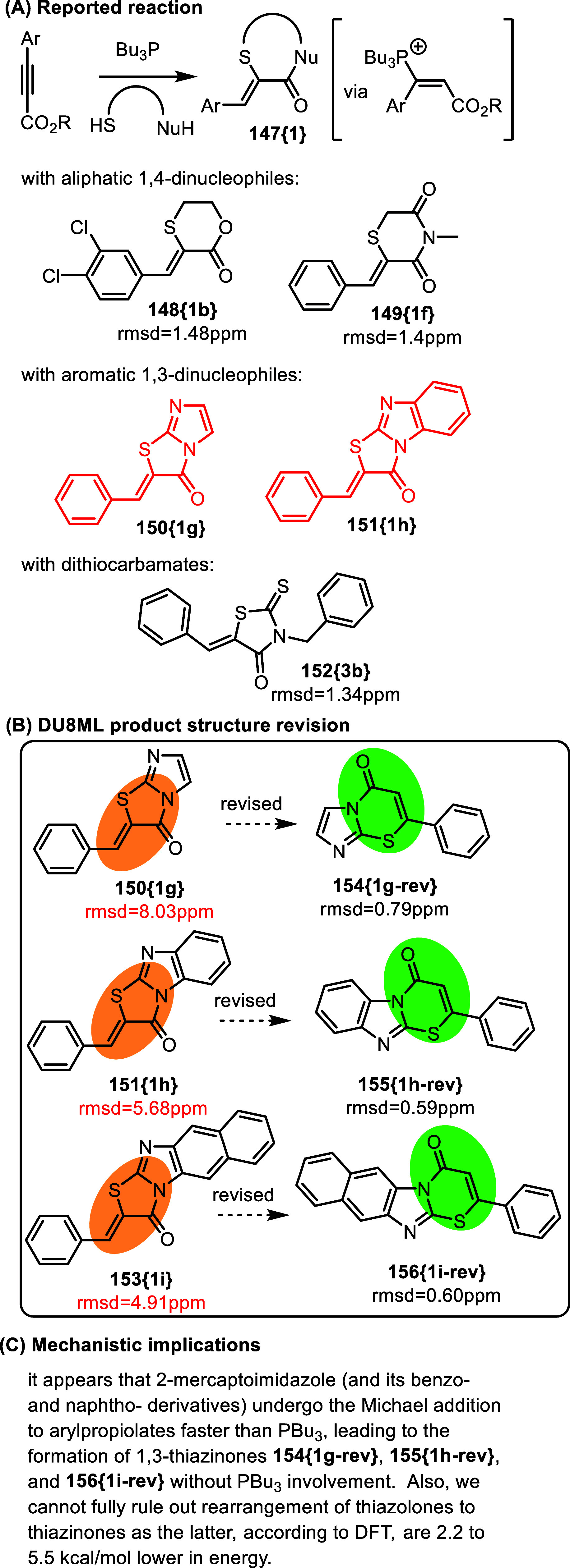
Phosphine-catalyzed construction of sulfur heterocycles.

Curiously, reaction with dithiocarbamates, formed in situ from CS_2_ and primary amines, produced five-membered 2-thioxothiazolidin-4-ones such as **152­{3b}** (structures were correctly assigned), i.e. with dithiocarbamates, arylproiolates acted as formal 1,2-dielectrophiles. Mechanistically, this implies that mercaptoimidazoles were able to outcompete tributylphosphine as *S*-nucleophiles, adding directly to arylpropiolates. We also cannot fully rule out formation of thiazolones and their subsequent rearrangement to thiazinones **154**–**156** as–according to DFT–they are 2.2–5.5 kcal/mol lower in energy.

A recent communication disclosed a shelf-stable C_2_O synthon, Ph_2_SCCO, which the authors engaged with α,β-unsaturated esters containing a tethered nucleophile to access complex cyclopropane-fused pyrrolidones, piperidones, and γ-valerolactones (Figure 25).^46^ These are impactful findings, as there is every reason to believe that this stable heterocumulene will find broad application in synthetic chemistry.

**Figure 25:**
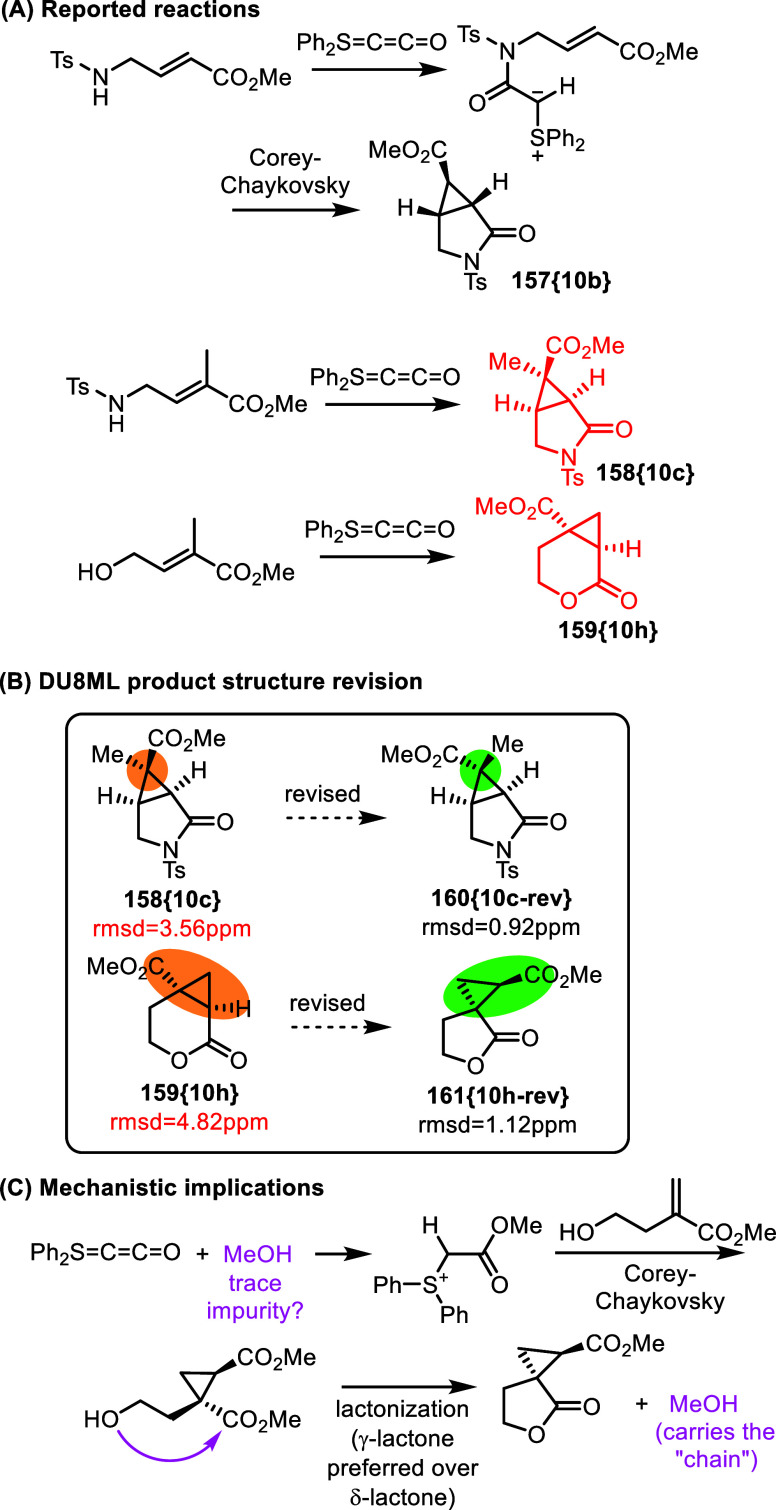
Stable C_2_O synthon, Ph_2_SCCO, and its Corey–Chaykovsky reactions with α,β-unsaturated esters containing a tethered nucleophile.

The authors proposed a plausible mechanism in which the tethered nucleophile undergoes addition to Ph_2_SCCO, generating a sulfur ylide suitable for intramolecular Corey–Chaykovsky reaction, Figure 25A. Most of the product structures were assigned correctly by the authors. Our revision of product **158­{10c}** to its epimer, Figure 25B, is a minor change, and it does not compel any reconsideration of the mechanism. However, one of the hydroxy-derived products, assigned the structure of cyclopropane-fused δ-valerolactone **159­{10h}**, required revision to spiro-connected γ-butyrolactone **161­{10h**-**rev}**. This particular structure cannot be reconciled with the originally proposed mechanism. A plausible revision of the mechanism is shown in Figure 25C. It involves a trace impurity of an alcohol (or any H-Nuc to that matter) which initiates the “chain” by reacting with the new CCO-transfer reagent to generate the shown sulfur ylide. The ylide is then reacts in a classical Corey–Chaykovsky manner with the α,β-unsaturated substrate to yield the cyclopropane intermediate bearing two carboxylates. This is followed by lactonization, which in this case preferentially produces the γ-lactone.^47^ The released molecule of methanol carries “the chain.”

Revision of compound **159­{10h}** fortuitously provided motivation for mechanism revision in this case, as the actual product, **161­{10h**-**rev}**, cannot be rationalized in terms of the original mechanism. However, either the original or the Figure 25C mechanisms could account for the formation of other compounds in this series. A mechanistic question to explore is which mechanism is operational in other cases? (this is unless the mechanism is substrate-dependent and either of the two could be operational under specific conditions).

### Quasi-Plausible or Implausible Reactions that Never Occurred

In the course of a study focused on transition-metal-free synthesis of indole-substituted indenes, an unusual carbocationic rearrangement was discovered, Figure 26A.^48^

**Figure 26:**
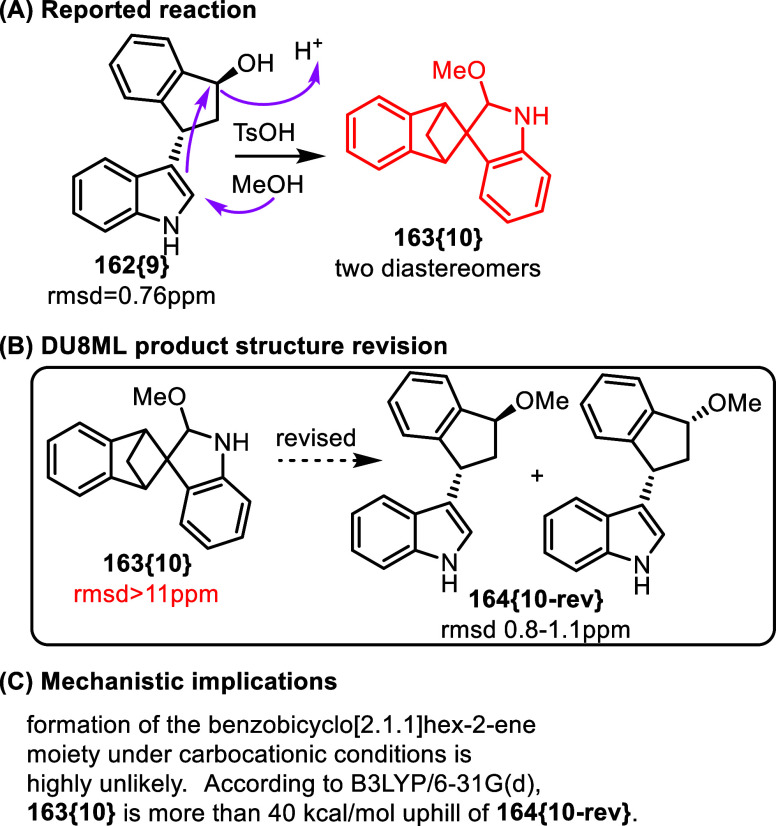
Synthesis and reactions of indole-substituted indenes.

DU8ML results were in strong disagreement with the putative structure **163­{10}** which purportedly contained a benzobicyclo[2.1.1]­hexene moiety spiro-connected to indoline (rmsd >11 ppm). Upon further investigation, it was apparent that the starting indanol **162­{9}** underwent an S_N_1-type etherification in methanol under acidic conditions. The two diastereomers possess very similar spectra, making it impossible to differentiate them based on ^13^C NMR chemical shifts. Table 1, shows that DU8ML-computed chemical shifts for the trans and cis isomers of **164­{10**-**rev}** match experimental data with high accuracy (rmsd from 0.82 to 1.09 ppm). While differentiation between the diastereomers is not possible, these low rmsd numbers impart confidence that, at least, the revision to the diastereomeric products of methanolysis is correct.

**Table 1:** RMSD (ppm) Values for DU8ML Computed *trans* and *cis* Diastereomers of **164­{10**-**rev}** Compared with the Experimental ^13^C NMR Chemical Shifts of the Major and Minor Isomers

	major (exp)	minor (exp)
***trans***	0.82	0.92
***cis***	1.09	1.09

In another study, TiCl_4_ catalyzes a formal [2 + 2] cycloaddition of an allenic moiety tethered to cyclohexadienone as shown in Figure 27.^49^ When the tether length is increased from dimethylene to trimethylene, the major product–according to the authors–is the meta-bridged **168­{12** ^′^ **}**. Structure **168­{12** ^′^ **}** conflicts with DU8ML calculations. After comparing several candidate structures, we revised **168­{12** ^′^ **}** to vinyl chloride **169­{12′**-**rev}**. According to the Sybyl force field, this open-chain vinyl chloride is about 32 kcal/mol lower in energy than the originally proposed 1,3-bridged benzene structure **168­{12** ^′^ **}**. Here, as in several other cases, discrepancies in the molecular formula raise concerns regarding the mass-spectrometric data. At present, we do not have a definitive explanation for these inconsistencies.

**Figure 27:**
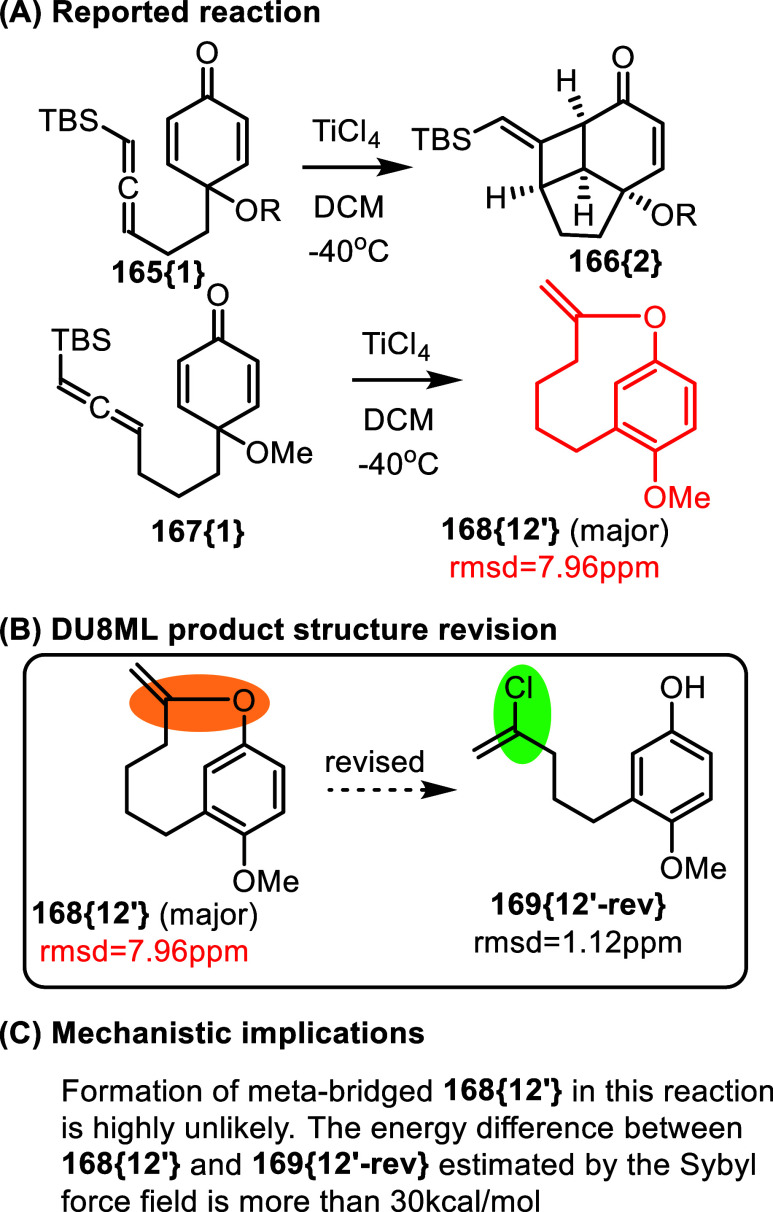
Alleged formation of 1,3-bridged benzene.

A three-component coupling of anilines, amines and difluorocarbene was developed for rapid access to formamidines, Figure 28.^50^ This reaction was also implemented in the intramolecular format for cyclization of 2-(*o*-aminophenyl)­benzoimidazole **170­{6a}** to benzo­[4,5]­imidazo­[1,2-*c*]­quinazoline **171­{7a}**. DU8ML calculations confirmed these structures; for example, **171­{7a}** give rmsd = 1.11 ppm, indicating correct assignment.

**Figure 28:**
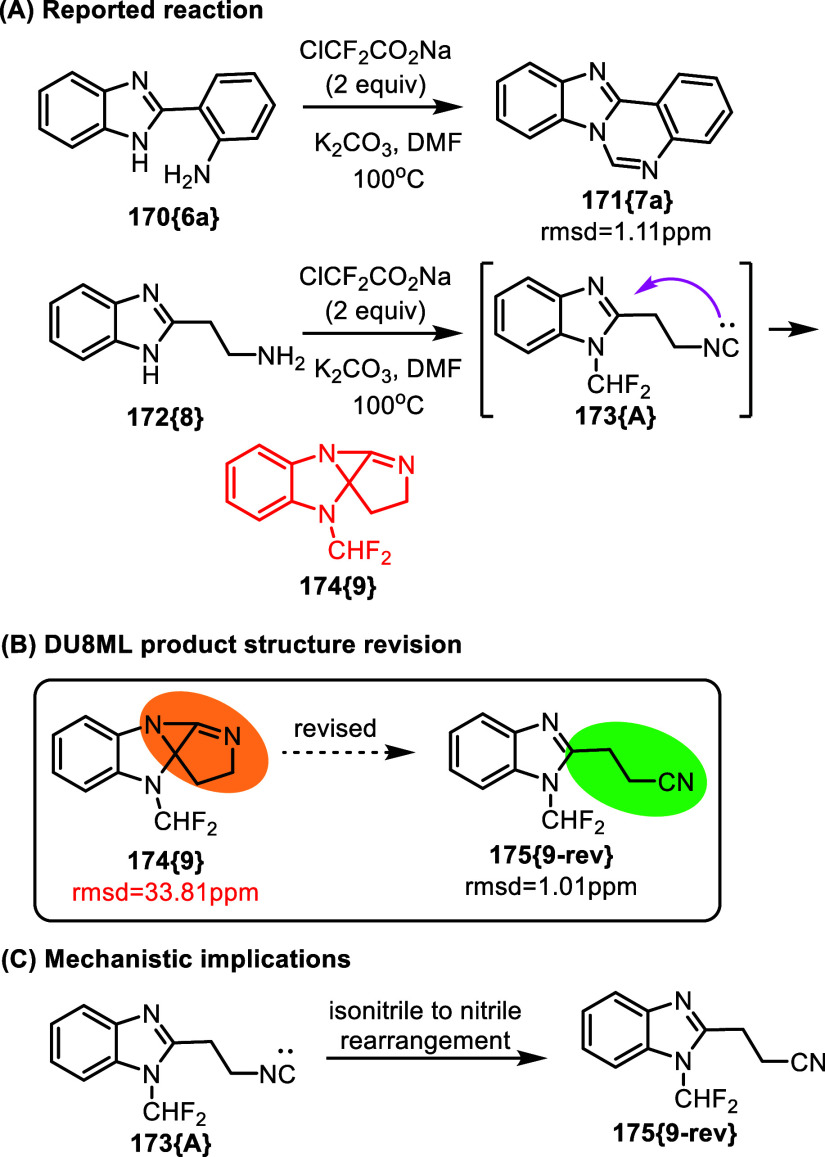
Three-component coupling of anilines, amines, and difluorocarbene to access formamidines.

However, for the product derived from aliphatic amine **172­{8}**, the authors suggested a rather unusual tetracyclic structure **174­{9}**, for which the computed and the experimental data were not reconcilable. We revised it to nitrile **175­{9**-**rev}** (rmsd = 1.01 ppm). Our mechanistic rationale involves isonitrile intermediate **173­{A}** (proposed by the authors) which rearranges into nitrile **175­{9**-**rev}**. Cyclization into **174­{9}** was unlikely to happen: according to DFT, structure **174­{9}** is 35 kcal/mol uphill of the revised cyanoethylbenzoimidazole **175­{9**-**rev}**.

### Heavy Atom Challenges

Figure 29 shows a study of Ru­(II)-catalyzed rearrangement of 2-diazo-1,3-diketones in the presence of halogenating reagents.^51^ The authors disclosed two substrate-dependent modes: (i) the Wolff rearrangement in the case of acyclic diazodiketones, yielding dihalomethylene products **180­{9a}**, **176­{9b}**, and **177­{10a}**, or (ii) bis-halogenation occurring without rearrangement of the molecular framework in cyclic diazodiketones, yielding products **179­{2b}**, **178­{3a}**, and other bis-halogenated cycloalkenones.

**Figure 29:**
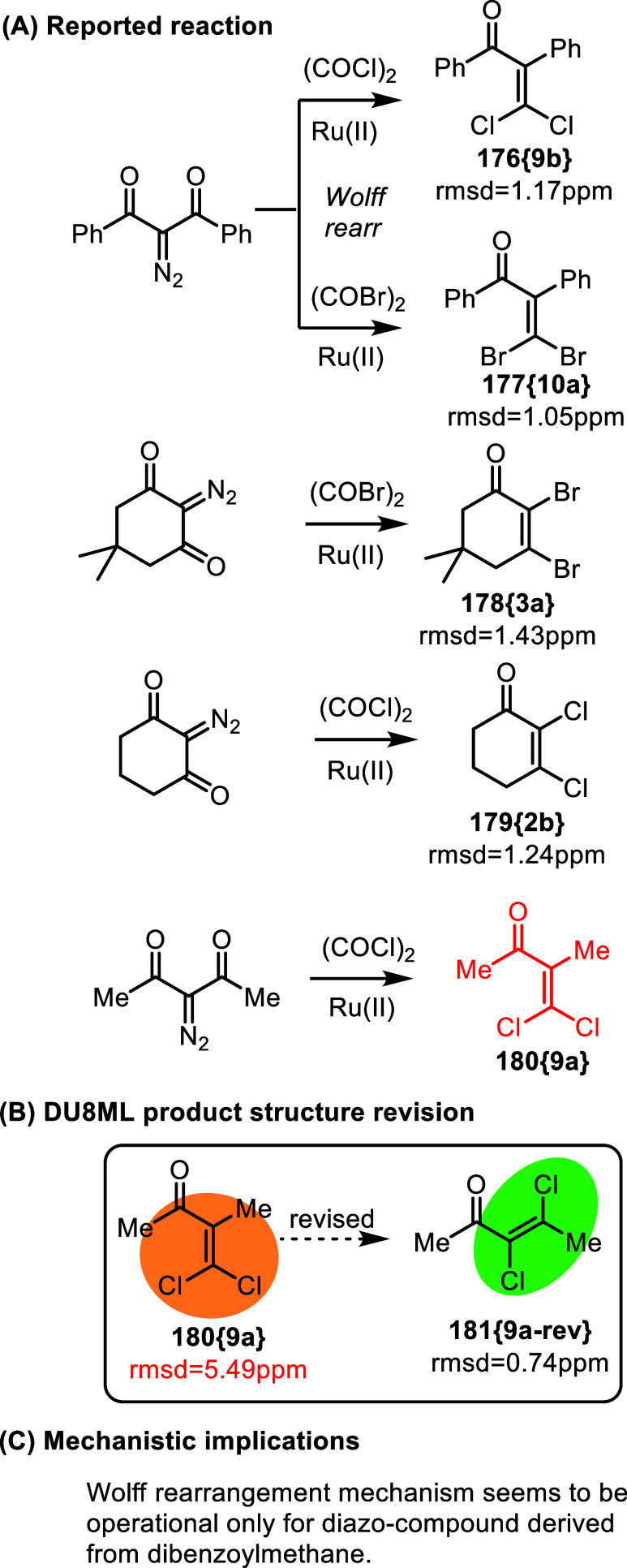
Dual mode Ru­(II)-catalyzed rearrangement of diazoketones in the presence of halogenating reagents.

4,4-Dichloro-3-methylbuten-2-one **180­{9a}**, as the rest of the products in this series, are typical examples of the challenges outlined above. ^1^H NMR is hardly useful, and ^13^C chemical shifts are difficult to predict, given the dihalomethylene moieties. Hence, somewhat expected errors in connectivity. DU8ML confirmed the correctness of most structures, but **180­{9a}** needed revision to **181­{9a**-**rev}** as shown. Mechanistic ramification: it appears that in this series the Wolff rearrangement only occurs in acyclic 2-diazo-1,3-diketones derived from aromatic ketones, such as dibenzoylmethane. Aliphatic precursors consistently produced 2,4-dihalogenated α,β-unsaturated ketones.

Another observation is that revised **181­{9a**-**rev}** has the *E*-stereoconfiguration of chlorine atoms. In cyclic systems such as **179­{2b}** and **178­{3a}** there is no alternative for the *cis* Hal-C=C-Hal geometry, but for acyclic **181­{9a**-**rev}** the *E*-isomer is preferred. Interestingly, DFT theory, B3LYP/6-31G­(d), predicts that *Z*-isomer is approximately 2.4 kcal/mol lower than the *E*-isomer, making it a product of *kinetic control*, which is another instructive feature of the mechanism.

Palladium-catalyzed cascade carboesterification of norbornene with alkynes, Figure 30,^52^ illustrates similar challenges of *E*/*Z* stereoconfiguration assignment in tetrasubstituted double bonds, especially with partial halogen substitution. The “carboesterification” products **182­{3}** seem to be assigned correctly. However, products **183­{5a}**/**184­{5b}** derived from substituted propargyl alcohols require revision to *Z* isomers. The case of **183­{5a}** and its revision to *Z* isomer **185­{5a**-**rev}** underscores the challenge of choosing the correct structure under the conditions where the differences between stereoisomers are extremely subtle. Additionally, the authors did not specify the configuration of the stereogenic center in the furan moiety (H–C–Me) of **183­{5a}**, which required calculations for both diastereomers of the original and revised structures (the clarified “Me-in” stereoconfiguration is accented by the gray circle in the revised product **185­{5a**-**rev}**).

**Figure 30:**
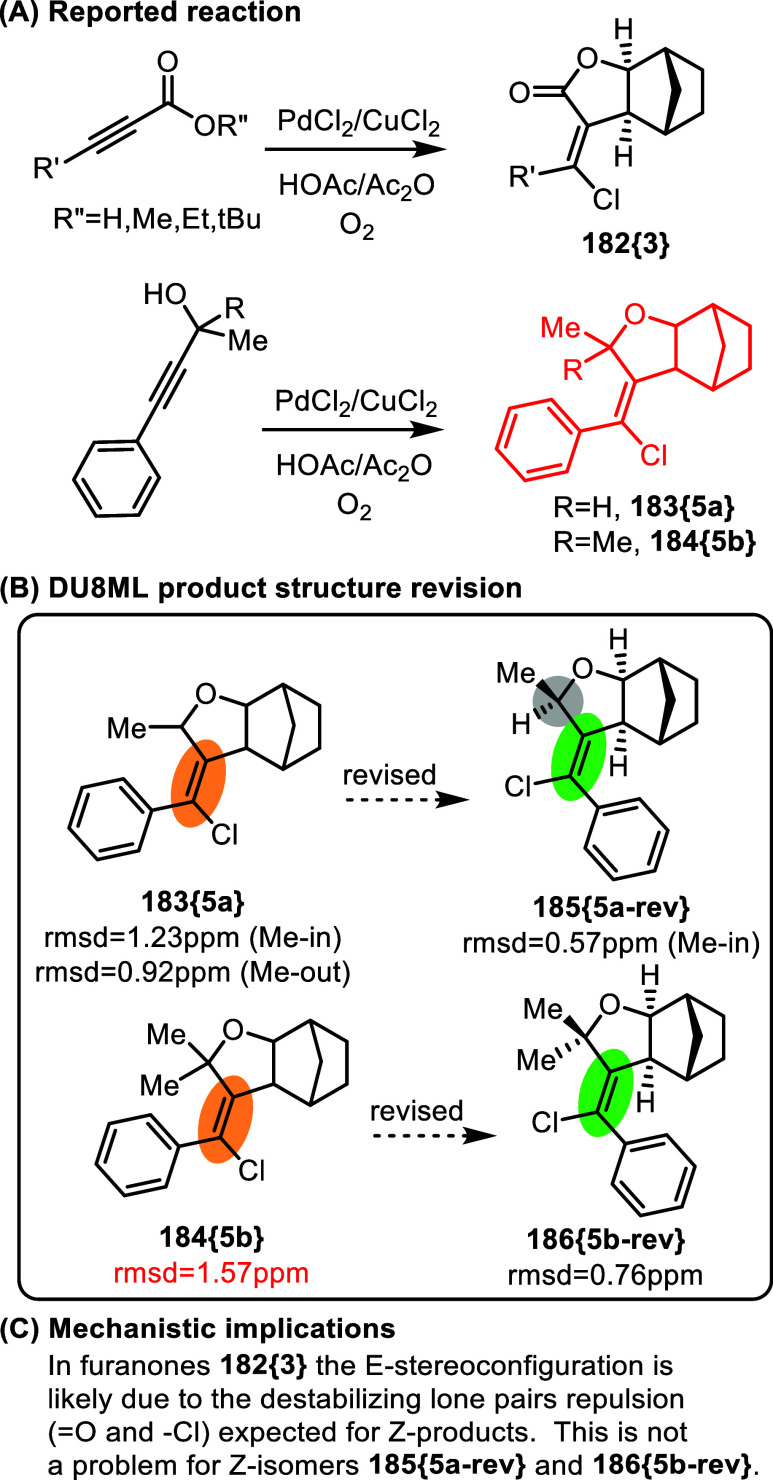
Palladium-catalyzed cascade carboesterification of norbornene with alkynes.

The differences between the original and the revised structures of **184­{5b}** are more pronounced and leave no doubt that the product has *Z*-configuration. Mechanistically, this may imply that the main driver for the *E*-configuration in the correctly assigned *furanone* products **182­{3}** is the lone pair repulsion between the = O and -Cl moieties (which is supported by small DFT energy bias of 1.7 kcal/mol in favor of the *E*-isomer). Such repulsion does not occur in furans **183­{5a}**/**184­{5b}**.

Iodine presents additional significant difficulties for NMR interpretation, because of its outsized spin–orbit contributions which make predicting C–I chemical shifts a guessing game. This could steer the structure elucidation effort down an unusual path, as shown in Figure 31. Transition metal-catalyzed decarboxylative halogenation of aryl carboxylic acids was developed for electron-deficient and electron-rich aromatics.^53^ The reaction seems to have a rather broad scope, with CuI used in the presence of Pd­(OAc)_2_ for electron-rich aromatic carboxylic acids, or CuI alone–for the electron-deficient aromatic acids. Reaction with *N*-methyl-3-indolecarboxylic acid produced the major 3-iodo product **187­{21a}** and the minor 2,3-diiodide **188­{21a** ^′^ **}**. While the major product was assigned correctly, ^13^C chemical shifts calculated for structure **188­{21a** ^′^ **}** using DU8ML did not agree with the experimental data. After a thorough analysis of the spectrum and a few candidate structures later, we arrived at *N*-methylisatin **189­{21a′**-**rev}** as the revision for diiodide **188­{21a** ^′^ **}** (rmsd = 1.45 ppm). This is a known compound.^54^ Its published experimental ^13^C NMR matches the ^13^C data for **188­{21a** ^′^ **-rev}** very well: rms*d*_exp‑exp_ = 0.47 ppm, leaving no doubt in this revision. The take home message for using a combination of CuI/Pd­(OAc)_2_ in the presence of oxygen is that for electron-rich aromatics overoxidation might be a problem.

**Figure 31:**
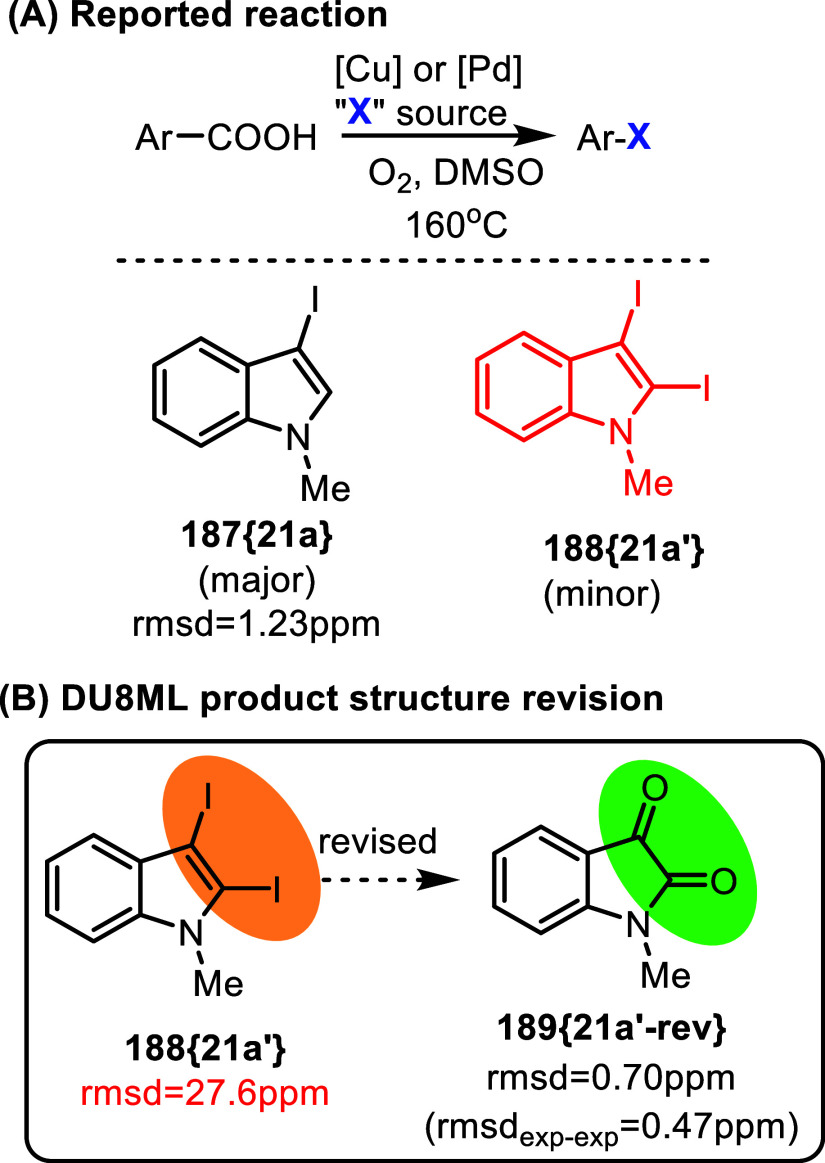
Decarboxylative halogenation.

A new method for direct C4 halogenations of isoquinolines via a Boc_2_O-mediated dearomatization strategy was reported recently.^55^ The majority of the products were assigned correctly. However, three products required revision as shown in Figure 32. It seems unlikely that this case carries any mechanistic implications. More likely scenario is that a wrong halogenating reagent was used to access **190­{5**–**4}** or **191­{5**–**5}** (TCCA instead of NBS?), whereas the case of **192­{5**–**24}** points to a wrong substrate precursor.

**Figure 32:**
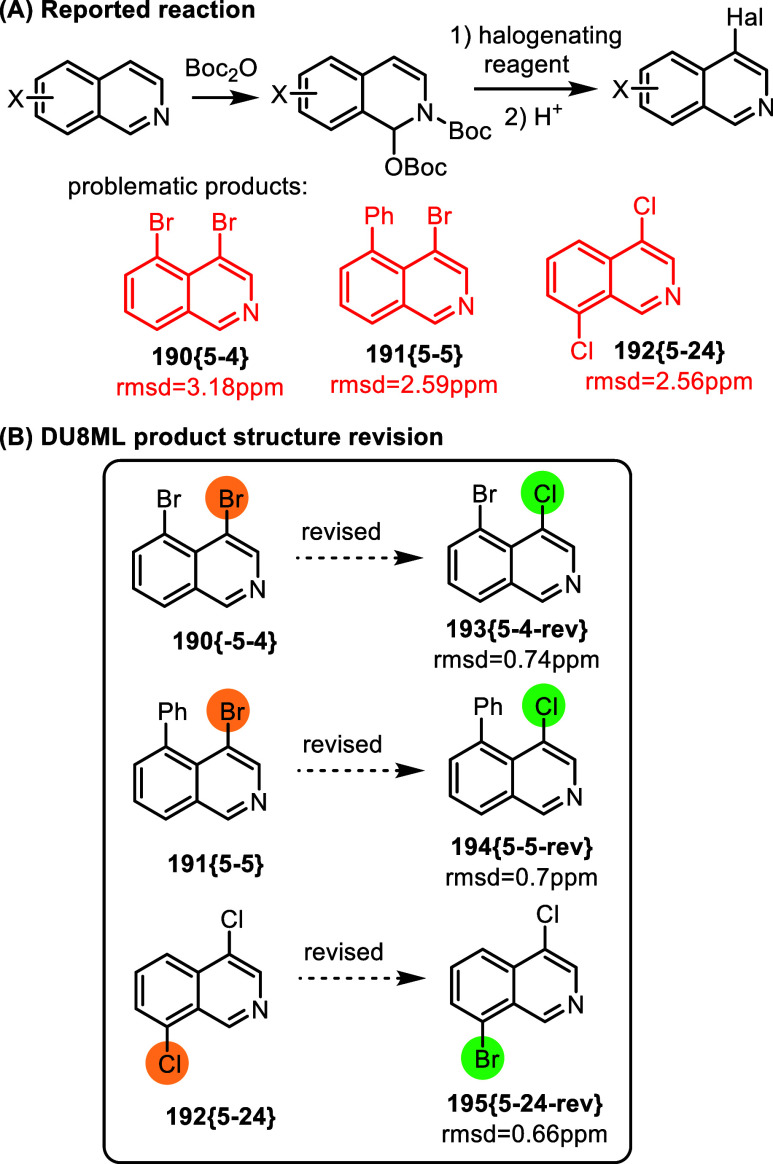
Boc_2_O-mediated 4-halogenation of isoquinolines.

Experimental errors are common for halogenation of aromatic compounds and, like in the case above, often do not have mechanistic implications. However, at times, a method yields both a subset of purported product which is a result of erroneous labeling of spectra, and another subset of misassigned compounds which clearly require mechanistic amendment. This seems to be the case of another halogenation study of indoles with NaX/PhI­(OAc)_2,_ where X = Cl, Br, I,^56^ Figure 33.

**Figure 33:**
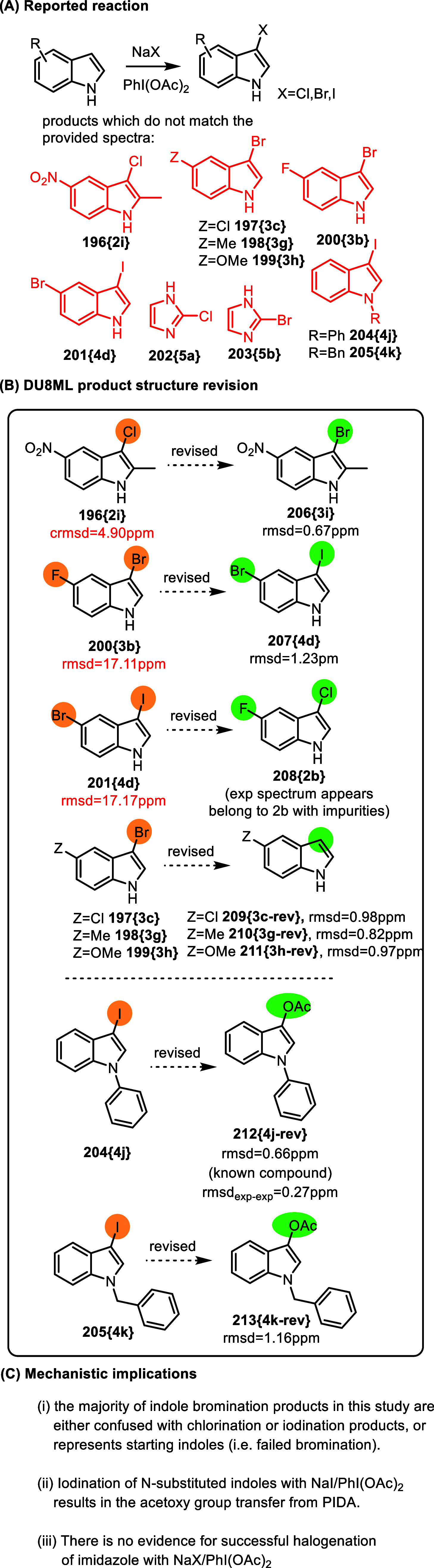
Halogenation of indoles with PIDA.

Most chlorinated and iodinated indole products were in agreement with their ^13^C spectra, as expected. However, a few spectra do not match. For example, ^13^C spectrum of chloride **196­{2i}** corresponds to the structure of bromide **206­{3i}**; the spectrum of 3-bromo-5-fluoroindole **200­{3b}** corresponds to the structure of **207­{4d}**, whereas ^13^C spectrum of **207­{4d}** itself is in keeping with the experimental ^13^C spectrum of **208­{2b}** (likely with impurities). More importantly from the reactivity standpoint, spectra for three bromides **197­{3c}**, **198­{3g}**, and **199­{3h}** indicate that bromination has failed and the isolated products are indistinguishable from the starting 3-unsubstituted indoles.

More important from the mechanistic standpoint is that, instead of 3-iodides **204­{4j}** and **205­{4k}**, iodination of *N*-phenyl or *N*-benzyl indole gave products of acetoxy-transfer from PIDA, **212­{4j**-**rev}** and **213­{4k**-**rev}**, which is a known reaction of *N*-unsubstituted phenols with PIDA and a base.^57^ Notice that **210­{3g**-**rev}**, **212­{4j**-**rev}** and **213­{4k**-**rev}** are known compounds.^58^

Finally, spectra of halogenation products of imidazole, **202­{5a}** and **203­{5b}**, appears to show not such halogenated products, but rather the imidazole precursor with varying quantities of acetic acid as an impurity affecting chemical shifts, which most likely indicates that halogenation of imidazole under these conditions was not successful.

The next two examples underscore challenges of structure elucidation of products possessing geminal dihalogen moieties. First example discloses an extensive mechanistic study–involving carbon isotope labeling–of transannular rearrangement of 5-cyclodecynone, Figure 34.^59^ Majority of the products were characterized correctly. However, unusual oxetane **216­{21}** caught our attention. The authors suggested that it was formed upon bromination of allylic alcohol **215­{20}** and had *trans*-dibromo stereoconfiguration. DU8ML agreed with neither *trans*- nor *cis*-dibromooxetane structures **216­{21}**. We therefore revised the product to decaline-based epoxide **217­{21**-**rev}** possessing a gem-dibromo moiety. Our minor revision of the mechanism is shown in Figure 34C, where the bromonium ion is opened to form epoxide **217­{21**-**rev}**. This revision also underscores rather common challenges with structure elucidation of both oxiranes^60^ and oxetanes.^61^

**Figure 34:**
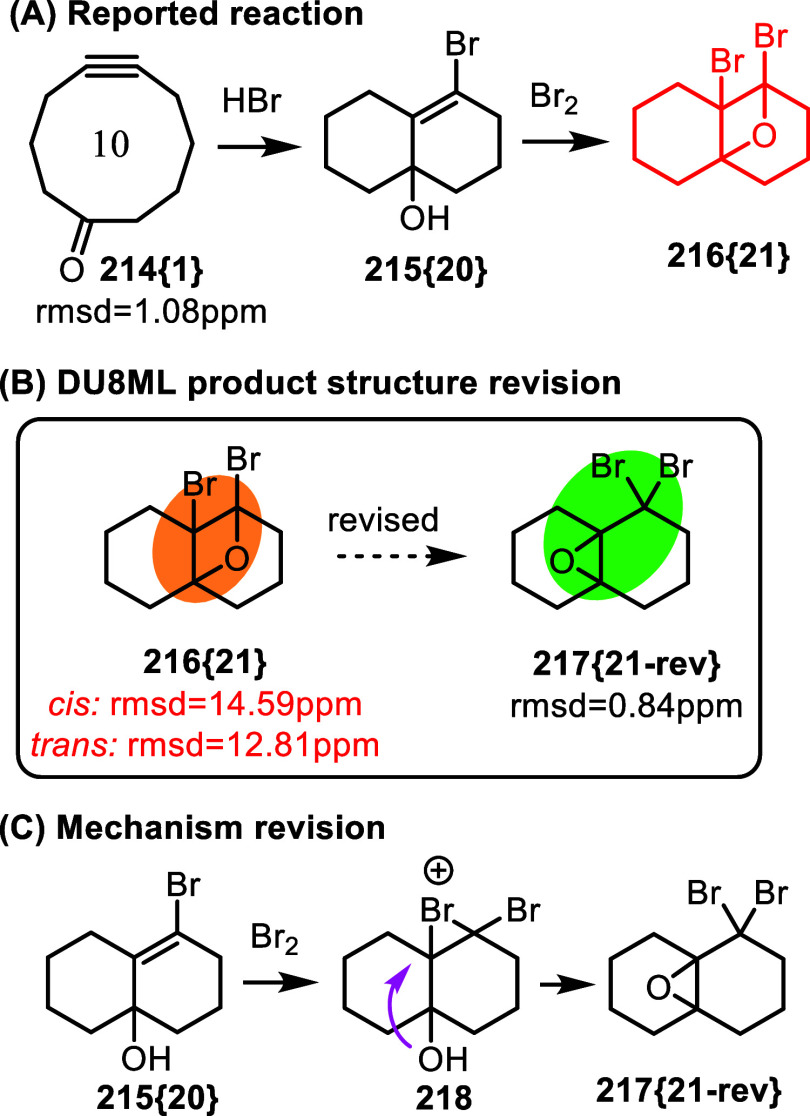
Transannular rearrangement of 5-cyclodecynone.

The second example has to do with the geminal mixed chloro-bromo moiety, Figure 35. Gem-dichlorocyclobutenones **219­{1a}** were used to gain access to Bpin-substituted cyclobutenones **220­{3a}**. These, in turn, were introduced into subsequent transformations, for example, bromination with NBS or oxidation with sodium perborate.^62^ Calculated ^13^C values for bromination product **221­{10}** disagree with experimental chemical shifts. Further analysis of the ^13^C NMR spectrum revealed that the aliphatic CH carbon has a chemical shift commensurate with that of an sp^2^ carbon, while the only aliphatic peak belongs to quaternary carbon. We therefore revised 2,4-dihalide **221­{10}** to geminal 4,4-chloro-bromo compound **223­{10**-**rev}**. The mechanism correction involved electrophilic allylic substitution of Bpin by [Br^+^], Figure 35C.

**Figure 35:**
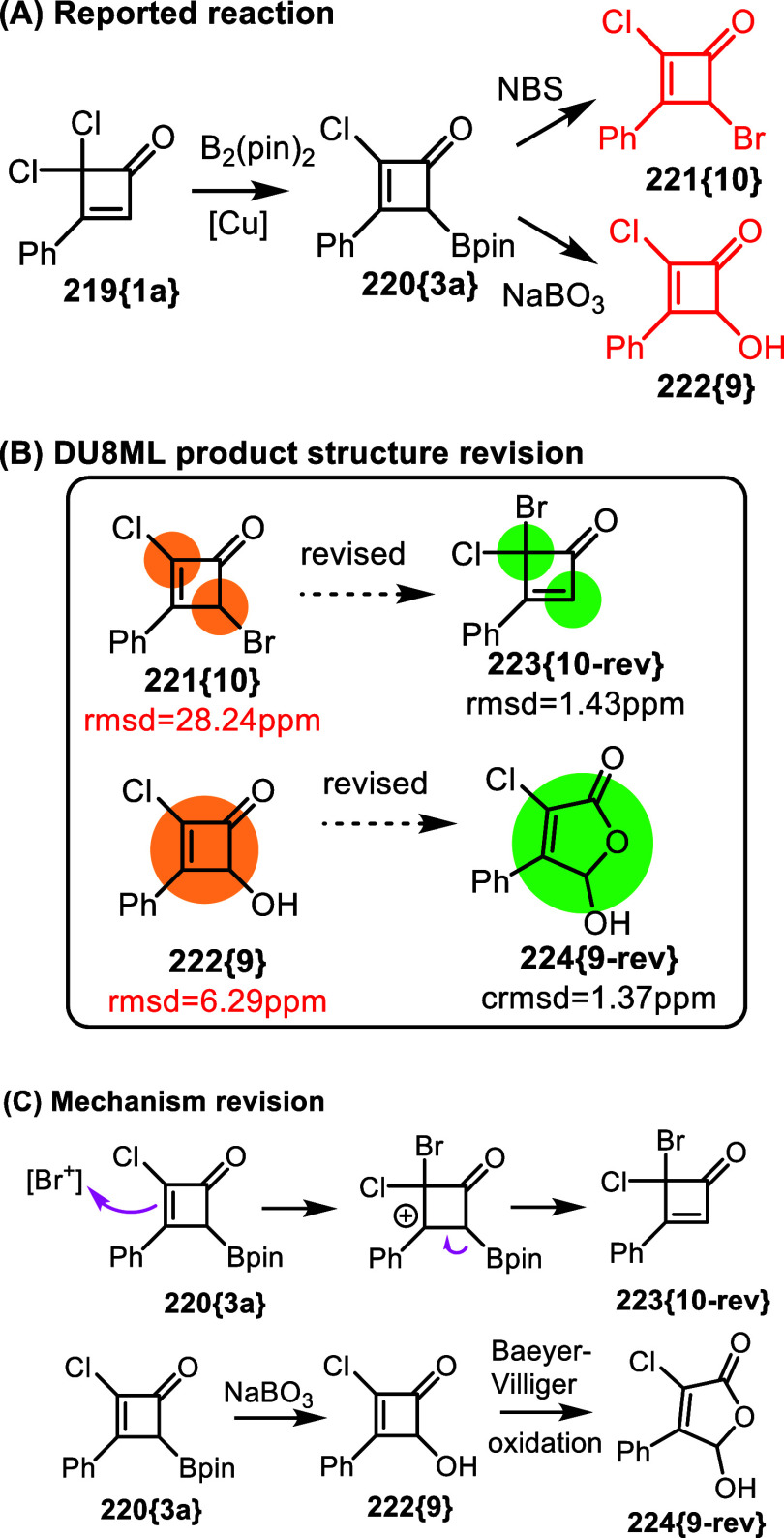
Bromination and oxidation of **220­{3a}**.

The oxidation product **222­{9}** also needed revision. The revised structure, i.e. lactol **224­{9**-**rev}**, indicated that oxidation of **220­{3a}** did not stop with the formation of alcohol **222­{9}**. It is likely, that overoxidation via the Baeyer–Villiger rearrangement produced the final product, lactol **224­{9**-**rev}**.

Another common “technical” problem: reported chemical shift values are offset by a random number due to imprecise referencing of ^13^C NMR spectra. Many initial red flags suggesting misassignment were, upon closer inspection of SI spectra, reclassified as issues stemming from careless chemical shift referencing rather than errors requiring structural revision. For example, two isomeric products of a Diels–Alder reaction of bicyclic strained allenes with furan, **225­{35b}** and **226­{36b}**, were reported in ref 63 Computed DU8ML values for compound **225­{35b}** showed excellent match with experimental data, rmsd­(δ_C_) = 1.23 ppm, whereas there was a mismatch for **226­{36b}**, rmsd­(δ_C_) = 2.11 ppm, plausibly indicating misassignment. As Figure 36 indicates, experimental ^13^C chemical shifts for isomer **226­{36b}** were referenced with an odd offset (TMS is referenced at −1.25 ppm and CDCl_3_ is referenced at 75.77 ppm). Correction of the experimental shifts for the universally agreed value of 77.16 ppm for chloroform-*d* gave a much better rmsd­(δ_C_) = 1.34 ppm, leaving no doubt that the structure **226­{36b}** is also correct and that it is the spectrum referencing that is sloppy. For isomer **225­{35b}**, CDCl_3_ peak was referenced more conventionally (i.e., sufficiently close to 77 ppm), which did not cause problems.

**Figure 36:**
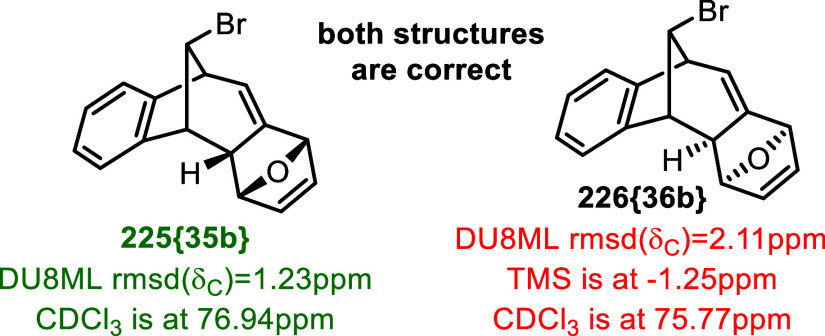
Careless referencing as a false-flag problem.

This erroneous referencing seems to be a rather common phenomenon, yet difficult to uncover unless spectra in a digital format or their high resolution images are published with Supporting Information. Several recent observations of this nature also gave us motivation to revisit a long lasting controversy with tribromide **227­{17}**, Figure 37, where DU8ML seemingly favored the wrong structure.^12^ With the method’s high accuracy confirmed for thousands of compounds (that is in addition to the current training set, which has more than 36,000 reliable experimental ^13^C chemical shifts, rmsd <1 ppm), we were curious about this failure of DU8ML to confirm the original (correct) structure of **227­{17}**.

**Figure 37:**
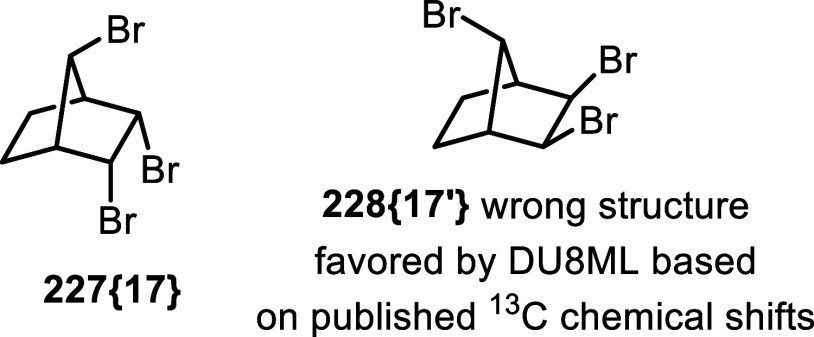
Tribromide **227­{17}** controversy.

In the original^64^ and more recent follow-up paper,^65^ Balci and co-workers did not provide ^13^C spectrum in the Supporting Information. Regrettably, our request for a copy was not acknowledged either. For that reason, we resynthesized tribromide **227­{17}**, for details see Supporting Information. Sure enough, experimental chemical shifts for the synthesized tribromide were different from the data set reported in the original publication (ref 64), i.e. all chemical shifts were offset by approximately 1.84 ppm, see Table 2. To be sure that these experimental shifts are not sensitive to concentration effects, we ran appropriate NMR experiments–no concentration dependence was observed.

**Table 2:**
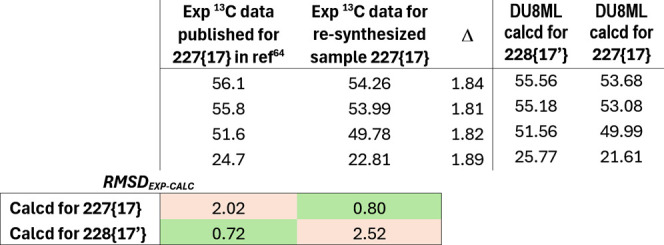
Experimental ^13^C Chemical Shifts from the Original Publication Compared with Re-synthesized Compound

Due to the *C*_S_ symmetry of the molecule, the tribromide has only four ^13^C peaks. Fortuitously, the set of ^13^C chemical shift values reported in the original publication favored structure **228­{17** ^′^ **}** (rmsd = 0.72 ppm), while giving a poor match with the original (correct) structure **227­{17}** (rmsd = 2.02 ppm).^66^

In contrast, experimental ^13^C data set for our *resynthesized sample* matched the DU8ML-computed values for structure **227­{17}** very well (rmsd = 0.8 ppm) while giving poor match with values calculated for incorrect structure **228­{17** ^′^ **}** (rmsd = 2.52 ppm). As the ^1^H NMR experimental spectra of the resynthesized sample match the originally published ^1^H NMR data, we have no reason to believe that these are two different compounds. The most plausible explanation is that ^13^C NMR spectrum in Balci’s original publication was incorrectly referenced to chloroform-*d*, which was mistakenly assigned the value of 79 ppm. Correcting this reference point to 77.16 ppm results in a near perfect match between the data from the original experiment, our synthetic sample, and DU8ML-computed values.

Considering the above, it might be helpful to respectfully bring up the issue with the conclusions by Sarotti in the most recent publication on the new HALO–DP4+ approach for DP4+ calculations in organohalogenated molecules.^67^ This new method from the DP4+ family is designed to better handle halogens. The HALO–DP4+ analysis was applied to this tribromide problem and strongly favored structure **227­{17}**, assigning it a probability greater than 99.9%, while the alternative structure **228­{17′}** was assigned the value less than 0.1%. This result would have been an impressive validation of this new method’s accuracy, except for one caveat: these probabilities were calculated using a poorly referenced set of original ^13^C chemical shifts from ref 64 Each of four values in that data set deviates by more than 1.8 ppm. This means that the HALO–DP4+ calculations assigned nearly 99.9% probability to a structure expected to exhibit at least a 1.8 ppm discrepancy between each computed and experimental ^13^C chemical shift (!). Such performance should give serious pause to anyone attempting to differentiate diastereomers using a method that evaluates chemical shifts with this level of inaccuracy.

A more general question: what should be done in the rare instance where two isomers happen to possess nearly linearly dependent sets of chemical shifts? In such cases, avoid methods based on additional linear scaling of calculated chemical shifts (intended to achieve a better match with the given set of experimental values). As mentioned above, we use such additional linear scaling (and denote the result as “corrected rmsd” or crmsd^23^) only in rare cases involving exotic structural elements and polar/protic NMR solvents, and only after ensuring that the experimental chemical shift sets for candidate isomers are not linearly dependent. In vast majority of cases, DU8ML chemical shifts are calculated using generalized scaling terms developed for a particular solvent and stored in the parameter tables (i.e., no additional linear scaling is involved). The importance of these generalized scaling terms for individual solvents was articulated before; for example, see Gonnella’s paper on DiCE.^68^

Finally, one notes that the whole saga with **227­{17}** could have been avoided if Balci and coauthors were to share the actual ^13^C spectrum in any of their publications. Another, perhaps more important, takeaway is that the excellent agreement between the experimental NMR data obtained from our synthetic sample and the DU8ML-predicted chemical shifts for **227­{17}** underscores the outstanding accuracy and reliability of our machine learning–augmented DFT approach for computational NMR.

The second set of ^13^C chemical shift data from the Balci group^69^ for the dibromide that we corrected in our previous publication,^12^ was referenced by the authors correctly, with sufficient precision. That is to say, our revision of **229­{17}** to its epimer **230­{17**-**rev}** stands, Figure 38.

**Figure 38:**
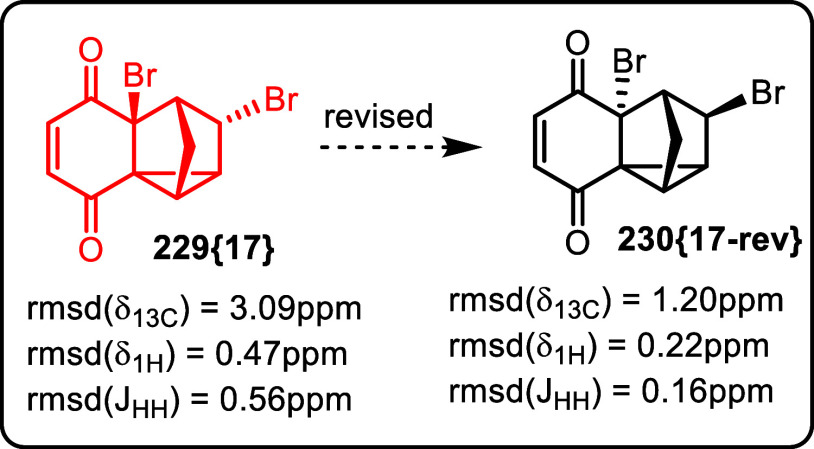
Earlier revision of dibromide **229­{17}** to its epimer **230­{17**-**rev}** (Adapted from ref 12 Copyright 2022 American Chemical Society).

Stereoconfiguration often remains undefined, particularly when there is little incentive for detailed structure elucidation, such as cases where subsequent transformations yield achiral products. For instance, bromoladderane **231­{S47}** (with C–Br stereoconfiguration unspecified) is commonly dehydrobrominated in various studies, making its configuration largely irrelevant.^70^ However, using DU8ML, we determined its stereoconfiguration to be exo (Figure 39A). Given the potential preference of bulky bases for an *exo* approach, this suggests that such bromoladderanes may preferentially undergo *syn*-elimination of HBr, yielding ladderene products.

**Figure 39:**
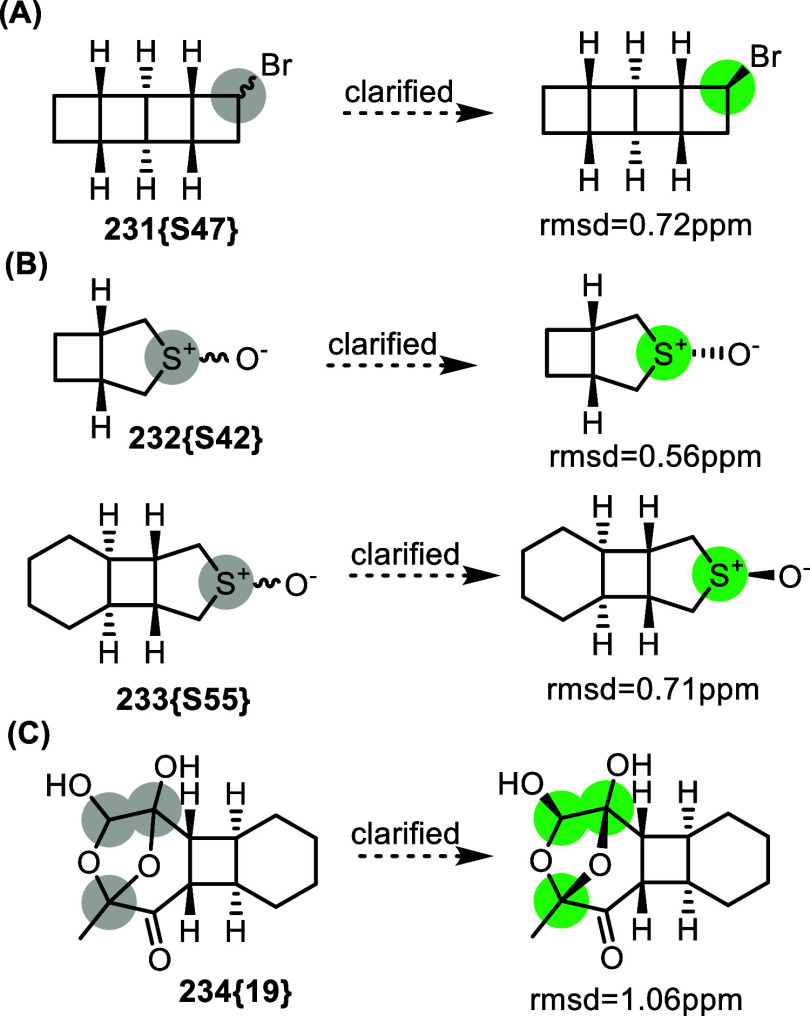
Undefined stereoconfiguration clarified with the help of DU8ML.

Another noteworthy finding enabled by DU8ML is that sulfoxides **232­{S42}** and **233­{S55}**, reported in the same paper, possess opposite configurations at the sulfoxide center for some reason. Stereoconfiguration of three undefined stereogenic centers in polyacetal **234­{19}** was also clarified.^71^ This is not inherently problematic; however, fully elucidating stereochemical outcomes, even for intermediates, can provide valuable support for mechanistic hypotheses proposed in related studies.

### Miscellaneous Cases

Frustrated Lewis pair catalyzed dehydrogenative oxidation of indolines offers high yielding access to indoles (and other heterocycles), Figure 40.^72^ One revision that was necessary according to DU8ML is shown in Figure 40B. The computed NMR data for the putative *N*-*t*-butyl isoindole structure **236­{6}** did not match the experimental values. Additionally, the number of peaks was not in keeping with this symmetric structure. We revised isoindole **236­{6}** to isoindolinone **237­{6**-**rev}** as shown. Literature search revealed that this is a known compound with matching NMR spectra.^73^

**Figure 40:**
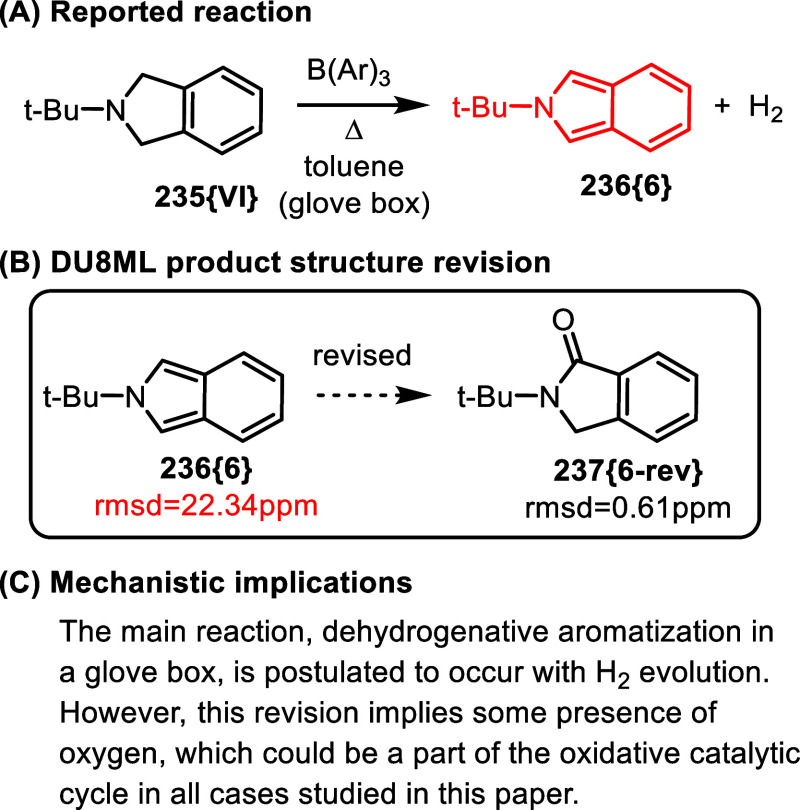
B­(Ar)_3_ catalyzed dehydrogenative aromatization.

This is a minor product revision in a paper with dozens of examples attesting to the broad scope of this useful reaction. However, this revision points to a scenario where nontrivial amount of molecular oxygen could be present during the reaction and could be a part of the proposed catalytic cycle, necessitating a slight tweaking of the mechanism.

Finally, a just-published study disclosed a base-induced cyclization of substituted 1,2,4-triazolidine-3,5-diones **238­{17}** yielding fused 1,2-diazetidin-3-ones **239**–**242**, Figure 41.^74^

**Figure 41:**
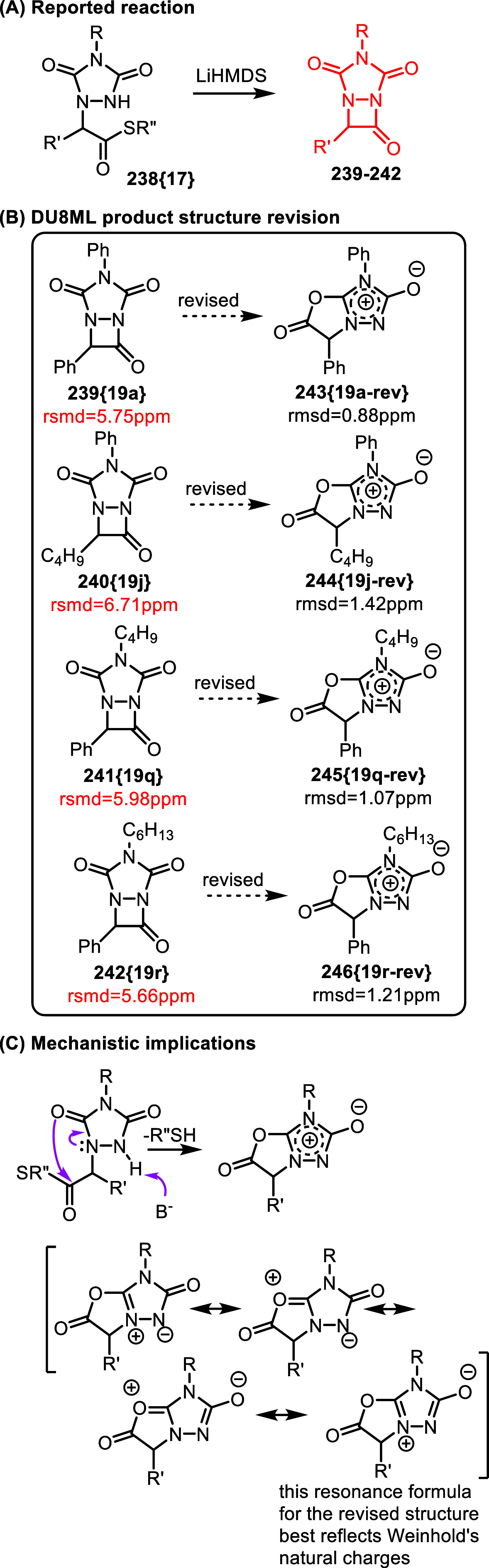
Base-induced cyclization in substituted 1,2,4-triazolidine-3,5-diones.

DU8ML computations did not match the experimental NMR data for products **239**–**242**, as illustrated by poor rmsd’s > 5 ppm. We hypothesized that after deprotonation of the NH, the delocalized anion could act as an ambident nucleophile and induce the formation of a five-membered ring, rather than the four-membered diazetidinone. For structural consideration, the carbonyl proximal to the thiol ester electrophile could act as the nucleophile. This hypothesis yielded zwitterionic candidate structures **243**–**246** for which DU8ML computations matched the experimental chemical shifts very well, confirming the validity of this hypothesis.

Similar products have been reported in the context of [3 + 2] cycloaddition of triazolinedione and tetracyclopropylethylene, Figure 42A.^75^ These meso-ionic species were kinetic products, obtained at 20 °C. The authors converted them to diazetidines **248­{4}** upon heating at 55 °C for 63 h. This gave us an opportunity to compare the accuracy of DU8ML when applied to meso-ionic structures. As shown in Figure 42A, for both [3 + 2] and formal [2 + 2] products DU8ML gave a very good match between the computed and experimental ^13^C data.

**Figure 42:**
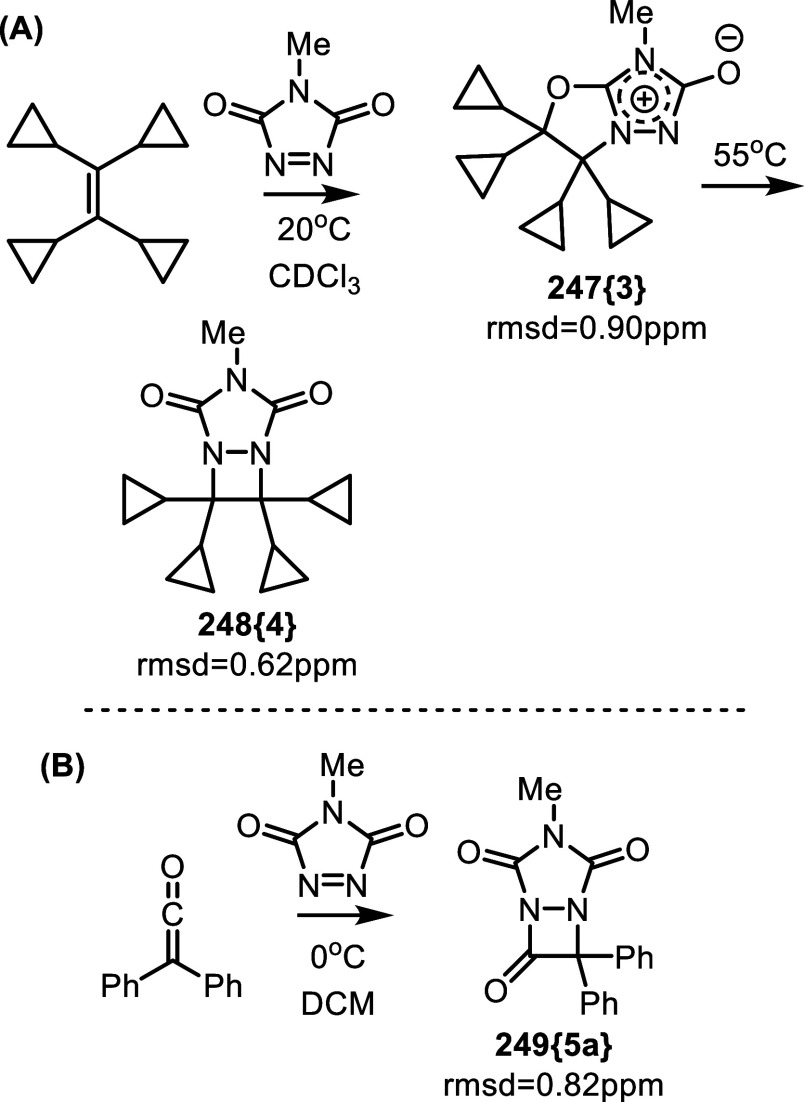
Products of [3 + 2] and [2 + 2] cycloadditions of triazolinedione with correctly assigned structures.

A separate study on ketene cycloadditions with triazolinedione reported the formation of a [2 + 2] product, **249­{5a}**, as the sole outcome of a low-temperature reaction (0 °C).^76^ Notably, the high accuracy of DU8ML computations for **249­{5a}** reinforces confidence in our ability to detect misassignments in diazetidinones **239**–**242**.

## Conclusions and Future Outlook

Incorrect structural assignments pose significant challenges. As demonstrated here, such errors often lead to flawed mechanistic hypotheses, amplifying their negative impact on the field of organic chemistry. Fortunately, solution-phase structure elucidation by NMR now benefits from major advances in computational methods, including our machine-learning–augmented DFT approach, DU8ML. Computational NMR is becoming faster, more accurate, and increasingly user-friendly, and should be embraced as a routine tool in the organic chemist’s toolbox.

A key consideration moving forward is the cost–benefit balance in computational strategies. Ideally, rapid ML-assisted workflows will employ inexpensive zeroth-order methods for structure optimization and NMR parameter calculations, followed by machine-learning refinement. However, our experience indicates that distinguishing closely related isomers, particularly diastereomers, remains challenging for simple neural-network models that forego higher-level computations as a starting point. In such cases, accurate NMR predictions from quantum methods are essential. Our approach offers a practical compromise: using relatively low-cost DFT methods for geometry optimization and NMR calculations, achieving an effective balance between accuracy and computational cost. As we have demonstrated before, the wall-clock-time for such calculations of an organic structure of strychnine size could be under 30–40 min on a compute node of a typical garden variety midtier Linux cluster.

Future method development in computational NMR will likely adopt hybrid strategies that integrate machine learning with quantum chemical data. Massive data sets that we have generated so far with DU8ML can serve as training material for faster, lower-cost predictive models, especially, given the high accuracy of DU8ML-computed data (rmsd ≈1 ppm). A major advantage of this paradigm is the ability to use computed data for individual conformers directly, eliminating the need for conformer averaging and enabling greater automation.^77^ This approach leverages the abundance of computed structures, including hypothetical compounds, while using sparse experimental data as quality-control checkpoints. Such workflows promise to accelerate and streamline ML training, making computational NMR more efficient and broadly applicable.

## Supplementary Material



## Data Availability

The data underlying this study are available in the published article and its Supporting Information

## Notes

Computational details, DU8ML method description, calculated NMR chemical shifts and DFT-optimized Cartesian coordinates for putative and revised molecular structures. Experimental procedures and experimental NMR data for resynthesized compound **227­{17}** (PDF)
