# Supplementary material for: Reaction Products Revised and Mechanisms Revisited with Machine Learning-Augmented Computational NMR
Source: J Org Chem. 2026 Mar 16;91(12):4130–53. doi: 10.1021/acs.joc.5c02863 (PMC13036792; doi:10.1021/acs.joc.5c02863)
Supplement: Supplementary file 1 [file jo5c02863_si_001.pdf]

Reaction Products Revised and Mechanisms Revisited with Machine Learning-Augmented Computational NMR.

Srinivas Beduru, Enoch Asimbisa, Ivan M. Novitskiy, and Andrei G. Kutateladze*

Department of Chemistry and Biochemistry, University of Denver, Denver, CO 80208

SUPPORTING INFORMATION

Table of contents

NMR computations (DU8ML)....8

COMPUTATIONAL DETAILS....8

DU8ML and machine learning protocols....8

How to read the NMR tables in the SI:....20

REVISED AND VALIDATED STRUCTURES....21

Originally assigned (correct) structure of 6{14} ($CDCl_3$)....21

Originally assigned (correct) structure of 7{15} ($CDCl_3$)....26

Originally assigned (incorrect) structure of 4{13} ($CDCl_3$)....30

Revised structure of 4{13}, i.e 8{13-rev} ($CDCl_3$)....34

Originally assigned (incorrect) structure of 2{7a} ($CDCl_3$)....36

Revised structure of 2{7a}, i.e *Z*-isomer 9{7a-rev} ($CDCl_3$)....43

Originally assigned (incorrect) structure of 13{4a'} ($CDCl_3$)....50

Revised structure of 13{4a'}, i.e epimer 14{4a'-rev} ($CDCl_3$)....53

Originally assigned correct structure of 17{3e} ($CDCl_3$)....56

Originally assigned correct structure of 18{3f} ($CDCl_3$)....58

Original correct structure of 19{3l} (xray) to test DU8ML performance ($CDCl_3$)....60

Testing the (incorrect) *cis*-structure for 19{3l} ($CDCl_3$)....62

Originally assigned (incorrect) structure 20{3a} ($CDCl_3$)....64

Revised structure of 20{3a}, i.e. 25{3a-rev} ($CDCl_3$)....67

Original (incorrect) structure of 21{3b} ($CDCl_3$)....69

Revised structure of 21{3b}, i.e. *cis* 26{3b-rev} ($CDCl_3$)....72

Original (incorrect) structure of 22{3g} ($CDCl_3$) 74
Revised structure of 22{3g}, i.e. *cis* 27{3g-rev} ($CDCl_3$) 77
Original (incorrect) structure of 23{3h} ($CDCl_3$) 79
Revised structure of 23{3h}, i.e. *cis* 28{3h-rev} ($CDCl_3$) 82
Original (incorrect) structure of 24{3m} ($CDCl_3$) 85
Revised structure of 24{3m}, i.e. *cis* 29{3m-rev} ($CDCl_3$) 88
Original (incorrect - *cis*) structure of 31{27} ($CDCl_3$) 90
Revised structure of 31{27}, i.e. *trans* 32{27-rev} ($CDCl_3$) 92
Originally assigned (incorrect) structure of 35{2n'} (minor product) ($CDCl_3$) 94
Revised structure of 35{2n'}, i.e. 36{2n'-rev} ($CDCl_3$) 97
Originally assigned correct structure of 40{12j} ($CDCl_3$) 99
Originally assigned (incorrect) structure of 41{14j} ($CDCl_3$) 102
Revised structure of 41{14j}, i.e. 42{14j-rev} ($CDCl_3$) 104
Originally assigned (incorrect) structure of 43{12m} ($CDCl_3$) 106
Revised structure of 43{12m}, i.e. 44{12m-rev} ($CDCl_3$) 108
Originally assigned (incorrect) structure of 46{4m} ($CDCl_3$) 110
Revised structure of 46{4m}, i.e 47{4m-rev} ($CDCl_3$) 112
Originally assigned (incorrect) structure of 48{4} (Acetone-d_6) 114
Revised structure of 48{4}, i.e 50{4-rev} (Acetone-d_6) 115
Originally assigned (incorrect) structure of 49{5} ($CDCl_3$) 116
Revised structure of 49{5}, i.e 51{5-rev} ($CDCl_3$) 117
Originally assigned (incorrect - benzotriazepinone) structure of 53{3ae} ($CDCl_3$) 118
Revised structure of 53{3ae}, i.e. quinazolin 54{3ae-rev} ($CDCl_3$) 120
Originally assigned (incorrect) structure of 58{4ap} as exo-isomer ($CDCl_3$) 122
Originally assigned (incorrect) structure of 58{4ap} as endo-isomer ($CDCl_3$) 124
Revised structure of 58{4ap}, i.e iso-thiophenyl/exo 59{4ap-rev} ($CDCl_3$) 126
Originally assigned (incorrect) structure of 62{3ra} ($CDCl_3$) 128
Revised structure of 62{3ra}, i.e. *cis* 63{3ra-rev} ($CDCl_3$) 129
Originally assigned correct structure of cinnoline 65{11g} ($CDCl_3$) 130
Originally assigned correct structure of 66{11u} (CD_3OD) 131
Originally assigned (incorrect) structure of 67{11t} (CD_3OD) 132
Revised structure of 67{11t}, i.e. 68{11t-rev} (CD_3OD) 133
Originally assigned (incorrect) structure of 64{6t} ($CDCl_3$) 134

Revised structure of precursor 64{6t}, i.e. 69{6t-rev} ($CDCl_3$) 135
Originally assigned (incorrect) structure of 71{2a} (DMSO-d_6) 136
Revised structure of 71{2a}, i.e. 75{2a-rev} (DMSO-d_6) 138
Originally assigned (incorrect) structure of 72{2d} (DMSO-d_6) 140
Revised structure of 72{2d}, i.e. 76{2d-rev} (DMSO-d_6) 142
Originally assigned (incorrect) structure of 73{2e} (DMSO-d_6) 144
Revised structure of 73{2e}, i.e. 77{2e-rev} (DMSO-d_6) 146
Originally assigned (incorrect) structure of 74{2u} (DMSO-d_6) 148
Revised structure of 74{2u}, i.e. 78{2u-rev} (DMSO-d_6) 150
Originally assigned (incorrect) structure of 79{2ab} (DMSO-d_6) 152
Revised structure of 79{2ab}, i.e. 80{2ab-rev} (DMSO-d_6) 153
Originally assigned (incorrect) structure of 84{8} ($CDCl_3$) 155
Revised structure of 84{8}, i.e 85{8-rev} ($CDCl_3$) 157
Originally assigned correct structure of 86{3k} (Acetone-d_6) additional linear scaling for acetone - crmsd .160
Originally assigned (incorrect) structure of 87{3a} ($CDCl_3$) 165
Revised structure of 87{3a}, i.e. 90{3a-rev} ($CDCl_3$) 170
Originally assigned (incorrect) structure of 88{3l} ($CDCl_3$) 173
Revised structure of 88{3l}, i.e. 91{3l-rev} ($CDCl_3$) 176
Originally assigned correct structure 92{1} ($CDCl_3$) 179
Originally assigned correct structure 95{2} ($CDCl_3$) 180
Originally assigned correct structure 93{4} ($CDCl_3$) 181
Originally assigned correct structure 94{5} ($CDCl_3$) 182
Originally assigned (incorrect) structure of 96{13} ($CDCl_3$) 183
Revised structure of 96{13}, i.e. 97{13-rev} ($CDCl_3$) 184
Originally assigned (incorrect) structure of 99{5a} ($CDCl_3$) 185
Revised structure of 99{5a}, i.e. 101{5a-rev} ($CDCl_3$) 189
Originally assigned (incorrect) structure of 100{5g} ($CDCl_3$) 192
Revised structure of 100{5g}, i.e. 102{5g-rev} ($CDCl_3$) 195
Originally assigned correct structure of 104{7b} ($CDCl_3$) 198
Originally assigned correct structure of 105{7d} ($CDCl_3$) 202
Originally assigned (incorrect) structure of 106{7a} ($CDCl_3$) 206
Revised structure of 106{7a}, (xray is obtained for a different regioisomer) i.e regioisomer 110{7a-rev} ($CDCl_3$) 210

Originally assigned (incorrect) structure of 107{7c} ($CDCl_3$) 214
Revised structure of 107{7c}, i.e regioisomer 111{7c-rev} ($CDCl_3$) 217
Originally assigned (incorrect) structure of 108{7e} ($CDCl_3$) 221
Revised structure of 108{7e}, i.e regioisomer 112{7e-rev} ($CDCl_3$) 223
Originally assigned (incorrect) structure of 109{7j} ($CDCl_3$) 227
Revised structure of 109{7j}, i.e regioisomer 113{7j-rev} ($CDCl_3$) 229
Originally assigned (incorrect) structure of 115{3a} ($CDCl_3$) 233
Revised structure of 115{3a}, i.e 117{3a-rev} ($CDCl_3$) 234
Table S1: Comparison between actual (115{3a}) compound spectral data with known compound references 236
Originally assigned (incorrect) structure of 116{3i} ($CDCl_3$) 237
Revised structure of 116{3i}, i.e 118{3i-rev} ($CDCl_3$) 239
Originally determined (correct) structure of 121{7a} ($CDCl_3$) 242
Originally assigned (incorrect) structure of 123{6b} ($CDCl_3$) 246
Revised structure of 123{6b}, i.e 126{6b-rev} ($CDCl_3$) 250
Originally assigned (incorrect) structure of 124{7b} ($CDCl_3$) 253
Revised structure of 124{7b}, i.e 127{7b-rev} ($CDCl_3$) 256
Originally assigned (incorrect) structure of 125{7c} ($CDCl_3$) 260
Revised structure of 125{7c}, i.e 128{7c-rev} ($CDCl_3$) 263
Originally assigned (incorrect) structure of 122{7a-1} ($CDCl_3$) 266
Revised structure of 122{7a-1}, i.e 129{7a-1-rev} ($CDCl_3$) 271
Originally assigned (incorrect) structure of 131{26} (DMSO-d_6) 274
Revised structure of 131{26}, i.e enamide 132{26-rev} (DMSO-d_6) 276
Originally assigned (incorrect) structure 133{3a} ($CDCl_3$) 278
Revised structure of 133{3a}, i.e. 140{3a-rev} ($CDCl_3$) 280
Originally assigned (incorrect) structure 134{3b} ($CDCl_3$) 282
Revised structure of 134{3b}, i.e. 141{3b-rev} ($CDCl_3$) 284
Originally assigned (incorrect) structure 135{3c} ($CDCl_3$) 286
Revised structure of 135{3c}, i.e. 142{3c-rev} ($CDCl_3$) 288
Originally assigned (incorrect) structure 136{3h} ($CDCl_3$) 290
Revised structure of 136{3h}, i.e. 143{3h-rev} ($CDCl_3$) 292
Originally assigned (incorrect) structure 137{3p} ($CDCl_3$) 294
Revised structure of 137{3p}, i.e. 144{3p-rev} ($CDCl_3$) 297

Originally assigned (incorrect) structure 138{3q} ($CDCl_3$)....300
Revised structure of 138{3q}, i.e. 145{3q-rev} ($CDCl_3$)....303
Table S2: Comparison ^{13}C spectral data between actual compounds (133-139) and revised compounds (140-146) with known compound references....306
Originally assigned (incorrect) structure 139{3r} ($CDCl_3$)....309
Revised structure of 139{3r}, i.e. 146{3r-rev} ($CDCl_3$)....312
Originally assigned correct structure 148{1b} ($CDCl_3$)....315
Originally assigned correct structure of 149{1f} ($CDCl_3$)....317
Originally assigned correct structure of 152{3b} ($CDCl_3$)....319
Originally assigned (incorrect) structure of 150{1g} ($CDCl_3$)....321
Revised structure of 150{1g}, i.e imidazothiazinone 154{1g-rev} ($CDCl_3$)....322
Originally assigned (incorrect) structure of 151{1h} ($CDCl_3$)....323
Revised structure of 151{1h}, i.e enamide 155{1h-rev} ($CDCl_3$)....325
Originally assigned (incorrect) structure of 153{1i} ($CDCl_3$)....327
Revised structure of 153{1i}, i.e enamide 156{1i-rev} ($CDCl_3$)....329
Originally assigned (incorrect) structure of 158{10c} (Benzene-d_6)....331
Revised structure of 158{10c}, i.e. 160{10c-rev} (Benzene-d_6)....333
Originally assigned (incorrect) structure of 159{10h} (Benzene-d_6)....335
Revised structure of 159{10h}, i.e. 161{10h-rev} (Benzene-d_6)....337
Structure (correct) of starting 162{9} ($CDCl_3$)....339
164{10-rev}(*trans*) matched with exp ^{13}C shifts of the major product ($CDCl_3$)....342
164{10-rev}(*trans*) matched with exp ^{13}C shifts of the minor product ($CDCl_3$)....348
164{10-rev}(*cis*) matched with exp ^{13}C shifts of the major product ($CDCl_3$)....353
164{10-rev}(*cis*) matched with exp ^{13}C shifts of the minor product ($CDCl_3$)....358
Originally assigned (incorrect) structure of 168{12’} ($CDCl_3$)....362
Revised structure of 168{12’}, i.e 169{12’-rev} ($CDCl_3$)....364
Originally assigned (correct) structure of 171{7a} (DMSO-d_6)....392
Originally assigned (incorrect) structure 174{9} ($CDCl_3$) Notice that aziridine's C-N bond in this highly strained and unusual structure needs to be fixed in order to prevent rearrangement into quinoxalinium..393
Revised structure of 174{9}, i.e cyanoethylbenzoimidazole 175{9-rev} ($CDCl_3$)....394
Originally assigned (incorrect) structure of 180{9a} ($CDCl_3$)....397
Revised structure of 180{9a}, i.e. 181{9a-rev} ($CDCl_3$)....398
Originally assigned (incorrect) structure of 183{5a} with in config of Me ($CDCl_3$)....399
Originally assigned (incorrect) structure of 183{5a} with out config of Me ($CDCl_3$)....401

Revised structure of 183{5a}, i.e 185{5a-rev} ($CDCl_3$)....403
Originally assigned (incorrect) structure of 184{5b} ($CDCl_3$)....405
Revised structure of 184{5b}, i.e 186{5b-rev} ($CDCl_3$)....407
Originally assigned correct structure of mono-iodide 187{21a} ($CDCl_3$)....410
Originally assigned (incorrect) structure of 188{21a'} ($CDCl_3$)....411
Revised 188{21a'} as *N*-methylisatin 189{21a'-rev} ($CDCl_3$)....412
Originally assigned (incorrect) structure of 190{5-4} ($CDCl_3$)....413
Revised structure of 190{5-4}, i.e 193{5-4-rev} ($CDCl_3$)....414
Originally assigned (incorrect) structure of 191{5-5} ($CDCl_3$)....415
Revised structure of 191{5-5}, i.e 194{5-5-rev} ($CDCl_3$)....417
Originally assigned (incorrect) structure of 192{5-24} ($CDCl_3$)....419
Revised structure of 192{5-24}, i.e 195{5-24-rev} ($CDCl_3$)....420
Originally assigned (incorrect) structure of 196{2i} (mix DMSO-d_6 & $CDCl_3$)....421
Revised structure of 196{2i}, i.e 206{3i} (mix DMSO-d_6 & $CDCl_3$)....422
Originally assigned (incorrect) structure of 200{3b} ($CDCl_3$)....423
Revised structure of 200{3b}, i.e 207{4d} ($CDCl_3$)....424
Originally assigned (incorrect) structure of 201{4d} ($CDCl_3$)....425
Revised structure of 197{3c}, i.e 209{3c-rev}, failed bromination? ($CDCl_3$)....426
Revised structure of 198{3g}, i.e 210{3g-rev}, failed bromination? ($CDCl_3$)....427
Revised structure of 199{3h}, i.e 211{3h-rev}, failed bromination? ($CDCl_3$)....428
Revised structure of 204{4j}, i.e 212{4j-rev} ($CDCl_3$)....430
Revised structure of 205{4k}, i.e 213{4k-rev} ($CDCl_3$)....432
Correct structure of the starting material, 214{1} ($CDCl_3$)....435
Originally assigned (incorrect) structure of 216{21} as *cis*-isomer ($CDCl_3$)....437
Originally assigned (incorrect) structure of 216{21} as *trans*-isomer ($CDCl_3$)....439
Revised structure of 216{21}, i.e 217{21-rev} ($CDCl_3$)....441
Originally assigned (incorrect) structure of 221{10} ($CDCl_3$)....444
Revised structure of 221{10}, i.e 223{10-rev} ($CDCl_3$)....445
Originally assigned (incorrect) structure of 222{9} (CD_3OD); additional linear scaling is used for methanol (crmsd)....446
Revised structure of 222{9}, i.e 224{9-rev} (CD_3OD); additional linear scaling is used for methanol (crmsd)....447
Originally assigned correct structure of 225{35b} with correct referencing of the specturm ($CDCl_3$)....448
Originally assigned correct structure of 226{36b} with incorrect referencing of the specturm ($CDCl_3$)....450

Experimental procedure and spectral data for tribromide compound 227{17}: 452
Syn-7-bromonorbornene (S2) (synthetic, ^{13}C values, $CDCl_3$) 457
Syn-7-exo-2-dibromonorbornane (S3) (synthetic, ^{13}C values, $CDCl_3$) 458
Stereoconfiguration is clarified for bromoladderane 231{S47} ($CDCl_3$).. 459
Stereoconfiguration is clarified for sulfoxide 232{S42} ($CDCl_3$) 461
Stereoconfiguration is clarified for sulfoxide 233{S55} ($CDCl_3$) 463
Stereoconfiguration is clarified for acetal 234{19} ($CDCl_3$) 466
Originally assigned (incorrect) structure of 236{6} ($CDCl_3$).. 468
Revised structure of 236{6}, i.e isoindolinone 237{6-rev} ($CDCl_3$) 469
Originally proposed (incorrect) structure of 239{19a} ($CDCl_3$) 470
Revised structure of 239{19a}, i.e. zwitterion 243{19a-rev} ($CDCl_3$) 473
Originally proposed (incorrect) structure of 240{19j} ($CDCl_3$) 475
Revised structure of 240{19j}, i.e. zwitterion 244{19j-rev} ($CDCl_3$) 478
Originally proposed (incorrect) structure of 241{19q} ($CDCl_3$) 481
Revised structure of 241{19q}, i.e. zwitterion 245{19q-rev} ($CDCl_3$) 483
Originally proposed (incorrect) structure of 242{19r} ($CDCl_3$).. 486
Revised structure of 242{19r}, i.e. zwitterion 246{19r-rev} ($CDCl_3$) 488
Originally proposed correct structure of 247{3} ($CDCl_3$).. 490
Originally proposed correct structure of 248{4} ($CDCl_3$).. 493
Originally proposed correct structure of 249{5a} ($CDCl_3$).. 498

NMR computations (DU8ML)

COMPUTATIONAL DETAILS

Computations were performed using Gaussian 16, Revision C.01

M. J. Frisch, G. W. Trucks, H. B. Schlegel, G. E. Scuseria, M. A. Robb, J. R. Cheeseman, G. Scalmani, V. Barone, G. A. Petersson, H. Nakatsuji, X. Li, M. Caricato, A. V. Marenich, J. Bloino, B. G. Janesko, R. Gomperts, B. Mennucci, H. P. Hratchian, J. V. Ortiz, A. F. Izmaylov, J. L. Sonnenberg, D. Williams-Young, F. Ding, F. Lipparini, F. Egidi, J. Goings, B. Peng, A. Petrone, T. Henderson, D. Ranasinghe, V. G. Zakrzewski, J. Gao, N. Rega, G. Zheng, W. Liang, M. Hada, M. Ehara, K. Toyota, R. Fukuda, J. Hasegawa, M. Ishida, T. Nakajima, Y. Honda, O. Kitao, H. Nakai, T. Vreven, K. Throssell, J. A. Montgomery, Jr., J. E. Peralta, F. Ogliaro, M. J. Bearpark, J. J. Heyd, E. N. Brothers, K. N. Kudin, V. N. Staroverov, T. A. Keith, R. Kobayashi, J. Normand, K. Raghavachari, A. P. Rendell, J. C. Burant, S. S. Iyengar, J. Tomasi, M. Cossi, J. M. Millam, M. Klene, C. Adamo, R. Cammi, J. W. Ochterski, R. L. Martin, K. Morokuma, O. Farkas, J. B. Foresman, and D. J. Fox, Gaussian, Inc., Wallingford CT, 2019.

DU8ML and machine learning protocols.

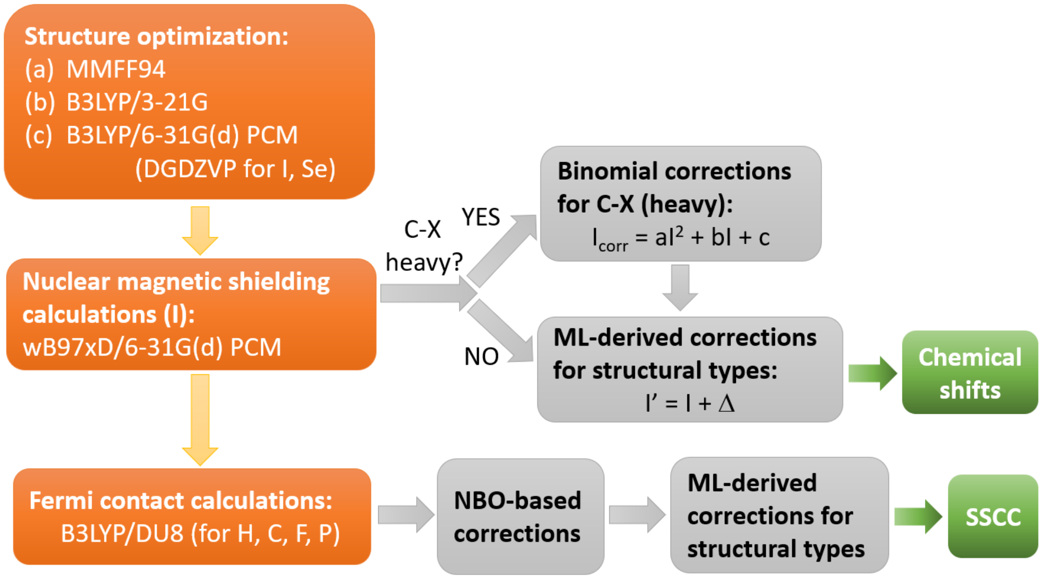

DU8ML flowchart

The training set of reliably assigned experimental ^{13}C NMR chemical shifts (36,445 entries as of Dec 2025, version: v25c), is matched with the calculated values and is analyzed/re-sorted for largest deviations from the experimental data. The structural fragments responsible for the highest deviations are "labeled" with SMARTS

strings, and a single fixed value parametric correction χ_{par} is assigned to each entry. The DFT-calculated isotropic magnetic shielding values, I_{iso}^{DFT}, are then individually corrected according to the formula, Eq S1:

$$I'_{iso} = I_{iso}^{DFT} + \chi_{par} \frac{A - I_{iso}^{DFT}}{B} \qquad \text{(Eq S1)}$$

i.e. the fixed value parametric corrections, χ_{par}, are additionally scaled by a linear function of I_{iso}^{DFT} (notice that the parameters A and B are global in the latest implementation of DU8ML (v25c); current values: A = 261.8; B = 137.64).

Due to the large size of the training set, we found it "safe" to convert the corrected magnetic shielding values I'_{iso} into ppm chemical shifts using a quadratic formula, Eq S2:

$$\delta(ppm) = a\,(I'_{iso})^2 + b\,I'_{iso} + c \qquad \text{(Eq S2)}$$

where

a = -0.00019772
b = -0.98874
c = 198.471

In the current implementation of DU8ML (v25c), with the addition of new sub-structures to the training set, any carbon atom could belong to any number of “overlapping” SMARTS substructures, with corrections applied consecutively. The individual χ_{par} values, together with the global *A*, *B* and *a*, *b*, *c* values, are reoptimized with the addition of new entries to the training set. Effectively, this could mean that as the system "learns," some of the earlier introduced χ_{par} values change their magnitude, or could decrease below a threshold, at which point they are "retired," i.e. eliminated from the consideration.

The current set of SMARTS substructures has more than a thousand entries, which translates into more than 35 points in the training set on average (for statistical significance, every SMARTS substructure corresponds to no less than 10 training set points).

ADDITIONAL LAYERS

Heavy atoms

As described above, the I_{iso}^{DFT} values calculated for carbons bearing heavy atoms are scaled by distinct quadratic functions [for details see *J. Org. Chem.* **2017**, *82* (7), 3368–3381. DOI: 10.1021/acs.joc.7b00188]

Intramolecular hydrogen bonds

Due to the limitations of smaller basis sets lacking diffuse functions (used *by design* to accelerate the DFT calculations), intramolecular H-bonds are not accurately computed, and the calculated chemical shifts of the involved carbons require additional corrections. The current implementation of DU8ML adds additional LAYER of correction to improve the chemical shift calculations in molecules containing intramolecular hydrogen bonds to carbonyl groups and to double and triple carbon-carbon bonds. This additional layer of corrections is applied using a simplified Eq S3:

$$\Delta\delta_{H-bond} = C_1 \left(\frac{\sin(\varphi)}{d}\right)^2 + C_2 \qquad \text{(Eq S3)}$$

where φ is the dihedral angle H--O--C--Z in the shown H-bonded substructure,

and *d* is the distance H----O

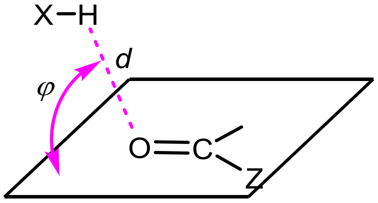

At this juncture, the H-bond training set does not have a statistically significant number of entries to introduce the angle X---H---O into the equation. As soon as we clear this threshold, the next generation of Eq S3 will have this angle as an additional argument. Nonetheless, even this first-generation approximation allows for a considerable improvement of rmsd (reduction by 0.6-0.8ppm) in molecules with intramolecular H-bonds.

Solvent.

The current implementation of DU8ML is developed to predict the absolute chemical shifts in chloroform (i.e. the calculated chemical shift values are not subjected to additional linear scaling to account for solvent effects). For these calculations we report the actual rmsd values.

For other NMR solvents, Gaussian calculations are run with a PCM in a given solvent, and additional linear scaling with *generalized* parameters for each solvent is applied, while utilizing the set of sub-structure corrections developed for chloroform (this is the current compromise; in the future, the sub-structure corrections will be separately carried out for the training set of experimental chemical shift values recorded in individual NMR solvents).

Notice that in rare cases of push-pull extended conjugated systems in polar solvents (i.e. this and other known deficiencies of DFT calculations), we use additional linear correction, applied to match the calculated and experimental values. In these cases, we report the corrected rmsd, i.e. crmsd values. WARNING: care should be taken to ensure that computed sets of chemical shifts for candidate structures are not linearly dependent. If they are linearly dependent, only generalized linear corrections should be applied. The CRMSD approach is not acceptable in these cases.

Conformer generation.

For polycyclic systems with limited conformational flexibility, individual conformers were generated using Revvity's Chem3D (via single or double dihedral drivers). For conformationally flexible molecules, initial conformational search was carried out with OpenBabel's confab using the following syntax:

>obabel <file>.mol -o <file>.sdf -confab -ecutoff <energy_cutoff>

where energy_cutoff was set to 5 kcal/mol

The structures of conformers generated with confab were then pre-optimized with OpenBabel's implementation of MMFF94 Force Field method, using SteepestDescent, and conformers accounting for ≥1% Boltzman populations were introduced into Gaussian16 DFT computations.

Gaussian 16 computations.

Template Gaussian input file:

```
=================================================================

%nprocShared=XX
%mem=YYGB
%chk=<filename>.chk
#t b3lyp/gen opt(maxcycles=512)  pop=nbo scrf(solvent=chloroform)

GEOMETRY OPTIMIZATION

0 1
< X … Y … Z  geometry specification>

@/path_to_basis/opt.gbs

--Link1--
%nprocShared=XX
%mem=YYGB
%chk=<filename>.chk
#t wB97xD/gen nmr scrf(solvent=chloroform) guess=read geom=check scf(maxcycle=512)

CHEM SHIFTS

0 1

@/path_to_basis/ppm.gbs

--Link1--
%nprocShared=XX
%mem=YYGB
%chk=<filename>.chk
#t b3lyp/gen nmr=(FCOnly,ReadAtoms) int=nobasistransform geom=check guess=read

FERMI CONTACTS

0 1

@/path_to_basis/du8.gbs

atoms=C,H



======================================================================
```

BASIS SETS USED:

(A) Geometry optimization (opt.gbs)

```
=======================================================================

-C -H -Li -Be -B -N -O -F -Cl -Br -Si -S -P
6-31G(d)
****
-As -Se -I -Te -Fe
DGDZVP
****
=======================================================================
```

(B) Chemical shifts calculations (ppm.gbs)

```
==================================================================

-C -H -Li -Be -B -N -O -F -Si -P
6-31G(d)
****
-S     0
S   6   1.00
  93413.4000000              0.0007430
  13961.7000000              0.0057930
   3169.9100000              0.0299540
    902.4560000              0.1190280
    297.1580000              0.3684320
    108.7020000              0.5772990
S   3   1.00
    108.7020000              0.1431860
     43.1553000              0.6244650
     18.1079000              0.2833660
S   1   1.00
      5.5600900              1.0000000
S   1   1.00
      2.1318300              1.0000000
S   1   1.00
      0.4204030              1.0000000
S   1   1.00
      0.1360450              1.0000000
P   4   1.00
    495.0400000              0.0083090
    117.2210000              0.0640240
     37.7749000              0.2776140
     14.0584000              0.7450760
P   2   1.00
      5.5657400              0.6137120
      2.2629700              0.4438180
P   1   1.00
      0.8079940              1.0000000
P   1   1.00
      0.2774600              1.0000000
P   1   1.00
      0.0771410              1.0000000
****
-Cl     0
S   6   1.00
 105819.0000000              0.0007380
  15872.0000000              0.0057180
   3619.6500000              0.0294950
   1030.8000000              0.1172860
    339.9080000              0.3629490
    124.5380000              0.5841490
S   3   1.00
    124.5380000              0.1341770
     49.5135000              0.6242500
     20.8056000              0.2917560
S   1   1.00
      6.5834600              1.0000000
S   1   1.00
      2.5646800              1.0000000
S   1   1.00
      0.5597630              1.0000000
S   1   1.00
      0.1832730              1.0000000
P   5   1.00
    589.7760000              0.0023910
    139.8490000              0.0185040
```

```
     45.1413000              0.0813770
     16.8733000              0.2215520
      6.7411000              0.7725690
P   2   1.00
      6.7411000             -1.5722440
      2.7715200              0.9923890
P   1   1.00
      1.0238700              1.0000000
P   1   1.00
      0.3813680              1.0000000
P   1   1.00
      0.1094370              1.0000000
****
-Se     0
S   6   1.00
 405400.0000000              0.0008310
  60850.0000000              0.0062331
  13910.0000000              0.0320710
   3989.0000000              0.1263600
   1324.0000000              0.3889900
    487.0000000              0.5488800
S   3   1.00
    487.0000000              0.1796300
    193.2000000              0.6222700
     82.1900000              0.2506700
S   1   1.00
     30.5200000              1.0000000
S   1   1.00
     13.2500000              1.0000000
S   1   1.00
      4.5100000              1.0000000
S   1   1.00
      1.8670000              1.0000000
S   1   1.00
      0.3190000              1.0000000
S   1   1.00
      0.1120000              1.0000000
P   3   1.00
   2706.0000000              0.0221460
    638.6000000              0.1818000
    203.8000000              0.8615500
P   3   1.00
     75.9600000              0.3424300
     31.0200000              0.5054100
     13.0500000              0.2623700
P   3   1.00
     13.0500000              0.0701630
      6.9860000              0.3841900
      3.2340000              0.6036800
P   1   1.00
      1.4750000              1.0000000
P   1   1.00
      0.7275000              1.0000000
P   1   1.00
      0.2869000              1.0000000
P   1   1.00
      0.0967900              1.0000000
D   4   1.00
     99.0100000              0.0255960
     28.4100000              0.1545900
      9.8630000              0.4287800
      3.5140000              0.5862000
D   1   1.00
```

```
      1.1710000              1.0000000
****
-Br     0
S   6   1.00
 439700.0000000              0.0008130
  66030.0000000              0.0062850
  15140.0000000              0.0319200
   4317.0000000              0.1288000
   1414.0000000              0.3946000
    523.9000000              0.5413000
S   3   1.00
    523.9000000              0.1831000
    207.7000000              0.6176000
     86.5400000              0.2538000
S   1   1.00
     30.5200000              1.0000000
S   1   1.00
     12.9800000              1.0000000
S   1   1.00
      4.4120000              1.0000000
S   1   1.00
      1.8620000              1.0000000
S   1   1.00
      0.3932000              1.0000000
S   1   1.00
      0.1400000              1.0000000
P   3   1.00
   2957.0000000              0.0222600
    700.3000000              0.1802000
    224.6000000              0.8624000
P   3   1.00
     82.5900000              0.3440000
     33.1900000              0.5071000
     14.2000000              0.2590000
P   3   1.00
     14.2000000              0.0796500
      7.4380000              0.3734000
      3.5260000              0.6049000
P   1   1.00
      1.5950000              1.0000000
P   1   1.00
      0.8462000              1.0000000
P   1   1.00
      0.3186000              1.0000000
P   1   1.00
      0.1096000              1.0000000
D   4   1.00
    134.8000000              0.0183100
     36.3900000              0.1350000
     12.1600000              0.4261000
      4.3410000              0.6043000
D   1   1.00
      1.5350000              1.0000000
****
-I     0
S   5   1.00
 444750.0000000              0.0008900
  66127.0000000              0.0069400
  14815.0000000              0.0360900
   4144.9000000              0.1356800
   1361.2000000              0.3387800
S   2   1.00
    508.4400000              0.4365900
```

```
    209.5900000              0.1837500
S   1   1.00
     81.9590000              1.0000000
S   1   1.00
     36.8050000              1.0000000
S   1   1.00
     13.4950000              1.0000000
S   1   1.00
      6.8859000              1.0000000
S   1   1.00
      2.5520000              1.0000000
S   1   1.00
      1.2088000              1.0000000
S   1   1.00
      0.2734000              1.0000000
S   1   1.00
      0.1009000              1.0000000
P   4   1.00
   2953.6000000              0.0122100
    712.6100000              0.0858700
    236.7100000              0.2949300
     92.6310000              0.4784900
P   1   1.00
     39.7320000              1.0000000
P   1   1.00
     17.2730000              1.0000000
P   1   1.00
      7.9570000              1.0000000
P   1   1.00
      3.1529000              1.0000000
P   1   1.00
      1.3328000              1.0000000
P   1   1.00
      0.4947000              1.0000000
P   1   1.00
      0.2160000              1.0000000
P   1   1.00
      0.0829300              1.0000000
D   3   1.00
    261.9500000              0.0314400
     76.7340000              0.1902800
     27.5510000              0.4724700
D   1   1.00
     10.6060000              1.0000000
D   1   1.00
      3.4217000              1.0000000
D   1   1.00
      1.1370000              1.0000000
****
```

===

(C) Fermi contacts calculations (du8.gbs)

===

```
-H 0
 S   1 1.00       0.000000000000
      3.5677121627D+00  1.0000000000D+00
 S   1 1.00       0.000000000000
      5.5609661054D+00  1.0000000000D+00
```

```
 S   1 1.00       0.000000000000
      3.2513472434D-01  1.0000000000D+00
 S   1 1.00       0.000000000000
      1.5497697683D-01  1.0000000000D+00
 S   1 1.00       0.000000000000
      5.6495555669D+02  1.0000000000D+00
 S   1 1.00       0.000000000000
      5.3720875566D+02  1.0000000000D+00
 S   1 1.00       0.000000000000
      6.8384961056D+03  1.0000000000D+00
 S   1 1.00       0.000000000000
      9.5989776562D+03  1.0000000000D+00
 P   1 1.00       0.000000000000
      6.2644132612D-01  1.0000000000D+00
****
-Li 0
 S   5 1.00       0.000000000000
      2.0992222050D+02  9.1681282702D-03
      1.9912294382D+01  5.3248599015D-02
      1.0571208165D+01  2.3309794883D-01
      1.3662950111D+00  6.6502285383D-01
      7.2339165393D-01  5.5219654959D-01
 SP  2 1.00       0.000000000000
      1.1469527901D+00 -8.1229779388D-01  4.8086403200D-02
      8.7197964050D-02  3.5002781299D-01  2.1894942175D+00
 SP  1 1.00       0.000000000000
      1.0494474787D-01  1.0000000000D+00  1.0000000000D+00
****
-B 0
 S   4 1.00       0.000000000000
      3.3075285200D+02  1.7994179600D-02
      4.9843865000D+01  1.2469370000D-01
      1.1117053500D+01  4.3433537500D-01
      2.9227243100D+00  5.6097937400D-01
 SP  3 1.00       0.000000000000
      5.6812646210D+00 -1.3038707790D-01  6.3742922520D-02
      1.4544045930D+00 -2.5143438980D-01  2.7613305310D-01
      4.2837857570D-01  1.2051291990D+00  3.5266045248D-01
 SP  1 1.00       0.000000000000
      2.4082906583D-01  1.0000000000D+00  1.0000000000D+00
****
-C 0
 S   4 1.00       0.000000000000
      4.4469088959D+02  1.9434935985D-02
      8.8581156759D+01  1.1630147416D-01
      1.6413457900D+01  5.7236977188D-01
      5.8916175974D+00  5.8171834809D-01
 SP  3 1.00       0.000000000000
      4.7230581216D+00 -2.6590255927D-03  3.8134851035D-02
      2.0966192600D+00 -2.1749831321D-01  3.0487741294D-01
      5.9920946704D-01  1.6298978314D+00  1.6406689248D+00
 SP  1 1.00       0.000000000000
      1.4868019395D-01  1.0000000000D+00  1.0000000000D+00
****
-N 0
 S   4 1.00       0.000000000000
      8.5671577776D+02  2.4202194035D-02
      1.3128891301D+02  1.6147165282D-01
      3.5805657642D+01  4.3535961754D-01
      3.9871155811D+00  1.8058751976D+00
 SP  3 1.00       0.000000000000
      1.4352633174D+01 -1.8084624986D-01  1.1557920417D-01
      5.0590808272D+00 -3.4070410584D-03  2.7686036839D-01
```

```
      7.5852702447D-01  1.0881032878D+00  1.8807485145D+00
 SP  1 1.00       0.000000000000
      1.1553345974D-01  1.0000000000D+00  1.0000000000D+00
****
-O 0
 S   4 1.00       0.000000000000
      9.6030652465D+02  1.8320065361D-02
      1.4841640473D+02  1.5530065069D-01
      2.7567422180D+01  3.4862465280D-01
      5.0356913036D+00  1.2607492538D+00
 SP  3 1.00       0.000000000000
      2.5396880840D+01 -3.2307639055D-01  9.7759012427D-02
      4.7124617316D+00 -1.9701959621D-01  3.4836001285D-01
      1.0560464197D+00  2.3475787192D+00  7.2002990857D-01
 SP  1 1.00       0.000000000000
      2.7492655440D-01  1.0000000000D+00  1.0000000000D+00
****
-F 0
 S   4 1.00       0.000000000000
      1.4003238415D+03  1.7860532226D-02
      1.9240943366D+02  1.1693945810D-01
      1.8962197250D+02  5.3341901092D-02
      3.3859429892D+00  1.8605877205D+00
 SP  3 1.00       0.000000000000
      2.7564697811D+01 -1.8633741160D-01  1.0879371351D-01
      4.3999920148D+00 -1.6544966913D-01  2.7892073071D-01
      1.7034233420D+00  1.1988921052D+00  6.0172044691D-01
 SP  1 1.00       0.000000000000
      3.7940977647D-01  1.0000000000D+00  1.0000000000D+00
****
-Si 0
 S   3 1.00       0.000000000000
      9.1065500000D+02  6.6082239590D-02
      1.3733600000D+02  3.8622864690D-01
      2.9760100000D+01  6.7237938540D-01
 SP  3 1.00       0.000000000000
      3.6671600000D+01 -1.0451103590D-01  1.1335501470D-01
      8.3172900000D+00  1.0741003690D-01  4.5757805930D-01
      2.2164500000D+00  9.5144632690D-01  6.0742707870D-01
 SP  2 1.00       0.000000000000
      1.0791300000D+00 -3.7610787950D-01  6.7102991120D-02
      3.0242200000D-01  1.2516495990D+00  9.5688287340D-01
 SP  1 1.00       0.000000000000
      9.3339200000D-02  1.0000000000D+00  1.0000000000D+00
 D   1 1.00       0.000000000000
      4.5000000000D-01  1.0000000000D+00
****
-P 0
 S   3 1.00       0.000000000000
      1.0549000000D+03  6.5540713550D-02
      1.5919500000D+02  3.8403607940D-01
      3.4530400000D+01  6.7454113940D-01
 SP  3 1.00       0.000000000000
      4.4286600000D+01 -1.0213005350D-01  1.1085100250D-01
      1.0101900000D+01  8.1592242710D-02  4.5649501040D-01
      2.7399700000D+00  9.6978850760D-01  6.0693601390D-01
 SP  2 1.00       0.000000000000
      1.2186500000D+00 -3.7149602190D-01  9.1582310220D-02
      3.9554600000D-01  1.2709934960D+00  9.3492410430D-01
 SP  1 1.00       0.000000000000
      1.2281100000D-01  1.0000000000D+00  1.0000000000D+00
 D   1 1.00       0.000000000000
      5.5000000000D-01  1.0000000000D+00
```

```
****
-S 0
 S   3 1.00       0.000000000000
      1.3878913540D+03  5.4640215961D-02
      1.7741809748D+02  3.3029992917D-01
      3.2879427334D+01  1.1245726976D+00
 SP  3 1.00       0.000000000000
      5.9362416872D+01 -1.1113481289D-01  1.0131287646D-01
      1.1962900000D+01  7.5393373089D-04  4.3486162799D-01
      2.5545325171D+00  7.9097386111D-01  6.0426083590D-01
 SP  2 1.00       0.000000000000
      2.9476227484D+00 -8.9765717347D-02  1.2408721801D-01
      3.1330137135D-01  1.6009530355D+00  7.3684145600D-01
 SP  1 1.00       0.000000000000
      1.2097032855D-01  1.0000000000D+00  1.0000000000D+00
 D   1 1.00       0.000000000000
      5.9228926230D-01  1.0000000000D+00
****
-Cl 0
 S   3 1.00       0.000000000000
      1.1671486318D+03  8.3204053491D-02
      1.6743704645D+02  5.7747789542D-01
      3.1311345153D+01  1.7620523607D+00
 SP  3 1.00       0.000000000000
      2.5183283483D+01 -1.2260837725D-02  3.8403044210D-02
      1.3976500000D+01  1.9272402191D-01  4.9354209788D-01
      2.7648306219D+00  7.1514915329D-01  3.2737907712D-01
 SP  2 1.00       0.000000000000
      1.1551503902D+00 -1.8094076829D-01  2.3587619779D-01
      5.2695500000D-01  6.5167458296D+01  5.8631169167D-01
 SP  1 1.00       0.000000000000
      1.6521357400D-01  1.0000000000D+00  1.0000000000D+00
 D   1 1.00       0.000000000000
      1.6230214743D+00  1.0000000000D+00
****
-Se 0
 S   3 1.00       0.000000000000
      6.1884219340D+03  6.2493402400D-02
      8.7025721000D+02  3.7236831430D-01
      1.9072949000D+02  6.8557992630D-01
 SP  3 1.00       0.000000000000
      2.5501639600D+02 -1.2851014076D-01  1.4614881100D-01
      5.5576539800D+01  9.7349750446D-02  5.5331141067D-01
      1.3468805084D+01  7.0839897376D-01  4.5320829582D-01
 SP  3 1.00       0.000000000000
      1.7781631278D+01 -2.7932246424D-01  1.7539797395D-02
      5.6695636728D+00  2.0240358839D-01  4.8336482780D-01
      1.3613980433D+00  5.6911939185D-01  6.7824136883D-01
 SP  2 1.00       0.000000000000
      6.2636075531D-01 -4.6028337137D-01  1.4416778762D-02
      4.0727913800D-01  5.2637606357D+00  1.1255484877D+01
 SP  1 1.00       0.000000000000
      2.1152701574D-01  1.0000000000D+00  1.0000000000D+00
 D   3 1.00       0.000000000000
      3.0627464000D+01  1.6084960783D-01
      7.9712764000D+00  5.1164028730D-01
      2.1348097000D+00  5.5786061652D-01
 D   1 1.00       0.000000000000
      4.3294725270D-01  1.0000000000D+00
****
-Br 0
 S   3 1.00       0.000000000000
      6.3806677088D+03  6.5266448131D-02
```

```
      9.9389843468D+02  4.1070806880D-01
      1.7398985376D+02  8.7960991753D-01
 SP  3 1.00       0.000000000000
      2.8143136383D+02 -1.2665725468D-01  1.4775138400D-01
      6.1934546824D+01  7.1839697449D-02  6.4407242010D-01
      1.9134955191D+01  1.0550546601D+00  4.1287038500D-01
 SP  3 1.00       0.000000000000
      2.8672934622D+01 -4.0211594835D-01  5.0527577578D-03
      7.1950994332D+00  1.2160548008D-01  4.8861834040D-01
      2.3238921300D+00  3.8360204708D+00  1.4964452027D-01
 SP  2 1.00       0.000000000000
      6.1887841278D-01 -1.1952883009D+00  2.3934349995D-02
      4.7394617068D-01  2.1793357577D+00  6.7090550703D-01
 SP  1 1.00       0.000000000000
      1.5826206786D-01  1.0000000000D+00  1.0000000000D+00
 D   3 1.00       0.000000000000
      3.6799144571D+01  1.1901131287D-01
      8.9998988650D+00  4.5823649440D-01
      2.4284360000D+00  7.2362745542D-01
 D   1 1.00       0.000000000000
      2.5611047866D+00  1.0000000000D+00
****
-I 0
 S   3 1.00       0.000000000000
      2.3149477207D+04  5.5199384947D-02
      1.6275611284D+03  3.4887606543D-01
      1.2541842891D+02  1.1779129171D+00
 SP  3 1.00       0.000000000000
      6.1970256507D+02 -1.1614139142D-01  1.4124373222D-01
      1.4439778402D+02  5.5887379423D-02  5.5220207568D-01
      3.3560209391D+01  6.0312233066D-01  5.7452119966D-01
 SP  3 1.00       0.000000000000
      5.6694689500D+01 -8.0770595285D-02 -1.6957321411D-01
      3.0303090564D+01  5.7277198681D-02  3.7245593031D-01
      6.2376003905D+00  4.7930368946D+00  7.3457764017D-01
 SP  3 1.00       0.000000000000
      8.1916786500D+00  2.4008713440D+00  1.0493433907D-02
      3.2445955900D+00 -7.0386049827D-01  2.5925683122D-01
      6.8681673359D-01 -2.8591806795D-02  3.0851442610D-01
 SP  2 1.00       0.000000000000
      1.9791786844D+00  6.2319575958D-02 -7.7740822178D-02
      3.2810331300D-01 -4.6823145859D+01 -3.5880753905D-01
 SP  1 1.00       0.000000000000
      1.0519190894D-01  1.0000000000D+00  1.0000000000D+00
 D   3 1.00       0.000000000000
      1.3390291107D+02  1.1586360440D-01
      3.7615234456D+01  4.7632961253D-01
      1.2047434733D+01  5.2262883559D-01
 D   3 1.00       0.000000000000
      7.0503774532D+00  2.7451605062D-01
      2.2209370000D+00  5.7202715882D-01
      1.4167624890D+00  1.0706017072D-01
 D   1 1.00       0.000000000000
      2.9503689365D-01  1.0000000000D+00
****
-Te 0
DGDZVP
****
======================================================================
```

TYPICAL COMPUTATIONAL TIMES: For a relatively large organic molecule, such as a C20-diterpene, DU8ML requires approximately 30min of computational time on a 32-core single node of a Linux cluster.

ACCURACY OF THE MATCH: The rms deviations for the correct structures (^{13}C chemical shifts) normally range from under 1.0ppm to 1.6ppm. We are using these rmsd values as the primary criterion, although work is in progress to develop a DU8 implementation of Goodman's absolute DP5 scheme (Howarth, A.; Goodman, J. *Apollo*, **2021**. https://doi.org/10.17863/CAM.65145) to attach probabilistic values to potential candidate structures.

How to read the NMR tables in the SI:

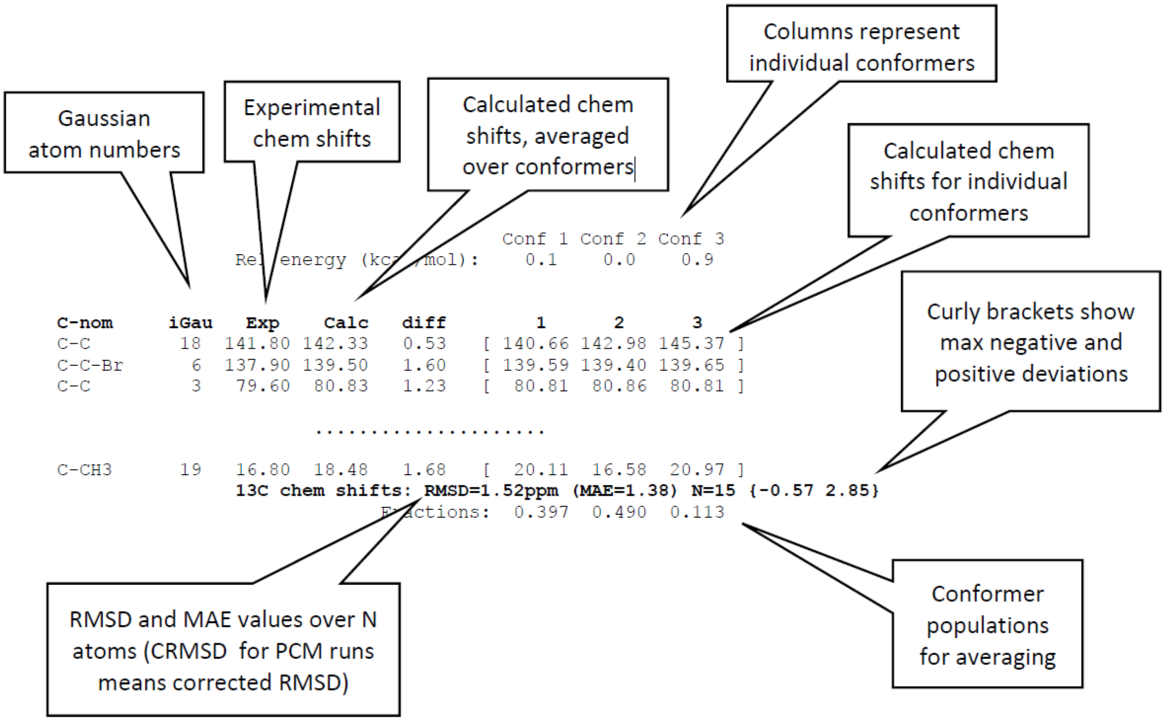

REVISED AND VALIDATED STRUCTURES

Originally assigned (correct) structure of 6{14} ($CDCl_3$)

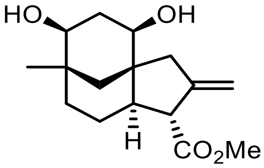

	Conf1	Conf2	Conf3	Conf4	Conf5	Conf6
Rel energy (kcal/mol):	0.00	0.23	0.98	1.15	1.59	2.67

C-nom	iGau	Exp	Calc	diff	1	2	3	4	5	6
C	17	174.10	171.90	-2.20	[171.77	172.04	171.91	172.33	171.51	171.24]
C	13	147.00	148.20	1.20	[147.53	148.89	148.20	149.60	148.32	148.38]
C	16	109.60	109.54	-0.06	[109.97	108.92	109.82	108.80	110.86	106.07]
C	5	75.40	76.71	1.31	[77.64	75.32	77.65	75.33	77.51	74.41]
C	1	75.00	74.39	-0.61	[73.54	75.62	73.51	75.58	74.52	74.52]
C	12	54.00	54.58	0.58	[53.71	54.23	56.00	56.58	61.57	60.61]
C	20	52.10	52.43	0.33	[52.50	52.38	52.41	52.31	52.35	52.32]
C	4	46.00	46.81	0.81	[46.41	47.02	46.86	47.55	48.75	46.65]
C	14	45.30	45.87	0.57	[45.06	47.13	45.12	47.23	44.85	43.74]
C	8	42.80	43.01	0.21	[42.84	42.49	44.24	43.97	44.85	44.85]
C	6	36.20	36.80	0.60	[35.95	38.60	35.97	38.59	31.07	29.76]
C	2	35.50	34.52	-0.98	[34.86	34.06	34.82	34.01	34.31	35.02]
C	3	31.80	33.48	1.68	[33.58	33.67	33.06	33.14	31.90	33.70]
C	7	30.10	32.02	1.92	[31.69	32.62	31.45	32.35	31.67	32.09]
C	9	27.90	27.60	-0.30	[27.63	27.61	27.57	27.50	27.37	27.46]
C	27	23.70	23.84	0.14	[23.74	23.82	23.51	23.58	26.44	26.23]

13C chem shifts: RMSD=1.05ppm (MAE=0.84) N=16 {-2.20 1.92}

	1	2	3	4	5	6
Fractions:	0.477	0.325	0.091	0.068	0.033	0.005

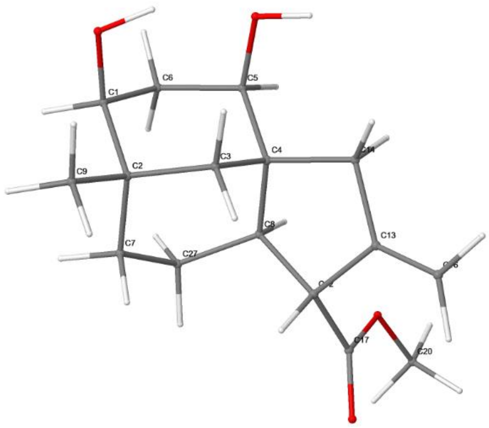

Conformer 1
Energy: -925.02397 Hartree (Rel: 0.0 kcal/mol)
XYZ coordinates for conf 1:

C	-3.11579	-0.68404	-0.44645
C	-2.05992	-1.13215	0.61446
C	-1.29428	0.11466	1.09625
C	-0.56575	0.79823	-0.07853
C	-1.60988	1.26687	-1.11785
C	-2.51643	0.13151	-1.60734
C	-1.02351	-2.14651	0.05926
C	0.48823	-0.17136	-0.69807
C	-2.79246	-1.79819	1.79170
O	-4.16740	0.07626	0.16465
O	-2.50551	2.25194	-0.55593
C	1.69543	-0.09116	0.26951

C	1.58278	1.28752	0.93997
C	0.32630	1.95929	0.42156
H	0.82927	0.26159	-1.64742
C	2.42857	1.77984	1.84543
C	3.02945	-0.24549	-0.44438
O	3.25409	0.05677	-1.59980
O	3.96808	-0.74180	0.38731
C	5.29365	-0.86497	-0.16402
H	-3.60119	-1.58311	-0.84880
H	-0.57641	-0.16907	1.87723
H	-1.98714	0.82629	1.56069
H	-1.08281	1.71647	-1.97374
H	-3.34049	0.56701	-2.18468
H	-1.96068	-0.50990	-2.29329
C	-0.00620	-1.60879	-0.97056
H	-0.47266	-2.52965	0.92954
H	-3.30157	-2.71556	1.46765
H	-2.08423	-2.07112	2.58370
H	-3.54550	-1.12678	2.21142
H	-3.85525	1.00027	0.18251
H	-1.97772	2.96256	-0.15782
H	1.64172	-0.87108	1.03733
H	-0.14883	2.58180	1.18916
H	0.58325	2.61586	-0.42519
H	2.26692	2.75378	2.30103
H	3.31170	1.23076	2.16063
H	5.28642	-1.53543	-1.02680
H	5.90537	-1.27890	0.63729
H	5.67265	0.11330	-0.46997
H	-1.54341	-3.01459	-0.36878
H	-0.43333	-1.65674	-1.97606
H	0.85877	-2.28459	-1.00103

Conformer 2
Energy: -925.02553 Hartree (Rel: 0.2 kcal/mol)
XYZ coordinates for conf 2:

C	-3.09479	-0.68161	-0.45744
C	-2.06052	-1.11973	0.61535
C	-1.28531	0.12319	1.09646
C	-0.55748	0.80549	-0.07955
C	-1.61149	1.28938	-1.10561
C	-2.49763	0.13823	-1.61366
C	-1.02687	-2.14005	0.06381
C	0.49188	-0.17017	-0.69677
C	-2.80029	-1.78422	1.79093
O	-4.08091	0.13979	0.22209
O	-2.42483	2.33084	-0.55133
C	1.70173	-0.08837	0.26845
C	1.58992	1.29335	0.93640
C	0.33336	1.96327	0.41902
H	0.83144	0.25787	-1.64885
C	2.44054	1.78367	1.83913
C	3.03457	-0.24654	-0.44510
O	3.26006	0.04801	-1.60256
O	3.97515	-0.73807	0.38881
C	5.30022	-0.86024	-0.16274
H	-3.59542	-1.57878	-0.85035
H	-0.56808	-0.17476	1.87274
H	-1.96649	0.83909	1.57051
H	-1.08633	1.74715	-1.95420
H	-3.31628	0.56456	-2.20904
H	-1.93783	-0.50990	-2.29121
C	-0.00265	-1.60934	-0.96298
H	-0.48220	-2.51743	0.93986
H	-3.33727	-2.68284	1.45972
H	-2.08706	-2.08844	2.56625
H	-3.52698	-1.10230	2.23892
H	-4.82484	0.26416	-0.38969
H	-3.12805	1.87533	-0.04993
H	1.64865	-0.86633	1.03860
H	-0.15493	2.59068	1.17043

H	0.58454	2.61646	-0.43101
H	2.28149	2.75849	2.29405
H	3.32421	1.23404	2.15247
H	5.29466	-1.53391	-1.02314
H	5.91375	-1.26959	0.63966
H	5.67708	0.11763	-0.47277
H	-1.54724	-3.00973	-0.36023
H	-0.42351	-1.66507	-1.97089
H	0.85982	-2.28878	-0.98228

Conformer 3
Energy: -925.02516 Hartree (Rel: 1.0 kcal/mol)
XYZ coordinates for conf 3:

C	-3.06204	-0.49036	-0.67229
C	-2.15925	-1.07471	0.46130
C	-1.35529	0.07950	1.08928
C	-0.45997	0.76148	0.03535
C	-1.35383	1.36655	-1.07174
C	-2.28582	0.33406	-1.71661
C	-1.15071	-2.14083	-0.04545
C	0.57532	-0.25881	-0.53631
C	-3.05808	-1.73690	1.51947
O	-4.11406	0.31810	-0.12750
O	-2.22806	2.38801	-0.54271
C	1.67630	-0.33016	0.56066
C	1.60106	1.01818	1.28908
C	0.46260	1.81850	0.68875
H	1.03437	0.19769	-1.42179
C	2.38387	1.38421	2.30426
C	3.07658	-0.61436	0.04537
O	3.83454	-1.43234	0.53064
O	3.40858	0.17270	-0.99772
C	4.74188	0.00308	-1.51268
H	-3.56670	-1.32642	-1.17435
H	-0.74635	-0.30092	1.91985
H	-2.03630	0.82073	1.52359
H	-0.70963	1.81801	-1.84253
H	-3.01086	0.86338	-2.34593
H	-1.71090	-0.31318	-2.38110
C	0.00500	-1.63715	-0.93748
H	-0.72283	-2.60995	0.85121
H	-3.60135	-2.59065	1.09326
H	-2.45787	-2.10823	2.35916
H	-3.79535	-1.02729	1.90269
H	-3.73787	1.21169	-0.02015
H	-1.69513	3.03062	-0.04763
H	1.46076	-1.13916	1.26644
H	-0.04052	2.43980	1.43916
H	0.85752	2.49194	-0.08872
H	2.25976	2.34253	2.80255
H	3.17989	0.74128	2.67263
H	5.48079	0.22632	-0.73900
H	4.82782	0.70893	-2.33867
H	4.88835	-1.02048	-1.86642
H	-1.68713	-2.94181	-0.57232
H	-0.31893	-1.60157	-1.98140
H	0.81474	-2.37864	-0.91802

Conformer 4
Energy: -925.02369 Hartree (Rel: 1.2 kcal/mol)
XYZ coordinates for conf 4:

C	3.04033	0.48267	-0.68399
C	2.16005	1.06723	0.45440
C	1.34582	-0.07612	1.09246
C	0.45067	-0.76531	0.04299
C	1.35325	-1.39472	-1.04680
C	2.26528	-0.35212	-1.71735
C	1.15576	2.13554	-0.05876
C	-0.57904	0.25593	-0.53578
C	3.06697	1.73611	1.50363
O	4.02687	-0.37594	-0.05294

O	2.13925	-2.46332	-0.50591
C	-1.68407	0.33441	0.55794
C	-1.60763	-1.00912	1.29795
C	-0.47045	-1.81366	0.70375
H	-1.03592	-0.20325	-1.42103
C	-2.39220	-1.36129	2.31730
C	-3.08262	0.61237	0.03686
O	-3.84171	1.43827	0.50774
O	-3.41433	-0.18943	-0.99561
C	-4.74761	-0.02873	-1.51201
H	3.56371	1.31076	-1.18416
H	0.73816	0.32589	1.91377
H	2.01512	-0.81697	1.54438
H	0.70994	-1.85883	-1.80611
H	2.98284	-0.87831	-2.36118
H	1.68836	0.29860	-2.37798
C	-0.00748	1.63293	-0.94198
H	0.73548	2.60781	0.83924
H	3.63282	2.56663	1.06144
H	2.46390	2.14264	2.32418
H	3.78278	1.02393	1.92097
H	4.68890	-0.59962	-0.72746
H	2.92341	-2.03750	-0.10917
H	-1.47214	1.14997	1.25747
H	0.04384	-2.43676	1.44128
H	-0.86101	-2.49006	-0.07236
H	-2.26998	-2.31473	2.82555
H	-3.18735	-0.71320	2.67917
H	-5.48615	-0.24255	-0.73522
H	-4.83313	-0.74596	-2.32825
H	-4.89637	0.98969	-1.87965
H	1.69368	2.93265	-0.58981
H	0.30919	1.59701	-1.98825
H	-0.81290	2.37895	-0.91684

Conformer 5
Energy: -925.02300 Hartree (Rel: 1.6 kcal/mol)
XYZ coordinates for conf 5:

C	-2.93403	0.69547	-0.68227
C	-2.49658	-0.76131	-0.33341
C	-1.52899	-0.69043	0.86720
C	-0.26431	0.12069	0.51752
C	-0.68405	1.56478	0.14713
C	-1.72557	1.60317	-0.97567
C	-1.76607	-1.40241	-1.56331
C	0.50330	-0.55669	-0.68967
C	-3.73520	-1.59269	0.03417
O	-3.74506	1.27186	0.34538
O	-1.29314	2.24474	1.26672
C	1.87916	-1.04876	-0.13591
C	1.72138	-0.99277	1.38002
C	0.74687	0.11362	1.69922
H	0.67356	0.18769	-1.47511
C	2.29653	-1.83250	2.24193
C	3.07057	-0.21320	-0.58497
O	4.10018	-0.67037	-1.03962
O	2.86921	1.11010	-0.40199
C	3.96409	1.96992	-0.77291
H	-3.57472	0.65150	-1.57359
H	-1.25015	-1.69853	1.19793
H	-2.03796	-0.22084	1.71658
H	0.21410	2.12131	-0.15857
H	-2.07535	2.63407	-1.10357
H	-1.24768	1.31355	-1.91638
C	-0.28788	-1.73123	-1.29669
H	-2.27800	-2.32808	-1.85210
H	-4.42797	-1.65695	-0.81514
H	-3.44699	-2.61538	0.30787
H	-4.27401	-1.14490	0.87406
H	-3.12638	1.67477	0.98336
H	-0.70312	2.17220	2.03425

```
H          2.10488       -2.06515       -0.46844
H          0.26703       -0.02718        2.67391
H          1.29354        1.06814        1.73104
H          2.11288       -1.76371        3.31140
H          2.97446       -2.61529        1.91088
H          3.62744        2.98257       -0.55213
H          4.85516        1.72528       -0.18984
H          4.18834        1.86384       -1.83704
H         -1.84233       -0.74190       -2.43717
H          0.19697       -2.05300       -2.22697
H         -0.22851       -2.58864       -0.61335

Conformer 6
Energy: -925.02127 Hartree (Rel:  2.7 kcal/mol)
XYZ coordinates for conf 6:
C          2.90812       -0.37056        0.99785
C          2.43546       -0.99329       -0.35292
C          1.73490        0.11387       -1.17263
C          0.49673        0.65274       -0.43180
C          0.95739        1.27169        0.91828
C          1.74197        0.26917        1.77411
C          1.43008       -2.16324       -0.06953
C         -0.53155       -0.51418       -0.14007
C          3.65353       -1.52675       -1.12279
O          3.96052        0.57941        0.81124
O          1.85512        2.38618        0.73213
C         -1.85873       -0.12282       -0.85148
C         -1.76449        1.38937       -1.00096
C         -0.30979        1.68350       -1.27573
H         -0.73728       -0.53771        0.93457
C         -2.75078        2.27731       -0.87849
C         -3.13641       -0.60185       -0.18731
O         -4.08894       -1.06120       -0.78742
O         -3.11796       -0.43566        1.14978
C         -4.31090       -0.83697        1.84871
H          3.34280       -1.17582        1.60562
H          1.44388       -0.27119       -2.15789
H          2.44036        0.93156       -1.36089
H          0.07051        1.62017        1.46941
H          2.14069        0.78926        2.65239
H          1.06197       -0.50187        2.14690
C         -0.00049       -1.89053       -0.56805
H          1.79234       -3.08032       -0.54899
H          4.15637       -2.32085       -0.55552
H          3.34603       -1.94934       -2.08753
H          4.38198       -0.73185       -1.30560
H          3.52328        1.43464        0.63859
H          1.45355        3.02126        0.11859
H         -1.87194       -0.56228       -1.85839
H         -0.09949        1.51657       -2.34245
H         -0.06159        2.73338       -1.07917
H         -2.56738        3.34331       -0.98724
H         -3.77677        1.97952       -0.67795
H         -4.51055       -1.89884        1.68534
H         -5.16876       -0.25269        1.50662
H         -4.11121       -0.64222        2.90219
H          1.40541       -2.38607        1.00543
H         -0.67531       -2.67821       -0.20883
H         -0.01885       -1.95509       -1.66420
```

Originally assigned (correct) structure of 7{15} ($CDCl_3$)

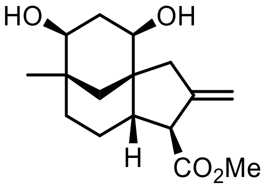

	Conf1	Conf2	Conf3	Conf4
Rel energy (kcal/mol):	0.00	0.72	0.99	1.68

C-nom	iGau	Exp	Calc	diff	1	2	3	4
C	17	174.00	171.53	-2.47	171.69	171.29	171.25	170.81
C	13	147.20	148.71	1.51	149.08	147.47	149.09	147.46
C	16	110.20	109.69	-0.51	109.54	110.40	109.23	110.11
C	1	75.60	76.28	0.68	76.92	74.25	76.77	74.11
C	5	68.30	69.14	0.84	68.67	70.95	68.29	70.64
C	20	52.00	52.32	0.32	52.27	52.41	52.35	52.49
C	12	51.60	52.15	0.55	51.90	51.27	54.36	53.66
C	8	49.90	48.61	-1.29	48.41	49.05	48.75	49.40
C	4	46.10	47.12	1.02	47.20	46.80	47.29	46.85
C	14	43.40	43.56	0.16	43.62	43.65	43.18	43.28
C	3	37.60	38.88	1.28	38.84	38.95	38.94	39.06
C	6	36.90	36.03	-0.87	36.07	35.96	35.99	35.88
C	2	36.20	35.69	-0.51	35.48	36.39	35.47	36.39
C	7	35.20	34.68	-0.52	34.74	34.49	34.71	34.46
C	9	27.60	26.48	-1.12	26.25	27.25	26.26	27.25
C	27	25.20	25.00	-0.20	24.98	24.92	25.17	25.12

13C chem shifts: RMSD=1.04ppm (MAE=0.87) N=16 {-2.47 1.51}

Fractions: 0.646 0.193 0.123 0.038

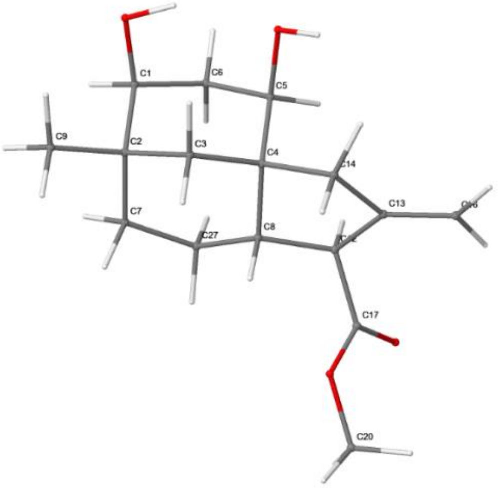

Conformer 1
Energy: -925.01956 Hartree (Rel: 0.0 kcal/mol)
XYZ coordinates for conf 1:

C	3.03962	-0.30436	0.87742
C	2.43143	-1.07344	-0.33206
C	1.70590	-0.05657	-1.24936
C	0.53679	0.57849	-0.48733
C	1.10638	1.35177	0.74799
C	2.00075	0.49948	1.66430
C	1.43256	-2.17194	0.14845
C	-0.48075	-0.55338	-0.16798
C	3.55785	-1.77757	-1.11299
O	4.01370	0.67674	0.45755
O	1.84225	2.50075	0.30291
C	-1.76111	0.17784	0.27115

C	-1.70417	1.49031	-0.54398
C	-0.37372	1.53864	-1.28418
H	-0.72246	-0.98907	-1.14861
C	-2.65307	2.42711	-0.55591
C	-3.02722	-0.60633	-0.02374
O	-3.15631	-1.43792	-0.90117
O	-4.02812	-0.24659	0.80694
C	-5.29693	-0.88776	0.57492
H	3.53283	-1.02912	1.54364
H	1.33095	-0.56909	-2.14588
H	2.41640	0.70337	-1.59374
H	0.27400	1.75707	1.33535
H	2.52851	1.17271	2.34993
H	1.40625	-0.17107	2.28548
C	0.05689	-1.70142	0.68525
H	1.24269	-2.81738	-0.71958
H	4.12713	-2.46036	-0.46899
H	3.14236	-2.36666	-1.93818
H	4.25974	-1.06404	-1.56296
H	4.69280	0.23774	-0.07800
H	2.74801	2.18584	0.12382
H	-1.75305	0.40979	1.34304
H	-0.50126	1.15566	-2.30776
H	0.03265	2.55035	-1.35574
H	-2.53524	3.34316	-1.12987
H	-3.57792	2.31411	0.00372
H	-5.20359	-1.97054	0.68939
H	-5.66176	-0.66180	-0.43012
H	-5.97186	-0.48042	1.32746
H	1.92436	-2.81047	0.89507
H	0.10932	-1.42106	1.74157
H	-0.64020	-2.54786	0.63939

Conformer 2
Energy: -925.02108 Hartree (Rel: 0.7 kcal/mol)
XYZ coordinates for conf 2:

C	-3.05600	-0.30820	-0.86653
C	-2.43497	-1.08383	0.33560
C	-1.71257	-0.06291	1.25152
C	-0.54486	0.57706	0.48831
C	-1.11039	1.33486	-0.75417
C	-2.00768	0.48537	-1.66287
C	-1.43163	-2.17858	-0.13908
C	0.47718	-0.55336	0.16440
C	-3.56502	-1.77881	1.11350
O	-4.10076	0.57353	-0.43619
O	-1.96905	2.42227	-0.33610
C	1.75759	0.17601	-0.27924
C	1.70138	1.49069	0.52838
C	0.38590	1.51983	1.29375
H	0.72035	-0.98455	1.14628
C	2.63296	2.44433	0.51826
C	3.02399	-0.60667	0.02435
O	3.14933	-1.43129	0.90836
O	4.02546	-0.25123	-0.80600
C	5.29455	-0.89112	-0.56877
H	-3.54153	-1.03646	-1.52975
H	-1.33053	-0.57013	2.14799
H	-2.42839	0.69185	1.59594
H	-0.28234	1.75550	-1.34229
H	-2.52966	1.16067	-2.35106
H	-1.40165	-0.17758	-2.28072
C	-0.05879	-1.70722	-0.68117
H	-1.23855	-2.82224	0.73018
H	-4.06215	-2.53486	0.49162
H	-3.17105	-2.28431	2.00367
H	-4.32000	-1.05363	1.42837
H	-3.66152	1.41073	-0.19540
H	-1.49913	2.96955	0.31221
H	1.75216	0.40090	-1.35242
H	0.52496	1.09985	2.30029

H	0.01978	2.54284	1.44192
H	2.51292	3.36004	1.09229
H	3.54812	2.34340	-0.05897
H	5.20008	-1.97464	-0.67359
H	5.65953	-0.65595	0.43407
H	5.96904	-0.49068	-1.32529
H	-1.92210	-2.82020	-0.88382
H	-0.11296	-1.43334	-1.73904
H	0.64505	-2.54776	-0.63063

Conformer 3
Energy: -925.02223 Hartree (Rel: 1.0 kcal/mol)
XYZ coordinates for conf 3:

C	3.03970	-0.32787	0.76052
C	2.28782	-1.17221	-0.31004
C	1.54164	-0.20651	-1.26529
C	0.47940	0.57286	-0.48113
C	1.19599	1.42634	0.61803
C	2.11867	0.61303	1.54200
C	1.27634	-2.15360	0.35957
C	-0.56804	-0.45675	0.03395
C	3.30237	-2.01906	-1.10254
O	4.02469	0.54422	0.16340
O	1.95143	2.47668	-0.00252
C	-1.75265	0.40635	0.51072
C	-1.71248	1.60428	-0.46423
C	-0.44117	1.50630	-1.29849
H	-0.91332	-0.96550	-0.87692
C	-2.63228	2.56770	-0.52224
C	-3.11746	-0.25491	0.52439
O	-3.98298	-0.01656	1.34570
O	-3.29199	-1.11136	-0.50216
C	-4.59055	-1.72540	-0.59173
H	3.54932	-1.01306	1.45569
H	1.06310	-0.77989	-2.07123
H	2.25933	0.47006	-1.74283
H	0.44229	1.93979	1.22626
H	2.74031	1.31555	2.10927
H	1.54463	0.04584	2.27539
C	-0.02021	-1.54724	0.95378
H	0.97814	-2.86846	-0.41910
H	3.88479	-2.67226	-0.43998
H	2.78589	-2.65663	-1.82879
H	4.00277	-1.39907	-1.67624
H	4.62921	0.01333	-0.37828
H	2.82052	2.09007	-0.21906
H	-1.59268	0.76354	1.53507
H	-0.66619	1.04054	-2.26959
H	0.01158	2.48117	-1.49505
H	-2.53530	3.40387	-1.21056
H	-3.51420	2.55587	0.11380
H	-5.36268	-0.96489	-0.73325
H	-4.80846	-2.29537	0.31481
H	-4.54204	-2.38627	-1.45710
H	1.79503	-2.74492	1.12649
H	0.13828	-1.16715	1.96725
H	-0.76225	-2.34999	1.05488

Conformer 4
Energy: -925.02066 Hartree (Rel: 1.7 kcal/mol)
XYZ coordinates for conf 4:

C	3.05284	-0.33580	0.74969
C	2.29005	-1.18272	-0.31498
C	1.54829	-0.21123	-1.26840
C	0.48714	0.57191	-0.48343
C	1.19929	1.40980	0.62569
C	2.12167	0.59585	1.54204
C	1.27333	-2.16225	0.34558
C	-0.56438	-0.45656	0.03490
C	3.31031	-2.01822	-1.10651
O	4.10434	0.43155	0.15048

O	2.07932	2.39242	0.03070
C	-1.74839	0.40440	0.51816
C	-1.70928	1.60613	-0.44904
C	-0.45586	1.48812	-1.30560
H	-0.91109	-0.95905	-0.87850
C	-2.60992	2.58801	-0.48775
C	-3.11463	-0.25657	0.52585
O	-3.98084	-0.01819	1.34590
O	-3.28577	-1.10885	-0.50345
C	-4.58356	-1.72526	-0.59811
H	3.55178	-1.02620	1.44279
H	1.06378	-0.77735	-2.07582
H	2.27170	0.46069	-1.74361
H	0.45102	1.93933	1.23202
H	2.73668	1.29952	2.11516
H	1.53415	0.03544	2.26958
C	-0.02002	-1.55583	0.94554
H	0.97220	-2.87311	-0.43632
H	3.81510	-2.74242	-0.45366
H	2.81546	-2.57941	-1.90866
H	4.07556	-1.37547	-1.54959
H	3.69467	1.26922	-0.13612
H	1.58540	2.91220	-0.62242
H	-1.58975	0.75483	1.54486
H	-0.69324	0.98735	-2.25519
H	-0.05035	2.47014	-1.57666
H	-2.51072	3.42433	-1.17554
H	-3.48052	2.58915	0.16345
H	-5.35625	-0.96532	-0.73901
H	-4.80180	-2.29819	0.30635
H	-4.53141	-2.38328	-1.46530
H	1.78977	-2.75880	1.10988
H	0.13936	-1.18492	1.96222
H	-0.76931	-2.35270	1.03924

Originally assigned (incorrect) structure of 4{13} ($CDCl_3$)

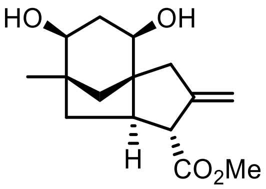

					Conf1	Conf2	Conf3	Conf4
	Rel energy (kcal/mol):				0.00	0.25	0.95	1.12

**C-nom**	**iGau**	**Exp**	**Calc**	**diff**	**1**	**2**	**3**	**4**
C	17	174.50	172.70	-1.80	[172.82	172.79	172.08	172.31]
C	13	149.60	151.21	1.61	[150.90	151.63	150.96	151.74]
C	16	109.80	110.23	0.43	[110.52	109.87	110.39	109.69]
C	5	74.50	75.99	1.49	[76.86	74.66	76.91	74.69]
C	1	70.80	75.19	4.39	[74.37	76.46	74.27	76.32]
C	12	58.90	60.29	1.39	[59.84	60.17	61.58	62.01]
C	4	56.40	58.91	2.51	[58.48	59.50	58.63	59.63]
C	20	52.20	52.47	0.27	[52.53	52.47	52.31	52.27]
C	8	46.60	49.25	2.65	[49.28	48.67	50.47	49.91]
C	2	45.50	47.60	2.10	[48.07	46.93	48.00	46.84]
C	7	45.00	43.97	-1.03	[43.70	44.19	44.15	44.57]
C	14	40.00	42.05	2.05	[41.36	42.85	41.93	43.36]
C	3	39.30	38.18	-1.12	[38.16	38.27	38.03	38.09]
C	6	37.10	34.57	-2.53	[34.56	34.55	34.63	34.65]
C	9	22.40	22.50	0.10	[22.82	22.05	22.75	21.95]

13C chem shifts: RMSD=2.00ppm (MAE=1.70) N=15 {-2.53 4.39}

Fractions: 0.499 0.325 0.100 0.075

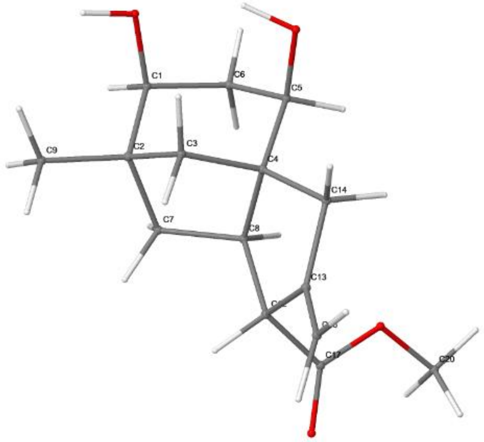

Conformer 1
Energy: -885.70999 Hartree (Rel: 0.0 kcal/mol)
XYZ coordinates for conf 1:

C	-2.93474	-0.72933	-0.70999
C	-1.92736	-1.22375	0.37449
C	-1.45703	0.00192	1.18234
C	-0.59609	0.76094	0.15295
C	-1.51556	1.37079	-0.92835
C	-2.28797	0.27816	-1.67703
C	-0.59385	-1.67900	-0.28939
C	0.27255	-0.39371	-0.45271
C	-2.57306	-2.31703	1.22791
O	-4.10707	-0.16353	-0.11308
O	-2.51677	2.24192	-0.36209
C	1.61134	-0.37302	0.33925
C	1.53610	0.85745	1.25967

C	0.41572	1.74574	0.76564
H	0.50306	-0.19262	-1.50289
C	2.30448	1.05750	2.33087
C	2.82504	-0.22554	-0.56837
O	2.84700	0.37356	-1.62624
O	3.90846	-0.81543	-0.02630
C	5.13866	-0.66442	-0.76030
H	-3.28520	-1.60124	-1.27803
H	-0.84039	-0.31650	2.03344
H	-2.28127	0.59957	1.58196
H	-0.90175	1.94489	-1.63945
H	-3.06816	0.74106	-2.29195
H	-1.60796	-0.23620	-2.36299
H	-0.09171	-2.39901	0.36817
H	-0.76098	-2.19420	-1.24305
H	-2.78993	-3.21035	0.62765
H	-1.91012	-2.61734	2.04867
H	-3.51655	-1.96369	1.65523
H	-3.89693	0.77345	0.05543
H	-2.07746	2.88002	0.22323
H	1.75361	-1.27679	0.93804
H	0.00541	2.37537	1.56368
H	0.79800	2.41498	-0.02122
H	2.18309	1.93535	2.96074
H	3.08561	0.35598	2.61251
H	5.89032	-1.20310	-0.18360
H	5.04176	-1.09416	-1.76027
H	5.40524	0.39205	-0.84535

Conformer 2
Energy: -885.71137 Hartree (Rel: 0.3 kcal/mol)
XYZ coordinates for conf 2:

C	-2.92231	-0.72325	-0.71979
C	-1.91840	-1.21979	0.36364
C	-1.44846	0.00022	1.18080
C	-0.59707	0.77240	0.15386
C	-1.53227	1.39879	-0.90788
C	-2.29316	0.30028	-1.67225
C	-0.58816	-1.66433	-0.31648
C	0.27300	-0.37362	-0.46309
C	-2.53910	-2.33670	1.21070
O	-4.05486	-0.04644	-0.13611
O	-2.43870	2.34040	-0.32503
C	1.60990	-0.36058	0.33263
C	1.53877	0.87273	1.25255
C	0.40827	1.75488	0.77224
H	0.50558	-0.16160	-1.51081
C	2.32104	1.07501	2.31365
C	2.82831	-0.22909	-0.57040
O	2.85814	0.34845	-1.63986
O	3.90922	-0.80907	-0.01069
C	5.14384	-0.66881	-0.73876
H	-3.28138	-1.59186	-1.29382
H	-0.82908	-0.32768	2.02633
H	-2.27023	0.59520	1.58902
H	-0.92149	1.97482	-1.61522
H	-3.08201	0.75822	-2.27967
H	-1.61871	-0.21382	-2.36380
H	-0.08329	-2.39440	0.32735
H	-0.76130	-2.16590	-1.27614
H	-2.81008	-3.20272	0.59372
H	-1.83280	-2.67683	1.97662
H	-3.44347	-2.00745	1.73952
H	-4.44083	-0.61477	0.54927
H	-3.20465	1.82060	-0.01896
H	1.74330	-1.26379	0.93482
H	-0.01445	2.37631	1.56786
H	0.77884	2.43516	-0.01036
H	2.20363	1.95269	2.94467
H	3.10987	0.37785	2.58491
H	5.89325	-1.19348	-0.14631

H	5.05574	-1.11859	-1.73080
H	5.40803	0.38652	-0.84323

Conformer 3

Energy: -885.71178 Hartree (Rel: 1.0 kcal/mol)

XYZ coordinates for conf 3:

C	2.86250	0.00796	-0.99674
C	2.00470	1.12835	-0.33039
C	1.47612	0.58777	1.01229
C	0.45908	-0.47900	0.55926
C	1.21799	-1.68287	-0.04163
C	2.03801	-1.26304	-1.26768
C	0.67578	1.33847	-1.11523
C	-0.32975	0.28423	-0.55999
C	2.82885	2.41099	-0.20052
O	4.01372	-0.30715	-0.20565
O	2.16272	-2.24837	0.89019
C	-1.60121	0.86140	0.14353
C	-1.57035	0.30721	1.57553
C	-0.59244	-0.84591	1.62147
H	-0.64909	-0.40740	-1.34421
C	-2.25643	0.81676	2.59920
C	-2.91133	0.48050	-0.52947
O	-3.79791	1.26145	-0.81361
O	-2.99874	-0.84929	-0.74715
C	-4.22817	-1.30967	-1.33787
H	3.25498	0.39305	-1.94713
H	0.96817	1.38496	1.57109
H	2.26281	0.18716	1.65784
H	0.49046	-2.45786	-0.32912
H	2.71361	-2.07855	-1.54970
H	1.36298	-1.10425	-2.11433
H	0.30610	2.35438	-0.93192
H	0.81585	1.25031	-2.19938
H	3.08772	2.81589	-1.18761
H	2.26980	3.18297	0.34235
H	3.76221	2.21606	0.33670
H	3.72119	-0.97571	0.44072
H	1.70659	-2.41391	1.73136
H	-1.59192	1.95405	0.16028
H	-0.17931	-0.99707	2.62543
H	-1.10464	-1.77517	1.32826
H	-2.17508	0.40260	3.60118
H	-2.92849	1.66225	2.47278
H	-4.11575	-2.38861	-1.44304
H	-5.07483	-1.07401	-0.68828
H	-4.38187	-0.84322	-2.31406

Conformer 4

Energy: -885.71026 Hartree (Rel: 1.1 kcal/mol)

XYZ coordinates for conf 4:

C	2.84755	-0.01291	-1.00391
C	1.99752	1.11541	-0.34646
C	1.46928	0.59595	1.00486
C	0.45693	-0.48161	0.56909
C	1.22786	-1.69788	0.00304
C	2.03614	-1.29177	-1.24302
C	0.66995	1.30665	-1.14026
C	-0.33239	0.26214	-0.56161
C	2.80426	2.41494	-0.24176
O	3.94715	-0.41862	-0.16310
O	2.07211	-2.29510	0.99174
C	-1.60070	0.85525	0.13533
C	-1.57038	0.31797	1.57536
C	-0.58946	-0.83104	1.63736
H	-0.65481	-0.44244	-1.33310
C	-2.26175	0.84163	2.58874
C	-2.91418	0.47476	-0.53009
O	-3.80019	1.25705	-0.81414
O	-3.00726	-0.85554	-0.74066
C	-4.24216	-1.31557	-1.31932

H	3.24630	0.35684	-1.96159
H	0.96208	1.40433	1.54807
H	2.25312	0.20447	1.65919
H	0.50185	-2.47379	-0.27342
H	2.71889	-2.10437	-1.51591
H	1.36578	-1.14172	-2.09489
H	0.30028	2.32617	-0.98001
H	0.81359	1.19604	-2.22176
H	3.11630	2.77311	-1.23090
H	2.20491	3.20319	0.22783
H	3.70764	2.29759	0.37164
H	4.45280	0.36910	0.09286
H	2.91267	-1.80217	0.96015
H	-1.58580	1.94813	0.13884
H	-0.16489	-0.97530	2.63562
H	-1.09783	-1.76644	1.35852
H	-2.18301	0.44166	3.59674
H	-2.93595	1.68342	2.44807
H	-4.13366	-2.39545	-1.41893
H	-5.08324	-1.07384	-0.66469
H	-4.40220	-0.85443	-2.29708

Revised structure of 4{13}, i.e 8{13-rev} ($CDCl_3$)

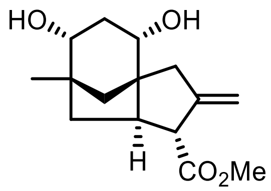

	Conf1	Conf2
Rel energy (kcal/mol):	0.00	0.20

C-nom	iGau	Exp	Calc	diff	1	2
C	17	174.50	175.54	1.04	[175.48	175.63]
C	13	149.60	150.61	1.01	[150.65	150.55]
C	16	109.80	110.85	1.05	[110.81	110.91]
C	1	74.50	74.64	0.14	[75.04	74.08]
C	5	70.80	70.97	0.17	[70.96	70.98]
C	4	58.90	57.56	-1.34	[57.61	57.50]
C	12	56.40	57.85	1.45	[57.86	57.84]
C	20	52.20	52.90	0.70	[52.90	52.90]
C	3	46.60	46.38	-0.22	[46.05	46.84]
C	2	45.50	46.15	0.65	[46.23	46.05]
C	8	45.00	45.82	0.82	[45.72	45.95]
C	6	40.00	40.33	0.33	[38.98	42.22]
C	7	39.30	39.35	0.05	[39.24	39.50]
C	14	37.10	36.44	-0.66	[36.44	36.45]
C	9	22.40	21.72	-0.68	[21.17	22.48]

13C chem shifts: RMSD=0.81ppm (MAE=0.69) N=15 {-1.34 1.45}

Fractions: 0.582 0.418

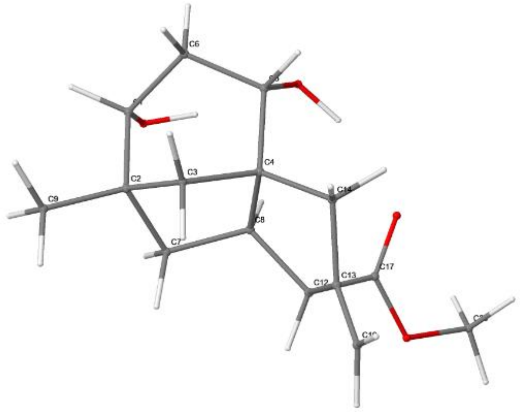

Conformer 1
Energy: -885.70450 Hartree (Rel: 0.0 kcal/mol)
XYZ coordinates for conf 1:

C	3.04625	-0.55806	-0.24628
C	2.31652	0.80896	-0.40947
C	1.63442	1.09876	0.93709
C	0.49424	0.06163	0.97344
C	1.10543	-1.31011	1.31666
C	2.13647	-1.70328	0.23352
C	1.08710	0.72035	-1.35112
C	-0.03375	0.05303	-0.51231
C	3.30951	1.89323	-0.84259
O	3.64178	-1.02035	-1.46243
O	0.12631	-2.33604	1.48165
C	-1.42862	0.73124	-0.47720
C	-1.52826	1.35297	0.92117
C	-0.69546	0.49939	1.85001
H	-0.17482	-0.97061	-0.86284
C	-2.17808	2.48015	1.21412

```
C          -2.52390       -0.32237       -0.58313
O          -2.42690       -1.46850       -0.17055
O          -3.63772        0.16301       -1.14836
C          -4.75912       -0.74157       -1.22118
H           3.83634       -0.39529        0.50621
H           1.22616        2.11802        0.94368
H           2.31128        1.01040        1.79764
H           1.60527       -1.23934        2.29147
H           2.75454       -2.53450        0.59053
H           1.59319       -2.08415       -0.63940
H           0.79677        1.73536       -1.65267
H           1.31293        0.16142       -2.26546
H           3.70068        1.70419       -1.85019
H           2.82500        2.87633       -0.86293
H           4.16213        1.95555       -0.15405
H           4.36959       -0.41885       -1.68422
H          -0.57669       -2.20426        0.81750
H          -1.58183        1.47674       -1.26187
H          -0.39011        1.03503        2.75514
H          -1.26449       -0.38486        2.16453
H          -2.17942        2.88801        2.22204
H          -2.73488        3.03243        0.46120
H          -5.55523       -0.17761       -1.70621
H          -4.49811       -1.62363       -1.81052
H          -5.06258       -1.05126       -0.21837

Conformer 2
Energy: -885.70060 Hartree (Rel:  0.2 kcal/mol)
XYZ coordinates for conf 2:
C           3.04687       -0.54989       -0.24525
C           2.32183        0.80977       -0.41678
C           1.63681        1.10600        0.92768
C           0.49423        0.07212        0.97216
C           1.10151       -1.29787        1.32852
C           2.13365       -1.69517        0.24595
C           1.08840        0.71918       -1.35604
C          -0.03166        0.05339       -0.51459
C           3.31799        1.88904       -0.85383
O           3.65174       -0.89709       -1.49453
O           0.12191       -2.32242        1.49758
C          -1.42828        0.72848       -0.48490
C          -1.52761        1.36454        0.90687
C          -0.69518        0.52007        1.84437
H          -0.17036       -0.97251       -0.85955
C          -2.17651        2.49512        1.18845
C          -2.52048       -0.32933       -0.57808
O          -2.41974       -1.47066       -0.15255
O          -3.63607        0.14572       -1.14806
C          -4.75467       -0.76307       -1.21049
H           3.83806       -0.38170        0.50525
H           1.22978        2.12564        0.92431
H           2.31273        1.02481        1.78945
H           1.60058       -1.22053        2.30310
H           2.74856       -2.52673        0.61426
H           1.58777       -2.08045       -0.62315
H           0.79901        1.73567       -1.65307
H           1.30788        0.16311       -2.27305
H           3.73663        1.66341       -1.83940
H           2.82732        2.86862       -0.90333
H           4.15298        1.96639       -0.14608
H           4.08017       -1.76078       -1.38285
H          -0.58592       -2.18709        0.83899
H          -1.58448        1.46508       -1.27730
H          -0.38891        1.06506        2.74357
H          -1.26525       -0.35995        2.16880
H          -2.17702        2.91335        2.19210
H          -2.73294        3.04023        0.43007
H          -5.55252       -0.20707       -1.70179
H          -4.49102       -1.65093       -1.78985
H          -5.05711       -1.06229       -0.20419
```

Originally assigned (incorrect) structure of 2{7a} ($CDCl_3$)

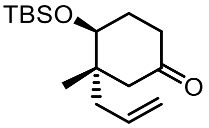

	Conf1	Conf2	Conf3	Conf4	Conf5	Conf6	Conf7	Conf8
Rel energy (kcal/mol):	0.00	0.24	0.32	0.35	0.37	0.44	0.48	0.85

C-nom	iGau	Exp	Calc	diff	1	2	3	4	5	6	7	8
C	4	211.60	211.19	-0.41	[211.63	210.32	209.86	212.29	211.01	211.64	211.92	210.51]
C	10	133.70	133.53	-0.17	[133.37	134.21	133.55	132.95	134.00	132.64	133.67	134.14]
C	11	118.00	117.13	-0.87	[115.97	116.67	117.45	118.56	117.84	117.83	116.54	117.32]
C	1	72.70	73.93	1.23	[71.92	76.97	76.57	75.00	71.90	71.28	75.15	72.34]
C	3	48.20	50.09	1.89	[51.47	51.02	50.56	46.73	50.71	51.39	47.14	49.94]
C	2	43.00	44.00	1.00	[43.96	43.17	43.19	45.17	43.56	44.85	44.90	43.42]
C	9	42.20	43.72	1.52	[43.33	46.07	45.65	41.23	44.00	43.15	41.87	43.80]
C	5	36.20	37.76	1.56	[36.40	39.40	39.33	36.50	39.37	36.48	36.47	39.27]
C	6	29.40	31.77	2.37	[31.06	31.94	32.20	31.72	32.05	31.57	31.97	32.75]
C	17	25.80	25.94	0.14	[25.94	25.87	25.87	25.92	25.93	25.91	25.94	26.39]
C	18	25.80	25.94	0.14	[25.94	25.87	25.87	25.92	25.93	25.91	25.94	26.39]
C	19	25.80	25.94	0.14	[25.94	25.87	25.87	25.92	25.93	25.91	25.94	26.39]
C	8	22.40	20.17	-2.23	[23.48	14.88	14.73	21.79	19.66	23.25	23.31	19.75]
C	15	18.00	19.22	1.22	[19.21	19.12	19.08	19.22	19.38	19.19	19.21	19.63]
C	16	-4.20	-5.77	-1.57	[-5.06	-6.80	-6.44	-5.12	-6.61	-5.05	-4.96	-6.80]
C	14	-5.00	-5.47	-0.47	[-6.66	-4.50	-4.83	-6.50	-3.32	-6.67	-6.65	-2.60]

13C chem shifts: RMSD=1.29ppm (MAE=1.06) N=16 {-2.23 2.37}

	1	2	3	4	5	6	7	8
Fractions:	0.222	0.149	0.129	0.124	0.118	0.106	0.099	0.053

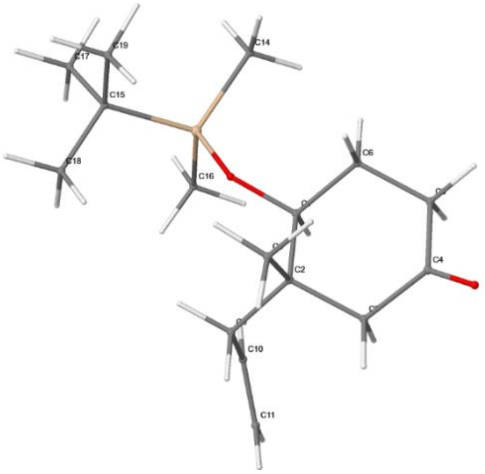

Conformer 1
Energy: -1067.75715 Hartree (Rel: 0.0 kcal/mol)
XYZ coordinates for conf 1:

C	-0.85719	-0.33579	-0.34236
C	-1.78816	-0.11374	0.88448
C	-1.91106	1.41299	1.15023
C	-2.33797	2.20045	-0.07757
C	-1.52461	1.92494	-1.33032
C	-1.35339	0.41405	-1.58874
O	0.46223	0.11574	-0.01699
C	-1.20160	-0.78891	2.13705
C	-3.21767	-0.67836	0.60525
C	-3.28891	-2.13243	0.21739
C	-3.85816	-2.59707	-0.89791
O	-3.25659	3.00481	-0.05389
Si	1.90403	-0.68210	-0.37390
C	1.92696	-2.37979	0.46137
C	3.27121	0.46756	0.31258

C	2.08134	-0.93424	-2.24177
C	4.65813	-0.16327	0.05566
C	3.20717	1.84333	-0.38574
C	3.08471	0.66930	1.83199
H	-0.84352	-1.41203	-0.56369
H	-0.92337	1.78689	1.45287
H	-2.61468	1.61575	1.96449
H	-0.53069	2.36676	-1.17408
H	-1.99001	2.43358	-2.17956
H	-2.30377	-0.02077	-1.91825
H	-0.64023	0.26129	-2.40543
H	-1.87114	-0.65178	2.99468
H	-1.06204	-1.86565	1.98832
H	-0.22906	-0.35998	2.39093
H	-3.72206	-0.08001	-0.16292
H	-3.79691	-0.52894	1.52887
H	-2.85935	-2.84596	0.92099
H	-3.90039	-3.66071	-1.11830
H	-4.31347	-1.93139	-1.62924
H	2.85365	-2.91861	0.22894
H	1.09429	-3.00261	0.11180
H	1.84710	-2.30181	1.55136
H	2.06406	0.01463	-2.79004
H	1.27016	-1.56017	-2.63384
H	3.02459	-1.44110	-2.48021
H	5.45108	0.49096	0.44622
H	4.76660	-1.13624	0.55073
H	4.85380	-0.30760	-1.01408
H	3.98779	2.50930	0.01054
H	2.24015	2.33361	-0.22484
H	3.36692	1.76197	-1.46792
H	2.11702	1.12907	2.06298
H	3.86987	1.32994	2.22831
H	3.14715	-0.27749	2.38248

Conformer 2
Energy: -1067.75638 Hartree (Rel: 0.2 kcal/mol)
XYZ coordinates for conf 2:

C	-0.70083	-0.62908	-0.28429
C	-1.75617	0.37266	0.27718
C	-3.15143	-0.05355	-0.25841
C	-3.48798	-1.50475	0.04145
C	-2.43585	-2.50619	-0.40421
C	-1.02642	-2.09123	0.06113
O	0.59207	-0.29929	0.21546
C	-1.75889	0.36491	1.81845
C	-1.41013	1.79379	-0.25828
C	-2.37191	2.89029	0.11492
C	-3.09738	3.59720	-0.75533
O	-4.52598	-1.83940	0.58861
Si	2.04499	-0.29722	-0.64045
C	2.43489	-2.02681	-1.30362
C	3.34667	0.27039	0.64161
C	1.92970	0.89171	-2.10786
C	4.74259	0.32127	-0.01872
C	2.99258	1.67519	1.17599
C	3.38401	-0.72015	1.82583
H	-0.71654	-0.52380	-1.38232
H	-3.93826	0.58621	0.14982
H	-3.16486	0.06818	-1.35292
H	-2.70685	-3.50150	-0.04061
H	-2.45946	-2.53638	-1.50426
H	-0.27902	-2.74230	-0.40402
H	-0.93078	-2.23082	1.14368
H	-0.77017	0.62637	2.20689
H	-2.48639	1.08329	2.20965
H	-2.03150	-0.61508	2.22485
H	-1.33437	1.74484	-1.35323
H	-0.41088	2.04822	0.11865
H	-2.45601	3.12580	1.17609
H	-3.76552	4.39148	-0.43210

H	-3.04530	3.41220	-1.82698
H	3.39756	-2.03553	-1.82964
H	1.67270	-2.35727	-2.02010
H	2.48455	-2.77310	-0.50265
H	1.71679	1.91840	-1.78988
H	1.13591	0.58818	-2.80158
H	2.86799	0.90292	-2.67591
H	5.49705	0.64436	0.71319
H	5.05626	-0.65960	-0.39681
H	4.77725	1.03016	-0.85533
H	3.74065	2.00186	1.91330
H	2.01418	1.68546	1.66961
H	2.97401	2.42601	0.37636
H	2.41088	-0.78846	2.32500
H	4.12148	-0.39333	2.57364
H	3.66935	-1.73002	1.50639

Conformer 3
Energy: -1067.75723 Hartree (Rel: 0.3 kcal/mol)
XYZ coordinates for conf 3:

C	0.75236	-0.67005	0.27045
C	1.80925	0.42486	-0.07086
C	3.19221	-0.07429	0.43574
C	3.56127	-1.45531	-0.08405
C	2.50486	-2.52428	0.13397
C	1.11218	-2.04350	-0.31707
O	-0.53039	-0.27154	-0.20561
C	1.86969	0.68712	-1.58730
C	1.41399	1.73375	0.67628
C	2.38443	2.87916	0.55627
C	2.11400	4.04894	-0.02814
O	4.63035	-1.68596	-0.62472
Si	-2.00193	-0.42508	0.60365
C	-2.32054	-2.23018	1.07636
C	-3.30014	0.21457	-0.64745
C	-1.98107	0.60724	2.18937
C	-4.71269	0.11658	-0.02920
C	-3.01125	1.68770	-1.00930
C	-3.25281	-0.63550	-1.93575
H	0.73257	-0.76192	1.36989
H	3.98898	0.62009	0.15558
H	3.17228	-0.13247	1.53560
H	2.80640	-3.44060	-0.38150
H	2.47979	-2.74284	1.21260
H	0.35499	-2.77290	-0.01150
H	1.06814	-1.98592	-1.41047
H	0.87564	0.93242	-1.97273
H	2.53707	1.52825	-1.80003
H	2.24455	-0.17902	-2.14359
H	1.29193	1.48635	1.74254
H	0.43193	2.05082	0.31001
H	3.36943	2.73448	1.00148
H	2.84745	4.85015	-0.07319
H	1.14467	4.25080	-0.48061
H	-3.28444	-2.33346	1.58987
H	-1.54860	-2.60131	1.76193
H	-2.33494	-2.89058	0.20198
H	-1.82044	1.67178	1.98571
H	-1.18385	0.27575	2.86615
H	-2.92875	0.50784	2.73282
H	-5.46533	0.48330	-0.74218
H	-4.98127	-0.91650	0.22381
H	-4.80692	0.72062	0.88181
H	-3.75358	2.05476	-1.73328
H	-2.02020	1.80643	-1.46178
H	-3.06103	2.34325	-0.13111
H	-2.26679	-0.59172	-2.41170
H	-3.99193	-0.26796	-2.66271
H	-3.48478	-1.68964	-1.73981

Conformer 4

Energy: -1067.75736 Hartree (Rel: 0.3 kcal/mol)
XYZ coordinates for conf 4:

C	0.60665	-0.18180	0.85176
C	1.72732	-0.64291	-0.12946
C	2.04050	0.51783	-1.11337
C	2.35854	1.82363	-0.40411
C	1.33837	2.25576	0.63537
C	0.97946	1.10798	1.60166
O	-0.60065	0.04357	0.11807
C	1.25477	-1.87212	-0.92410
C	3.00372	-1.00132	0.69968
C	4.12188	-1.63837	-0.08602
C	5.27498	-1.03650	-0.38794
O	3.35587	2.48216	-0.65689
Si	-2.15188	-0.43550	0.57534
C	-2.23690	-2.32204	0.69333
C	-3.28936	0.24427	-0.80497
C	-2.59929	0.28681	2.26691
C	-4.75658	-0.13409	-0.50228
C	-3.17134	1.78213	-0.87954
C	-2.88716	-0.35480	-2.17008
H	0.45542	-0.98774	1.58626
H	1.15075	0.68705	-1.73510
H	2.87499	0.25898	-1.77099
H	0.42929	2.55342	0.09487
H	1.71715	3.13230	1.16888
H	1.81965	0.91190	2.27745
H	0.13709	1.40996	2.23238
H	2.00040	-2.16680	-1.66965
H	1.08562	-2.72916	-0.25943
H	0.31983	-1.65714	-1.44679
H	2.69996	-1.68719	1.50498
H	3.39590	-0.10140	1.18661
H	3.96303	-2.66552	-0.41382
H	6.05401	-1.54299	-0.95223
H	5.48026	-0.01140	-0.08537
H	-3.23747	-2.65047	1.00028
H	-1.52829	-2.70312	1.43905
H	-2.00268	-2.80448	-0.26210
H	-2.57087	1.38240	2.26984
H	-1.90734	-0.06623	3.04163
H	-3.60752	-0.02239	2.56899
H	-5.41859	0.25226	-1.29078
H	-4.90159	-1.22069	-0.45982
H	-5.10352	0.28825	0.44888
H	-3.82309	2.17682	-1.67280
H	-2.14633	2.09825	-1.10487
H	-3.47283	2.26288	0.05921
H	-1.85314	-0.10413	-2.43283
H	-3.53726	0.03836	-2.96550
H	-2.98172	-1.44761	-2.18250

Conformer 5
Energy: -1067.75774 Hartree (Rel: 0.4 kcal/mol)
XYZ coordinates for conf 5:

C	-0.83652	-0.39334	-0.28064
C	-1.78027	0.40407	0.66885
C	-3.23695	0.25944	0.14915
C	-3.66481	-1.18427	-0.04454
C	-2.71371	-2.02093	-0.88382
C	-1.25388	-1.86762	-0.41400
O	0.50138	-0.29919	0.20081
C	-1.69268	-0.13089	2.11260
C	-1.39657	1.91504	0.70439
C	-1.42819	2.64720	-0.61084
C	-2.27876	3.63113	-0.91590
O	-4.69728	-1.63461	0.42515
Si	1.94781	-0.32363	-0.66397
C	2.22786	-2.00222	-1.49359
C	3.28960	0.02014	0.65746
C	1.93049	1.00715	-2.00943

C	4.68156	0.06799	-0.01172
C	3.02185	1.37263	1.35208
C	3.28093	-1.09960	1.72104
H	-0.90499	0.06771	-1.27840
H	-3.93943	0.74778	0.83262
H	-3.32159	0.76485	-0.82422
H	-3.03845	-3.06517	-0.86475
H	-2.79763	-1.66582	-1.92237
H	-0.58944	-2.36799	-1.12542
H	-1.11322	-2.36870	0.55020
H	-0.66082	-0.10015	2.47402
H	-2.30976	0.48224	2.77948
H	-2.05111	-1.16237	2.19866
H	-0.38757	1.98547	1.13102
H	-2.07548	2.41551	1.40690
H	-0.68413	2.35593	-1.35194
H	-2.24733	4.13893	-1.87672
H	-3.03475	3.97229	-0.21066
H	3.22766	-2.05001	-1.94284
H	1.50423	-2.17584	-2.29914
H	2.14154	-2.83126	-0.78223
H	1.82384	2.01550	-1.59386
H	1.10765	0.84791	-2.71746
H	2.86141	0.98157	-2.58905
H	5.45806	0.26195	0.74241
H	4.93663	-0.87881	-0.50351
H	4.75117	0.86538	-0.76195
H	3.79400	1.57160	2.10981
H	2.05013	1.38140	1.85833
H	3.03887	2.20921	0.64252
H	2.31151	-1.16926	2.22755
H	4.04436	-0.90302	2.48816
H	3.50275	-2.08105	1.28423

Conformer 6
Energy: -1067.75704 Hartree (Rel: 0.4 kcal/mol)
XYZ coordinates for conf 6:

C	0.83236	-0.18879	0.54873
C	1.84336	-0.17167	-0.63513
C	1.91251	1.27365	-1.20312
C	2.21474	2.31991	-0.14308
C	1.33816	2.25353	1.09640
C	1.21196	0.81831	1.64671
O	-0.47178	0.13167	0.05319
C	1.38723	-1.12961	-1.74759
C	3.27833	-0.58044	-0.16649
C	3.39862	-1.91469	0.52331
C	4.03506	-2.97713	0.02436
O	3.08534	3.16448	-0.28532
Si	-1.91208	-0.64374	0.46448
C	-1.82801	-2.46834	-0.02867
C	-3.25771	0.29924	-0.51572
C	-2.20918	-0.54480	2.33144
C	-4.64000	-0.33539	-0.24428
C	-3.28960	1.78064	-0.08134
C	-2.95949	0.22830	-2.02907
H	0.82654	-1.19977	0.97945
H	0.93244	1.51793	-1.63501
H	2.66149	1.34869	-1.99840
H	0.33860	2.60256	0.80236
H	1.72814	2.94529	1.84859
H	2.15591	0.51394	2.11434
H	0.45196	0.79392	2.43446
H	2.08689	-1.09427	-2.59148
H	1.35302	-2.16349	-1.38709
H	0.39430	-0.85891	-2.11497
H	3.67993	0.19749	0.49786
H	3.92213	-0.57787	-1.05523
H	2.94510	-1.99774	1.51180
H	4.10506	-3.91283	0.57331
H	4.51651	-2.94835	-0.95146

H	-2.74894	-2.99288	0.25399
H	-0.99782	-2.97623	0.47751
H	-1.68472	-2.59681	-1.10728
H	-2.25707	0.49036	2.68816
H	-1.40835	-1.05087	2.88482
H	-3.15104	-1.03654	2.60444
H	-5.42062	0.20120	-0.80285
H	-4.67969	-1.38582	-0.55806
H	-4.91239	-0.29177	0.81754
H	-4.05748	2.32805	-0.64751
H	-2.32853	2.27546	-0.26277
H	-3.52906	1.89099	0.98340
H	-1.98897	0.67645	-2.27072
H	-3.72878	0.77339	-2.59562
H	-2.95390	-0.80474	-2.39813

Conformer 7
Energy: -1067.75719 Hartree (Rel: 0.5 kcal/mol)
XYZ coordinates for conf 7:

C	0.65443	-0.06381	0.82110
C	1.78083	-0.43320	-0.19273
C	1.98134	0.76909	-1.15800
C	2.25552	2.06978	-0.42315
C	1.23824	2.42167	0.64912
C	0.95770	1.23268	1.59196
O	-0.57938	0.09542	0.11457
C	1.37675	-1.67682	-1.00081
C	3.09035	-0.73613	0.60357
C	4.26726	-1.17779	-0.22722
C	4.87889	-2.35914	-0.11734
O	3.21452	2.78086	-0.68044
Si	-2.08876	-0.48634	0.59220
C	-2.04055	-2.37283	0.72715
C	-3.28508	0.10036	-0.78126
C	-2.56910	0.21943	2.28176
C	-4.71949	-0.37437	-0.45775
C	-3.27393	1.64185	-0.87174
C	-2.85807	-0.48334	-2.14549
H	0.57088	-0.89180	1.54165
H	1.05050	0.89880	-1.72623
H	2.78763	0.58421	-1.87334
H	0.30448	2.68752	0.13463
H	1.58366	3.30212	1.19857
H	1.81455	1.07440	2.25640
H	0.10550	1.47045	2.23673
H	2.15790	-1.94196	-1.72022
H	1.23009	-2.54005	-0.33916
H	0.44610	-1.50279	-1.54621
H	2.86991	-1.50903	1.35226
H	3.39306	0.16108	1.16051
H	4.65082	-0.45328	-0.94598
H	5.74116	-2.61559	-0.72761
H	4.54338	-3.11382	0.59192
H	-3.01496	-2.76783	1.03967
H	-1.30583	-2.69714	1.47441
H	-1.77485	-2.84570	-0.22476
H	-2.61009	1.31464	2.27602
H	-1.85074	-0.08283	3.05386
H	-3.55362	-0.15041	2.59389
H	-5.41516	-0.04198	-1.24186
H	-4.78955	-1.46785	-0.40363
H	-5.08378	0.03287	0.49349
H	-3.95919	1.98257	-1.66179
H	-2.27544	2.02531	-1.11105
H	-3.59855	2.11046	0.06542
H	-1.84788	-0.16240	-2.42418
H	-3.54424	-0.14590	-2.93619
H	-2.87456	-1.58017	-2.14607

Conformer 8
Energy: -1067.75698 Hartree (Rel: 0.9 kcal/mol)

XYZ coordinates for conf 8:

C	-0.94893	-0.27660	-0.27065
C	-1.84778	0.63357	0.61795
C	-3.30028	0.58910	0.06212
C	-3.84282	-0.82311	-0.06971
C	-2.95818	-1.75662	-0.87649
C	-1.49667	-1.71026	-0.38868
O	0.37153	-0.28958	0.26736
C	-1.85086	0.15106	2.08253
C	-1.32643	2.10185	0.60687
C	-1.28632	2.79909	-0.72793
C	-0.19184	3.32160	-1.28793
O	-4.90334	-1.17348	0.42096
Si	1.81517	-0.73063	-0.48430
C	2.01822	-2.61389	-0.50858
C	3.19199	0.05853	0.59081
C	1.84262	-0.08689	-2.26282
C	4.57908	-0.33357	0.03358
C	3.07227	1.59701	0.58322
C	3.07572	-0.44405	2.04656
H	-0.92509	0.17159	-1.27588
H	-3.97384	1.17288	0.69793
H	-3.32446	1.03058	-0.94438
H	-3.36582	-2.77066	-0.83431
H	-3.00022	-1.42340	-1.92452
H	-0.87118	-2.27968	-1.08391
H	-1.41119	-2.20553	0.58516
H	-0.83334	0.12749	2.48257
H	-2.44968	0.82843	2.70236
H	-2.27965	-0.85119	2.19078
H	-0.32622	2.11201	1.05268
H	-1.97937	2.67796	1.28029
H	-2.23755	2.91487	-1.24890
H	-0.23170	3.84401	-2.24062
H	0.78244	3.25686	-0.80830
H	3.01667	-2.89396	-0.86645
H	1.29012	-3.09119	-1.17435
H	1.88854	-3.04741	0.48973
H	1.64673	0.98994	-2.31278
H	1.08645	-0.59015	-2.87792
H	2.81680	-0.27597	-2.72996
H	5.37383	0.12438	0.64024
H	4.74095	-1.41794	0.05236
H	4.72136	0.00918	-0.99900
H	3.86472	2.04520	1.20064
H	2.11019	1.92921	0.98899
H	3.17332	2.01099	-0.42778
H	2.11027	-0.17628	2.49078
H	3.86473	0.00330	2.66904
H	3.18556	-1.53336	2.11404

Revised structure of 2{7a}, i.e *Z*-isomer 9{7a-rev} ($CDCl_3$)

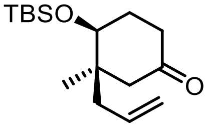

	Conf1	Conf2	Conf3	Conf4	Conf5	Conf6	Conf7	Conf8
Rel energy (kcal/mol):	0.00	0.16	0.22	0.23	0.48	0.58	1.40	2.13

C-nom	iGau	Exp	Calc	diff	1	2	3	4	5	6	7	8
C	4	211.60	211.21	-0.39	211.41	211.90	211.33	210.66	210.34	211.53	209.80	209.84
C	10	133.70	134.17	0.47	134.47	133.72	133.99	133.84	133.94	134.51	137.13	136.92
C	11	118.00	116.92	-1.08	117.13	117.15	116.21	118.08	116.85	116.30	114.26	111.10
C	1	72.70	74.86	2.16	75.28	74.88	71.66	77.16	76.73	72.32	77.77	77.20
C	3	48.20	49.41	1.21	47.33	47.73	51.27	49.83	50.28	51.24	55.51	55.49
C	2	43.00	43.89	0.89	44.13	44.38	44.09	43.51	43.24	43.68	42.87	42.39
C	9	42.20	41.23	-0.97	43.72	43.59	43.90	34.71	35.25	43.75	41.29	40.70
C	5	36.20	37.55	1.35	36.70	36.66	36.75	39.57	39.33	36.66	39.55	39.69
C	6	29.40	31.78	2.38	31.90	31.89	31.60	31.70	31.95	31.35	32.30	31.93
C	17	25.80	25.97	0.17	25.94	25.96	25.96	25.85	25.87	26.43	25.96	25.90
C	18	25.80	25.97	0.17	25.94	25.96	25.96	25.85	25.87	26.43	25.96	25.90
C	19	25.80	25.97	0.17	25.94	25.96	25.96	25.85	25.87	26.43	25.96	25.90
C	8	22.40	22.47	0.07	21.07	20.75	22.25	24.62	25.48	21.85	25.27	24.88
C	15	18.00	19.31	1.31	19.28	19.32	19.47	19.11	19.13	19.76	19.13	19.18
C	16	-4.20	-5.04	-0.84	-4.84	-4.83	-3.91	-6.51	-6.51	-3.15	-6.56	-6.60
C	14	-5.00	-6.10	-1.10	-6.71	-6.74	-6.39	-4.83	-4.89	-6.73	-5.00	-4.87

13C chem shifts: RMSD=1.14ppm (MAE=0.92) N=16 {-1.10 2.38}

	1	2	3	4	5	6	7	8
Fractions:	0.246	0.188	0.169	0.166	0.109	0.092	0.023	0.007

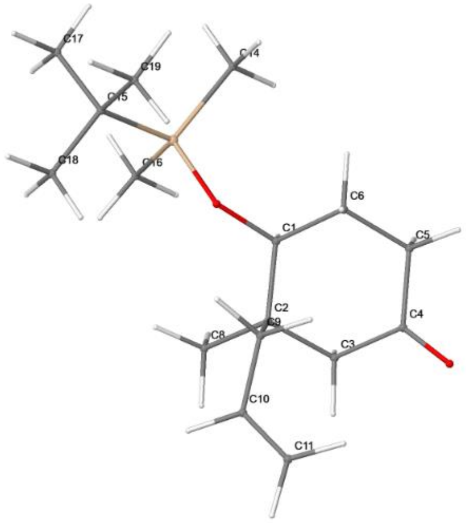

Conformer 1
Energy: -1067.75729 Hartree (Rel: 0.0 kcal/mol)
XYZ coordinates for conf 1:

C	-0.73630	-0.40133	0.98663
C	-1.95952	0.26318	0.28582
C	-2.25836	-0.50707	-1.03060
C	-2.42148	-2.00414	-0.81735
C	-1.30957	-2.67185	-0.02757
C	-0.97000	-1.89600	1.26085
O	0.42452	-0.25465	0.16280
C	-3.18696	0.20619	1.21822
C	-1.61556	1.75189	-0.01723
C	-2.67544	2.53386	-0.74642
C	-3.31327	3.60042	-0.25760
O	-3.37206	-2.62590	-1.26553
Si	1.97865	0.14446	0.68350

C	1.95789	1.83594	1.53092
C	3.03218	0.15862	-0.91393
C	2.61361	-1.13556	1.92478
C	4.48977	0.53902	-0.56997
C	3.01880	-1.23855	-1.57159
C	2.46714	1.18778	-1.91678
H	-0.58394	0.11512	1.94665
H	-1.41301	-0.36318	-1.71821
H	-3.16215	-0.13141	-1.51843
H	-0.41912	-2.68612	-0.67122
H	-1.59054	-3.70790	0.18230
H	-1.77810	-2.01188	1.99177
H	-0.07093	-2.32120	1.71856
H	-2.95399	0.63609	2.20055
H	-4.01154	0.78521	0.79090
H	-3.54976	-0.81466	1.37567
H	-0.69280	1.76499	-0.60961
H	-1.39129	2.25261	0.93473
H	-2.91141	2.20482	-1.75902
H	-4.05587	4.14038	-0.83968
H	-3.11088	3.97852	0.74308
H	2.95819	2.10525	1.89136
H	1.29085	1.83272	2.40183
H	1.61797	2.62997	0.85677
H	2.63171	-2.14696	1.50307
H	1.98290	-1.15990	2.82203
H	3.63175	-0.89121	2.25199
H	5.10472	0.54820	-1.48156
H	4.55995	1.53730	-0.12058
H	4.94954	-0.17488	0.12463
H	3.62206	-1.23279	-2.49132
H	2.00310	-1.54774	-1.84412
H	3.43878	-2.00723	-0.91134
H	1.43585	0.95011	-2.20158
H	3.07215	1.19676	-2.83536
H	2.47752	2.20655	-1.51012

Conformer 2
Energy: -1067.75690 Hartree (Rel: 0.2 kcal/mol)
XYZ coordinates for conf 2:

C	0.74808	-0.59843	-0.98727
C	1.95658	0.27691	-0.53525
C	2.34936	-0.12481	0.91268
C	2.60568	-1.61524	1.05914
C	1.50104	-2.51315	0.52816
C	1.05363	-2.10278	-0.88976
O	-0.38698	-0.30102	-0.16884
C	3.14743	0.04253	-1.48856
C	1.53595	1.77598	-0.57686
C	2.59148	2.76665	-0.16036
C	2.50486	3.56265	0.90836
O	3.61812	-2.05818	1.57901
Si	-1.98197	-0.11778	-0.68542
C	-2.09706	1.29731	-1.93612
C	-2.95988	0.26164	0.91508
C	-2.60149	-1.70056	-1.51858
C	-4.45396	0.46319	0.57628
C	-2.82320	-0.91180	1.90966
C	-2.41829	1.54575	1.57988
H	0.53062	-0.34859	-2.03714
H	1.51514	0.13326	1.57990
H	3.23276	0.42386	1.25013
H	0.64436	-2.40895	1.20831
H	1.83496	-3.55414	0.56347
H	1.82853	-2.36820	-1.61755
H	0.15790	-2.66701	-1.16890
H	2.87406	0.27525	-2.52530
H	3.99381	0.67948	-1.21349
H	3.50884	-0.99020	-1.46136
H	0.65301	1.90468	0.05586
H	1.22514	2.00115	-1.60923

H	3.47430	2.84272	-0.79564
H	3.28905	4.27258	1.15932
H	1.64186	3.53719	1.57151
H	-3.12904	1.42798	-2.28418
H	-1.48115	1.09419	-2.82090
H	-1.76215	2.25019	-1.51145
H	-2.54368	-2.56828	-0.85161
H	-2.01446	-1.93064	-2.41630
H	-3.64613	-1.59142	-1.83527
H	-5.02432	0.68115	1.49082
H	-4.61084	1.30271	-0.11229
H	-4.89825	-0.43103	0.12196
H	-3.37895	-0.69529	2.83381
H	-1.77707	-1.08798	2.18526
H	-3.22486	-1.84642	1.49929
H	-1.36156	1.44516	1.85262
H	-2.97979	1.76431	2.50010
H	-2.51489	2.41977	0.92406

Conformer 3
Energy: -1067.75543 Hartree (Rel: 0.2 kcal/mol)
XYZ coordinates for conf 3:

C	-0.93826	-0.06052	0.84719
C	-2.05498	0.39313	-0.13978
C	-2.27096	-0.72861	-1.19453
C	-2.54800	-2.08580	-0.57035
C	-1.55608	-2.51743	0.49585
C	-1.28607	-1.39871	1.52225
O	0.29201	-0.18770	0.12830
C	-3.37286	0.64304	0.62244
C	-1.64910	1.68792	-0.90303
C	-1.49698	2.92491	-0.05831
C	-2.21484	4.04152	-0.20319
O	-3.49011	-2.78369	-0.91178
Si	1.86106	0.09221	0.67946
C	2.02801	1.87107	1.30280
C	2.96444	-0.22961	-0.85125
C	2.30740	-1.07434	2.10227
C	4.44572	0.01512	-0.48528
C	2.79920	-1.68959	-1.32688
C	2.57056	0.71914	-2.00392
H	-0.83717	0.70675	1.62828
H	-1.35513	-0.82205	-1.79450
H	-3.09527	-0.47722	-1.86976
H	-0.61432	-2.75743	-0.01704
H	-1.91936	-3.43060	0.97606
H	-2.16296	-1.26215	2.16512
H	-0.46340	-1.69690	2.17946
H	-3.23025	1.34892	1.44776
H	-4.11911	1.07507	-0.05433
H	-3.80198	-0.27703	1.03214
H	-2.40629	1.87546	-1.67556
H	-0.70380	1.48705	-1.42240
H	-0.72601	2.89812	0.71184
H	-2.05012	4.91443	0.42364
H	-2.99235	4.12697	-0.96045
H	3.04566	2.05835	1.66698
H	1.34722	2.06511	2.14100
H	1.81239	2.60675	0.51995
H	2.16872	-2.12773	1.83347
H	1.69527	-0.87188	2.98956
H	3.35572	-0.94076	2.39688
H	5.08709	-0.16791	-1.35953
H	4.62511	1.04781	-0.16153
H	4.78951	-0.65205	0.31470
H	3.43225	-1.87816	-2.20638
H	1.76350	-1.90746	-1.61202
H	3.09369	-2.40926	-0.55316
H	1.52903	0.57162	-2.31106
H	3.20564	0.53516	-2.88294
H	2.69351	1.77377	-1.72827

Conformer 4
Energy: -1067.75427 Hartree (Rel: 0.2 kcal/mol)
XYZ coordinates for conf 4:

C	0.59087	-0.73520	0.24530
C	1.65821	0.33113	0.63923
C	2.98048	-0.41419	0.97425
C	3.43803	-1.35250	-0.13113
C	2.38651	-2.34292	-0.60280
C	1.04662	-1.64426	-0.90691
O	-0.63048	-0.09201	-0.10856
C	1.18332	1.08504	1.89395
C	1.88922	1.32349	-0.54322
C	2.76950	2.50437	-0.22489
C	4.00263	2.68827	-0.70368
O	4.56438	-1.31648	-0.59958
Si	-2.18760	-0.55801	0.34132
C	-2.54144	-2.33160	-0.21727
C	-3.33073	0.69880	-0.53819
C	-2.36887	-0.47988	2.22303
C	-4.80588	0.38817	-0.19958
C	-2.99999	2.13392	-0.07402
C	-3.13530	0.61001	-2.06750
H	0.43753	-1.36594	1.13740
H	3.78154	0.29948	1.18572
H	2.82468	-1.02119	1.87939
H	2.76690	-2.88811	-1.47134
H	2.23689	-3.07177	0.20829
H	0.27414	-2.39704	-1.09515
H	1.13059	-1.04853	-1.82278
H	1.96543	1.74983	2.27523
H	0.29594	1.68847	1.67864
H	0.92774	0.38083	2.69507
H	0.90282	1.68154	-0.86322
H	2.32327	0.78634	-1.39515
H	2.34672	3.25971	0.43786
H	4.59045	3.56664	-0.44885
H	4.47128	1.96446	-1.36794
H	-3.56096	-2.63043	0.05618
H	-1.85719	-3.04129	0.26410
H	-2.43715	-2.45155	-1.30153
H	-2.19469	0.52990	2.61096
H	-1.65596	-1.15159	2.71691
H	-3.37411	-0.79113	2.53268
H	-5.47007	1.10549	-0.70316
H	-5.10219	-0.61533	-0.52927
H	-5.00586	0.46064	0.87668
H	-3.65169	2.85896	-0.58344
H	-1.96233	2.40337	-0.30130
H	-3.15192	2.26127	1.00494
H	-2.10063	0.82672	-2.35602
H	-3.78371	1.33753	-2.57753
H	-3.39131	-0.38331	-2.45619

Conformer 5
Energy: -1067.75741 Hartree (Rel: 0.5 kcal/mol)
XYZ coordinates for conf 5:

C	0.67271	-0.77385	0.23458
C	1.73752	0.31238	0.58193
C	3.06362	-0.42824	0.91844
C	3.51941	-1.36464	-0.18820
C	2.47525	-2.37794	-0.62617
C	1.11427	-1.70839	-0.90394
O	-0.56136	-0.15337	-0.11485
C	1.28220	1.09966	1.82159
C	1.92913	1.28198	-0.62465
C	2.90049	2.41170	-0.40097
C	2.57756	3.70710	-0.41160
O	4.63458	-1.30883	-0.68026
Si	-2.10414	-0.61939	0.38152
C	-2.44824	-2.41603	-0.10548

```
C          -3.27736        0.59170       -0.52204
C          -2.25038       -0.47461        2.26234
C          -4.74300        0.27483       -0.14977
C          -2.95781        2.04603       -0.11311
C          -3.10378        0.45174       -2.05013
H           0.54237       -1.38208        1.14570
H           3.86811        0.27700        1.14432
H           2.90118       -1.03691        1.82113
H           2.84380       -2.92406       -1.49920
H           2.36053       -3.10189        0.19487
H           0.34969       -2.47857       -1.04912
H           1.16010       -1.13679       -1.83757
H           2.04492        1.82262        2.12770
H           0.35964        1.65084        1.61534
H           1.09515        0.42438        2.66555
H           0.94724        1.68948       -0.88711
H           2.27440        0.70665       -1.49554
H           3.94386        2.13418       -0.24689
H           3.32332        4.48454       -0.26535
H           1.55345        4.03940       -0.57262
H          -3.45786       -2.71610        0.20136
H          -1.74528       -3.10052        0.38535
H          -2.36656       -2.57380       -1.18684
H          -2.07730        0.54990        2.61011
H          -1.52211       -1.12219        2.76601
H          -3.24668       -0.78223        2.60287
H          -5.42402        0.96652       -0.66660
H          -5.03155       -0.74280       -0.44078
H          -4.92737        0.38123        0.92648
H          -3.62588        2.74500       -0.63757
H          -1.92697        2.31984       -0.36502
H          -3.09552        2.20917        0.96291
H          -2.07598        0.66951       -2.36147
H          -3.76810        1.15371       -2.57527
H          -3.35396       -0.55739       -2.40003

Conformer 6
Energy: -1067.75766 Hartree (Rel:  0.6 kcal/mol)
XYZ coordinates for conf 6:
C          -1.02721        0.08974        0.81636
C          -2.05823        0.73696       -0.15293
C          -2.42392       -0.29921       -1.25180
C          -2.90512       -1.62261       -0.67882
C          -2.01630       -2.22675        0.39580
C          -1.59191       -1.19024        1.45595
O           0.16791       -0.20835        0.08757
C          -3.33843        1.13067        0.61824
C          -1.46396        1.99224       -0.85350
C          -1.16379        3.16625        0.04192
C           0.02029        3.77749        0.13382
O          -3.92547       -2.16970       -1.06706
Si          1.73182       -0.41862        0.68641
C           2.11897        0.91328        1.97186
C           2.86669       -0.29593       -0.85356
C           1.93034       -2.11881        1.49938
C           4.33392       -0.55437       -0.44258
C           2.44605       -1.34969       -1.90146
C           2.77158        1.10663       -1.49013
H          -0.80066        0.81849        1.60720
H          -1.52487       -0.50484       -1.84885
H          -3.19492        0.09333       -1.92300
H          -1.11465       -2.60167       -0.10819
H          -2.52940       -3.08241        0.84416
H          -2.44685       -0.93117        2.09032
H          -0.84117       -1.63191        2.11849
H          -3.10894        1.70730        1.52112
H          -3.98620        1.74588       -0.01730
H          -3.92558        0.25912        0.92446
H          -2.19923        2.31139       -1.60852
H          -0.55807        1.70274       -1.39451
H          -1.99699        3.55810        0.62580
```

```
H          0.16776        4.64315        0.77496
H          0.88313        3.44260       -0.43800
H          3.16942        0.85925        2.28303
H          1.50828        0.78162        2.87355
H          1.92953        1.92173        1.58863
H          1.60204       -2.93000        0.83915
H          1.35482       -2.19202        2.42947
H          2.98061       -2.30458        1.75590
H          4.99205       -0.47290       -1.31978
H          4.68956        0.17298        0.29798
H          4.47564       -1.55739       -0.02287
H          3.09491       -1.28753       -2.78752
H          1.41322       -1.19737       -2.23484
H          2.52567       -2.37147       -1.51028
H          1.75229        1.33189       -1.82303
H          3.42875        1.17199       -2.36981
H          3.08106        1.89492       -0.79269

Conformer 7
Energy: -1067.75673 Hartree (Rel:  1.4 kcal/mol)
XYZ coordinates for conf 7:
C          0.89987       -0.64682        0.14933
C          1.84821        0.47179        0.67681
C          3.21435       -0.21147        0.98189
C          3.79658       -0.95438       -0.21057
C          2.85006       -1.94261       -0.87333
C          1.44867       -1.34410       -1.10546
O         -0.40380       -0.13608       -0.11661
C          1.29486        1.06289        1.98259
C          2.11937        1.61709       -0.35064
C          0.95324        2.48485       -0.74690
C          0.87469        3.79595       -0.50438
O          4.94234       -0.78415       -0.59382
Si        -1.86253       -0.86371        0.32175
C         -1.98713       -2.60000       -0.42289
C         -3.21465        0.29617       -0.37539
C         -1.96899       -1.01469        2.20490
C         -4.61029       -0.28433       -0.05363
C         -3.09632        1.69832        0.26112
C         -3.06655        0.42511       -1.90708
H          0.84384       -1.39840        0.95536
H          3.94695        0.52170        1.33342
H          3.06959       -0.94603        1.78879
H          3.29971       -2.30170       -1.80344
H          2.76398       -2.80548       -0.19502
H          0.75634       -2.13791       -1.40386
H          1.47497       -0.62710       -1.93392
H          1.99875        1.79090        2.40376
H          0.34416        1.57337        1.81170
H          1.13513        0.27797        2.73190
H          2.55877        1.18369       -1.26079
H          2.90130        2.25617        0.08079
H          0.14400        1.99208       -1.27959
H          0.02425        4.38846       -0.83292
H          1.65996        4.32868        0.02977
H         -2.93670       -3.07534       -0.14763
H         -1.18297       -3.24794       -0.05272
H         -1.92556       -2.58525       -1.51701
H         -1.90883       -0.03836        2.69847
H         -1.15568       -1.63611        2.59951
H         -2.91203       -1.48692        2.50643
H         -5.39566        0.37731       -0.44690
H         -4.76086       -1.27213       -0.50635
H         -4.77782       -0.38083        1.02629
H         -3.86683        2.36799       -0.14867
H         -2.11980        2.15433        0.06404
H         -3.23821        1.66659        1.34845
H         -2.09296        0.84306       -2.18693
H         -3.84347        1.09126       -2.31010
H         -3.17313       -0.54289       -2.41209
```

Conformer 8
Energy: -1067.75731 Hartree (Rel: 2.1 kcal/mol)
XYZ coordinates for conf 8:

C	0.90895	-0.61501	0.08687
C	1.84428	0.31464	0.91402
C	3.21431	-0.41578	1.03125
C	3.80936	-0.79605	-0.31691
C	2.87075	-1.54889	-1.24670
C	1.47020	-0.90799	-1.31267
O	-0.39767	-0.06119	-0.01483
C	1.27250	0.50878	2.32842
C	2.11618	1.69768	0.24548
C	0.94215	2.60132	-0.02432
C	0.73720	3.24825	-1.17440
O	4.96090	-0.53174	-0.62135
Si	-1.84857	-0.90578	0.14489
C	-1.91590	-2.35701	-1.06963
C	-3.20996	0.38416	-0.23201
C	-1.99914	-1.59836	1.89991
C	-4.60014	-0.28188	-0.12508
C	-3.13516	1.55175	0.77554
C	-3.02622	0.94224	-1.66029
H	0.86387	-1.56918	0.64045
H	3.93934	0.19809	1.57525
H	3.07360	-1.34509	1.60407
H	3.33296	-1.62636	-2.23498
H	2.77817	-2.57053	-0.84644
H	0.78597	-1.57936	-1.84123
H	1.50076	0.02368	-1.88795
H	1.95398	1.10918	2.94295
H	0.30178	1.01040	2.30236
H	1.13434	-0.45764	2.82848
H	2.67842	1.56455	-0.68724
H	2.80283	2.22556	0.92635
H	0.25459	2.76992	0.80303
H	-0.09854	3.93092	-1.30505
H	1.39897	3.12129	-2.02960
H	-2.86045	-2.90562	-0.96816
H	-1.10586	-3.07174	-0.87820
H	-1.83055	-2.02831	-2.11156
H	-1.98016	-0.80674	2.65728
H	-1.17640	-2.28915	2.12254
H	-2.93410	-2.15892	2.02187
H	-5.38980	0.45281	-0.33960
H	-4.71962	-1.10492	-0.84045
H	-4.79103	-0.67981	0.87940
H	-3.91866	2.29276	0.55842
H	-2.16916	2.06601	0.72561
H	-3.28468	1.21305	1.80826
H	-2.05185	1.42808	-1.78151
H	-3.80354	1.68901	-1.88010
H	-3.10721	0.15524	-2.42030

Originally assigned (incorrect) structure of 13{4a'} ($CDCl_3$)

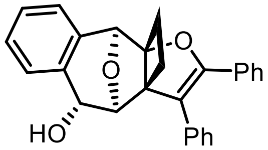

					Conf1	Conf2
Rel energy (kcal/mol):					0.00	0.47

C-nom	iGau	Exp	Calc	diff	1	2
C	13	155.00	156.79	1.79	[156.83	156.69]
C	5	136.80	136.04	-0.76	[136.23	135.62]
C	15	134.80	134.83	0.03	[135.28	133.83]
C	4	134.10	134.80	0.70	[134.72	134.99]
C	21	132.00	131.24	-0.76	[131.24	131.23]
C	3	129.20	130.38	1.18	[130.41	130.32]
C	24	128.60	128.62	0.02	[128.48	128.93]
C	22	128.50	128.33	-0.17	[128.10	128.85]
C	26	128.50	128.33	-0.17	[128.10	128.85]
C	16	128.40	127.05	-1.35	[127.49	126.09]
C	20	128.40	127.05	-1.35	[127.49	126.09]
C	2	128.40	127.51	-0.89	[127.47	127.59]
C	17	128.40	127.45	-0.95	[127.45	127.45]
C	19	127.50	127.45	-0.05	[127.45	127.45]
C	1	127.50	127.38	-0.12	[127.38	127.39]
C	23	127.20	127.08	-0.12	[127.04	127.17]
C	25	126.20	127.08	0.88	[127.04	127.17]
C	18	126.00	125.21	-0.79	[125.38	124.84]
C	6	125.10	125.06	-0.04	[125.01	125.18]
C	12	116.00	116.40	0.40	[116.45	116.29]
C	10	94.60	94.23	-0.37	[94.12	94.46]
C	8	83.70	88.62	4.92	[88.10	89.76]
C	11	83.70	84.50	0.80	[84.74	83.96]
C	7	68.90	68.25	-0.65	[68.23	68.29]
C	9	63.30	63.47	0.17	[63.83	62.67]
C	30	25.90	25.77	-0.13	[26.80	23.50]
C	29	25.20	25.73	0.53	[26.28	24.52]

13C chem shifts: RMSD=1.20ppm (MAE=0.74) N=27 {-1.35 4.92}

Fractions: 0.687 0.313

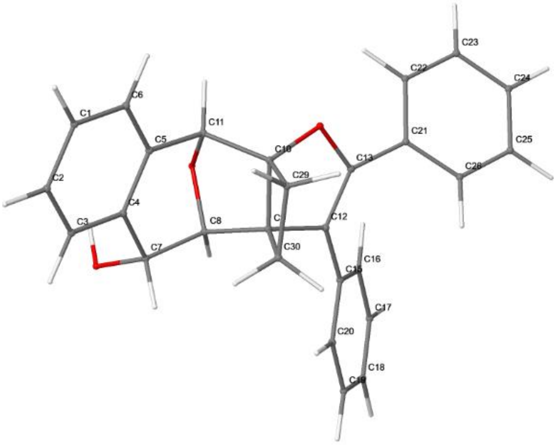

Conformer 1
Energy: -1267.71712 Hartree (Rel: 0.0 kcal/mol)
XYZ coordinates for conf 1:

C	5.43578	-1.72938	0.32922
C	5.84073	-0.39748	0.46248
C	4.93935	0.63302	0.19551
C	3.62534	0.34801	-0.19367

C	3.22353	-0.99223	-0.33275
C	4.13074	-2.02352	-0.06800
C	2.66022	1.47572	-0.54575
C	1.23540	0.94137	-0.75622
C	0.48276	0.36899	0.47459
C	0.79856	-1.14083	0.42743
C	1.79230	-1.26473	-0.74670
C	-1.02920	0.38049	0.31271
C	-1.44626	-0.90444	0.15562
O	-0.42571	-1.84156	0.15691
C	-1.79617	1.63380	0.19688
C	-2.86093	1.77801	-0.71614
C	-3.54639	2.98501	-0.83789
C	-3.17817	4.09053	-0.06513
C	-2.11058	3.97390	0.82701
C	-1.42767	2.76374	0.95430
C	-2.78266	-1.50984	0.01939
C	-2.94633	-2.66720	-0.76211
C	-4.19825	-3.26999	-0.88153
C	-5.30249	-2.73600	-0.21300
C	-5.14544	-1.59595	0.58102
C	-3.89668	-0.98893	0.70042
O	1.34487	-0.21253	-1.62696
O	3.09654	2.15323	-1.72959
C	1.22589	-1.13006	1.91520
C	1.06372	0.42312	1.93367
H	6.13667	-2.53564	0.52741
H	6.85671	-0.16464	0.76920
H	5.25383	1.66996	0.28452
H	3.81586	-3.05854	-0.18078
H	0.65464	1.69425	-1.29285
H	1.68962	-2.21359	-1.27844
H	-3.14030	0.93839	-1.34399
H	-4.36182	3.06700	-1.55195
H	-3.71013	5.03257	-0.16555
H	-1.80619	4.82660	1.42842
H	-0.60219	2.69247	1.65620
H	-2.08730	-3.08677	-1.27498
H	-4.31014	-4.15952	-1.49549
H	-6.27686	-3.20824	-0.30332
H	-5.99582	-1.18383	1.11736
H	-3.77817	-0.11388	1.33063
H	0.49575	-1.65299	2.53734
H	2.22421	-1.51496	2.13412
H	0.36775	0.80243	2.68556
H	2.00826	0.96494	2.02611
H	2.66675	2.24627	0.23215
H	3.05069	1.49375	-2.44404

Conformer 2
Energy: -1267.71637 Hartree (Rel: 0.5 kcal/mol)
XYZ coordinates for conf 2:

C	5.44335	-1.56581	0.70432
C	5.83295	-0.23140	0.55221
C	4.92928	0.70326	0.04674
C	3.62793	0.32151	-0.29981
C	3.24179	-1.02254	-0.15275
C	4.15167	-1.95811	0.35084
C	2.65954	1.33426	-0.90349
C	1.24729	0.74029	-1.02351
C	0.48905	0.42932	0.30193
C	0.78700	-1.06296	0.54195
C	1.82835	-1.40383	-0.54230
C	-1.02905	0.42648	0.19293
C	-1.43776	-0.87187	0.13058
O	-0.42612	-1.79886	0.31281
C	-1.83086	1.65860	0.28113
C	-3.13481	1.66849	0.82179
C	-3.86320	2.85026	0.93273
C	-3.30873	4.06802	0.52661
C	-2.00915	4.08477	0.01824

C	-1.28066	2.90035	-0.09764
C	-2.74614	-1.49883	-0.12559
C	-3.08184	-2.70419	0.51542
C	-4.30147	-3.32872	0.25509
C	-5.19741	-2.76767	-0.65805
C	-4.86403	-1.57870	-1.31433
C	-3.64760	-0.95067	-1.05484
O	1.39388	-0.56553	-1.63146
O	3.11462	1.75644	-2.19341
C	1.15185	-0.77969	2.01986
C	1.06688	0.75224	1.72330
H	6.14625	-2.29940	1.08943
H	6.83887	0.07719	0.82308
H	5.23205	1.73910	-0.08522
H	3.85007	-2.99781	0.45652
H	0.66760	1.34445	-1.72335
H	1.75849	-2.44337	-0.87045
H	-3.57403	0.74082	1.17167
H	-4.86488	2.82143	1.35402
H	-3.87686	4.98957	0.61794
H	-1.55482	5.02299	-0.28959
H	-0.26949	2.94739	-0.48860
H	-2.38181	-3.14488	1.21754
H	-4.55024	-4.25612	0.76375
H	-6.14563	-3.25673	-0.86364
H	-5.54838	-1.14544	-2.03853
H	-3.38547	-0.03728	-1.57863
H	0.36847	-1.13551	2.69339
H	2.11788	-1.15782	2.36181
H	0.38841	1.31985	2.36479
H	2.04046	1.24738	1.71731
H	2.63892	2.25006	-0.30310
H	3.09987	0.96067	-2.75376

Revised structure of 13{4a'}, i.e epimer 14{4a'-rev} (CDCl$_3$)

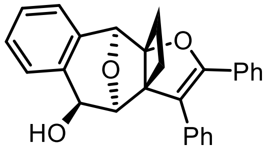

	Conf1	Conf2
Rel energy (kcal/mol):	0.00	0.73

C-nom	iGau	Exp	Calc	diff	1	2
C	13	155.00	156.79	1.79	[156.70	157.09]
C	4	136.80	137.29	0.49	[137.33	137.15]
C	5	134.80	135.19	0.39	[135.15	135.34]
C	15	134.10	133.67	-0.43	[133.33	134.86]
C	21	132.00	131.41	-0.59	[131.41	131.43]
C	24	129.20	128.96	-0.24	[129.06	128.60]
C	22	128.60	128.72	0.12	[128.88	128.17]
C	26	128.50	128.72	0.22	[128.88	128.17]
C	2	128.50	127.64	-0.86	[127.68	127.50]
C	17	128.40	127.49	-0.91	[127.52	127.38]
C	19	128.40	127.49	-0.91	[127.52	127.38]
C	23	128.40	127.20	-1.20	[127.22	127.11]
C	25	128.40	127.20	-1.20	[127.22	127.11]
C	1	127.50	126.77	-0.73	[126.79	126.69]
C	3	127.50	125.97	-1.53	[126.00	125.87]
C	16	127.20	125.88	-1.32	[125.48	127.26]
C	20	126.20	125.88	-0.32	[125.48	127.26]
C	6	126.00	125.25	-0.75	[125.30	125.08]
C	18	125.10	124.82	-0.28	[124.69	125.27]
C	12	116.00	116.57	0.57	[116.49	116.83]
C	10	94.60	95.64	1.04	[95.67	95.55]
C	8	83.70	85.57	1.87	[85.90	84.43]
C	11	83.70	84.65	0.95	[84.52	85.08]
C	7	68.90	70.90	2.00	[70.95	70.72]
C	9	63.30	63.69	0.39	[63.37	64.77]
C	30	25.90	25.90	0.00	[25.15	28.50]
C	29	25.20	24.50	-0.70	[24.11	25.83]

13C chem shifts: RMSD=0.97ppm (MAE=0.81) N=27 {-1.53 2.00}

Fractions: 0.775 0.225

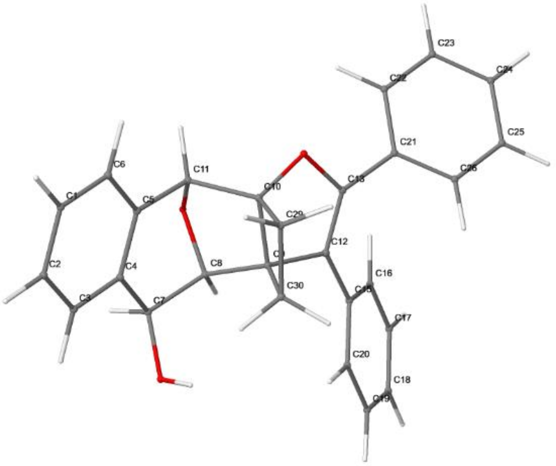

Conformer 1
Energy: -1267.71250 Hartree (Rel: 0.0 kcal/mol)
XYZ coordinates for conf 1:

C	5.41528	-1.63234	0.60590
C	5.80611	-0.31262	0.36765
C	4.90582	0.58908	-0.20360
C	3.60814	0.18554	-0.53385

C	3.21253	-1.14422	-0.28605
C	4.12076	-2.04327	0.27969
C	2.63438	1.15171	-1.20494
C	1.20819	0.57608	-1.19457
C	0.52679	0.34641	0.19326
C	0.77950	-1.14627	0.47634
C	1.78656	-1.54520	-0.61938
C	-0.99282	0.39945	0.15180
C	-1.44900	-0.88505	0.12963
O	-0.46360	-1.84435	0.29585
C	-1.72511	1.67405	0.21961
C	-3.05104	1.76718	0.69502
C	-3.70425	2.99408	0.78009
C	-3.05224	4.17476	0.41098
C	-1.73002	4.10803	-0.03044
C	-1.07390	2.87970	-0.11911
C	-2.78475	-1.47493	-0.06859
C	-3.14797	-2.63671	0.63487
C	-4.39358	-3.22892	0.42815
C	-5.28836	-2.67986	-0.49357
C	-4.92796	-1.53584	-1.21217
C	-3.68542	-0.93959	-1.00569
O	1.31449	-0.76101	-1.72958
O	2.70246	2.49166	-0.71851
C	1.18153	-0.83385	1.93709
C	1.16825	0.68735	1.58652
H	6.11573	-2.34089	1.03916
H	6.81166	0.01274	0.61990
H	5.20332	1.61686	-0.39173
H	3.81463	-3.07073	0.46328
H	0.57411	1.14375	-1.87800
H	1.70872	-2.59965	-0.89455
H	-3.56924	0.87028	1.01475
H	-4.72565	3.02894	1.15061
H	-3.56310	5.13111	0.48043
H	-1.19973	5.01611	-0.30565
H	-0.03950	2.86556	-0.44891
H	-2.44853	-3.06904	1.34285
H	-4.66376	-4.12198	0.98501
H	-6.25695	-3.14414	-0.65738
H	-5.61163	-1.11329	-1.94328
H	-3.40263	-0.06140	-1.57704
H	0.39012	-1.12261	2.63262
H	2.13209	-1.24655	2.28169
H	0.54876	1.32119	2.22539
H	2.17712	1.10490	1.54771
H	2.91944	1.24246	-2.25993
H	2.57364	2.47743	0.24316

Conformer 2
Energy: -1267.71367 Hartree (Rel: 0.7 kcal/mol)
XYZ coordinates for conf 2:

C	5.41580	-1.75764	0.30933
C	5.82293	-0.42170	0.34309
C	4.93247	0.58854	-0.02618
C	3.62842	0.27638	-0.42353
C	3.21637	-1.07025	-0.44796
C	4.11419	-2.07755	-0.08375
C	2.66773	1.36759	-0.88971
C	1.22593	0.83104	-0.97260
C	0.52511	0.34079	0.32923
C	0.80558	-1.17883	0.34974
C	1.77924	-1.37265	-0.82915
C	-0.99313	0.37567	0.22839
C	-1.43885	-0.90657	0.13445
O	-0.43697	-1.86291	0.12716
C	-1.74034	1.63857	0.09286
C	-2.87387	1.75602	-0.73821
C	-3.54158	2.97064	-0.87874
C	-3.08806	4.11138	-0.20934
C	-1.95132	4.02153	0.59591

C	-1.28575	2.80396	0.74236
C	-2.78971	-1.49310	0.07674
C	-3.01073	-2.65949	-0.67647
C	-4.27523	-3.24622	-0.71821
C	-5.33394	-2.68692	0.00140
C	-5.11889	-1.53752	0.76785
C	-3.85752	-0.94634	0.80925
O	1.30522	-0.38044	-1.75976
O	2.78399	2.59753	-0.18084
C	1.24657	-1.11433	1.83054
C	1.14700	0.44101	1.77234
H	6.10878	-2.54809	0.58394
H	6.83373	-0.16711	0.65000
H	5.24243	1.62943	-0.00438
H	3.79358	-3.11673	-0.10801
H	0.60500	1.54049	-1.52333
H	1.67401	-2.35285	-1.30045
H	-3.22228	0.88918	-1.28943
H	-4.41127	3.02994	-1.52812
H	-3.60706	5.05894	-0.32442
H	-1.57778	4.90135	1.11336
H	-0.40155	2.75807	1.37051
H	-2.18643	-3.09877	-1.22852
H	-4.43240	-4.14296	-1.31138
H	-6.31792	-3.14677	-0.02831
H	-5.93318	-1.10566	1.34320
H	-3.69275	-0.06306	1.41740
H	0.49966	-1.57330	2.48226
H	2.22984	-1.52753	2.06451
H	0.49456	0.89588	2.52110
H	2.12673	0.92248	1.82437
H	2.94048	1.63455	-1.91808
H	2.64530	2.42501	0.76340

Originally assigned correct structure of 17{3e} ($CDCl_3$)

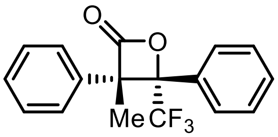

					Conf1
		Rel energy (kcal/mol):			0.00
**C-nom**	**iGau**	**Exp**	**Calc**	**diff**	**1**
C	1	169.70	170.00	0.30	[170.00]
C	6	134.80	135.07	0.27	[135.07]
C	12	130.10	130.83	0.73	[130.83]
C	15	129.80	129.02	-0.78	[129.02]
C	14	128.90	128.23	-0.67	[128.23]
C	16	128.90	128.23	-0.67	[128.23]
C	8	128.80	127.77	-1.03	[127.77]
C	10	128.80	127.77	-1.03	[127.77]
C	9	128.40	127.61	-0.79	[127.61]
C	7	126.80	126.45	-0.35	[126.45]
C	11	126.80	126.45	-0.35	[126.45]
C	13	126.30	126.02	-0.28	[126.02]
C	17	126.30	126.02	-0.28	[126.02]
C	20	123.20	123.51	0.31	[123.51]
C	3	84.70	81.64	-3.06	[81.64]
C	2	67.00	66.73	-0.27	[66.73]
C	19	24.60	24.71	0.11	[24.71]

13C chem shifts: RMSD=0.94ppm (MAE=0.66) N=17 {-3.06 0.73}

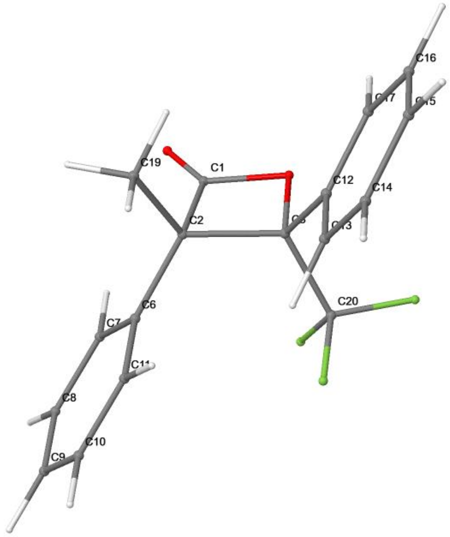

Conformer 1
Energy: -1105.59292 Hartree (Rel: 0.0 kcal/mol)
XYZ coordinates for conf 1:

C	0.45421	2.03783	-0.62115
C	0.54334	0.52703	-0.85093
C	-0.60475	0.50948	0.25907
O	-0.61983	1.97654	0.23405
O	1.00254	3.03049	-1.00601
C	1.87783	-0.15559	-0.59024
C	3.05183	0.58508	-0.39985
C	4.27481	-0.06147	-0.20697
C	4.34204	-1.45518	-0.19873
C	3.17686	-2.20153	-0.39136
C	1.95655	-1.55654	-0.59068
C	-1.95440	-0.08553	-0.07777

C	-2.11521	-1.47501	-0.18940
C	-3.36090	-2.01488	-0.50952
C	-4.45873	-1.17700	-0.71927
C	-4.30344	0.20537	-0.60594
C	-3.05816	0.75053	-0.28676
H	-5.42779	-1.59972	-0.96873
C	0.02975	0.19799	-2.26824
C	-0.15355	0.12979	1.68113
F	0.97733	0.77031	2.03132
F	0.06924	-1.19567	1.79778
F	-1.10098	0.46701	2.57579
H	5.29351	-1.95690	-0.04631
H	3.01597	1.66912	-0.41079
H	5.17481	0.52981	-0.06282
H	3.21620	-3.28726	-0.38895
H	1.06238	-2.15308	-0.74870
H	-1.27570	-2.14013	-0.01734
H	-3.47124	-3.09216	-0.59414
H	-5.15162	0.86536	-0.76402
H	-2.94106	1.82468	-0.19863
H	0.77814	0.52641	-2.99514
H	-0.11716	-0.87876	-2.38541
H	-0.91692	0.69931	-2.48767

Originally assigned correct structure of 18{3f} ($CDCl_3$)

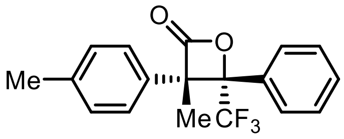

	Conf1
Rel energy (kcal/mol):	0.00

C-nom	iGau	Exp	Calc	diff	1
C	1	169.90	170.14	0.24	[170.14]
C	9	138.30	138.33	0.03	[138.33]
C	6	131.80	132.04	0.24	[132.04]
C	12	130.30	131.00	0.70	[131.00]
C	15	129.70	128.93	-0.77	[128.93]
C	8	129.40	128.56	-0.84	[128.56]
C	10	129.40	128.56	-0.84	[128.56]
C	14	129.00	128.17	-0.83	[128.17]
C	16	129.00	128.17	-0.83	[128.17]
C	7	128.90	126.55	-2.35	[126.55]
C	11	128.90	126.55	-2.35	[126.55]
C	13	126.20	126.05	-0.15	[126.05]
C	17	126.20	126.05	-0.15	[126.05]
C	20	123.30	123.50	0.20	[123.50]
C	3	84.80	81.62	-3.18	[81.62]
C	2	66.80	66.64	-0.16	[66.64]
C	19	24.70	24.61	-0.09	[24.61]
C	24	21.00	20.17	-0.83	[20.17]

13C chem shifts: RMSD=1.20ppm (MAE=0.82) N=18 {-3.18 0.70}

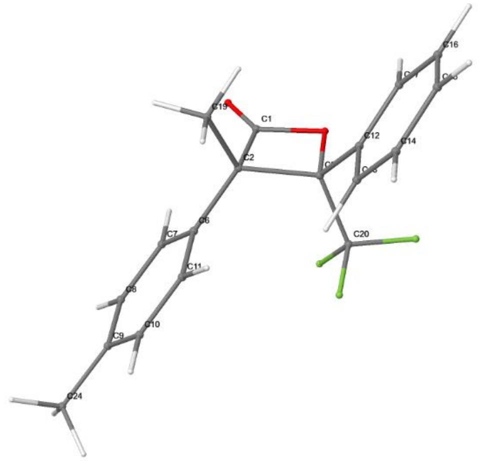

Conformer 1
Energy: -1144.91055 Hartree (Rel: 0.0 kcal/mol)
XYZ coordinates for conf 1:

C	-0.06860	2.15105	-0.61829
C	0.18647	0.66014	-0.85279
C	-0.95026	0.51307	0.26016
O	-1.12316	1.97004	0.24435
O	0.36370	3.19926	-1.00393
C	1.58987	0.13384	-0.59478
C	2.67271	0.99660	-0.39020
C	3.95990	0.49058	-0.19731
C	4.21011	-0.88605	-0.20076
C	3.12093	-1.74617	-0.40676
C	1.83624	-1.24764	-0.60633
C	-2.22971	-0.22056	-0.07790
C	-2.23923	-1.61738	-0.21091
C	-3.42157	-2.28495	-0.53055

C	-4.60608	-1.56877	-0.71841
C	-4.60134	-0.17963	-0.58354
C	-3.42004	0.49307	-0.26467
H	-5.52553	-2.09079	-0.96771
C	-0.29130	0.27783	-2.26906
C	-0.45872	0.17245	1.67846
F	0.59489	0.92929	2.03631
F	-0.09253	-1.12197	1.78160
F	-1.43693	0.39466	2.57654
C	5.60521	-1.43356	-0.00978
H	2.51871	2.07042	-0.38662
H	4.78263	1.18392	-0.03989
H	3.28044	-2.82199	-0.41173
H	1.02070	-1.94660	-0.76984
H	-1.33043	-2.18952	-0.05738
H	-3.41462	-3.36639	-0.63231
H	-5.51779	0.38629	-0.72455
H	-3.41989	1.57210	-0.15981
H	0.41505	0.68647	-2.99735
H	-0.32033	-0.80860	-2.38674
H	-1.28772	0.67274	-2.48579
H	6.02433	-1.79101	-0.95952
H	5.61010	-2.28269	0.68309
H	6.28374	-0.67005	0.38327

Original correct structure of 19{3l} (xray) to test DU8ML performance ($CDCl_3$)

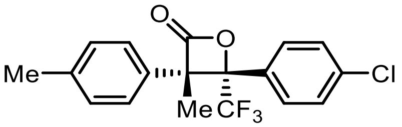


```
                                    Conf1
              Rel energy (kcal/mol):   0.00

 C-nom     iGau   Exp    Calc   diff        1
 C           1  169.50 169.65   0.15   [ 169.65 ]
 C           9  138.50 138.53   0.03   [ 138.53 ]
 C          15  136.20 135.96  -0.24   [ 135.96 ]
 C           6  131.50 131.67   0.17   [ 131.67 ]
 C          12  129.60 130.69   1.09   [ 130.69 ]
 C           8  129.30 128.64  -0.66   [ 128.64 ]
 C          10  129.30 128.64  -0.66   [ 128.64 ]
 C          14  129.00 128.24  -0.76   [ 128.24 ]
 C          16  129.00 128.24  -0.76   [ 128.24 ]
 C          13  126.70 127.67   0.97   [ 127.67 ]
 C          17  126.70 127.67   0.97   [ 127.67 ]
 C           7  126.20 126.45   0.25   [ 126.45 ]
 C          11  126.20 126.45   0.25   [ 126.45 ]
 C          20  123.10 123.22   0.12   [ 123.22 ]
 C           3   84.40  81.15  -3.25   [  81.15 ]
 C           2   67.10  66.97  -0.13   [  66.97 ]
 C          19   24.70  24.47  -0.23   [  24.47 ]
 C          24   21.10  20.11  -0.99   [  20.11 ]
        13C chem shifts: RMSD=0.97ppm (MAE=0.65) N=18 {-3.25 1.09}
```

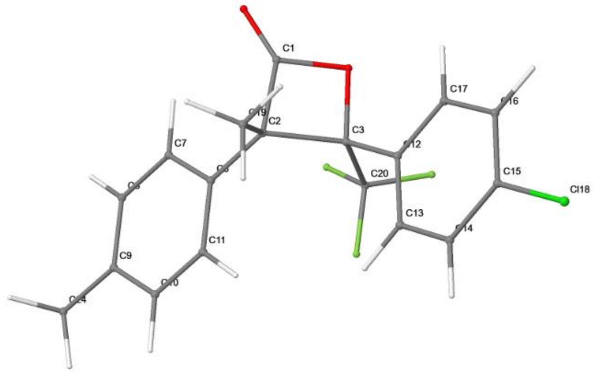

```
Conformer 1
Energy: -1604.50249 Hartree (Rel:  0.0 kcal/mol)
XYZ coordinates for conf 1:
C          0.78174        2.14501       -0.91411
C          0.79590        0.61403       -0.91418
C         -0.27286        0.82193        0.25563
O         -0.22571        2.26669        0.01487
O          1.33597        3.04030       -1.48346
C          2.12003       -0.07925       -0.63149
C          3.33044        0.62749       -0.61417
C          4.53782       -0.03501       -0.39731
C          4.58278       -1.42020       -0.18848
C          3.37024       -2.12104       -0.20705
C          2.15987       -1.46529       -0.43035
C         -1.66942        0.26336        0.09255
C         -1.90808       -1.11525        0.18955
C         -3.19444       -1.62804        0.03159
C         -4.24893       -0.75162       -0.22283
C         -4.03693        0.62202       -0.31937
C         -2.74464        1.12323       -0.16043
Cl        -5.87418       -1.39310       -0.42542
```

C	0.17574	0.09635	-2.22854
C	0.25196	0.62113	1.68883
F	1.42959	1.24173	1.87918
F	0.41952	-0.68651	1.97647
F	-0.62054	1.12871	2.57947
C	5.89792	-2.12994	0.03343
H	3.33526	1.69978	-0.77849
H	5.46186	0.53858	-0.38970
H	3.37034	-3.19636	-0.04609
H	1.24288	-2.04775	-0.44523
H	-1.09587	-1.80271	0.39839
H	-3.37306	-2.69505	0.10568
H	-4.86609	1.29338	-0.51399
H	-2.57442	2.19107	-0.23363
H	0.88532	0.27442	-3.04157
H	-0.01688	-0.97812	-2.16823
H	-0.76441	0.60149	-2.46690
H	6.49515	-1.63122	0.80563
H	5.74458	-3.16869	0.34187
H	6.50237	-2.14067	-0.88266

Testing the (incorrect) *cis*-structure for 19{3l} ($CDCl_3$)

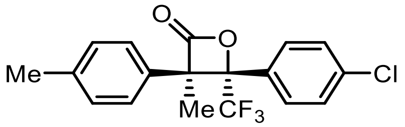

					Conf1
		Rel energy (kcal/mol):			0.00
**C-nom**	**iGau**	**Exp**	**Calc**	**diff**	**1**
C	1	169.50	170.08	0.58	[170.08]
C	9	138.50	138.01	-0.49	[138.01]
C	15	136.20	135.11	-1.09	[135.11]
C	6	131.50	132.59	1.09	[132.59]
C	12	129.60	131.83	2.23	[131.83]
C	8	129.30	128.60	-0.70	[128.60]
C	10	129.30	128.60	-0.70	[128.60]
C	14	129.00	127.55	-1.45	[127.55]
C	16	129.00	127.55	-1.45	[127.55]
C	13	126.70	127.22	0.52	[127.22]
C	17	126.70	127.22	0.52	[127.22]
C	7	126.20	126.09	-0.11	[126.09]
C	11	126.20	126.09	-0.11	[126.09]
C	20	123.10	124.34	1.24	[124.34]
C	3	84.40	81.69	-2.71	[81.69]
C	2	67.10	70.86	3.76	[70.86]
C	19	24.70	21.37	-3.33	[21.37]
C	24	21.10	19.85	-1.25	[19.85]

13C chem shifts: RMSD=1.66ppm (MAE=1.30) N=18 {-3.33 3.76}

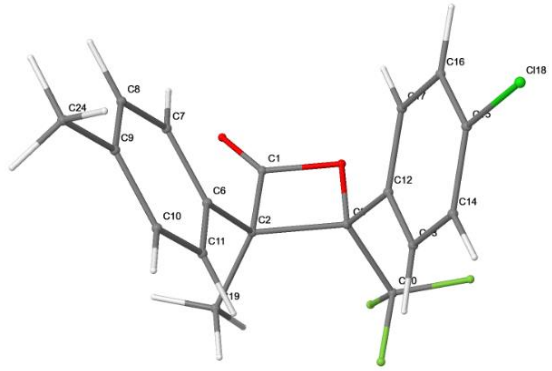

Conformer 1
Energy: -1604.50030 Hartree (Rel: 0.0 kcal/mol)
XYZ coordinates for conf 1:

C	2.25482	0.82465	1.39586
C	1.68457	0.95100	-0.01898
C	1.45197	-0.62755	0.17608
O	1.99516	-0.51851	1.53191
O	2.78538	1.53245	2.20244
C	0.41461	1.78897	-0.12008
C	-0.05352	2.56055	0.94849
C	-1.20400	3.34309	0.81394
C	-1.92269	3.38081	-0.38445
C	-1.44557	2.60842	-1.45553
C	-0.29888	1.83045	-1.32923
C	0.03321	-1.16014	0.18690
C	-0.60821	-1.55035	-0.99804
C	-1.91891	-2.02241	-0.97790
C	-2.59428	-2.10402	0.23915
C	-1.97854	-1.72504	1.42931
C	-0.66569	-1.25396	1.39572
Cl	-4.24970	-2.69961	0.27043
C	2.73187	1.43686	-1.03957

C	2.40436	-1.55011	-0.61145
F	3.69597	-1.29934	-0.32566
F	2.24071	-1.38383	-1.94500
F	2.18049	-2.84495	-0.32987
C	-3.17058	4.21936	-0.53132
H	0.48282	2.57303	1.89175
H	-1.54240	3.93524	1.66068
H	-1.97973	2.61776	-2.40294
H	0.03880	1.24812	-2.18183
H	-0.09115	-1.49816	-1.94911
H	-2.40690	-2.32403	-1.89819
H	-2.51107	-1.79758	2.37125
H	-0.18326	-0.96825	2.32286
H	2.92070	2.49754	-0.85080
H	2.35643	1.33464	-2.06043
H	3.67737	0.89957	-0.96003
H	-3.08137	4.92411	-1.36698
H	-4.04846	3.59290	-0.73301
H	-3.37290	4.79663	0.37599

Originally assigned (incorrect) structure 20{3a} ($CDCl_3$)

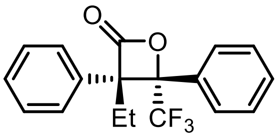

	Conf1	Conf2	Conf3
Rel energy (kcal/mol):	0.00	2.36	2.78

C-nom	iGau	Exp	Calc	diff	1	2	3
C	1	169.60	169.64	0.04	[169.64	169.16	170.76]
C	6	132.80	133.52	0.72	[133.46	136.90	132.85]
C	12	131.40	130.94	-0.46	[130.95	130.48	130.43]
C	15	129.70	128.94	-0.76	[128.94	129.01	129.15]
C	14	128.70	128.18	-0.52	[128.18	128.07	128.06]
C	16	128.70	128.18	-0.52	[128.18	128.07	128.06]
C	8	128.40	127.76	-0.64	[127.76	127.82	127.32]
C	10	128.40	127.76	-0.64	[127.76	127.82	127.32]
C	9	127.90	127.62	-0.28	[127.63	127.36	127.26]
C	7	127.50	126.96	-0.54	[126.98	125.82	127.08]
C	11	127.50	126.96	-0.54	[126.98	125.82	127.08]
C	13	126.10	126.10	0.00	[126.08	126.89	126.85]
C	17	126.10	126.10	0.00	[126.08	126.89	126.85]
C	20	124.10	123.48	-0.62	[123.48	123.32	123.93]
C	3	84.80	81.71	-3.09	[81.72	80.80	82.55]
C	2	75.10	70.20	-4.90	[70.19	70.82	70.03]
C	19	24.80	32.70	7.90	[32.73	32.39	30.54]
C	18	9.80	7.83	-1.97	[7.80	9.45	7.62]

13C chem shifts: RMSD=2.40ppm (MAE=1.34) N=18 {-4.90 7.90}

Fractions: 0.973 0.018 0.009

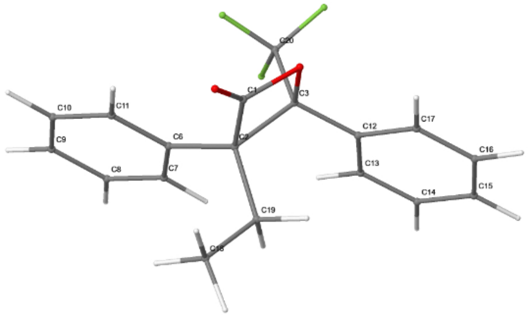

Conformer 1
Energy: -1144.90531 Hartree (Rel: 0.0 kcal/mol)
XYZ coordinates for conf 1:

C	0.44532	1.56484	1.32660
C	0.53042	0.83652	-0.01673
C	-0.66447	-0.01590	0.61975
O	-0.66778	0.89232	1.77125
O	1.01942	2.44408	1.90430
C	1.84247	0.15494	-0.37394
C	1.90602	-0.69450	-1.48914
C	3.10563	-1.30618	-1.85215
C	4.26610	-1.07143	-1.11054
C	4.21494	-0.22077	-0.00569
C	3.01248	0.38770	0.36137
C	-2.00775	-0.06507	-0.07612
C	-2.17895	-0.81307	-1.25089
C	-3.41998	-0.84916	-1.88683
C	-4.50329	-0.14367	-1.35791
C	-4.33813	0.59829	-0.18737
C	-3.09747	0.63863	0.45189
C	1.05390	2.89585	-1.48090
C	0.05086	1.78476	-1.15239

C -0.27389 -1.39305 1.18808
F 0.84541 -1.32580 1.93260
F -0.06535 -2.29750 0.20908
F -1.25537 -1.86458 1.97975
H 1.01590 -0.88284 -2.08262
H 3.13247 -1.96511 -2.71559
H 5.20138 -1.54582 -1.39422
H 5.11158 -0.02644 0.57629
H 2.98946 1.05238 1.21820
H -1.35143 -1.37661 -1.66824
H -3.53804 -1.43082 -2.79658
H -5.46877 -0.17335 -1.85488
H -5.17506 1.14781 0.23406
H -2.97290 1.21526 1.36142
H 0.63936 3.54750 -2.25723
H 1.26879 3.51139 -0.60148
H 2.00018 2.48902 -1.84986
H -0.14462 1.17703 -2.04291
H -0.90891 2.22805 -0.86603

Conformer 2
Energy: -1144.90089 Hartree (Rel: 2.4 kcal/mol)
XYZ coordinates for conf 2:
C 0.50057 1.93655 0.68771
C 0.59127 0.82045 -0.35655
C -0.55464 0.17785 0.55135
O -0.58204 1.40926 1.34933
O 1.05621 2.96664 0.94368
C 1.92859 0.10206 -0.49727
C 2.01519 -1.07742 -1.25210
C 3.24046 -1.71787 -1.43716
C 4.40350 -1.18423 -0.87738
C 4.32896 -0.00551 -0.13422
C 3.10180 0.63383 0.05509
C -1.90875 -0.17515 -0.02310
C -2.03392 -1.18027 -0.99207
C -3.29103 -1.52358 -1.49162
C -4.43478 -0.87426 -1.02274
C -4.31578 0.11798 -0.04731
C -3.06037 0.46453 0.45416
C -0.92924 2.43461 -1.81242
C 0.13721 1.33297 -1.75976
C -0.09011 -0.92487 1.52513
F 1.04815 -0.59089 2.16060
F 0.11578 -2.09433 0.88656
F -1.02828 -1.13213 2.46888
H 1.12443 -1.50350 -1.70404
H 3.28429 -2.63365 -2.02016
H 5.35833 -1.68188 -1.02188
H 5.22694 0.42253 0.30273
H 3.06163 1.55333 0.62885
H -1.15673 -1.70747 -1.35251
H -3.37358 -2.30052 -2.24617
H -5.41248 -1.14186 -1.41324
H -5.20070 0.62384 0.32814
H -2.96945 1.23622 1.21051
H -1.10846 2.70578 -2.85837
H -1.88315 2.12167 -1.38222
H -0.59561 3.33839 -1.29140
H 1.04584 1.70520 -2.24449
H -0.18916 0.46495 -2.34274

Conformer 3
Energy: -1144.90155 Hartree (Rel: 2.8 kcal/mol)
XYZ coordinates for conf 3:
C 0.47934 1.59617 -1.37031
C 0.56570 0.14769 -0.88117
C -0.61077 0.64212 0.09232
O -0.61134 1.93197 -0.61148
O 1.03773 2.30035 -2.16299
C 1.90797 -0.30168 -0.32069

C	2.00263	-1.37568	0.57743
C	3.24519	-1.81305	1.03800
C	4.41677	-1.19057	0.60269
C	4.33408	-0.12884	-0.29912
C	3.09126	0.31197	-0.75826
C	-1.97717	-0.01503	0.04601
C	-2.23539	-1.21269	0.73182
C	-3.49931	-1.80032	0.67394
C	-4.52272	-1.20255	-0.06489
C	-4.27357	-0.00978	-0.74426
C	-3.01008	0.58223	-0.68893
C	0.03521	-2.26489	-1.82657
C	0.09475	-0.75373	-2.06655
C	-0.19590	1.00213	1.53383
F	0.95499	1.69874	1.55727
F	-0.03144	-0.08775	2.31322
F	-1.14394	1.76850	2.10407
H	1.10646	-1.87725	0.92528
H	3.29436	-2.64309	1.73738
H	5.38376	-1.53151	0.96189
H	5.23761	0.36343	-0.64825
H	3.04229	1.13714	-1.46048
H	-1.45994	-1.68884	1.32086
H	-3.68169	-2.72702	1.21042
H	-5.50596	-1.66228	-0.10763
H	-5.06254	0.46810	-1.31797
H	-2.82666	1.51386	-1.21141
H	-0.30938	-2.75533	-2.74357
H	1.01718	-2.67522	-1.57636
H	-0.66442	-2.52942	-1.02855
H	-0.88504	-0.39890	-2.40599
H	0.79825	-0.54869	-2.88253

Revised structure of 20{3a}, i.e. 25{3a-rev} ($CDCl_3$)

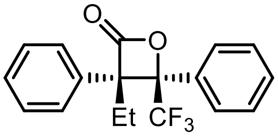

	Conf1	Conf2
Rel energy (kcal/mol):	0.00	3.63

C-nom	iGau	Exp	Calc	diff	1	2
C	1	169.60	170.23	0.63	[170.23	169.12]
C	6	132.80	134.40	1.60	[134.39	139.91]
C	12	131.40	132.07	0.67	[132.07	131.74]
C	15	129.70	128.22	-1.48	[128.22	128.21]
C	8	128.70	127.62	-1.08	[127.62	127.54]
C	10	128.70	127.62	-1.08	[127.62	127.54]
C	14	128.40	127.52	-0.88	[127.52	127.37]
C	16	128.40	127.52	-0.88	[127.52	127.37]
C	9	127.90	126.91	-0.99	[126.91	126.76]
C	7	127.50	126.82	-0.68	[126.82	126.85]
C	11	127.50	126.82	-0.68	[126.82	126.85]
C	13	126.10	125.60	-0.50	[125.60	125.94]
C	17	126.10	125.60	-0.50	[125.60	125.94]
C	20	124.10	124.61	0.51	[124.61	124.65]
C	3	84.80	82.47	-2.33	[82.47	82.25]
C	2	75.10	74.81	-0.29	[74.81	73.97]
C	19	24.80	28.45	3.65	[28.44	31.19]
C	18	9.80	8.96	-0.84	[8.95	11.27]

13C chem shifts: RMSD=1.33ppm (MAE=1.07) N=18 {-2.33 3.65}

Fractions: 0.998 0.002

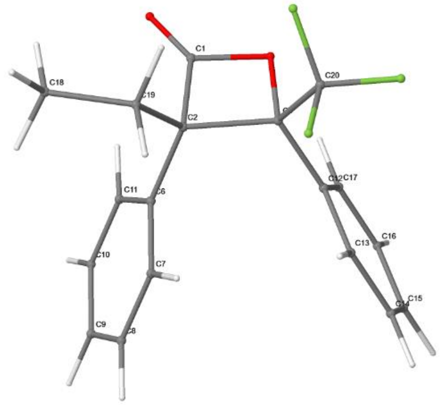

Conformer 1
Energy: -1144.90244 Hartree (Rel: 0.0 kcal/mol)
XYZ coordinates for conf 1:

C	0.22692	-1.60985	1.46925
C	-0.13116	-1.22027	0.03257
C	1.07349	-0.17491	0.25750
O	1.22443	-0.67994	1.62492
O	-0.12173	-2.41703	2.28399
C	-1.50799	-0.58426	-0.13174
C	-1.85935	-0.00495	-1.36144
C	-3.11903	0.56215	-1.54826
C	-4.05439	0.55595	-0.50979
C	-3.71767	-0.02314	0.71315
C	-2.45375	-0.58973	0.90145
C	0.76326	1.30944	0.24192
C	0.79627	2.04547	-0.95255
C	0.49297	3.40643	-0.95025
C	0.15276	4.05174	0.24081
C	0.12065	3.32486	1.43110
C	0.42358	1.96176	1.43323

```
C         -0.98168       -3.49034       -0.83005
C          0.07030       -2.38316       -0.97690
C          2.39610       -0.48690       -0.47398
F          2.88003       -1.70154       -0.14814
F          2.22432       -0.46310       -1.81667
F          3.34964        0.40970       -0.16819
H         -1.14568        0.00917       -2.18001
H         -3.36960        1.00886       -2.50648
H         -5.03670        0.99633       -0.65604
H         -4.43730       -0.04036        1.52694
H         -2.21691       -1.05045        1.85475
H          1.06476        1.56489       -1.88650
H          0.52520        3.96198       -1.88321
H         -0.08220        5.11236        0.24012
H         -0.13731        3.81599        2.36511
H          0.40675        1.40761        2.36443
H         -0.75967       -4.30142       -1.53170
H         -0.97849       -3.90719        0.18199
H         -1.98963       -3.12310       -1.04398
H          0.03846       -1.96885       -1.98999
H          1.06306       -2.81735       -0.84576

Conformer 2
Energy: -1144.89666 Hartree (Rel:  3.6 kcal/mol)
XYZ coordinates for conf 2:
C          1.17781       -1.14236        1.40192
C          0.66231       -1.05496       -0.03960
C          0.84538        0.52440        0.23887
O          1.27407        0.21124        1.60427
O          1.44139       -1.99546        2.20041
C         -0.79783       -1.48700       -0.17739
C         -1.43821       -1.35436       -1.42091
C         -2.76516       -1.74592       -1.58724
C         -3.47866       -2.28572       -0.51327
C         -2.85126       -2.42811        0.72333
C         -1.52146       -2.02993        0.89161
C         -0.37327        1.42661        0.22975
C         -0.89388        1.93043       -0.97232
C         -2.01806        2.75544       -0.96166
C         -2.63756        3.08903        0.24485
C         -2.12321        2.59131        1.44235
C         -0.99800        1.76450        1.43641
C          2.76741       -2.53911       -0.59773
C          1.55723       -1.75455       -1.12375
C          2.05335        1.20720       -0.42922
F          3.19408        0.51881       -0.22843
F          1.88420        1.32509       -1.76731
F          2.24063        2.44109        0.06868
H         -0.89652       -0.94297       -2.26803
H         -3.24102       -1.63119       -2.55724
H         -4.51182       -2.59544       -0.64313
H         -3.39095       -2.85353        1.56484
H         -1.05056       -2.16643        1.85926
H         -0.42452        1.68794       -1.91860
H         -2.40876        3.13735       -1.90061
H         -3.51300        3.73228        0.25008
H         -2.59387        2.84619        2.38769
H         -0.59971        1.38765        2.37118
H          3.28811       -3.00270       -1.44258
H          3.48252       -1.89712       -0.07657
H          2.46085       -3.33342        0.08870
H          0.91504       -2.44828       -1.67128
H          1.88881       -1.01275       -1.85364
```

Original (incorrect) structure of 21{3b} ($CDCl_3$)

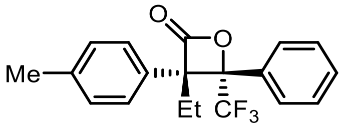

					Conf1	Conf2	Conf3
Rel energy (kcal/mol):					0.00	2.39	2.77
**C-nom**	**iGau**	**Exp**	**Calc**	**diff**	**1**	**2**	**3**
C	1	169.80	169.78	-0.02	[169.78	169.29	170.87]
C	9	137.70	138.28	0.58	[138.29	137.96	137.92]
C	12	131.50	131.05	-0.45	[131.06	130.55	130.56]
C	6	129.70	130.51	0.81	[130.45	134.00	129.84]
C	8	129.00	128.52	-0.48	[128.52	128.55	128.07]
C	10	129.00	128.52	-0.48	[128.52	128.55	128.07]
C	15	128.60	128.88	0.28	[128.88	128.96	129.09]
C	14	127.80	128.14	0.34	[128.14	128.05	128.01]
C	16	127.80	128.14	0.34	[128.14	128.05	128.01]
C	7	127.30	127.03	-0.27	[127.05	125.89	127.19]
C	11	127.30	127.03	-0.27	[127.05	125.89	127.19]
C	13	126.10	126.11	0.01	[126.09	126.87	126.83]
C	17	126.10	126.11	0.01	[126.09	126.87	126.83]
C	20	124.20	123.51	-0.69	[123.51	123.34	123.99]
C	3	84.80	81.69	-3.11	[81.70	80.76	82.51]
C	2	74.90	70.01	-4.89	[70.00	70.61	69.83]
C	19	24.70	32.63	7.93	[32.65	32.39	30.53]
C	24	20.70	20.26	-0.44	[20.26	20.20	20.21]
C	18	9.70	7.84	-1.86	[7.81	9.44	7.64]

13C chem shifts: RMSD=2.32ppm (MAE=1.22) N=19 {-4.89 7.93}
Fractions: 0.974 0.017 0.009

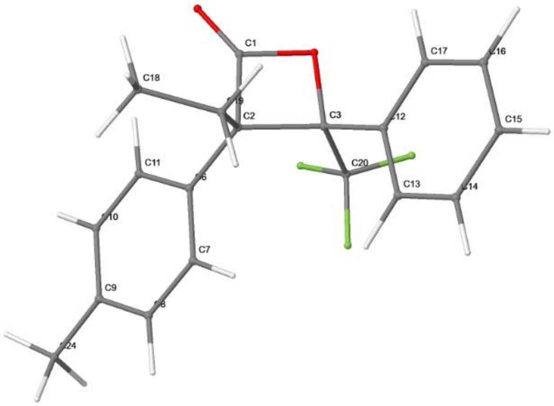

Conformer 1
Energy: -1184.22294 Hartree (Rel: 0.0 kcal/mol)
XYZ coordinates for conf 1:

C	-0.07662	1.99773	0.84229
C	0.17376	0.92009	-0.21500
C	-0.98139	0.16747	0.59691
O	-1.15124	1.36727	1.42292
O	0.36899	3.06641	1.15198
C	1.56544	0.31306	-0.29159
C	1.79879	-0.81225	-1.09621
C	3.07414	-1.36230	-1.20413
C	4.16493	-0.80762	-0.51910
C	3.92931	0.32291	0.27205
C	2.65280	0.87541	0.38832
C	-2.26037	-0.22958	-0.10818
C	-2.27356	-1.29709	-1.01867
C	-3.45645	-1.65476	-1.66573
C	-4.63820	-0.95516	-1.41038

C	-4.63005	0.10482	-0.50241
C	-3.44815	0.46723	0.14670
C	0.61318	2.49905	-2.20760
C	-0.30673	1.43331	-1.60220
C	-0.50961	-0.93266	1.56579
F	0.53991	-0.53015	2.30587
F	-0.14736	-2.05447	0.90967
F	-1.50010	-1.25829	2.41793
C	5.54095	-1.42329	-0.61767
H	0.98222	-1.26704	-1.65011
H	3.22473	-2.23618	-1.83376
H	4.75613	0.78390	0.80721
H	2.51000	1.75739	1.00362
H	-1.36712	-1.85814	-1.21932
H	-3.45208	-2.48195	-2.36978
H	-5.55814	-1.23544	-1.91557
H	-5.54436	0.65329	-0.29439
H	-3.44534	1.29057	0.85199
H	0.19864	2.84470	-3.16047
H	0.70846	3.36572	-1.54553
H	1.61617	2.10517	-2.39709
H	-0.38225	0.57260	-2.27611
H	-1.32067	1.83569	-1.50294
H	5.65959	-2.24282	0.10364
H	5.72255	-1.84095	-1.61387
H	6.32426	-0.68795	-0.40718

Conformer 2
Energy: -1184.21852 Hartree (Rel: 2.4 kcal/mol)
XYZ coordinates for conf 2:

C	-0.01333	2.13314	0.40428
C	0.24014	0.88179	-0.44049
C	-0.88079	0.27354	0.52075
O	-1.07100	1.60601	1.10582
O	0.42411	3.24366	0.50838
C	1.64671	0.29669	-0.41542
C	1.89529	-0.96849	-0.96732
C	3.18588	-1.49243	-1.00381
C	4.27684	-0.77226	-0.49711
C	4.02541	0.49633	0.03900
C	2.73441	1.02500	0.08233
C	-2.16165	-0.30882	-0.03433
C	-2.13534	-1.46229	-0.83038
C	-3.32436	-2.01331	-1.31039
C	-4.55010	-1.42447	-0.99331
C	-4.58153	-0.28325	-0.18906
C	-3.39461	0.27110	0.29218
C	-1.36008	2.07314	-2.18968
C	-0.19230	1.11457	-1.92261
C	-0.35879	-0.60530	1.67618
F	0.70456	-0.05468	2.28998
F	-0.00266	-1.83155	1.24334
F	-1.31963	-0.76221	2.60719
C	5.67025	-1.35582	-0.51174
H	1.07999	-1.55498	-1.38091
H	3.34738	-2.47723	-1.43607
H	4.85149	1.08579	0.42994
H	2.58014	2.01549	0.49689
H	-1.19212	-1.94247	-1.06955
H	-3.28969	-2.90433	-1.93064
H	-5.47467	-1.85397	-1.36843
H	-5.53085	0.17777	0.06823
H	-3.42048	1.15830	0.91518
H	-1.51258	2.15518	-3.27123
H	-2.29834	1.73170	-1.74685
H	-1.14569	3.07836	-1.81119
H	0.69779	1.50102	-2.43001
H	-0.39942	0.13542	-2.36802
H	5.84330	-1.98760	0.36969
H	5.83313	-1.98240	-1.39532
H	6.43297	-0.57042	-0.50431

Conformer 3
Energy: -1184.21914 Hartree (Rel: 2.8 kcal/mol)
XYZ coordinates for conf 3:

C	-0.01582	2.03707	-0.85465
C	0.21630	0.52376	-0.83235
C	-0.93892	0.58900	0.28005
O	-1.08475	2.02453	0.00344
O	0.43639	2.99686	-1.41185
C	1.62002	0.05836	-0.47361
C	1.86168	-1.22204	0.04598
C	3.16063	-1.65230	0.31301
C	4.26648	-0.82730	0.06748
C	4.02169	0.44391	-0.46592
C	2.72429	0.88257	-0.73250
C	-2.24758	-0.15072	0.07636
C	-2.36724	-1.51788	0.37282
C	-3.57970	-2.17911	0.17570
C	-4.68902	-1.48787	-0.31641
C	-4.57766	-0.12806	-0.60789
C	-3.36602	0.53795	-0.41201
C	-0.16740	-1.53181	-2.45111
C	-0.24748	-0.02009	-2.22101
C	-0.47315	0.53518	1.74964
F	0.61282	1.30289	1.95322
F	-0.17092	-0.71760	2.15166
F	-1.44885	0.99455	2.55530
C	5.66965	-1.28642	0.38765
H	1.03654	-1.89706	0.24546
H	3.31543	-2.65000	0.71730
H	4.85785	1.10636	-0.67757
H	2.57304	1.87268	-1.14904
H	-1.52282	-2.07199	0.76648
H	-3.65454	-3.23712	0.41002
H	-5.63192	-2.00535	-0.46842
H	-5.43446	0.42230	-0.98602
H	-3.28962	1.59645	-0.63181
H	-0.52215	-1.75530	-3.46326
H	0.85814	-1.90101	-2.36655
H	-0.79533	-2.09017	-1.75096
H	-1.27132	0.32415	-2.40667
H	0.38831	0.48747	-2.95654
H	5.93449	-1.05246	1.42746
H	5.77526	-2.36937	0.26262
H	6.40789	-0.79440	-0.25415

Revised structure of 21{3b}, i.e. *cis* 26{3b-rev} ($CDCl_3$)

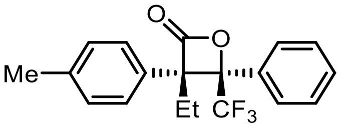

					Conf1	Conf2
Rel energy (kcal/mol):					0.00	3.61

C-nom	iGau	Exp	Calc	diff	1	2
C	1	169.80	170.40	0.60	[170.40	169.23]
C	9	137.70	137.57	-0.13	[137.57	137.41]
C	12	131.50	132.19	0.69	[132.19	131.79]
C	6	129.70	131.43	1.73	[131.42	136.89]
C	8	129.00	128.44	-0.56	[128.44	128.36]
C	10	129.00	128.44	-0.56	[128.44	128.36]
C	15	128.60	128.12	-0.48	[128.12	128.14]
C	14	127.80	127.48	-0.32	[127.48	127.29]
C	16	127.80	127.48	-0.32	[127.48	127.29]
C	7	127.30	126.84	-0.46	[126.84	126.95]
C	11	127.30	126.84	-0.46	[126.84	126.95]
C	13	126.10	125.61	-0.49	[125.61	126.02]
C	17	126.10	125.61	-0.49	[125.61	126.02]
C	20	124.20	124.63	0.43	[124.63	124.65]
C	3	84.80	82.44	-2.36	[82.44	82.28]
C	2	74.90	74.63	-0.27	[74.63	73.68]
C	19	24.70	28.12	3.42	[28.11	31.03]
C	24	20.70	19.90	-0.80	[19.90	19.80]
C	18	9.70	8.97	-0.73	[8.96	11.31]

13C chem shifts: RMSD=1.14ppm (MAE=0.81) N=19 {-2.36 3.42}

Fractions: 0.998 0.002

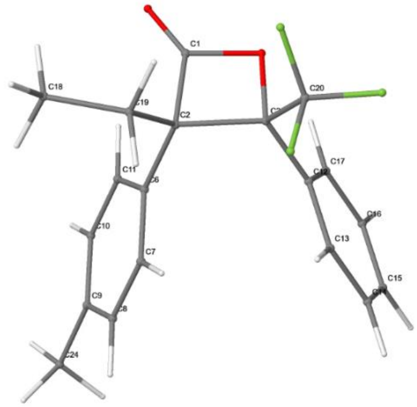

Conformer 1
Energy: -1184.22007 Hartree (Rel: 0.0 kcal/mol)
XYZ coordinates for conf 1:

C	0.87357	-1.54006	1.46572
C	0.37811	-1.23191	0.05018
C	1.36540	0.02943	0.22536
O	1.67933	-0.43447	1.57935
O	0.72739	-2.39648	2.29162
C	-1.10174	-0.87779	-0.04345
C	-1.63113	-0.40858	-1.25660
C	-2.98309	-0.09930	-1.37455
C	-3.86129	-0.24813	-0.28970
C	-3.33190	-0.72565	0.91312
C	-1.97500	-1.03576	1.03816
C	0.76995	1.42399	0.23716
C	0.58225	2.14264	-0.95364
C	0.02200	3.41914	-0.92538
C	-0.35878	3.99616	0.28829

C	-0.17267	3.28643	1.47479
C	0.38805	2.00781	1.45095
C	-0.04926	-3.62678	-0.78774
C	0.75808	-2.33551	-0.97414
C	2.68559	-0.01608	-0.57207
F	3.41584	-1.11038	-0.28063
F	2.44588	-0.02823	-1.90445
F	3.45760	1.05185	-0.30604
C	-5.32304	0.11000	-0.42051
H	-0.98499	-0.28195	-2.12064
H	-3.36436	0.26267	-2.32673
H	-3.98708	-0.86389	1.76985
H	-1.60901	-1.42092	1.98433
H	0.87801	1.71566	-1.90532
H	-0.11452	3.96289	-1.85589
H	-0.79404	4.99132	0.30773
H	-0.45998	3.72589	2.42585
H	0.53880	1.46800	2.37852
H	0.29503	-4.38057	-1.50388
H	0.08077	-4.03266	0.22042
H	-1.11809	-3.46386	-0.95384
H	0.60107	-1.93862	-1.98249
H	1.82150	-2.56671	-0.89001
H	-5.91065	-0.29979	0.40695
H	-5.46526	1.19867	-0.41955
H	-5.74550	-0.26803	-1.35848

Conformer 2
Energy: -1184.21188 Hartree (Rel: 3.6 kcal/mol)
XYZ coordinates for conf 2:

C	1.01414	-1.44024	1.40959
C	0.49999	-1.19217	-0.01376
C	1.25602	0.21628	0.21074
O	1.60572	-0.21218	1.56714
O	0.98571	-2.31858	2.22355
C	-1.02133	-1.06777	-0.08241
C	-1.63576	-0.74823	-1.30563
C	-3.01826	-0.63007	-1.40426
C	-3.84712	-0.83279	-0.28906
C	-3.23341	-1.15614	0.92403
C	-1.84387	-1.27064	1.03028
C	0.44912	1.49981	0.22161
C	0.06898	2.12683	-0.97498
C	-0.67193	3.30787	-0.94639
C	-1.04360	3.87867	0.27299
C	-0.66819	3.25879	1.46512
C	0.07373	2.07609	1.44116
C	1.88980	-3.35237	-0.60049
C	1.03387	-2.18346	-1.10823
C	2.59600	0.40561	-0.52364
F	3.41802	-0.64810	-0.35057
F	2.41693	0.56281	-1.85626
F	3.24198	1.49233	-0.06767
C	-5.34845	-0.71590	-0.40869
H	-1.02886	-0.58878	-2.19272
H	-3.46286	-0.37695	-2.36415
H	-3.84610	-1.32597	1.80611
H	-1.41471	-1.53882	1.98997
H	0.35177	1.70233	-1.93129
H	-0.95833	3.78118	-1.88132
H	-1.62032	4.79913	0.29224
H	-0.94871	3.69430	2.42002
H	0.36795	1.60370	2.37106
H	2.17002	-3.98622	-1.44877
H	2.81086	-3.01232	-0.11939
H	1.33984	-3.96882	0.11627
H	0.16163	-2.59826	-1.61866
H	1.58805	-1.62508	-1.86586
H	-5.83458	-0.80282	0.56784
H	-5.63979	0.24603	-0.84731
H	-5.75836	-1.50083	-1.05698

Original (incorrect) structure of 22{3g} ($CDCl_3$)

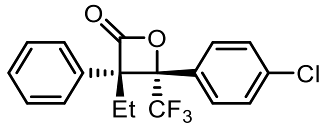

					Conf1	Conf2	Conf3
	Rel energy (kcal/mol):				0.00	2.42	2.81
**C-nom**	**iGau**	**Exp**	**Calc**	**diff**	**1**	**2**	**3**
C	1	169.10	169.19	0.09	[169.19	168.69	170.26]
C	15	134.90	135.92	1.02	[135.92	136.05	136.19]
C	6	132.50	133.02	0.52	[132.97	136.43	132.35]
C	12	130.10	130.63	0.53	[130.64	130.09	130.11]
C	8	128.60	127.84	-0.76	[127.84	127.89	127.39]
C	10	128.60	127.84	-0.76	[127.84	127.89	127.39]
C	14	128.20	128.25	0.05	[128.25	128.17	128.08]
C	16	128.20	128.25	0.05	[128.25	128.17	128.08]
C	9	128.10	127.83	-0.27	[127.84	127.56	127.47]
C	13	127.50	127.70	0.20	[127.68	128.41	128.48]
C	17	127.50	127.70	0.20	[127.68	128.41	128.48]
C	7	127.30	126.88	-0.42	[126.90	125.73	126.94]
C	11	127.30	126.88	-0.42	[126.90	125.73	126.94]
C	20	123.90	123.25	-0.65	[123.25	123.07	123.67]
C	3	84.40	81.22	-3.18	[81.23	80.31	82.05]
C	2	75.30	70.50	-4.80	[70.49	71.12	70.39]
C	19	24.70	32.59	7.89	[32.62	32.18	30.46]
C	18	9.70	7.78	-1.92	[7.76	9.33	7.49]

13C chem shifts: RMSD=2.39ppm (MAE=1.32) N=18 {-4.80 7.89}

Fractions: 0.975 0.016 0.009

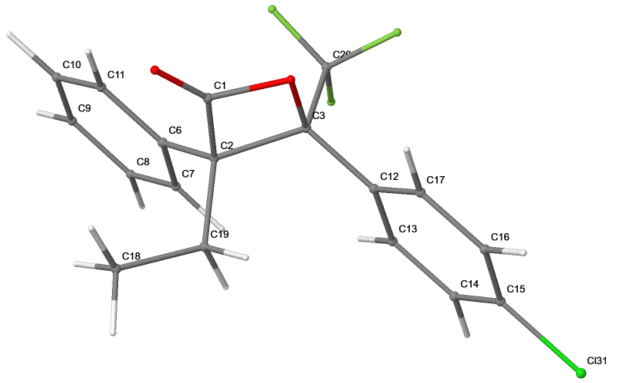

Conformer 1
Energy: -1604.49727 Hartree (Rel: 0.0 kcal/mol)
XYZ coordinates for conf 1:

C	1.22893	2.05860	0.11974
C	1.09163	0.66887	-0.50670
C	0.00246	0.49652	0.65280
O	0.19142	1.90435	1.01033
O	1.89657	3.04246	-0.02565
C	2.32163	-0.22564	-0.52886
C	2.19782	-1.57183	-0.90516
C	3.31662	-2.40263	-0.95970
C	4.58145	-1.89821	-0.64747
C	4.71584	-0.55845	-0.28159
C	3.59482	0.27249	-0.22135
C	-1.43471	0.18747	0.29375
C	-1.80509	-1.08786	-0.15715
C	-3.12901	-1.36869	-0.49049
C	-4.08854	-0.36426	-0.36754
C	-3.74526	0.90888	0.08258
C	-2.41658	1.17829	0.41180

C	1.40504	1.37617	-2.96799
C	0.45075	0.79712	-1.91793
C	0.45718	-0.30670	1.88608
F	1.68028	0.06857	2.30084
F	0.49283	-1.63126	1.63186
F	-0.39450	-0.10804	2.90929
H	5.45373	-2.54421	-0.69205
H	1.22383	-1.98000	-1.15986
H	3.19880	-3.44352	-1.24780
H	5.69476	-0.15321	-0.04113
H	3.71584	1.31361	0.05778
H	-1.06795	-1.87835	-0.24379
H	-3.40925	-2.35603	-0.84056
Cl	-5.76058	-0.71211	-0.78818
H	-4.50160	1.68027	0.17742
H	-2.14452	2.16675	0.76338
H	0.88134	1.47696	-3.92433
H	1.76697	2.36659	-2.67402
H	2.27371	0.72956	-3.12367
H	0.10710	-0.19627	-2.22749
H	-0.44418	1.42499	-1.84907

Conformer 2
Energy: -1604.49341 Hartree (Rel: 2.4 kcal/mol)
XYZ coordinates for conf 2:

C	1.31968	2.05706	0.04171
C	1.17629	0.67066	-0.59256
C	0.08977	0.49607	0.56486
O	0.27466	1.90527	0.92336
O	1.99915	3.03394	-0.09158
C	2.40933	-0.22571	-0.60283
C	2.28828	-1.57986	-0.94925
C	3.41287	-2.40224	-1.01685
C	4.68061	-1.88153	-0.74759
C	4.81197	-0.53336	-0.41240
C	3.68589	0.28952	-0.34010
C	-1.35057	0.16229	0.24755
C	-1.69142	-1.06817	-0.32914
C	-3.02341	-1.38890	-0.58913
C	-4.01904	-0.47094	-0.25828
C	-3.70527	0.75349	0.33000
C	-2.36942	1.06242	0.58399
C	-0.34155	1.94004	-2.36043
C	0.61138	0.78005	-2.04406
C	0.55514	-0.30362	1.79982
F	1.78737	0.05788	2.19896
F	0.56573	-1.62916	1.55342
F	-0.28133	-0.08444	2.83207
H	5.55717	-2.52100	-0.80132
H	1.31288	-2.00210	-1.17253
H	3.29639	-3.44952	-1.28139
H	5.79300	-0.11507	-0.20496
H	3.80555	1.33695	-0.08541
H	-0.92428	-1.79568	-0.57115
H	-3.28138	-2.34044	-1.04062
Cl	-5.70084	-0.86671	-0.58637
H	-4.49040	1.45531	0.58890
H	-2.11839	2.01340	1.03991
H	-0.61389	1.89670	-3.42033
H	-1.26556	1.90202	-1.77921
H	0.13694	2.90921	-2.18377
H	1.48885	0.86462	-2.69356
H	0.13775	-0.17604	-2.29245

Conformer 3
Energy: -1604.49280 Hartree (Rel: 2.8 kcal/mol)
XYZ coordinates for conf 3:

C	1.24327	1.54788	-1.43424
C	1.13114	0.12256	-0.88552
C	0.04716	0.81741	0.07316
O	0.21971	2.06454	-0.68071

O	1.88392	2.13437	-2.25893
C	2.40350	-0.48696	-0.31329
C	2.35739	-1.52468	0.63010
C	3.53180	-2.11311	1.10096
C	4.77360	-1.68112	0.63096
C	4.82964	-0.65716	-0.31542
C	3.65552	-0.06503	-0.78502
C	-1.39915	0.36107	0.05756
C	-1.82253	-0.76054	0.78701
C	-3.15548	-1.16738	0.76231
C	-4.07455	-0.44441	0.00275
C	-3.68091	0.67577	-0.72534
C	-2.34390	1.07328	-0.69220
C	0.25857	-2.22908	-1.72481
C	0.52558	-0.75229	-2.02904
C	0.51902	1.16796	1.49964
F	1.75765	1.69077	1.49545
F	0.52760	0.09403	2.31786
F	-0.30601	2.08303	2.04045
H	5.68748	-2.13973	0.99800
H	1.40433	-1.88001	1.00589
H	3.47262	-2.91155	1.83542
H	5.78886	-0.31246	-0.69171
H	3.71445	0.72838	-1.52216
H	-1.12219	-1.32546	1.39075
H	-3.47348	-2.03562	1.32883
Cl	-5.75692	-0.95458	-0.03299
H	-4.40468	1.23484	-1.30812
H	-2.03823	1.95015	-1.25047
H	-0.15874	-2.70436	-2.61917
H	1.17618	-2.76098	-1.46010
H	-0.46394	-2.36214	-0.91440
H	-0.39789	-0.27779	-2.38021
H	1.24247	-0.68214	-2.85595

Revised structure of 22{3g}, i.e. *cis* 27{3g-rev} ($CDCl_3$)

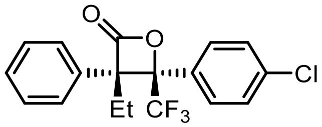

	Conf1	Conf2
Rel energy (kcal/mol):	0.00	3.59

C-nom	iGau	Exp	Calc	diff	1	2
C	1	169.10	169.81	0.71	[169.81	168.62]
C	15	134.90	135.13	0.23	[135.13	135.17]
C	6	132.50	133.93	1.43	[133.92	139.46]
C	12	130.10	131.82	1.72	[131.82	131.63]
C	8	128.60	127.86	-0.74	[127.86	127.80]
C	10	128.60	127.86	-0.74	[127.86	127.80]
C	14	128.20	127.52	-0.68	[127.52	127.35]
C	16	128.20	127.52	-0.68	[127.52	127.35]
C	9	128.10	127.22	-0.88	[127.22	127.07]
C	13	127.50	127.22	-0.28	[127.22	127.61]
C	17	127.50	127.22	-0.28	[127.22	127.61]
C	7	127.30	126.64	-0.66	[126.64	126.53]
C	11	127.30	126.64	-0.66	[126.64	126.53]
C	20	123.90	124.34	0.44	[124.34	124.26]
C	3	84.40	81.99	-2.41	[81.99	81.70]
C	2	75.30	75.19	-0.11	[75.19	74.33]
C	19	24.70	28.23	3.53	[28.22	30.98]
C	18	9.70	8.91	-0.79	[8.90	11.26]

13C chem shifts: RMSD=1.26ppm (MAE=0.94) N=18 {-2.41 3.53}

Fractions: 0.998 0.002

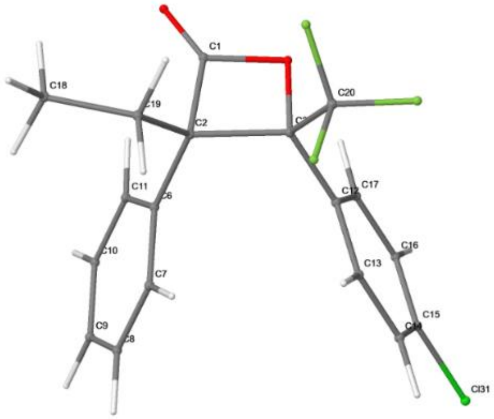

Conformer 1
Energy: -1604.49471 Hartree (Rel: 0.0 kcal/mol)
XYZ coordinates for conf 1:

C	-2.09894	-0.14547	1.49490
C	-1.72524	0.19306	0.04899
C	-0.73260	-1.05944	0.25366
O	-1.20873	-1.18348	1.63219
O	-2.87134	0.23833	2.32637
C	-1.02582	1.53718	-0.13012
C	-0.46799	1.86589	-1.37607
C	0.16190	3.09380	-1.57337
C	0.24113	4.01962	-0.52941
C	-0.31652	3.70593	0.70950
C	-0.94608	2.47386	0.90840
C	0.76329	-0.81843	0.20247
C	1.47048	-0.88376	-1.00720
C	2.84280	-0.64659	-1.04545
C	3.51381	-0.34078	0.13792
C	2.83473	-0.27258	1.35178

C	1.46039	-0.51208	1.37658
C	-3.96992	1.14877	-0.76528
C	-2.91758	0.04515	-0.93455
C	-1.11994	-2.36778	-0.46779
F	-2.34758	-2.79381	-0.11545
F	-1.11462	-2.19792	-1.81086
F	-0.26049	-3.35969	-0.17803
H	0.73074	4.97700	-0.68372
H	-0.52095	1.16020	-2.20013
H	0.59045	3.32709	-2.54411
H	-0.26790	4.41897	1.52773
H	-1.38863	2.25604	1.87480
H	0.96086	-1.12752	-1.93209
H	3.38240	-0.70088	-1.98454
Cl	5.24702	-0.04090	0.09550
H	3.36727	-0.03943	2.26728
H	0.93374	-0.46921	2.32251
H	-4.80531	0.96538	-1.44925
H	-4.36489	1.16566	0.25535
H	-3.55969	2.13796	-0.98833
H	-2.52469	0.05587	-1.95652
H	-3.39509	-0.92583	-0.79153

Conformer 2
Energy: -1604.48618 Hartree (Rel: 3.6 kcal/mol)
XYZ coordinates for conf 2:

C	-2.09100	0.16794	1.45640
C	-1.69539	0.44849	0.00166
C	-0.82758	-0.89412	0.22505
O	-1.31903	-0.95913	1.60157
O	-2.79237	0.65474	2.29563
C	-0.84706	1.70862	-0.16431
C	-0.34537	2.03549	-1.43534
C	0.43017	3.17766	-1.62551
C	0.71389	4.02187	-0.54826
C	0.21480	3.71115	0.71577
C	-0.55797	2.56187	0.90791
C	0.68620	-0.80862	0.18323
C	1.38275	-0.81445	-1.03444
C	2.77349	-0.73375	-1.06110
C	3.47346	-0.64593	0.14159
C	2.80517	-0.64027	1.36324
C	1.41230	-0.72189	1.37655
C	-4.28586	0.21812	-0.44266
C	-2.88243	0.43210	-1.02712
C	-1.32979	-2.17302	-0.47017
F	-2.63754	-2.39183	-0.23924
F	-1.16047	-2.10385	-1.81188
F	-0.66096	-3.25168	-0.02902
H	1.31455	4.91481	-0.69642
H	-0.56327	1.39487	-2.28546
H	0.81044	3.40928	-2.61655
H	0.42038	4.36283	1.56035
H	-0.94869	2.35063	1.89765
H	0.85043	-0.88853	-1.97507
H	3.30461	-0.73859	-2.00655
Cl	5.22986	-0.54302	0.11339
H	3.35960	-0.57605	2.29307
H	0.89170	-0.72534	2.32669
H	-5.01974	0.25169	-1.25506
H	-4.38561	-0.74910	0.05671
H	-4.54167	1.00086	0.27698
H	-2.87327	1.39661	-1.53992
H	-2.68376	-0.31871	-1.79530

Original (incorrect) structure of 23{3h} ($CDCl_3$)

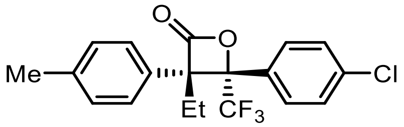

					Conf1	Conf2	Conf3
	Rel energy (kcal/mol):				0.00	2.44	2.77

C-nom	iGau	Exp	Calc	diff	1	2	3
C	1	169.40	169.35	-0.05	[169.35	168.83	170.38]
C	9	138.00	138.54	0.54	[138.55	138.19	138.11]
C	15	134.90	135.90	1.00	[135.89	136.07	136.14]
C	12	130.20	130.73	0.53	[130.74	130.20	130.25]
C	6	129.40	129.98	0.58	[129.93	133.53	129.44]
C	8	129.30	128.65	-0.65	[128.65	128.68	128.13]
C	10	129.30	128.65	-0.65	[128.65	128.68	128.13]
C	14	128.10	128.22	0.12	[128.22	128.13	128.05]
C	16	128.10	128.22	0.12	[128.22	128.13	128.05]
C	13	127.60	127.68	0.08	[127.66	128.43	128.45]
C	17	127.60	127.68	0.08	[127.66	128.43	128.45]
C	7	127.20	126.96	-0.24	[126.98	125.73	127.07]
C	11	127.20	126.96	-0.24	[126.98	125.73	127.07]
C	20	123.90	123.22	-0.68	[123.22	123.05	123.72]
C	3	84.40	81.17	-3.23	[81.18	80.24	81.96]
C	2	75.00	70.33	-4.67	[70.32	70.92	70.21]
C	19	24.60	32.59	7.99	[32.61	32.32	30.40]
C	24	20.80	20.12	-0.68	[20.12	20.04	20.16]
C	33	9.70	7.78	-1.92	[7.76	9.35	7.52]

13C chem shifts: RMSD=2.33ppm (MAE=1.26) N=19 {-4.67 7.99}

Fractions: 0.975 0.016 0.009

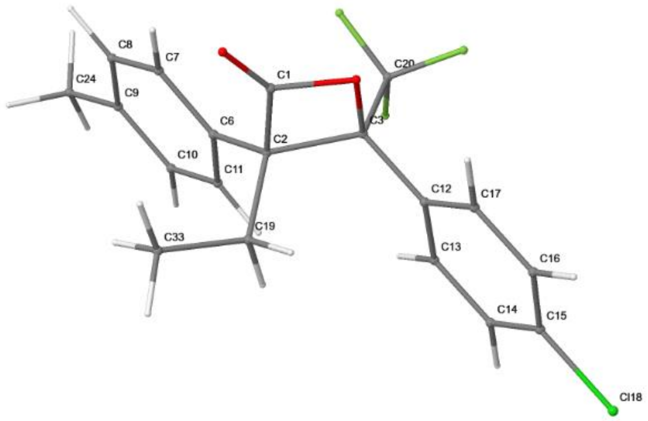

Conformer 1
Energy: -1643.81488 Hartree (Rel: 0.0 kcal/mol)
XYZ coordinates for conf 1:

C	0.76860	2.24420	-0.34674
C	0.77275	0.74281	-0.64384
C	-0.32230	0.73398	0.52286
O	-0.27242	2.19754	0.55236
O	1.34792	3.22857	-0.70778
C	2.07834	-0.01414	-0.46201
C	3.29475	0.65158	-0.25657
C	4.48404	-0.06358	-0.12309
C	4.50467	-1.46352	-0.18801
C	3.28674	-2.12351	-0.39549
C	2.09461	-1.41343	-0.53567
C	-1.71563	0.21537	0.24186
C	-1.95294	-1.15802	0.08570
C	-3.23679	-1.63363	-0.17566

C	-4.29009	-0.72543	-0.27796
C	-4.07946	0.64305	-0.12216
C	-2.78968	1.10704	0.13791
Cl	-5.91225	-1.31939	-0.60969
C	0.15353	0.49138	-2.04817
C	0.17598	0.27622	1.90656
F	1.34781	0.85172	2.22958
F	0.34134	-1.06174	1.95835
F	-0.71532	0.61627	2.85668
C	5.80064	-2.22821	-0.05265
H	3.31798	1.73505	-0.20696
H	5.41287	0.47956	0.03553
H	3.26803	-3.20931	-0.44916
H	1.17271	-1.96392	-0.70264
H	-1.14138	-1.87161	0.17549
H	-3.41432	-2.69644	-0.29736
H	-4.90785	1.33854	-0.20039
H	-2.62028	2.17049	0.26074
C	1.07274	0.91053	-3.20054
H	-0.08790	-0.57452	-2.12744
H	-0.79789	1.02926	-2.12292
H	6.36344	-1.90605	0.83124
H	5.62279	-3.30466	0.03300
H	6.44969	-2.06623	-0.92280
H	0.56468	0.74233	-4.15589
H	2.00229	0.33362	-3.20442
H	1.33206	1.97220	-3.13724

Conformer 2
Energy: -1643.81100 Hartree (Rel: 2.4 kcal/mol)
XYZ coordinates for conf 2:

C	0.85155	2.26127	-0.20994
C	0.85562	0.79450	-0.64910
C	-0.24090	0.67479	0.50560
O	-0.19738	2.13077	0.67070
O	1.43952	3.27215	-0.46765
C	2.16676	0.02953	-0.51818
C	3.38524	0.69381	-0.31921
C	4.58244	-0.01801	-0.26104
C	4.61035	-1.41258	-0.39955
C	3.39081	-2.07020	-0.60211
C	2.18987	-1.36336	-0.66606
C	-1.63481	0.16476	0.21688
C	-1.84284	-1.15561	-0.20274
C	-3.13180	-1.63556	-0.43290
C	-4.21809	-0.78562	-0.23059
C	-4.03664	0.52792	0.20002
C	-2.74261	0.99572	0.42597
Cl	-5.84662	-1.38288	-0.52119
C	0.32298	0.65145	-2.10965
C	0.26151	0.09668	1.84504
F	1.44417	0.62206	2.20997
F	0.39796	-1.24321	1.78306
F	-0.61967	0.37438	2.82521
C	5.91495	-2.17267	-0.34459
H	3.40352	1.77332	-0.21446
H	5.51233	0.52376	-0.10361
H	3.37689	-3.15183	-0.71221
H	1.26788	-1.91307	-0.83270
H	-1.00388	-1.82831	-0.34494
H	-3.28720	-2.65675	-0.76279
H	-4.89116	1.17623	0.35975
H	-2.59408	2.01646	0.75958
C	-0.73212	1.65401	-2.59474
H	1.20578	0.73842	-2.75158
H	-0.04471	-0.37311	-2.23263
H	6.49724	-1.90212	0.54403
H	5.74710	-3.25389	-0.32273
H	6.54111	-1.95040	-1.21816
H	-0.96725	1.44265	-3.64335
H	-0.35927	2.68251	-2.54287

H	-1.66444	1.59782	-2.02846

Conformer 3
Energy: -1643.81047 Hartree (Rel: 2.8 kcal/mol)
XYZ coordinates for conf 3:

C	0.79370	2.01099	-1.10563
C	0.81051	0.49566	-0.88570
C	-0.28462	0.87132	0.22627
O	-0.23690	2.26344	-0.23526
O	1.35625	2.81556	-1.79179
C	2.14552	-0.11703	-0.48818
C	3.34578	0.49738	-0.87189
C	4.57614	-0.08641	-0.56792
C	4.65608	-1.29855	0.12817
C	3.45332	-1.91507	0.49889
C	2.22039	-1.33974	0.19537
C	-1.69159	0.31128	0.14147
C	-2.00045	-0.97173	0.61887
C	-3.29838	-1.47271	0.53330
C	-4.29781	-0.68226	-0.03290
C	-4.01848	0.59652	-0.50884
C	-2.71544	1.08686	-0.41721
Cl	-5.93665	-1.31013	-0.14408
C	0.22754	-0.15197	-2.18176
C	0.21544	0.93688	1.68435
F	1.40650	1.55419	1.77115
F	0.34381	-0.28677	2.24051
F	-0.65784	1.63340	2.43507
C	5.98971	-1.91078	0.48685
H	3.32276	1.43572	-1.41564
H	5.49008	0.41403	-0.87935
H	3.47851	-2.86284	1.03167
H	1.31516	-1.85593	0.49620
H	-1.23680	-1.59269	1.07212
H	-3.52771	-2.46539	0.90465
H	-4.80425	1.20568	-0.94189
H	-2.49871	2.08527	-0.77809
C	0.08614	-1.67638	-2.20794
H	-0.74267	0.30979	-2.39838
H	0.90368	0.15751	-2.98779
H	6.30896	-1.60169	1.49111
H	5.94180	-3.00504	0.48526
H	6.77295	-1.60166	-0.21296
H	-0.33332	-1.97662	-3.17433
H	-0.58747	-2.04400	-1.42850
H	1.05213	-2.17486	-2.09238

Revised structure of 23{3h}, i.e. *cis* 28{3h-rev} ($CDCl_3$)

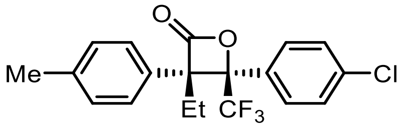

					Conf1	Conf2	Conf3
Rel energy (kcal/mol):					0.00	2.17	3.57

C-nom	iGau	Exp	Calc	diff	1	2	3
C	1	169.40	169.96	0.56	[169.95	170.52	168.80]
C	9	138.00	137.91	-0.09	[137.92	137.53	137.79]
C	15	134.90	135.07	0.17	[135.06	135.44	135.11]
C	12	130.20	131.93	1.73	[131.92	132.17	131.68]
C	6	129.40	130.87	1.47	[130.90	129.33	136.49]
C	8	129.30	128.64	-0.66	[128.66	127.77	128.53]
C	10	129.30	128.64	-0.66	[128.66	127.77	128.53]
C	14	128.10	127.51	-0.59	[127.51	127.67	127.34]
C	16	128.10	127.51	-0.59	[127.51	127.67	127.34]
C	13	127.60	127.24	-0.36	[127.22	127.92	127.62]
C	17	127.60	127.24	-0.36	[127.22	127.92	127.62]
C	7	127.20	126.71	-0.49	[126.68	127.97	126.58]
C	11	127.20	126.71	-0.49	[126.68	127.97	126.58]
C	20	123.90	124.39	0.49	[124.38	124.81	124.36]
C	3	84.40	81.99	-2.41	[81.97	82.73	81.76]
C	2	75.00	75.01	0.01	[75.03	74.30	74.15]
C	19	24.60	28.11	3.51	[28.09	28.45	30.99]
C	24	20.80	19.84	-0.96	[19.84	19.85	19.79]
C	33	9.70	8.90	-0.80	[8.90	8.64	11.18]

13C chem shifts: RMSD=1.21ppm (MAE=0.86) N=19 {-2.41 3.51}

Fractions: 0.973 0.025 0.002

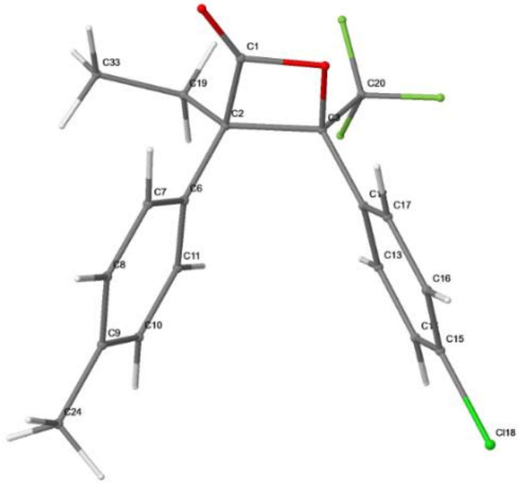

Conformer 1
Energy: -1643.81237 Hartree (Rel: 0.0 kcal/mol)
XYZ coordinates for conf 1:

C	2.20155	-0.03297	1.49151
C	1.74586	0.29154	0.06611
C	1.01193	-1.13483	0.22138
O	1.52146	-1.22344	1.59051
O	2.90381	0.45168	2.33254
C	0.80165	1.48445	-0.03895
C	0.56469	2.34271	1.03983
C	-0.29037	3.44047	0.90605
C	-0.93462	3.71783	-0.30297
C	-0.68898	2.85706	-1.38481
C	0.16296	1.76408	-1.25864
C	-0.50279	-1.18105	0.18721

C	-1.19986	-1.32526	-1.02155
C	-2.59271	-1.35088	-1.04357
C	-3.29451	-1.23066	0.15525
C	-2.62550	-1.08983	1.36843
C	-1.23046	-1.06561	1.37688
Cl	-5.05379	-1.26205	0.13354
C	2.92481	0.41614	-0.93632
C	1.63128	-2.31424	-0.55766
F	2.92251	-2.51215	-0.23132
F	1.57494	-2.09358	-1.89219
F	0.98080	-3.46332	-0.30600
C	-1.86361	4.89990	-0.45004
H	1.05769	2.17410	1.99169
H	-0.45262	4.09153	1.76158
H	-1.17200	3.04644	-2.34077
H	0.32676	1.12353	-2.12077
H	-0.66521	-1.42700	-1.95879
H	-3.12404	-1.46434	-1.98210
H	-3.18107	-1.00312	2.29575
H	-0.70955	-0.96651	2.32172
C	3.75363	1.68996	-0.72573
H	2.51670	0.39939	-1.95238
H	3.57958	-0.45254	-0.84619
H	-1.53717	5.56547	-1.25846
H	-2.88316	4.57609	-0.69375
H	-1.91044	5.48776	0.47170
H	4.15799	1.73516	0.29033
H	3.15972	2.59252	-0.89644
H	4.59522	1.69910	-1.42648

Conformer 2
Energy: -1643.80891 Hartree (Rel: 2.2 kcal/mol)
XYZ coordinates for conf 2:

C	2.17567	0.19203	1.57809
C	1.73587	0.44980	0.13399
C	1.08436	-1.01579	0.31646
O	1.53186	-1.01169	1.71356
O	2.85377	0.73301	2.40423
C	0.73775	1.60155	0.01915
C	0.74969	2.65214	0.94469
C	-0.11975	3.73774	0.80744
C	-1.02662	3.81456	-0.25359
C	-1.02791	2.76465	-1.18518
C	-0.16362	1.68127	-1.05546
C	-0.42042	-1.19008	0.23337
C	-1.05380	-1.60889	-0.94622
C	-2.43944	-1.74513	-1.00436
C	-3.19963	-1.46180	0.12925
C	-2.59475	-1.05020	1.31446
C	-1.20720	-0.91730	1.35912
Cl	-4.94922	-1.63257	0.06196
C	2.96765	0.67170	-0.79170
C	1.83553	-2.18837	-0.35106
F	3.13051	-2.22784	0.01613
F	1.79909	-2.09883	-1.70142
F	1.29180	-3.36842	-0.00764
C	-1.97750	4.97934	-0.39954
H	1.44434	2.63562	1.77789
H	-0.08722	4.53704	1.54389
H	-1.71666	2.79494	-2.02642
H	-0.19711	0.89200	-1.79840
H	-0.47566	-1.83612	-1.83407
H	-2.91950	-2.07115	-1.92046
H	-3.19423	-0.83844	2.19300
H	-0.73845	-0.60736	2.28531
C	2.70185	0.90008	-2.28344
H	3.66885	-0.15634	-0.66763
H	3.46758	1.55655	-0.37877
H	-1.89454	5.44017	-1.39130
H	-3.02028	4.65813	-0.28277
H	-1.78011	5.75206	0.34963

```
H          2.10859        1.80107       -2.45973
H          2.18697        0.05180       -2.74268
H          3.66004        1.02486       -2.79955

Conformer 3
Energy: -1643.80668 Hartree (Rel:  3.6 kcal/mol)
XYZ coordinates for conf 3:
C          2.15903        0.35601        1.45494
C          1.67651        0.59986        0.02009
C          1.12681       -0.90770        0.19885
O          1.64158       -0.91172        1.56846
O          2.76383        0.94822        2.30163
C          0.56955        1.64869       -0.06867
C          0.13857        2.38401        1.04015
C         -0.87183        3.34227        0.91421
C         -1.48840        3.59396       -0.31450
C         -1.05014        2.85647       -1.42613
C         -0.04105        1.90619       -1.30818
C         -0.36855       -1.15752        0.16961
C         -1.06334       -1.28242       -1.04269
C         -2.43797       -1.50990       -1.05686
C         -3.12351       -1.61272        0.15289
C         -2.45631       -1.49385        1.36937
C         -1.07969       -1.26649        1.37008
Cl        -4.85975       -1.89937        0.14042
C          2.80703        0.88415       -1.03217
C          1.89088       -2.01897       -0.54433
F          3.21828       -1.94440       -0.33294
F          1.68881       -1.94990       -1.88149
F          1.48894       -3.23340       -0.13294
C         -2.59174        4.61669       -0.44985
H          0.59916        2.23591        2.01127
H         -1.17876        3.90429        1.79290
H         -1.50415        3.03178       -2.39881
H          0.27606        1.36120       -2.19321
H         -0.54086       -1.21080       -1.98908
H         -2.96792       -1.60539       -1.99815
H         -2.99854       -1.57959        2.30465
H         -0.55798       -1.18133        2.31579
C          4.23639        0.98397       -0.48087
H          2.56264        1.83323       -1.51480
H          2.76758        0.12787       -1.81935
H         -2.39793        5.30621       -1.27992
H         -3.55651        4.13405       -0.65227
H         -2.70191        5.20868        0.46389
H          4.56898        0.05101       -0.01818
H          4.32288        1.78169        0.26229
H          4.92212        1.21161       -1.30409
```

Original (incorrect) structure of 24{3m} ($CDCl_3$)

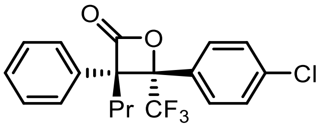

					**Conf1**	**Conf2**	**Conf3**
		Rel energy (kcal/mol):			0.00	2.44	2.83
**C-nom**	**iGau**	**Exp**	**Calc**	**diff**	**1**	**2**	**3**
C	1	169.20	169.22	0.02	[169.22	168.73	170.13]
C	15	135.00	135.92	0.92	[135.92	136.06	136.09]
C	6	133.00	133.48	0.48	[133.43	136.63	132.81]
C	12	130.00	130.65	0.65	[130.66	130.09	130.18]
C	8	128.80	127.84	-0.96	[127.84	127.91	127.37]
C	10	128.80	127.84	-0.96	[127.84	127.91	127.37]
C	14	128.60	128.25	-0.35	[128.25	128.14	128.03]
C	16	128.60	128.25	-0.35	[128.25	128.14	128.03]
C	13	128.20	127.73	-0.47	[127.71	128.46	128.47]
C	17	128.20	127.73	-0.47	[127.71	128.46	128.47]
C	9	127.60	127.75	0.15	[127.76	127.49	127.41]
C	7	127.20	126.73	-0.47	[126.75	125.62	126.93]
C	11	127.20	126.73	-0.47	[126.75	125.62	126.93]
C	20	123.90	123.21	-0.69	[123.21	123.05	123.67]
C	3	84.30	81.23	-3.07	[81.24	80.36	82.09]
C	2	74.90	70.33	-4.57	[70.32	70.81	70.58]
C	19	33.60	40.50	6.90	[40.53	40.24	38.07]
C	33	18.70	17.12	-1.58	[17.10	18.34	16.81]
C	38	14.20	12.70	-1.50	[12.70	12.74	12.71]

13C chem shifts: RMSD=2.15ppm (MAE=1.32) N=19 {-4.57 6.90}
Fractions: 0.976 0.016 0.008

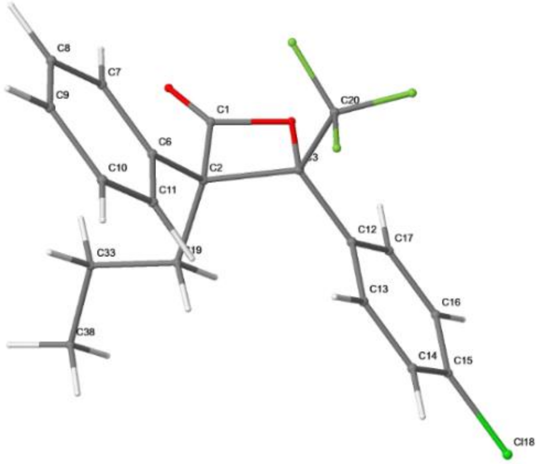

Conformer 1
Energy: -1643.81026 Hartree (Rel: 0.0 kcal/mol)
XYZ coordinates for conf 1:

C	-1.20884	0.35526	1.98124
C	-1.07649	0.48086	0.46129
C	0.03753	-0.64640	0.68819
O	-0.15360	-0.51406	2.13428
O	-1.88516	0.80836	2.86002
C	-2.30255	0.17153	-0.38484
C	-3.57154	0.02544	0.19145
C	-4.68975	-0.22402	-0.60735
C	-4.55673	-0.33347	-1.99199
C	-3.29612	-0.18581	-2.57576
C	-2.18021	0.06893	-1.77900
C	1.46952	-0.38185	0.27656

C	1.84089	-0.37894	-1.07585
C	3.16025	-0.13231	-1.45164
C	4.11426	0.11004	-0.46381
C	3.77000	0.10800	0.88629
C	2.44592	-0.13887	1.24986
Cl	5.78052	0.42353	-0.93190
C	-0.46514	1.86439	0.10281
C	-0.38611	-2.08750	0.34686
F	-1.60327	-2.37830	0.83950
F	-0.41668	-2.29487	-0.98597
F	0.48465	-2.96549	0.87876
H	-5.42674	-0.52950	-2.61233
H	-3.69164	0.11500	1.26570
H	-5.66539	-0.33237	-0.14183
H	-3.17935	-0.26727	-3.65282
H	-1.20980	0.18892	-2.25227
H	1.10834	-0.57825	-1.84998
H	3.44137	-0.12961	-2.49895
H	4.52201	0.29429	1.64512
H	2.17322	-0.14271	2.29879
C	-1.43561	3.03953	0.29187
H	-0.12344	1.82819	-0.93873
H	0.42971	2.03169	0.71382
H	-1.80302	3.04767	1.32517
H	-2.31315	2.89977	-0.34966
C	-0.77009	4.38193	-0.03124
H	-1.47507	5.20956	0.10335
H	-0.41522	4.41035	-1.06881
H	0.09195	4.56737	0.62096

Conformer 2
Energy: -1643.80575 Hartree (Rel: 2.4 kcal/mol)
XYZ coordinates for conf 2:

C	-1.33514	1.02202	1.71752
C	-1.19495	0.71762	0.22295
C	-0.13484	-0.34753	0.76477
O	-0.31280	0.19243	2.11583
O	-2.00108	1.71812	2.42877
C	-2.44074	0.22816	-0.50778
C	-3.71630	0.37061	0.05504
C	-4.85282	-0.02594	-0.65318
C	-4.73317	-0.56960	-1.93273
C	-3.46631	-0.70942	-2.50392
C	-2.33109	-0.30956	-1.79893
C	1.30885	-0.31895	0.31526
C	1.65110	-0.57583	-1.01885
C	2.98574	-0.58609	-1.42312
C	3.98244	-0.34635	-0.47842
C	3.66703	-0.10252	0.85753
C	2.32863	-0.09413	1.24861
Cl	5.66760	-0.35488	-0.98127
C	-0.59885	1.93859	-0.54531
C	-0.63240	-1.80620	0.84054
F	-1.87102	-1.89042	1.35697
F	-0.64427	-2.38694	-0.37624
F	0.18233	-2.52917	1.63239
H	-5.61786	-0.87969	-2.48169
H	-3.82669	0.79870	1.04539
H	-5.83282	0.09230	-0.19925
H	-3.35865	-1.12889	-3.50033
H	-1.35643	-0.41815	-2.26536
H	0.88299	-0.78241	-1.75622
H	3.24480	-0.78015	-2.45804
H	4.45281	0.07554	1.58337
H	2.07643	0.09565	2.28573
C	0.36397	2.87330	0.20456
H	-1.46013	2.52917	-0.87745
H	-0.11859	1.56093	-1.45579
H	1.23514	2.32290	0.57255
H	-0.14318	3.28811	1.08450
C	0.83420	4.02182	-0.69604

H	1.50091	4.69798	-0.15006
H	-0.01404	4.61184	-1.06369
H	1.38161	3.64374	-1.56790

Conformer 3
Energy: -1643.80638 Hartree (Rel: 2.8 kcal/mol)
XYZ coordinates for conf 3:

C	-1.26495	-0.58263	2.03840
C	-1.14441	0.25566	0.76231
C	-0.07730	-0.88332	0.38438
O	-0.25503	-1.45633	1.72351
O	-1.90257	-0.57029	3.05220
C	-2.41724	0.43251	-0.05496
C	-3.66918	0.36875	0.57464
C	-4.84265	0.58528	-0.15065
C	-4.78594	0.87017	-1.51544
C	-3.54397	0.94387	-2.14939
C	-2.37038	0.73150	-1.42516
C	1.37387	-0.52453	0.12836
C	1.80211	-0.04494	-1.11905
C	3.13945	0.28104	-1.33842
C	4.05820	0.12343	-0.30146
C	3.65982	-0.35637	0.94392
C	2.31835	-0.67924	1.15115
Cl	5.74624	0.53418	-0.57321
C	-0.51727	1.62241	1.18128
C	-0.56776	-1.99095	-0.57110
F	-1.81106	-2.39675	-0.25980
F	-0.57511	-1.59240	-1.86115
F	0.24267	-3.06145	-0.48225
H	-5.69926	1.03541	-2.08002
H	-3.72883	0.15012	1.63529
H	-5.80187	0.52778	0.35641
H	-3.48418	1.16860	-3.21069
H	-1.41694	0.79998	-1.93715
H	1.10235	0.07116	-1.93816
H	3.46108	0.65155	-2.30547
H	4.38332	-0.48131	1.74204
H	2.00902	-1.06205	2.11640
C	-0.23849	2.65302	0.07914
H	0.40864	1.42708	1.73615
H	-1.22141	2.05792	1.90183
H	-1.17339	2.91552	-0.42678
H	0.42446	2.22641	-0.68220
C	0.40393	3.92034	0.65660
H	0.58263	4.66050	-0.13105
H	1.36650	3.69847	1.13269
H	-0.24302	4.38404	1.41115

Revised structure of 24{3m}, i.e. *cis* 29{3m-rev} ($CDCl_3$)

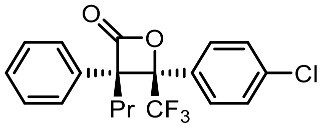

					Conf1	Conf2
		Rel energy (kcal/mol):			0.00	3.43

C-nom	iGau	Exp	Calc	diff	1	2
C	1	169.20	169.84	0.64	[169.84	168.66]
C	15	135.00	135.09	0.09	[135.09	135.14]
C	6	133.00	134.33	1.33	[134.31	139.46]
C	12	130.00	131.80	1.80	[131.80	131.54]
C	8	128.80	127.87	-0.93	[127.87	127.79]
C	10	128.80	127.87	-0.93	[127.87	127.79]
C	14	128.60	127.51	-1.09	[127.51	127.36]
C	16	128.60	127.51	-1.09	[127.51	127.36]
C	13	128.20	127.24	-0.96	[127.24	127.64]
C	17	128.20	127.24	-0.96	[127.24	127.64]
C	9	127.60	127.19	-0.41	[127.19	127.07]
C	7	127.20	126.47	-0.73	[126.47	126.62]
C	11	127.20	126.47	-0.73	[126.47	126.62]
C	20	123.90	124.34	0.44	[124.34	124.28]
C	3	84.30	81.98	-2.32	[81.98	81.72]
C	2	74.90	74.95	0.05	[74.95	73.93]
C	19	33.60	36.13	2.53	[36.12	39.01]
C	33	18.70	18.27	-0.43	[18.26	20.45]
C	38	14.20	13.11	-1.09	[13.11	13.54]

13C chem shifts: RMSD=1.17ppm (MAE=0.98) N=19 {-2.32 2.53}

Fractions: 0.997 0.003

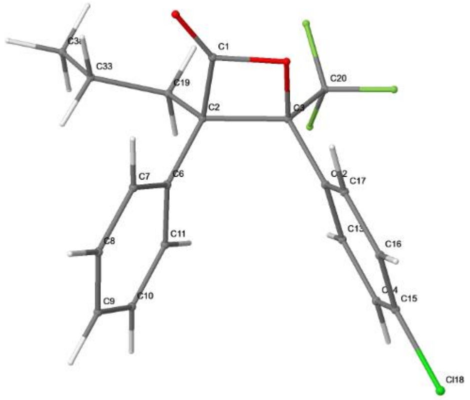

Conformer 1
Energy: -1643.80773 Hartree (Rel: 0.0 kcal/mol)
XYZ coordinates for conf 1:

C	1.74815	-0.32240	1.69256
C	1.49633	0.06772	0.23310
C	0.42419	-1.13150	0.33783
O	0.79616	-1.31215	1.74164
O	2.47914	0.00010	2.58529
C	0.88426	1.45211	0.04032
C	0.77804	2.36759	1.09520
C	0.23085	3.63652	0.88466
C	-0.21755	4.00849	-0.38216
C	-0.11138	3.10377	-1.44212
C	0.43685	1.83920	-1.23312
C	-1.04888	-0.80485	0.19130
C	-1.67108	-0.79640	-1.06593
C	-3.02214	-0.48053	-1.19165
C	-3.75756	-0.16977	-0.04850

C	-3.16362	-0.17435	1.21104
C	-1.80973	-0.49241	1.32351
Cl	-5.46427	0.22967	-0.20115
C	2.74316	-0.12723	-0.67025
C	0.78724	-2.44352	-0.38951
F	1.96146	-2.94758	0.03450
F	0.88476	-2.24370	-1.72491
F	-0.14465	-3.39037	-0.18516
H	-0.64318	4.99452	-0.54553
H	1.13671	2.10395	2.08466
H	0.16045	4.33228	1.71608
H	-0.45508	3.38215	-2.43449
H	0.51309	1.15093	-2.06994
H	-1.11155	-1.04377	-1.96053
H	-3.49599	-0.47797	-2.16707
H	-3.74627	0.06310	2.09435
H	-1.35029	-0.50648	2.30466
C	3.84998	0.90821	-0.41160
H	2.42455	-0.07380	-1.71751
H	3.15714	-1.12687	-0.51939
H	4.12936	0.88373	0.64843
H	3.46916	1.91607	-0.61133
C	5.08524	0.64301	-1.27939
H	5.86457	1.38881	-1.08796
H	4.83751	0.68476	-2.34725
H	5.51166	-0.34653	-1.07475

Conformer 2
Energy: -1643.80227 Hartree (Rel: 3.4 kcal/mol)
XYZ coordinates for conf 2:

C	1.76965	0.12173	1.63661
C	1.46490	0.41189	0.16134
C	0.56050	-0.91456	0.33089
O	0.96254	-0.98528	1.73588
O	2.43074	0.59254	2.51675
C	0.64940	1.68847	-0.04387
C	0.32607	2.54774	1.01369
C	-0.41483	3.71141	0.78546
C	-0.84766	4.03076	-0.50058
C	-0.52903	3.18076	-1.56347
C	0.21507	2.02444	-1.33713
C	-0.94561	-0.80469	0.19230
C	-1.56321	-0.80395	-1.06730
C	-2.94798	-0.70138	-1.18260
C	-3.72149	-0.59779	-0.02715
C	-3.13227	-0.59769	1.23456
C	-1.74459	-0.70140	1.33670
Cl	-5.47065	-0.46737	-0.16736
C	2.70365	0.37561	-0.80127
C	1.08586	-2.20445	-0.32622
F	2.37552	-2.43744	-0.01829
F	0.99555	-2.14463	-1.67576
F	0.37894	-3.27101	0.08380
H	-1.42383	4.93469	-0.67671
H	0.66498	2.32983	2.02085
H	-0.64776	4.36732	1.61961
H	-0.85750	3.41889	-2.57135
H	0.46137	1.37956	-2.17615
H	-0.97328	-0.88977	-1.97189
H	-3.41808	-0.70151	-2.15983
H	-3.74371	-0.52090	2.12693
H	-1.28548	-0.70938	2.31800
C	4.07828	0.16482	-0.14336
H	2.72973	1.33151	-1.33133
H	2.54740	-0.38765	-1.56818
H	4.09510	-0.77916	0.41157
H	4.25821	0.96005	0.58855
C	5.19863	0.16054	-1.18868
H	6.17618	0.02444	-0.71335
H	5.22658	1.10472	-1.74638
H	5.06265	-0.65126	-1.91377

Original (incorrect - *cis*) structure of 31{27} ($CDCl_3$)

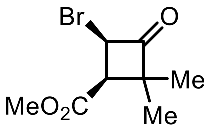

	Conf1	Conf2
Rel energy (kcal/mol):	0.00	0.68

iGau	jGau	Jexp	Jcalc	diff	1	2
13	14	7.90	8.47	0.57	[8.59	8.08]

For Js: RMSD=0.57Hz N=1 {0.57 0.57}

C-nom	iGau	Exp	Calc	diff	1	2
C	5	203.40	207.91	4.51	[208.01	207.60]
C	9	169.80	167.16	-2.64	[167.33	166.63]
C	4	62.50	64.79	2.29	[64.66	65.19]
C	12	52.70	52.47	-0.23	[52.48	52.45]
C	2	52.00	49.74	-2.26	[49.85	49.41]
C	1	46.30	44.42	-1.88	[44.61	43.84]
C	7	24.10	22.18	-1.92	[22.25	21.97]
C	8	18.90	22.30	3.40	[22.19	22.66]

13C chem shifts: RMSD=2.66ppm (MAE=2.39) N=8 {-2.64 4.51}

Fractions: 0.758 0.242

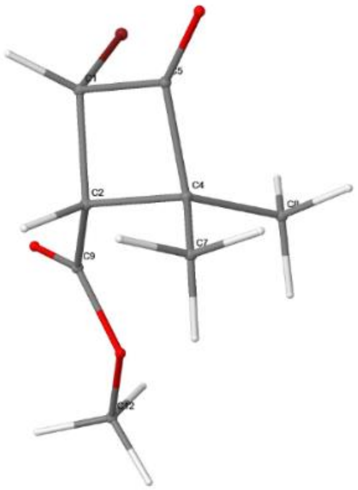

Conformer 1
Energy: -3108.52504 Hartree (Rel: 0.0 kcal/mol)
XYZ coordinates for conf 1:

C	-0.47611	0.63939	-0.92022
C	-0.11992	-0.85778	-0.68026
Br	0.40030	1.95427	0.27037
C	-1.48831	-1.09937	0.06106
C	-1.89567	0.30629	-0.42164
O	-2.93838	0.90727	-0.46084
C	-2.34685	-2.23719	-0.49826
C	-1.44440	-1.14699	1.60143
C	1.20364	-1.23230	-0.04681
O	1.35130	-1.93502	0.93117
O	2.21878	-0.71782	-0.76224
C	3.54394	-1.01002	-0.27570
H	-0.37043	1.03466	-1.92966
H	-0.13894	-1.35211	-1.66037
H	-3.36280	-2.18104	-0.09324
H	-2.41400	-2.19706	-1.59119
H	-1.91940	-3.20610	-0.21652
H	-1.04785	-2.10682	1.93965
H	-0.81954	-0.35485	2.02270

H	-2.46228	-1.02456	1.98714
H	4.22351	-0.52161	-0.97328
H	3.67434	-0.60866	0.73206
H	3.71533	-2.08907	-0.26238

Conformer 2
Energy: -3108.52612 Hartree (Rel: 0.7 kcal/mol)
XYZ coordinates for conf 2:

C	1.01014	0.07014	-0.90445
C	-0.51149	0.36990	-0.82680
Br	1.73454	-1.36469	0.24755
C	-0.25202	1.62941	0.09152
C	1.23916	1.44645	-0.25037
O	2.21395	2.14267	-0.12538
C	-0.86620	2.94659	-0.38478
C	-0.49704	1.41072	1.59692
C	-1.50828	-0.70840	-0.46089
O	-1.36018	-1.89499	-0.65902
O	-2.63232	-0.16388	0.04255
C	-3.69372	-1.09064	0.35111
H	1.44138	-0.09821	-1.89038
H	-0.81905	0.72010	-1.82202
H	-0.45524	3.78648	0.18544
H	-0.66645	3.12720	-1.44694
H	-1.95179	2.92989	-0.23791
H	-1.57018	1.41779	1.80651
H	-0.08190	0.46118	1.94873
H	-0.02318	2.21948	2.16308
H	-4.51189	-0.47898	0.72980
H	-4.00000	-1.63019	-0.54826
H	-3.36536	-1.80587	1.10881

Revised structure of 31{27}, i.e. *trans* 32{27-rev} ($CDCl_3$)

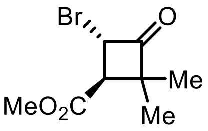

	Conf1	Conf2
Rel energy (kcal/mol):	0.00	1.24

iGau	jGau	Jexp	Jcalc	diff	1	2
13	14	7.90	8.43	0.53	[8.43	8.42]

For Js: RMSD=0.53Hz N=1 {0.53 0.53}

C-nom	iGau	Exp	Calc	diff	1	2
C	5	203.40	204.46	1.06	[204.43	204.67]
C	9	169.80	168.92	-0.88	[169.21	166.62]
C	4	62.50	63.84	1.34	[63.77	64.45]
C	2	52.70	54.42	1.72	[54.25	55.79]
C	12	52.00	52.91	0.91	[52.93	52.79]
C	1	46.30	44.54	-1.76	[44.58	44.25]
C	7	24.10	23.05	-1.05	[23.02	23.32]
C	8	18.90	17.41	-1.49	[17.41	17.40]

13C chem shifts: RMSD=1.32ppm (MAE=1.28) N=8 {-1.76 1.72}

Fractions: 0.890 0.110

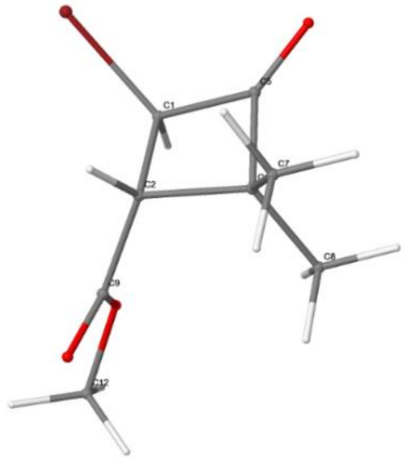

Conformer 1
Energy: -3108.53366 Hartree (Rel: 0.0 kcal/mol)
XYZ coordinates for conf 1:

C	-0.80524	-0.06422	-0.49193
C	0.49200	0.02756	0.33924
Br	-2.27778	-1.17324	0.16769
C	0.50063	1.60834	0.12875
C	-0.95562	1.45290	-0.35964
O	-1.87987	2.21500	-0.47014
C	0.64781	2.43457	1.40840
C	1.43708	2.11131	-0.98134
C	1.66488	-0.75100	-0.20614
O	1.76377	-1.14352	-1.35302
O	2.60825	-0.92511	0.73330
C	3.79791	-1.62362	0.31059
H	-0.60161	-0.38165	-1.51774
H	0.33615	-0.20851	1.39305
H	0.43751	3.48926	1.20165
H	-0.04037	2.09658	2.19005
H	1.67122	2.35554	1.79200
H	2.47804	2.07146	-0.64055
H	1.35731	1.51853	-1.89727
H	1.19712	3.15206	-1.22165

H	4.44048	-1.66009	1.18955
H	3.54526	-2.63254	-0.02369
H	4.28741	-1.08333	-0.50303

Conformer 2
Energy: -3108.53169 Hartree (Rel: 1.2 kcal/mol)
XYZ coordinates for conf 2:

C	-0.66114	-0.12792	-0.43700
C	0.47048	0.14709	0.58134
Br	-2.24838	-1.10709	0.16593
C	0.53743	1.65933	0.08345
C	-0.80793	1.38613	-0.62123
O	-1.68381	2.09474	-1.04297
C	0.46719	2.71408	1.18992
C	1.66708	1.96225	-0.91435
C	1.74753	-0.65741	0.52147
O	2.56297	-0.68351	1.42154
O	1.89938	-1.30657	-0.64716
C	3.11608	-2.06774	-0.79225
H	-0.30951	-0.62999	-1.33943
H	0.10786	0.11788	1.61035
H	0.31479	3.70744	0.75472
H	-0.35496	2.51638	1.88546
H	1.40418	2.72543	1.75778
H	2.63067	1.98276	-0.39267
H	1.72881	1.21923	-1.71536
H	1.50457	2.94435	-1.36979
H	3.05782	-2.52264	-1.78035
H	3.98522	-1.40986	-0.72056
H	3.17546	-2.83497	-0.01709

Originally assigned (incorrect) structure of 35{2n'} (minor product) ($CDCl_3$)

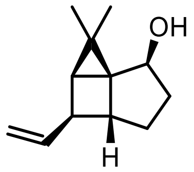

					Conf1	Conf2	Conf3	Conf4
Rel energy (kcal/mol):					0.00	0.15	1.63	1.66

C-nom	iGau	Exp	Calc	diff	1	2	3	4
C	13	138.40	138.75	0.35	[137.61	140.20	137.72	140.08]
C	14	114.40	112.07	-2.33	[113.12	110.74	112.74	111.09]
C	5	69.70	74.65	4.95	[74.56	74.63	75.39	75.45]
C	1	47.50	41.39	-6.11	[41.54	41.17	41.52	41.55]
C	8	38.80	40.39	1.59	[40.69	39.42	44.62	43.22]
C	2	37.90	36.99	-0.91	[37.48	35.77	41.34	39.99]
C	4	34.90	36.75	1.85	[36.53	36.79	38.22	38.28]
C	7	26.10	28.97	2.87	[27.65	30.38	29.40	32.27]
C	3	25.50	28.35	2.85	[27.99	27.97	33.72	33.46]
C	9	24.80	26.59	1.79	[26.61	26.62	26.19	26.28]
C	10	21.10	22.53	1.43	[22.55	22.59	21.91	21.95]
C	11	15.30	19.58	4.28	[20.53	18.45	20.12	17.92]

13C chem shifts: RMSD=3.08ppm (MAE=2.61) N=12 {-6.11 4.95}

Fractions: 0.526 0.408 0.034 0.032

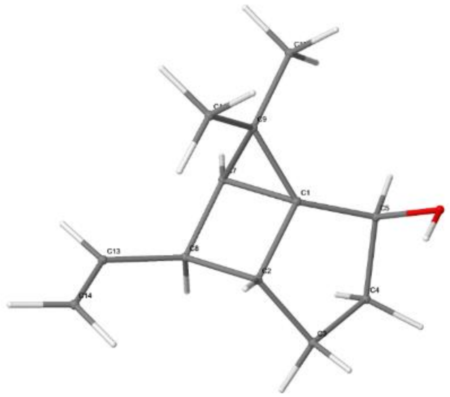

Conformer 1
Energy: -543.23400 Hartree (Rel: 0.0 kcal/mol)
XYZ coordinates for conf 1:

C	0.57085	0.26274	-0.02700
C	0.09229	-1.05844	0.63291
C	1.28089	-2.02609	0.44063
C	2.20820	-1.35138	-0.60391
C	2.03438	0.17181	-0.38765
O	2.90091	0.69051	0.63401
C	-0.64690	0.23780	-0.97327
C	-1.08121	-1.12544	-0.40934
C	-0.42329	1.38279	0.01170
C	0.02048	2.67322	-0.67169
C	-1.28739	1.64935	1.23626
H	-0.23055	-1.00667	1.67789
C	-2.48813	-1.39094	0.04489
C	-3.54384	-0.60580	-0.17900
H	1.81409	-2.13785	1.39360
H	0.96881	-3.03124	0.13489
H	3.25566	-1.65526	-0.50925
H	1.87985	-1.60245	-1.62047
H	2.29684	0.73985	-1.28446
H	2.60135	0.33054	1.48442
H	-0.63646	0.46077	-2.03866

H	-0.81740	-1.90042	-1.14555
H	-0.85112	3.26888	-0.97033
H	0.62653	3.28255	0.00961
H	0.61819	2.48227	-1.56893
H	-1.63773	0.73495	1.71847
H	-0.72147	2.23151	1.97566
H	-2.17343	2.23504	0.96093
H	-2.63135	-2.33603	0.57364
H	-4.54194	-0.88025	0.15269
H	-3.44504	0.34201	-0.70354

Conformer 2
Energy: -543.23396 Hartree (Rel: 0.2 kcal/mol)
XYZ coordinates for conf 2:

C	-0.61833	0.20011	0.03440
C	0.30102	-0.95976	-0.43552
C	-0.58238	-2.21983	-0.28845
C	-1.80267	-1.78325	0.56368
C	-2.03402	-0.29165	0.22014
O	-2.85189	-0.11036	-0.94674
C	0.40534	0.57775	1.12247
C	1.29202	-0.62193	0.72520
C	0.01686	1.55985	0.02527
C	-0.86867	2.69593	0.52905
C	0.95655	2.01091	-1.08400
H	0.72990	-0.87698	-1.43918
C	2.76162	-0.42102	0.47666
C	3.46344	-0.90993	-0.54878
H	-0.91939	-2.54310	-1.28182
H	-0.04635	-3.06731	0.15332
H	-2.69768	-2.38448	0.37371
H	-1.56720	-1.86569	1.63235
H	-2.57369	0.22750	1.01695
H	-2.34130	-0.41563	-1.71390
H	0.18297	0.83100	2.15765
H	1.18419	-1.38214	1.51430
H	-0.25405	3.53048	0.88883
H	-1.50901	3.07334	-0.27738
H	-1.51901	2.38085	1.35162
H	1.61271	1.21336	-1.43731
H	0.38045	2.38512	-1.94057
H	1.59490	2.83134	-0.73153
H	3.27985	0.16213	1.24096
H	4.53304	-0.73864	-0.63927
H	3.00008	-1.50134	-1.33558

Conformer 3
Energy: -543.23659 Hartree (Rel: 1.6 kcal/mol)
XYZ coordinates for conf 3:

C	0.56937	0.09967	-0.08483
C	-0.02882	-1.20151	0.48776
C	1.03902	-2.24974	0.10923
C	2.36971	-1.47057	0.15699
C	2.05986	-0.05198	-0.37451
O	2.90410	0.86999	0.32546
C	-0.66793	0.26652	-0.99740
C	-1.26099	-1.05316	-0.47710
C	-0.30433	1.31129	0.05017
C	0.25225	2.59030	-0.57274
C	-1.09910	1.59366	1.31710
H	-0.25532	-1.21832	1.56088
C	-2.66741	-1.14053	0.04471
C	-3.63162	-0.23701	-0.14236
H	1.04327	-3.12235	0.77106
H	0.86031	-2.61575	-0.91056
H	2.69753	-1.35000	1.19734
H	3.18032	-1.96269	-0.39032
H	2.26291	0.01517	-1.45409
H	2.75637	1.74869	-0.05660
H	-0.67479	0.55492	-2.04732
H	-1.15645	-1.81964	-1.25924

```
H         -0.56359        3.26948       -0.84952
H          0.89270        3.12672        0.13900
H          0.83498        2.39186       -1.47998
H         -1.51017        0.69025        1.77079
H         -0.45471        2.08429        2.05851
H         -1.93938        2.26747        1.10723
H         -2.90221       -2.06261        0.58121
H         -4.64109       -0.39239        0.22968
H         -3.44358        0.69335       -0.67380
```

```
Conformer 4
Energy: -543.23635 Hartree (Rel:  1.7 kcal/mol)
XYZ coordinates for conf 4:
C         -0.58646        0.05302        0.10870
C          0.42500       -1.03815       -0.29863
C         -0.33788       -2.33490        0.04822
C         -1.81862       -1.98464       -0.20479
C         -1.99741       -0.51343        0.23611
O         -2.96334        0.08773       -0.63514
C          0.42094        0.59730        1.14680
C          1.43674       -0.50102        0.76797
C         -0.08647        1.46646        0.00838
C         -1.06697        2.53749        0.48340
C          0.76844        1.95041       -1.15396
H          0.77041       -1.03527       -1.33916
C          2.84867       -0.12640        0.40889
C          3.56047       -0.62020       -0.60725
H         -0.00804       -3.19983       -0.53709
H         -0.19161       -2.58436        1.10739
H         -2.03362       -2.01644       -1.28039
H         -2.51654       -2.66614        0.29239
H         -2.35700       -0.45513        1.27457
H         -3.11743        0.99141       -0.31959
H          0.21412        0.89190        2.17457
H          1.48608       -1.21589        1.60249
H         -0.52576        3.42969        0.82190
H         -1.73074        2.85219       -0.33219
H         -1.68676        2.19348        1.31983
H          1.47407        1.19554       -1.50458
H          0.12806        2.23681       -1.99851
H          1.34834        2.83592       -0.86231
H          3.32011        0.59125        1.08370
H          4.59176       -0.32193       -0.77782
H          3.14260       -1.34078       -1.30714
```

Revised structure of 35{2n'}, i.e. 36{2n'-rev} ($CDCl_3$)

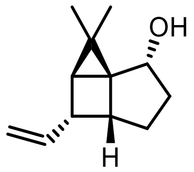

	Conf1	Conf2
Rel energy (kcal/mol):	0.00	2.33

C-nom	iGau	Exp	Calc	diff	1	2
C	13	138.40	138.01	-0.39	[138.01	137.92]
C	14	114.40	112.36	-2.04	[112.31	114.96]
C	5	69.70	70.09	0.39	[70.08	70.55]
C	1	47.50	44.90	-2.60	[44.90	45.05]
C	4	38.80	38.56	-0.24	[38.56	38.31]
C	2	37.90	38.49	0.59	[38.51	37.51]
C	8	34.90	34.75	-0.15	[34.74	35.00]
C	9	26.10	26.18	0.08	[26.18	26.15]
C	3	25.50	25.79	0.29	[25.74	28.14]
C	7	24.80	25.02	0.22	[24.95	28.33]
C	10	21.10	20.50	-0.60	[20.50	20.30]
C	11	15.30	14.79	-0.51	[14.80	14.33]

13C chem shifts: RMSD=1.02ppm (MAE=0.68) N=12 {-2.60 0.59}

Fractions: 0.981 0.019

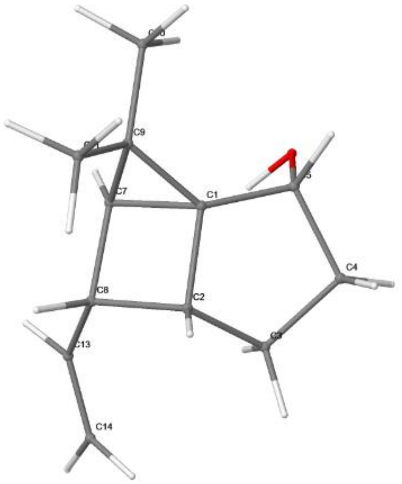

Conformer 1
Energy: -543.23822 Hartree (Rel: 0.0 kcal/mol)
XYZ coordinates for conf 1:

C	0.59087	0.29841	-0.23127
C	-0.54718	0.02535	-1.23461
C	-1.42186	1.28630	-1.08096
C	-0.39456	2.40393	-0.78942
C	0.64978	1.76919	0.16559
O	0.36342	1.99656	1.55109
C	0.16697	-0.94256	0.58605
C	-0.93615	-1.27925	-0.43128
C	1.55699	-0.83204	-0.01777
C	2.64124	-0.54716	1.01642
C	2.04050	-1.71736	-1.15696
H	-0.26261	-0.13765	-2.28190
C	-2.35161	-1.36530	0.05472
C	-2.77307	-1.34942	1.32235
H	-2.03667	1.49318	-1.96298
H	-2.10329	1.17476	-0.22892
H	0.10783	2.69403	-1.72208
H	-0.83460	3.30688	-0.35388

```
H          1.64075        2.21092        0.01932
H         -0.38777        1.42456        1.78016
H          0.06509       -1.02281        1.66573
H         -0.72898       -2.18934       -1.01273
H          2.99877       -1.47912        1.47188
H          3.50245       -0.05477        0.54729
H          2.27401        0.10846        1.81245
H          1.27194       -1.90287       -1.91265
H          2.89430       -1.25036       -1.66415
H          2.37478       -2.69019       -0.77422
H         -3.09449       -1.45928       -0.73979
H         -3.82911       -1.43217        1.56573
H         -2.09114       -1.26714        2.16468

Conformer 2
Energy: -543.23451 Hartree (Rel:  2.3 kcal/mol)
XYZ coordinates for conf 2:
C          0.62927        0.21535       -0.24060
C         -0.70818       -0.03225       -0.97631
C         -1.41616        1.32669       -0.79056
C         -0.25778        2.34634       -0.85220
C          0.89664        1.70287       -0.04241
O          0.91603        2.10295        1.33391
C          0.26913       -0.88881        0.77504
C         -1.02507       -1.22222        0.00225
C          1.52811       -0.97832       -0.06846
C          2.80459       -0.69391        0.71635
C          1.72275       -2.01754       -1.16265
H         -0.65016       -0.30249       -2.03891
C         -2.29040       -1.24620        0.81714
C         -3.50533       -0.85179        0.42865
H         -2.19042        1.51610       -1.54134
H         -1.90033        1.37470        0.19084
H          0.06646        2.47408       -1.89386
H         -0.51761        3.33501       -0.46017
H          1.87344        2.01428       -0.42633
H          0.18025        1.64384        1.77136
H          0.33857       -0.84764        1.86132
H         -0.95179       -2.19549       -0.50545
H          3.17116       -1.60551        1.20467
H          3.59711       -0.33322        0.04838
H          2.64180        0.06893        1.48420
H          0.81914       -2.19391       -1.75308
H          2.50890       -1.69329       -1.85622
H          2.03583       -2.97810       -0.73359
H         -2.18011       -1.69030        1.80896
H         -4.36970       -0.96281        1.07834
H         -3.68614       -0.40906       -0.54785
```

Originally assigned correct structure of 40{12j} ($CDCl_3$)

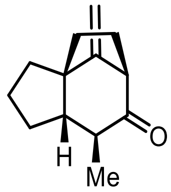

	Conf1	Conf2	Conf3
Rel energy (kcal/mol):	0.00	0.44	2.08

C-nom	iGau	Exp	Calc	diff	1	2	3
C	5	216.50	215.88	-0.62	[215.76	216.21	214.53]
C	1	152.80	153.14	0.34	[153.28	152.91	152.32]
C	12	103.00	102.27	-0.73	[102.33	102.00	104.45]
C	6	56.10	57.12	1.02	[56.86	57.50	59.84]
C	2	53.50	53.40	-0.10	[52.99	54.23	53.70]
C	3	52.20	49.75	-2.45	[50.27	48.53	51.94]
C	4	46.90	47.54	0.64	[47.57	47.65	44.96]
C	10	38.30	38.65	0.35	[39.69	36.76	34.07]
C	9	34.90	34.25	-0.65	[34.92	32.91	32.97]
C	7	32.10	32.19	0.09	[31.58	33.54	31.09]
C	11	26.20	26.47	0.27	[26.32	26.90	24.81]
C	8	26.20	26.57	0.37	[26.90	26.20	21.67]
C	16	13.60	13.59	-0.01	[13.19	13.82	23.16]

13C chem shifts: RMSD=0.84ppm (MAE=0.59) N=13 {-2.45 1.02}

Fractions: 0.664 0.316 0.020

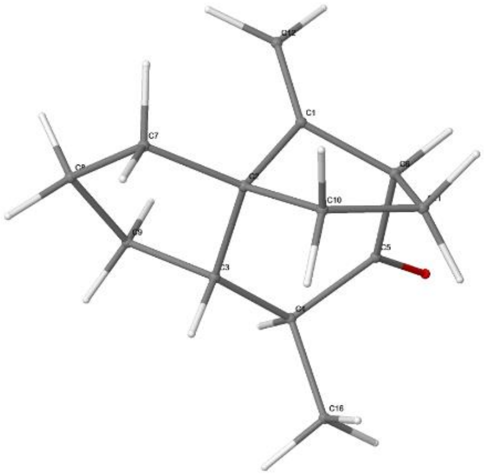

Conformer 1

Energy: -581.41970 Hartree (Rel: 0.0 kcal/mol)

XYZ coordinates for conf 1:

C	-0.06161	-1.29708	0.80585
C	-0.94512	-0.55989	-0.18870
C	-0.42205	0.93883	-0.32413
C	0.92059	1.21407	0.39247
C	1.90891	0.06827	0.15773
C	1.28691	-1.33324	0.12079
C	-2.45407	-0.43781	0.12846
C	-2.84094	1.00497	-0.24269
C	-1.59819	1.81030	0.16103
C	-0.60165	-1.35139	-1.48002
C	0.91557	-1.67203	-1.36668
C	-0.36623	-1.84445	1.98229
O	3.10594	0.23953	0.00471
H	-0.27211	1.14027	-1.39432
H	0.71813	1.17027	1.47604
C	1.52617	2.57916	0.06003

H	2.00292	-2.04099	0.54612
H	-2.62800	-0.58607	1.20105
H	-3.04738	-1.19268	-0.39881
H	-3.00670	1.09113	-1.32487
H	-3.75574	1.34287	0.25637
H	-1.57848	2.81996	-0.26298
H	-1.56088	1.91211	1.25525
H	-0.85098	-0.79234	-2.38868
H	-1.18112	-2.28165	-1.49714
H	1.11648	-2.72869	-1.56526
H	1.52310	-1.09460	-2.07100
H	0.38173	-2.37457	2.56728
H	-1.36527	-1.79432	2.40693
H	2.48094	2.71509	0.57526
H	1.71366	2.67110	-1.01637
H	0.85241	3.38845	0.35876

Conformer 2
Energy: -581.42301 Hartree (Rel: 0.4 kcal/mol)
XYZ coordinates for conf 2:

C	-0.26037	-1.19176	0.79598
C	-0.92866	-0.52498	-0.39914
C	-0.35169	0.94961	-0.53986
C	0.91640	1.21715	0.31405
C	1.85809	0.01152	0.31617
C	1.17294	-1.35975	0.34804
C	-2.45132	-0.33576	-0.32336
C	-2.64241	1.00010	0.40800
C	-1.53915	1.89670	-0.18799
C	-0.42090	-1.44006	-1.54278
C	1.01546	-1.86974	-1.12650
C	-0.78740	-1.60551	1.94846
O	3.07258	0.11423	0.29093
H	-0.06857	1.10215	-1.58908
H	0.58394	1.29436	1.36332
C	1.64293	2.50775	-0.07034
H	1.77507	-2.03407	0.96281
H	-2.95282	-1.18174	0.15985
H	-2.85613	-0.25972	-1.34200
H	-3.64475	1.42262	0.27802
H	-2.48213	0.86495	1.48440
H	-1.90805	2.37958	-1.10052
H	-1.24137	2.69940	0.49563
H	-0.44863	-0.93167	-2.51306
H	-1.07077	-2.31984	-1.61684
H	1.12842	-2.95751	-1.14978
H	1.78964	-1.45321	-1.77860
H	-0.18166	-2.11067	2.69726
H	-1.83724	-1.46283	2.19069
H	2.51162	2.67171	0.57308
H	2.00107	2.45798	-1.10531
H	0.97823	3.37305	0.01834

Conformer 3
Energy: -581.42231 Hartree (Rel: 2.1 kcal/mol)
XYZ coordinates for conf 3:

C	0.35825	-1.26308	-0.54511
C	0.90268	-0.35208	0.54138
C	0.42177	1.10404	0.21348
C	-1.05289	1.26829	-0.24980
C	-1.76230	-0.03670	-0.65528
C	-1.12131	-1.35734	-0.23568
C	2.43289	-0.19615	0.61246
C	2.80981	0.97996	-0.33536
C	1.46590	1.58119	-0.81398
C	0.22095	-0.93337	1.80438
C	-1.14281	-1.50591	1.32055
C	0.99626	-1.86545	-1.54971
O	-2.80851	-0.00290	-1.28105
H	0.56051	1.68815	1.13477
H	-1.06881	1.88000	-1.16163

C	-1.92576	2.00986	0.78835
H	-1.64380	-2.16538	-0.75303
H	2.94410	-1.13121	0.35894
H	2.71633	0.04507	1.64389
H	3.40342	1.72730	0.20221
H	3.41879	0.64495	-1.18146
H	1.49139	2.67352	-0.89728
H	1.20412	1.18741	-1.80367
H	0.11041	-0.17440	2.58713
H	0.84044	-1.73672	2.21935
H	-1.23746	-2.56626	1.57281
H	-2.00127	-0.99639	1.76980
H	0.46739	-2.49959	-2.25729
H	2.06704	-1.75651	-1.70121
H	-2.95089	2.11541	0.42092
H	-1.51907	3.00997	0.97367
H	-1.95514	1.47846	1.74633

Originally assigned (incorrect) structure of 41{14j} ($CDCl_3$)

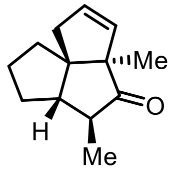

					Conf1	Conf2
		Rel energy (kcal/mol):			0.00	0.51

C-nom	iGau	Exp	Calc	diff	1	2
C	8	220.90	221.34	0.44	[221.04	222.04]
C	2	136.30	135.70	-0.60	[135.60	135.94]
C	3	131.90	130.44	-1.46	[130.22	130.95]
C	1	63.90	66.05	2.15	[65.72	66.82]
C	5	56.90	58.40	1.50	[58.72	57.65]
C	6	52.30	52.47	0.17	[51.65	54.38]
C	7	49.60	51.56	1.96	[52.97	48.26]
C	4	42.90	47.76	4.86	[46.38	50.99]
C	9	36.10	33.75	-2.35	[34.00	33.15]
C	11	29.00	28.62	-0.38	[28.59	28.69]
C	10	25.60	27.38	1.78	[28.01	25.91]
C	13	16.60	19.31	2.71	[19.14	19.71]
C	14	10.20	13.75	3.55	[13.21	15.02]

13C chem shifts: RMSD=2.25ppm (MAE=1.84) N=13 {-2.35 4.86}
Fractions: 0.701 0.299

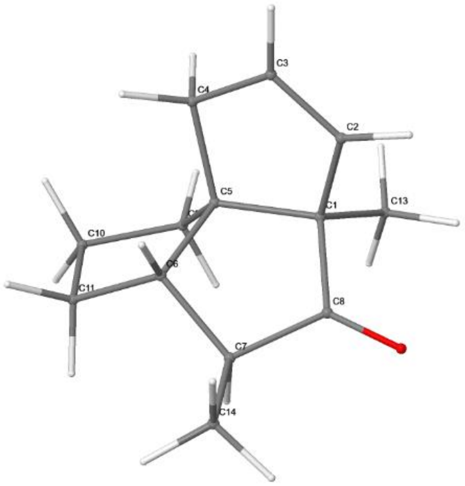

Conformer 1
Energy: -581.42904 Hartree (Rel: 0.0 kcal/mol)
XYZ coordinates for conf 1:

C	0.94380	-0.75067	0.33884
C	1.48449	-1.48367	-0.88140
C	0.51325	-2.01612	-1.62677
C	-0.85633	-1.73437	-1.06075
C	-0.58721	-0.64753	0.01967
C	-0.79759	0.79701	-0.56263
C	0.31350	1.66693	0.05810
C	1.46142	0.69824	0.33120
C	-1.58585	-0.68969	1.19255
C	-2.82673	0.03215	0.63871
C	-2.24945	1.21005	-0.18749
O	2.62247	1.01402	0.51687
C	1.35835	-1.46589	1.63839
C	0.72786	2.91197	-0.72630
H	0.67081	-2.61218	-2.52193

H	2.55262	-1.56550	-1.06258
H	-1.56850	-1.39476	-1.82448
H	-1.28990	-2.64645	-0.62228
H	-0.65507	0.78535	-1.65031
H	-0.01666	1.98266	1.06337
H	-1.79667	-1.70784	1.53842
H	-1.19097	-0.13175	2.05243
H	-3.51308	0.36324	1.42556
H	-3.38971	-0.64767	-0.01285
H	-2.85988	1.42212	-1.07121
H	-2.23690	2.12930	0.41104
H	1.01223	-2.50487	1.63507
H	2.44965	-1.46538	1.72870
H	0.94864	-0.96837	2.52453
H	1.02850	2.64589	-1.74660
H	1.57654	3.40546	-0.24221
H	-0.09850	3.62809	-0.79220

Conformer 2
Energy: -581.42823 Hartree (Rel: 0.5 kcal/mol)
XYZ coordinates for conf 2:

C	1.10973	-0.27547	0.41220
C	2.07029	-0.56099	-0.73723
C	1.57659	-1.43735	-1.61425
C	0.20436	-1.91300	-1.21633
C	-0.23403	-0.92060	-0.10113
C	-1.06104	0.28324	-0.70135
C	-0.53135	1.57577	-0.04597
C	0.86946	1.24045	0.45231
C	-1.19281	-1.57711	0.94174
C	-2.44111	-0.67556	1.03824
C	-2.52775	-0.02629	-0.35306
O	1.69510	2.05513	0.82413
C	1.69598	-0.76365	1.74828
C	-0.58201	2.83715	-0.91260
H	2.10452	-1.81005	-2.48812
H	3.05148	-0.09564	-0.77395
H	-0.51111	-1.92793	-2.05023
H	0.25434	-2.94640	-0.84064
H	-0.90545	0.35166	-1.78489
H	-1.10539	1.77149	0.87522
H	-1.49127	-2.56631	0.57402
H	-0.72040	-1.74086	1.91473
H	-2.30279	0.09400	1.80863
H	-3.34433	-1.23399	1.30705
H	-2.93898	-0.74614	-1.07319
H	-3.16813	0.86357	-0.38106
H	1.88199	-1.84260	1.72214
H	2.64533	-0.25298	1.93926
H	1.02887	-0.54719	2.59031
H	-0.03731	2.68604	-1.85202
H	-0.12409	3.68071	-0.38659
H	-1.61606	3.10208	-1.15877

Revised structure of 41{14j}, i.e. 42{14j-rev} ($CDCl_3$)

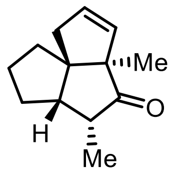

	Conf1	Conf2
Rel energy (kcal/mol):	0.00	0.05

C-nom	iGau	Exp	Calc	diff	1	2
C	8	220.90	219.86	-1.04	[219.75	219.98]
C	2	136.30	136.78	0.48	[136.58	137.00]
C	3	131.90	132.69	0.79	[132.76	132.62]
C	1	63.90	64.83	0.93	[65.48	64.11]
C	5	56.90	55.81	-1.09	[54.92	56.79]
C	6	52.30	51.64	-0.66	[52.83	50.33]
C	4	49.60	51.05	1.45	[53.35	48.52]
C	7	42.90	43.83	0.93	[43.48	44.21]
C	9	36.10	36.97	0.87	[37.59	36.30]
C	11	29.00	27.35	-1.65	[29.44	25.06]
C	10	25.60	26.92	1.32	[27.02	26.81]
C	13	16.60	16.97	0.37	[17.41	16.49]
C	14	10.20	10.23	0.03	[9.89	10.60]

13C chem shifts: RMSD=0.99ppm (MAE=0.89) N=13 {-1.65 1.45}

Fractions: 0.523 0.477

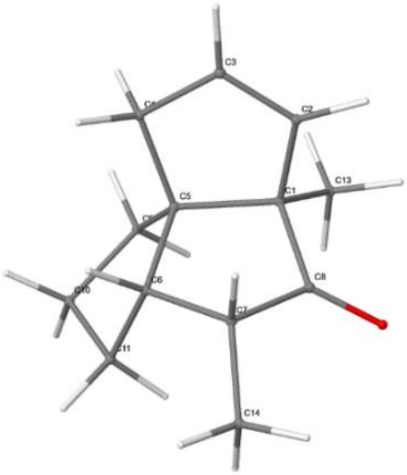

Conformer 1
Energy: -581.42638 Hartree (Rel: 0.0 kcal/mol)
XYZ coordinates for conf 1:

C	-0.95726	-0.60696	0.44240
C	-1.96567	-1.07142	-0.60213
C	-2.28540	-0.11678	-1.47767
C	-1.56756	1.17621	-1.19750
C	-0.51057	0.81120	-0.10930
C	0.93576	0.58058	-0.73015
C	1.18012	-0.94362	-0.79848
C	0.29578	-1.48994	0.31660
C	-0.30391	1.93512	0.94877
C	1.04533	2.58756	0.60333
C	1.89238	1.38337	0.17569
O	0.53605	-2.46530	1.00385
C	-1.56000	-0.69348	1.85212
C	2.62347	-1.44337	-0.75922
H	-3.00935	-0.22728	-2.28098
H	-2.37772	-2.07716	-0.57639
H	-1.10221	1.60477	-2.09539

```
H         -2.27772        1.93507       -0.83627
H          0.97709        1.01282       -1.73649
H          0.71349       -1.30961       -1.72633
H         -1.13552        2.64757        0.96290
H         -0.23640        1.51048        1.95612
H          1.47579        3.14664        1.44157
H          0.93330        3.28626       -0.23687
H          2.82439        1.65516       -0.33178
H          2.16512        0.79955        1.06634
H         -2.41870       -0.02148        1.95163
H         -1.89888       -1.71622        2.04579
H         -0.82886       -0.44379        2.62772
H          3.14085       -1.11632        0.14829
H          2.64440       -2.53774       -0.77364
H          3.18769       -1.07709       -1.62382

Conformer 2
Energy: -581.42629 Hartree (Rel:  0.1 kcal/mol)
XYZ coordinates for conf 2:
C         -0.87390       -0.60863        0.47530
C         -1.88481       -1.25637       -0.46585
C         -2.37858       -0.40134       -1.36394
C         -1.80313        0.98092       -1.20475
C         -0.62159        0.79850       -0.21121
C          0.79806        0.67595       -0.92737
C          1.21782       -0.81849       -0.85291
C          0.45648       -1.36120        0.34666
C         -0.45041        1.98464        0.76366
C          1.03145        1.97378        1.15467
C          1.72327        1.68201       -0.18657
O          0.83620       -2.24760        1.09020
C         -1.38967       -0.63360        1.92147
C          2.70475       -1.17084       -0.85104
H         -3.14039       -0.64436       -2.10022
H         -2.17159       -2.29893       -0.35218
H         -1.47251        1.42255       -2.15447
H         -2.56431        1.66704       -0.80173
H          0.71938        0.96621       -1.97949
H          0.75290       -1.31420       -1.71949
H         -0.67104        2.91676        0.22466
H         -1.13734        1.94126        1.61469
H          1.23369        1.17064        1.87704
H          1.36509        2.91156        1.61265
H          1.77432        2.61008       -0.76963
H          2.75120        1.32625       -0.07404
H         -2.35684       -0.12713        2.00218
H         -1.51585       -1.67083        2.24771
H         -0.68745       -0.15696        2.61364
H          3.20816       -0.81389        0.05296
H          2.83155       -2.25750       -0.88421
H          3.21308       -0.73969       -1.72042
```

Originally assigned (incorrect) structure of 43{12m} ($CDCl_3$)

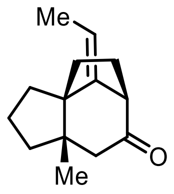

					Conf1
Rel energy (kcal/mol):					0.00
**C-nom**	**iGau**	**Exp**	**Calc**	**diff**	**1**
C	5	210.90	209.08	-1.82	[209.08]
C	1	144.60	143.07	-1.53	[143.07]
C	12	115.60	119.24	3.64	[119.24]
C	6	56.90	62.72	5.82	[62.72]
C	2	52.90	57.17	4.27	[57.17]
C	4	47.90	47.12	-0.78	[47.12]
C	3	44.90	46.02	1.12	[46.02]
C	9	38.80	38.91	0.11	[38.91]
C	10	28.90	31.79	2.89	[31.79]
C	7	28.50	29.38	0.88	[29.38]
C	14	24.90	23.57	-1.33	[23.57]
C	11	24.50	23.31	-1.19	[23.31]
C	8	21.10	22.41	1.31	[22.41]
C	15	13.70	12.85	-0.85	[12.85]

13C chem shifts: RMSD=2.50ppm (MAE=1.97) N=14 {-1.82 5.82}

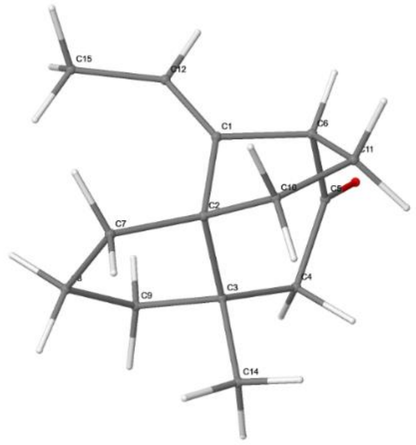

Conformer 1
Energy: -620.72859 Hartree (Rel: 0.0 kcal/mol)
XYZ coordinates for conf 1:

C	0.15905	1.14500	0.31032
C	-0.50705	-0.17059	0.72402
C	-0.21566	-1.24603	-0.41108
C	1.22103	-1.18549	-1.00633
C	2.02167	0.09040	-0.76338
C	1.64682	0.85917	0.49453
C	-2.04568	-0.19713	0.82305
C	-2.57215	-0.56527	-0.59532
C	-1.30911	-0.86574	-1.43911
C	0.24311	-0.44265	2.04964
C	1.71086	-0.01802	1.78291
C	-0.27014	2.34124	-0.11856
O	2.89164	0.46263	-1.53362
C	-0.46603	-2.69077	0.07402
C	-1.64676	2.87472	-0.40737
H	1.82933	-1.98884	-0.56433
H	1.21370	-1.38189	-2.08426

H	2.26516	1.75832	0.54591
H	-2.34105	-0.96597	1.54602
H	-2.44379	0.74592	1.20657
H	-3.16678	0.23757	-1.04237
H	-3.22746	-1.44121	-0.54402
H	-1.46864	-1.65957	-2.17802
H	-0.99806	0.03081	-1.98787
H	-0.19335	0.18695	2.83461
H	0.15037	-1.47950	2.38543
H	2.11932	0.56777	2.61153
H	2.37010	-0.88342	1.65334
H	0.51628	3.07867	-0.29387
H	-1.47299	-2.83282	0.47889
H	-0.35054	-3.38306	-0.76898
H	0.25115	-2.99508	0.84400
H	-2.44078	2.13342	-0.33330
H	-1.88876	3.70049	0.27628
H	-1.67600	3.29743	-1.42061

Revised structure of 43{12m}, i.e. 44{12m-rev} ($CDCl_3$)

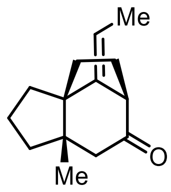

					**Conf1**
	Rel energy (kcal/mol):				0.00

C-nom	iGau	Exp	Calc	diff	1
C	5	210.90	208.89	-2.01	[208.89]
C	1	144.60	144.58	-0.02	[144.58]
C	12	115.60	115.48	-0.12	[115.48]
C-C	2	56.90	57.29	0.39	[57.29]
C-CH	6	52.90	54.46	1.56	[54.46]
C-CH2	4	47.90	46.94	-0.96	[46.94]
C-C	3	44.90	45.88	0.98	[45.88]
C	9	38.80	37.39	-1.41	[37.39]
C	10	28.90	29.10	0.20	[29.10]
C	7	28.50	29.05	0.55	[29.05]
C	11	24.90	24.86	-0.04	[24.86]
C	14	24.50	23.96	-0.54	[23.96]
C	8	21.10	21.45	0.35	[21.45]
C	15	13.70	13.60	-0.10	[13.60]

13C chem shifts: RMSD=0.90ppm (MAE=0.66) N=14 {-2.01 1.56}

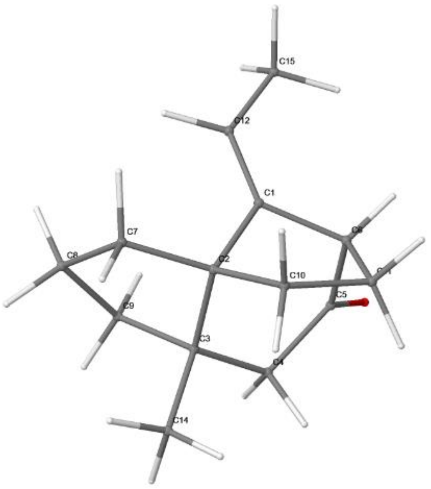

Conformer 1
Energy: -620.73471 Hartree (Rel: 0.0 kcal/mol)
XYZ coordinates for conf 1:

C	0.81810	0.68038	0.35377
C	-0.62675	0.33980	0.71692
C	-1.21838	-0.57118	-0.43990
C	-0.23859	-1.67175	-0.93804
C	1.24671	-1.43137	-0.68611
C	1.56679	-0.62462	0.56735
C	-1.59168	1.53809	0.73188
C	-2.06309	1.72751	-0.73987
C	-1.52557	0.49793	-1.51833
C	-0.40698	-0.40271	2.05415
C	0.90324	-1.21511	1.85338
C	1.32080	1.85227	-0.05232
O	2.11012	-1.84991	-1.43926
C	-2.53828	-1.25799	-0.02950
C	2.75258	2.15505	-0.39889

H	-0.46792	-2.61661	-0.42225
H	-0.37671	-1.87431	-2.00583
H	2.65068	-0.52928	0.64857
H	-2.44355	1.31560	1.38406
H	-1.11344	2.43227	1.14509
H	-1.68395	2.66012	-1.17032
H	-3.15560	1.78251	-0.79151
H	-2.23540	0.12497	-2.26554
H	-0.60462	0.76057	-2.04994
H	-0.27045	0.33792	2.85121
H	-1.25528	-1.02955	2.34374
H	1.58282	-1.09575	2.70226
H	0.71111	-2.28878	1.75257
H	0.63900	2.69715	-0.14814
H	-3.30388	-0.54017	0.28244
H	-2.94090	-1.81666	-0.88333
H	-2.39445	-1.97296	0.78781
H	2.83961	2.47333	-1.44663
H	3.13442	2.98515	0.21081
H	3.41411	1.29609	-0.25795

Originally assigned (incorrect) structure of 46{4m} ($CDCl_3$)

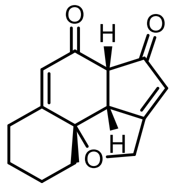

					Conf1
	Rel energy (kcal/mol):				0.00
**C-nom**	**iGau**	**Exp**	**Calc**	**diff**	**1**
C	9	200.50	200.01	-0.49	[200.01]
C	6	189.30	187.19	-2.11	[187.19]
C	7	188.70	183.38	-5.32	[183.38]
C	2	153.40	163.24	9.84	[163.24]
C	8	126.70	123.16	-3.54	[123.16]
C	1	121.20	121.96	0.76	[121.96]
C	3	78.00	80.06	2.06	[80.06]
C	11	66.40	65.58	-0.82	[65.58]
C	5	65.10	60.49	-4.61	[60.49]
C	4	52.50	52.93	0.43	[52.93]
C	12	34.80	41.54	6.74	[41.54]
C	18	33.00	33.75	0.75	[33.75]
C	27	23.20	29.13	5.93	[29.13]
C	23	20.50	22.76	2.26	[22.76]

13C chem shifts: RMSD=4.27ppm (MAE=3.26) N=14 {-5.32 9.84}

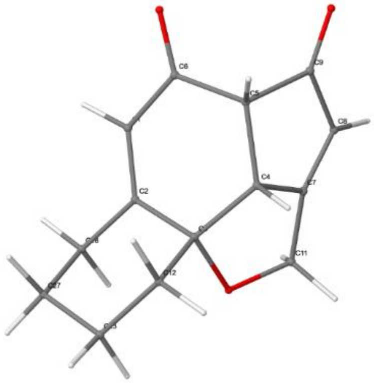

Conformer 1
Energy: -767.54982 Hartree (Rel: 0.0 kcal/mol)
XYZ coordinates for conf 1:

C	-0.03265	1.63928	-0.75280
C	-0.99981	0.70800	-0.62418
C	-0.87540	-0.46358	0.33550
C	0.56783	-0.69934	0.80360
C	1.61185	0.42491	0.83716
C	1.23628	1.65189	-0.00977
C	1.09302	-1.63104	-0.26239
C	2.38155	-1.42094	-0.56815
C	2.86755	-0.24196	0.18666
O	-1.22441	-1.70852	-0.34505
C	-0.05721	-2.40539	-0.81372
C	-1.91562	-0.27736	1.45500
H	0.54210	-1.22550	1.76779
H	1.86865	0.78203	1.83770
O	1.99050	2.61573	-0.05279
O	4.00335	0.18381	0.25597
H	-0.18527	2.50517	-1.39402
C	-2.35442	0.83735	-1.26399
H	2.99710	-1.95439	-1.28329
H	-0.09514	-3.43806	-0.44223

H	-0.04072	-2.43851	-1.91069
H	-1.64294	0.61569	2.03313
C	-3.33671	-0.12745	0.88577
H	-1.85608	-1.13947	2.13030
H	-3.63339	-1.07621	0.42324
H	-4.04071	0.06262	1.70477
C	-3.42366	1.00073	-0.15393
H	-2.57232	-0.07365	-1.83543
H	-2.37887	1.68654	-1.95461
H	-3.27291	1.97072	0.33888
H	-4.42070	1.02412	-0.60920

Revised structure of 46{4m}, i.e 47{4m-rev} ($CDCl_3$)

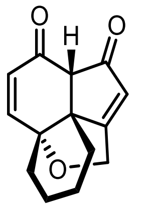

C-nom	iGau	Exp	Calc	diff	Conf1 (Rel energy (kcal/mol): 0.00) 1
C	9	200.50	201.03	0.53	[201.03]
C	7	189.30	189.75	0.45	[189.75]
C	6	188.70	189.44	0.74	[189.44]
C	4	153.40	151.73	-1.67	[151.73]
C	5	126.70	126.00	-0.70	[126.00]
C	8	121.20	120.77	-0.43	[120.77]
C	3	78.00	79.65	1.65	[79.65]
C	1	66.40	67.37	0.97	[67.37]
C	11	65.10	65.13	0.03	[65.13]
C	2	52.50	50.85	-1.65	[50.85]
C	18	34.80	35.37	0.57	[35.37]
C	12	33.00	32.79	-0.21	[32.79]
C	27	23.20	22.31	-0.89	[22.31]
C	23	20.50	20.39	-0.11	[20.39]

13C chem shifts: RMSD=0.93ppm (MAE=0.76) N=14 {-1.67 1.65}

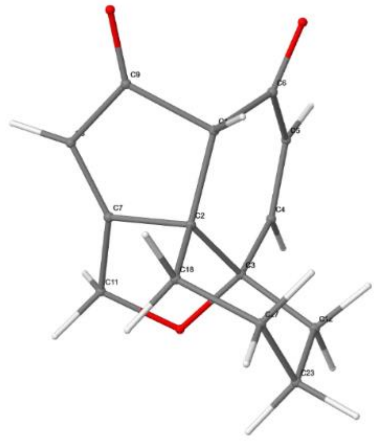

Conformer 1
Energy: -767.54182 Hartree (Rel: 0.0 kcal/mol)
XYZ coordinates for conf 1:

C	-1.12994	0.17791	0.78243
C	0.19250	-0.40610	0.24795
C	1.09681	0.45316	-0.67355
C	0.27892	1.40621	-1.51272
C	-0.92838	1.87137	-1.15611
C	-1.61116	1.45759	0.08692
C	-0.27545	-1.44132	-0.75274
C	-1.55683	-1.80350	-0.59498
C	-2.17038	-0.94896	0.44670
O	1.71954	-0.54011	-1.53452
C	0.81395	-1.63697	-1.76030
C	2.22239	1.24090	0.02250
H	-1.44757	2.62035	-1.74853
H	0.75980	1.75608	-2.42492
O	-2.55855	2.08616	0.53790
O	-3.28633	-1.03158	0.91985

H	-1.14087	0.36561	1.85924
C	1.01294	-1.11630	1.36621
H	-2.12177	-2.54131	-1.15313
H	0.42129	-1.61419	-2.78529
H	1.36748	-2.57481	-1.62922
H	1.77125	2.12558	0.48919
C	2.97035	0.43267	1.08893
H	2.90904	1.60577	-0.75066
H	3.54933	-0.36704	0.61020
H	3.68659	1.08772	1.59871
C	1.98364	-0.17780	2.09055
H	0.30799	-1.58165	2.06505
H	1.59501	-1.93403	0.92301
H	2.51855	-0.73908	2.86591
H	1.43039	0.62197	2.60385

Originally assigned (incorrect) structure of 48{4} (Acetone-d_6)

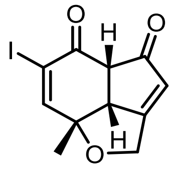

					Conf1
		Rel energy (kcal/mol):			0.00

**C-nom**	**iGau**	**Exp**	**Calc**	**diff**	**1**
C	9	196.80	198.96	2.16	[199.22]
C	7	189.70	184.86	-4.84	[185.04]
C	6	187.80	182.90	-4.90	[183.07]
C	2	153.30	160.25	6.95	[160.28]
C	8	128.50	123.44	-5.06	[123.25]
C	1	92.10	100.26	8.16	[99.93]
C	3	78.90	81.41	2.51	[80.97]
C	11	69.40	67.43	-1.97	[66.91]
C	5	59.00	61.42	2.42	[60.86]
C	4	55.90	53.04	-2.86	[52.43]
C	12	27.50	26.00	-1.50	[25.23]

13C chem shifts: CRMSD=4.46ppm (CMAE=3.94) N=11 {-5.06 8.16}

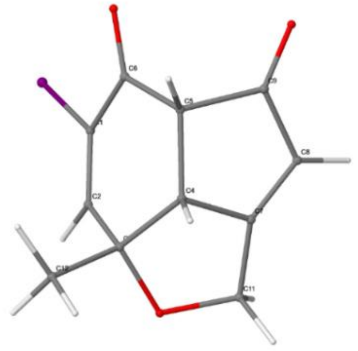

Conformer 1
Energy: -7570.06990 Hartree (Rel: 0.0 kcal/mol)
XYZ coordinates for conf 1:

C	-0.60977	-0.23808	0.24025
C	0.11212	-1.35001	0.04119
C	1.55728	-1.49884	0.47692
C	2.23976	-0.12929	0.60888
C	1.44558	1.12942	0.97181
C	-0.07798	0.99738	0.87748
C	2.68407	0.11696	-0.81146
C	2.55845	1.39502	-1.19898
C	1.90254	2.16519	-0.12126
O	2.30229	-2.16156	-0.57728
C	2.94001	-1.20699	-1.45192
C	1.64009	-2.39177	1.71588
O	1.67938	3.35850	-0.07854
O	-0.79461	1.89669	1.27982
I	-2.69121	-0.17031	-0.29852
H	-0.32720	-2.23351	-0.41358
H	3.11674	-0.23048	1.26075
H	1.66875	1.53615	1.96079
H	2.80911	1.83111	-2.15884
H	4.00788	-1.45205	-1.51314
H	2.51421	-1.26609	-2.46062
H	1.11445	-1.93292	2.55885
H	1.19014	-3.36913	1.51590
H	2.69041	-2.53980	1.98696

Revised structure of 48{4}, i.e 50{4-rev} (Acetone-d_6)

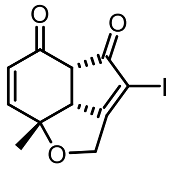

					Conf1
		Rel energy (kcal/mol):			0.00
**C-nom**	**iGau**	**Exp**	**Calc**	**diff**	**1**
C	9	196.80	196.86	0.06	[196.07]
C	7	189.70	189.87	0.17	[189.06]
C	6	187.80	188.12	0.32	[187.30]
C	2	153.30	151.72	-1.58	[150.76]
C	1	128.50	127.07	-1.43	[126.01]
C	8	92.10	92.71	0.61	[91.52]
C	3	78.90	80.14	1.24	[78.90]
C	11	69.40	68.34	-1.06	[67.05]
C	5	59.00	60.54	1.54	[59.22]
C	4	55.90	56.20	0.30	[54.86]
C	12	27.50	26.85	-0.65	[25.40]

13C chem shifts: CRMSD=0.98ppm (CMAE=0.81) N=11 {-1.58 1.54}

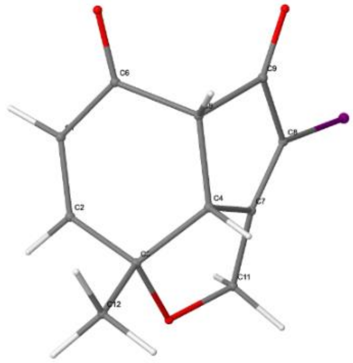

Conformer 1
Energy: -7570.07154 Hartree (Rel: 0.0 kcal/mol)
XYZ coordinates for conf 1:

C	2.50492	0.98504	1.43962
C	2.77700	-0.31490	1.23833
C	2.57667	-1.02794	-0.07688
C	1.54706	-0.29314	-0.95523
C	1.33635	1.22614	-0.85801
C	1.93607	1.87054	0.40573
C	0.25453	-0.87642	-0.44251
C	-0.73477	0.02715	-0.41006
C	-0.21366	1.37175	-0.77546
O	1.96062	-2.32605	0.15801
C	0.52640	-2.24446	0.08325
C	3.92177	-1.29365	-0.75397
O	-0.83887	2.40017	-0.91706
O	1.90291	3.08486	0.54356
H	2.72737	1.46664	2.38819
H	3.21037	-0.91478	2.03651
H	1.69199	-0.59308	-2.00100
H	1.70211	1.78987	-1.71958
I	-2.73807	-0.26574	0.18729
H	0.16327	-3.03919	-0.58047
H	0.07064	-2.38973	1.07178
H	4.42678	-0.35199	-0.99030
H	4.57166	-1.87967	-0.09656
H	3.76233	-1.85608	-1.67964

Originally assigned (incorrect) structure of 49{5} ($CDCl_3$)

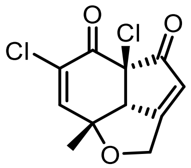

					Conf1
		Rel energy (kcal/mol):			0.00
**C-nom**	**iGau**	**Exp**	**Calc**	**diff**	**1**
C	9	188.40	193.61	5.21	[193.61]
C	7	183.10	181.35	-1.75	[181.35]
C	6	173.10	179.57	6.47	[179.57]
C	2	151.50	149.39	-2.11	[149.39]
C	1	126.00	128.08	2.08	[128.08]
C	8	125.60	122.60	-3.00	[122.60]
C	3	76.60	78.35	1.75	[78.35]
C	5	67.60	69.38	1.78	[69.38]
C	11	65.60	66.04	0.44	[66.04]
C	4	60.10	61.74	1.64	[61.74]
C	12	25.40	24.57	-0.83	[24.57]

13C chem shifts: RMSD=3.01ppm (MAE=2.46) N=11 {-3.00 6.47}

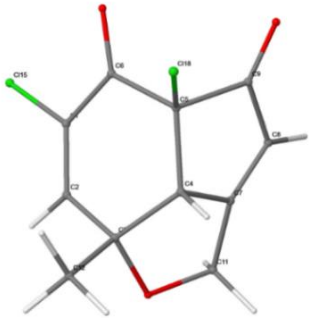

Conformer 1
Energy: -1569.98006 Hartree (Rel: 0.0 kcal/mol)
XYZ coordinates for conf 1:

C	0.92703	-1.42004	-0.07735
C	1.82612	-0.45705	0.17598
C	1.46328	0.93108	0.64806
C	-0.00482	1.25908	0.33699
C	-1.08592	0.17546	0.28793
C	-0.54550	-1.26474	0.08383
C	0.08206	1.71324	-1.10001
C	-0.98349	1.37132	-1.84039
C	-1.89926	0.55254	-1.02075
O	2.19361	1.90189	-0.14525
C	1.45085	2.27057	-1.32303
C	1.88047	1.13318	2.10397
O	-3.01781	0.17081	-1.27363
O	-1.31142	-2.20343	0.03138
Cl	1.44802	-3.01622	-0.58238
H	2.88856	-0.65750	0.06477
H	-0.33367	2.08907	0.97274
Cl	-2.17097	0.17254	1.73179
H	-1.17230	1.58892	-2.88491
H	1.45997	3.36408	-1.40611
H	1.91560	1.85334	-2.22547
H	1.33119	0.45054	2.75920
H	2.95200	0.94789	2.22452
H	1.66893	2.16437	2.40392

Revised structure of 49{5}, i.e 51{5-rev} ($CDCl_3$)

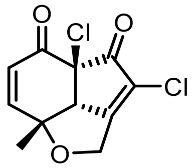

					Conf1
	Rel energy (kcal/mol):				0.00
**C-nom**	**iGau**	**Exp**	**Calc**	**diff**	**1**
C	9	188.40	189.29	0.89	[189.29]
C	6	183.10	184.09	0.99	[184.09]
C	7	173.10	173.45	0.35	[173.45]
C	2	151.50	150.32	-1.18	[150.32]
C	8	126.00	124.74	-1.26	[124.74]
C	1	125.60	124.43	-1.17	[124.43]
C	3	76.60	77.90	1.30	[77.90]
C	5	67.60	65.99	-1.61	[65.99]
C	11	65.60	65.09	-0.51	[65.09]
C	4	60.10	60.96	0.86	[60.96]
C	12	25.40	24.51	-0.89	[24.51]

13C chem shifts: RMSD=1.06ppm (MAE=1.00) N=11 {-1.61 1.30}

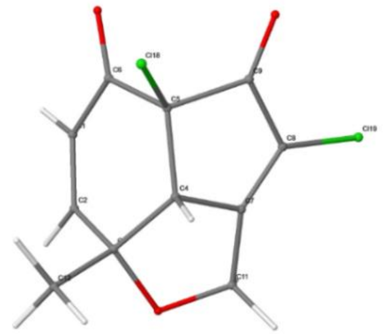

Conformer 1
Energy: -1569.98140 Hartree (Rel: 0.0 kcal/mol)
XYZ coordinates for conf 1:

C	1.35307	0.33987	1.93697
C	1.89041	-0.75153	1.36907
C	1.78247	-1.08720	-0.09710
C	0.60655	-0.33899	-0.75491
C	0.12875	1.03280	-0.25117
C	0.57883	1.35808	1.20636
C	-0.54689	-1.25057	-0.42272
C	-1.68293	-0.57736	-0.19061
C	-1.43388	0.88593	-0.22985
O	1.40673	-2.48380	-0.24530
C	-0.02276	-2.63651	-0.25107
C	3.12954	-0.91240	-0.79676
O	-2.23538	1.78633	-0.18438
O	0.25951	2.41655	1.71496
H	1.49419	0.55483	2.99256
H	2.46964	-1.45142	1.96819
H	0.75725	-0.30733	-1.84011
Cl	0.61807	2.40262	-1.32217
Cl	-3.24020	-1.22100	0.19220
H	-0.29714	-3.30622	-1.07512
H	-0.38003	-3.08379	0.68638
H	3.45495	0.13111	-0.75092
H	3.89036	-1.53609	-0.31757
H	3.04121	-1.21400	-1.84526

Originally assigned (incorrect - benzotriazepinone) structure of 53{3ae} ($CDCl_3$)

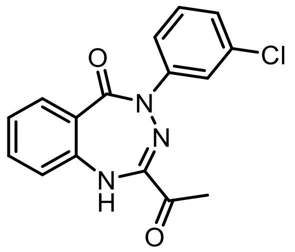

		Conf1	Conf2
Rel energy (kcal/mol):		0.00	0.15

C-nom	iGau	Exp	Calc	diff		1	2	
C	20	193.80	194.77	0.97	[	194.81	194.73	]
C	11	160.40	166.77	6.37	[	166.78	166.75	]
C	13	152.60	145.39	-7.21	[	145.35	145.43	]
C	8	146.80	143.89	-2.91	[	144.09	143.63	]
C	4	146.30	142.01	-4.29	[	142.05	141.96	]
C	6	135.30	135.29	-0.01	[	135.27	135.32	]
C	2	135.20	135.10	-0.10	[	135.09	135.12	]
C	15	130.40	133.21	2.81	[	133.70	132.59	]
C	17	128.40	128.11	-0.29	[	127.59	128.77	]
C	14	128.30	126.24	-2.06	[	125.30	127.43	]
C	16	127.20	125.79	-1.41	[	125.70	125.91	]
C	18	123.00	125.71	2.71	[	126.46	124.75	]
C	1	122.20	122.26	0.06	[	122.28	122.24	]
C	5	114.70	119.96	5.26	[	119.98	119.93	]
C	3	112.60	118.00	5.40	[	118.00	118.00	]
C	22	28.50	24.52	-3.98	[	24.52	24.51	]

13C chem shifts: RMSD=3.66ppm (MAE=2.87) N=16 {-7.21 6.37}

Fractions: 0.561 0.439

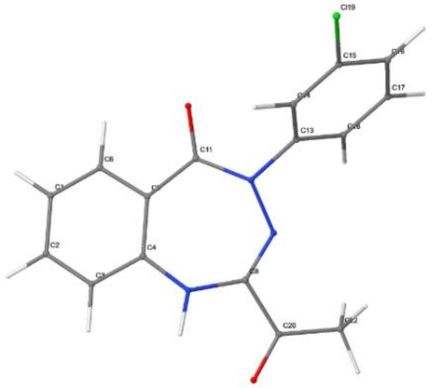

Conformer 1
Energy: -1391.79449 Hartree (Rel: 0.0 kcal/mol)
XYZ coordinates for conf 1:

C	-4.24846	-2.30389	0.92275
C	-4.99176	-1.21011	0.46930
C	-4.34544	-0.10509	-0.07255
C	-2.94579	-0.06976	-0.18094
C	-2.18713	-1.18124	0.23564
C	-2.86612	-2.28201	0.79074
N	-2.36920	1.09450	-0.70925
C	-1.16156	1.61903	-0.29285
N	-0.04854	1.03072	-0.04210
N	0.14860	-0.32456	-0.26582
C	-0.71767	-1.38948	0.03502
O	-0.24614	-2.51788	0.12141
C	1.54882	-0.62880	-0.42947
C	2.48637	-0.03390	0.41868

C	3.83731	-0.30784	0.21976
C	4.27535	-1.15841	-0.79341
C	3.32055	-1.74181	-1.62943
C	1.96254	-1.47781	-1.45949
Cl	5.02237	0.44525	1.28767
C	-1.16128	3.12982	-0.19466
O	-2.19031	3.72368	-0.48682
C	0.09871	3.83173	0.23822
H	-4.74214	-3.16636	1.35894
H	-6.07538	-1.21076	0.54445
H	-4.92191	0.75120	-0.41269
H	-2.26896	-3.13206	1.09906
H	-3.03009	1.84937	-0.88453
H	2.16305	0.63572	1.20541
H	5.33315	-1.35476	-0.92858
H	3.64298	-2.40242	-2.42861
H	1.22745	-1.92718	-2.11675
H	0.94071	3.53960	-0.39624
H	-0.05937	4.91033	0.18609
H	0.35920	3.54447	1.26271

Conformer 2
Energy: -1391.79472 Hartree (Rel: 0.1 kcal/mol)
XYZ coordinates for conf 2:

C	3.62095	-3.06235	-0.57669
C	4.51408	-2.23271	0.10755
C	4.09128	-0.99731	0.58466
C	2.76831	-0.56576	0.39653
C	1.84837	-1.40749	-0.25872
C	2.30536	-2.64830	-0.74051
N	2.42970	0.70436	0.88463
C	1.53583	1.56161	0.27420
N	0.39247	1.32328	-0.25907
N	-0.21192	0.07601	-0.21528
C	0.37337	-1.18804	-0.39779
O	-0.35863	-2.13258	-0.67213
C	-1.63878	0.18352	-0.39947
C	-2.49244	-0.50650	0.46557
C	-3.86739	-0.37010	0.29678
C	-4.41101	0.44310	-0.69729
C	-3.53970	1.13234	-1.54222
C	-2.15759	1.00649	-1.40154
Cl	-4.94773	-1.24111	1.38432
C	1.96521	3.01231	0.32057
O	3.02428	3.28060	0.87146
C	1.06967	4.05733	-0.29120
H	3.94327	-4.02376	-0.96352
H	5.54368	-2.54136	0.26425
H	4.78839	-0.34606	1.10539
H	1.58318	-3.28787	-1.23410
H	3.21354	1.23401	1.26174
H	-2.09078	-1.13742	1.24851
H	-5.48604	0.53528	-0.80498
H	-3.94480	1.77041	-2.32196
H	-1.48131	1.54696	-2.05337
H	0.06520	4.00312	0.13920
H	1.50369	5.04329	-0.11639
H	0.96334	3.88366	-1.36733

Revised structure of 53{3ae}, i.e. quinazolin 54{3ae-rev} ($CDCl_3$)

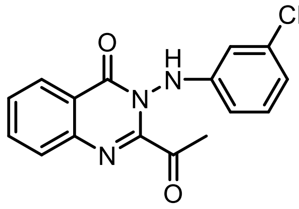

					Conf1
		Rel energy (kcal/mol):			0.00
**C-nom**	**iGau**	**Exp**	**Calc**	**diff**	**1**
C	12	193.80	196.96	3.16	[196.96]
C	10	160.40	160.96	0.56	[160.96]
C	8	152.60	154.39	1.79	[154.39]
C	16	146.80	147.76	0.96	[147.76]
C	4	146.30	145.44	-0.86	[145.44]
C	20	135.30	135.17	-0.13	[135.17]
C	2	135.20	134.86	-0.34	[134.86]
C	18	130.40	128.94	-1.46	[128.94]
C	3	128.40	128.34	-0.06	[128.34]
C	6	128.30	128.46	0.16	[128.46]
C	1	127.20	127.86	0.66	[127.86]
C	5	123.00	122.40	-0.60	[122.40]
C	19	122.20	121.53	-0.67	[121.53]
C	21	114.70	115.48	0.78	[115.48]
C	17	112.60	113.85	1.25	[113.85]
C	13	28.50	27.30	-1.20	[27.30]

13C chem shifts: RMSD=1.18ppm (MAE=0.91) N=16 {-1.46 3.16}

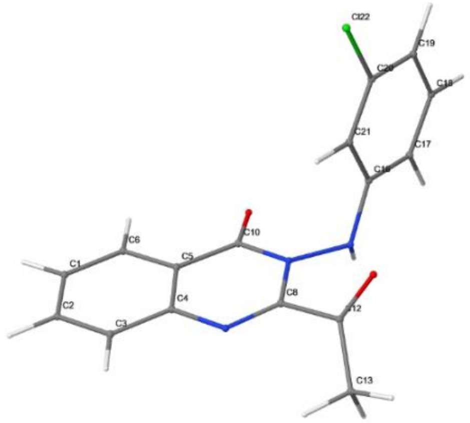

Conformer 1
Energy: -1391.80688 Hartree (Rel: 0.0 kcal/mol)
XYZ coordinates for conf 1:

C	4.28425	-2.38219	0.25622
C	4.84455	-1.29997	0.96013
C	4.18214	-0.08318	1.03221
C	2.93371	0.07701	0.40118
C	2.37384	-1.01423	-0.30343
C	3.05816	-2.23867	-0.37647
N	2.29461	1.30691	0.48213
C	1.13104	1.43162	-0.06831
N	0.48987	0.41952	-0.76254
C	1.09280	-0.84492	-0.98962
O	0.54865	-1.65847	-1.72443
C	0.39407	2.74391	0.18114
C	1.17807	4.00054	-0.09213
O	-0.73248	2.72840	0.63737
N	-0.68300	0.71745	-1.47790

C	-1.88057	0.09946	-1.01919
C	-2.86787	-0.21811	-1.96159
C	-4.08360	-0.75183	-1.54164
C	-4.33558	-0.98916	-0.18826
C	-3.33710	-0.66681	0.72820
C	-2.11579	-0.11989	0.34209
Cl	-3.62513	-0.95107	2.44593
H	4.81357	-3.32889	0.20815
H	5.80598	-1.41841	1.45148
H	4.60133	0.76104	1.57000
H	2.60745	-3.05581	-0.92982
H	2.14846	3.95456	0.41140
H	1.37702	4.08226	-1.16858
H	0.60989	4.87001	0.24353
H	-0.51377	0.48968	-2.45534
H	-2.68107	-0.05113	-3.01909
H	-4.84250	-0.99862	-2.27829
H	-5.27646	-1.41278	0.14380
H	-1.37524	0.14822	1.08344

Originally assigned (incorrect) structure of 58{4ap} as exo-isomer ($CDCl_3$)

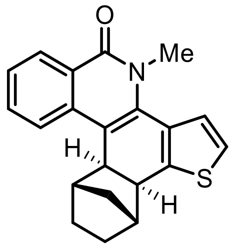

C-nom	iGau	Exp	Calc	diff	Conf1 Rel energy (kcal/mol): 0.00 1
C	10	163.10	163.91	0.81	[163.91]
C	13	142.80	147.73	4.93	[147.73]
C	4	136.20	137.28	1.08	[137.28]
C	8	133.10	136.00	2.90	[136.00]
C	2	132.30	131.52	-0.78	[131.52]
C	14	128.80	131.00	2.20	[131.00]
C	6	128.50	129.13	0.63	[129.13]
C	5	128.10	127.12	-0.98	[127.12]
C	21	126.10	125.85	-0.25	[125.85]
C	1	125.90	125.36	-0.54	[125.36]
C	3	124.90	123.94	-0.96	[123.94]
C	20	123.30	122.47	-0.83	[122.47]
C	7	112.00	113.21	1.21	[113.21]
C	18	47.30	48.66	1.36	[48.66]
C	11	46.90	45.89	-1.01	[45.89]
C	12	44.50	45.68	1.18	[45.68]
C	15	43.70	45.18	1.48	[45.18]
C	24	35.40	38.55	3.15	[38.55]
C	22	34.20	35.30	1.10	[35.30]
C	16	30.30	31.65	1.35	[31.65]
C	17	29.50	27.97	-1.53	[27.97]

13C chem shifts: RMSD=1.78ppm (MAE=1.44) N=21 {-1.53 4.93}

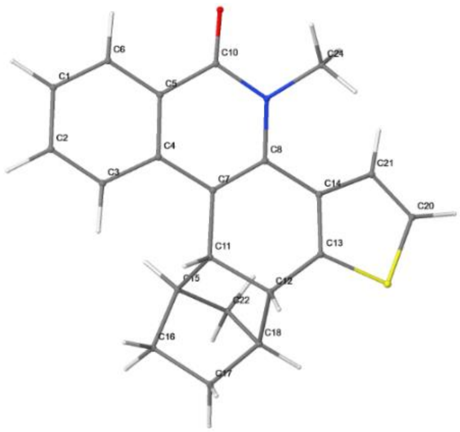

Conformer 1
Energy: -1339.83863 Hartree (Rel: 0.0 kcal/mol)
XYZ coordinates for conf 1:

C	-4.65654	1.48090	0.06634
C	-3.65513	2.43065	-0.20145
C	-2.32717	2.05030	-0.31688
C	-1.93668	0.69459	-0.16894
C	-2.96199	-0.25052	0.07971
C	-4.30484	0.14805	0.19972
C	-0.55985	0.24841	-0.22767
C	-0.27975	-1.09444	-0.11842

N	-1.32030	-2.03625	-0.05587
C	-2.65859	-1.68043	0.13866
C	0.52158	1.29167	-0.45626
C	1.97307	0.76871	-0.77731
C	2.13756	-0.67500	-0.40151
C	1.12691	-1.54538	-0.04599
C	0.76828	2.30329	0.72069
C	1.32382	3.60292	0.09635
C	2.73461	3.15743	-0.41174
C	2.85803	1.70289	0.10514
S	3.70547	-1.40928	-0.22298
C	3.00907	-2.87710	0.38206
C	1.64957	-2.80295	0.43375
C	2.03114	1.74384	1.40497
O	-3.51961	-2.55668	0.28185
C	-1.12814	-3.46054	-0.36954
H	-5.69370	1.78928	0.15934
H	-3.92119	3.47777	-0.31973
H	-1.58360	2.81119	-0.52117
H	-5.04816	-0.61938	0.38715
H	0.18763	1.86693	-1.32770
H	2.21151	0.90757	-1.83992
H	-0.10171	2.44645	1.36483
H	1.40886	4.38801	0.85607
H	0.68793	3.99251	-0.70680
H	2.82763	3.21221	-1.50233
H	3.52662	3.78385	0.01209
H	3.89156	1.35777	0.19373
H	3.64227	-3.70438	0.67246
H	1.05261	-3.60958	0.83296
H	1.88752	0.75984	1.86249
H	2.44188	2.42766	2.15650
H	-0.25404	-3.58370	-1.00555
H	-1.02305	-4.06845	0.53436
H	-2.01900	-3.80379	-0.89489

Originally assigned (incorrect) structure of 58{4ap} as endo-isomer ($CDCl_3$)

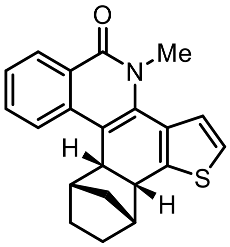

C-nom	iGau	Exp	Calc	diff	Conf1 (Rel energy (kcal/mol): 0.00) 1
C	10	163.10	164.28	1.18	[164.28]
C	13	142.80	148.38	5.58	[148.38]
C	4	136.20	137.31	1.11	[137.31]
C	8	133.10	137.09	3.99	[137.09]
C	2	132.30	131.34	-0.96	[131.34]
C	14	128.80	130.24	1.44	[130.24]
C	6	128.50	128.99	0.49	[128.99]
C	5	128.10	127.29	-0.81	[127.29]
C	21	126.10	126.06	-0.04	[126.06]
C	1	125.90	125.43	-0.47	[125.43]
C	3	124.90	123.82	-1.08	[123.82]
C	20	123.30	122.52	-0.78	[122.52]
C	7	112.00	110.63	-1.37	[110.63]
C	18	47.30	47.90	0.60	[47.90]
C	15	46.90	43.92	-2.98	[43.92]
C	11	44.50	43.40	-1.10	[43.40]
C	12	43.70	39.37	-4.33	[39.37]
C	24	35.40	38.97	3.57	[38.97]
C	22	34.20	37.75	3.55	[37.75]
C	17	30.30	25.46	-4.84	[25.46]
C	16	29.50	22.19	-7.31	[22.19]

13C chem shifts: RMSD=3.00ppm (MAE=2.27) N=21 {-7.31 5.58}

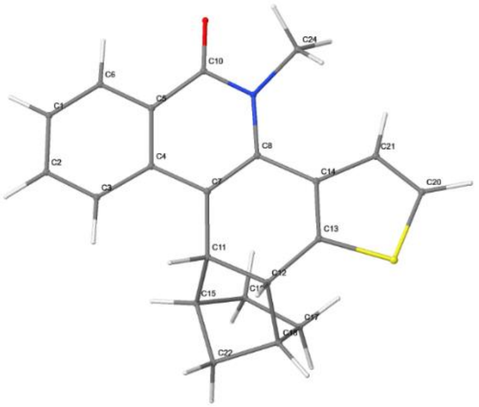

Conformer 1
Energy: -1339.83650 Hartree (Rel: 0.0 kcal/mol)
XYZ coordinates for conf 1:

C	4.78471	-1.14516	-0.08428
C	3.89476	-2.13962	-0.52594
C	2.53735	-1.88112	-0.63565
C	2.00198	-0.60881	-0.30828
C	2.91974	0.39028	0.09752
C	4.29331	0.11411	0.21623
C	0.58647	-0.29702	-0.35205
C	0.17201	0.99990	-0.13552
N	1.11964	2.02046	0.06239

C	2.47255	1.76601	0.31157
C	-0.38421	-1.42040	-0.67151
C	-1.88979	-1.02935	-0.93058
C	-2.18368	0.39478	-0.58623
C	-1.27344	1.32605	-0.12521
C	-0.51526	-2.56880	0.38316
C	-1.02648	-2.00552	1.72858
C	-2.53596	-1.72279	1.44553
C	-2.67722	-2.06725	-0.05492
S	-3.81221	1.00467	-0.54470
C	-3.28802	2.49789	0.16502
C	-1.93747	2.52315	0.34053
C	-1.76028	-3.30120	-0.15784
O	3.23403	2.69312	0.61087
C	0.82156	3.44684	-0.13859
H	5.84556	-1.35935	0.00615
H	4.27158	-3.12506	-0.78694
H	1.88576	-2.67153	-0.98618
H	4.94888	0.91838	0.53249
H	-0.01659	-1.88629	-1.59437
H	-2.14049	-1.21487	-1.98285
H	0.38844	-3.17283	0.48079
H	-0.90093	-2.74483	2.52708
H	-0.47919	-1.10659	2.02950
H	-2.82699	-0.69676	1.68280
H	-3.17954	-2.38665	2.03390
H	-3.71128	-2.19846	-0.38715
H	-4.00488	3.26855	0.41353
H	-1.44840	3.36027	0.81467
H	-2.08800	-4.12650	0.48448
H	-1.63521	-3.67580	-1.18070
H	0.00126	3.55680	-0.84487
H	0.58000	3.95318	0.80100
H	1.71975	3.91379	-0.54114

Revised structure of 58{4ap}, i.e iso-thiophenyl/exo 59{4ap-rev} ($CDCl_3$)

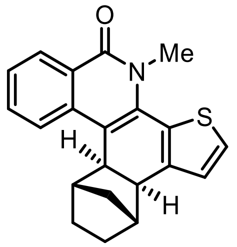

					Conf1
	Rel energy (kcal/mol):				0.00
**C-nom**	**iGau**	**Exp**	**Calc**	**diff**	**1**
C	10	163.10	163.04	-0.06	[163.04]
C	13	142.80	143.00	0.20	[143.00]
C	4	136.20	136.95	0.75	[136.95]
C	8	133.10	136.24	3.14	[136.24]
C	2	132.30	131.65	-0.65	[131.65]
C	14	128.80	130.22	1.42	[130.22]
C	6	128.50	129.41	0.91	[129.41]
C	19	128.10	127.51	-0.59	[127.51]
C	20	126.10	126.43	0.33	[126.43]
C	5	125.90	126.74	0.84	[126.74]
C	1	124.90	125.47	0.57	[125.47]
C	3	123.30	123.68	0.38	[123.68]
C	7	112.00	112.99	0.99	[112.99]
C	18	47.30	47.73	0.43	[47.73]
C	15	46.90	47.23	0.33	[47.23]
C	12	44.50	45.54	1.04	[45.54]
C	11	43.70	45.24	1.54	[45.24]
C	24	35.40	35.61	0.21	[35.61]
C	22	34.20	33.88	-0.32	[33.88]
C	17	30.30	30.70	0.40	[30.70]
C	16	29.50	30.30	0.80	[30.30]

13C chem shifts: RMSD=1.00ppm (MAE=0.76) N=21 {-0.65 3.14}

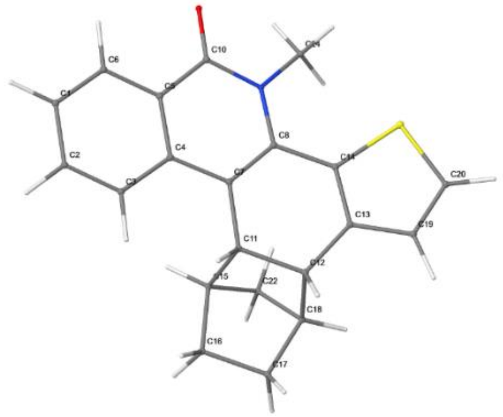

Conformer 1
Energy: -1339.84041 Hartree (Rel: 0.0 kcal/mol)
XYZ coordinates for conf 1:

C	-4.65283	1.21887	-0.52598
C	-3.70294	2.23107	-0.74886
C	-2.34656	1.97148	-0.63271
C	-1.87268	0.67698	-0.29355
C	-2.84503	-0.32922	-0.07254
C	-4.21832	-0.05189	-0.18802
C	-0.46701	0.33990	-0.21904
C	-0.10078	-0.96434	0.04860
N	-1.06860	-1.91304	0.41911

C	-2.44523	-1.67759	0.32732
C	0.54530	1.45041	-0.44354
C	2.05154	1.02760	-0.60080
C	2.28102	-0.45272	-0.52370
C	1.30151	-1.35823	-0.15997
C	0.60595	2.43164	0.78137
C	1.29275	3.73944	0.32329
C	2.78907	3.31382	0.15916
C	2.76693	1.81670	0.54274
C	3.52337	-1.06710	-0.86047
C	3.49111	-2.42962	-0.77357
S	1.93995	-2.99908	-0.26818
C	1.70128	1.78866	1.65384
O	-3.25267	-2.57083	0.60977
C	-0.71714	-3.18291	1.06950
H	-5.71369	1.43284	-0.61641
H	-4.03220	3.23208	-1.01495
H	-1.64564	2.77762	-0.81224
H	-4.91775	-0.85971	-0.00189
H	0.24111	2.00848	-1.33590
H	2.44030	1.37036	-1.56775
H	-0.36239	2.58403	1.26267
H	1.18433	4.51734	1.08719
H	0.86076	4.13247	-0.60419
H	3.16742	3.47452	-0.85652
H	3.43725	3.87564	0.84086
H	3.74874	1.40629	0.79504
H	4.40206	-0.50584	-1.16102
H	4.28777	-3.13788	-0.95920
H	1.45108	0.77919	1.99648
H	1.96945	2.40049	2.52291
H	0.16594	-3.04819	1.69329
H	-1.56417	-3.47206	1.68954
H	-0.54586	-3.98232	0.34243

Originally assigned (incorrect) structure of 62{3ra} ($CDCl_3$)

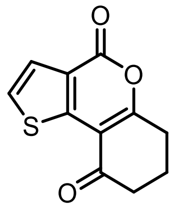

					Conf1
Rel energy (kcal/mol):					0.00
**C-nom**	**iGau**	**Exp**	**Calc**	**diff**	**1**
C-C	7	195.10	195.07	-0.03	[195.07]
C-C	4	170.80	170.23	-0.57	[170.23]
C-C	6	156.50	155.17	-1.33	[155.17]
C-C	2	143.70	143.43	-0.27	[143.43]
C-C	1	123.00	123.00	0.00	[123.00]
C-C	3	112.40	112.88	0.48	[112.88]
C-CH	12	137.70	126.86	-10.84	[126.86]
C-CH	11	126.20	124.17	-2.03	[124.17]
C-CH2	8	37.60	35.95	-1.65	[35.95]
C-CH2	10	28.20	27.70	-0.50	[27.70]
C-CH2	9	20.30	20.63	0.33	[20.63]

13C chem shifts: RMSD=3.40ppm (MAE=1.64) N=11 {-10.84 0.48}

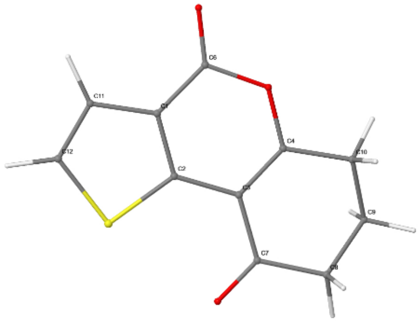

Conformer 1
Energy: -1047.85802 Hartree (Rel: 0.0 kcal/mol)
XYZ coordinates for conf 1:

C	1.65959	0.60419	0.00465
C	0.75635	-0.45412	0.00087
C	-0.66081	-0.22859	-0.03614
C	-1.08166	1.07666	-0.05059
O	-0.21881	2.11168	-0.04717
C	1.19133	1.96959	-0.02841
C	-1.63458	-1.33507	-0.08546
C	-3.10296	-0.96528	-0.21483
C	-3.43257	0.38042	0.44410
C	-2.52019	1.49577	-0.08509
C	3.02818	0.18528	0.03926
C	3.13792	-1.17178	0.06231
S	1.58410	-1.97798	0.03950
O	1.84062	2.99046	-0.03721
O	-1.25489	-2.50263	-0.05908
H	-3.69792	-1.78389	0.20038
H	-3.33014	-0.91968	-1.29112
H	-4.47927	0.64308	0.26235
H	-3.30612	0.29674	1.53047
H	-2.63308	2.41707	0.49619
H	-2.78386	1.74898	-1.12319
H	3.86446	0.87357	0.04629
H	4.04136	-1.76666	0.09037

Revised structure of 62{3ra}, i.e. *cis* 63{3ra-rev} ($CDCl_3$)

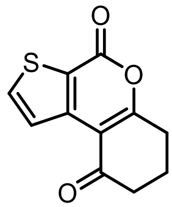

					Conf1
			Rel energy (kcal/mol):		0.00
**C-nom**	**iGau**	**Exp**	**Calc**	**diff**	**1**
C-C	7	195.10	196.52	1.42	[196.52]
C-C	4	170.80	173.02	2.22	[173.02]
C-C	6	156.50	155.56	-0.94	[155.56]
C-C	2	143.70	145.29	1.59	[145.29]
C-C	1	123.00	122.39	-0.61	[122.39]
C-C	3	112.40	112.70	0.30	[112.70]
C-CH	12	137.70	137.54	-0.16	[137.54]
C-CH	13	126.20	125.61	-0.59	[125.61]
C-CH2	8	37.60	37.23	-0.37	[37.23]
C-CH2	10	28.20	28.08	-0.12	[28.08]
C-CH2	9	20.30	20.32	0.02	[20.32]

13C chem shifts: RMSD=1.02ppm (MAE=0.76) N=11 {-0.94 2.22}

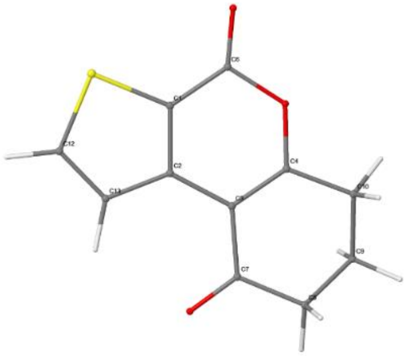

Conformer 1
Energy: -1047.85546 Hartree (Rel: 0.0 kcal/mol)
XYZ coordinates for conf 1:

C	1.55412	0.33304	-0.00020
C	0.60464	-0.68634	0.00288
C	-0.79781	-0.31995	-0.03322
C	-1.10294	1.01692	-0.04920
O	-0.15640	1.98198	-0.04995
C	1.23105	1.72826	-0.03473
C	-1.88889	-1.32283	-0.07932
C	-3.31089	-0.79767	-0.22265
C	-3.51301	0.56596	0.44563
C	-2.49181	1.58122	-0.07947
S	3.18372	-0.27059	0.03893
C	2.58624	-1.89760	0.05995
C	1.21984	-1.97775	0.03809
O	1.97331	2.68593	-0.04675
O	-1.66267	-2.52798	-0.04094
H	-3.98877	-1.55795	0.17577
H	-3.51833	-0.71813	-1.30105
H	-4.52809	0.93236	0.26344
H	-3.39742	0.46427	1.53182
H	-2.50588	2.50802	0.50370
H	-2.72875	1.86348	-1.11653
H	3.28969	-2.71982	0.08846
H	0.66079	-2.90171	0.04598

Originally assigned correct structure of cinnoline 65{11g} ($CDCl_3$)

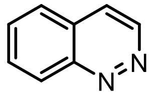

					Conf1
	Rel energy (kcal/mol):				0.00
**C-nom**	**iGau**	**Exp**	**Calc**	**diff**	**1**
C	4	150.80	151.00	0.20	[151.00]
C	9	145.00	146.02	1.02	[146.02]
C	1	131.20	131.18	-0.02	[131.18]
C	2	130.70	130.51	-0.19	[130.51]
C	3	129.80	130.09	0.29	[130.09]
C	6	126.60	126.44	-0.16	[126.44]
C	5	126.00	125.01	-0.99	[125.01]
C	10	122.60	121.58	-1.02	[121.58]

13C chem shifts: RMSD=0.64ppm (MAE=0.49) N=8 {-1.02 1.02}

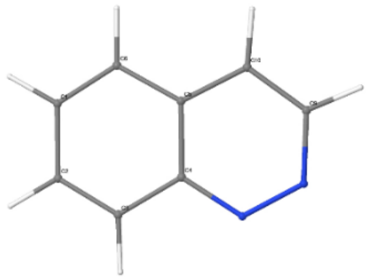

Conformer 1
Energy: -417.92910 Hartree (Rel: 0.0 kcal/mol)
XYZ coordinates for conf 1:

C	2.39743	0.70297	0.00000
C	2.37926	-0.71996	-0.00000
C	1.18706	-1.40377	-0.00000
C	-0.04097	-0.68551	0.00000
C	-0.02303	0.74345	0.00000
C	1.22556	1.42324	0.00000
N	-1.20112	-1.40752	0.00000
N	-2.35666	-0.79393	0.00000
C	-2.40297	0.56699	-0.00000
C	-1.28419	1.37108	-0.00000
H	3.35220	1.22086	0.00000
H	3.32003	-1.26256	-0.00000
H	1.14321	-2.48838	-0.00000
H	1.23632	2.51005	0.00000
H	-3.40519	0.98502	-0.00000
H	-1.37097	2.45415	-0.00000

Originally assigned correct structure of 66{11u} (CD_3OD)

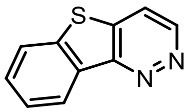

	Conf1
Rel energy (kcal/mol):	0.00

C-nom	iGau	Exp	Calc	diff	1
C	6	157.00	158.96	1.96	[158.96]
C	9	147.20	148.80	1.60	[148.80]
C	2	141.40	141.95	0.55	[141.95]
C	5	141.00	141.19	0.19	[141.19]
C	3	133.10	133.53	0.43	[133.53]
C	11	132.00	130.87	-1.13	[130.87]
C	12	127.40	126.79	-0.61	[126.79]
C	13	124.40	124.46	0.06	[124.46]
C	10	124.30	123.12	-1.18	[123.12]
C	4	122.90	121.38	-1.52	[121.38]

13C chem shifts: RMSD=1.11ppm (MAE=0.92) N=10 {-1.52 1.96}

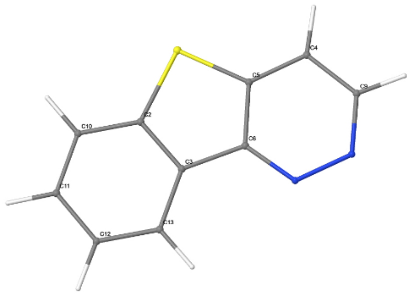

Conformer 1
Energy: -892.34329 Hartree (Rel: 0.0 kcal/mol)
XYZ coordinates for conf 1:

S	-0.04220	1.98654	-0.00000
C	1.22623	0.73940	0.00000
C	0.70184	-0.56952	-0.00000
C	-2.66572	0.88632	0.00000
C	-1.28795	0.75118	-0.00000
C	-0.75211	-0.56324	-0.00000
N	-1.51427	-1.66239	0.00000
N	-2.84079	-1.53445	0.00000
C	-3.39025	-0.30956	0.00000
C	2.60313	0.97161	0.00000
C	3.45579	-0.13208	0.00000
C	2.94696	-1.44204	0.00000
C	1.57427	-1.66700	-0.00000
H	-3.16879	1.84725	0.00000
H	-4.47645	-0.29459	0.00000
H	3.00209	1.98106	0.00000
H	4.53012	0.02725	0.00000
H	3.63209	-2.28438	0.00000
H	1.16849	-2.67378	-0.00000

Originally assigned (incorrect) structure of 67{11t} (CD_3OD)

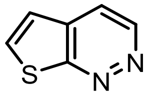

	Conf1
Rel energy (kcal/mol):	0.00

C-nom	iGau	Exp	Calc	diff	1
C	4	161.00	168.01	7.01	[168.01]
C	1	144.70	148.73	4.03	[148.73]
C	5	139.70	135.68	-4.02	[135.68]
C	8	136.40	134.61	-1.79	[134.61]
C	9	124.00	122.12	-1.88	[122.12]
C	6	123.00	121.64	-1.36	[121.64]

13C chem shifts: RMSD=3.88ppm (MAE=3.35) N=6 {-4.02 7.01}

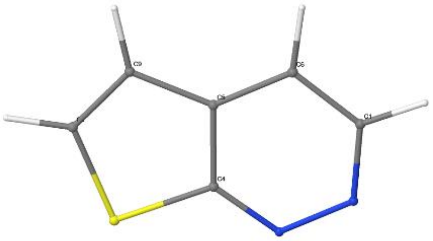

Conformer 1
Energy: -738.69215 Hartree (Rel: 0.0 kcal/mol)
XYZ coordinates for conf 1:

C	2.45343	0.34960	-0.00002
N	2.22027	-0.97666	-0.00002
N	0.96333	-1.42432	-0.00002
C	-0.01796	-0.52844	0.00000
C	0.14122	0.88489	0.00000
C	1.46282	1.32999	-0.00001
S	-1.72096	-0.98236	0.00002
C	-2.17312	0.70958	0.00001
C	-1.12045	1.57128	0.00002
H	3.50479	0.62252	-0.00003
H	1.71870	2.38495	-0.00001
H	-3.22497	0.96564	0.00001
H	-1.22401	2.65010	0.00000

Revised structure of 67{11t}, i.e. 68{11t-rev} (CD_3OD)

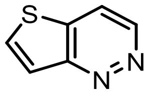

	Conf1
Rel energy (kcal/mol):	0.00

C-nom	iGau	Exp	Calc	diff	1
C	4	161.00	161.57	0.57	[161.57]
C	1	144.70	146.08	1.38	[146.08]
C	5	139.70	139.33	-0.37	[139.33]
C	8	136.40	135.21	-1.19	[135.21]
C	7	124.00	124.44	0.44	[124.44]
C	6	123.00	120.91	-2.09	[120.91]

13C chem shifts: RMSD=1.18ppm (MAE=1.01) N=6 {-2.09 1.38}

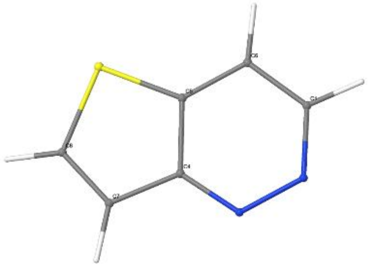

Conformer 1
Energy: -738.69171 Hartree (Rel: 0.0 kcal/mol)
XYZ coordinates for conf 1:

C	2.29827	-0.74765	0.00001
N	2.44632	0.59071	-0.00001
N	1.37768	1.37858	-0.00002
C	0.15529	0.81863	-0.00001
C	-0.04831	-0.58913	0.00001
C	1.06911	-1.40871	0.00003
C	-1.08665	1.55153	-0.00002
C	-2.16335	0.72784	0.00006
S	-1.75030	-0.98354	-0.00003
H	3.22754	-1.30980	0.00001
H	1.01254	-2.49208	0.00003
H	-1.13984	2.63349	-0.00003
H	-3.20953	1.00493	0.00010

Originally assigned (incorrect) structure of 64{6t} ($CDCl_3$)

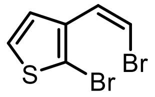

					Conf1
		Rel energy (kcal/mol):			0.00
**C-nom**	**iGau**	**Exp**	**Calc**	**diff**	**1**
C	2	130.70	133.29	2.59	[133.29]
C	3	129.50	125.52	-3.98	[125.52]
C	4	126.70	125.16	-1.54	[125.16]
C	7	125.00	123.04	-1.96	[123.04]
C	1	114.30	117.96	3.66	[117.96]
C	8	106.50	106.08	-0.42	[106.08]

13C chem shifts: RMSD=2.66ppm (MAE=2.36) N=6 {-3.98 3.66}

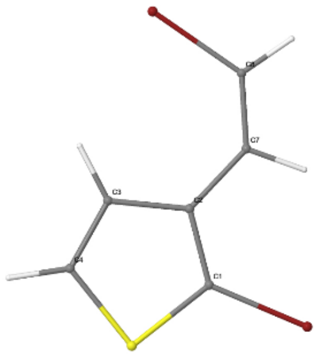

Conformer 1
Energy: -5771.99768 Hartree (Rel: 0.0 kcal/mol)
XYZ coordinates for conf 1:

C	1.48708	0.28887	0.00008
C	0.10281	0.23706	0.00045
C	-0.43932	1.57518	0.00054
C	0.50722	2.55425	0.00023
S	2.11818	1.90288	-0.00019
Br	2.69381	-1.16443	-0.00017
C	-0.62315	-1.02598	0.00078
C	-1.92765	-1.33058	0.00060
Br	-3.41233	-0.13219	-0.00030
H	-1.50027	1.78165	0.00085
H	0.36036	3.62590	0.00024
H	0.01850	-1.90575	0.00124
H	-2.26324	-2.35919	0.00094

Revised structure of precursor 64{6t}, i.e. 69{6t-rev} ($CDCl_3$)

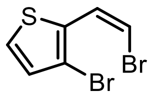

	**Conf1**
Rel energy (kcal/mol):	0.00

C-nom	iGau	Exp	Calc	diff	1
C	2	130.70	130.31	-0.39	[130.31]
C	5	129.50	128.38	-1.12	[128.38]
C	4	126.70	126.52	-0.18	[126.52]
C	7	125.00	123.08	-1.92	[123.08]
C	1	114.30	114.26	-0.04	[114.26]
C	8	106.50	106.41	-0.09	[106.41]

13C chem shifts: RMSD=0.93ppm (MAE=0.62) N=6 {-1.92 -0.04}

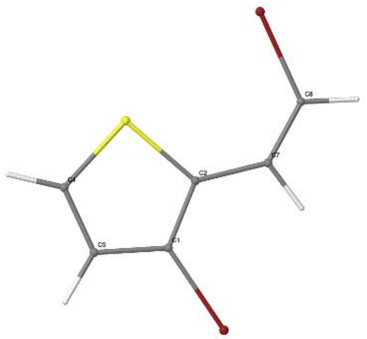

Conformer 1
Energy: -5772.00428 Hartree (Rel: 0.0 kcal/mol)
XYZ coordinates for conf 1:

C	-1.61147	0.44964	0.00002
C	-0.24799	0.20576	0.00005
S	0.59774	1.74807	0.00035
C	-0.88718	2.63764	-0.00033
C	-1.98298	1.82077	-0.00015
Br	-2.93229	-0.91963	-0.00001
C	0.37818	-1.09670	0.00008
C	1.65991	-1.49029	0.00006
Br	3.18551	-0.35356	-0.00009
H	-0.86487	3.71937	-0.00057
H	-3.00935	2.16540	-0.00030
H	-0.33813	-1.91698	0.00013
H	1.93511	-2.53623	0.00011

Originally assigned (incorrect) structure of 71{2a} (DMSO-d_6)

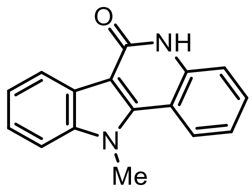

Conf1

Rel energy (kcal/mol): 0.00

C-nom	iGau	Exp	Calc	diff	1
C	14	156.90	159.42	2.52	[159.42]
C	8	140.60	141.26	0.66	[141.26]
C	4	135.40	138.03	2.63	[138.03]
C	12	126.60	137.86	11.26	[137.86]
C	18	126.60	129.71	3.11	[129.71]
C	16	126.40	123.88	-2.52	[123.88]
C	5	123.40	124.43	1.03	[124.43]
C	2	123.00	123.29	0.29	[123.29]
C	1	122.80	121.36	-1.44	[121.36]
C	6	121.70	120.63	-1.07	[120.63]
C	17	121.50	121.64	0.14	[121.64]
C	11	119.10	115.72	-3.38	[115.72]
C	19	118.30	115.76	-2.54	[115.76]
C	9	116.30	108.92	-7.38	[108.92]
C	3	111.60	109.40	-2.20	[109.40]
C	10	31.70	32.17	0.47	[32.17]

13C chem shifts: RMSD=3.86ppm (MAE=2.67) N=16 {-7.38 11.26}

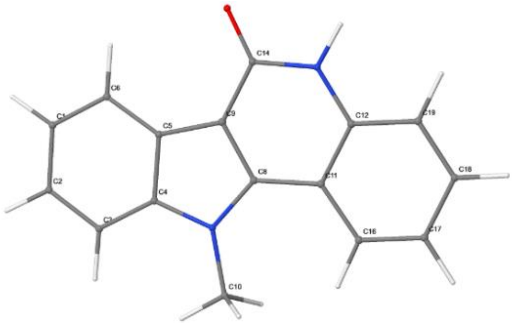

Conformer 1
Energy: -801.70648 Hartree (Rel: 0.0 kcal/mol)
XYZ coordinates for conf 1:

C	-4.26870	0.69061	-0.00044
C	-4.36639	-0.71578	-0.00036
C	-3.23197	-1.52377	-0.00015
C	-1.98273	-0.89046	-0.00001
C	-1.87072	0.52427	-0.00013
C	-3.02885	1.31917	-0.00033
N	-0.69872	-1.43978	0.00027
C	0.22404	-0.40895	0.00013
C	-0.46680	0.80945	0.00003
C	-0.43429	-2.87307	0.00119
C	1.67144	-0.45922	-0.00004
C	2.32705	0.80776	0.00025
N	1.58748	1.98036	0.00051
C	0.20178	2.08657	0.00030
O	-0.34431	3.19874	0.00010
C	2.48353	-1.61575	-0.00071
C	3.86838	-1.52919	-0.00082
C	4.49444	-0.27431	-0.00030
C	3.73064	0.88309	0.00018

H	-5.17644	1.28762	-0.00060
H	-5.34708	-1.18313	-0.00048
H	-3.33146	-2.60426	-0.00016
H	-2.93958	2.40001	-0.00040
H	0.12299	-3.16836	0.89454
H	-1.38448	-3.40474	0.00334
H	0.12017	-3.17021	-0.89333
H	2.08595	2.86346	0.00059
H	2.02983	-2.59639	-0.00130
H	4.46252	-2.43770	-0.00135
H	5.57802	-0.20224	-0.00036
H	4.20776	1.85937	0.00046

Revised structure of 71{2a}, i.e. 75{2a-rev} (DMSO-d_6)

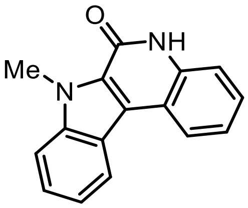

					Conf1
		Rel energy (kcal/mol):			0.00
**C-nom**	**iGau**	**Exp**	**Calc**	**diff**	**1**
C	6	156.90	157.06	0.16	[157.06]
C	13	140.60	138.93	-1.67	[138.93]
C	4	135.40	134.18	-1.22	[134.18]
C	2	126.60	127.91	1.31	[127.91]
C	10	126.60	126.48	-0.12	[126.48]
C	16	126.40	125.51	-0.89	[125.51]
C	8	123.40	123.58	0.18	[123.58]
C	18	123.00	122.70	-0.30	[122.70]
C	14	122.80	122.58	-0.22	[122.58]
C	9	121.70	122.70	1.00	[122.70]
C	3	121.50	120.79	-0.71	[120.79]
C	1	119.10	119.42	0.32	[119.42]
C	17	118.30	120.31	2.01	[120.31]
C	11	116.30	115.35	-0.95	[115.35]
C	15	111.60	110.11	-1.49	[110.11]
C	19	31.70	31.64	-0.06	[31.64]

13C chem shifts: RMSD=0.99ppm (MAE=0.79) N=16 {-1.67 2.01}

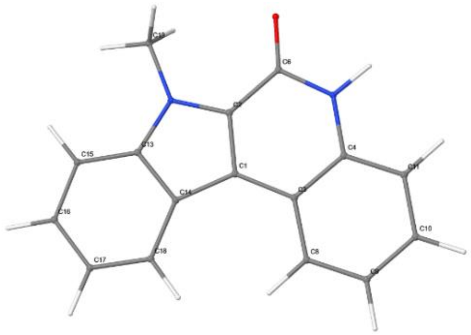

Conformer 1
Energy: -801.70403 Hartree (Rel: 0.0 kcal/mol)
XYZ coordinates for conf 1:

C	0.04392	-0.21593	0.00011
C	-0.26632	1.14587	0.00017
C	1.42623	-0.64378	0.00010
C	2.41499	0.37647	-0.00008
N	2.02157	1.71177	-0.00015
C	0.72862	2.20336	0.00004
O	0.50636	3.42146	-0.00024
C	1.86720	-1.98285	0.00022
C	3.21991	-2.29862	0.00015
C	4.18043	-1.27652	-0.00006
C	3.78112	0.05376	-0.00017
N	-1.63417	1.33590	0.00019
C	-2.23361	0.09055	0.00006
C	-1.21785	-0.91437	0.00005
C	-3.59857	-0.23820	-0.00009
C	-3.94278	-1.58282	-0.00019
C	-2.95616	-2.59184	-0.00018
C	-1.60667	-2.26955	-0.00007
C	-2.32675	2.62071	0.00023

H	2.74244	2.42548	-0.00036
H	1.13853	-2.78421	0.00041
H	3.52995	-3.33932	0.00026
H	5.23905	-1.51892	-0.00012
H	4.51847	0.85219	-0.00032
H	-4.36856	0.52612	-0.00020
H	-4.99270	-1.86173	-0.00031
H	-3.25849	-3.63507	-0.00028
H	-0.86989	-3.06473	-0.00012
H	-3.40078	2.43805	0.00255
H	-2.05977	3.20053	0.88594
H	-2.06332	3.19889	-0.88767

Originally assigned (incorrect) structure of 72{2d} (DMSO-d_6)

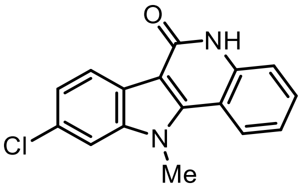

					**Conf1**
	Rel energy (kcal/mol):				0.00

**C-nom**	**iGau**	**Exp**	**Calc**	**diff**	**1**
C	14	156.70	159.15	2.45	[159.15]
C	8	141.20	142.48	1.28	[142.48]
C	4	135.50	138.22	2.72	[138.22]
C	12	131.30	138.05	6.75	[138.05]
C	18	130.10	130.22	0.12	[130.22]
C	2	127.30	129.72	2.42	[129.72]
C	16	127.00	123.95	-3.05	[123.95]
C	5	123.50	123.81	0.31	[123.81]
C	6	123.00	121.57	-1.43	[121.57]
C	17	121.90	121.85	-0.05	[121.85]
C	1	120.40	121.38	0.98	[121.38]
C	11	119.20	115.28	-3.92	[115.28]
C	19	117.80	115.89	-1.91	[115.89]
C	9	116.40	108.68	-7.72	[108.68]
C	3	111.60	109.59	-2.01	[109.59]
C	10	32.00	32.34	0.34	[32.34]

13C chem shifts: RMSD=3.18ppm (MAE=2.34) N=16 {-7.72 6.75}

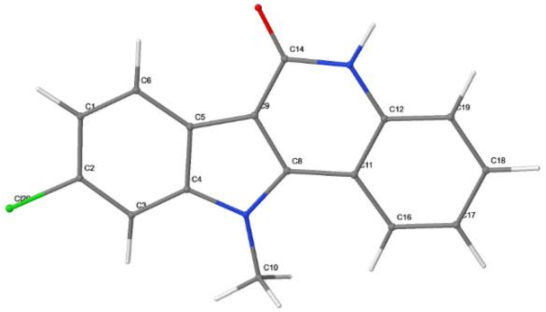

Conformer 1
Energy: -1261.29924 Hartree (Rel: 0.0 kcal/mol)
XYZ coordinates for conf 1:

C	-3.49121	1.26992	-0.00027
C	-3.72966	-0.11632	-0.00017
C	-2.71150	-1.06269	0.00001
C	-1.40024	-0.57230	0.00012
C	-1.12591	0.82010	-0.00003
C	-2.18420	1.74124	-0.00020
N	-0.19204	-1.26678	0.00036
C	0.84467	-0.34766	0.00016
C	0.29970	0.94144	0.00007
C	-0.09362	-2.72181	0.00129
C	2.27622	-0.56333	-0.00004
C	3.07154	0.62109	0.00017
N	2.47209	1.87110	0.00042
C	1.10855	2.13548	0.00028
O	0.68888	3.30059	0.00012
C	2.95112	-1.80503	-0.00067
C	4.33650	-1.87637	-0.00083
C	5.10126	-0.70057	-0.00041
C	4.47447	0.53610	0.00004
Cl	-5.40393	-0.68183	-0.00032
H	-4.32849	1.95903	-0.00041

H	-2.94580	-2.12028	-0.00001
H	-1.97487	2.80492	-0.00029
H	0.42601	-3.07738	0.89509
H	-1.09759	-3.14286	0.00340
H	0.42307	-3.07891	-0.89363
H	3.06997	2.69025	0.00050
H	2.38871	-2.72766	-0.00118
H	4.82352	-2.84645	-0.00133
H	6.18594	-0.75239	-0.00052
H	5.05886	1.45219	0.00025

Revised structure of 72{2d}, i.e. 76{2d-rev} (DMSO-d_6)

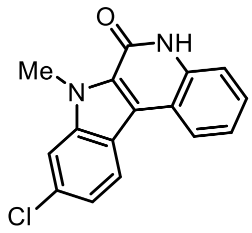

					Conf1
		Rel energy (kcal/mol):			0.00
**C-nom**	**iGau**	**Exp**	**Calc**	**diff**	**1**
C	6	156.70	156.78	0.08	[156.78]
C	13	141.20	139.01	-2.19	[139.01]
C	4	135.50	134.33	-1.17	[134.33]
C	16	131.30	132.01	0.71	[132.01]
C	2	130.10	128.95	-1.15	[128.95]
C	10	127.30	127.00	-0.30	[127.00]
C	8	124.50	123.52	-0.98	[123.52]
C	18	123.50	123.73	0.23	[123.73]
C	9	123.00	122.86	-0.14	[122.86]
C	14	121.90	121.91	0.01	[121.91]
C	1	120.40	119.45	-0.95	[119.45]
C	3	119.20	120.18	0.98	[120.18]
C	17	117.80	120.32	2.52	[120.32]
C	11	116.40	115.45	-0.95	[115.45]
C	15	111.60	110.08	-1.52	[110.08]
C	19	32.00	31.86	-0.14	[31.86]

13C chem shifts: RMSD=1.13ppm (MAE=0.88) N=16 {-2.19 2.52}

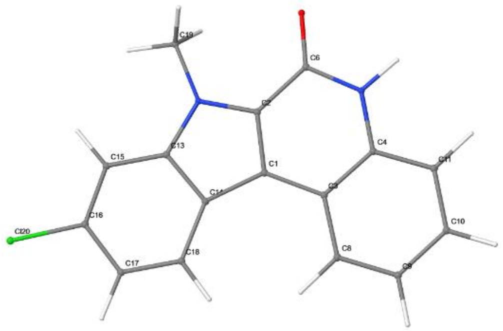

Conformer 1
Energy: -1261.29659 Hartree (Rel: 0.0 kcal/mol)
XYZ coordinates for conf 1:

C	-0.65401	-0.12481	-0.00014
C	-0.61820	1.27033	-0.00015
C	-1.92359	-0.81864	-0.00009
C	-3.09401	-0.01329	0.00007
N	-2.97192	1.37308	0.00010
C	-1.80262	2.11135	-0.00005
O	-1.82381	3.34880	0.00016
C	-2.09046	-2.21848	-0.00013
C	-3.35386	-2.79504	-0.00003
C	-4.49740	-1.98249	0.00014
C	-4.36964	-0.59974	0.00019
N	0.68576	1.72871	-0.00021
C	1.51677	0.62812	-0.00012
C	0.72085	-0.55865	-0.00015
C	2.91932	0.58484	0.00003
C	3.50666	-0.66966	0.00002
C	2.75875	-1.86332	-0.00006
C	1.37322	-1.80654	-0.00014

C	1.10882	3.12633	-0.00010
Cl	5.26715	-0.78192	0.00020
H	-3.81978	1.92997	0.00030
H	-1.21793	-2.86031	-0.00026
H	-3.45238	-3.87636	-0.00006
H	-5.48708	-2.42952	0.00024
H	-5.25062	0.03680	0.00032
H	3.52849	1.48058	0.00023
H	3.27067	-2.81905	-0.00005
H	0.81497	-2.73519	-0.00016
H	2.19749	3.16273	-0.00362
H	0.72944	3.63993	-0.88533
H	0.73525	3.63835	0.88860

Originally assigned (incorrect) structure of 73{2e} (DMSO-d_6)

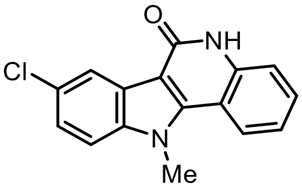

					Conf1
		Rel energy (kcal/mol):			0.00

C-nom	iGau	Exp	Calc	diff	1
C	14	156.70	159.13	2.43	[159.13]
C	8	139.00	142.66	3.66	[142.66]
C	12	135.40	138.12	2.72	[138.12]
C	4	127.50	136.89	9.39	[136.89]
C	18	126.90	130.31	3.41	[130.31]
C	1	126.50	127.50	1.00	[127.50]
C	5	126.10	125.42	-0.68	[125.42]
C	16	123.60	124.01	0.41	[124.01]
C	2	123.00	122.77	-0.23	[122.77]
C	17	122.40	121.84	-0.56	[121.84]
C	6	122.00	119.74	-2.26	[119.74]
C	11	118.60	115.28	-3.32	[115.28]
C	19	117.80	115.91	-1.89	[115.91]
C	3	116.30	110.81	-5.49	[110.81]
C	9	113.40	108.32	-5.08	[108.32]
C	10	32.00	32.41	0.41	[32.41]

13C chem shifts: RMSD=3.57ppm (MAE=2.68) N=16 {-5.49 9.39}

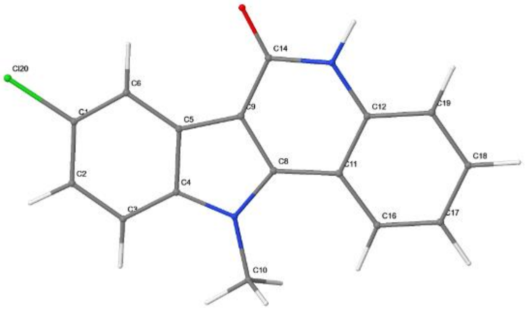

Conformer 1
Energy: -1261.29966 Hartree (Rel: 0.0 kcal/mol)
XYZ coordinates for conf 1:

C	3.64946	-0.02305	-0.00037
C	3.58772	1.38233	-0.00044
C	2.35626	2.03071	-0.00020
C	1.19750	1.24540	0.00016
C	1.27053	-0.17216	0.00005
C	2.51527	-0.82040	-0.00017
N	-0.14467	1.62189	0.00059
C	-0.92492	0.47888	0.00031
C	-0.08145	-0.63919	0.00021
C	-0.59582	3.00895	0.00218
C	-2.36618	0.33675	-0.00010
C	-2.84720	-1.00614	0.00036
N	-1.96043	-2.07181	0.00088
C	-0.57344	-1.99440	0.00056
O	0.11918	-3.02097	0.00064
C	-3.32350	1.37621	-0.00134
C	-4.68421	1.10685	-0.00166
C	-5.13823	-0.22026	-0.00082
C	-4.22855	-1.26643	0.00012
Cl	5.24163	-0.79471	-0.00065
H	4.50433	1.96167	-0.00074

H	2.32368	3.11483	-0.00049
H	2.57238	-1.90192	-0.00018
H	-1.18745	3.22583	0.89564
H	0.27615	3.66105	0.00599
H	-1.18299	3.22973	-0.89332
H	-2.33898	-3.01255	0.00113
H	-3.00318	2.40828	-0.00229
H	-5.39405	1.92814	-0.00265
H	-6.20287	-0.43456	-0.00104
H	-4.57179	-2.29744	0.00057

Revised structure of 73{2e}, i.e. 77{2e-rev} (DMSO-d_6)

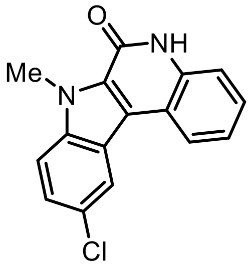

C-nom	iGau	Exp	Calc	diff	Conf1 Rel energy (kcal/mol): 0.00 1
C	6	156.70	156.82	0.12	[156.82]
C	13	139.00	137.65	-1.35	[137.65]
C	4	135.40	134.25	-1.15	[134.25]
C	2	127.50	129.11	1.61	[129.11]
C	10	126.90	126.92	0.02	[126.92]
C	17	126.50	126.31	-0.19	[126.31]
C	16	126.10	125.07	-1.03	[125.07]
C	8	123.60	123.33	-0.27	[123.33]
C	14	123.00	123.15	0.15	[123.15]
C	18	122.40	121.90	-0.50	[121.90]
C	9	122.00	122.87	0.87	[122.87]
C	3	118.60	120.14	1.54	[120.14]
C	1	117.80	118.93	1.13	[118.93]
C	11	116.30	115.50	-0.80	[115.50]
C	15	113.40	111.47	-1.93	[111.47]
C	19	32.00	31.89	-0.11	[31.89]

13C chem shifts: RMSD=1.00ppm (MAE=0.80) N=16 {-1.93 1.61}

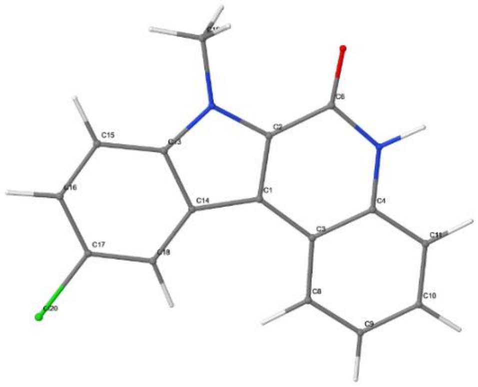

Conformer 1
Energy: -1261.29648 Hartree (Rel: 0.0 kcal/mol)
XYZ coordinates for conf 1:

C	0.49356	0.02790	0.00012
C	0.85166	1.37737	0.00015
C	1.51634	-0.99549	0.00010
C	2.86615	-0.55224	-0.00008
N	3.13964	0.81280	-0.00016
C	2.22579	1.85022	0.00002
O	2.59352	3.03169	-0.00028
C	1.28282	-2.38569	0.00021
C	2.33287	-3.29460	0.00014
C	3.65871	-2.83686	-0.00005
C	3.92501	-1.47393	-0.00016
N	-0.26859	2.18409	0.00020
C	-1.37757	1.36316	0.00011
C	-0.94795	0.00068	0.00012

C	-2.73840	1.70543	-0.00005
C	-3.67492	0.68302	-0.00010
C	-3.25287	-0.66111	-0.00003
C	-1.91628	-1.02203	0.00006
C	-0.28218	3.64448	0.00016
Cl	-4.48432	-1.92822	-0.00014
H	4.11005	1.10856	-0.00038
H	0.26533	-2.75683	0.00038
H	2.12289	-4.35989	0.00024
H	4.48263	-3.54430	-0.00011
H	4.94941	-1.11103	-0.00030
H	-3.06899	2.73825	-0.00021
H	-4.73433	0.91446	-0.00022
H	-1.64431	-2.06960	0.00004
H	-1.31734	3.98380	0.00320
H	0.22569	4.03128	0.88558
H	0.22049	4.03143	-0.88823

Originally assigned (incorrect) structure of 74{2u} (DMSO-d_6)

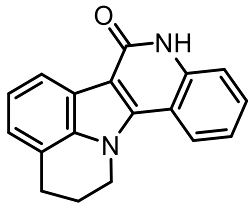

C-nom	iGau	Exp	Calc	diff	1	2
				Rel energy (kcal/mol):	Conf1 0.00	Conf2 0.00
C	6	156.90	159.69	2.79	[159.69	159.69]
C	1	137.20	140.60	3.40	[140.60	140.60]
C	4	135.30	137.75	2.45	[137.75	137.75]
C	13	126.40	135.42	9.02	[135.42	135.42]
C	10	126.10	129.56	3.46	[129.56	129.56]
C	8	124.00	123.76	-0.24	[123.76	123.76]
C	18	123.50	123.15	-0.35	[123.15	123.15]
C	12	123.30	122.79	-0.51	[122.79	122.79]
C	16	122.80	121.85	-0.95	[121.85	121.85]
C	9	121.60	121.54	-0.06	[121.54	121.54]
C	17	120.30	121.21	0.91	[121.21	121.21]
C	15	119.90	118.12	-1.78	[118.12	118.12]
C	11	118.90	115.62	-3.28	[115.62	115.62]
C	3	118.70	115.54	-3.16	[115.54	115.54]
C	2	116.30	108.82	-7.48	[108.82	108.82]
C	21	44.20	44.85	0.65	[44.85	44.85]
C	19	24.70	23.07	-1.63	[23.07	23.07]
C	20	22.70	21.80	-0.90	[21.80	21.80]

13C chem shifts: RMSD=3.37ppm (MAE=2.39) N=18 {-7.48 9.02}

Fractions: 0.500 0.500

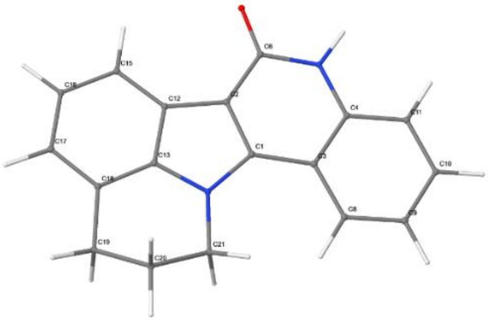

Conformer 1
Energy: -879.13468 Hartree (Rel: 0.0 kcal/mol)
XYZ coordinates for conf 1:

C	0.45287	-0.15115	-0.01913
C	0.02985	1.18945	0.00033
C	1.85607	-0.50211	-0.03958
C	2.76398	0.59282	0.04972
N	2.28728	1.89289	0.12159
C	0.95458	2.29232	0.07545
O	0.65757	3.49448	0.11119
C	2.39854	-1.79968	-0.16996
C	3.76967	-2.01265	-0.17325
C	4.64864	-0.92596	-0.05194
C	4.15114	0.36460	0.05264
C	-1.40504	1.20606	-0.03461
C	-1.79182	-0.15072	-0.06402
N	-0.66404	-0.96211	-0.03844
C	-2.40891	2.19105	-0.04779
C	-3.73744	1.77639	-0.08926

```
C         -4.09038        0.40804       -0.11726
C         -3.11839       -0.58986       -0.11081
C         -3.35163       -2.08267       -0.14469
C         -2.19243       -2.80820        0.56476
C         -0.80753       -2.42355        0.01456
H          2.95901        2.65039        0.17882
H          1.74077       -2.65014       -0.28916
H          4.15759       -3.02134       -0.27556
H          5.72238       -1.08867       -0.05069
H          4.82707        1.21196        0.12931
H         -2.14465        3.24275       -0.02508
H         -4.52830        2.52174       -0.09769
H         -5.14111        0.12889       -0.14448
H         -3.41981       -2.43075       -1.18575
H         -4.30411       -2.33873        0.33239
H         -2.22301       -2.57432        1.63577
H         -2.30145       -3.89307        0.46648
H         -0.03847       -2.83275        0.67197
H         -0.65976       -2.84220       -0.98891

Conformer 2
Energy: -879.13468 Hartree (Rel:  0.0 kcal/mol)
XYZ coordinates for conf 2:
C         -0.45287       -0.15115       -0.01913
C         -0.02985        1.18945        0.00033
C         -1.85607       -0.50211       -0.03958
C         -2.76398        0.59282        0.04972
N         -2.28728        1.89289        0.12159
C         -0.95458        2.29232        0.07545
O         -0.65757        3.49448        0.11119
C         -2.39854       -1.79968       -0.16996
C         -3.76967       -2.01265       -0.17325
C         -4.64864       -0.92596       -0.05194
C         -4.15114        0.36460        0.05264
C          1.40504        1.20606       -0.03461
C          1.79182       -0.15072       -0.06402
N          0.66404       -0.96211       -0.03844
C          2.40891        2.19105       -0.04779
C          3.73744        1.77639       -0.08926
C          4.09038        0.40804       -0.11726
C          3.11839       -0.58986       -0.11081
C          3.35163       -2.08267       -0.14469
C          2.19243       -2.80820        0.56476
C          0.80753       -2.42355        0.01456
H         -2.95901        2.65039        0.17882
H         -1.74077       -2.65014       -0.28916
H         -4.15759       -3.02134       -0.27556
H         -5.72238       -1.08867       -0.05069
H         -4.82707        1.21196        0.12931
H          2.14465        3.24275       -0.02508
H          4.52831        2.52174       -0.09769
H          5.14111        0.12889       -0.14448
H          4.30411       -2.33873        0.33239
H          3.41981       -2.43075       -1.18575
H          2.30145       -3.89307        0.46648
H          2.22301       -2.57432        1.63577
H          0.65976       -2.84220       -0.98891
H          0.03847       -2.83275        0.67197
```

Revised structure of 74{2u}, i.e. 78{2u-rev} (DMSO-d_6)

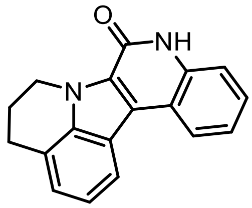

	Conf1	Conf2
Rel energy (kcal/mol):	0.00	0.00

C-nom	iGau	Exp	Calc	diff	1	2
C	6	156.90	157.11	0.21	[157.11	157.11]
C	13	137.20	136.68	-0.52	[136.68	136.67]
C	4	135.30	133.82	-1.48	[133.82	133.83]
C	2	126.40	126.74	0.34	[126.74	126.73]
C	10	126.10	126.20	0.10	[126.20	126.20]
C	8	124.00	123.57	-0.43	[123.57	123.56]
C	15	123.50	123.95	0.45	[123.95	123.95]
C	16	123.30	123.18	-0.12	[123.18	123.17]
C	9	122.80	122.72	-0.08	[122.72	122.71]
C	3	121.60	121.19	-0.41	[121.19	121.19]
C	17	120.30	121.07	0.77	[121.07	121.07]
C	1	119.90	119.47	-0.43	[119.47	119.48]
C	14	118.90	120.72	1.82	[120.72	120.72]
C	18	118.70	119.99	1.29	[119.99	119.99]
C	11	116.30	115.36	-0.94	[115.36	115.36]
C	19	44.20	43.27	-0.93	[43.27	43.27]
C	21	24.70	23.67	-1.03	[23.67	23.67]
C	20	22.70	21.00	-1.70	[21.00	21.00]

13C chem shifts: RMSD=0.90ppm (MAE=0.73) N=18 {-1.70 1.82}

Fractions: 0.500 0.500

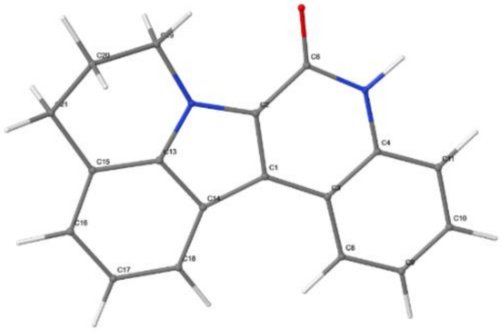

Conformer 1
Energy: -879.13312 Hartree (Rel: 0.0 kcal/mol)
XYZ coordinates for conf 1:

C	-0.53449	0.31464	-0.02313
C	-0.01577	-0.98794	-0.04988
C	-1.96510	0.51896	0.00453
C	-2.78335	-0.64244	0.00185
N	-2.18805	-1.90084	-0.02884
C	-0.83379	-2.18540	-0.05245
O	-0.42330	-3.35416	-0.07369
C	-2.60561	1.77457	0.03462
C	-3.99031	1.87776	0.06133
C	-4.78176	0.71892	0.05956
C	-4.18288	-0.53392	0.03035
N	1.36143	-0.95380	-0.06481
C	1.74527	0.36663	-0.05761
C	0.60183	1.20662	-0.02727
C	3.07181	0.82116	-0.06855
C	3.24124	2.19918	-0.03932

C	2.12828	3.07362	-0.01327
C	0.82138	2.60141	-0.00681
C	2.34979	-2.03600	-0.12991
C	3.66936	-1.55589	0.49477
C	4.16432	-0.22565	-0.11127
H	-2.78936	-2.71766	-0.03153
H	-2.00482	2.67654	0.03692
H	-4.45819	2.85749	0.08408
H	-5.86484	0.79588	0.08112
H	-4.78823	-1.43668	0.02952
H	4.24556	2.61634	-0.03549
H	2.30456	4.14555	0.00637
H	-0.00187	3.30719	0.01546
H	2.50270	-2.32118	-1.17913
H	1.94739	-2.90298	0.39315
H	4.42227	-2.33794	0.35272
H	3.52705	-1.43339	1.57555
H	4.47493	-0.39571	-1.15251
H	5.05422	0.12475	0.42364

Conformer 2
Energy: -879.13312 Hartree (Rel: 0.0 kcal/mol)
XYZ coordinates for conf 2:

C	0.53448	0.31460	-0.02332
C	0.01570	-0.98798	-0.04976
C	1.96507	0.51902	0.00456
C	2.78332	-0.64242	0.00155
N	2.18809	-1.90082	-0.02954
C	0.83374	-2.18539	-0.05314
O	0.42330	-3.35410	-0.07505
C	2.60563	1.77460	0.03543
C	3.99030	1.87774	0.06234
C	4.78177	0.71888	0.06007
C	4.18288	-0.53390	0.03030
N	-1.36148	-0.95377	-0.06467
C	-1.74526	0.36661	-0.05748
C	-0.60190	1.20660	-0.02727
C	-3.07184	0.82117	-0.06857
C	-3.24125	2.19916	-0.03986
C	-2.12828	3.07365	-0.01428
C	-0.82139	2.60140	-0.00757
C	-2.34976	-2.03615	-0.12836
C	-3.66935	-1.55557	0.49590
C	-4.16418	-0.22581	-0.11121
H	2.78931	-2.71767	-0.03349
H	2.00482	2.67656	0.03846
H	4.45827	2.85741	0.08574
H	5.86484	0.79595	0.08177
H	4.78816	-1.43672	0.02928
H	-4.24555	2.61637	-0.03627
H	-2.30460	4.14557	0.00477
H	0.00190	3.30716	0.01410
H	-1.94719	-2.90247	0.39568
H	-2.50278	-2.32264	-1.17723
H	-3.52715	-1.43231	1.57664
H	-4.42228	-2.33767	0.35440
H	-5.05454	0.12491	0.42271
H	-4.47396	-0.39664	-1.15260

Originally assigned (incorrect) structure of 79{2ab} (DMSO-d_6)

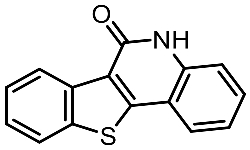

C-nom	iGau	Exp	Calc	diff	Conf1<br>Rel energy (kcal/mol): 0.00<br>1
C	13	158.40	159.00	0.60	[159.00]
C	8	141.80	150.26	8.46	[150.26]
C	5	138.10	136.74	-1.36	[136.74]
C	11	136.40	135.60	-0.80	[135.60]
C	4	136.00	137.25	1.25	[137.25]
C	17	132.70	131.48	-1.22	[131.48]
C	9	129.30	126.77	-2.53	[126.77]
C	2	128.00	125.27	-2.73	[125.27]
C	1	126.40	125.26	-1.14	[125.26]
C	15	126.30	124.67	-1.63	[124.67]
C	6	124.60	124.45	-0.15	[124.45]
C	16	124.10	122.86	-1.24	[122.86]
C	3	123.20	121.23	-1.97	[121.23]
C	10	117.90	118.07	0.17	[118.07]
C	18	117.20	115.54	-1.66	[115.54]

13C chem shifts: RMSD=2.63ppm (MAE=1.79) N=15 {-2.73 8.46}

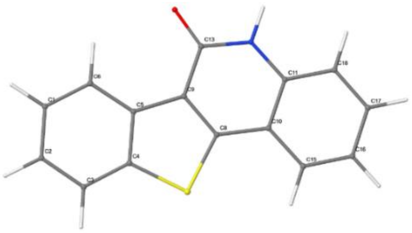

Conformer 1
Energy: -1105.22952 Hartree (Rel: 0.0 kcal/mol)
XYZ coordinates for conf 1:

C	-4.23764	0.77461	0.00007
C	-4.48555	-0.60940	0.00013
C	-3.43221	-1.52140	0.00009
C	-2.12773	-1.02149	0.00001
C	-1.85672	0.36843	-0.00004
C	-2.93811	1.26941	-0.00001
S	-0.64396	-1.97772	-0.00006
C	0.33603	-0.52063	-0.00010
C	-0.43400	0.63025	-0.00014
C	1.77408	-0.50161	-0.00007
C	2.38557	0.77862	0.00001
N	1.58524	1.90938	-0.00004
C	0.19760	1.94642	-0.00028
O	-0.39861	3.02948	0.00010
C	2.59645	-1.64703	-0.00004
C	3.97728	-1.52915	0.00005
C	4.57037	-0.25433	0.00013
C	3.78669	0.89076	0.00012
H	-5.07446	1.46726	0.00009
H	-5.50832	-0.97501	0.00018
H	-3.62174	-2.59055	0.00013
H	-2.73678	2.33382	-0.00006
H	2.03206	2.82021	0.00009
H	2.13626	-2.63120	-0.00008
H	4.59854	-2.41912	0.00007
H	5.65216	-0.15977	0.00021
H	4.24518	1.87564	0.00019

Revised structure of 79{2ab}, i.e. 80{2ab-rev} (DMSO-d_6)

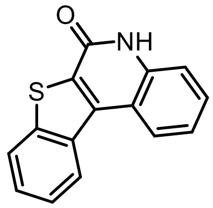

C-nom	iGau	Exp	Calc	diff	Conf1<br>Rel energy (kcal/mol): 0.00<br>1
C	9	158.40	156.78	-1.62	[156.78]
C	1	141.80	141.57	-0.23	[141.57]
C	4	138.10	138.45	0.35	[138.45]
C	7	136.40	137.08	0.68	[137.08]
C	3	136.00	136.34	0.34	[136.34]
C	5	132.70	132.99	0.29	[132.99]
C	13	129.30	129.57	0.27	[129.57]
C	16	128.00	127.10	-0.90	[127.10]
C	18	126.40	125.90	-0.50	[125.90]
C	17	126.30	125.15	-1.15	[125.15]
C	11	124.60	124.54	-0.06	[124.54]
C	12	124.10	122.90	-1.20	[122.90]
C	15	123.20	122.64	-0.56	[122.64]
C	6	117.90	119.77	1.87	[119.77]
C	14	117.20	116.39	-0.81	[116.39]

13C chem shifts: RMSD=0.89ppm (MAE=0.72) N=15 {-1.62 1.87}

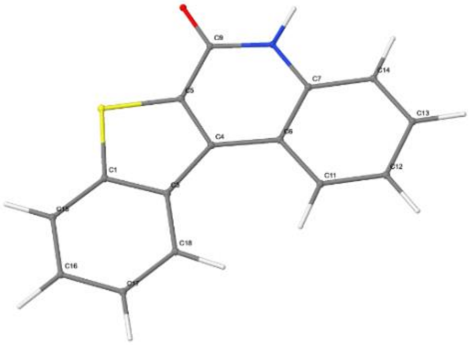

Conformer 1
Energy: -1105.22290 Hartree (Rel: 0.0 kcal/mol)
XYZ coordinates for conf 1:

C	2.36918	0.32897	-0.00084
S	1.72418	1.96009	-0.00141
C	1.35225	-0.66641	0.00030
C	0.01860	-0.06589	-0.00011
C	0.10486	1.31728	-0.00040
C	-1.30853	-0.67188	-0.00035
C	-2.43003	0.20571	0.00099
N	-2.23779	1.58045	0.00174
C	-1.02252	2.23003	0.00053
O	-0.93374	3.46328	0.00047
C	-1.58067	-2.05735	-0.00241
C	-2.87842	-2.54818	-0.00226
C	-3.96604	-1.66302	-0.00013
C	-3.74284	-0.29502	0.00134
C	3.73183	0.01190	-0.00097
C	4.10327	-1.32646	0.00040
C	3.12220	-2.33249	0.00222
C	1.77017	-2.01530	0.00225
H	-3.05341	2.18374	0.00260

H	-0.76679	-2.76683	-0.00480
H	-3.04612	-3.62073	-0.00389
H	-4.98335	-2.04269	0.00011
H	-4.57608	0.40260	0.00258
H	4.48040	0.79843	-0.00197
H	5.15560	-1.59452	0.00034
H	3.42242	-3.37607	0.00375
H	1.05509	-2.82591	0.00439

Originally assigned (incorrect) structure of 84{8} ($CDCl_3$)

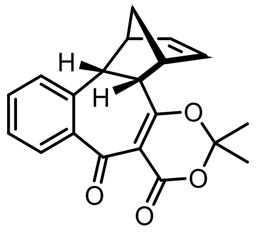

					**Conf1**
	Rel energy (kcal/mol):				0.00

**C-nom**	**iGau**	**Exp**	**Calc**	**diff**	**1**
C	7	189.50	196.99	7.49	[196.99]
C	9	165.30	169.76	4.46	[169.76]
C	12	148.90	156.78	7.88	[156.78]
C	4	147.50	143.43	-4.07	[143.43]
C	5	135.10	137.79	2.69	[137.79]
C	22	134.10	136.36	2.26	[136.36]
C	21	132.00	135.99	3.99	[135.99]
C	1	130.80	130.84	0.04	[130.84]
C	3	130.30	129.72	-0.58	[129.72]
C	6	127.63	127.92	0.29	[127.92]
C	2	127.59	125.98	-1.61	[125.98]
C	8	112.10	112.65	0.55	[112.65]
C	14	106.10	106.59	0.49	[106.59]
C	10	65.00	48.08	-16.92	[48.08]
C	11	52.40	47.52	-4.88	[47.52]
C	23	46.70	46.64	-0.06	[46.64]
C	24	43.40	46.07	2.67	[46.07]
C	20	41.20	45.82	4.62	[45.82]
C	19	28.40	26.47	-1.93	[26.47]
C	18	26.70	21.58	-5.12	[21.58]

13C chem shifts: RMSD=5.27ppm (MAE=3.63) N=20 {-16.92 7.88}

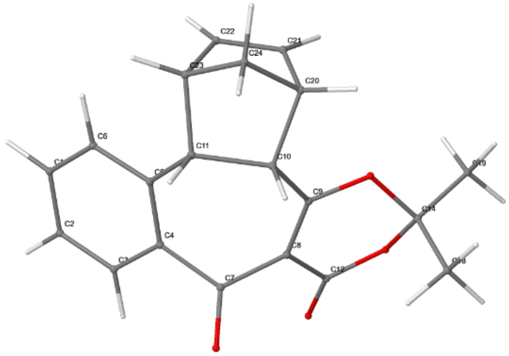

Conformer 1
Energy: -1073.80842 Hartree (Rel: 0.0 kcal/mol)
XYZ coordinates for conf 1:

C	-4.19272	-0.99100	-0.92644
C	-3.74160	-2.29263	-0.71281
C	-2.52085	-2.48515	-0.07374
C	-1.73644	-1.39434	0.33742
C	-2.19870	-0.07122	0.16092
C	-3.43656	0.09414	-0.48048
C	-0.38914	-1.76873	0.87101
C	0.80637	-1.06688	0.31169
C	0.95979	0.28357	0.30480
C	0.03415	1.30197	0.91092
C	-1.54437	1.13309	0.83109
C	1.80211	-1.91237	-0.36668
O	2.90661	-1.25289	-0.85195

C	3.26485	0.00608	-0.28195
O	2.09434	0.85099	-0.15487
O	-0.24350	-2.73596	1.60615
O	1.67933	-3.09047	-0.62285
C	3.89742	-0.18366	1.09520
C	4.17724	0.69591	-1.27921
C	0.26024	2.76172	0.35922
C	-0.12392	2.74546	-1.11344
C	-1.45653	2.61343	-1.17886
C	-1.97572	2.53733	0.25112
C	-0.97033	3.46318	0.96489
H	-1.89216	1.10107	1.87198
H	0.30646	1.31270	1.97590
H	-5.14043	-0.81279	-1.42720
H	-4.32759	-3.14536	-1.04303
H	-2.13672	-3.48602	0.09413
H	-3.83871	1.09031	-0.62244
H	4.77764	-0.82580	1.00297
H	3.19845	-0.65291	1.79303
H	4.20564	0.78453	1.49960
H	5.08365	0.10165	-1.42092
H	3.66531	0.80065	-2.23930
H	4.45567	1.68603	-0.90855
H	1.24271	3.16510	0.60323
H	0.58344	2.73409	-1.93532
H	-2.05948	2.47465	-2.06962
H	-3.03178	2.76520	0.40318
H	-1.01936	3.40937	2.05898
H	-1.05867	4.50483	0.64354

Revised structure of 84{8}, i.e 85{8-rev} ($CDCl_3$)

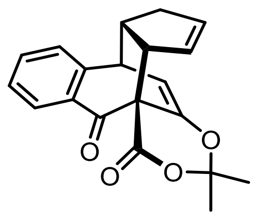

						Conf1	Conf2	
	Rel energy (kcal/mol):					0.00	0.51	
**C-nom**	**iGau**	**Exp**	**Calc**	**diff**		**1**	**2**	
C-C	7	189.50	191.79	2.29	[	192.40	190.33	]
C-C	12	165.30	164.76	-0.54	[	164.87	164.51	]
C-C	5	148.90	149.70	0.80	[	150.14	148.64	]
C-C	9	147.50	147.74	0.24	[	147.83	147.54	]
C-C	4	130.80	130.77	-0.03	[	130.53	131.33	]
C-C	14	106.10	105.93	-0.17	[	105.96	105.87	]
C-C	8	65.00	65.62	0.62	[	66.26	64.08	]
C-CH	21	135.10	135.41	0.31	[	135.09	136.18	]
C-CH	1	134.10	133.82	-0.28	[	134.02	133.35	]
C-CH	3	132.00	132.74	0.74	[	132.78	132.65	]
C-CH	20	130.30	131.31	1.01	[	130.94	132.20	]
C-CH	6	127.63	127.33	-0.30	[	127.44	127.07	]
C-CH	2	127.59	126.47	-1.12	[	126.40	126.65	]
C-CH	10	112.10	112.37	0.27	[	110.61	116.57	]
C-CH	24	52.40	54.66	2.26	[	55.70	52.19	]
C-CH	11	46.70	47.53	0.83	[	47.93	46.57	]
C-CH	23	43.40	45.60	2.20	[	45.61	45.59	]
C-CH2	22	41.20	40.79	-0.41	[	41.00	40.29	]
C-CH3	19	28.40	27.57	-0.83	[	27.66	27.37	]
C-CH3	18	26.70	25.54	-1.16	[	25.06	26.70	]

13C chem shifts: RMSD=1.06ppm (MAE=0.82) N=20 {-1.16 2.29}

Fractions: 0.704 0.296

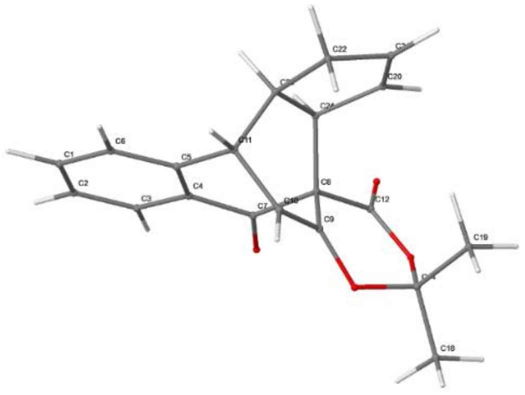

Conformer 1
Energy: -1073.82608 Hartree (Rel: 0.0 kcal/mol)
XYZ coordinates for conf 1:

C	4.56620	-0.92814	-0.51940
C	4.11972	-1.91188	0.36672
C	2.79484	-1.89934	0.78633
C	1.89903	-0.91016	0.33977
C	2.34877	0.08654	-0.55713
C	3.68598	0.05608	-0.96993
C	0.49942	-1.01520	0.85203
C	-0.55267	0.08628	0.52801
C	-0.76954	0.16944	-0.97206
C	0.19310	0.67793	-1.74306

C	1.44146	1.19698	-1.05881
C	-1.82407	-0.30645	1.29032
O	-2.81456	-0.91982	0.60699
C	-2.72041	-1.20471	-0.81485
O	-1.99536	-0.17649	-1.47676
O	0.15745	-1.97364	1.53399
O	-1.97869	-0.07807	2.46780
C	-2.08676	-2.57762	-1.02085
C	-4.14382	-1.12184	-1.34157
C	-1.13593	2.53353	1.10610
C	-0.87900	3.59337	0.33775
C	0.43667	3.48403	-0.38291
C	1.03852	2.13528	0.11854
C	-0.03799	1.49162	1.05518
H	2.01766	1.78995	-1.77600
H	0.06815	0.76467	-2.81729
H	5.59735	-0.92703	-0.86203
H	4.79841	-2.68037	0.72463
H	2.42231	-2.65311	1.47110
H	4.03997	0.81572	-1.66218
H	-2.68690	-3.33894	-0.51503
H	-2.05362	-2.80535	-2.09020
H	-1.07351	-2.62190	-0.61802
H	-4.15364	-1.32659	-2.41565
H	-4.77314	-1.85623	-0.83263
H	-4.54479	-0.12066	-1.16383
H	-2.01720	2.40439	1.72460
H	-1.53217	4.45595	0.23431
H	0.29781	3.49345	-1.47308
H	1.93831	2.33883	0.70651
H	1.10177	4.32668	-0.15148
H	0.36137	1.31028	2.05917

Conformer 2
Energy: -1073.82526 Hartree (Rel: 0.5 kcal/mol)
XYZ coordinates for conf 2:

C	4.74794	-0.40641	-0.57285
C	4.42964	-1.53822	0.18047
C	3.10607	-1.76598	0.54265
C	2.08642	-0.87091	0.17499
C	2.40655	0.27639	-0.59011
C	3.74234	0.48511	-0.95024
C	0.70229	-1.23970	0.61765
C	-0.44500	-0.19013	0.51597
C	-0.68746	-0.04219	-0.97477
C	0.17486	0.65708	-1.71429
C	1.35431	1.29572	-1.00685
C	-1.63899	-0.77273	1.27514
O	-2.69997	-1.21929	0.56924
C	-2.90398	-0.89773	-0.83531
O	-1.66668	-0.82429	-1.53005
O	0.45723	-2.36233	1.03255
O	-1.65151	-0.86424	2.48136
C	-3.65620	-2.07843	-1.42379
C	-3.66541	0.42165	-0.92177
C	-1.20962	2.08062	1.48730
C	-1.18206	3.22666	0.80218
C	0.01242	3.32770	-0.10849
C	0.86006	2.06943	0.24467
C	-0.02886	1.18213	1.17696
H	1.83325	2.01112	-1.68302
H	0.06952	0.72198	-2.79238
H	5.77548	-0.21822	-0.87191
H	5.20499	-2.23711	0.48010
H	2.83255	-2.64118	1.12188
H	3.99626	1.35881	-1.54523
H	-4.60687	-2.21331	-0.90181
H	-3.05669	-2.98637	-1.31844
H	-3.85348	-1.89972	-2.48434
H	-3.07691	1.23962	-0.49543
H	-4.60808	0.34582	-0.37293

H	-3.88139	0.65233	-1.96923
H	-1.97076	1.79739	2.20699
H	-1.92916	4.01237	0.87899
H	-0.29450	3.33508	-1.16385
H	1.73950	2.38693	0.81319
H	0.58200	4.25162	0.05486
H	0.49988	0.92361	2.10084

Originally assigned correct structure of 86{3k} (Acetone-d_6) additional linear scaling for acetone - crmsd

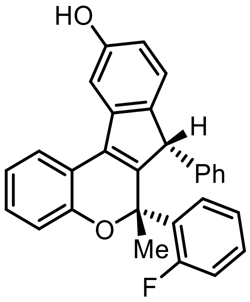

						Conf1	Conf2	Conf3	Conf4	
Rel energy (kcal/mol):						0.00	0.08	0.87	0.89	
**C-nom**	**iGau**	**Exp**	**Calc**	**diff**		**1**	**2**	**3**	**4**	
C	48	161.47	160.61	-0.86	[	159.18	159.18	162.88	161.41	]
C	16	157.80	155.29	-2.51	[	154.41	154.37	154.67	154.57	]
C	4	153.90	153.57	-0.33	[	152.40	152.43	154.72	153.22	]
C	9	146.70	145.81	-0.89	[	145.04	145.08	143.22	145.91	]
C	13	142.30	142.02	-0.28	[	141.10	140.77	142.00	142.19	]
C	12	141.80	142.30	0.50	[	141.47	141.55	140.78	141.70	]
C	19	140.00	139.55	-0.45	[	138.84	138.72	138.11	138.65	]
C	10	132.60	136.36	3.76	[	135.95	135.79	134.22	133.85	]
C	26	130.70	130.97	0.27	[	129.95	129.92	131.92	129.90	]
C	29	130.60	129.80	-0.80	[	128.75	128.77	129.60	130.02	]
C	2	130.10	130.34	0.24	[	129.56	129.55	129.23	129.32	]
C	27	129.80	128.46	-1.34	[	127.77	127.74	126.43	127.74	]
C	21	129.70	128.35	-1.35	[	127.56	127.57	127.57	126.99	]
C	23	128.70	128.35	-0.35	[	127.56	127.57	127.57	126.99	]
C	20	128.70	128.13	-0.57	[	127.05	127.08	127.76	128.68	]
C	24	127.10	128.13	1.03	[	127.05	127.08	127.76	128.68	]
C	14	125.70	126.37	0.67	[	125.34	125.78	124.97	126.02	]
C	22	124.40	126.38	1.98	[	125.49	125.51	125.88	125.60	]
C	6	123.88	125.00	1.12	[	124.27	124.04	124.19	124.10	]
C	28	123.85	123.42	-0.43	[	122.73	122.73	122.13	121.82	]
C	5	122.00	121.81	-0.19	[	121.31	121.30	119.19	120.02	]
C	1	121.20	121.51	0.31	[	120.97	120.94	118.95	120.11	]
C	3	117.50	118.27	0.77	[	117.82	117.90	114.51	116.90	]
C	47	116.80	116.77	-0.03	[	115.95	115.96	115.96	115.74	]
C	15	113.70	113.00	-0.70	[	111.82	112.65	111.53	112.53	]
C	17	109.40	108.72	-0.68	[	108.11	107.48	108.67	107.69	]
C	8	80.80	81.41	0.61	[	80.63	80.64	80.06	80.86	]
C	11	55.50	56.66	1.16	[	55.79	55.89	56.08	55.82	]
C	25	26.50	26.39	-0.11	[	25.19	25.16	29.83	24.83	]
**13C chem shifts: CRMSD=1.14ppm (CMAE=0.84) N=29 {-2.51 3.76}**										
Fractions:						0.431	0.374	0.099	0.096	

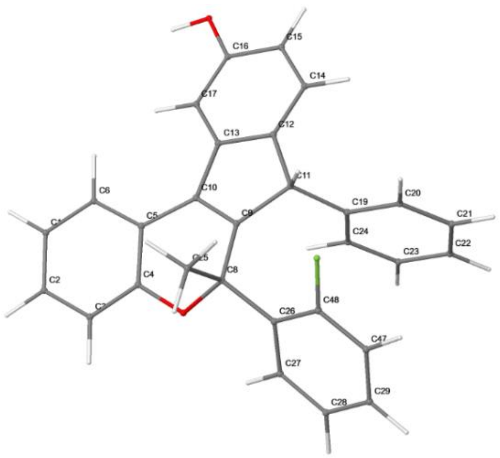

Conformer 1
Energy: -1367.99482 Hartree (Rel: 0.0 kcal/mol)
XYZ coordinates for conf 1:

C	4.01187	3.23902	-0.56331
C	3.09263	4.24618	-0.87098
C	1.72273	3.99785	-0.77500
C	1.27792	2.73496	-0.38324
C	2.18599	1.68615	-0.11889
C	3.55901	1.97267	-0.19287
O	-0.07305	2.53525	-0.31358
C	-0.57304	1.55157	0.64028
C	0.26083	0.28797	0.48285
C	1.58578	0.38686	0.18234
C	-0.13566	-1.15294	0.76930
C	1.19529	-1.87201	0.59769
C	2.19866	-0.95733	0.21652
C	1.48269	-3.22394	0.71954
C	2.78282	-3.67806	0.46926
C	3.77610	-2.77176	0.07709
C	3.49206	-1.40515	-0.05832
O	5.05900	-3.16375	-0.19708
C	-1.20923	-1.76654	-0.13054
C	-2.20327	-2.58524	0.41887
C	-3.14349	-3.21669	-0.40098
C	-3.10209	-3.03414	-1.78399
C	-2.11364	-2.21608	-2.34142
C	-1.17423	-1.59115	-1.52161
C	-0.39686	2.15385	2.05385
C	-2.06303	1.40706	0.29774
C	-2.61214	1.99557	-0.85128
C	-3.97615	1.91360	-1.13792
C	-4.83640	1.23590	-0.27318
H	5.07845	3.43689	-0.61286
H	3.43924	5.23238	-1.16672
H	0.99018	4.77105	-0.98497
H	4.28195	1.20630	0.05894
H	-0.48441	-1.23743	1.80680
H	0.70846	-3.93412	0.99803
H	3.02167	-4.73437	0.56824
H	4.28685	-0.74842	-0.38959
H	5.13322	-4.12455	-0.07683
H	-2.24813	-2.72330	1.49626
H	-3.90854	-3.84746	0.04425
H	-3.83347	-3.52134	-2.42312
H	-2.07401	-2.06575	-3.41716
H	-0.40787	-0.95819	-1.96156
H	0.66139	2.35873	2.23960
H	-0.95859	3.09108	2.12636
H	-0.75705	1.46557	2.81903
C	-4.32014	0.63396	0.87416
C	-2.95896	0.73006	1.13056
H	-1.95259	2.53037	-1.52251
H	-4.36322	2.38402	-2.03696
H	-5.89913	1.16989	-0.48644
H	-4.94848	0.08886	1.57076
F	-2.49887	0.10830	2.25294

Conformer 2
Energy: -1367.99353 Hartree (Rel: 0.1 kcal/mol)
XYZ coordinates for conf 2:

C	-4.03823	-3.20745	-0.54859
C	-3.12763	-4.21945	-0.86599
C	-1.75553	-3.98058	-0.77781
C	-1.29981	-2.72195	-0.38480
C	-2.19918	-1.66756	-0.11286
C	-3.57451	-1.94522	-0.17741
O	0.05263	-2.53156	-0.32049
C	0.56291	-1.55605	0.63662
C	-0.26151	-0.28549	0.48614
C	-1.58784	-0.37338	0.18792

C	0.14509	1.15191	0.77583
C	-1.18027	1.88121	0.60511
C	-2.18859	0.97601	0.22216
C	-1.45702	3.23759	0.72647
C	-2.74946	3.70293	0.47124
C	-3.74964	2.80564	0.07333
C	-3.47887	1.43660	-0.05849
O	-4.98838	3.32876	-0.18333
C	1.22288	1.76091	-0.12214
C	2.22307	2.57050	0.42952
C	3.16698	3.19846	-0.38884
C	3.12300	3.02154	-1.77250
C	2.12830	2.21261	-2.33219
C	1.18530	1.59114	-1.51390
C	0.38642	-2.16334	2.04800
C	2.05290	-1.42154	0.29036
C	2.59463	-2.01041	-0.86199
C	3.95846	-1.93749	-1.15196
C	4.82594	-1.26912	-0.28716
H	-5.10635	-3.39858	-0.59063
H	-3.48268	-5.20239	-1.16254
H	-1.02950	-4.75813	-0.99430
H	-4.29097	-1.17591	0.08442
H	0.49402	1.23103	1.81375
H	-0.67646	3.93963	1.00801
H	-2.99542	4.75625	0.56315
H	-4.26739	0.77194	-0.39538
H	-5.58791	2.61485	-0.45535
H	2.26965	2.70437	1.50735
H	3.93669	3.82230	0.05806
H	3.85707	3.50620	-2.41047
H	2.08655	2.06688	-3.40849
H	0.41393	0.96545	-1.95551
H	-0.67282	-2.36018	2.23671
H	0.94054	-3.10554	2.11431
H	0.75509	-1.48149	2.81488
C	4.31714	-0.66720	0.86354
C	2.95591	-0.75389	1.12302
H	1.92938	-2.53810	-1.53326
H	4.33972	-2.40773	-2.05357
H	5.88858	-1.21027	-0.50298
H	4.95126	-0.12903	1.56027
F	2.50311	-0.13230	2.24847

Conformer 3
Energy: -1367.99495 Hartree (Rel: 0.9 kcal/mol)
XYZ coordinates for conf 3:

C	3.17748	3.61924	-0.83192
C	2.29487	4.56488	-0.30496
C	1.14823	4.14988	0.37301
C	0.89099	2.78690	0.53589
C	1.78649	1.81334	0.04293
C	2.91805	2.25905	-0.65766
O	-0.24161	2.46853	1.22551
C	-0.81356	1.11825	1.24666
C	0.20755	0.09416	0.79983
C	1.42665	0.41819	0.29523
C	0.06820	-1.40798	1.01015
C	1.42003	-1.90553	0.52736
C	2.22397	-0.81815	0.12437
C	1.90555	-3.20397	0.47909
C	3.21507	-3.43450	0.04188
C	4.02347	-2.35493	-0.33410
C	3.53753	-1.03968	-0.29416
O	5.31594	-2.51950	-0.75515
C	-1.11305	-2.09190	0.32761
C	-1.99130	-2.89330	1.06601
C	-3.05233	-3.55706	0.44093
C	-3.24572	-3.42552	-0.93435
C	-2.37187	-2.62717	-1.68118
C	-1.31422	-1.96886	-1.05573

C	-1.14821	0.89809	2.73196
C	-2.07728	1.16221	0.36544
C	-3.37106	0.97364	0.87327
C	-4.50253	1.06108	0.05831
C	-4.36714	1.34928	-1.29983
H	4.06189	3.93483	-1.37727
H	2.48866	5.62671	-0.42961
H	0.43937	4.86591	0.77740
H	3.59622	1.53724	-1.09484
H	-0.01753	-1.60916	2.08829
H	1.28164	-4.04178	0.77951
H	3.60897	-4.44730	0.00063
H	4.21286	-0.24210	-0.57622
H	5.54074	-3.46405	-0.73578
H	-1.84477	-3.00303	2.13824
H	-3.72401	-4.17471	1.03127
H	-4.06916	-3.93905	-1.42316
H	-2.51598	-2.51803	-2.75286
H	-0.64244	-1.34501	-1.63972
H	-0.21806	0.89999	3.30648
H	-1.78373	1.70873	3.09955
H	-1.65753	-0.05470	2.89808
C	-3.09437	1.54332	-1.83838
C	-1.99020	1.44391	-1.00322
H	-3.50736	0.75117	1.92488
H	-5.48682	0.90676	0.48998
H	-5.24132	1.42254	-1.93988
H	-2.94238	1.76678	-2.88930
F	-0.76904	1.62942	-1.56281

Conformer 4
Energy: -1367.99356 Hartree (Rel: 0.9 kcal/mol)
XYZ coordinates for conf 4:

C	-4.01893	-3.14368	-0.59750
C	-3.12330	-4.20842	-0.72685
C	-1.76081	-4.00603	-0.50581
C	-1.29289	-2.73411	-0.16995
C	-2.17317	-1.63174	-0.08117
C	-3.54318	-1.87184	-0.27782
O	0.05254	-2.60325	0.02340
C	0.55424	-1.51054	0.86093
C	-0.25899	-0.25710	0.59749
C	-1.56074	-0.33453	0.21202
C	0.13087	1.16946	0.96688
C	-1.16899	1.91168	0.69892
C	-2.15002	1.02273	0.21681
C	-1.44261	3.26811	0.82627
C	-2.70530	3.75303	0.47675
C	-3.67651	2.87417	-0.02072
C	-3.40814	1.50505	-0.15871
O	-4.88549	3.41436	-0.36760
C	1.31543	1.79642	0.23160
C	2.43470	2.24530	0.94172
C	3.50025	2.86598	0.28206
C	3.45504	3.04972	-1.10046
C	2.33841	2.60783	-1.81861
C	1.27799	1.98932	-1.15792
C	0.36448	-1.93662	2.32972
C	2.02578	-1.40350	0.45786
C	3.08250	-1.54190	1.36676
C	4.41609	-1.48114	0.95344
C	4.72195	-1.28883	-0.39386
H	-5.08341	-3.30012	-0.74459
H	-3.48389	-5.20112	-0.98154
H	-1.04799	-4.82179	-0.57727
H	-4.25214	-1.06193	-0.16006
H	0.36477	1.19805	2.04223
H	-0.68151	3.95486	1.18750
H	-2.94880	4.80665	0.57126
H	-4.17314	0.85930	-0.57613
H	-5.46756	2.71108	-0.69887

H	2.47572	2.10824	2.01975
H	4.36083	3.20723	0.85143
H	4.27991	3.53427	-1.61588
H	2.29328	2.74856	-2.89545
H	0.41440	1.64916	-1.72207
H	-0.70113	-2.09232	2.51958
H	0.89565	-2.87164	2.53268
H	0.72134	-1.16653	3.02022
C	3.69063	-1.14696	-1.32398
C	2.37562	-1.20400	-0.88323
H	2.86897	-1.69888	2.41772
H	5.21046	-1.59082	1.68548
H	5.75544	-1.24549	-0.72470
H	3.88711	-0.98895	-2.37927
F	1.39700	-1.04153	-1.80382

Originally assigned (incorrect) structure of 87{3a} ($CDCl_3$)

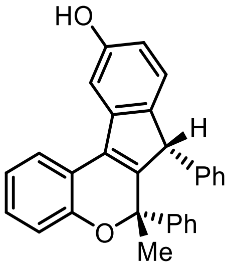

	Conf1	Conf2	Conf3	Conf4
Rel energy (kcal/mol):	0.00	0.10	0.66	0.77

C-nom	iGau	Exp	Calc	diff	1	2	3	4
C	16	154.10	154.83	0.73	[154.94	154.91	154.54	154.54]
C	4	153.30	153.51	0.21	[153.46	153.52	153.54	153.63]
C	9	146.70	146.38	-0.32	[146.38	146.43	146.02	146.69]
C	13	144.80	142.10	-2.70	[142.43	141.89	141.83	141.89]
C	12	141.20	140.82	-0.38	[140.46	140.47	141.90	141.97]
C	26	140.40	139.27	-1.13	[138.76	138.56	141.31	140.90]
C	19	138.40	137.37	-1.03	[137.05	136.86	138.63	138.59]
C	10	135.60	134.83	-0.77	[134.96	134.75	135.04	134.34]
C	2	129.30	128.79	-0.51	[128.73	128.72	129.01	128.96]
C	20	129.30	128.38	-0.92	[128.68	128.69	127.36	127.58]
C	24	128.30	128.38	0.08	[128.68	128.69	127.36	127.58]
C	21	128.30	127.07	-1.23	[127.14	127.18	126.83	126.80]
C	23	128.00	127.07	-0.93	[127.14	127.18	126.83	126.80]
C	29	128.00	126.94	-1.06	[127.12	127.15	126.27	126.41]
C	28	127.60	126.84	-0.76	[126.95	126.96	126.56	126.37]
C	47	127.30	126.84	-0.46	[126.95	126.96	126.56	126.37]
C	27	126.50	126.08	-0.42	[125.79	125.83	126.77	127.12]
C	48	126.40	126.08	-0.32	[125.79	125.83	126.77	127.12]
C	22	125.50	125.49	-0.01	[125.71	125.76	124.77	124.72]
C	14	125.50	125.27	-0.23	[124.88	125.55	125.19	125.91]
C	6	123.30	123.68	0.38	[123.84	123.42	123.95	123.56]
C	5	121.80	121.06	-0.74	[121.18	121.26	120.52	120.66]
C	1	120.90	119.74	-1.16	[119.72	119.59	120.06	119.89]
C	3	117.40	117.22	-0.18	[117.08	117.24	117.37	117.48]
C	15	114.00	111.66	-2.34	[111.04	112.40	110.97	112.42]
C	17	112.80	107.52	-5.28	[108.03	106.93	108.06	106.88]
C	8	82.00	79.67	-2.33	[78.91	78.97	81.97	81.88]
C	11	47.50	55.97	8.47	[56.13	56.25	55.34	55.30]
C	25	29.40	25.42	-3.98	[25.78	25.76	24.54	24.13]

13C chem shifts: RMSD=2.24ppm (MAE=1.35) N=29 {-5.28 8.47}

Fractions: 0.408 0.347 0.133 0.112

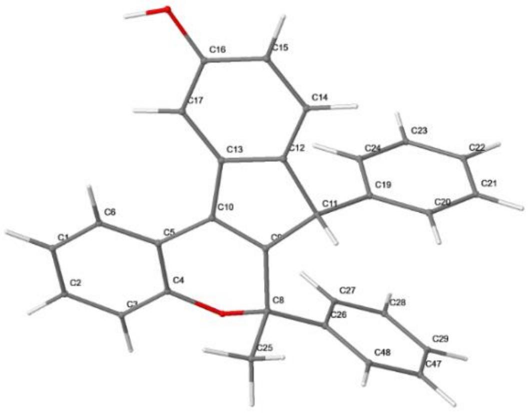

Conformer 1
Energy: -1268.75771 Hartree (Rel: 0.0 kcal/mol)

XYZ coordinates for conf 1:

C	2.14581	4.18557	-0.84931
C	1.16376	4.91090	-0.16969
C	0.20629	4.24735	0.59875
C	0.24175	2.85566	0.69990
C	1.25149	2.10611	0.05417
C	2.18439	2.79534	-0.73579
O	-0.69589	2.25900	1.49194
C	-1.11178	0.87220	1.21579
C	0.10313	0.07361	0.79564
C	1.21358	0.66063	0.27688
C	0.30656	-1.42258	1.00262
C	1.72268	-1.60717	0.48164
C	2.25938	-0.36521	0.07904
C	2.48652	-2.76264	0.40698
C	3.80491	-2.68803	-0.05798
C	4.34553	-1.45161	-0.43215
C	3.57937	-0.27901	-0.36434
O	5.63335	-1.32057	-0.87824
C	-0.72682	-2.35454	0.37553
C	-1.49455	-3.20486	1.18040
C	-2.42891	-4.07857	0.61520
C	-2.60392	-4.11385	-0.76813
C	-1.83751	-3.27276	-1.58186
C	-0.90689	-2.40356	-1.01494
C	-1.62589	0.39425	2.57970
C	-2.21074	0.92891	0.13590
C	-1.86414	1.01894	-1.22145
C	-2.84215	1.15015	-2.20808
C	-4.19337	1.18472	-1.85848
H	2.87861	4.69769	-1.46587
H	1.12969	5.99398	-0.24959
H	-0.57715	4.78888	1.11972
H	2.93510	2.23931	-1.28532
H	0.30300	-1.62200	2.08533
H	2.07330	-3.72250	0.70601
H	4.41389	-3.58708	-0.12328
H	4.04779	0.65682	-0.64341
H	6.06312	-2.19096	-0.87055
H	-1.35783	-3.18898	2.25953
H	-3.01455	-4.73058	1.25793
H	-3.32850	-4.79149	-1.21149
H	-1.96500	-3.29560	-2.66093
H	-0.31094	-1.75831	-1.65432
H	-2.35773	1.10063	2.97884
H	-0.79239	0.34170	3.28564
H	-2.08998	-0.59244	2.50075
C	-4.55361	1.09206	-0.51342
C	-3.57179	0.97031	0.47236
H	-0.82049	0.98150	-1.51343
H	-2.54574	1.21972	-3.25138
H	-4.95690	1.28016	-2.62579
H	-5.60142	1.11386	-0.22570
H	-3.88405	0.90431	1.50859

Conformer 2
Energy: -1268.75787 Hartree (Rel: 0.1 kcal/mol)
XYZ coordinates for conf 2:

C	2.16197	4.16769	-0.85075
C	1.18944	4.89767	-0.16243
C	0.23241	4.23836	0.61017
C	0.25901	2.84614	0.70739
C	1.26069	2.09177	0.05461
C	2.19208	2.77698	-0.74049
O	-0.67854	2.25266	1.50141
C	-1.10690	0.87074	1.21693
C	0.10219	0.06477	0.79434
C	1.21561	0.64630	0.27560
C	0.29892	-1.43255	0.99865
C	1.71413	-1.62316	0.47771
C	2.25576	-0.38500	0.07829

C	2.47458	-2.78371	0.40343
C	3.79223	-2.71644	-0.05655
C	4.34039	-1.48176	-0.42814
C	3.57983	-0.30609	-0.36239
O	5.64185	-1.48049	-0.85286
C	-0.73982	-2.35841	0.37135
C	-1.51585	-3.20091	1.17650
C	-2.45608	-4.06823	0.61125
C	-2.62867	-4.10501	-0.77235
C	-1.85383	-3.27199	-1.58633
C	-0.91744	-2.40902	-1.01935
C	-1.62695	0.39002	2.57756
C	-2.20413	0.94397	0.13636
C	-1.85693	1.02130	-1.22151
C	-2.83284	1.16894	-2.20803
C	-4.18268	1.23302	-1.85763
H	2.89308	4.67671	-1.47191
H	1.16186	5.98111	-0.23951
H	-0.54449	4.78372	1.13687
H	2.93348	2.21684	-1.29871
H	0.29540	-1.63324	2.08115
H	2.05465	-3.74107	0.70116
H	4.41007	-3.60620	-0.12544
H	4.03896	0.63928	-0.63350
H	5.90344	-0.57261	-1.07625
H	-1.38092	-3.18416	2.25583
H	-3.04800	-4.71434	1.25417
H	-3.35759	-4.77794	-1.21572
H	-1.97898	-3.29647	-2.66564
H	-0.31453	-1.77049	-1.65898
H	-2.35161	1.10143	2.98073
H	-0.79477	0.32423	3.28394
H	-2.10129	-0.59120	2.49181
C	-4.54369	1.15307	-0.51188
C	-3.56389	1.01481	0.47359
H	-0.81457	0.96093	-1.51405
H	-2.53590	1.22799	-3.25183
H	-4.94469	1.34130	-2.62473
H	-5.59059	1.19768	-0.22352
H	-3.87666	0.95955	1.51029

Conformer 3
Energy: -1268.75893 Hartree (Rel: 0.7 kcal/mol)
XYZ coordinates for conf 3:

C	-4.04686	-3.05943	-0.67096
C	-3.17530	-4.12905	-0.89352
C	-1.80216	-3.96240	-0.71252
C	-1.30148	-2.71977	-0.32114
C	-2.15840	-1.61044	-0.13948
C	-3.53881	-1.81506	-0.29882
O	0.05251	-2.61076	-0.17043
C	0.57276	-1.60872	0.76029
C	-0.19318	-0.31458	0.54447
C	-1.50566	-0.34193	0.18489
C	0.26233	1.09671	0.88819
C	-1.02588	1.88338	0.68713
C	-2.05207	1.03159	0.22664
C	-1.25173	3.24364	0.83845
C	-2.51397	3.77012	0.54099
C	-3.52809	2.92793	0.06903
C	-3.30519	1.55366	-0.09778
O	-4.77410	3.39331	-0.25671
C	1.40025	1.68656	0.05338
C	2.49260	2.29962	0.67573
C	3.49805	2.91008	-0.08072
C	3.42100	2.91370	-1.47341
C	2.33223	2.30260	-2.10460
C	1.33048	1.69797	-1.34751
C	0.31815	-2.12622	2.19128
C	2.06567	-1.52059	0.42669
C	2.48973	-1.63301	-0.90514

C	3.84308	-1.55654	-1.23198
C	4.79970	-1.36192	-0.23245
H	-5.11848	-3.19144	-0.78749
H	-3.56343	-5.09959	-1.18995
H	-1.10671	-4.78348	-0.85643
H	-4.22666	-0.99946	-0.11261
H	0.57402	1.13288	1.94304
H	-0.45740	3.90446	1.17572
H	-2.70608	4.83387	0.66290
H	-4.11304	0.95082	-0.49310
H	-4.80312	4.35296	-0.11321
H	2.56039	2.29988	1.76124
H	4.33942	3.38030	0.42174
H	4.20094	3.38666	-2.06413
H	2.26292	2.30015	-3.18939
H	0.48412	1.23036	-1.84402
H	-0.75020	-2.31886	2.32363
H	0.87166	-3.05423	2.36669
H	0.61662	-1.38836	2.94172
C	4.38859	-1.24759	1.09533
C	3.03219	-1.32863	1.42176
H	1.75297	-1.79117	-1.68475
H	4.15065	-1.64949	-2.27024
H	5.85450	-1.30299	-0.48701
H	5.12143	-1.09990	1.88416
H	2.74003	-1.24379	2.46306

Conformer 4
Energy: -1268.75878 Hartree (Rel: 0.8 kcal/mol)
XYZ coordinates for conf 4:

C	-4.00159	-3.09951	-0.69471
C	-3.11186	-4.14839	-0.94281
C	-1.74117	-3.96039	-0.76451
C	-1.26067	-2.71685	-0.35111
C	-2.13653	-1.62585	-0.14680
C	-3.51382	-1.85332	-0.30198
O	0.09131	-2.58754	-0.19934
C	0.59117	-1.59419	0.75455
C	-0.19206	-0.30869	0.55236
C	-1.50417	-0.35102	0.19337
C	0.24533	1.10773	0.90075
C	-1.05429	1.87632	0.71202
C	-2.06787	1.01504	0.24762
C	-1.29986	3.23414	0.87624
C	-2.56672	3.74629	0.58660
C	-3.57076	2.89461	0.10790
C	-3.33045	1.52521	-0.07050
O	-4.78307	3.46210	-0.17958
C	1.37437	1.70174	0.05552
C	2.53771	2.18889	0.65940
C	3.55230	2.77086	-0.10728
C	3.40980	2.87926	-1.49048
C	2.24615	2.40140	-2.10275
C	1.23887	1.81875	-1.33606
C	0.33118	-2.13734	2.17409
C	2.08358	-1.47975	0.42998
C	2.49962	-1.45983	-0.90921
C	3.85086	-1.35595	-1.23529
C	4.81434	-1.27054	-0.22640
H	-5.07132	-3.24953	-0.80697
H	-3.48387	-5.12005	-1.25573
H	-1.03191	-4.76617	-0.92633
H	-4.21566	-1.05492	-0.09353
H	0.56674	1.14302	1.95255
H	-0.51333	3.90139	1.21922
H	-2.78982	4.80106	0.71241
H	-4.11961	0.89818	-0.47247
H	-5.38800	2.77567	-0.50400
H	2.65647	2.10794	1.73728
H	4.45066	3.14026	0.38043
H	4.19552	3.33349	-2.08822

H	2.12398	2.48390	-3.17970
H	0.33607	1.45234	-1.81790
H	-0.73662	-2.33811	2.29716
H	0.88796	-3.06550	2.33792
H	0.62168	-1.40967	2.93765
C	4.41166	-1.29022	1.10843
C	3.05608	-1.39504	1.43347
H	1.75731	-1.53345	-1.69641
H	4.15219	-1.34298	-2.27935
H	5.86800	-1.19149	-0.48030
H	5.14989	-1.22849	1.90362
H	2.77046	-1.41344	2.47969

Revised structure of 87{3a}, i.e. 90{3a-rev} ($CDCl_3$)

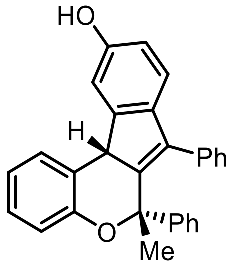

		Conf1	Conf2
Rel energy (kcal/mol):		0.00	0.11

C-nom	iGau	Exp	Calc	diff	1	2
C	4	154.10	154.12	0.02	[154.10	154.14]
C	16	153.30	153.73	0.43	[153.75	153.70]
C	9	146.70	145.41	-1.29	[145.59	145.20]
C	13	144.80	143.83	-0.97	[144.07	143.55]
C	26	141.20	141.62	0.42	[141.66	141.58]
C	12	140.40	138.34	-2.06	[138.38	138.30]
C	11	138.40	139.71	1.31	[139.62	139.83]
C	19	135.60	135.09	-0.51	[135.12	135.06]
C	20	129.30	128.20	-1.10	[128.20	128.20]
C	24	129.30	128.20	-1.10	[128.20	128.20]
C	2	128.30	127.10	-1.20	[127.08	127.12]
C	21	128.30	126.88	-1.42	[126.87	126.89]
C	23	128.00	126.88	-1.12	[126.87	126.89]
C	29	128.00	126.48	-1.52	[126.47	126.50]
C	28	127.60	126.45	-1.15	[126.45	126.46]
C	46	127.30	126.45	-0.85	[126.45	126.46]
C	5	126.50	126.02	-0.48	[126.03	126.00]
C	22	126.40	126.25	-0.15	[126.23	126.27]
C	27	125.50	125.85	0.35	[125.85	125.86]
C	47	125.50	125.85	0.35	[125.85	125.86]
C	6	123.30	124.48	1.18	[124.59	124.35]
C	14	121.80	122.30	0.50	[121.95	122.72]
C	1	120.90	120.81	-0.09	[120.85	120.76]
C	3	117.40	118.61	1.21	[118.56	118.67]
C	15	114.00	113.27	-0.73	[112.66	114.00]
C	17	112.80	112.17	-0.63	[112.65	111.59]
C	8	82.00	84.58	2.58	[84.55	84.61]
C	10	47.50	49.92	2.42	[49.94	49.90]
C	25	29.40	29.59	0.19	[29.58	29.61]

13C chem shifts: RMSD=1.14ppm (MAE=0.94) N=29 {-2.06 2.58}

Fractions: 0.548 0.452

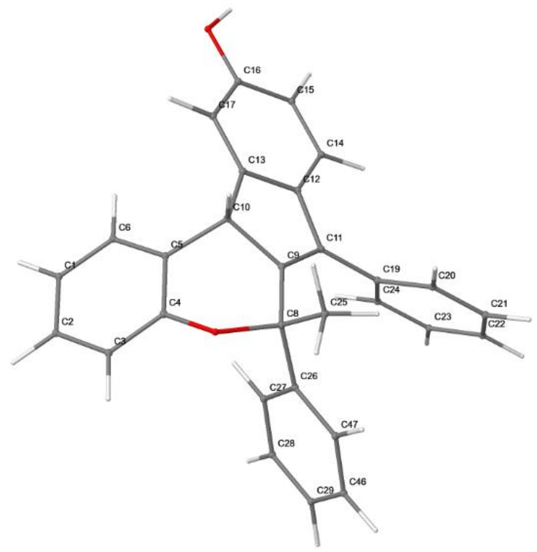

Conformer 1
Energy: -1268.75227 Hartree (Rel: 0.0 kcal/mol)
XYZ coordinates for conf 1:

C	2.48485	3.67475	-1.28664
C	1.39458	4.50250	-1.00856
C	0.38397	4.06854	-0.14807
C	0.48723	2.81008	0.44799
C	1.59209	1.97805	0.20007
C	2.57797	2.41471	-0.68590
O	-0.47892	2.41618	1.33621
C	-0.98156	1.02184	1.27877
C	0.14894	0.10436	0.86150
C	1.56905	0.65895	0.95356
C	0.15956	-1.15597	0.35527
C	1.56017	-1.54915	0.09417
C	2.41430	-0.48259	0.44323
C	2.09849	-2.74878	-0.37284
C	3.48556	-2.87295	-0.49339
C	4.32751	-1.81213	-0.13341
C	3.79283	-0.60550	0.34858
O	5.69057	-1.89344	-0.22304
C	-0.97273	-2.07522	0.07346
C	-1.77751	-2.58498	1.10424
C	-2.81650	-3.47584	0.82667
C	-3.06398	-3.87848	-0.48700
C	-2.26096	-3.38980	-1.52106
C	-1.22121	-2.50232	-1.24266
C	-1.37615	0.75728	2.73916
C	-2.16488	1.03222	0.30151
C	-1.92284	1.13818	-1.07793
C	-2.97589	1.23445	-1.98658
C	-4.29821	1.21804	-1.53516
H	3.26395	4.00654	-1.96710
H	1.32072	5.48273	-1.47178
H	-0.48329	4.68482	0.06773
H	3.42468	1.77193	-0.90522
H	1.45528	-3.58326	-0.63634
H	3.91855	-3.80209	-0.85838
H	4.47021	0.19084	0.64216
H	5.93656	-2.77154	-0.55610
H	-1.57332	-2.29862	2.13149
H	-3.42796	-3.85903	1.63931
H	-3.87267	-4.57116	-0.70370
H	-2.44451	-3.69924	-2.54657
H	-0.60298	-2.12236	-2.05134
H	-2.02365	1.55781	3.10940
H	-0.47683	0.73293	3.36070

H	-1.89527	-0.19839	2.83850
C	-4.55243	1.10823	-0.16817
C	-3.49470	1.02107	0.74126
H	-0.90131	1.13915	-1.44430
H	-2.76267	1.31680	-3.04912
H	-5.12026	1.28655	-2.24257
H	-5.57567	1.09047	0.19772
H	-3.72273	0.94061	1.79839
H	1.81476	0.86539	2.00927

Conformer 2
Energy: -1268.75209 Hartree (Rel: 0.1 kcal/mol)
XYZ coordinates for conf 2:

C	2.50467	3.65349	-1.29106
C	1.42228	4.48979	-1.00789
C	0.41155	4.06317	-0.14391
C	0.50691	2.80348	0.45089
C	1.60460	1.96312	0.19880
C	2.58999	2.39243	-0.69129
O	-0.45958	2.41562	1.34097
C	-0.97380	1.02510	1.28081
C	0.14952	0.09973	0.86175
C	1.57380	0.64389	0.95185
C	0.15119	-1.16006	0.35410
C	1.54914	-1.56225	0.09107
C	2.40935	-0.50356	0.43948
C	2.07957	-2.76735	-0.37775
C	3.46260	-2.90318	-0.50005
C	4.31363	-1.84856	-0.14052
C	3.78924	-0.63867	0.34287
O	5.65921	-2.05789	-0.27544
C	-0.98743	-2.07141	0.07247
C	-1.79564	-2.57547	1.10334
C	-2.84047	-3.45952	0.82592
C	-3.09053	-3.86082	-0.48766
C	-2.28432	-3.37765	-1.52182
C	-1.23873	-2.49704	-1.24360
C	-1.37082	0.76170	2.74068
C	-2.15682	1.04659	0.30336
C	-1.91380	1.14983	-1.07611
C	-2.96593	1.25556	-1.98480
C	-4.28832	1.25164	-1.53338
H	3.28305	3.97919	-1.97528
H	1.35426	5.47076	-1.47041
H	-0.45024	4.68592	0.07508
H	3.42982	1.74239	-0.91578
H	1.42847	-3.59584	-0.64092
H	3.90385	-3.82613	-0.86363
H	4.46061	0.16351	0.64309
H	6.13643	-1.25967	0.00310
H	-1.58935	-2.29062	2.13058
H	-3.45431	-3.83867	1.63865
H	-3.90357	-4.54842	-0.70420
H	-2.46963	-3.68634	-2.54722
H	-0.61775	-2.12176	-2.05235
H	-2.01156	1.56708	3.11216
H	-0.47184	0.72859	3.36232
H	-1.89809	-0.18964	2.83833
C	-4.54357	1.14469	-0.16635
C	-3.48671	1.04798	0.74311
H	-0.89237	1.14077	-1.44260
H	-2.75195	1.33531	-3.04738
H	-5.10969	1.32739	-2.24085
H	-5.56694	1.13642	0.19953
H	-3.71555	0.96982	1.80024
H	1.82229	0.84805	2.00739

Originally assigned (incorrect) structure of 88{3l} ($CDCl_3$)

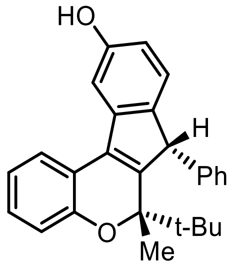

	Conf1	Conf2
Rel energy (kcal/mol):	0.00	0.10

C-nom	iGau	Exp	Calc	diff	1	2
C	16	154.70	154.24	-0.46	[154.26	154.22]
C	4	153.50	153.55	0.05	[153.51	153.59]
C	9	145.10	147.06	1.96	[146.97	147.16]
C	12	142.90	143.00	0.10	[142.98	143.03]
C	19	141.90	141.54	-0.36	[141.65	141.42]
C	13	137.30	140.79	3.49	[141.04	140.49]
C	10	136.20	139.62	3.42	[139.74	139.47]
C	2	130.60	129.10	-1.50	[129.11	129.08]
C	20	130.30	128.32	-1.98	[128.31	128.33]
C	24	127.68	128.32	0.64	[128.31	128.33]
C	21	127.68	127.88	0.20	[127.87	127.89]
C	23	127.65	127.88	0.23	[127.87	127.89]
C	22	127.00	125.59	-1.41	[125.56	125.62]
C	14	126.70	125.66	-1.04	[125.34	126.05]
C	6	124.00	123.66	-0.34	[123.86	123.43]
C	5	121.70	120.80	-0.90	[120.76	120.84]
C	1	121.60	119.43	-2.17	[119.49	119.36]
C	3	118.30	116.95	-1.35	[116.87	117.05]
C	15	113.90	111.78	-2.12	[111.13	112.55]
C	17	112.60	107.53	-5.07	[108.07	106.89]
C	8	88.10	87.70	-0.40	[87.66	87.75]
C	11	51.60	56.98	5.38	[56.92	57.06]
C	26	40.10	39.04	-1.06	[39.04	39.04]
C	27	26.60	27.09	0.49	[27.09	27.09]
C	28	26.60	27.09	0.49	[27.09	27.09]
C	29	26.60	27.09	0.49	[27.09	27.09]
C	25	24.30	20.74	-3.56	[20.76	20.72]

13C chem shifts: RMSD=2.10ppm (MAE=1.51) N=27 {-5.07 5.38}

Fractions: 0.542 0.458

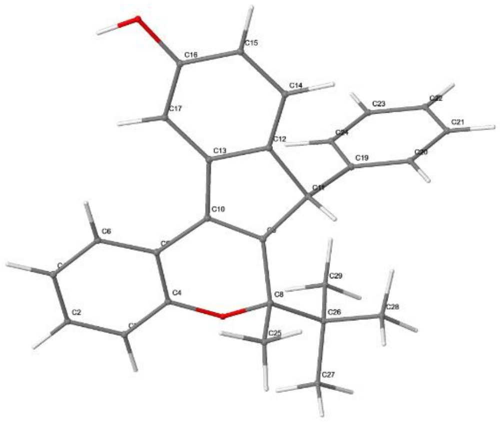

Conformer 1
Energy: -1194.95291 Hartree (Rel: 0.0 kcal/mol)
XYZ coordinates for conf 1:

C	4.53614	1.68295	0.71510

C	5.04687	0.40661	0.96840
C	4.23046	-0.71389	0.81966
C	2.89838	-0.55129	0.43355
C	2.34297	0.72942	0.23075
C	3.19913	1.83786	0.35049
O	2.13909	-1.67166	0.30218
C	1.00861	-1.67238	-0.62965
C	0.22068	-0.37281	-0.39577
C	0.91396	0.76566	-0.08548
C	-1.21114	-0.01813	-0.81990
C	-1.19250	1.50270	-0.74755
C	0.03782	1.94807	-0.22958
C	-2.21498	2.39980	-1.02248
C	-2.01231	3.76492	-0.79514
C	-0.79937	4.20814	-0.25388
C	0.23162	3.30529	0.04029
O	-0.55845	5.52842	0.01914
C	-2.38922	-0.50861	0.02933
C	-3.53897	-1.01917	-0.58530
C	-4.66052	-1.37498	0.17137
C	-4.64810	-1.21792	1.55705
C	-3.50869	-0.69717	2.18010
C	-2.39378	-0.34094	1.42269
C	1.56859	-1.60012	-2.06999
C	0.33133	-3.06632	-0.32501
C	1.36540	-4.20917	-0.49106
C	-0.83313	-3.36964	-1.28917
C	-0.16865	-3.11156	1.13338
H	5.17522	2.55673	0.80186
H	6.08561	0.27980	1.26089
H	4.60870	-1.71849	0.98234
H	2.82776	2.83160	0.13751
H	-1.37822	-0.35901	-1.85125
H	-3.17187	2.05275	-1.40343
H	-2.79926	4.48084	-1.02131
H	1.13415	3.69794	0.49013
H	-1.34449	6.04990	-0.21042
H	-3.56082	-1.14013	-1.66619
H	-5.54059	-1.77350	-0.32649
H	-5.51673	-1.49478	2.14814
H	-3.49033	-0.56473	3.25867
H	-1.51603	0.07211	1.91284
H	2.17424	-0.69727	-2.18733
H	2.19412	-2.46476	-2.30513
H	0.75492	-1.55007	-2.79957
H	2.25317	-4.04551	0.12365
H	0.90208	-5.15150	-0.17673
H	1.68336	-4.33952	-1.53061
H	-1.65750	-2.66221	-1.19517
H	-1.23578	-4.36308	-1.05854
H	-0.50550	-3.38819	-2.33416
H	-0.60256	-4.09634	1.34418
H	0.65589	-2.94793	1.83333
H	-0.93800	-2.36268	1.32901

Conformer 2
Energy: -1194.95307 Hartree (Rel: 0.1 kcal/mol)
XYZ coordinates for conf 2:

C	4.57548	1.58040	0.69235
C	5.05450	0.29452	0.95916
C	4.21044	-0.80694	0.82276
C	2.88220	-0.61548	0.43660
C	2.35777	0.67664	0.22368
C	3.24192	1.76436	0.32854
O	2.09529	-1.71729	0.31375
C	0.96823	-1.69684	-0.62295
C	0.20983	-0.37857	-0.39627
C	0.92937	0.74456	-0.08964
C	-1.21249	0.00998	-0.82312
C	-1.15848	1.53003	-0.75130
C	0.07959	1.94548	-0.23352

C	-2.16183	2.45248	-1.02520
C	-1.93070	3.80997	-0.79594
C	-0.70819	4.22554	-0.25205
C	0.30252	3.30021	0.03991
O	-0.55581	5.56578	-0.01693
C	-2.40414	-0.45203	0.02311
C	-3.56362	-0.93650	-0.59435
C	-4.69516	-1.26558	0.15949
C	-4.68302	-1.10746	1.54505
C	-3.53377	-0.61237	2.17085
C	-2.40884	-0.28271	1.41624
C	1.53483	-1.64422	-2.06145
C	0.25840	-3.07354	-0.31455
C	1.26568	-4.24063	-0.47668
C	-0.91246	-3.35201	-1.27839
C	-0.24351	-3.10253	1.14360
H	5.23695	2.43851	0.76716
H	6.09029	0.14504	1.25130
H	4.56441	-1.81886	0.99428
H	2.89560	2.76423	0.10139
H	-1.38460	-0.32742	-1.85481
H	-3.12544	2.12606	-1.40752
H	-2.68913	4.55534	-1.01351
H	1.22049	3.65234	0.49770
H	0.32478	5.72619	0.35894
H	-3.58533	-1.05792	-1.67518
H	-5.58284	-1.64399	-0.34049
H	-5.55956	-1.36338	2.13388
H	-3.51557	-0.47893	3.24930
H	-1.52333	0.11092	1.90843
H	2.15898	-0.75457	-2.18229
H	2.14343	-2.52278	-2.28928
H	0.72477	-1.58213	-2.79406
H	2.15884	-4.09363	0.13451
H	0.78202	-5.17032	-0.15548
H	1.57740	-4.38421	-1.51637
H	-1.72023	-2.62557	-1.18579
H	-1.33782	-4.33547	-1.04576
H	-0.58534	-3.38022	-2.32330
H	-0.70351	-4.07503	1.35597
H	0.58456	-2.95947	1.84387
H	-0.99296	-2.33313	1.33699

Revised structure of 88{3l}, i.e. 91{3l-rev} (CDCl$_3$)

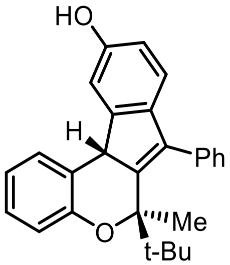

					Conf1	Conf2
Rel energy (kcal/mol):					0.00	0.10

C-nom	iGau	Exp	Calc	diff	1	2
C	4	154.70	154.52	-0.18	[154.50	154.54]
C	16	153.50	153.44	-0.06	[153.46	153.42]
C	9	145.10	145.17	0.07	[145.31	145.00]
C	13	142.90	143.14	0.24	[143.39	142.85]
C	11	141.90	140.02	-1.88	[139.94	140.11]
C	12	137.30	139.68	2.38	[139.74	139.62]
C	19	136.20	136.22	0.02	[136.26	136.18]
C	20	130.60	130.44	-0.16	[130.45	130.43]
C	24	130.30	130.44	0.14	[130.45	130.43]
C	2	127.68	126.97	-0.71	[126.95	126.99]
C	21	127.68	126.91	-0.77	[126.90	126.93]
C	23	127.65	126.91	-0.74	[126.90	126.93]
C	5	127.00	126.65	-0.35	[126.66	126.63]
C	22	126.70	126.48	-0.22	[126.46	126.50]
C	6	124.00	124.06	0.06	[124.18	123.92]
C	14	121.70	122.38	0.68	[122.03	122.80]
C	1	121.60	120.75	-0.85	[120.78	120.72]
C	3	118.30	118.14	-0.16	[118.09	118.19]
C	15	113.90	113.10	-0.80	[112.52	113.79]
C	17	112.60	111.45	-1.15	[111.91	110.90]
C	8	88.10	89.91	1.81	[89.87	89.95]
C	10	51.60	52.95	1.35	[52.98	52.92]
C	26	40.10	41.03	0.93	[41.02	41.04]
C	27	26.60	26.83	0.23	[26.84	26.82]
C	28	26.60	26.83	0.23	[26.84	26.82]
C	29	26.60	26.83	0.23	[26.84	26.82]
C	25	24.30	24.85	0.55	[24.86	24.84]

13C chem shifts: RMSD=0.88ppm (MAE=0.63) N=27 {-1.88 2.38}

Fractions: 0.541 0.459

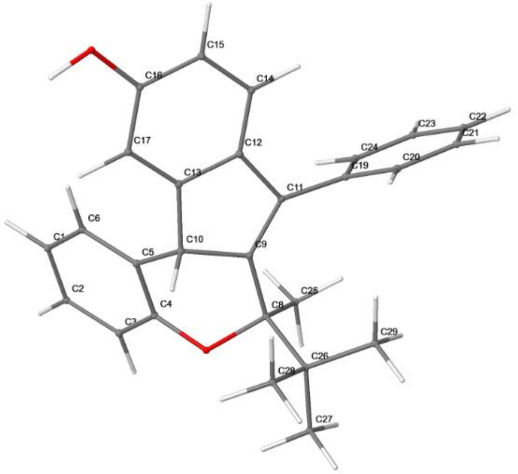

Conformer 1

Energy: -1194.95408 Hartree (Rel: 0.0 kcal/mol)
XYZ coordinates for conf 1:

C	-4.28844	-0.20053	-1.76676
C	-4.24593	-1.58873	-1.91587
C	-3.25860	-2.33586	-1.26884
C	-2.33025	-1.68080	-0.45785
C	-2.37778	-0.28978	-0.27705
C	-3.35248	0.44579	-0.95165
O	-1.39675	-2.41508	0.22412
C	0.00435	-1.95160	0.22507
C	0.00609	-0.42802	0.28133
C	-1.31546	0.24828	0.66738
C	0.93132	0.52023	-0.03600
C	0.34407	1.85922	0.18802
C	-0.99345	1.71781	0.60426
C	0.90106	3.13179	0.05283
C	0.11936	4.25229	0.34789
C	-1.20524	4.10129	0.78008
C	-1.77250	2.82315	0.91480
O	-2.00185	5.17011	1.09082
C	2.32611	0.42235	-0.55403
C	3.41232	0.74168	0.27889
C	4.71961	0.72832	-0.21070
C	4.96442	0.41244	-1.54924
C	3.89240	0.11818	-2.39438
C	2.58502	0.13000	-1.90290
C	0.61257	-2.47987	-1.08386
C	0.61493	-2.63967	1.51724
C	0.36671	-4.16524	1.48593
C	-0.04534	-2.07284	2.79168
C	2.13284	-2.39832	1.61067
H	-5.04875	0.38111	-2.28042
H	-4.97390	-2.09286	-2.54586
H	-3.19577	-3.41392	-1.38201
H	-3.38602	1.52481	-0.83780
H	1.92979	3.25969	-0.27077
H	0.54035	5.25059	0.24741
H	-2.79724	2.73407	1.26310
H	-1.49787	5.99028	0.96447
H	3.22851	0.99788	1.31899
H	5.54561	0.96745	0.45380
H	5.98139	0.40279	-1.93182
H	4.07074	-0.11564	-3.44061
H	1.75541	-0.08238	-2.57040
H	0.09150	-2.02944	-1.93447
H	0.49396	-3.56410	-1.14952
H	1.67183	-2.23404	-1.17004
H	0.89661	-4.65401	0.66177
H	0.73777	-4.60882	2.41741
H	-0.69708	-4.39839	1.39743
H	0.17326	-1.00720	2.92135
H	0.34416	-2.59712	3.67209
H	-1.13112	-2.20729	2.77439
H	2.51777	-2.87482	2.52007
H	2.67576	-2.82726	0.76211
H	2.37449	-1.33425	1.66644
H	-1.59843	-0.03691	1.69168

Conformer 2
Energy: -1194.95393 Hartree (Rel: 0.1 kcal/mol)
XYZ coordinates for conf 2:

C	-4.27390	-0.27591	-1.78424
C	-4.21103	-1.66438	-1.92335
C	-3.21543	-2.39286	-1.26772
C	-2.29962	-1.71907	-0.45784
C	-2.36873	-0.32781	-0.28573
C	-3.35064	0.38918	-0.96968
O	-1.35664	-2.43407	0.23109
C	0.03754	-1.94859	0.23054
C	0.01477	-0.42495	0.27852
C	-1.31825	0.23067	0.65983

C	0.92410	0.53808	-0.04045
C	0.31397	1.86782	0.18088
C	-1.01943	1.70437	0.59422
C	0.85056	3.15191	0.04752
C	0.05443	4.25854	0.34455
C	-1.26842	4.08601	0.77642
C	-1.81639	2.79974	0.90495
O	-1.98542	5.21573	1.06500
C	2.32075	0.46105	-0.55677
C	3.40072	0.80382	0.27495
C	4.70854	0.80806	-0.21334
C	4.95981	0.48655	-1.54935
C	3.89359	0.16911	-2.39343
C	2.58569	0.16333	-1.90329
C	0.65539	-2.47493	-1.07449
C	0.65753	-2.61922	1.52744
C	0.42898	-4.14806	1.50817
C	-0.00886	-2.05148	2.79830
C	2.17231	-2.35758	1.61749
H	-5.03979	0.29120	-2.30580
H	-4.92907	-2.18313	-2.55283
H	-3.13627	-3.47054	-1.37396
H	-3.39909	1.46860	-0.86464
H	1.87758	3.29504	-0.27532
H	0.44431	5.26784	0.25464
H	-2.84115	2.67618	1.24956
H	-2.87321	4.96178	1.36511
H	3.21175	1.06433	1.31306
H	5.52986	1.06541	0.45013
H	5.97719	0.49066	-1.93091
H	4.07679	-0.06909	-3.43783
H	1.76029	-0.06694	-2.57009
H	0.12706	-2.03927	-1.92830
H	0.55597	-3.56151	-1.13311
H	1.71019	-2.21098	-1.16170
H	0.96214	-4.63611	0.68571
H	0.80918	-4.57984	2.44152
H	-0.63200	-4.39567	1.42542
H	0.19626	-0.98223	2.92002
H	0.38793	-2.56435	3.68215
H	-1.09287	-2.20007	2.78303
H	2.56377	-2.81980	2.53145
H	2.72003	-2.78842	0.77300
H	2.40065	-1.29004	1.66205
H	-1.59875	-0.05681	1.68418

Originally assigned correct structure 92{1} ($CDCl_3$)

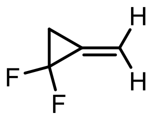

					Conf1
	Rel energy (kcal/mol):				0.00

C-nom	iGau	Exp	Calc	diff	1
C-C	3	127.90	128.23	0.33	[128.23]
C-C	2	106.30	106.12	-0.18	[106.12]
C-CH2	4	111.40	112.53	1.13	[112.53]
C-CH2	1	18.60	16.76	-1.84	[16.76]

13C chem shifts: RMSD=1.10ppm (MAE=0.87) N=4 {-1.84 1.13}

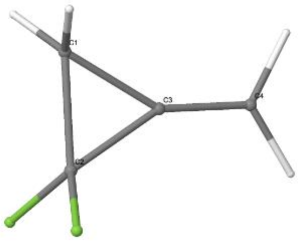

Conformer 1
Energy: -354.42080 Hartree (Rel: 0.0 kcal/mol)
XYZ coordinates for conf 1:

C	0.16614	1.34241	-0.00007
C	-0.51740	0.00205	-0.00000
C	0.93516	0.05479	0.00000
C	2.10339	-0.56623	0.00003
F	-1.19956	-0.39932	1.10462
F	-1.19955	-0.39945	-1.10458
H	0.13101	1.92869	-0.91839
H	0.13099	1.92879	0.91817
H	3.04083	-0.01496	0.00000
H	2.16538	-1.65165	0.00009

Originally assigned correct structure 95{2} ($CDCl_3$)

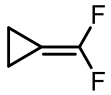

	Conf1
Rel energy (kcal/mol):	0.00

C-nom	iGau	Exp	Calc	diff	1
C-C	5	150.40	150.35	-0.05	[150.35]
C-C	3	67.80	67.45	-0.35	[67.45]
C-CH2	1	3.10	2.92	-0.18	[2.92]
C-CH2	2	3.10	2.92	-0.18	[2.92]

13C chem shifts: RMSD=0.22ppm (MAE=0.19) N=4 {-0.35 -0.05}

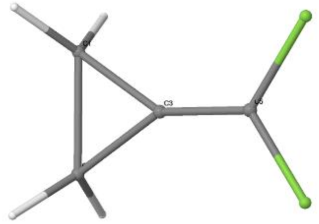

Conformer 1
Energy: -354.42595 Hartree (Rel: 0.0 kcal/mol)
XYZ coordinates for conf 1:

C	0.00000	0.76640	-1.77430
C	-0.00000	-0.76640	-1.77430
C	-0.00000	0.00000	-0.52210
F	0.00000	1.09040	1.55490
C	0.00000	0.00000	0.78630
F	-0.00000	-1.09040	1.55490
H	0.91480	1.27720	-2.07050
H	-0.91480	1.27720	-2.07050
H	-0.91480	-1.27720	-2.07050
H	0.91480	-1.27720	-2.07050

Originally assigned correct structure 93{4} ($CDCl_3$)

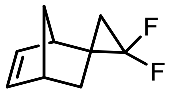

C-nom	iGau	Exp	Calc	diff	Conf1
				Rel energy (kcal/mol):	0.00
					1
C	7	136.90	137.35	0.45	[137.35]
C	2	134.60	135.51	0.91	[135.51]
C	9	115.80	115.02	-0.78	[115.02]
C	5	49.30	49.51	0.21	[49.51]
C	1	48.10	48.90	0.80	[48.90]
C	6	42.20	43.40	1.20	[43.40]
C	3	34.50	35.06	0.56	[35.06]
C	4	34.00	34.00	0.00	[34.00]
C	8	20.60	19.99	-0.61	[19.99]

13C chem shifts: RMSD=0.70ppm (MAE=0.61) N=9 {-0.78 1.20}

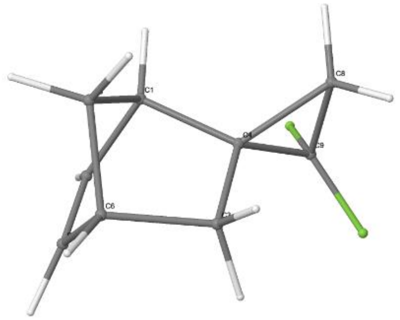

Conformer 1
Energy: -548.57275 Hartree (Rel: 0.0 kcal/mol)
XYZ coordinates for conf 1:

C	-0.74075	0.57850	0.93554
C	-1.22495	1.49300	-0.18596
C	-0.56400	-1.26249	-0.67841
C	0.27823	-0.38625	0.27044
C	-1.95349	-0.37570	1.05979
C	-2.00126	-0.67067	-0.46019
C	-1.97406	0.74940	-1.01294
C	1.48503	-0.94949	1.06088
C	1.65727	0.03667	-0.03324
H	-0.23458	-1.19212	-1.71909
H	-0.52876	-2.31648	-0.38004
H	-0.37784	1.06440	1.84295
H	-2.39370	1.04791	-1.96855
H	-2.85218	0.12963	1.42612
H	-1.74908	-1.26253	1.67150
H	-0.90145	2.51916	-0.32590
H	1.62823	-0.60421	2.08202
H	1.78372	-1.97703	0.86768
F	2.24486	-0.34775	-1.19875
F	2.05046	1.30667	0.26421
H	-2.80440	-1.31682	-0.82136

Originally assigned correct structure 94{5} ($CDCl_3$)

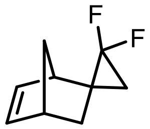

				**Conf1**	
	Rel energy (kcal/mol):			0.00	

C-nom	iGau	Exp	Calc	diff	1
C	7	136.90	138.87	1.97	[138.87]
C	2	134.60	134.86	0.26	[134.86]
C	9	115.80	115.03	-0.77	[115.03]
C	5	49.30	50.13	0.83	[50.13]
C	1	48.10	46.31	-1.79	[46.31]
C	6	42.70	43.17	0.47	[43.17]
C	3	34.50	34.49	-0.01	[34.49]
C	4	34.00	33.53	-0.47	[33.53]
C	8	18.60	18.10	-0.50	[18.10]

13C chem shifts: RMSD=1.01ppm (MAE=0.79) N=9 {-1.79 1.97}

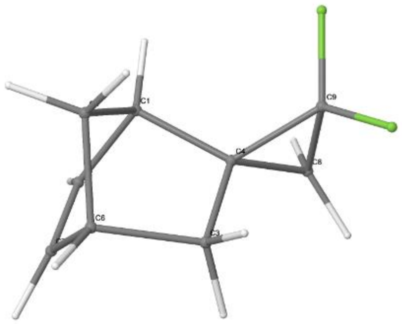

Conformer 1
Energy: -548.57282 Hartree (Rel: 0.0 kcal/mol)
XYZ coordinates for conf 1:

C	-0.70422	-0.96448	-0.42745
C	-1.87474	-1.16390	0.53064
C	-0.48902	1.34679	0.36757
C	0.26887	0.00564	0.29279
C	-1.33934	0.02108	-1.43990
C	-1.84373	0.98299	-0.33537
C	-2.55181	-0.00769	0.58167
C	1.24477	-0.47345	1.38865
C	1.72792	-0.05078	0.05276
H	-0.63412	1.69226	1.39581
H	0.04154	2.13127	-0.18322
H	-0.23044	-1.86393	-0.82347
H	-3.38350	0.24020	1.23385
H	-2.15225	-0.43071	-2.01618
H	-0.60942	0.47677	-2.11961
H	-2.03722	-2.05220	1.13247
H	1.26678	-1.53550	1.62137
H	1.42991	0.19222	2.22828
F	2.43780	1.10351	-0.07393
F	2.24022	-0.97892	-0.80214
H	-2.42567	1.85103	-0.65289

Originally assigned (incorrect) structure of 96{13} ($CDCl_3$)

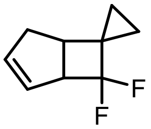

					Conf1
	Rel energy (kcal/mol):				0.00
**C-nom**	**iGau**	**Exp**	**Calc**	**diff**	**1**
C	6	140.80	135.77	-5.03	[135.77]
C	7	131.80	127.82	-3.98	[127.82]
C	3	130.80	122.94	-7.86	[122.94]
C	2	51.10	59.53	8.43	[59.53]
C	5	49.00	36.18	-12.82	[36.18]
C	1	47.90	35.39	-12.51	[35.39]
C	4	31.90	32.98	1.08	[32.98]
C	9	8.70	9.49	0.79	[9.49]
C	8	7.40	6.64	-0.76	[6.64]

13C chem shifts: RMSD=7.43ppm (MAE=5.92) N=9 {-12.82 8.43}

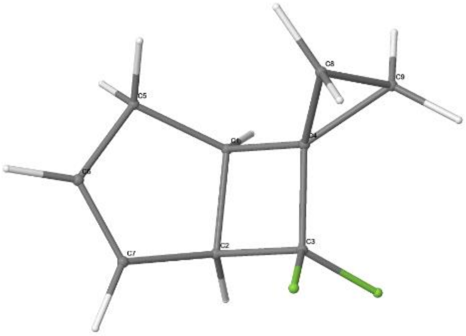

Conformer 1
Energy: -548.57983 Hartree (Rel: 0.0 kcal/mol)
XYZ coordinates for conf 1:

C	-0.61438	0.64582	-0.92592
C	-0.68155	-0.91380	-0.70025
C	0.71049	-0.85022	-0.03550
C	0.69171	0.66554	-0.09950
C	-1.88758	1.23957	-0.28410
C	-2.48160	0.07077	0.47074
C	-1.84028	-1.08065	0.24565
C	1.10146	1.57332	1.02711
C	1.90905	1.53437	-0.24496
F	1.70001	-1.42412	-0.79785
F	0.82398	-1.41156	1.20912
H	-0.46664	0.95940	-1.96236
H	-0.71013	-1.57001	-1.57663
H	-2.58713	1.62013	-1.04181
H	-1.66172	2.08661	0.37747
H	-3.34832	0.17790	1.11800
H	-2.10277	-2.03982	0.68180
H	0.48919	2.45044	1.22032
H	1.50933	1.10255	1.91772
H	2.87902	1.04364	-0.23299
H	1.83938	2.38191	-0.92256

Revised structure of 96{13}, i.e. 97{13-rev} ($CDCl_3$)

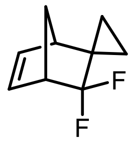

					Conf1
	Rel energy (kcal/mol):				0.00
**C-nom**	**iGau**	**Exp**	**Calc**	**diff**	**1**
C	2	140.80	141.17	0.37	[141.17]
C	7	131.80	132.68	0.88	[132.68]
C	3	130.80	129.91	-0.89	[129.91]
C	6	51.10	51.68	0.58	[51.68]
C	1	49.00	48.65	-0.35	[48.65]
C	5	47.90	48.06	0.16	[48.06]
C	4	31.90	32.25	0.35	[32.25]
C	8	8.70	8.99	0.29	[8.99]
C	9	7.40	7.68	0.28	[7.68]

13C chem shifts: RMSD=0.52ppm (MAE=0.46) N=9 {-0.89 0.88}

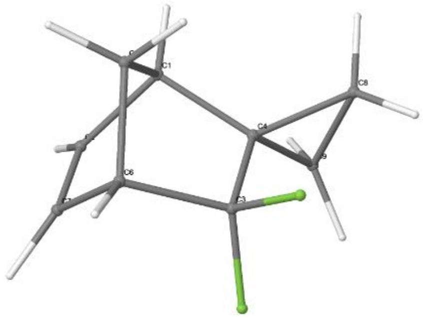

Conformer 1
Energy: -548.58680 Hartree (Rel: 0.0 kcal/mol)
XYZ coordinates for conf 1:

C	0.58769	-1.31111	0.49571
C	1.55895	-1.23666	-0.68210
C	-0.22144	0.89702	-0.06131
C	-0.69074	-0.55970	0.04594
C	1.21027	-0.24158	1.43142
C	1.27896	0.83205	0.31504
C	1.95999	0.03653	-0.79452
C	-2.06654	-0.88189	0.56507
C	-1.72183	-1.12702	-0.88535
F	-0.43106	1.44757	-1.30251
F	-0.89667	1.72037	0.81751
H	0.39895	-2.30172	0.91448
H	2.57024	0.47429	-1.57638
H	2.20066	-0.51658	1.80388
H	0.55888	0.03824	2.26613
H	1.77761	-2.05727	-1.35732
H	-2.16775	-1.74087	1.22418
H	-2.72251	-0.04999	0.80605
H	-2.14345	-0.46091	-1.63324
H	-1.59110	-2.15341	-1.22011
H	1.69613	1.81085	0.55792

Originally assigned (incorrect) structure of 99{5a} ($CDCl_3$)

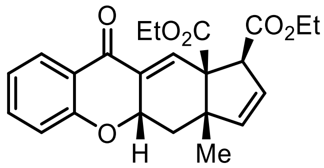

	Conf1	Conf2	Conf3	Conf4
Rel energy (kcal/mol):	0.00	0.01	0.57	0.77

C-nom	iGau	Exp	Calc	diff	1	2	3	4
C	10	192.10	181.73	-10.37	[181.80	181.75	181.63	181.57]
C	20	169.50	171.72	2.22	[171.72	171.85	171.45	171.60]
C	25	165.70	170.61	4.91	[169.83	171.47	170.03	171.16]
C	4	160.70	161.03	0.33	[161.07	161.01	161.00	160.98]
C	14	145.70	140.39	-5.31	[139.99	140.38	140.95	141.13]
C	17	142.30	138.09	-4.21	[137.69	137.90	138.80	139.21]
C	2	135.80	135.60	-0.20	[135.68	135.55	135.61	135.43]
C	9	132.60	129.04	-3.56	[129.17	128.64	129.68	129.10]
C	6	128.60	129.72	1.12	[129.70	129.71	129.77	129.79]
C	18	124.00	128.22	4.22	[128.64	128.04	128.13	127.43]
C	5	122.40	122.77	0.37	[122.67	122.82	122.81	122.89]
C	1	119.90	120.45	0.55	[120.46	120.45	120.47	120.39]
C	3	117.50	117.54	0.04	[117.57	117.52	117.55	117.49]
C	8	79.00	73.55	-5.45	[73.48	73.65	73.44	73.58]
C	24	61.30	62.50	1.20	[62.67	62.56	62.46	61.72]
C	29	60.80	62.11	1.31	[61.69	62.33	62.35	62.56]
C	13	51.50	62.05	10.55	[62.30	62.44	61.22	60.89]
C	19	44.70	60.21	15.51	[59.71	60.77	59.68	60.73]
C	12	43.70	53.17	9.47	[53.76	52.71	53.37	52.39]
C	11	36.10	42.01	5.91	[41.97	41.70	42.63	42.39]
C	30	26.00	20.80	-5.20	[20.90	20.76	20.78	20.56]
C	28	14.30	12.35	-1.95	[12.43	12.30	12.29	12.32]
C	23	13.90	12.22	-1.68	[12.23	12.19	12.13	12.41]

13C chem shifts: RMSD=5.74ppm (MAE=4.16) N=23 {-10.37 15.51}

Fractions: 0.379 0.372 0.145 0.103

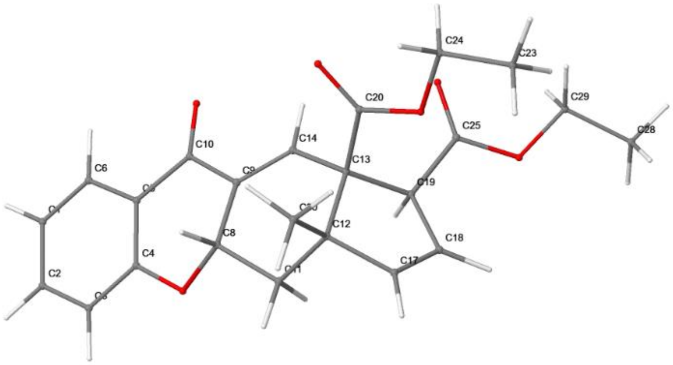

Conformer 1
Energy: -1342.22713 Hartree (Rel: 0.0 kcal/mol)
XYZ coordinates for conf 1:

C	-5.94890	1.55510	-0.38701
C	-6.30866	0.20056	-0.47957
C	-5.34260	-0.79906	-0.43615
C	-3.99206	-0.45155	-0.29770
C	-3.61361	0.90441	-0.21801
C	-4.60976	1.89611	-0.26476
O	-3.08749	-1.46764	-0.28953
C	-1.81482	-1.21102	0.34990
C	-1.23927	0.13162	-0.02417
C	-2.18365	1.27510	-0.17845
C	-0.88063	-2.33661	-0.07782

```
C          0.56925       -2.15816        0.44768
C          1.13996       -0.74046       -0.02172
C          0.08034        0.32086       -0.17504
O         -1.79997        2.43944       -0.29069
H         -2.00462       -1.24247        1.43324
C          1.47531       -3.12286       -0.29639
C          2.16391       -2.54608       -1.27879
C          1.86323       -1.07071       -1.38251
C          2.20097       -0.27176        0.99506
O          3.36795       -0.60919        0.98039
O          1.67151        0.53392        1.92752
C          1.77829        1.92296        3.87481
C          2.57395        1.01051        2.96198
C          3.06724       -0.21391       -1.75393
O          4.06558       -0.63224       -2.30194
O          2.84530        1.08721       -1.48596
C          3.45562        3.40250       -1.44423
C          3.91475        2.00822       -1.82458
C          0.60998       -2.38981        1.97150
H         -6.71240        2.32628       -0.41746
H         -7.35464       -0.07581       -0.58073
H         -5.60862       -1.84918       -0.50213
H         -4.29321        2.93306       -0.21081
H         -1.27645       -3.29964        0.26403
H         -0.87664       -2.35840       -1.17338
H          0.42425        1.32072       -0.42451
H          1.49157       -4.18163       -0.05242
H          2.84116       -3.03748       -1.96787
H          1.14257       -0.89864       -2.19904
H          2.43047        2.30649        4.66659
H          1.37137        2.77427        3.32010
H          0.94857        1.38399        4.34325
H          2.97887        0.14441        3.49391
H          3.40650        1.53099        2.47998
H          4.24113        4.12604       -1.68704
H          2.54960        3.68106       -1.99194
H          3.24727        3.46812       -0.37138
H          4.81387        1.70695       -1.27900
H          4.12205        1.92025       -2.89510
H          0.04699       -1.62980        2.52219
H          0.17578       -3.36868        2.20440
H          1.63844       -2.38878        2.34618

Conformer 2
Energy: -1342.22745 Hartree (Rel:  0.0 kcal/mol)
XYZ coordinates for conf 2:
C         -6.19001       -0.40061       -1.45111
C         -6.38858       -1.10499       -0.25238
C         -5.33272       -1.33541        0.62323
C         -4.05364       -0.85857        0.30641
C         -3.83513       -0.16270       -0.90017
C         -4.91925        0.05694       -1.76847
O         -3.05206       -1.13587        1.18460
C         -1.90292       -0.25581        1.16888
C         -1.43116        0.05382       -0.23025
C         -2.46743        0.24672       -1.28459
C         -0.81097       -0.95740        1.96834
C          0.53677       -0.18685        1.96178
C          1.00510        0.05275        0.45558
C         -0.12997        0.19807       -0.52469
O         -2.20136        0.68974       -2.40163
H         -2.22239        0.67307        1.66468
C          1.62648       -1.10729        2.48375
C          2.36058       -1.65431        1.51638
C          1.90104       -1.20240        0.14943
C          1.90108        1.30880        0.41279
O          3.08192        1.32369        0.69781
O          1.20514        2.40089        0.06551
C          0.97979        4.73139       -0.43388
C          1.94301        3.65160        0.01900
C          2.95226       -0.95652       -0.92427
```

O	2.76519	-0.25242	-1.89923
O	4.06654	-1.66977	-0.71587
C	6.26329	-2.42970	-1.28828
C	5.10522	-1.55339	-1.72441
C	0.41341	1.09874	2.80374
H	-7.02282	-0.22273	-2.12445
H	-7.37928	-1.47183	0.00197
H	-5.47459	-1.87418	1.55463
H	-4.72449	0.58697	-2.69555
H	-1.14296	-1.10537	3.00223
H	-0.67174	-1.95104	1.52770
H	0.14157	0.43925	-1.54873
H	1.73000	-1.31284	3.54579
H	3.15778	-2.37703	1.64648
H	1.25770	-1.98415	-0.28800
H	1.50454	5.69135	-0.48447
H	0.57715	4.50526	-1.42614
H	0.14445	4.83209	0.26655
H	2.34902	3.85114	1.01536
H	2.78252	3.52952	-0.67108
H	7.06949	-2.36545	-2.02676
H	6.65679	-2.10527	-0.31966
H	5.95444	-3.47689	-1.20646
H	4.69069	-1.86608	-2.68748
H	5.39134	-0.50072	-1.80413
H	-0.28565	1.81669	2.36335
H	0.05328	0.84669	3.80759
H	1.38275	1.59423	2.91611

Conformer 3
Energy: -1342.22834 Hartree (Rel: 0.6 kcal/mol)
XYZ coordinates for conf 3:

C	-6.29232	1.32472	0.32929
C	-6.57199	0.04906	-0.18755
C	-5.54632	-0.84592	-0.47324
C	-4.21595	-0.47139	-0.24187
C	-3.91815	0.81111	0.26269
C	-4.97322	1.69771	0.54269
O	-3.25096	-1.37460	-0.56517
C	-1.98180	-1.25168	0.11931
C	-1.49660	0.17408	0.18915
C	-2.51249	1.24485	0.40578
C	-0.99046	-2.11979	-0.64530
C	0.45825	-2.02646	-0.09239
C	0.93304	-0.50171	-0.07657
C	-0.19449	0.48409	0.11177
O	-2.20343	2.40895	0.65816
H	-2.14433	-1.63588	1.13735
C	1.39511	-2.64255	-1.11607
C	2.02653	-1.73888	-1.86231
C	1.65371	-0.32909	-1.47100
C	1.90724	-0.27301	1.09613
O	1.58104	0.23224	2.15164
O	3.14592	-0.71964	0.83655
C	5.45441	-1.07180	1.35114
C	4.14104	-0.52589	1.87698
C	2.80945	0.66166	-1.53480
O	3.81722	0.50479	-2.19283
O	2.53988	1.77298	-0.82517
C	3.04966	3.94305	0.04724
C	3.54952	2.81661	-0.83618
C	0.54531	-2.72634	1.27852
H	-7.10165	2.01281	0.55312
H	-7.60185	-0.24994	-0.36273
H	-5.75016	-1.83597	-0.86835
H	-4.71824	2.68230	0.92211
H	-1.31929	-3.16510	-0.62844
H	-1.00871	-1.79148	-1.69057
H	0.08619	1.52916	0.20588
H	1.46910	-3.72022	-1.23480
H	2.70698	-1.93971	-2.68198

H	0.91383	0.06860	-2.18550
H	6.23893	-0.92664	2.10135
H	5.37607	-2.14219	1.13609
H	5.75128	-0.55487	0.43309
H	4.19802	0.54298	2.10358
H	3.80604	-1.04557	2.77934
H	3.79202	4.74794	0.07021
H	2.10882	4.35277	-0.33376
H	2.88636	3.59344	1.07168
H	4.48943	2.39174	-0.47124
H	3.70264	3.13822	-1.87060
H	-0.02594	-2.19805	2.04866
H	0.14816	-3.74462	1.19974
H	1.58208	-2.80210	1.62002

Conformer 4
Energy: -1342.22836 Hartree (Rel: 0.8 kcal/mol)
XYZ coordinates for conf 4:

C	6.50230	1.10824	-0.70504
C	6.67823	0.48641	0.54190
C	5.59848	-0.06713	1.22191
C	4.31797	-0.00479	0.65606
C	4.12272	0.62922	-0.58794
C	5.23066	1.18138	-1.25495
O	3.29366	-0.53718	1.37635
C	2.12009	-0.95315	0.63818
C	1.68982	0.06509	-0.38816
C	2.75824	0.79110	-1.13414
C	1.02135	-1.18244	1.66879
C	-0.35046	-1.53721	1.03195
C	-0.77078	-0.39748	-0.00017
C	0.39718	0.28556	-0.66863
O	2.52030	1.49574	-2.11430
H	2.39291	-1.89157	0.13275
C	-1.41778	-1.40341	2.10430
C	-2.09106	-0.25637	2.03416
C	-1.61194	0.59757	0.88114
C	-1.62147	-1.00862	-1.13227
O	-1.18044	-1.26491	-2.23446
O	-2.88344	-1.27261	-0.75617
C	-5.12281	-2.01959	-1.14890
C	-3.75907	-1.81502	-1.77964
C	-2.65043	1.39192	0.10154
O	-2.53218	1.69912	-1.06991
O	-3.67191	1.77734	0.87881
C	-5.72116	2.93890	1.30838
C	-4.68194	2.61382	0.25316
C	-0.29949	-2.95218	0.42306
H	7.35335	1.53242	-1.22887
H	7.66965	0.42894	0.98295
H	5.72255	-0.55442	2.18372
H	5.05341	1.67203	-2.20684
H	1.31914	-1.97880	2.36036
H	0.92904	-0.26192	2.25614
H	0.15417	1.00567	-1.44507
H	-1.54561	-2.16253	2.87142
H	-2.85537	0.08400	2.72332
H	-0.92948	1.37183	1.27117
H	-5.81395	-2.42705	-1.89436
H	-5.06690	-2.72123	-0.31043
H	-5.53088	-1.07196	-0.78255
H	-3.79147	-1.10704	-2.61283
H	-3.32875	-2.75157	-2.14595
H	-6.50237	3.56930	0.87061
H	-6.18957	2.02687	1.69217
H	-5.27240	3.48007	2.14753
H	-4.19488	3.51199	-0.13759
H	-5.10938	2.06590	-0.59177
H	0.37233	-3.00526	-0.43979
H	0.05596	-3.66610	1.17460
H	-1.29134	-3.28057	0.09857

Revised structure of 99{5a}, i.e. 101{5a-rev} ($CDCl_3$)

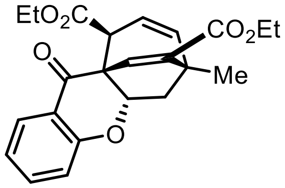

C-nom	iGau	Exp	Calc	diff	1	2
				Rel energy (kcal/mol):	Conf1 0.00	Conf2 0.28
C	10	192.10	194.52	2.42	[194.52	194.51]
C	25	169.50	168.44	-1.06	[168.61	168.16]
C	20	165.70	165.07	-0.63	[165.02	165.15]
C	4	160.70	161.48	0.78	[161.55	161.38]
C	13	145.70	145.81	0.11	[146.08	145.37]
C	17	142.30	143.04	0.74	[143.32	142.59]
C	2	135.80	136.42	0.62	[136.38	136.49]
C	14	132.60	136.62	4.02	[136.33	137.09]
C	6	128.60	130.47	1.87	[130.32	130.72]
C	18	124.00	124.32	0.32	[124.51	124.01]
C	1	122.40	121.13	-1.27	[121.11	121.15]
C	5	119.90	120.49	0.59	[120.64	120.25]
C	3	117.50	117.30	-0.20	[117.38	117.16]
C	8	79.00	79.26	0.26	[79.33	79.14]
C	29	61.30	61.93	0.63	[61.63	62.42]
C	24	60.80	61.58	0.78	[61.58	61.58]
C	9	51.50	52.48	0.98	[52.60	52.29]
C	19	44.70	45.54	0.84	[44.70	46.89]
C	11	43.70	45.16	1.46	[45.12	45.23]
C	12	36.10	36.50	0.40	[36.52	36.47]
C	30	26.00	24.85	-1.15	[24.84	24.87]
C	23	14.30	12.39	-1.91	[12.38	12.40]
C	28	13.90	12.12	-1.78	[12.01	12.29]

13C chem shifts: RMSD=1.38ppm (MAE=1.08) N=23 {-1.91 4.02}

Fractions: 0.616 0.384

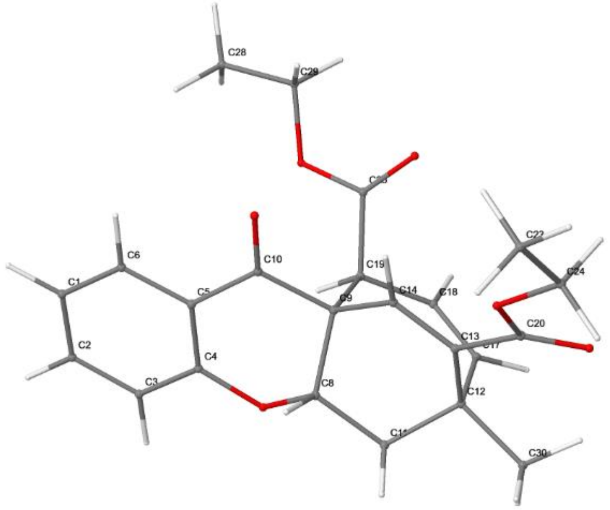

Conformer 1
Energy: -1342.21757 Hartree (Rel: 0.0 kcal/mol)
XYZ coordinates for conf 1:

C	-4.75681	-0.75078	1.89519
C	-4.85496	-1.97670	1.21584
C	-3.76731	-2.50028	0.52543
C	-2.55984	-1.79172	0.50274

C	-2.45020	-0.54688	1.15901
C	-3.56268	-0.04667	1.86178
O	-1.51423	-2.35004	-0.17086
C	-0.22320	-1.83686	0.20972
C	-0.16675	-0.29898	0.06240
C	-1.21402	0.25730	1.04925
C	0.87128	-2.58209	-0.56098
C	1.98865	-1.65457	-1.13578
C	2.27562	-0.53336	-0.11750
C	1.23183	0.12121	0.41931
O	-1.03800	1.30504	1.65705
H	-0.11210	-2.03979	1.28393
C	1.46917	-0.99249	-2.41136
C	0.38655	-0.21790	-2.50222
C	-0.56909	0.17317	-1.39447
C	3.65805	-0.03248	0.13709
O	4.58913	-0.12210	-0.64499
O	3.76489	0.57251	1.33848
C	4.92973	1.83927	2.99867
C	5.03972	1.19596	1.62938
C	-0.75368	1.69543	-1.41021
O	0.14627	2.49900	-1.54336
O	-2.04583	2.02775	-1.24936
C	-3.83643	3.58829	-0.95714
C	-2.33766	3.44538	-1.13827
C	3.19735	-2.54710	-1.46986
H	-5.61170	-0.35706	2.43601
H	-5.78852	-2.53211	1.23523
H	-3.82507	-3.45502	0.01285
H	-3.45734	0.91112	2.36157
H	1.32874	-3.32543	0.10050
H	0.41041	-3.12726	-1.39013
H	1.36031	0.95875	1.09463
H	2.04514	-1.17979	-3.31603
H	0.13008	0.20342	-3.47298
H	-1.54865	-0.26955	-1.60818
H	5.87901	2.32171	3.25501
H	4.14209	2.59947	3.01214
H	4.70493	1.09186	3.76647
H	5.81949	0.42862	1.59784
H	5.25853	1.92938	0.84718
H	-4.09490	4.64797	-0.85867
H	-4.37672	3.18039	-1.81759
H	-4.17187	3.06557	-0.05577
H	-1.77916	3.84301	-0.28584
H	-1.98096	3.94609	-2.04310
H	3.63292	-2.97638	-0.56098
H	2.86804	-3.37486	-2.10905
H	3.97770	-1.99026	-1.98892

Conformer 2
Energy: -1342.21712 Hartree (Rel: 0.3 kcal/mol)
XYZ coordinates for conf 2:

C	5.22072	0.40129	-1.66128
C	5.58682	-0.71208	-0.88664
C	4.63014	-1.44856	-0.19635
C	3.28432	-1.07044	-0.27100
C	2.90079	0.05888	-1.02628
C	3.88818	0.77926	-1.72473
O	2.37831	-1.82915	0.40952
C	1.02205	-1.69162	-0.05457
C	0.57081	-0.21267	-0.03966
C	1.49819	0.52561	-1.02585
C	0.11189	-2.63150	0.74337
C	-1.23531	-1.98180	1.19418
C	-1.73786	-1.05597	0.06884
C	-0.86486	-0.19764	-0.48531
O	1.09846	1.45450	-1.71568
H	1.02172	-1.99550	-1.11055
C	-0.97449	-1.11308	2.42439
C	-0.12648	-0.08436	2.48731

C	0.76555	0.44838	1.38447
C	-3.18364	-0.95431	-0.28430
O	-4.10738	-1.21531	0.46760
O	-3.36837	-0.50203	-1.54257
C	-4.71794	0.23887	-3.37227
C	-4.74088	-0.27006	-1.94347
C	0.65142	1.97653	1.29569
O	1.60431	2.72939	1.33386
O	-0.62151	2.37946	1.17184
C	-2.32816	4.03692	0.90545
C	-0.83453	3.80894	1.03280
C	-2.19575	-3.12680	1.56246
H	5.97735	0.96444	-2.19850
H	6.62966	-1.01146	-0.82981
H	4.89840	-2.32060	0.39118
H	3.57298	1.64572	-2.29734
H	-0.10399	-3.51751	0.13696
H	0.64959	-2.97205	1.63332
H	-1.16062	0.51986	-1.24121
H	-1.53570	-1.37250	3.32058
H	-0.03420	0.45556	3.42870
H	1.80799	0.27290	1.67251
H	-5.74221	0.42566	-3.71215
H	-4.15424	1.17445	-3.44656
H	-4.26117	-0.49603	-4.04297
H	-5.29558	-1.20886	-1.85105
H	-5.19039	0.45387	-1.25655
H	-2.52721	5.10853	0.79799
H	-2.72905	3.52201	0.02658
H	-2.85756	3.67488	1.79261
H	-0.41410	4.30950	1.91006
H	-0.28728	4.15351	0.15047
H	-2.45525	-3.72189	0.68005
H	-1.70515	-3.79061	2.28411
H	-3.12034	-2.75130	2.00154

Originally assigned (incorrect) structure of 100{5g} ($CDCl_3$)

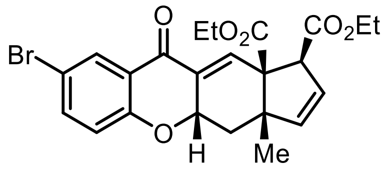

	Conf1	Conf2
Rel energy (kcal/mol):	0.00	0.54

C-nom	iGau	Exp	Calc	diff	1	2
C	10	191.00	180.21	-10.79	180.26	180.08
C	20	169.30	171.40	2.10	171.44	171.31
C	25	165.60	169.78	4.18	169.75	169.84
C	4	159.60	160.36	0.76	160.38	160.31
C	14	146.00	141.31	-4.69	141.02	142.03
C	17	142.30	137.81	-4.49	137.49	138.62
C	2	138.50	138.05	-0.45	138.08	137.99
C	6	132.00	131.99	-0.01	131.97	132.05
C	9	131.00	131.06	0.06	130.95	131.35
C	18	123.90	128.52	4.62	128.67	128.16
C	5	121.30	124.10	2.80	124.09	124.14
C	3	119.60	119.60	0.00	119.62	119.56
C	1	115.10	114.02	-1.08	114.03	114.00
C	8	79.30	73.81	-5.49	73.83	73.75
C	24	61.50	62.58	1.08	62.64	62.43
C	29	61.00	61.89	0.89	61.70	62.35
C	13	51.30	60.56	9.26	60.88	59.78
C	19	44.60	59.43	14.83	59.42	59.44
C	12	43.70	52.24	8.54	52.37	51.90
C	11	36.20	41.97	5.77	41.77	42.45
C	30	26.00	20.68	-5.32	20.75	20.52
C	28	14.30	12.25	-2.05	12.30	12.14
C	23	13.90	12.03	-1.87	12.05	11.97

13C chem shifts: RMSD=5.48ppm (MAE=3.96) N=23 {-10.79 14.83}

Fractions: 0.713 0.287

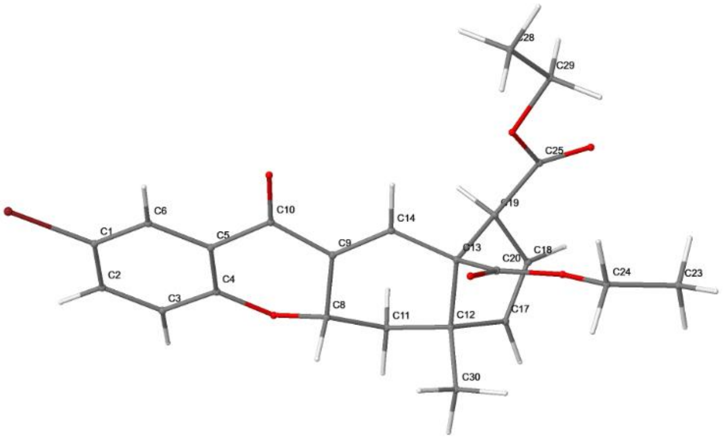

Conformer 1
Energy: -3913.02967 Hartree (Rel: 0.0 kcal/mol)
XYZ coordinates for conf 1:

C	-5.05224	0.08145	-0.16837
C	-5.13964	-1.30599	-0.35128
C	-3.98153	-2.07191	-0.39226
C	-2.72899	-1.45754	-0.24951
C	-2.64512	-0.06140	-0.08130
C	-3.82275	0.70373	-0.04220
O	-1.63357	-2.25471	-0.32391

C	-0.42113	-1.77267	0.30716
C	-0.15657	-0.31632	0.01807
C	-1.32385	0.60661	-0.04000
C	0.71486	-2.64179	-0.21777
C	2.10931	-2.19389	0.29732
C	2.35237	-0.65865	-0.07708
C	1.08818	0.16003	-0.13980
O	-1.20680	1.83007	-0.07869
H	-0.56450	-1.91561	1.38822
C	3.17658	-2.89057	-0.52779
C	3.69883	-2.11474	-1.47498
C	3.08958	-0.73410	-1.46800
C	3.31677	-0.04170	0.95714
O	4.52789	-0.11482	0.90143
O	2.65224	0.56178	1.95351
C	2.51001	1.78710	4.00579
C	3.45910	1.15044	3.00952
C	4.07386	0.38253	-1.79353
O	5.12373	0.22273	-2.38004
O	3.58665	1.58575	-1.43531
C	3.69516	3.96970	-1.23318
C	4.42440	2.73507	-1.72642
C	2.24434	-2.51428	1.79952
Br	-6.66006	1.11939	-0.10353
H	-6.10878	-1.78164	-0.45398
H	-4.02867	-3.14726	-0.52833
H	-3.73613	1.77757	0.07710
H	0.54223	-3.68773	0.05953
H	0.68927	-2.58752	-1.31190
H	1.20535	1.22421	-0.32426
H	3.42375	-3.93547	-0.36100
H	4.44497	-2.40168	-2.20695
H	2.32609	-0.66519	-2.26048
H	3.08583	2.24739	4.81565
H	1.90381	2.56361	3.52877
H	1.83904	1.04019	4.44203
H	4.06565	0.36061	3.46249
H	4.13675	1.88087	2.55822
H	4.30012	4.85920	-1.43886
H	2.73084	4.08484	-1.73846
H	3.51826	3.91626	-0.15404
H	5.38634	2.59576	-1.22454
H	4.60559	2.76719	-2.80484
H	1.55076	-1.93113	2.41323
H	2.03473	-3.57675	1.96693
H	3.26033	-2.31940	2.15703

Conformer 2
Energy: -3913.03053 Hartree (Rel: 0.5 kcal/mol)
XYZ coordinates for conf 2:

C	-5.28991	0.09283	-0.00740
C	-5.35324	-1.23468	-0.45425
C	-4.18185	-1.95751	-0.64097
C	-2.93997	-1.35995	-0.38138
C	-2.88046	-0.02121	0.05304
C	-4.07131	0.70093	0.23761
O	-1.83094	-2.10817	-0.60787
C	-0.62485	-1.73465	0.10384
C	-0.38696	-0.24564	0.10238
C	-1.57121	0.65059	0.22203
C	0.52422	-2.46355	-0.58076
C	1.91553	-2.09878	0.00615
C	2.12927	-0.51758	-0.05483
C	0.84814	0.27398	0.04524
O	-1.47615	1.86078	0.41531
H	-0.76064	-2.08632	1.13705
C	2.97885	-2.60210	-0.95380
C	3.47676	-1.64600	-1.73490
C	2.85933	-0.30060	-1.43810
C	3.00926	-0.06544	1.12747
O	2.56503	0.43784	2.13998

O	4.31242	-0.31592	0.93007
C	6.62633	-0.25516	1.53073
C	5.22272	0.09373	1.98592
C	3.83418	0.86694	-1.52969
O	4.87740	0.84736	-2.14954
O	3.35570	1.95278	-0.89479
C	3.45629	4.22353	-0.14376
C	4.17589	3.15066	-0.93756
C	2.06643	-2.69945	1.41783
Br	-6.91578	1.07010	0.25426
H	-6.31409	-1.69888	-0.64749
H	-4.21028	-2.98718	-0.98173
H	-4.00334	1.73300	0.56180
H	0.37294	-3.54663	-0.51087
H	0.49100	-2.19915	-1.64361
H	0.94858	1.35474	0.08551
H	3.23673	-3.65641	-1.00599
H	4.21098	-1.77198	-2.52240
H	2.08908	-0.07335	-2.19389
H	7.34756	0.05748	2.29323
H	6.73212	-1.33371	1.37692
H	6.86926	0.25403	0.59272
H	5.09588	1.16768	2.15139
H	4.94262	-0.42626	2.90662
H	4.05170	5.14272	-0.14642
H	2.47777	4.44566	-0.58120
H	3.30940	3.90937	0.89453
H	5.15658	2.91248	-0.51496
H	4.31799	3.43485	-1.98439
H	1.38461	-2.23653	2.13855
H	1.85082	-3.77340	1.38772
H	3.08694	-2.57832	1.79333

Revised structure of 100{5g}, i.e. 102{5g-rev} ($CDCl_3$)

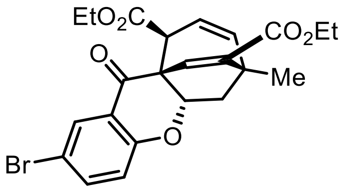

					Conf1	Conf2
	Rel energy (kcal/mol):				0.00	0.28

C-nom	iGau	Exp	Calc	diff	1	2
C	10	191.00	193.73	2.73	[193.73	193.72]
C	25	169.30	168.17	-1.13	[168.31	167.95]
C	20	165.60	165.61	0.01	[165.60	165.62]
C	4	159.60	160.67	1.07	[160.72	160.58]
C	13	146.00	147.87	1.87	[148.27	147.22]
C	17	142.30	142.41	0.11	[142.70	141.95]
C	2	138.50	138.75	0.25	[138.71	138.82]
C	14	132.00	136.08	4.08	[135.57	136.90]
C	6	131.00	132.76	1.76	[132.63	132.97]
C	18	123.90	124.49	0.59	[124.64	124.24]
C	5	121.30	122.03	0.73	[122.15	121.83]
C	3	119.60	119.19	-0.41	[119.26	119.09]
C	1	115.10	114.85	-0.25	[114.81	114.92]
C	8	79.30	79.45	0.15	[79.53	79.31]
C	24	61.50	61.90	0.40	[61.93	61.84]
C	29	61.00	62.00	1.00	[61.70	62.48]
C	9	51.30	52.39	1.09	[52.46	52.27]
C	11	44.60	45.25	0.65	[45.20	45.34]
C	19	43.70	45.33	1.63	[44.45	46.74]
C	12	36.20	36.39	0.19	[36.41	36.37]
C	30	26.00	25.74	-0.26	[25.70	25.80]
C	23	14.30	12.44	-1.86	[12.44	12.44]
C	28	13.90	11.91	-1.99	[11.84	12.01]

13C chem shifts: RMSD=1.43ppm (MAE=1.05) N=23 {-1.99 4.08}

Fractions: 0.615 0.385

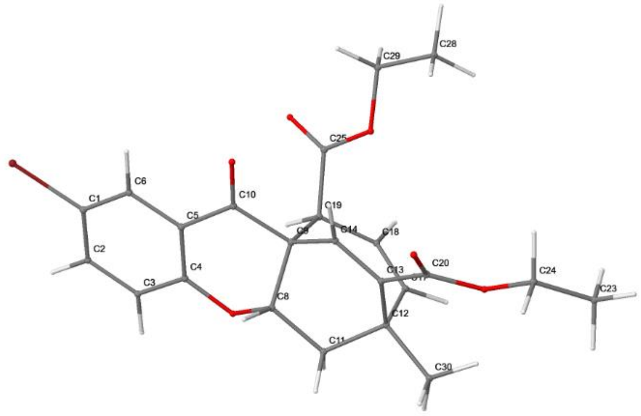

Conformer 1
Energy: -3913.01696 Hartree (Rel: 0.0 kcal/mol)
XYZ coordinates for conf 1:

C	-4.28046	-0.86842	0.24504
C	-4.16080	-1.85586	-0.74412
C	-2.90329	-2.23611	-1.19464
C	-1.75857	-1.62722	-0.66353
C	-1.88140	-0.61709	0.31278
C	-3.15954	-0.25117	0.77076
O	-0.54927	-2.04673	-1.12345
C	0.55726	-1.73206	-0.25265

C	0.61713	-0.21718	0.05038
C	-0.68669	0.11038	0.80474
C	1.85017	-2.30663	-0.83967
C	3.06302	-1.32151	-0.80182
C	3.01186	-0.53119	0.52216
C	1.83868	0.00463	0.89696
O	-0.73130	0.93229	1.70791
H	0.34448	-2.22385	0.70632
C	2.91692	-0.31964	-1.94585
C	1.88888	0.51129	-2.12713
C	0.64746	0.65329	-1.27221
C	4.18791	-0.19860	1.38681
O	4.11475	-0.01926	2.59008
O	5.33134	-0.05823	0.69055
C	7.66452	0.43999	0.48714
C	6.50339	0.32192	1.45583
C	0.44945	2.13380	-0.92194
O	1.33814	2.88881	-0.58578
O	-0.84275	2.48620	-1.02917
C	-2.67079	4.01786	-0.82168
C	-1.17835	3.83930	-0.62175
C	4.33229	-2.16847	-1.00767
Br	-6.02236	-0.36945	0.86083
H	-5.04809	-2.32932	-1.14960
H	-2.78950	-3.01076	-1.94559
H	-3.23639	0.52488	1.52363
H	2.11389	-3.21785	-0.29263
H	1.66847	-2.59459	-1.87945
H	1.74830	0.60586	1.79480
H	3.73077	-0.29567	-2.66860
H	1.91227	1.18608	-2.98131
H	-0.21990	0.34900	-1.86909
H	8.56718	0.73591	1.03228
H	7.86154	-0.51519	-0.01046
H	7.46108	1.19640	-0.27760
H	6.29448	1.26822	1.96337
H	6.68161	-0.43847	2.22233
H	-2.96319	5.02812	-0.51635
H	-2.94537	3.88272	-1.87287
H	-3.23475	3.29882	-0.21910
H	-0.88399	3.96338	0.42444
H	-0.59344	4.53879	-1.22602
H	4.51294	-2.82316	-0.14797
H	4.20244	-2.80337	-1.89195
H	5.21608	-1.54743	-1.15625

Conformer 2
Energy: -3913.01740 Hartree (Rel: 0.3 kcal/mol)
XYZ coordinates for conf 2:

C	4.62491	-0.41839	-0.23531
C	4.60008	-1.56080	0.57819
C	3.38472	-2.12707	0.94049
C	2.18735	-1.55156	0.49613
C	2.21299	-0.38951	-0.30277
C	3.45032	0.16723	-0.67248
O	1.02387	-2.15235	0.86508
C	-0.10841	-1.81115	0.03941
C	-0.31335	-0.28015	-0.02698
C	0.95430	0.28858	-0.69361
C	-1.34026	-2.58648	0.51706
C	-2.64348	-1.72734	0.60607
C	-2.66537	-0.74609	-0.58440
C	-1.54741	-0.05123	-0.85264
O	0.91960	1.24915	-1.44831
H	0.14695	-2.12679	-0.98129
C	-2.59920	-0.89921	1.88963
C	-1.65447	-0.01392	2.21379
C	-0.42498	0.36450	1.41408
C	-3.86273	-0.39643	-1.41177
O	-3.79871	-0.00248	-2.56364
O	-5.02253	-0.50235	-0.73624

C	-7.39839	-0.29540	-0.51997
C	-6.22127	-0.12799	-1.46172
C	-0.28231	1.89113	1.33446
O	0.73522	2.49000	1.62114
O	-1.40834	2.47904	0.90715
C	-2.72844	4.37051	0.26570
C	-1.36148	3.92162	0.74398
C	-3.82374	-2.71462	0.66325
Br	6.31122	0.33726	-0.73232
H	5.52849	-2.00735	0.91658
H	3.34558	-3.02042	1.55476
H	3.45277	1.06166	-1.28464
H	-1.51459	-3.42882	-0.16058
H	-1.13189	-3.00472	1.50634
H	-1.51257	0.68246	-1.65001
H	-3.41535	-1.06028	2.59205
H	-1.74186	0.51445	3.16212
H	0.45942	0.04042	1.97379
H	-8.32227	-0.01171	-1.03523
H	-7.49463	-1.33491	-0.19009
H	-7.28761	0.34200	0.36319
H	-6.11226	0.90564	-1.80309
H	-6.30773	-0.76621	-2.34642
H	-2.72916	5.45699	0.12785
H	-2.98187	3.90155	-0.69031
H	-3.50318	4.11511	0.99584
H	-1.09138	4.37052	1.70435
H	-0.57318	4.15946	0.02385
H	-3.93918	-3.24671	-0.28740
H	-3.63186	-3.45976	1.44417
H	-4.76438	-2.21216	0.89019

Originally assigned correct structure of 104{7b} ($CDCl_3$)

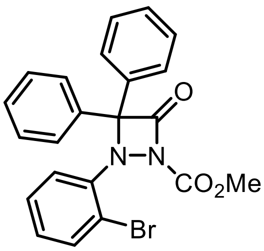

					Conf1	Conf2	Conf3	Conf4
Rel energy (kcal/mol):					0.00	0.01	3.56	3.56

C-nom	iGau	Exp	Calc	diff	1	2	3	4
C	4	165.30	167.21	1.91	[167.15	167.28	164.30	164.30]
C	25	148.70	149.69	0.99	[149.63	149.75	150.25	150.25]
C	6	143.80	143.45	-0.35	[143.43	143.49	140.44	140.44]
C	8	135.80	134.97	-0.83	[133.52	136.43	134.02	137.60]
C	7	133.30	134.95	1.65	[136.35	133.53	137.60	134.02]
C	22	132.60	132.47	-0.13	[132.46	132.46	134.56	134.56]
C	9	130.00	128.78	-1.22	[129.62	127.93	130.16	127.12]
C	13	130.00	128.78	-1.22	[129.62	127.93	130.16	127.12]
C	11	129.40	128.47	-0.93	[128.92	128.01	128.71	127.88]
C	16	128.80	128.45	-0.35	[128.00	128.91	127.88	128.71]
C	14	128.60	128.77	0.17	[127.95	129.60	127.12	130.16]
C	18	128.60	128.77	0.17	[127.95	129.60	127.12	130.16]
C	10	128.50	127.55	-0.95	[127.63	127.46	127.26	127.81]
C	12	128.50	127.55	-0.95	[127.63	127.46	127.26	127.81]
C	17	128.10	127.54	-0.56	[127.45	127.64	127.81	127.26]
C	15	128.10	127.54	-0.56	[127.45	127.64	127.81	127.26]
C	20	127.40	127.48	0.08	[127.47	127.49	127.48	127.49]
C	21	126.90	126.52	-0.38	[126.49	126.55	126.74	126.74]
C	19	122.20	123.74	1.54	[123.70	123.75	130.65	130.65]
C	23	118.60	119.44	0.84	[119.39	119.50	116.94	116.93]
C	1	95.50	98.99	3.49	[99.00	98.98	97.18	97.20]
C	28	54.30	53.54	-0.76	[53.52	53.56	54.04	54.04]

13C chem shifts: RMSD=1.18ppm (MAE=0.91) N=22 {-1.22 3.49}

Fractions: 0.502 0.496 0.001 0.001

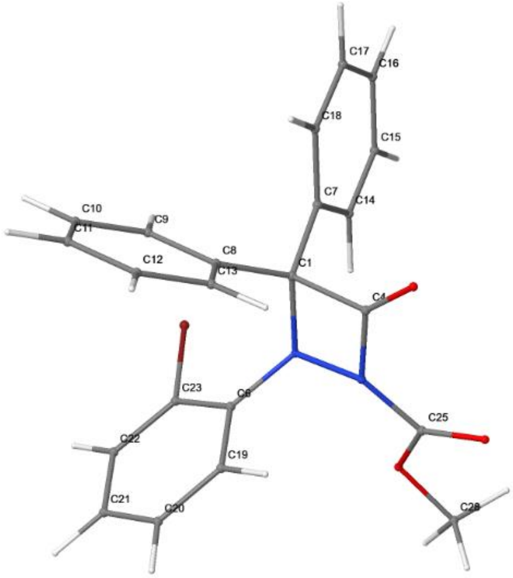

Conformer 1
Energy: -3755.09216 Hartree (Rel: 0.0 kcal/mol)
XYZ coordinates for conf 1:

C	0.92917	0.15098	-0.69428
N	-0.43343	0.62061	-0.09914
N	-0.56398	1.53028	-1.22479
C	0.66885	1.20812	-1.79925
O	1.27440	1.65891	-2.73483
C	-1.51983	-0.31239	0.00305
C	2.11193	0.60539	0.15704
C	0.99132	-1.29312	-1.14640
C	1.23331	-2.30538	-0.20410
C	1.26321	-3.64300	-0.59464
C	1.06549	-3.98884	-1.93457
C	0.83608	-2.98856	-2.87961
C	0.79603	-1.64789	-2.48829
C	1.95055	1.48080	1.23781
C	3.06455	1.94141	1.94288
C	4.35007	1.54622	1.56885
C	4.51732	0.68510	0.48151
C	3.40663	0.21850	-0.22171
C	-2.41346	-0.55135	-1.05081
C	-3.48812	-1.42529	-0.90145
C	-3.68511	-2.09315	0.30756
C	-2.80627	-1.87227	1.36759
C	-1.74309	-0.98316	1.21601
Br	-0.60253	-0.69416	2.72424
C	-1.28286	2.73291	-1.23213
O	-1.14940	3.57121	-2.09625
O	-2.11822	2.77745	-0.19064
C	-2.93014	3.96848	-0.10750
H	1.39467	-2.04218	0.83655
H	1.44635	-4.41520	0.14728
H	1.09584	-5.03133	-2.23900
H	0.69105	-3.24610	-3.92493
H	0.63297	-0.88073	-3.23850
H	0.95209	1.78321	1.52945
H	2.92366	2.61164	2.78656
H	5.21525	1.90653	2.11860
H	5.51316	0.37364	0.17856
H	3.54650	-0.45034	-1.06516
H	-2.26161	-0.03917	-1.99449
H	-4.16538	-1.58693	-1.73475
H	-4.51907	-2.77682	0.43378
H	-2.94782	-2.37574	2.31743
H	-3.54863	3.82966	0.77796
H	-3.55035	4.06628	-1.00120
H	-2.29616	4.85131	-0.00093

Conformer 2
Energy: -3755.09216 Hartree (Rel: 0.0 kcal/mol)
XYZ coordinates for conf 2:

C	-0.93505	0.12792	-0.68902
N	0.42753	0.61950	-0.11288
N	0.53530	1.52024	-1.24817
C	-0.69810	1.17446	-1.80834
O	-1.31813	1.60710	-2.74281
C	1.52603	-0.30000	-0.01399
C	-0.98955	-1.32192	-1.12401
C	-2.11299	0.58127	0.17004
C	-3.40817	0.17848	-0.18951
C	-4.51510	0.64362	0.52069
C	-4.34353	1.51886	1.59595
C	-3.05745	1.92981	1.95087
C	-1.94740	1.47088	1.23864
C	-1.20305	-2.32553	-0.16572
C	-1.22820	-3.66786	-0.54036
C	-1.05443	-4.02712	-1.88006
C	-0.85322	-3.03560	-2.84067
C	-0.81786	-1.69024	-2.46546
C	2.40667	-0.54452	-1.07756
C	3.49201	-1.40577	-0.93220
C	3.71325	-2.05484	0.28294
C	2.84835	-1.82749	1.35289

C	1.77411	-0.95113	1.20486
Br	0.65455	-0.65094	2.72663
C	1.22557	2.73974	-1.26611
O	1.06383	3.57192	-2.13153
O	2.07043	2.80664	-0.23363
C	2.85517	4.01652	-0.16147
H	-3.55159	-0.50118	-1.02363
H	-5.51141	0.31987	0.23254
H	-5.20572	1.87797	2.15115
H	-2.91324	2.61119	2.78500
H	-0.94839	1.78543	1.51541
H	-1.34611	-2.05194	0.87495
H	-1.38917	-4.43314	0.21377
H	-1.08152	-5.07318	-2.17229
H	-0.72690	-3.30380	-3.88575
H	-0.67718	-0.93032	-3.22743
H	2.23617	-0.04649	-2.02550
H	4.15845	-1.57288	-1.77310
H	4.55571	-2.72859	0.40610
H	3.00938	-2.31587	2.30750
H	3.48718	3.89333	0.71676
H	2.20191	4.88434	-0.04820
H	3.46222	4.12744	-1.06260

Conformer 3
Energy: -3755.09781 Hartree (Rel: 3.6 kcal/mol)
XYZ coordinates for conf 3:

C	-1.15747	-0.05084	-0.28009
N	-0.09193	-0.51078	0.73647
N	0.36613	-1.48723	-0.21941
C	-0.60930	-1.19564	-1.17538
O	-0.91168	-1.70925	-2.22018
C	0.85500	0.40745	1.29603
C	-2.57077	-0.38452	0.19135
C	-1.00565	1.35919	-0.81810
C	-0.40062	1.61279	-2.05606
C	-0.26221	2.92303	-2.52090
C	-0.71948	3.99341	-1.75185
C	-1.32578	3.74905	-0.51624
C	-1.47495	2.44174	-0.05666
C	-3.65133	-0.07401	-0.64907
C	-4.95254	-0.41312	-0.28028
C	-5.19298	-1.06427	0.93298
C	-4.12132	-1.38114	1.76863
C	-2.81494	-1.05143	1.39759
C	0.43710	0.95798	2.52129
C	1.21329	1.87202	3.22427
C	2.46723	2.23442	2.73030
C	2.91354	1.69011	1.52908
C	2.11408	0.79963	0.80577
Br	2.86180	0.19621	-0.85620
C	1.06758	-2.63457	0.16009
O	1.53235	-2.78680	1.26885
O	1.15587	-3.47296	-0.87600
C	1.87491	-4.69651	-0.61447
H	-0.04776	0.79081	-2.66978
H	0.20212	3.10223	-3.48646
H	-0.61057	5.01195	-2.11396
H	-1.68945	4.57610	0.08708
H	-1.96288	2.25850	0.89572
H	-3.47355	0.43618	-1.59088
H	-5.77895	-0.16574	-0.94082
H	-6.20781	-1.32315	1.22212
H	-4.29686	-1.89024	2.71243
H	-1.98018	-1.31190	2.03842
H	-0.52090	0.63358	2.91479
H	0.85061	2.27795	4.16369
H	3.09997	2.93003	3.27302
H	3.88503	1.96240	1.13170
H	1.85447	-5.24422	-1.55557
H	1.37904	-5.26721	0.17393

H	2.90273	-4.47514	-0.31865

Conformer 4
Energy: -3755.09783 Hartree (Rel: 3.6 kcal/mol)
XYZ coordinates for conf 4:

C	1.15748	-0.05083	-0.27993
N	0.09178	-0.51060	0.73665
N	-0.36621	-1.48711	-0.21916
C	0.60919	-1.19561	-1.17521
O	0.91146	-1.70929	-2.22001
C	-0.85515	0.40793	1.29585
C	1.00607	1.35925	-0.81788
C	2.57065	-0.38489	0.19154
C	3.65137	-0.07422	-0.64861
C	4.95247	-0.41369	-0.27979
C	5.19262	-1.06543	0.93323
C	4.12080	-1.38251	1.76857
C	2.81453	-1.05240	1.39751
C	1.47540	2.44172	-0.05637
C	1.32670	3.74905	-0.51604
C	0.72086	3.99353	-1.75184
C	0.26355	2.92323	-2.52099
C	0.40147	1.61298	-2.05607
C	-0.43715	0.95903	2.52083
C	-1.21339	1.87323	3.22354
C	-2.46748	2.23519	2.72964
C	-2.91386	1.69037	1.52868
C	-2.11431	0.79978	0.80559
Br	-2.86197	0.19596	-0.85629
C	-1.06783	-2.63434	0.16022
O	-1.53295	-2.78649	1.26886
O	-1.15588	-3.47282	-0.87584
C	-1.87530	-4.69618	-0.61449
H	3.47375	0.43639	-1.59024
H	5.77902	-0.16618	-0.94009
H	6.20736	-1.32461	1.22239
H	4.29612	-1.89208	2.71217
H	1.97963	-1.31301	2.03812
H	1.96298	2.25844	0.89619
H	1.69040	4.57602	0.08737
H	0.61232	5.01209	-2.11402
H	-0.20044	3.10252	-3.48670
H	0.04859	0.79109	-2.66987
H	0.52093	0.63493	2.91439
H	-0.85060	2.27963	4.16272
H	-3.10030	2.93083	3.27223
H	-3.88543	1.96237	1.13131
H	-1.85538	-5.24358	-1.55578
H	-2.90293	-4.47454	-0.31822
H	-1.37935	-5.26734	0.17354

Originally assigned correct structure of 105{7d} ($CDCl_3$)

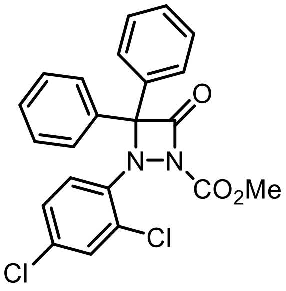

	Conf1	Conf2	Conf3	Conf4
Rel energy (kcal/mol):	0.00	0.00	0.00	0.00

C-nom	iGau	Exp	Calc	diff	1	2	3	4
C	4	165.00	165.96	0.96	[165.96	165.96	165.95	165.97]
C	25	148.70	150.28	1.58	[150.28	150.28	150.27	150.28]
C	6	141.50	140.97	-0.53	[140.97	140.97	140.96	140.97]
C	8	135.50	134.38	-1.12	[133.12	135.65	133.12	135.65]
C	7	132.50	134.38	1.88	[135.65	133.11	135.65	133.11]
C	21	131.10	131.63	0.53	[131.63	131.63	131.63	131.63]
C	23	129.80	130.49	0.69	[130.49	130.49	130.49	130.49]
C	14	129.80	128.69	-1.11	[127.84	129.55	127.84	129.55]
C	18	129.80	128.69	-1.11	[127.84	129.55	127.84	129.55]
C	16	129.80	128.76	-1.04	[128.22	129.30	128.22	129.30]
C	22	128.90	128.53	-0.37	[128.53	128.53	128.53	128.53]
C	11	128.80	128.76	-0.04	[129.30	128.22	129.30	128.22]
C	9	128.60	128.70	0.10	[129.55	127.84	129.55	127.84]
C	13	128.60	128.70	0.10	[129.55	127.84	129.55	127.84]
C	15	128.30	127.73	-0.57	[127.65	127.81	127.65	127.81]
C	17	128.30	127.73	-0.57	[127.65	127.81	127.65	127.81]
C	10	128.30	127.73	-0.57	[127.81	127.65	127.81	127.65]
C	12	128.30	127.73	-0.57	[127.81	127.65	127.81	127.65]
C	20	127.00	126.83	-0.17	[126.83	126.83	126.83	126.83]
C	19	122.30	123.72	1.42	[123.72	123.72	123.72	123.71]
C	1	95.60	98.42	2.82	[98.42	98.43	98.43	98.42]
C	28	54.50	54.23	-0.27	[54.23	54.23	54.23	54.23]

13C chem shifts: RMSD=1.05ppm (MAE=0.82) N=22 {-1.12 2.82}

Fractions: 0.250 0.250 0.250 0.250

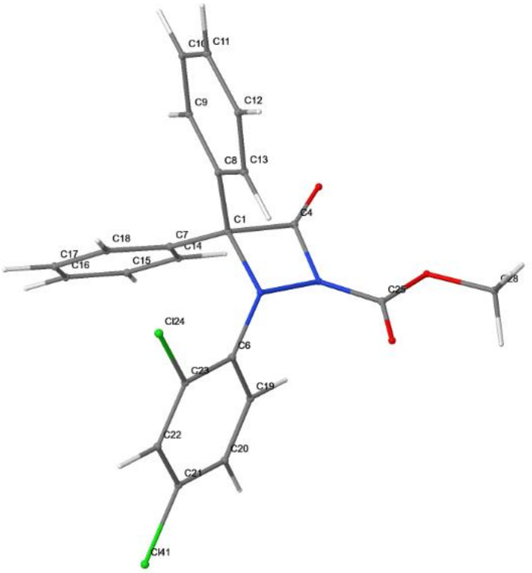

Conformer 1
Energy: -2103.47792 Hartree (Rel: 0.0 kcal/mol)

XYZ coordinates for conf 1:

C	1.18554	-0.34923	-0.43418
N	0.28582	0.67713	0.31818
N	0.73974	1.69830	-0.60086
C	1.62839	0.86379	-1.29247
O	2.44394	1.07486	-2.14919
C	-1.13531	0.50958	0.32855
C	2.36100	-0.81205	0.42120
C	0.45315	-1.45328	-1.16917
C	0.07389	-2.61571	-0.47945
C	-0.63887	-3.62326	-1.12793
C	-0.97255	-3.49035	-2.47854
C	-0.58960	-2.34366	-3.17534
C	0.11648	-1.32931	-2.52464
C	3.25097	-1.75947	-0.10799
C	4.38285	-2.14317	0.61124
C	4.64203	-1.58427	1.86527
C	3.76475	-0.63409	2.39071
C	2.63373	-0.24248	1.67094
C	-1.97525	1.05607	-0.65103
C	-3.35951	0.92401	-0.58342
C	-3.92052	0.21847	0.47865
C	-3.12031	-0.34101	1.47165
C	-1.73771	-0.18223	1.39346
Cl	-0.76357	-0.86347	2.68260
C	0.77058	3.05294	-0.23131
O	0.19917	3.48350	0.74503
O	1.47735	3.74510	-1.12735
C	1.58048	5.16192	-0.86694
H	0.34014	-2.73089	0.56633
H	-0.92881	-4.51465	-0.57885
H	-1.52216	-4.27889	-2.98487
H	-0.83505	-2.23556	-4.22797
H	0.42024	-0.45128	-3.08589
H	3.05768	-2.19932	-1.08139
H	5.06085	-2.88044	0.19033
H	5.52161	-1.88679	2.42684
H	3.95802	-0.19228	3.36442
H	1.95218	0.49537	2.07700
H	-1.53547	1.59886	-1.47978
H	-3.99005	1.35773	-1.35135
Cl	-5.66527	0.03599	0.58156
H	-3.55883	-0.87979	2.30279
H	0.58713	5.61527	-0.84578
H	2.16876	5.55946	-1.69256
H	2.08611	5.33549	0.08542

Conformer 2

Energy: -2103.47792 Hartree (Rel: 0.0 kcal/mol)

XYZ coordinates for conf 2:

C	-1.18544	-0.34948	-0.43429
N	-0.28587	0.67713	0.31798
N	-0.73991	1.69812	-0.60113
C	-1.62845	0.86344	-1.29268
O	-2.44405	1.07432	-2.14940
C	1.13529	0.50980	0.32846
C	-0.45289	-1.45347	-1.16918
C	-2.36078	-0.81237	0.42119
C	-3.25038	-1.76033	-0.10762
C	-4.38218	-2.14410	0.61171
C	-4.64166	-1.58471	1.86544
C	-3.76474	-0.63399	2.39052
C	-2.63381	-0.24232	1.67067
C	-0.07341	-2.61577	-0.47934
C	0.63959	-3.62322	-1.12770
C	0.97331	-3.49035	-2.47830
C	0.59013	-2.34381	-3.17523
C	-0.11621	-1.32956	-2.52465
C	1.97522	1.05631	-0.65113
C	3.35949	0.92440	-0.58341
C	3.92052	0.21902	0.47876

C	3.12032	-0.34043	1.47178
C	1.73770	-0.18180	1.39349
Cl	0.76352	-0.86292	2.68265
C	-0.77129	3.05275	-0.23151
O	-0.20048	3.48340	0.74513
O	-1.47787	3.74476	-1.12780
C	-1.58167	5.16151	-0.86725
H	-3.05686	-2.20057	-1.08079
H	-5.05988	-2.88180	0.19106
H	-5.52118	-1.88727	2.42709
H	-3.95823	-0.19181	3.36403
H	-1.95252	0.49591	2.07648
H	-0.33969	-2.73091	0.56643
H	0.92969	-4.51450	-0.57853
H	1.52314	-4.27880	-2.98453
H	0.83560	-2.23576	-4.22786
H	-0.42018	-0.45168	-3.08602
H	1.53543	1.59901	-1.47993
H	3.99004	1.35811	-1.35134
Cl	5.66528	0.03671	0.58175
H	3.55884	-0.87906	2.30301
H	-2.16976	5.55892	-1.69306
H	-0.58852	5.61525	-0.84561
H	-2.08778	5.33474	0.08492

Conformer 3
Energy: -2103.47792 Hartree (Rel: 0.0 kcal/mol)
XYZ coordinates for conf 3:

C	1.18578	-0.34900	-0.43426
N	0.28578	0.67706	0.31823
N	0.73949	1.69840	-0.60053
C	1.62816	0.86429	-1.29254
O	2.44351	1.07573	-2.14936
C	-1.13532	0.50921	0.32858
C	2.36140	-0.81140	0.42107
C	0.45371	-1.45335	-1.16908
C	0.11677	-1.32957	-2.52448
C	-0.58911	-2.34422	-3.17501
C	-0.97151	-3.49098	-2.47809
C	-0.63748	-3.62373	-1.12753
C	0.07501	-2.61593	-0.47924
C	3.25161	-1.75858	-0.10815
C	4.38363	-2.14196	0.61103
C	4.64272	-1.58296	1.86503
C	3.76520	-0.63302	2.39050
C	2.63403	-0.24174	1.67079
C	-1.97532	1.05559	-0.65105
C	-3.35953	0.92326	-0.58347
C	-3.92045	0.21759	0.47859
C	-3.12020	-0.34172	1.47161
C	-1.73761	-0.18267	1.39348
Cl	-0.76338	-0.86355	2.68268
C	0.76930	3.05307	-0.23128
O	0.19741	3.48340	0.74489
O	1.47581	3.74556	-1.12729
C	1.57803	5.16244	-0.86709
H	0.42017	-0.45151	-3.08588
H	-0.83481	-2.23622	-4.22760
H	-1.52099	-4.27973	-2.98424
H	-0.92695	-4.51525	-0.57841
H	0.34153	-2.73092	0.56649
H	3.05839	-2.19849	-1.08154
H	5.06179	-2.87905	0.19007
H	5.52239	-1.88524	2.42658
H	3.95838	-0.19114	3.36420
H	1.95229	0.49591	2.07691
H	-1.53559	1.59849	-1.47975
H	-3.99018	1.35687	-1.35137
Cl	-5.66517	0.03481	0.58138
H	-3.55861	-0.88060	2.30275
H	2.16532	5.56041	-1.69321

H	2.08434	5.33649	0.08483
H	0.58437	5.61506	-0.84511

Conformer 4
Energy: -2103.47792 Hartree (Rel: 0.0 kcal/mol)
XYZ coordinates for conf 4:

C	-1.18514	-0.34982	-0.43434
N	-0.28581	0.67699	0.31776
N	-0.74005	1.69788	-0.60136
C	-1.62825	0.86284	-1.29306
O	-2.44376	1.07344	-2.14991
C	1.13536	0.51005	0.32826
C	-0.45245	-1.45399	-1.16881
C	-2.36062	-0.81248	0.42111
C	-3.25030	-1.76037	-0.10773
C	-4.38218	-2.14398	0.61154
C	-4.64169	-1.58449	1.86523
C	-3.76472	-0.63383	2.39030
C	-2.63368	-0.24232	1.67051
C	-0.11485	-1.33002	-2.52404
C	0.59172	-2.34436	-3.17428
C	0.97419	-3.49103	-2.47723
C	0.63954	-3.62397	-1.12685
C	-0.07364	-2.61645	-0.47885
C	1.97520	1.05695	-0.65121
C	3.35949	0.92546	-0.58341
C	3.92067	0.22011	0.47872
C	3.12059	-0.33973	1.47158
C	1.73792	-0.18154	1.39323
Cl	0.76388	-0.86310	2.68223
C	-0.77241	3.05241	-0.23152
O	-0.20275	3.48325	0.74570
O	-1.47858	3.74419	-1.12835
C	-1.58362	5.16078	-0.86757
H	-3.05676	-2.20067	-1.08088
H	-5.05993	-2.88163	0.19088
H	-5.52126	-1.88694	2.42684
H	-3.95823	-0.19155	3.36376
H	-1.95237	0.49590	2.07630
H	-0.41824	-0.45203	-3.08554
H	0.83790	-2.23621	-4.22674
H	1.52420	-4.27956	-2.98315
H	0.92908	-4.51539	-0.57761
H	-0.34060	-2.73160	0.56675
H	1.53527	1.59962	-1.47994
H	3.98998	1.35947	-1.35121
Cl	5.66549	0.03839	0.58180
H	3.55918	-0.87836	2.30278
H	-2.09191	5.33347	0.08354
H	-2.17011	5.55820	-1.69452
H	-0.59075	5.61502	-0.84358

Originally assigned (incorrect) structure of 106{7a} ($CDCl_3$)

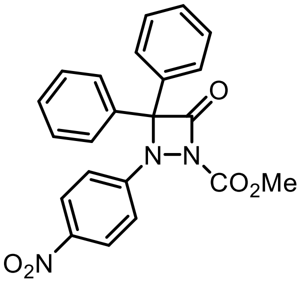

	Conf1	Conf2	Conf3	Conf4
Rel energy (kcal/mol):	0.00	0.00	0.00	0.00

**C-nom**	**iGau**	**Exp**	**Calc**	**diff**		**1**	**2**	**3**	**4**	
C-C	4	165.30	166.88	1.58	[	166.88	166.88	166.89	166.89	]
C-C	6	158.60	151.97	-6.63	[	151.97	151.97	151.96	151.96	]
C-C	24	144.10	151.22	7.12	[	151.22	151.22	151.22	151.22	]
C-C	21	142.60	143.27	0.67	[	143.27	143.27	143.26	143.27	]
C-C	8	134.30	134.20	-0.10	[	135.17	133.23	135.17	133.23	]
C-C	7	134.30	134.20	-0.10	[	133.23	135.17	133.23	135.17	]
C-C	1	93.00	98.02	5.02	[	98.03	98.03	98.01	98.01	]
C-CH	11	129.70	129.12	-0.58	[	128.55	129.69	128.55	129.69	]
C-CH	16	129.70	129.12	-0.58	[	129.69	128.55	129.69	128.55	]
C-CH	14	128.80	128.73	-0.07	[	130.11	127.36	130.10	127.36	]
C-CH	9	128.80	128.73	-0.07	[	127.36	130.11	127.36	130.10	]
C-CH	13	128.80	128.73	-0.07	[	127.36	130.11	127.36	130.10	]
C-CH	18	128.80	128.73	-0.07	[	130.11	127.36	130.10	127.36	]
C-CH	10	128.10	127.98	-0.12	[	128.02	127.94	128.02	127.94	]
C-CH	12	128.10	127.98	-0.12	[	128.02	127.94	128.02	127.94	]
C-CH	15	128.10	127.98	-0.12	[	127.94	128.02	127.94	128.02	]
C-CH	17	128.10	127.98	-0.12	[	127.94	128.02	127.94	128.02	]
C-CH	20	124.90	124.63	-0.27	[	124.63	124.63	124.64	124.64	]
C-CH	22	124.90	124.63	-0.27	[	124.63	124.63	124.64	124.64	]
C-CH	19	116.20	118.06	1.86	[	118.06	118.06	118.05	118.05	]
C-CH	23	116.20	118.06	1.86	[	118.06	118.06	118.05	118.05	]
C-CH3	27	53.70	54.65	0.95	[	54.65	54.65	54.65	54.65	]

13C chem shifts: RMSD=2.45ppm (MAE=1.29) N=22 {-6.63 7.12}

Fractions: 0.250 0.250 0.250 0.250

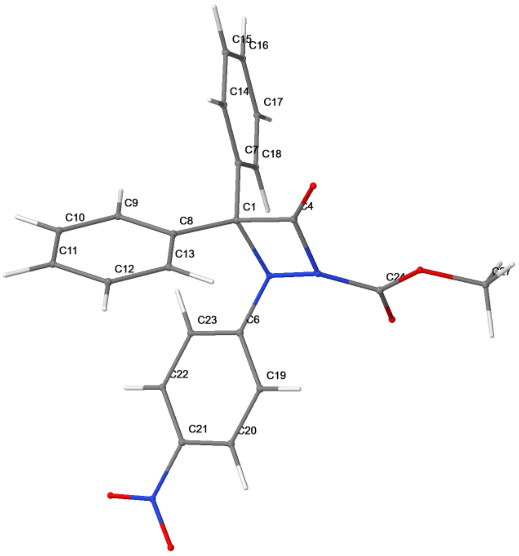

Conformer 1

Energy: -1388.80164 Hartree (Rel: 0.0 kcal/mol)
XYZ coordinates for conf 1:

C	-1.28396	-0.40710	-0.27211
N	-0.35866	0.65853	0.34903
N	-0.87416	1.64177	-0.57508
C	-1.81178	0.75920	-1.14871
O	-2.69134	0.91683	-1.94842
C	1.04060	0.50049	0.40456
C	-0.59222	-1.52574	-1.03204
C	-2.36526	-0.85504	0.70249
C	-3.46237	-1.57431	0.20247
C	-4.48504	-1.98068	1.05798
C	-4.42379	-1.67705	2.42153
C	-3.33776	-0.95746	2.92060
C	-2.31454	-0.54015	2.06446
C	-0.45757	-2.80110	-0.46495
C	0.21628	-3.81414	-1.14874
C	0.76106	-3.56780	-2.41020
C	0.63071	-2.30119	-2.98453
C	-0.03918	-1.28741	-2.30017
C	1.93038	1.35903	-0.26164
C	3.30185	1.19450	-0.11282
C	3.78420	0.16856	0.70027
C	2.91467	-0.69322	1.37564
C	1.54751	-0.52010	1.23334
C	-0.98074	2.99622	-0.20115
O	-0.38944	3.46617	0.74438
O	-1.77588	3.63421	-1.05963
C	-1.95758	5.04422	-0.79992
N	5.22201	-0.00597	0.85188
O	5.62380	-0.92468	1.57275
O	5.97014	0.77145	0.25122
H	-3.51532	-1.81262	-0.85567
H	-5.33014	-2.53456	0.65905
H	-5.21990	-1.99730	3.08778
H	-3.28380	-0.71259	3.97768
H	-1.48216	0.03880	2.44894
H	-0.88707	-3.00675	0.51003
H	0.31143	-4.79627	-0.69457
H	1.28049	-4.35812	-2.94462
H	1.04576	-2.10132	-3.96812
H	-0.13506	-0.31073	-2.76582
H	1.55456	2.14906	-0.89910
H	3.99822	1.84638	-0.62511
H	3.31537	-1.47553	2.00767
H	0.86354	-1.16585	1.77226
H	-2.62081	5.39379	-1.58948
H	-0.99694	5.56177	-0.84345
H	-2.41357	5.19149	0.18128

Conformer 2
Energy: -1388.80164 Hartree (Rel: 0.0 kcal/mol)
XYZ coordinates for conf 2:

C	1.28395	-0.40711	-0.27212
N	0.35866	0.65852	0.34901
N	0.87417	1.64176	-0.57509
C	1.81178	0.75918	-1.14872
O	2.69136	0.91681	-1.94842
C	-1.04060	0.50049	0.40455
C	2.36525	-0.85503	0.70250
C	0.59221	-1.52576	-1.03204
C	0.45760	-2.80112	-0.46496
C	-0.21624	-3.81417	-1.14875
C	-0.76105	-3.56783	-2.41020
C	-0.63072	-2.30121	-2.98452
C	0.03915	-1.28742	-2.30016
C	3.46238	-1.57426	0.20249
C	4.48505	-1.98063	1.05801
C	4.42377	-1.67702	2.42156
C	3.33770	-0.95748	2.92063
C	2.31449	-0.54018	2.06447

C	-1.93038	1.35905	-0.26163
C	-3.30185	1.19453	-0.11282
C	-3.78420	0.16858	0.70026
C	-2.91468	-0.69322	1.37563
C	-1.54752	-0.52010	1.23332
C	0.98076	2.99621	-0.20117
O	0.38946	3.46617	0.74437
O	1.77590	3.63419	-1.05965
C	1.95763	5.04419	-0.79993
N	-5.22202	-0.00595	0.85188
O	-5.62381	-0.92465	1.57275
O	-5.97015	0.77147	0.25121
H	0.88711	-3.00678	0.51001
H	-0.31137	-4.79631	-0.69459
H	-1.28047	-4.35814	-2.94462
H	-1.04579	-2.10133	-3.96809
H	0.13501	-0.31073	-2.76580
H	3.51537	-1.81256	-0.85566
H	5.33017	-2.53447	0.65908
H	5.21987	-1.99726	3.08782
H	3.28371	-0.71263	3.97771
H	1.48208	0.03874	2.44895
H	-1.55456	2.14909	-0.89908
H	-3.99822	1.84641	-0.62509
H	-3.31538	-1.47553	2.00765
H	-0.86355	-1.16586	1.77223
H	2.62085	5.39376	-1.58950
H	2.41364	5.19146	0.18127
H	0.99700	5.56176	-0.84344

Conformer 3
Energy: -1388.80164 Hartree (Rel: 0.0 kcal/mol)
XYZ coordinates for conf 3:

C	1.28396	-0.40709	0.27213
N	0.35865	0.65849	-0.34863
N	0.87411	1.64171	0.57553
C	1.81203	0.75916	1.14872
O	2.69172	0.91674	1.94830
C	-1.04060	0.50049	-0.40447
C	0.59235	-1.52575	1.03216
C	2.36511	-0.85501	-0.70269
C	3.46215	-1.57460	-0.20297
C	4.48471	-1.98085	-1.05868
C	4.42342	-1.67678	-2.42211
C	3.33744	-0.95689	-2.92090
C	2.31435	-0.53971	-2.06455
C	0.45756	-2.80107	0.46505
C	-0.21620	-3.81413	1.14893
C	-0.76075	-3.56781	2.41050
C	-0.63026	-2.30122	2.98484
C	0.03953	-1.28744	2.30041
C	-1.93040	1.35905	0.26167
C	-3.30185	1.19449	0.11278
C	-3.78414	0.16853	-0.70035
C	-2.91454	-0.69319	-1.37574
C	-1.54739	-0.52007	-1.23334
C	0.98048	2.99617	0.20153
O	0.38819	3.46615	-0.74338
O	1.77647	3.63410	1.05923
C	1.95754	5.04422	0.79974
N	-5.22187	-0.00606	-0.85200
O	-5.62358	-0.92466	-1.57306
O	-5.97007	0.77108	-0.25105
H	3.51512	-1.81328	0.85509
H	5.32973	-2.53500	-0.65997
H	5.21945	-1.99689	-3.08851
H	3.28345	-0.71169	-3.97790
H	1.48200	0.03945	-2.44879
H	0.88689	-3.00671	-0.51000
H	-0.31146	-4.79624	0.69475
H	-1.28007	-4.35814	2.94499

```
H         -1.04514       -2.10136        3.96850
H          0.13548       -0.31076        2.76604
H         -1.55462        2.14917        0.89903
H         -3.99831        1.84630        0.62504
H         -3.31519       -1.47542       -2.00788
H         -0.86334       -1.16574       -1.77226
H          2.62049        5.39400        1.58944
H          0.99668        5.56133        0.84315
H          2.41366        5.19180       -0.18136

Conformer 4
Energy: -1388.80164 Hartree (Rel:  0.0 kcal/mol)
XYZ coordinates for conf 4:
C         -1.28397       -0.40708        0.27213
N         -0.35865        0.65849       -0.34865
N         -0.87410        1.64172        0.57550
C         -1.81203        0.75918        1.14870
O         -2.69173        0.91678        1.94827
C          1.04061        0.50048       -0.40448
C         -2.36513       -0.85500       -0.70269
C         -0.59237       -1.52574        1.03216
C         -0.45756       -2.80106        0.46506
C          0.21618       -3.81411        1.14895
C          0.76070       -3.56780        2.41053
C          0.63021       -2.30120        2.98487
C         -0.03957       -1.28743        2.30042
C         -3.46217       -1.57458       -0.20296
C         -4.48474       -1.98083       -1.05865
C         -4.42345       -1.67677       -2.42210
C         -3.33747       -0.95690       -2.92089
C         -2.31437       -0.53971       -2.06456
C          1.93041        1.35902        0.26167
C          3.30186        1.19445        0.11279
C          3.78413        0.16850       -0.70036
C          2.91454       -0.69320       -1.37577
C          1.54739       -0.52007       -1.23337
C         -0.98043        2.99618        0.20151
O         -0.38815        3.46616       -0.74340
O         -1.77639        3.63413        1.05923
C         -1.95745        5.04425        0.79975
N          5.22187       -0.00611       -0.85199
O          5.62358       -0.92470       -1.57307
O          5.97007        0.77101       -0.25102
H         -0.88687       -3.00670       -0.51000
H          0.31145       -4.79623        0.69478
H          1.28001       -4.35812        2.94504
H          1.04507       -2.10134        3.96854
H         -0.13553       -0.31074        2.76605
H         -3.51514       -1.81325        0.85510
H         -5.32976       -2.53497       -0.65994
H         -5.21949       -1.99688       -3.08849
H         -3.28348       -0.71170       -3.97789
H         -1.48202        0.03943       -2.44880
H          1.55464        2.14914        0.89904
H          3.99832        1.84624        0.62506
H          3.31518       -1.47544       -2.00792
H          0.86333       -1.16572       -1.77230
H         -2.62038        5.39404        1.58946
H         -2.41357        5.19184       -0.18135
H         -0.99657        5.56135        0.84315
```

Revised structure of 106{7a}, (xray is obtained for a different regioisomer) i.e regioisomer 110{7a-rev} ($CDCl_3$)

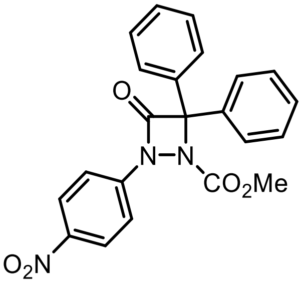

	**Conf1**	**Conf2**	**Conf3**	**Conf4**
Rel energy (kcal/mol):	0.00	0.00	0.83	0.83

**C-nom**	**iGau**	**Exp**	**Calc**	**diff**	**1**	**2**	**3**	**4**
C-C	4	165.30	167.67	2.37	[167.48	167.47	168.45	168.45]
C-C	24	158.60	159.68	1.08	[160.04	160.04	158.22	158.22]
C-C	6	144.10	143.84	-0.26	[143.81	143.80	143.96	143.96]
C-C	21	142.60	143.81	1.21	[143.78	143.77	143.94	143.95]
C-C	8	134.30	134.42	0.12	[134.93	133.95	134.38	134.29]
C-C	7	134.30	134.41	0.11	[133.95	134.92	134.29	134.37]
C-C	1	93.00	95.26	2.26	[95.27	95.28	95.19	95.18]
C-CH	11	129.70	128.94	-0.76	[129.63	128.27	128.17	129.60]
C-CH	16	129.70	128.94	-0.76	[128.27	129.63	129.60	128.17]
C-CH	14	128.80	128.17	-0.63	[127.30	128.95	129.53	127.22]
C-CH	9	128.80	128.17	-0.63	[128.95	127.30	127.22	129.53]
C-CH	13	128.80	128.17	-0.63	[128.95	127.30	127.22	129.53]
C-CH	18	128.80	128.17	-0.63	[127.30	128.95	129.53	127.22]
C-CH	10	128.10	127.85	-0.25	[128.11	127.62	127.61	127.92]
C-CH	12	128.10	127.85	-0.25	[128.11	127.62	127.61	127.92]
C-CH	15	128.10	127.85	-0.25	[127.62	128.11	127.92	127.61]
C-CH	17	128.10	127.85	-0.25	[127.62	128.11	127.92	127.61]
C-CH	20	124.90	125.15	0.25	[125.15	125.15	125.16	125.15]
C-CH	22	124.90	125.15	0.25	[125.15	125.15	125.16	125.15]
C-CH	19	116.20	115.63	-0.57	[115.62	115.62	115.66	115.65]
C-CH	23	116.20	115.63	-0.57	[115.62	115.62	115.66	115.65]
C-CH3	27	53.70	53.71	0.01	[53.64	53.64	54.00	54.00]

13C chem shifts: RMSD=0.88ppm (MAE=0.64) N=22 {-0.76 2.37}

Fractions: 0.402 0.402 0.098 0.098

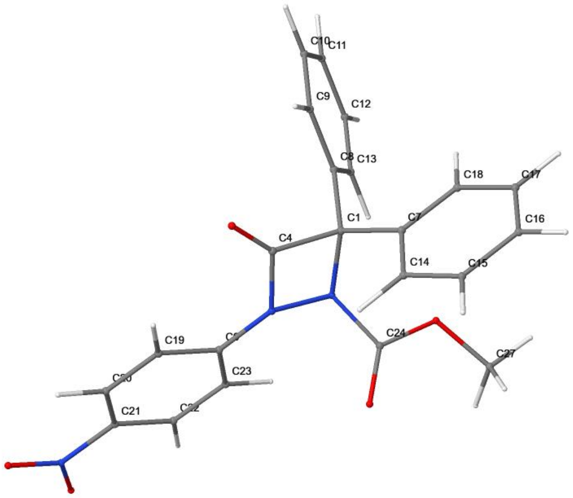

Conformer 1
Energy: -1388.80886 Hartree (Rel: 0.0 kcal/mol)
XYZ coordinates for conf 1:
C 1.44226 0.31676 -0.31724

N	-0.58421	-0.24352	-0.46992
N	0.36965	-0.28110	0.60822
C	0.28804	0.38231	-1.35478
O	0.13030	0.83085	-2.46350
C	-1.97520	-0.17308	-0.33426
C	1.86935	1.70210	0.13505
C	2.57700	-0.62748	-0.69159
C	2.31701	-1.75666	-1.48539
C	3.34261	-2.64136	-1.81755
C	4.64325	-2.40880	-1.36370
C	4.90954	-1.28910	-0.57358
C	3.88348	-0.40405	-0.23855
C	2.31736	2.62386	-0.82208
C	2.73159	3.89657	-0.42889
C	2.70756	4.25785	0.92091
C	2.26424	3.34041	1.87541
C	1.84332	2.06676	1.48558
C	-2.75230	0.03751	-1.48934
C	-4.13263	0.11832	-1.37985
C	-4.72883	-0.01698	-0.12378
C	-3.96707	-0.22842	1.02668
C	-2.58526	-0.30896	0.92289
C	0.52719	-1.53222	1.23365
O	-0.25851	-2.45240	1.14199
O	1.61114	-1.48919	2.01741
C	1.89601	-2.70059	2.74734
N	-6.18063	0.06343	-0.01138
O	-6.68467	-0.06081	1.10844
O	-6.83326	0.25303	-1.04160
H	1.31227	-1.95156	-1.85048
H	3.12509	-3.50700	-2.43646
H	5.44359	-3.09447	-1.62723
H	5.91770	-1.10066	-0.21551
H	4.10044	0.46230	0.37653
H	2.33022	2.34836	-1.87224
H	3.07006	4.60634	-1.17843
H	3.03061	5.24932	1.22576
H	2.24166	3.61413	2.92658
H	1.49403	1.35506	2.22588
H	-2.26997	0.14191	-2.45301
H	-4.74997	0.28221	-2.25396
H	-4.45844	-0.32726	1.98626
H	-1.98455	-0.47456	1.80731
H	2.80356	-2.48778	3.31054
H	2.05852	-3.52979	2.05534
H	1.07017	-2.93868	3.42114

Conformer 2
Energy: -1388.80753 Hartree (Rel: 0.0 kcal/mol)
XYZ coordinates for conf 2:

C	-1.44248	0.31681	-0.31712
N	0.58437	-0.24202	-0.46950
N	-0.36957	-0.28036	0.60853
C	-0.28818	0.38295	-1.35457
O	-0.13063	0.83136	-2.46339
C	1.97533	-0.17193	-0.33401
C	-2.57663	-0.62810	-0.69158
C	-1.87040	1.70193	0.13509
C	-2.31856	2.62351	-0.82214
C	-2.73360	3.89599	-0.42903
C	-2.71023	4.25719	0.92081
C	-2.26676	3.33993	1.87541
C	-1.84504	2.06652	1.48565
C	-2.31579	-1.75733	-1.48506
C	-3.34081	-2.64262	-1.81736
C	-4.64174	-2.41062	-1.36399
C	-4.90886	-1.29089	-0.57422
C	-3.88336	-0.40523	-0.23903
C	2.75236	0.03918	-1.48904
C	4.13273	0.11966	-1.37965
C	4.72903	-0.01636	-0.12372

C	3.96734	-0.22822	1.02672
C	2.58551	-0.30852	0.92301
C	-0.52655	-1.53177	1.23362
O	0.25951	-2.45160	1.14165
O	-1.61042	-1.48934	2.01749
C	-1.89484	-2.70106	2.74707
N	6.18086	0.06376	-0.01143
O	6.68494	-0.06032	1.10839
O	6.83348	0.25290	-1.04173
H	-2.33089	2.34806	-1.87233
H	-3.07217	4.60562	-1.17864
H	-3.03391	5.24847	1.22560
H	-2.24468	3.61359	2.92660
H	-1.49562	1.35498	2.22604
H	-1.31083	-1.95178	-1.84977
H	-3.12266	-3.50828	-2.43602
H	-5.44163	-3.09678	-1.62763
H	-5.91723	-1.10287	-0.21652
H	-4.10098	0.46113	0.37578
H	2.26996	0.14421	-2.45261
H	4.74998	0.28387	-2.25376
H	4.45876	-0.32760	1.98621
H	1.98490	-0.47445	1.80743
H	-2.80250	-2.48877	3.31029
H	-1.06894	-2.93901	3.42085
H	-2.05699	-3.53012	2.05482

Conformer 3
Energy: -1388.80753 Hartree (Rel: 0.8 kcal/mol)
XYZ coordinates for conf 3:

C	-1.54034	0.29509	-0.25823
N	0.48367	-0.27321	-0.45924
N	-0.44371	-0.27487	0.64922
C	-0.41924	0.33475	-1.33093
O	-0.29189	0.75586	-2.45379
C	1.87418	-0.13429	-0.35465
C	-2.68792	-0.65454	-0.57573
C	-1.96017	1.68866	0.17727
C	-2.39721	2.60738	-0.78697
C	-2.81031	3.88308	-0.40143
C	-2.79715	4.24867	0.94722
C	-2.36707	3.33244	1.90928
C	-1.94674	2.05643	1.52739
C	-2.48340	-1.74446	-1.43678
C	-3.52239	-2.63177	-1.71724
C	-4.78033	-2.44194	-1.14081
C	-4.99102	-1.36177	-0.28163
C	-3.95235	-0.47343	-0.00068
C	2.62484	-0.00275	-1.53796
C	4.00249	0.14186	-1.46204
C	4.62193	0.14569	-0.20999
C	3.88639	0.01507	0.96917
C	2.50725	-0.12675	0.89818
C	-0.65856	-1.45638	1.37661
O	-1.59836	-1.57379	2.13571
O	0.32728	-2.34405	1.19556
C	0.25310	-3.52530	2.02312
N	6.07152	0.28972	-0.13209
O	6.59653	0.28465	0.98487
O	6.70049	0.40981	-1.18695
H	-2.40241	2.32797	-1.83612
H	-3.13983	4.59154	-1.15620
H	-3.11956	5.24225	1.24585
H	-2.35550	3.60876	2.95993
H	-1.61313	1.34231	2.27299
H	-1.51364	-1.90450	-1.90004
H	-3.34909	-3.46568	-2.39138
H	-5.59135	-3.12986	-1.36261
H	-5.96552	-1.20755	0.17286
H	-4.12563	0.36200	0.66860
H	2.12478	-0.00743	-2.49829

```
H          4.59996        0.24708       -2.35870
H          4.39517        0.02890        1.92470
H          1.92360       -0.22007        1.80477
H          1.13620       -4.10908        1.76726
H         -0.65779       -4.08418        1.79939
H          0.26507       -3.24827        3.07949

Conformer 4
Energy: -1388.80886 Hartree (Rel:  0.8 kcal/mol)
XYZ coordinates for conf 4:
C         -1.54036       -0.29509       -0.25827
N          0.48365        0.27330       -0.45922
N         -0.44376        0.27482        0.64925
C         -0.41926       -0.33461       -1.33097
O         -0.29191       -0.75561       -2.45387
C          1.87415        0.13437       -0.35463
C         -1.96010       -1.68875        0.17711
C         -2.68793        0.65456       -0.57571
C         -2.48304        1.74518       -1.43580
C         -3.52199        2.63254       -1.71622
C         -4.78025        2.44208       -1.14073
C         -4.99131        1.36121       -0.28251
C         -3.95268        0.47282       -0.00160
C         -2.39667       -2.60761       -0.78719
C         -2.80970       -3.88333       -0.40167
C         -2.79694       -4.24880        0.94702
C         -2.36732       -3.33243        1.90914
C         -1.94706       -2.05638        1.52727
C          2.62482        0.00287       -1.53794
C          4.00247       -0.14171       -1.46203
C          4.62191       -0.14568       -0.20998
C          3.88636       -0.01519        0.96918
C          2.50723        0.12669        0.89820
C         -0.65869        1.45627        1.37671
O         -1.59854        1.57360        2.13577
O          0.32710        2.34401        1.19577
C          0.25289        3.52518        2.02342
N          6.07152       -0.28969       -0.13209
O          6.59636       -0.28631        0.98496
O          6.70066       -0.40810       -1.18704
H         -1.51301        1.90572       -1.89834
H         -3.34841        3.46700       -2.38961
H         -5.59125        3.13005       -1.36248
H         -5.96607        1.20651        0.17125
H         -4.12625       -0.36316        0.66692
H         -2.40154       -2.32830       -1.83637
H         -3.13886       -4.59192       -1.15648
H         -3.11929       -5.24242        1.24561
H         -2.35605       -3.60865        2.95982
H         -1.61382       -1.34214        2.27292
H          2.12478        0.00757       -2.49828
H          4.59994       -0.24681       -2.35871
H          4.39513       -0.02914        1.92471
H          1.92357        0.21996        1.80479
H          1.13645        4.10859        1.76825
H          0.26396        3.24803        3.07977
H         -0.65758        4.08450        1.79908
```

Originally assigned (incorrect) structure of 107{7c} ($CDCl_3$)

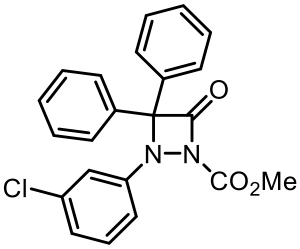

	Conf1	Conf2
Rel energy (kcal/mol):	0.00	0.07

C-nom	iGau	Exp	Calc	diff	1	2
C	4	165.40	166.66	1.26	166.66	166.66
C	25	158.90	150.83	-8.07	150.80	150.86
C	6	138.90	145.59	6.69	145.56	145.62
C	13	134.74	136.52	1.78	136.48	136.57
C	8	134.66	134.34	-0.32	134.55	134.10
C	12	134.66	133.88	-0.78	133.89	133.86
C	19	130.00	130.01	0.01	130.03	129.98
C	23	129.40	130.01	0.61	130.03	129.98
C	21	129.40	129.12	-0.28	129.12	129.13
C	10	128.70	128.87	0.17	128.80	128.94
C	16	128.70	128.11	-0.59	128.11	128.12
C	15	128.70	127.80	-0.90	127.78	127.83
C	17	128.70	127.80	-0.90	127.78	127.83
C	20	128.10	127.61	-0.49	127.60	127.63
C	22	128.10	127.61	-0.49	127.60	127.63
C	14	128.10	127.36	-0.74	127.37	127.34
C	18	128.10	127.36	-0.74	127.37	127.34
C	9	125.00	123.33	-1.67	123.29	123.37
C	7	116.50	119.57	3.07	118.07	121.24
C	11	114.70	118.82	4.12	120.36	117.10
C	1	92.40	97.34	4.94	97.30	97.38
C	28	53.40	54.22	0.82	54.30	54.14

13C chem shifts: RMSD=2.81ppm (MAE=1.79) N=22 {-8.07 6.69}

Fractions: 0.528 0.472

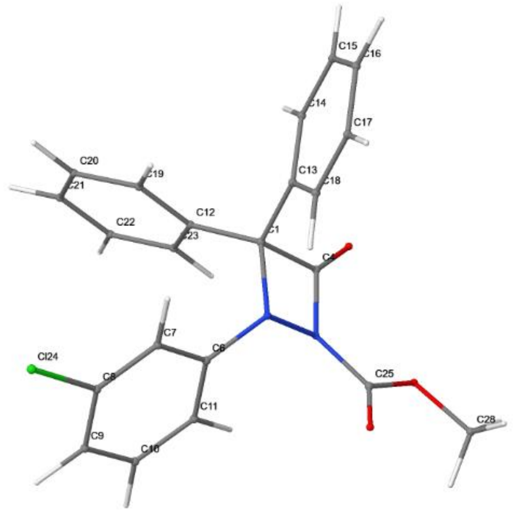

Conformer 1
Energy: -1643.89154 Hartree (Rel: 0.0 kcal/mol)
XYZ coordinates for conf 1:

C	1.12712	-0.14586	0.28639
N	0.04854	0.56752	-0.56210

N	-0.06994	1.57389	0.47030
C	0.96183	1.04594	1.26363
O	1.50889	1.44144	2.25693
C	-1.16081	-0.10411	-0.91036
C	-2.38605	0.17391	-0.29529
C	-3.52828	-0.48755	-0.74335
C	-3.48801	-1.42629	-1.77062
C	-2.25360	-1.69605	-2.36829
C	-1.09905	-1.03649	-1.95651
C	0.74695	-1.50609	0.84131
C	2.49667	-0.09029	-0.37954
C	3.62176	-0.46630	0.37145
C	4.89617	-0.40280	-0.18964
C	5.06333	0.03327	-1.50765
C	3.94822	0.41302	-2.25516
C	2.66944	0.35988	-1.69274
C	1.12465	-2.67571	0.16483
C	0.73933	-3.92832	0.64274
C	-0.02432	-4.03086	1.80765
C	-0.40209	-2.87310	2.49040
C	-0.02132	-1.61950	2.00974
Cl	-5.07159	-0.12362	0.03051
C	-0.44767	2.89228	0.16229
O	-0.95982	3.20418	-0.88918
O	-0.18780	3.68647	1.20311
C	-0.54684	5.07343	1.02197
H	-2.45314	0.88698	0.51622
H	-4.39279	-1.92446	-2.09997
H	-2.19876	-2.41911	-3.17670
H	-0.15065	-1.22916	-2.44684
H	3.49767	-0.80724	1.39506
H	5.75879	-0.69479	0.40279
H	6.05664	0.07847	-1.94550
H	4.06837	0.75726	-3.27871
H	1.80463	0.67517	-2.26561
H	1.72902	-2.60656	-0.73398
H	1.04014	-4.82365	0.10600
H	-0.31889	-5.00668	2.18348
H	-0.98984	-2.94238	3.40123
H	-0.31598	-0.73140	2.56064
H	-0.27728	5.56046	1.95790
H	0.01276	5.50329	0.18839
H	-1.61870	5.16424	0.83317

Conformer 2
Energy: -1643.89164 Hartree (Rel: 0.1 kcal/mol)
XYZ coordinates for conf 2:

C	-0.79899	0.62183	0.34630
N	-0.35516	-0.66355	-0.39140
N	-1.31365	-1.41686	0.38687
C	-1.83924	-0.29201	1.04292
O	-2.77234	-0.14358	1.78462
C	0.99471	-1.11870	-0.30677
C	1.93048	-0.45728	-1.11590
C	3.25488	-0.88000	-1.10086
C	3.67764	-1.96044	-0.32616
C	2.72945	-2.61809	0.45729
C	1.39687	-2.20357	0.47958
C	0.23350	1.25775	1.25799
C	-1.50307	1.59527	-0.59169
C	-2.16978	2.69806	-0.03453
C	-2.85256	3.59553	-0.85387
C	-2.87544	3.40578	-2.23905
C	-2.21842	2.30817	-2.79584
C	-1.54101	1.40039	-1.97648
C	1.05303	2.29170	0.78034
C	2.03870	2.84984	1.59424
C	2.21461	2.38832	2.90077
C	1.40024	1.36418	3.38753
C	0.41837	0.80054	2.57123
Cl	4.42668	-0.03450	-2.11172

C	-1.90866	-2.58309	-0.12309
O	-1.47684	-3.17924	-1.08413
O	-2.95861	-2.91691	0.63086
C	-3.65571	-4.11246	0.21889
H	1.62033	0.36303	-1.75253
H	4.71389	-2.27862	-0.34117
H	3.03517	-3.46339	1.06688
H	0.67914	-2.72037	1.10536
H	-2.15344	2.85183	1.04042
H	-3.36591	4.44401	-0.41014
H	-3.40401	4.10840	-2.87730
H	-2.23340	2.15022	-3.87073
H	-1.04774	0.53566	-2.40585
H	0.91434	2.66666	-0.22867
H	2.66599	3.64791	1.20745
H	2.97788	2.82774	3.53683
H	1.52341	1.00421	4.40498
H	-0.21272	0.01274	2.97125
H	-4.46207	-4.23321	0.94073
H	-4.05651	-3.98859	-0.78949
H	-2.98133	-4.97119	0.24514

Revised structure of 107{7c}, i.e regioisomer 111{7c-rev} ($CDCl_3$)

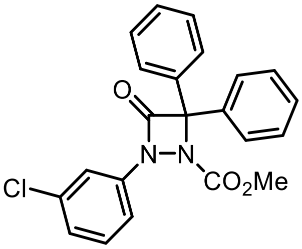

					Conf1	Conf2	Conf3	Conf4	
	Rel energy (kcal/mol):				0.00	0.08	0.66	0.79	
**C-nom**	**iGau**	**Exp**	**Calc**	**diff**	**1**	**2**	**3**	**4**	
C	4	165.40	167.42	2.02	[167.19	167.13	168.32	168.12	]
C	25	158.90	159.78	0.88	[160.15	160.26	158.42	158.51	]
C	6	138.90	138.31	-0.59	[138.28	138.28	138.44	138.37	]
C	7	134.74	135.08	0.34	[135.24	135.22	134.64	134.54	]
C	22	134.66	134.46	-0.20	[134.52	134.40	134.47	134.46	]
C	8	134.66	134.47	-0.19	[134.37	134.33	134.81	134.91	]
C	20	130.00	129.32	-0.68	[129.41	129.21	129.46	129.19	]
C	16	129.40	129.36	-0.04	[129.37	129.37	129.30	129.32	]
C	11	129.40	128.00	-1.40	[128.02	128.03	127.92	127.91	]
C	18	128.70	129.13	0.43	[128.99	128.97	129.53	129.65	]
C	14	128.70	129.13	0.43	[128.99	128.97	129.53	129.65	]
C	15	128.70	127.92	-0.78	[127.97	127.98	127.76	127.74	]
C	17	128.70	127.92	-0.78	[127.97	127.98	127.76	127.74	]
C	10	128.10	127.48	-0.62	[127.50	127.47	127.49	127.47	]
C	12	128.10	127.48	-0.62	[127.50	127.47	127.49	127.47	]
C	9	128.10	127.28	-0.82	[127.30	127.32	127.20	127.15	]
C	13	128.10	127.28	-0.82	[127.30	127.32	127.20	127.15	]
C	21	125.00	123.24	-1.76	[123.18	123.20	123.39	123.44	]
C	19	116.50	114.54	-1.96	[115.51	113.26	116.08	113.18	]
C	23	114.70	115.22	0.52	[114.30	116.17	114.23	116.78	]
C	1	92.40	94.58	2.18	[94.59	94.64	94.35	94.60	]
C	28	53.40	53.49	0.09	[53.44	53.38	53.79	53.68	]
	**13C chem shifts: RMSD=1.03ppm (MAE=0.82) N=22 {-1.96 2.18}**								
	Fractions:				0.405	0.354	0.133	0.108	

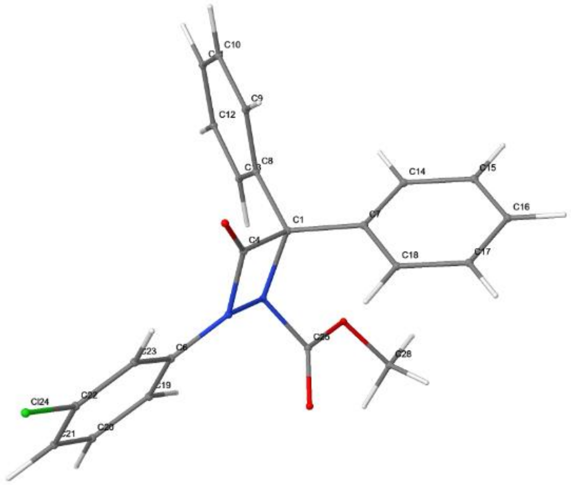

Conformer 1
Energy: -1643.89810 Hartree (Rel: 0.0 kcal/mol)
XYZ coordinates for conf 1:

C	1.17943	0.30826	0.34172
N	-0.04381	-0.25873	-0.39480
N	-0.80650	-0.19983	0.82634

C	0.20723	0.40568	1.55113
O	0.24544	0.86365	2.66858
C	-2.20794	-0.07477	0.90892
C	2.33398	-0.66696	0.52955
C	1.56669	1.68145	-0.17772
C	2.19632	2.58555	0.69007
C	2.57590	3.84763	0.23290
C	2.33556	4.21644	-1.09355
C	1.71067	3.31707	-1.95950
C	1.32420	2.05413	-1.50431
C	3.55145	-0.48576	-0.13929
C	4.59377	-1.39918	0.02701
C	4.43286	-2.50608	0.86241
C	3.22122	-2.69686	1.53116
C	2.17992	-1.78360	1.36741
C	-2.79471	0.11962	2.16861
C	-4.17787	0.25589	2.25783
C	-4.98733	0.19365	1.12097
C	-4.37424	-0.00401	-0.11454
C	-2.99482	-0.14100	-0.24622
Cl	-5.36964	-0.08437	-1.56685
C	-0.02335	-1.51193	-1.03290
O	-0.80305	-2.41449	-0.80918
O	0.91441	-1.49517	-1.98976
C	1.04065	-2.71271	-2.75183
H	2.37612	2.30486	1.72342
H	3.05631	4.54362	0.91487
H	2.63157	5.19971	-1.44846
H	1.51886	3.59679	-2.99171
H	0.83332	1.35679	-2.17459
H	3.68612	0.37042	-0.79114
H	5.53198	-1.24301	-0.49800
H	5.24624	-3.21404	0.99452
H	3.08644	-3.55238	2.18671
H	1.24567	-1.94569	1.89792
H	-2.17150	0.16969	3.05279
H	-4.63506	0.40961	3.23067
H	-6.06444	0.29264	1.19255
H	-2.54639	-0.29157	-1.21880
H	1.84055	-2.52154	-3.46599
H	1.30332	-3.54502	-2.09505
H	0.10505	-2.93380	-3.27020

Conformer 2
Energy: -1643.89790 Hartree (Rel: 0.1 kcal/mol)
XYZ coordinates for conf 2:

C	-1.09018	0.32482	-0.28197
N	-0.21164	-0.32966	0.79558
N	0.91544	-0.29349	-0.10197
C	0.23209	0.38621	-1.09678
O	0.59160	0.86718	-2.14548
C	2.26954	-0.25443	0.28752
C	-2.16371	-0.57550	-0.87919
C	-1.55856	1.70823	0.13274
C	-1.81219	2.66733	-0.85846
C	-2.25860	3.94060	-0.50408
C	-2.46059	4.26594	0.83996
C	-2.21078	3.31170	1.82808
C	-1.75869	2.03719	1.47789
C	-3.52412	-0.33017	-0.65241
C	-4.49561	-1.17566	-1.19084
C	-4.12059	-2.27789	-1.96118
C	-2.76615	-2.53247	-2.19003
C	-1.79479	-1.68700	-1.65459
C	2.64196	-0.42225	1.62600
C	3.99230	-0.36962	1.96743
C	4.97620	-0.15558	1.00082
C	4.57416	0.00721	-0.32448
C	3.23711	-0.04097	-0.70627
Cl	5.79451	0.27975	-1.56605
C	-0.51021	-1.59053	1.34203

O	0.24836	-2.53762	1.35456
O	-1.71061	-1.53096	1.93494
C	-2.15129	-2.75278	2.56084
H	-1.64753	2.42002	-1.90279
H	-2.44500	4.67906	-1.27878
H	-2.80833	5.25794	1.11458
H	-2.36411	3.55730	2.87533
H	-1.55907	1.29664	2.24489
H	-3.82575	0.52297	-0.05469
H	-5.54640	-0.97013	-1.00683
H	-4.87767	-2.93271	-2.38340
H	-2.46317	-3.38469	-2.79148
H	-0.74623	-1.89804	-1.84581
H	1.88978	-0.58999	2.38586
H	4.28332	-0.49871	3.00558
H	6.02636	-0.11847	1.26703
H	2.94523	0.09227	-1.73970
H	-3.13334	-2.52500	2.97325
H	-2.22192	-3.55346	1.82123
H	-1.45814	-3.04392	3.35322

Conformer 3
Energy: -1643.89915 Hartree (Rel: 0.7 kcal/mol)
XYZ coordinates for conf 3:

C	-1.27638	0.26115	-0.28502
N	-0.03034	-0.23431	0.45339
N	0.70008	-0.27543	-0.79449
C	-0.34316	0.27766	-1.52636
O	-0.41485	0.65491	-2.67143
C	2.09338	-0.08882	-0.92492
C	-2.42624	-0.73449	-0.36790
C	-1.67104	1.66149	0.15102
C	-2.30215	2.51335	-0.76609
C	-2.69297	3.79495	-0.37702
C	-2.46367	4.23359	0.92987
C	-1.83955	3.38435	1.84589
C	-1.44123	2.10269	1.45898
C	-3.57752	-0.57068	0.41311
C	-4.61548	-1.50087	0.34834
C	-4.51736	-2.60696	-0.49815
C	-3.37251	-2.77997	-1.27909
C	-2.33423	-1.85054	-1.21444
C	2.64765	-0.04790	-2.21284
C	4.01945	0.14814	-2.35442
C	4.84968	0.29053	-1.23996
C	4.26896	0.24111	0.02577
C	2.90099	0.05526	0.20830
Cl	5.29091	0.42108	1.45038
C	-0.08438	-1.37168	1.27115
O	-0.88873	-1.46956	2.17604
O	0.89597	-2.23996	0.98994
C	0.99976	-3.36415	1.88911
H	-2.47417	2.17738	-1.78415
H	-3.17408	4.45127	-1.09681
H	-2.76890	5.23175	1.23147
H	-1.65875	3.71752	2.86412
H	-0.95673	1.44071	2.16905
H	-3.66256	0.28453	1.07452
H	-5.50105	-1.35924	0.96139
H	-5.32838	-3.32788	-0.55143
H	-3.28788	-3.63398	-1.94507
H	-1.45405	-1.99807	-1.83398
H	2.00851	-0.15900	-3.08005
H	4.45153	0.18494	-3.34989
H	5.91850	0.43339	-1.35092
H	2.47374	0.03320	1.20185
H	1.85360	-3.93815	1.53162
H	1.16815	-3.01749	2.91113
H	0.08782	-3.96355	1.85007

Conformer 4

Energy: -1643.89902 Hartree (Rel: 0.8 kcal/mol)
XYZ coordinates for conf 4:

C	-1.21271	0.28888	-0.23411
N	-0.26932	-0.30085	0.81875
N	0.82700	-0.23994	-0.12234
C	0.07299	0.39716	-1.09880
O	0.37461	0.87783	-2.16510
C	2.18403	-0.07276	0.22852
C	-2.26817	-0.66575	-0.77634
C	-1.73630	1.65362	0.17874
C	-2.04716	2.59598	-0.81150
C	-2.55125	3.84684	-0.45429
C	-2.75524	4.16457	0.89124
C	-2.45022	3.22537	1.87845
C	-1.93953	1.97400	1.52560
C	-3.60725	-0.55779	-0.37851
C	-4.56354	-1.45136	-0.86171
C	-4.19510	-2.46432	-1.74946
C	-2.86246	-2.58101	-2.15032
C	-1.90550	-1.68825	-1.66708
C	2.58904	-0.05504	1.56773
C	3.93936	0.11811	1.86733
C	4.89045	0.27387	0.85749
C	4.45609	0.25353	-0.46753
C	3.11811	0.07712	-0.80710
Cl	5.63501	0.44842	-1.76216
C	-0.57274	-1.51140	1.45758
O	-1.62193	-1.67707	2.04686
O	0.45086	-2.37431	1.41371
C	0.26822	-3.58534	2.17680
H	-1.88197	2.35397	-1.85696
H	-2.78170	4.57377	-1.22809
H	-3.14848	5.13890	1.16782
H	-2.60695	3.46445	2.92668
H	-1.70179	1.24237	2.29064
H	-3.90273	0.22539	0.31101
H	-5.59747	-1.35422	-0.54281
H	-4.94167	-3.15630	-2.12910
H	-2.56601	-3.36167	-2.84508
H	-0.87512	-1.78945	-1.99612
H	1.85840	-0.16459	2.35926
H	4.25615	0.13467	2.90586
H	5.94060	0.40681	1.09115
H	2.80108	0.06554	-1.84175
H	1.19168	-4.14790	2.04576
H	0.10664	-3.34848	3.23086
H	-0.58464	-4.14863	1.79246

Originally assigned (incorrect) structure of 108{7e} ($CDCl_3$)

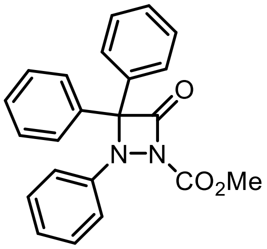

					Conf1
		Rel energy (kcal/mol):			0.00
**C-nom**	**iGau**	**Exp**	**Calc**	**diff**	**1**
C-C	4	165.30	166.93	1.63	[166.93]
C-C	25	159.00	150.82	-8.18	[150.82]
C-C	6	138.00	143.84	5.84	[143.84]
C-C	8	134.90	137.39	2.49	[137.39]
C-C	7	134.90	134.54	-0.36	[134.54]
C-C	1	92.00	96.81	4.81	[96.81]
C-CH	14	129.20	130.06	0.86	[130.06]
C-CH	18	129.20	130.06	0.86	[130.06]
C-CH	16	128.80	128.70	-0.10	[128.70]
C-CH	11	128.80	127.84	-0.96	[127.84]
C-CH	20	128.60	127.79	-0.81	[127.79]
C-CH	22	128.60	127.79	-0.81	[127.79]
C-CH	10	128.60	127.68	-0.92	[127.68]
C-CH	12	128.60	127.68	-0.92	[127.68]
C-CH	9	128.10	127.46	-0.64	[127.46]
C-CH	13	128.10	127.46	-0.64	[127.46]
C-CH	15	128.10	127.39	-0.71	[127.39]
C-CH	17	128.10	127.39	-0.71	[127.39]
C-CH	21	124.90	123.88	-1.02	[123.88]
C-CH	19	116.50	120.06	3.56	[120.06]
C-CH	23	116.50	120.06	3.56	[120.06]
C-CH3	28	53.30	54.09	0.79	[54.09]

13C chem shifts: RMSD=2.76ppm (MAE=1.87) N=22 {-8.18 5.84}

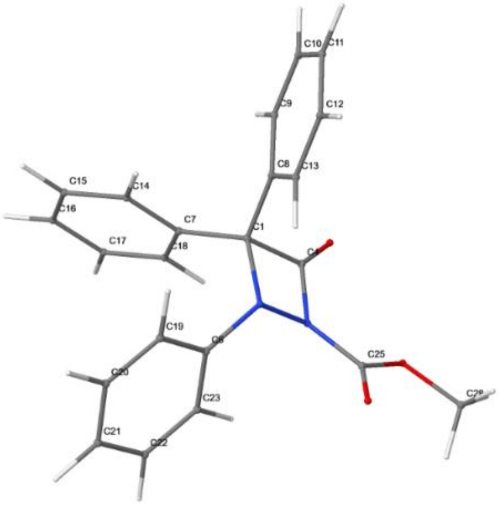

Conformer 1
Energy: -1184.29888 Hartree (Rel: 0.0 kcal/mol)
XYZ coordinates for conf 1:

C	0.63333	-0.32601	-0.27898
N	-0.65298	-0.18897	0.56949
N	-1.37867	-0.89861	-0.46297
C	-0.24764	-1.14772	-1.25317

O	-0.07302	-1.80619	-2.24349
C	-1.16812	1.10607	0.89721
C	1.20677	0.96210	-0.83924
C	1.65950	-1.23208	0.39184
C	2.77957	-1.63982	-0.35000
C	3.72579	-2.49384	0.21427
C	3.56830	-2.94854	1.52715
C	2.45408	-2.54953	2.26598
C	1.49786	-1.70123	1.69984
C	2.18727	1.66448	-0.12205
C	2.69176	2.87186	-0.60435
C	2.22942	3.39219	-1.81556
C	1.25829	2.69846	-2.53940
C	0.74826	1.49315	-2.05360
C	-0.57435	1.76809	1.98138
C	-1.04347	3.01882	2.37795
C	-2.12379	3.61106	1.71709
C	-2.72384	2.94172	0.64970
C	-2.24740	1.69689	0.23175
H	-2.71381	1.19116	-0.60637
C	-2.51872	-1.66141	-0.16631
O	-3.11998	-1.57641	0.88110
O	-2.82657	-2.43302	-1.21294
C	-3.99157	-3.26705	-1.03789
H	2.90818	-1.28804	-1.36941
H	4.58686	-2.80342	-0.37155
H	4.30852	-3.61070	1.96778
H	2.32094	-2.90046	3.28571
H	0.62080	-1.40669	2.26490
H	2.56262	1.26050	0.81287
H	3.44963	3.40299	-0.03532
H	2.62775	4.32908	-2.19490
H	0.89813	3.09025	-3.48642
H	0.00280	0.96160	-2.63716
H	0.24404	1.29292	2.51363
H	-0.57266	3.52431	3.21667
H	-2.49743	4.57964	2.03650
H	-3.56667	3.38926	0.12988
H	-4.09352	-3.81233	-1.97505
H	-4.87376	-2.65056	-0.85157
H	-3.84199	-3.95748	-0.20487

Revised structure of 108{7e}, i.e regioisomer 112{7e-rev} ($CDCl_3$)

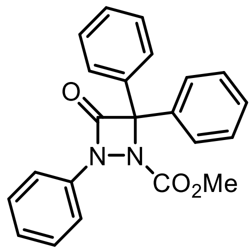

					Conf1	Conf2	Conf3	Conf4
			Rel energy (kcal/mol):		0.00	0.00	0.64	0.64
**C-nom**	**iGau**	**Exp**	**Calc**	**diff**	**1**	**2**	**3**	**4**
C-C	4	165.30	167.16	1.86	[166.89	166.87	168.01	168.00]
C-C	25	159.00	159.90	0.90	[160.29	160.28	158.76	158.76]
C-C	6	138.00	137.21	-0.79	[137.20	137.19	137.27	137.26]
C-C	7	134.90	135.16	0.26	[134.66	135.68	134.89	135.40]
C-C	8	134.90	135.16	0.26	[135.68	134.66	135.40	134.90]
C-C	1	92.00	93.95	1.95	[93.94	93.94	93.99	93.99]
C-CH	16	129.20	128.49	-0.71	[127.86	129.15	129.14	127.72]
C-CH	14	129.20	128.23	-0.97	[127.41	128.88	129.76	127.19]
C-CH	18	128.80	128.23	-0.57	[127.41	128.88	129.76	127.19]
C-CH	20	128.80	128.03	-0.77	[128.02	128.02	128.06	128.06]
C-CH	22	128.60	128.03	-0.57	[128.02	128.02	128.06	128.06]
C-CH	15	128.60	127.62	-0.98	[127.39	127.93	127.63	127.41]
C-CH	17	128.60	127.62	-0.98	[127.39	127.93	127.63	127.41]
C-CH	11	128.60	128.49	-0.11	[129.15	127.86	127.72	129.14]
C-CH	9	128.10	128.23	0.13	[128.88	127.41	127.19	129.76]
C-CH	13	128.10	128.23	0.13	[128.88	127.41	127.19	129.76]
C-CH	10	128.10	127.62	-0.48	[127.93	127.39	127.41	127.63]
C-CH	12	128.10	127.62	-0.48	[127.93	127.39	127.41	127.63]
C-CH	21	124.90	123.52	-1.38	[123.46	123.46	123.70	123.70]
C-CH	19	116.50	115.37	-1.13	[115.30	115.30	115.56	115.56]
C-CH	23	116.50	115.37	-1.13	[115.30	115.30	115.56	115.56]
C-CH3	28	53.30	53.33	0.03	[53.25	53.24	53.58	53.58]

13C chem shifts: RMSD=0.92ppm (MAE=0.75) N=22 {-1.38 1.95}

Fractions: 0.374 0.374 0.126 0.126

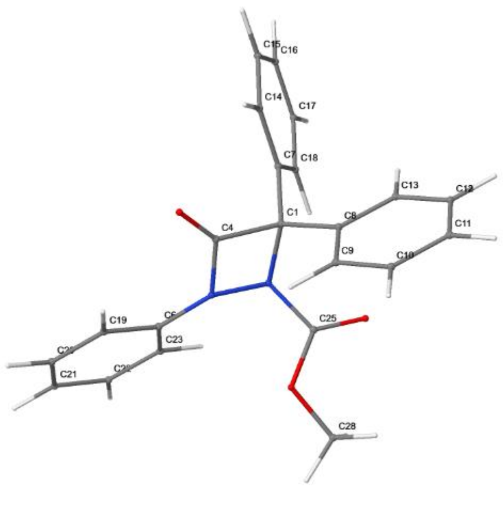


```
Conformer 1
Energy: -1184.30597 Hartree (Rel:  0.0 kcal/mol)
XYZ coordinates for conf 1:
C         -0.67528        0.30440       -0.32266
N          1.36037       -0.21349       -0.47157
N          0.40880       -0.25225        0.61067
C          0.48305        0.38377       -1.35766
```

O	0.62675	0.82804	-2.47339
C	2.76171	-0.10268	-0.32680
C	-1.14193	1.68316	0.10794
C	-1.78406	-0.67561	-0.68507
C	-1.48684	-1.81945	-1.44422
C	-2.48650	-2.73658	-1.76736
C	-3.79934	-2.52266	-1.33995
C	-4.10305	-1.38875	-0.58452
C	-3.10241	-0.47167	-0.25796
C	-1.60505	2.58117	-0.86451
C	-2.05511	3.84804	-0.49203
C	-2.05190	4.22786	0.85289
C	-1.59306	3.33459	1.82297
C	-1.13695	2.06697	1.45350
C	3.35921	-0.14704	0.93772
C	4.74503	-0.02563	1.04230
C	5.53496	0.13740	-0.09761
C	4.92623	0.17919	-1.35477
C	3.54349	0.05567	-1.48107
H	3.06792	0.08891	-2.45405
C	0.27629	-1.49132	1.25968
O	1.07233	-2.40429	1.18438
O	-0.80546	-1.45051	2.05191
C	-1.06608	-2.65283	2.80252
H	-0.47171	-1.99975	-1.78711
H	-2.23929	-3.61342	-2.35892
H	-4.57957	-3.23368	-1.59682
H	-5.12073	-1.21423	-0.24655
H	-3.34894	0.40518	0.33057
H	-1.59955	2.29143	-1.91084
H	-2.40485	4.53909	-1.25391
H	-2.40272	5.21474	1.14179
H	-1.58585	3.62289	2.87055
H	-0.77540	1.37393	2.20551
H	2.75039	-0.27148	1.82486
H	5.20589	-0.05993	2.02552
H	6.61345	0.22815	-0.00810
H	5.52940	0.30544	-2.24948
H	-0.23324	-2.86752	3.47587
H	-1.21890	-3.49703	2.12634
H	-1.97400	-2.44649	3.36795

Conformer 2
Energy: -1184.30700 Hartree (Rel: 0.0 kcal/mol)
XYZ coordinates for conf 2:

C	0.67556	0.30443	-0.32245
N	-1.36033	-0.21231	-0.47141
N	-0.40876	-0.25170	0.61081
C	-0.48269	0.38435	-1.35755
O	-0.62614	0.82857	-2.47332
C	-2.76164	-0.10179	-0.32687
C	1.78386	-0.67611	-0.68487
C	1.14277	1.68302	0.10813
C	1.60634	2.58079	-0.86436
C	2.05671	3.84756	-0.49195
C	2.05335	4.22756	0.85293
C	1.59410	3.33454	1.82302
C	1.13769	2.06700	1.45363
C	1.48581	-1.82033	-1.44318
C	2.48497	-2.73794	-1.76641
C	3.79818	-2.52414	-1.33997
C	4.10271	-1.38989	-0.58542
C	3.10252	-0.47232	-0.25873
C	-3.35930	-0.14650	0.93760
C	-4.74512	-0.02528	1.04205
C	-5.53495	0.13796	-0.09794
C	-4.92608	0.18018	-1.35500
C	-3.54330	0.05685	-1.48117
H	-3.06762	0.09045	-2.45410
C	-0.27694	-1.49095	1.25965
O	-1.07350	-2.40343	1.18421

O	0.80483	-1.45084	2.05191
C	1.06484	-2.65346	2.80222
H	1.60097	2.29093	-1.91065
H	2.40680	4.53842	-1.25386
H	2.40439	5.21438	1.14178
H	1.58681	3.62296	2.87058
H	0.77587	1.37413	2.20567
H	0.47039	-2.00052	-1.78528
H	2.23715	-3.61508	-2.35727
H	4.57803	-3.23556	-1.59693
H	5.12064	-1.21545	-0.24820
H	3.34970	0.40476	0.32918
H	-2.75055	-0.27108	1.82478
H	-5.20612	-0.05987	2.02519
H	-6.61346	0.22854	-0.00850
H	-5.52914	0.30664	-2.24977
H	1.21625	-3.49780	2.12589
H	0.23234	-2.86734	3.47626
H	1.97342	-2.44807	3.36694

Conformer 3
Energy: -1184.30700 Hartree (Rel: 0.6 kcal/mol)
XYZ coordinates for conf 3:

C	-0.81139	-0.25376	-0.26388
N	1.24915	0.12856	-0.50313
N	0.34856	0.19471	0.62726
C	0.28636	-0.38849	-1.35508
O	0.34827	-0.81471	-2.48479
C	2.63586	-0.14317	-0.42274
C	-1.86682	0.80704	-0.54836
C	-1.36316	-1.60314	0.16188
C	-1.92841	-2.44753	-0.80396
C	-2.46175	-3.68132	-0.43018
C	-2.44176	-4.07925	0.90938
C	-1.88310	-3.23753	1.87327
C	-1.34236	-2.00410	1.50235
C	-1.59854	1.84903	-1.44972
C	-2.55218	2.83707	-1.69710
C	-3.78712	2.79757	-1.04646
C	-4.06102	1.76558	-0.14636
C	-3.10811	0.77717	0.10096
C	3.26780	-0.30681	0.81472
C	4.63504	-0.58192	0.85201
C	5.37153	-0.69634	-0.32905
C	4.72835	-0.53272	-1.55878
C	3.36433	-0.24989	-1.61641
H	2.86233	-0.12166	-2.56818
C	0.26062	1.36833	1.38660
O	-0.65010	1.55729	2.16891
O	1.32453	2.16372	1.21181
C	1.37781	3.31812	2.07502
H	-1.93755	-2.14363	-1.84628
H	-2.89048	-4.33249	-1.18686
H	-2.85827	-5.04001	1.19915
H	-1.86469	-3.53917	2.91694
H	-0.90787	-1.34797	2.24922
H	-0.64673	1.89109	-1.97146
H	-2.33075	3.63200	-2.40361
H	-4.53194	3.56394	-1.24225
H	-5.01818	1.72780	0.36612
H	-3.32956	-0.02002	0.80214
H	2.69589	-0.22952	1.73171
H	5.12311	-0.70994	1.81418
H	6.43581	-0.90923	-0.29200
H	5.29007	-0.62008	-2.48455
H	1.38181	3.00875	3.12266
H	0.52120	3.96935	1.88856
H	2.30816	3.82380	1.81950

Conformer 4
Energy: -1184.30597 Hartree (Rel: 0.6 kcal/mol)

XYZ coordinates for conf 4:

C	-0.81144	0.25379	-0.26390
N	1.24914	-0.12827	-0.50304
N	0.34852	-0.19451	0.62729
C	0.28635	0.38864	-1.35506
O	0.34829	0.81486	-2.48477
C	2.63584	0.14342	-0.42265
C	-1.36342	1.60310	0.16178
C	-1.86665	-0.80722	-0.54840
C	-1.59801	-1.84937	-1.44948
C	-2.55141	-2.83765	-1.69677
C	-3.78647	-2.79824	-1.04636
C	-4.06075	-1.76606	-0.14658
C	-3.10807	-0.77743	0.10068
C	-1.92870	2.44741	-0.80412
C	-2.46222	3.68114	-0.43040
C	-2.44239	4.07909	0.90915
C	-1.88372	3.23744	1.87311
C	-1.34279	2.00408	1.50225
C	3.36436	0.24989	-1.61631
C	4.72839	0.53272	-1.55868
C	5.37153	0.69654	-0.32897
C	4.63498	0.58234	0.85211
C	3.26775	0.30727	0.81481
H	2.69578	0.23019	1.73180
C	0.26068	-1.36812	1.38664
O	-0.65005	-1.55716	2.16893
O	1.32464	-2.16343	1.21186
C	1.37796	-3.31785	2.07503
H	-0.64612	-1.89135	-1.97107
H	-2.32970	-3.63271	-2.40306
H	-4.53110	-3.56482	-1.24207
H	-5.01801	-1.72835	0.36572
H	-3.32979	0.01990	0.80163
H	-1.93773	2.14349	-1.84644
H	-2.89096	4.33225	-1.18713
H	-2.85906	5.03980	1.19889
H	-1.86546	3.53909	2.91678
H	-0.90829	1.34800	2.24916
H	2.86239	0.12149	-2.56807
H	5.29013	0.61989	-2.48446
H	6.43581	0.90940	-0.29190
H	5.12303	0.71051	1.81426
H	0.52129	-3.96902	1.88866
H	1.38210	-3.00849	3.12268
H	2.30825	-3.82357	1.81939

Originally assigned (incorrect) structure of 109{7j} ($CDCl_3$)

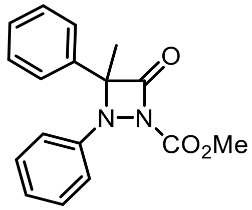

					Conf1	Conf2
Rel energy (kcal/mol):					0.00	2.08

**C-nom**	**iGau**	**Exp**	**Calc**	**diff**	**1**	**2**
C	4	167.10	167.30	0.20	[167.26	168.47]
C	19	159.10	151.08	-8.02	[151.08	150.96]
C	6	137.90	144.31	6.41	[144.32	144.01]
C	13	136.10	137.19	1.09	[137.36	131.50]
C	8	128.84	128.45	-0.39	[128.47	127.68]
C	10	128.84	128.45	-0.39	[128.47	127.68]
C	15	128.84	127.85	-0.99	[127.86	127.49]
C	17	128.78	127.85	-0.93	[127.86	127.49]
C	16	128.78	127.48	-1.30	[127.44	128.75]
C	14	125.80	125.44	-0.36	[125.35	128.33]
C	18	125.80	125.44	-0.36	[125.35	128.33]
C	9	124.80	123.52	-1.28	[123.53	123.10]
C	7	116.50	118.33	1.83	[118.31	118.84]
C	11	116.50	118.33	1.83	[118.31	118.84]
C	1	85.80	87.89	2.09	[87.85	89.28]
C	22	53.80	54.01	0.21	[54.01	54.03]
C	12	21.80	21.08	-0.72	[21.07	21.35]

13C chem shifts: RMSD=2.70ppm (MAE=1.67) N=17 {-8.02 6.41}

Fractions: 0.971 0.029

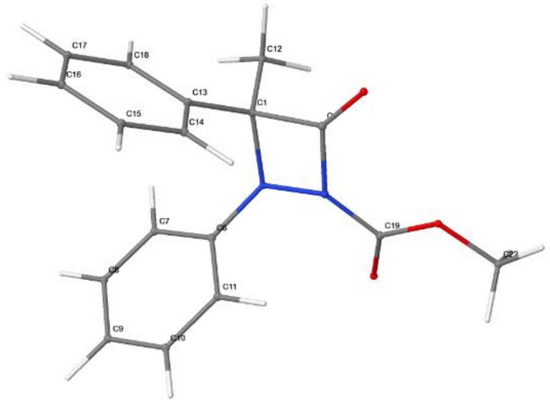

Conformer 1
Energy: -992.56783 Hartree (Rel: 0.0 kcal/mol)
XYZ coordinates for conf 1:

C	-0.75142	0.28548	0.81744
N	0.40471	0.19000	-0.17787
N	0.68173	-1.14949	0.29944
C	-0.43493	-1.18695	1.15574
O	-0.92827	-2.06099	1.81671
C	1.48945	1.11741	-0.09727
C	1.24408	2.42752	-0.53816
C	2.26546	3.37509	-0.51614
C	3.54690	3.02595	-0.07935
C	3.79186	1.71859	0.34229
C	2.77074	0.76511	0.34165
C	-0.53732	1.24123	1.99356
C	-2.10362	0.45071	0.14021
C	-2.21367	0.78239	-1.21524

C	-3.47124	0.94344	-1.80270
C	-4.63063	0.75991	-1.04794
C	-4.52690	0.40916	0.30087
C	-3.27289	0.25616	0.89202
C	1.26692	-2.11838	-0.52980
O	1.83192	-1.85285	-1.56738
O	1.12821	-3.32576	0.02590
C	1.69957	-4.41616	-0.72747
H	0.25627	2.69426	-0.90233
H	2.06047	4.38695	-0.85482
H	4.34472	3.76284	-0.07491
H	4.78366	1.43173	0.68124
H	2.97287	-0.24391	0.68212
H	-1.31773	1.07575	2.74108
H	0.43569	1.07842	2.46579
H	-0.59590	2.28119	1.66016
H	-1.31356	0.90075	-1.80821
H	-3.54063	1.20731	-2.85451
H	-5.60781	0.88267	-1.50672
H	-5.42289	0.25181	0.89496
H	-3.20614	-0.03036	1.93756
H	1.50317	-5.30637	-0.13165
H	1.21958	-4.49372	-1.70552
H	2.77386	-4.26601	-0.85533

Conformer 2
Energy: -992.57114 Hartree (Rel: 2.1 kcal/mol)
XYZ coordinates for conf 2:

C	-0.18527	-1.20041	0.81664
N	0.54101	0.17070	0.88609
N	1.55754	-0.35805	-0.00165
C	1.01527	-1.65461	-0.03719
O	1.41724	-2.69157	-0.49510
C	-0.11314	1.35965	0.43812
C	-1.07587	1.91309	1.29566
C	-1.73364	3.08906	0.94232
C	-1.42265	3.74230	-0.25440
C	-0.45086	3.20040	-1.09612
C	0.19951	2.01032	-0.76037
C	-0.14435	-1.86782	2.19209
C	-1.52870	-1.22767	0.11486
C	-1.59818	-1.12440	-1.28455
C	-2.82669	-1.13689	-1.94343
C	-4.01255	-1.25260	-1.21452
C	-3.95783	-1.35539	0.17594
C	-2.72664	-1.34333	0.83455
C	2.87731	0.11665	0.02450
O	3.20224	1.15044	0.56512
O	3.66934	-0.71889	-0.65508
C	5.05876	-0.33354	-0.71496
H	-1.29255	1.42413	2.24070
H	-2.48119	3.50438	1.61271
H	-1.92646	4.66675	-0.52147
H	-0.19549	3.69976	-2.02694
H	0.94436	1.59355	-1.42891
H	-0.46273	-2.91187	2.11397
H	0.87205	-1.83766	2.59428
H	-0.79695	-1.34938	2.89961
H	-0.68753	-1.04148	-1.87056
H	-2.85567	-1.06123	-3.02669
H	-4.97035	-1.26604	-1.72705
H	-4.87327	-1.44786	0.75361
H	-2.71091	-1.42902	1.91556
H	5.54448	-1.11288	-1.30048
H	5.16167	0.63768	-1.20410
H	5.48331	-0.28601	0.29031

Revised structure of 109{7j}, i.e regioisomer 113{7j-rev} (CDCl₃)

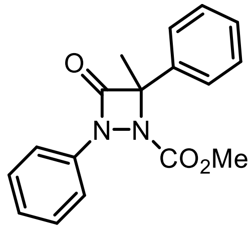

					Conf1	Conf2	Conf3	Conf4
Rel energy (kcal/mol):					0.00	0.05	1.51	2.35

C-nom	iGau	Exp	Calc	diff	1	2	3	4
C	1	167.10	168.07	0.97	[168.61	167.45	168.14	169.28]
C	19	159.10	160.34	1.24	[160.33	160.42	160.05	158.09]
C	6	137.90	137.20	-0.70	[137.20	137.16	137.55	137.65]
C	12	136.10	137.03	0.93	[137.36	137.02	133.84	133.51]
C	15	128.84	127.26	-1.58	[127.16	127.20	128.86	128.80]
C	14	128.84	127.44	-1.40	[127.38	127.42	128.27	128.11]
C	16	128.84	127.44	-1.40	[127.38	127.42	128.27	128.11]
C	8	128.78	127.96	-0.82	[127.98	127.92	128.03	128.06]
C	10	128.78	127.96	-0.82	[127.98	127.92	128.03	128.06]
C	13	125.80	125.13	-0.67	[125.10	125.00	126.50	127.03]
C	17	125.80	125.13	-0.67	[125.10	125.00	126.50	127.03]
C	9	124.80	123.38	-1.42	[123.52	123.23	123.37	123.61]
C	7	116.50	115.44	-1.06	[115.59	115.28	115.37	115.69]
C	11	116.50	115.44	-1.06	[115.59	115.28	115.37	115.69]
C	4	85.80	87.54	1.74	[87.28	87.84	87.41	86.88]
C	22	53.80	53.80	0.00	[53.82	53.87	52.89	53.38]
C	5	21.80	23.97	2.17	[24.25	24.26	18.21	18.37]

13C chem shifts: RMSD=1.20ppm (MAE=1.10) N=17 {-1.58 2.17}

Fractions: 0.497 0.455 0.039 0.010

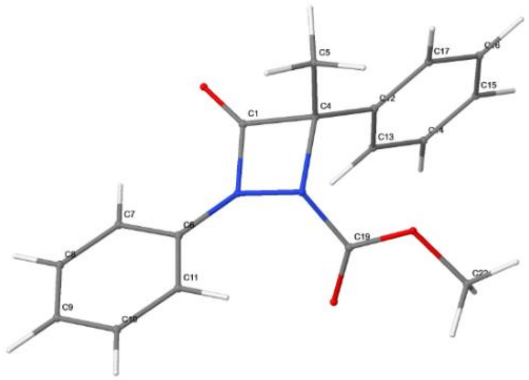

Conformer 1
Energy: -992.57652 Hartree (Rel: 0.0 kcal/mol)
XYZ coordinates for conf 1:

C	-0.16262	-0.73572	1.39892
N	0.86691	-0.16346	0.66179
N	-0.01202	0.78526	0.01222
C	-1.17142	0.30989	0.87254
C	-1.64862	1.29617	1.94196
C	1.99975	-0.76643	0.06384
C	2.38827	-0.43993	-1.23998
C	3.50382	-1.06584	-1.79679
C	4.22807	-2.01215	-1.06830
C	3.82828	-2.33153	0.23195
C	2.72139	-1.71010	0.80891
C	-2.31324	-0.26389	0.05331
C	-2.73354	0.38207	-1.11643

C	-3.81940	-0.11405	-1.83895
C	-4.49442	-1.25632	-1.40006
C	-4.07851	-1.90030	-0.23306
C	-2.99240	-1.40685	0.49347
O	-0.19672	-1.70290	2.12487
C	0.33754	2.13651	-0.04946
O	-0.48341	3.00536	-0.28322
O	1.65776	2.32268	0.07646
C	2.11319	3.67656	-0.12503
H	-2.18353	2.13048	1.48298
H	-2.32888	0.76954	2.61792
H	-0.81007	1.68761	2.52784
H	1.82045	0.28557	-1.81021
H	3.80293	-0.81134	-2.80980
H	5.09591	-2.49453	-1.50826
H	4.38307	-3.06613	0.80885
H	2.40966	-1.95387	1.81782
H	-2.21065	1.27227	-1.45274
H	-4.13722	0.39240	-2.74630
H	-5.33822	-1.64234	-1.96537
H	-4.59484	-2.79168	0.11255
H	-2.66330	-1.91616	1.39412
H	3.19546	3.63377	-0.00996
H	1.84481	4.02236	-1.12584
H	1.67239	4.33979	0.62234

Conformer 2
Energy: -992.57520 Hartree (Rel: 0.1 kcal/mol)
XYZ coordinates for conf 2:

C	0.05937	-0.85692	1.35023
N	1.00138	-0.12932	0.64188
N	0.02192	0.75015	0.05276
C	-1.07183	0.08883	0.88201
C	-1.66270	0.94270	2.00681
C	2.22337	-0.51697	0.04716
C	2.63202	0.02199	-1.17776
C	3.83999	-0.39454	-1.73751
C	4.63845	-1.33958	-1.08967
C	4.21884	-1.87072	0.13279
C	3.01777	-1.46335	0.71160
C	-2.13139	-0.58936	0.03345
C	-2.51274	-0.06046	-1.20523
C	-3.52415	-0.67253	-1.94812
C	-4.16060	-1.81715	-1.46261
C	-3.78015	-2.34893	-0.22826
C	-2.77119	-1.73836	0.51851
O	0.14057	-1.85964	2.02316
C	0.31457	2.11953	0.11337
O	1.42017	2.58726	0.29224
O	-0.79797	2.82071	-0.17004
C	-0.62657	4.25139	-0.22391
H	-2.33312	1.70481	1.60321
H	-2.23761	0.29043	2.67098
H	-0.87649	1.42722	2.59596
H	2.01455	0.75341	-1.68456
H	4.15482	0.02619	-2.68842
H	5.57881	-1.65670	-1.53077
H	4.83080	-2.60628	0.64734
H	2.68927	-1.87303	1.65942
H	-2.01645	0.82680	-1.58433
H	-3.81200	-0.25458	-2.90898
H	-4.94527	-2.29364	-2.04377
H	-4.26404	-3.24364	0.15362
H	-2.46660	-2.16447	1.46982
H	-1.61496	4.64979	-0.44974
H	-0.26989	4.62978	0.73675
H	0.08449	4.51797	-1.00901

Conformer 3
Energy: -992.57885 Hartree (Rel: 1.5 kcal/mol)
XYZ coordinates for conf 3:

C	0.32327	-1.56384	0.29062
N	1.06576	-0.42827	-0.00208
N	0.16542	0.45697	0.69704
C	-0.78924	-0.71529	0.95703
C	-0.89824	-0.99573	2.45181
C	2.46240	-0.24424	-0.12170
C	3.22515	-1.27055	-0.69962
C	4.60553	-1.11215	-0.81306
C	5.22930	0.05610	-0.36716
C	4.45745	1.07185	0.20078
C	3.07520	0.93118	0.32703
C	-2.09675	-0.67984	0.18169
C	-2.07099	-0.58517	-1.22059
C	-3.25463	-0.56043	-1.95571
C	-4.48790	-0.64055	-1.30289
C	-4.52449	-0.74169	0.08780
C	-3.33774	-0.75940	0.82519
O	0.54163	-2.74207	0.12972
C	-0.21941	1.61984	0.01589
O	0.36304	2.09306	-0.93898
O	-1.26182	2.17150	0.65633
C	-1.75918	3.39467	0.08051
H	-1.48814	-0.22061	2.94898
H	-1.36981	-1.96844	2.62026
H	0.09739	-1.00900	2.90322
H	2.73890	-2.17569	-1.04336
H	5.19415	-1.90976	-1.25768
H	6.30441	0.17462	-0.46474
H	4.92976	1.98573	0.55024
H	2.48066	1.72217	0.76717
H	-1.12057	-0.52721	-1.74487
H	-3.21238	-0.48630	-3.03863
H	-5.41116	-0.62739	-1.87525
H	-5.47712	-0.80635	0.60605
H	-3.39203	-0.83683	1.90535
H	-2.59124	3.69376	0.71702
H	-0.97974	4.16004	0.08131
H	-2.10245	3.22187	-0.94222

Conformer 4
Energy: -992.57894 Hartree (Rel: 2.3 kcal/mol)
XYZ coordinates for conf 4:

C	0.09978	-1.59486	-0.03771
N	0.91384	-0.46678	-0.07681
N	0.07650	0.27173	0.84730
C	-0.94306	-0.86590	0.84630
C	-1.04649	-1.49509	2.23230
C	2.32545	-0.37731	-0.13905
C	3.00650	-1.21790	-1.03177
C	4.39797	-1.15857	-1.09715
C	5.11221	-0.26502	-0.29455
C	4.42130	0.57039	0.58592
C	3.02957	0.52022	0.67117
C	-2.25867	-0.57166	0.14278
C	-2.25096	-0.18142	-1.20708
C	-3.44176	0.07856	-1.88210
C	-4.66575	-0.05367	-1.22050
C	-4.68506	-0.44399	0.11837
C	-3.49016	-0.70009	0.79569
O	0.24168	-2.70974	-0.48108
C	-0.30016	1.58912	0.56810
O	-1.22283	2.13137	1.14326
O	0.52718	2.17117	-0.31361
C	0.29123	3.57524	-0.54152
H	-1.57292	-0.82732	2.91975
H	-1.58150	-2.44768	2.17605
H	-0.04690	-1.67985	2.63491
H	2.44950	-1.90866	-1.65386
H	4.92367	-1.81375	-1.78610
H	6.19547	-0.21894	-0.35661
H	4.96539	1.26794	1.21666

H	2.49560	1.16280	1.36092
H	-1.30754	-0.07772	-1.73764
H	-3.41367	0.37826	-2.92591
H	-5.59551	0.14429	-1.74640
H	-5.63038	-0.54959	0.64318
H	-3.53077	-1.00014	1.83683
H	1.05201	3.88084	-1.25886
H	-0.70921	3.72961	-0.95144
H	0.39449	4.13458	0.39125

Originally assigned (incorrect) structure of 115{3a} ($CDCl_3$)

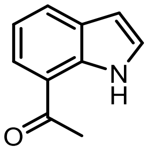

					Conf1
Rel energy (kcal/mol):					0.00

C-nom	iGau	Exp	Calc	diff	1
C	10	193.89	201.28	7.39	[201.28]
C	4	136.37	136.47	0.10	[136.47]
C	5	131.79	129.45	-2.34	[129.45]
C	6	125.37	127.48	2.11	[127.48]
C	8	123.70	126.28	2.58	[126.28]
C	2	122.68	125.69	3.01	[125.69]
C	3	122.33	120.29	-2.04	[120.29]
C	1	118.43	118.76	0.33	[118.76]
C	9	111.43	100.93	-10.50	[100.93]
C	11	27.63	26.60	-1.03	[26.60]

13C chem shifts: RMSD=4.43ppm (MAE=3.14) N=10 {-10.50 7.39}

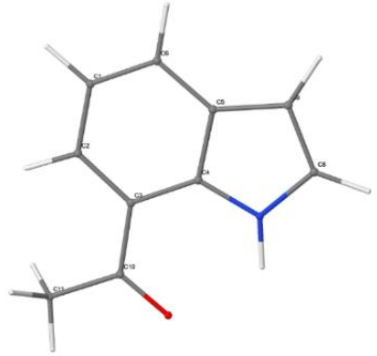

Conformer 1
Energy: -516.46863 Hartree (Rel: 0.0 kcal/mol)
XYZ coordinates for conf 1:

C	-0.35703	2.45354	-0.00001
C	0.82062	1.68954	0.00004
C	0.79075	0.28676	0.00007
C	-0.48845	-0.31469	0.00004
C	-1.69632	0.44440	-0.00002
C	-1.61177	1.84492	-0.00004
N	-0.83519	-1.63820	0.00006
C	-2.21258	-1.75230	0.00001
C	-2.77803	-0.50267	-0.00004
C	2.00462	-0.55717	0.00012
C	3.37199	0.10191	-0.00004
O	1.91518	-1.78847	-0.00011
H	-0.28478	3.53722	-0.00003
H	1.77644	2.20350	0.00006
H	-2.51446	2.45066	-0.00009
H	-0.14250	-2.37455	0.00009
H	-2.67865	-2.72765	0.00001
H	-3.83622	-0.28055	-0.00008
H	4.13814	-0.67541	-0.00000
H	3.50199	0.73814	-0.88307
H	3.50212	0.73836	0.88281

Revised structure of 115{3a}, i.e 117{3a-rev} ($CDCl_3$)

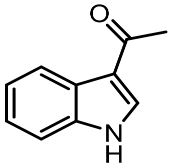

					Conf1	Conf2
Rel energy (kcal/mol):					0.00	1.25

C-nom	iGau	Exp	Calc	diff	1	2
C	10	193.89	192.65	-1.24	[192.75	191.84]
C	4	136.37	135.89	-0.48	[135.81	136.55]
C	8	131.79	132.87	1.08	[132.82	133.29]
C	5	125.37	126.15	0.78	[126.26	125.24]
C	2	123.70	122.74	-0.96	[122.80	122.22]
C	6	122.68	122.28	-0.40	[122.54	120.11]
C	1	122.33	122.24	-0.09	[122.28	121.95]
C	9	118.43	116.00	-2.43	[115.89	116.87]
C	3	111.43	110.63	-0.80	[110.51	111.60]
C	11	27.63	27.47	-0.16	[27.13	30.26]

13C chem shifts: RMSD=1.06ppm (MAE=0.84) N=10 {-2.43 1.08}

Fractions: 0.891 0.109

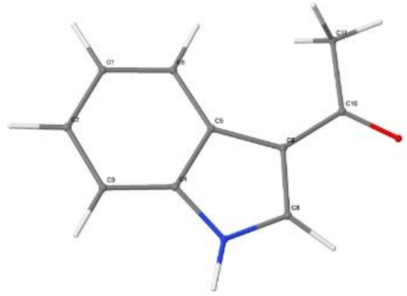

Conformer 1
Energy: -516.46629 Hartree (Rel: 0.0 kcal/mol)
XYZ coordinates for conf 1:

C	-2.34188	-1.58085	0.00003
C	-3.15129	-0.42596	0.00025
C	-2.58915	0.84706	0.00025
C	-1.19334	0.92871	0.00002
C	-0.35859	-0.21888	-0.00021
C	-0.95354	-1.49188	-0.00020
N	-0.36299	2.04179	-0.00004
C	0.94123	1.64295	-0.00025
C	1.01107	0.25805	-0.00045
C	2.22497	-0.55689	-0.00074
C	3.57070	0.15621	0.00056
O	2.17281	-1.78750	0.00021
H	-3.20887	1.73937	0.00041
H	-4.23267	-0.53074	0.00043
H	-0.32844	-2.37716	-0.00039
H	-0.67284	3.00321	0.00007
H	1.73591	2.37572	-0.00030
H	-2.81527	-2.55901	0.00004
H	3.67482	0.79572	0.88511
H	3.67556	0.79778	-0.88239
H	4.36917	-0.58846	0.00008

Conformer 2
Energy: -516.46827 Hartree (Rel: 1.2 kcal/mol)
XYZ coordinates for conf 2:

C	2.24155	-1.65358	-0.00006
C	3.10802	-0.54269	-0.00020
C	2.60380	0.75342	-0.00016
C	1.21453	0.90260	0.00002
C	0.31281	-0.19871	0.00019
C	0.85910	-1.49630	0.00012
N	0.45407	2.06259	0.00003
C	-0.86795	1.74386	0.00017
C	-1.02689	0.36652	0.00038
C	-2.35780	-0.25381	0.00069
C	-2.45865	-1.76869	-0.00004
O	-3.37830	0.43618	-0.00072
H	3.26148	1.61813	-0.00029
H	4.18279	-0.69990	-0.00034
H	0.21982	-2.37222	0.00020
H	0.82494	3.00217	-0.00005
H	-1.63270	2.50657	0.00022
H	2.66263	-2.65521	-0.00011
H	-1.96538	-2.19271	0.88293
H	-1.96529	-2.19188	-0.88338
H	-3.51148	-2.05826	-0.00027

Table S1: Comparison between actual (115{3a}) compound spectral data with known compound references

Original reference data for actual structure (**115{3a}**)[37]		References for revised structure (**117{3a-rev}**)[38]			
**^{1}H**[37]	**^{13}C**[37]	**^{1}H**[38a]	**^{13}C**[38a]	**^{1}H**[38b]	**^{1}H**[38c]
8.87 (s, 1H)	193.89	8.93 (bs, NH 1H)	194.2	8.91 (br s, 1H)	8.64 (s, 1H)
8.43 – 8.36 (m, 1H)	136.37	8.43−8.37 (m, CH, 1H)	136.6	8.42 – 8.38 (m, 1H)	8.43 – 8.37 (m, 1H)
7.87 (d, *J* = 2.3 Hz, 1H)	131.79	7.87 (d, *J* = 3.0 Hz, CH, 1H)	132.3	7.88 – 7.87 (m, 1H)	7.87 (d, *J* = 3.0 Hz, 1H)
7.46 – 7.39 (m, 1H)	125.37	7.48−7.39 (m, CH, 1H)	125.4	7.44 – 7.40 (m, 1H)	7.46 – 7.39 (m, 1H)
7.31 – 7.28 (m, 2H)	123.70	7.35−7.23 (m, CH, 2H)	123.6	7.32 – 7.27 (m, 2H)	7.34 – 7.28 (m, 2H)
2.56 (s, 3H)	122.68	2.56 (s, CH_3, 3H)	122.6	2.56 (s, 3H)	2.56 (s, 3H)
	122.33		122.2		
	118.43		118.2		
	111.43		111.7		
	27.63		27.6		

Originally assigned (incorrect) structure of 116{3i} ($CDCl_3$)

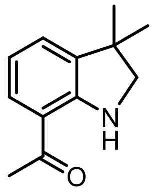

					Conf1
	Rel energy (kcal/mol):				0.00
**C-nom**	**iGau**	**Exp**	**Calc**	**diff**	**1**
C-C	8	196.52	198.91	2.39	[198.91]
C-C	4	154.75	153.02	-1.73	[153.02]
C-C	5	135.17	138.65	3.48	[138.65]
C-C	3	128.00	116.64	-11.36	[116.64]
C-C	12	41.13	41.89	0.76	[41.89]
C-CH	2	130.56	129.50	-1.06	[129.50]
C-CH	6	122.50	127.45	4.95	[127.45]
C-CH	1	107.16	115.05	7.89	[115.05]
C-CH2	11	61.61	60.92	-0.69	[60.92]
C-CH3	13	27.82	28.77	0.95	[28.77]
C-CH3	10	27.82	26.64	-1.18	[26.64]
C-CH3	14	26.20	25.43	-0.77	[25.43]

13C chem shifts: RMSD=4.49ppm (MAE=3.10) N=12 {-11.36 7.89}

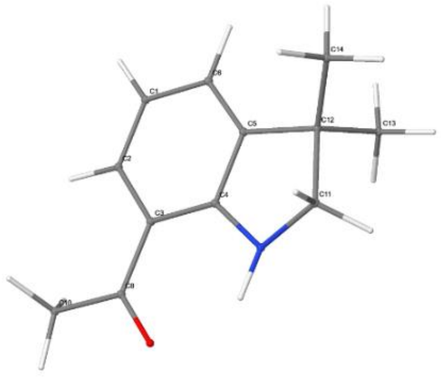

Conformer 1
Energy: -596.29856 Hartree (Rel: 0.0 kcal/mol)
XYZ coordinates for conf 1:

C	0.47235	2.51218	-0.01662
C	1.58045	1.68091	0.01117
C	1.45966	0.26806	0.01572
C	0.14650	-0.26059	0.00140
C	-0.98650	0.58718	-0.03674
C	-0.82788	1.95730	-0.03991
N	-0.24347	-1.57103	0.03329
C	2.61955	-0.63523	-0.00241
O	2.47816	-1.86429	-0.04532
C	4.02174	-0.04760	0.02433
C	-1.65822	-1.68393	-0.33187
C	-2.24863	-0.27368	-0.00608
C	-2.85136	-0.26195	1.41688
C	-3.30858	0.15040	-1.03301
H	0.60054	3.59031	-0.02466
H	2.57123	2.12430	0.01939
H	-1.69457	2.61408	-0.06615
H	0.44747	-2.27783	-0.18572
H	4.17467	0.57564	0.91299
H	4.74453	-0.86564	0.02961
H	4.20191	0.58486	-0.85294
H	-1.76838	-1.91267	-1.40315

H	-2.14960	-2.48016	0.23673
H	-3.16334	0.75061	1.69694
H	-3.72982	-0.91706	1.46964
H	-2.12052	-0.60843	2.15583
H	-2.89318	0.17487	-2.04635
H	-4.15488	-0.54748	-1.02638
H	-3.70145	1.14772	-0.80337

Revised structure of 116{3i}, i.e 118{3i-rev} ($CDCl_3$)

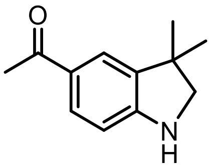

					Conf1	Conf2	Conf3	Conf4
Rel energy (kcal/mol):					0.00	0.00	0.20	0.20

C-nom	iGau	Exp	Calc	diff	1	2	3	4
C-C	8	196.52	195.05	-1.47	[195.04	195.05	195.05	195.06]
C-C	4	154.75	154.12	-0.63	[154.10	154.11	154.15	154.14]
C-C	5	135.17	136.06	0.89	[136.46	136.46	135.51	135.50]
C-C-ipso	1	128.00	127.41	-0.59	[127.49	127.49	127.30	127.28]
C-C	12	41.13	42.50	1.37	[42.49	42.48	42.52	42.52]
C-CH	2	130.56	131.47	0.91	[131.88	131.88	130.91	130.91]
C-CH	6	122.50	124.68	2.18	[124.18	124.18	125.37	125.37]
C-CH	3	107.16	106.94	-0.22	[106.43	106.43	107.66	107.63]
C-CH2	11	61.61	61.57	-0.04	[61.55	61.56	61.60	61.60]
C-CH3	14	27.82	26.75	-1.07	[28.26	25.20	28.30	25.24]
C-CH3	13	27.82	26.75	-1.07	[25.20	28.26	25.25	28.31]
C-Ac-CH3	10	26.20	26.17	-0.03	[26.11	26.11	26.26	26.26]

13C chem shifts: RMSD=1.06ppm (MAE=0.87) N=12 {-1.47 2.18}

Fractions: 0.291 0.291 0.209 0.209

NOTICE:
messy spectrum, removed reported peak at 138.11 and added broad peak at approx 128 - C5{ipso-Acetyl}

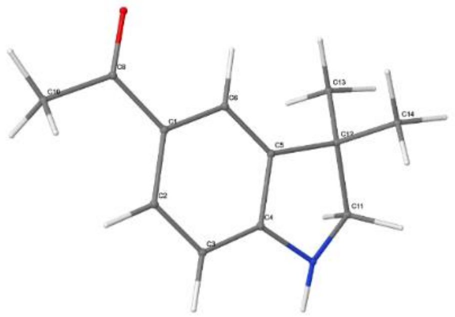

Conformer 1
Energy: -596.29563 Hartree (Rel: 0.0 kcal/mol)
XYZ coordinates for conf 1:

C	1.67364	0.08221	-0.00321
C	1.55629	1.48446	0.01355
C	0.31384	2.11766	0.01082
C	-0.83621	1.31940	-0.01058
C	-0.74199	-0.09081	-0.03039
C	0.49364	-0.70083	-0.02120
N	-2.16098	1.71206	0.01343
C	2.98300	-0.61293	-0.00588
O	3.05254	-1.84142	-0.02325
C	4.26042	0.21516	0.01391
C	-3.00076	0.57004	-0.38659
C	-2.15046	-0.68174	-0.00321
C	-2.35708	-1.83507	-0.99509
C	-2.49322	-1.14436	1.43095
H	2.44920	2.10181	0.02297
H	0.24324	3.20171	0.01862
H	0.59692	-1.78177	-0.03723
H	-2.40179	2.62770	-0.34596
H	5.11740	-0.46108	0.01188
H	4.31959	0.87346	-0.86085
H	4.30659	0.85210	0.90504
H	-3.96457	0.59362	0.13171

H	-3.19240	0.58650	-1.47040
H	-1.73956	-2.69937	-0.72514
H	-3.40377	-2.16331	-0.99476
H	-2.09232	-1.53514	-2.01489
H	-2.40042	-0.31783	2.14395
H	-3.52108	-1.52508	1.47851
H	-1.81726	-1.94547	1.75013

Conformer 2
Energy: -596.29532 Hartree (Rel: 0.0 kcal/mol)
XYZ coordinates for conf 2:

C	-1.67364	0.08220	-0.00325
C	-1.55630	1.48445	0.01365
C	-0.31384	2.11763	0.01101
C	0.83621	1.31938	-0.01048
C	0.74199	-0.09084	-0.03044
C	-0.49362	-0.70086	-0.02131
N	2.16096	1.71208	0.01365
C	-2.98297	-0.61290	-0.00585
O	-3.05255	-1.84143	-0.02258
C	-4.26042	0.21518	0.01336
C	3.00072	0.57006	-0.38662
C	2.15045	-0.68171	-0.00331
C	2.49325	-1.14440	1.43084
C	2.35711	-1.83502	-0.99522
H	-2.44921	2.10179	0.02316
H	-0.24319	3.20168	0.01901
H	-0.59697	-1.78179	-0.03746
H	2.40164	2.62762	-0.34607
H	-5.11736	-0.46111	0.01056
H	-4.30718	0.85167	0.90478
H	-4.31906	0.87384	-0.86115
H	3.19228	0.58673	-1.47044
H	3.96455	0.59362	0.13162
H	1.81734	-1.94558	1.74995
H	3.52113	-1.52504	1.47837
H	2.40038	-0.31792	2.14389
H	2.09241	-1.53505	-2.01504
H	3.40378	-2.16329	-0.99485
H	1.73953	-2.69931	-0.72536

Conformer 3
Energy: -596.29563 Hartree (Rel: 0.2 kcal/mol)
XYZ coordinates for conf 3:

C	1.68943	0.21718	-0.00098
C	1.49831	1.61269	0.01422
C	0.22995	2.18281	0.01095
C	-0.87973	1.32611	-0.01077
C	-0.71465	-0.07524	-0.03042
C	0.55255	-0.62552	-0.01846
N	-2.22264	1.65275	0.01321
C	3.07620	-0.30853	-0.00125
O	4.04835	0.44555	0.01308
C	3.29072	-1.81570	-0.01893
C	-3.00332	0.47021	-0.38766
C	-2.09132	-0.73733	-0.00347
C	-2.24047	-1.89901	-0.99613
C	-2.41243	-1.21695	1.43015
H	2.38024	2.24521	0.02304
H	0.10305	3.26187	0.01812
H	0.67241	-1.70505	-0.03291
H	-2.50762	2.55493	-0.34816
H	4.36339	-2.01827	-0.01571
H	2.83180	-2.29164	0.85575
H	2.84125	-2.26972	-0.91005
H	-3.96777	0.44458	0.12931
H	-3.19405	0.47675	-1.47178
H	-1.58406	-2.73411	-0.72511
H	-3.27022	-2.27681	-0.99833
H	-1.98818	-1.58647	-2.01531
H	-2.36078	-0.38711	2.14337

H	-3.42041	-1.64763	1.47672
H	-1.69880	-1.98449	1.75040

Conformer 4
Energy: -596.29532 Hartree (Rel: 0.2 kcal/mol)
XYZ coordinates for conf 4:

C	-1.68944	0.21723	-0.00107
C	-1.49831	1.61273	0.01403
C	-0.22995	2.18284	0.01064
C	0.87974	1.32614	-0.01105
C	0.71465	-0.07530	-0.03042
C	-0.55253	-0.62552	-0.01846
N	2.22252	1.65269	0.01262
C	-3.07617	-0.30853	-0.00117
O	-4.04838	0.44551	0.01416
C	-3.29068	-1.81570	-0.01973
C	3.00346	0.47007	-0.38765
C	2.09131	-0.73732	-0.00339
C	2.41234	-1.21658	1.43044
C	2.24046	-1.89932	-0.99570
H	-2.38026	2.24521	0.02291
H	-0.10299	3.26191	0.01753
H	-0.67246	-1.70504	-0.03296
H	2.50789	2.55521	-0.34754
H	-4.36337	-2.01819	-0.01753
H	-2.84035	-2.26939	-0.91056
H	-2.83269	-2.29196	0.85527
H	3.19475	0.47654	-1.47164
H	3.96760	0.44468	0.12989
H	1.69867	-1.98403	1.75080
H	3.42032	-1.64721	1.47714
H	2.36061	-0.38652	2.14339
H	1.98844	-1.58705	-2.01500
H	3.27013	-2.27734	-0.99762
H	1.58384	-2.73422	-0.72457

Originally determined (correct) structure of 121{7a} ($CDCl_3$)

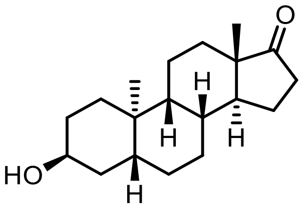

					Conf1	Conf2	Conf3	Conf4
		Rel energy (kcal/mol):			0.00	0.04	1.59	1.65

C-nom	iGau	Exp	Calc	diff	1	2	3	4
C	2	66.40	66.16	-0.24	[66.20	66.13	66.15	66.13]
C-C	12	47.90	49.26	1.36	[49.33	49.32	48.22	48.21]
C	10	45.70	46.34	0.64	[46.45	46.41	45.06	45.07]
C	11	44.00	44.11	0.11	[43.97	44.02	45.87	45.83]
C	4	38.70	38.97	0.27	[39.41	39.44	32.22	32.23]
C-C	5	37.80	37.79	-0.01	[37.96	37.97	35.19	35.22]
C	6	35.60	36.27	0.67	[36.26	36.12	37.32	37.41]
C	16	35.60	35.92	0.32	[35.89	35.90	36.29	36.27]
C	9	35.10	35.46	0.36	[35.57	35.56	33.92	33.98]
C	3	34.20	36.60	2.40	[38.34	34.67	35.38	38.91]
C	13	30.10	31.36	1.26	[31.40	31.40	30.79	30.77]
C	1	28.70	29.37	0.67	[27.64	31.18	31.21	27.87]
C	8	27.30	27.98	0.68	[28.29	28.26	23.55	23.52]
C	7	24.90	24.83	-0.07	[24.78	24.70	26.20	26.19]
C	15	22.70	23.33	0.63	[23.30	23.32	23.57	23.58]
C	14	20.50	21.32	0.82	[21.37	21.37	20.63	20.66]
C	22	15.10	15.73	0.63	[15.80	15.75	15.06	15.09]
C	19	13.60	12.99	-0.61	[12.55	12.53	19.64	19.61]

13C chem shifts: RMSD=0.86ppm (MAE=0.65) N=18 {-0.61 2.40}

Fractions: 0.485 0.452 0.033 0.030

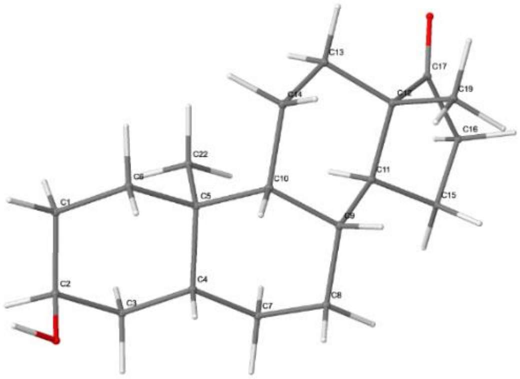

Conformer 1
Energy: -892.52533 Hartree (Rel: 0.0 kcal/mol)
XYZ coordinates for conf 1:

C	3.77432	-1.46450	-0.53855
C	4.42436	-0.07918	-0.51255
C	3.40908	1.01734	-0.87098
C	2.14546	0.93465	0.00110
C	1.42092	-0.44494	-0.11572
C	2.46994	-1.52874	0.27635
C	1.18910	2.11656	-0.19950
C	0.13346	2.11641	0.91092
C	-0.66975	0.80238	1.00303
C	0.25060	-0.47156	0.94488
C	-1.83167	0.70037	0.00330
C	-2.70898	-0.55410	0.25410

C	-1.85920	-1.81169	0.05171
C	-0.60395	-1.77785	0.95676
C	-2.85383	1.84982	-0.13682
C	-3.99200	1.19623	-0.95696
C	-3.83554	-0.32124	-0.75356
O	-4.49773	-1.17771	-1.30832
C	-3.41412	-0.57701	1.63891
H	-1.40935	0.56408	-1.00199
H	0.78724	-0.46705	1.90522
C	0.95150	-0.72676	-1.56151
O	4.94658	0.10587	0.81169
H	-1.12430	0.79731	2.00410
H	2.50032	0.99860	1.04147
H	5.29653	1.00982	0.86388
H	4.49038	-2.19561	-0.14367
H	3.59221	-1.74151	-1.58390
H	5.25596	-0.05625	-1.23534
H	3.88066	2.00263	-0.73655
H	3.16242	0.94697	-1.93861
H	2.71959	-1.40565	1.33789
H	2.04206	-2.53284	0.16877
H	1.75389	3.05849	-0.16854
H	0.71846	2.07578	-1.19148
H	0.65378	2.26956	1.86730
H	-0.55320	2.96484	0.80002
H	-2.44149	-2.71467	0.27458
H	-1.57504	-1.88107	-1.00424
H	-0.93075	-1.94557	1.99085
H	0.02713	-2.63981	0.71439
H	-2.43552	2.72516	-0.64238
H	-3.21360	2.18429	0.84345
H	-5.00000	1.51677	-0.67075
H	-3.89237	1.40267	-2.03002
H	-4.01833	0.31811	1.81964
H	-4.08300	-1.44362	1.68509
H	-2.69872	-0.66143	2.46108
H	1.79125	-0.73814	-2.26239
H	0.47343	-1.70899	-1.62877
H	0.24137	0.01465	-1.93559

Conformer 2
Energy: -892.52543 Hartree (Rel: 0.0 kcal/mol)
XYZ coordinates for conf 2:

C	3.77104	-1.45043	-0.55342
C	4.42363	-0.06118	-0.51578
C	3.41171	1.03596	-0.85979
C	2.14429	0.94203	0.00711
C	1.42184	-0.43793	-0.11725
C	2.47322	-1.52101	0.26998
C	1.18573	2.12205	-0.19391
C	0.12793	2.11717	0.91453
C	-0.67190	0.80068	1.00436
C	0.25128	-0.47146	0.94273
C	-1.83475	0.69868	0.00562
C	-2.70830	-0.55914	0.25264
C	-1.85507	-1.81346	0.04470
C	-0.60022	-1.77983	0.95014
C	-2.86035	1.84567	-0.12932
C	-3.99729	1.19179	-0.95098
C	-3.83646	-0.32592	-0.75314
O	-4.49662	-1.18239	-1.31034
C	-3.41201	-0.58896	1.63797
H	-1.41340	0.56739	-1.00069
H	0.78745	-0.46858	1.90338
C	0.95271	-0.71423	-1.56421
O	4.92984	0.24479	0.79238
H	-1.12566	0.79209	2.00577
H	2.49364	1.00419	1.04871
H	5.51671	-0.48239	1.05469
H	4.48555	-2.19764	-0.17821
H	3.58193	-1.72049	-1.60009

H	5.25352	-0.03165	-1.24035
H	3.88896	2.01155	-0.70130
H	3.16847	0.97611	-1.92820
H	2.72936	-1.39471	1.33007
H	2.04724	-2.52630	0.16731
H	1.74871	3.06467	-0.15902
H	0.71742	2.08257	-1.18716
H	0.64639	2.27098	1.87170
H	-0.56131	2.96352	0.80366
H	-2.43491	-2.71907	0.26333
H	-1.57021	-1.87716	-1.01143
H	-0.92692	-1.95193	1.98355
H	0.03275	-2.63964	0.70481
H	-2.44507	2.72405	-0.63202
H	-3.22029	2.17546	0.85248
H	-5.00601	1.50857	-0.66307
H	-3.89889	1.40235	-2.02337
H	-4.01974	0.30317	1.82170
H	-4.07734	-1.45841	1.68238
H	-2.69559	-0.67253	2.45935
H	1.79078	-0.71060	-2.26710
H	0.48418	-1.70072	-1.63837
H	0.23444	0.02287	-1.93068

Conformer 3
Energy: -892.52796 Hartree (Rel: 1.6 kcal/mol)
XYZ coordinates for conf 3:

C	3.96254	-1.40523	-0.21435
C	4.48060	-0.16375	0.52559
C	3.72501	1.08903	0.07813
C	2.19439	0.94571	0.17212
C	1.64040	-0.30751	-0.56906
C	2.43959	-1.54845	-0.07940
C	1.49780	2.25151	-0.26894
C	-0.00630	2.07146	-0.57111
C	-0.61551	0.86972	0.16881
C	0.13804	-0.48646	-0.13229
C	-2.11036	0.71797	-0.14688
C	-2.70220	-0.63417	0.32262
C	-2.05204	-1.80428	-0.45436
C	-0.68910	-1.39851	-1.06779
C	-3.11079	1.77683	0.36335
C	-4.48197	1.10074	0.13446
C	-4.19548	-0.40822	0.06689
O	-5.02421	-1.26945	-0.15960
C	-2.58587	-0.85752	1.85586
H	-2.20804	0.71977	-1.24513
H	0.19049	-1.01367	0.83001
C	1.80184	-0.18034	-2.10281
O	4.29462	-0.28028	1.94383
H	-0.51457	1.06948	1.24531
H	1.96306	0.78676	1.23479
H	4.71574	-1.10814	2.22572
H	4.45803	-2.30284	0.18363
H	4.26093	-1.33830	-1.26860
H	5.55370	-0.03315	0.31072
H	4.04984	1.93818	0.69363
H	4.02212	1.32126	-0.95318
H	2.21208	-1.71159	0.98162
H	2.10396	-2.44607	-0.61511
H	1.63577	2.99789	0.52427
H	2.00123	2.65980	-1.15556
H	-0.55231	2.98390	-0.30130
H	-0.16513	1.94035	-1.64929
H	-1.90876	-2.65702	0.22034
H	-2.72690	-2.15294	-1.24566
H	-0.12468	-2.30944	-1.29966
H	-0.85294	-0.89556	-2.02854
H	-3.02451	2.72827	-0.16971
H	-2.94895	1.98644	1.42738
H	-5.23076	1.31534	0.90494

```
H         -4.92749        1.39622       -0.82410
H         -3.02753       -0.04313        2.44031
H         -3.10728       -1.78248        2.12611
H         -1.54030       -0.95891        2.16153
H          2.83803        0.03647       -2.38121
H          1.52541       -1.11432       -2.60379
H          1.18286        0.61408       -2.52828

Conformer 4
Energy: -892.52790 Hartree (Rel:  1.7 kcal/mol)
XYZ coordinates for conf 4:
C          3.96246       -1.42101       -0.18926
C          4.47955       -0.17380        0.53102
C          3.72737        1.07732        0.05748
C          2.19739        0.94136        0.15841
C          1.64089       -0.31922       -0.56841
C          2.43710       -1.55738       -0.06587
C          1.50122        2.24442       -0.29215
C         -0.00244        2.06284       -0.59445
C         -0.61334        0.87035        0.15850
C          0.13788       -0.48988       -0.12937
C         -2.10869        0.71806       -0.15448
C         -2.70239       -0.62830        0.32899
C         -2.05461       -1.80713       -0.43674
C         -0.69146       -1.40972       -1.05519
C         -3.10682        1.78377        0.34590
C         -4.47935        1.10780        0.12495
C         -4.19547       -0.40231        0.07209
O         -5.02594       -1.26415       -0.14524
C         -2.58525       -0.83652        1.86429
H         -2.20715        0.70901       -1.25260
H          0.18976       -1.00752        0.83801
C          1.80316       -0.20853       -2.10331
O          4.29965       -0.40014        1.93679
H         -0.51136        1.08116        1.23290
H          1.97005        0.79136        1.22386
H          4.55255        0.41559        2.39796
H          4.45049       -2.30627        0.23657
H          4.26939       -1.36767       -1.24113
H          5.55405       -0.05046        0.31880
H          4.05558        1.94717        0.64695
H          4.02357        1.29184       -0.97862
H          2.20111       -1.71792        0.99306
H          2.10529       -2.45636       -0.60187
H          1.63824        2.99666        0.49593
H          2.00559        2.64646       -1.18096
H         -0.54746        2.97925       -0.33632
H         -0.15948        1.91911       -1.67121
H         -1.91212       -2.65328        0.24630
H         -2.73059       -2.16255       -1.22405
H         -0.12868       -2.32383       -1.27804
H         -0.85515       -0.91609       -2.02081
H         -3.01939        2.72968       -0.19678
H         -2.94375        2.00371        1.40767
H         -5.22698        1.33126        0.89406
H         -4.92535        1.39460       -0.83601
H         -3.02515       -0.01558        2.44089
H         -3.10796       -1.75785        2.14414
H         -1.53963       -0.93688        2.17008
H          2.84225       -0.00888       -2.38378
H          1.51477       -1.14394       -2.59455
H          1.19471        0.58957       -2.53719
```

Originally assigned (incorrect) structure of 123{6b} ($CDCl_3$)

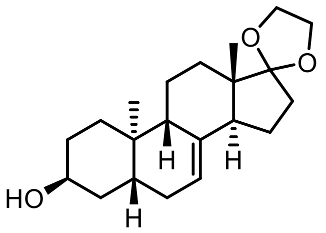

					Conf1	Conf2	Conf3
		Rel energy (kcal/mol):			0.00	0.22	0.22

**C-nom**	**iGau**	**Exp**	**Calc**	**diff**	**1**	**2**	**3**
C	9	136.80	140.73	3.93	[140.85	140.78	140.49]
C	17	122.30	118.42	-3.88	[118.17	118.59	118.60]
C	8	119.80	117.20	-2.60	[117.00	117.18	117.51]
C	2	67.20	66.06	-1.14	[66.06	66.07	66.06]
C	26	65.20	65.51	0.31	[65.44	65.56	65.55]
C	25	64.60	65.08	0.48	[64.35	65.60	65.60]
C	10	46.60	48.66	2.06	[48.72	48.63	48.61]
C	12	43.60	47.19	3.59	[46.91	47.40	47.40]
C	11	41.50	45.85	4.35	[45.81	45.88	45.87]
C	3	40.00	37.19	-2.81	[38.22	38.21	34.66]
C	16	37.50	35.97	-1.53	[35.62	36.21	36.22]
C	4	36.60	35.25	-1.35	[35.24	35.26	35.27]
C	5	33.60	34.85	1.25	[34.86	34.86	34.84]
C	6	33.50	33.96	0.46	[34.01	33.99	33.87]
C	7	28.80	30.34	1.54	[30.32	30.34	30.36]
C	1	28.30	29.19	0.89	[28.22	28.22	31.56]
C	13	26.20	28.84	2.64	[28.79	28.86	28.88]
C	15	23.20	22.34	-0.86	[22.27	22.38	22.40]
C	18	22.50	21.21	-1.29	[21.51	20.99	21.00]
C	14	21.40	20.69	-0.71	[20.62	20.73	20.74]
C	21	14.10	11.45	-2.65	[11.46	11.46	11.43]

13C chem shifts: RMSD=2.28ppm (MAE=1.92) N=21 {-3.88 4.35}

Fractions: 0.420 0.291 0.289

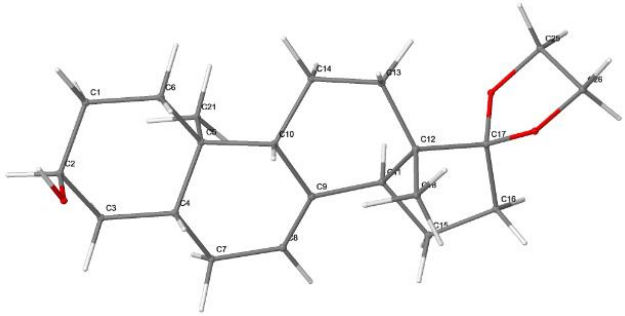

Conformer 1
Energy: -1045.13487 Hartree (Rel: 0.0 kcal/mol)
XYZ coordinates for conf 1:

C	4.54148	-1.63818	-0.31143
C	5.21600	-0.43009	0.34133
C	4.52991	0.87317	-0.08997
C	3.01099	0.85178	0.14873
C	2.30056	-0.35752	-0.53896
C	3.02103	-1.65403	-0.08303
C	2.36858	2.19957	-0.21890
C	0.86429	2.14694	-0.25423
C	0.15785	1.01122	-0.18723
C	0.83020	-0.35606	-0.01162
C	-1.34191	0.94974	-0.35494
C	-2.00891	-0.12835	0.54170
C	-1.44657	-1.53321	0.19312

C	-0.09295	-1.46817	-0.56036
C	-2.20035	2.21598	-0.15752
C	-3.63084	1.67199	0.11500
C	-3.50148	0.12459	0.18130
C	-1.81597	0.16373	2.04589
H	-1.52573	0.61057	-1.38677
H	0.91398	-0.54359	1.07177
C	2.32782	-0.24710	-2.07861
O	5.13765	-0.62460	1.76083
O	-4.44238	-0.43381	1.10196
O	-3.79007	-0.46495	-1.08819
C	-5.13758	-0.92630	-1.04011
C	-5.26698	-1.37366	0.41189
H	2.86584	0.70453	1.22869
H	4.98789	-2.55575	0.09106
H	4.76954	-1.62156	-1.38462
H	6.27525	-0.39886	0.03828
H	4.97454	1.71930	0.45600
H	4.75039	1.05314	-1.15121
H	2.83382	-1.80166	0.98859
H	2.59470	-2.52280	-0.59940
H	2.70601	2.96748	0.49356
H	2.73818	2.54079	-1.20056
H	0.34655	3.09588	-0.39246
H	-1.31370	-2.10851	1.11795
H	-2.16503	-2.08296	-0.42255
H	-0.27517	-1.30189	-1.62918
H	0.39892	-2.44364	-0.48162
H	-2.16532	2.87304	-1.03214
H	-1.83625	2.79997	0.69491
H	-4.03405	2.04369	1.06123
H	-4.34228	1.94236	-0.67153
H	-0.75921	0.10626	2.32280
H	-2.17936	1.15475	2.33658
H	-2.36431	-0.57498	2.63865
H	3.34430	-0.12026	-2.46524
H	1.91959	-1.15414	-2.53892
H	1.73145	0.60071	-2.43068
H	5.49012	0.17434	2.18465
H	-5.25410	-1.72875	-1.77235
H	-5.84198	-0.11429	-1.27371
H	-6.28482	-1.30570	0.80676
H	-4.89242	-2.39693	0.55231

Conformer 2
Energy: -1045.13522 Hartree (Rel: 0.2 kcal/mol)
XYZ coordinates for conf 2:

C	4.53904	-1.64789	-0.25209
C	5.21341	-0.42162	0.36597
C	4.53018	0.86932	-0.10531
C	3.01035	0.85678	0.12871
C	2.30071	-0.37191	-0.52448
C	3.01800	-1.65489	-0.02731
C	2.37079	2.19353	-0.28201
C	0.86651	2.14158	-0.32036
C	0.15852	1.00917	-0.22125
C	0.82869	-0.35277	-0.00234
C	-1.34103	0.94498	-0.38920
C	-2.01044	-0.10484	0.53745
C	-1.45240	-1.51909	0.22510
C	-0.09397	-1.47892	-0.52179
C	-2.19784	2.21604	-0.23015
C	-3.63355	1.67699	0.02761
C	-3.50135	0.13078	0.16448
C	-1.81629	0.23089	2.03288
H	-1.52617	0.57501	-1.41052
H	0.90859	-0.50829	1.08639
C	2.33343	-0.30793	-2.06669
O	5.13137	-0.57393	1.79042
O	-4.38446	-0.44539	1.11477
O	-3.82847	-0.47320	-1.10103

```
C         -4.89122       -1.40693       -0.90571
C         -5.51841       -0.93526        0.40230
H          2.86127        0.74262        1.21214
H          4.98301       -2.55344        0.17927
H          4.76999       -1.66401       -1.32467
H          6.27343       -0.40060        0.06474
H          4.97404        1.73051        0.41735
H          4.75435        1.01810       -1.17059
H          2.82794       -1.76952        1.04785
H          2.59183       -2.53844       -0.51807
H          2.70683        2.98250        0.40770
H          2.74390        2.50407       -1.27246
H          0.35037        3.08656       -0.48841
H         -1.33110       -2.07577        1.16252
H         -2.16707       -2.08054       -0.38494
H         -0.27032       -1.34389       -1.59592
H          0.39544       -2.45256       -0.41169
H         -2.14885        2.85654       -1.11630
H         -1.84586        2.81505        0.61690
H         -4.06000        2.08393        0.94928
H         -4.32034        1.91234       -0.78942
H         -0.75787        0.19603        2.30768
H         -2.19245        1.22491        2.29596
H         -2.35163       -0.49823        2.64878
H          3.35143       -0.19395       -2.45326
H          1.92573       -1.22790       -2.50107
H          1.73942        0.52963       -2.44615
H          5.48583        0.23591        2.19114
H         -4.49824       -2.42901       -0.81345
H         -5.56728       -1.35734       -1.76426
H         -6.25436       -0.13597        0.23336
H         -5.97815       -1.73202        0.99192

Conformer 3
Energy: -1045.13487 Hartree (Rel:  0.2 kcal/mol)
XYZ coordinates for conf 3:
C          4.53975       -1.63199       -0.27551
C          5.21441       -0.40972        0.36192
C          4.52984        0.88272       -0.08689
C          3.00813        0.86204        0.13967
C          2.30123       -0.36160       -0.52590
C          3.02126       -1.64692       -0.03973
C          2.36816        2.20039       -0.26455
C          0.86394        2.14673       -0.30410
C          0.15717        1.01286       -0.21306
C          0.82885       -0.35016       -0.00497
C         -1.34243        0.94816       -0.38136
C         -2.01036       -0.11080        0.53591
C         -1.45078       -1.52145        0.21046
C         -0.09200       -1.47273       -0.53538
C         -2.20078        2.21660       -0.21034
C         -3.63588        1.67337        0.04202
C         -3.50165        0.12618        0.16554
C         -1.81644        0.21164        2.03431
H         -1.52759        0.58735       -1.40598
H          0.90788       -0.51507        1.08245
C          2.33479       -0.28443       -2.06754
O          5.13403       -0.44940        1.79425
O         -4.38362       -0.45938        1.11120
O         -3.82858       -0.46755       -1.10495
C         -4.89181       -1.40232       -0.91760
C         -5.51817       -0.94225        0.39498
H          2.85400        0.74157        1.22105
H          4.98870       -2.55108        0.12847
H          4.76372       -1.63403       -1.35037
H          6.27393       -0.38139        0.05961
H          4.96967        1.72630        0.46099
H          4.75750        1.04665       -1.14854
H          2.83952       -1.76410        1.03710
H          2.59250       -2.52970       -0.52938
H          2.70276        2.98528        0.43019
```

H	2.74163	2.51711	-1.25302
H	0.34632	3.09220	-0.46483
H	-1.32930	-2.08685	1.14267
H	-2.16472	-2.07776	-0.40514
H	-0.26786	-1.32800	-1.60833
H	0.39833	-2.44698	-0.43420
H	-2.15251	2.86560	-1.09031
H	-1.84954	2.80787	0.64240
H	-4.06347	2.07179	0.96689
H	-4.32248	1.91479	-0.77341
H	-0.75789	0.17706	2.30856
H	-2.19485	1.20231	2.30649
H	-2.34998	-0.52427	2.64368
H	3.35172	-0.15558	-2.45222
H	1.93681	-1.20450	-2.51085
H	1.73240	0.55042	-2.43952
H	5.50430	-1.29885	2.08324
H	-4.49934	-2.42543	-0.83483
H	-5.56833	-1.34463	-1.77530
H	-6.25385	-0.14116	0.23364
H	-5.97797	-1.74411	0.97760

Revised structure of 123{6b}, i.e 126{6b-rev} ($CDCl_3$)

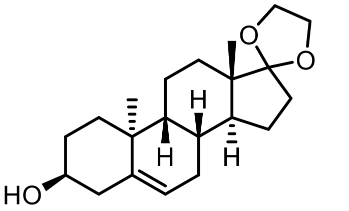

				Conf1	Conf2		
Rel energy (kcal/mol):				0.00	1.34		

C-nom	iGau	Exp	Calc	diff		1	2	
C	4	136.80	135.41	-1.39	[	135.16	137.75	]
C	7	122.30	122.27	-0.03	[	122.48	120.26	]
C	17	119.80	119.10	-0.70	[	119.10	119.13	]
C-CH	2	67.20	65.77	-1.43	[	65.59	67.48	]
C	26	65.20	65.66	0.46	[	65.66	65.64	]
C	25	64.60	65.69	1.09	[	65.83	64.33	]
C-C	12	46.60	49.23	2.63	[	49.27	48.81	]
C	10	43.60	44.44	0.84	[	44.50	43.91	]
C	11	41.50	42.52	1.02	[	42.49	42.85	]
C	3	40.00	41.03	1.03	[	41.21	39.28	]
C-C	5	37.50	37.57	0.07	[	37.58	37.48	]
C-CH2	6	36.60	39.15	2.55	[	39.28	37.91	]
C	16	33.60	35.66	2.06	[	35.71	35.15	]
C	9	33.50	34.08	0.58	[	34.05	34.34	]
C	1	28.80	29.87	1.07	[	29.74	31.14	]
C	13	28.30	29.54	1.24	[	29.47	30.16	]
C	8	26.20	26.59	0.39	[	26.60	26.54	]
C	14	23.20	24.50	1.30	[	24.51	24.45	]
C	15	22.50	23.90	1.40	[	23.89	23.97	]
C	21	21.40	21.72	0.32	[	21.69	22.04	]
C	18	14.10	13.91	-0.19	[	13.84	14.58	]

13C chem shifts: RMSD=1.26ppm (MAE=1.04) N=21 {-1.43 2.63}

Fractions: 0.905 0.095

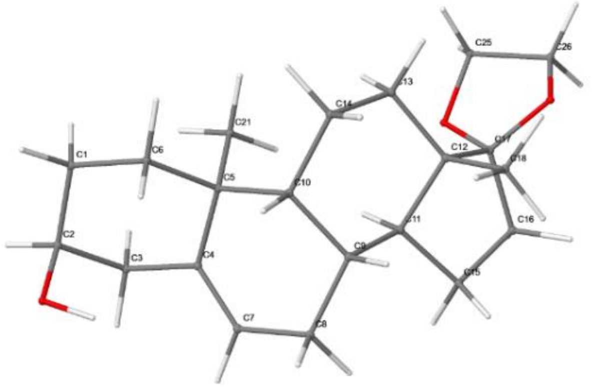

Conformer 1
Energy: -1045.12772 Hartree (Rel: 0.0 kcal/mol)
XYZ coordinates for conf 1:

C	4.25725	-1.68552	0.27321
C	4.80129	-0.64845	-0.72151
C	3.66181	-0.12386	-1.61554
C	2.54313	0.44295	-0.76000
C	1.91446	-0.52262	0.25225
C	3.09038	-1.12033	1.09828
C	2.21915	1.74133	-0.84861
C	1.18804	2.43777	-0.00790
C	0.21457	1.48293	0.70472
C	0.95282	0.22673	1.25955

C	-1.00925	1.06762	-0.12095
C	-2.04297	0.27810	0.73061
C	-1.38502	-0.99694	1.28137
C	-0.05303	-0.69092	2.01158
C	-1.86019	2.14012	-0.83463
C	-3.21999	1.42889	-1.09183
C	-3.14948	0.06643	-0.34232
C	-2.64743	1.10298	1.89096
H	-0.67312	0.37857	-0.90552
H	1.62870	0.62024	2.03166
C	1.23764	-1.68877	-0.51619
O	5.44608	0.43695	-0.04454
O	-4.38626	-0.34303	0.23284
O	-2.79076	-0.95688	-1.28744
C	-3.68933	-2.05407	-1.12775
C	-4.93914	-1.37229	-0.58531
H	2.74950	2.35990	-1.57404
H	5.06587	-1.99283	0.94745
H	3.95034	-2.58290	-0.27969
H	5.58167	-1.10383	-1.34215
H	4.05458	0.64994	-2.28513
H	3.30412	-0.94588	-2.24911
H	3.48797	-0.32678	1.74461
H	2.69928	-1.89630	1.76884
H	0.63278	3.15849	-0.62235
H	-2.06154	-1.50409	1.98277
H	-1.21389	-1.68695	0.45239
H	0.43061	-1.63622	2.28749
H	-0.28696	-0.19874	2.96444
H	-1.39494	2.48164	-1.76447
H	-1.99380	3.02529	-0.20119
H	-4.06452	2.00610	-0.70446
H	-3.40020	1.24663	-2.15458
H	-1.90630	1.32060	2.66496
H	-3.06629	2.05906	1.56152
H	-3.45723	0.53233	2.35479
H	1.95310	-2.20534	-1.16147
H	0.84310	-2.43713	0.17755
H	0.41791	-1.34840	-1.15355
H	4.74613	1.06126	0.21333
H	-3.29208	-2.78814	-0.41293
H	-3.82792	-2.53318	-2.10074
H	-5.54973	-0.94736	-1.39497
H	-5.56403	-2.01366	0.04136
H	1.70845	3.05067	0.74553
H	-0.16227	2.02820	1.58082

Conformer 2
Energy: -1045.12985 Hartree (Rel: 1.3 kcal/mol)
XYZ coordinates for conf 2:

C	4.22397	-1.70822	0.24661
C	4.81817	-0.67957	-0.72548
C	3.71529	-0.03828	-1.57991
C	2.56914	0.50181	-0.74430
C	1.92390	-0.48403	0.23507
C	3.08367	-1.10699	1.08084
C	2.20476	1.78494	-0.85102
C	1.14476	2.46162	-0.03082
C	0.19315	1.49390	0.69517
C	0.95461	0.24798	1.24483
C	-1.03681	1.06590	-0.11559
C	-2.04779	0.25585	0.74460
C	-1.36581	-1.01264	1.28213
C	-0.03128	-0.68916	2.00031
C	-1.91270	2.13535	-0.80731
C	-3.28066	1.42632	-1.02306
C	-3.17056	0.04888	-0.31265
C	-2.64727	1.06472	1.91884
H	-0.70165	0.38997	-0.91099
H	1.62911	0.65154	2.01313
C	1.24672	-1.62560	-0.56999

O	5.47446	0.38494	-0.02636
O	-4.43586	-0.35801	0.21261
O	-2.77519	-0.97367	-1.23558
C	-3.93568	-1.73426	-1.55912
C	-4.72745	-1.67339	-0.25880
H	2.74690	2.42130	-1.55192
H	5.01519	-2.07848	0.91459
H	3.88089	-2.58035	-0.32435
H	5.53960	-1.17894	-1.39223
H	4.15479	0.76757	-2.17833
H	3.36201	-0.80152	-2.28730
H	3.50864	-0.31764	1.71227
H	2.67699	-1.87174	1.75509
H	0.57148	3.15738	-0.65802
H	-2.02751	-1.53009	1.99123
H	-1.19890	-1.70026	0.45153
H	0.46872	-1.62941	2.26418
H	-0.26269	-0.20783	2.95941
H	-1.47400	2.47539	-1.75042
H	-2.02874	3.02218	-0.17264
H	-4.10958	1.99296	-0.58932
H	-3.51011	1.26784	-2.08134
H	-1.90211	1.27180	2.69149
H	-3.06833	2.02526	1.60586
H	-3.45509	0.48725	2.37825
H	1.95682	-2.10889	-1.24661
H	0.86520	-2.40498	0.09727
H	0.41719	-1.26638	-1.18320
H	6.14785	-0.01646	0.54612
H	-3.62067	-2.73959	-1.84963
H	-4.49279	-1.27287	-2.38796
H	-5.80924	-1.76445	-0.39038
H	-4.38311	-2.43317	0.45664
H	1.64472	3.10108	0.71488
H	-0.18159	2.03477	1.57525

Originally assigned (incorrect) structure of 124{7b} ($CDCl_3$)

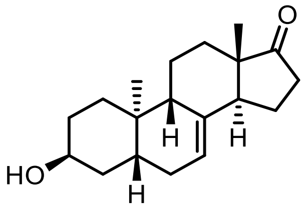

					Conf1	Conf2
	Rel energy (kcal/mol):				0.00	0.03

C-nom	iGau	Exp	Calc	diff	1	2
C	17	221.00	220.73	-0.27	[220.75	220.71]
C	9	134.10	138.71	4.61	[138.56	138.86]
C	8	130.70	118.45	-12.25	[118.61	118.28]
C	2	66.30	66.00	-0.30	[65.98	66.03]
C	10	47.60	48.82	1.22	[48.80	48.84]
C	12	46.60	48.15	1.55	[48.15	48.14]
C	11	39.40	45.39	5.99	[45.39	45.40]
C	3	39.10	36.34	-2.76	[34.63	38.12]
C	16	36.50	36.16	-0.34	[36.16	36.16]
C	4	35.70	35.31	-0.39	[35.31	35.32]
C	5	32.20	34.95	2.75	[34.95	34.96]
C	6	30.00	33.87	3.87	[33.82	33.92]
C	7	29.20	30.30	1.10	[30.31	30.29]
C	13	29.20	30.29	1.09	[30.30	30.28]
C	1	27.20	29.92	2.72	[31.52	28.25]
C	15	23.00	21.72	-1.28	[21.72	21.71]
C	19	22.10	20.07	-2.03	[20.07	20.06]
C	14	17.50	20.05	2.55	[20.05	20.04]
C	22	12.50	11.35	-1.15	[11.33	11.37]

13C chem shifts: RMSD=3.74ppm (MAE=2.54) N=19 {-12.25 5.99}

Fractions: 0.511 0.489

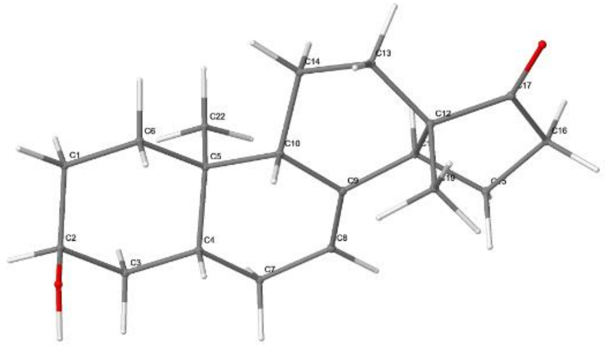

Conformer 1
Energy: -891.31001 Hartree (Rel: 0.0 kcal/mol)
XYZ coordinates for conf 1:

C	3.97529	-1.31360	-0.43062
C	4.50608	-0.13403	0.39536
C	3.70190	1.13467	0.10598
C	2.18653	0.93082	0.27708
C	1.62211	-0.25067	-0.57573
C	2.46223	-1.51392	-0.25079
C	1.42064	2.24413	0.04477
C	-0.06879	2.04915	-0.05484
C	-0.65411	0.84899	-0.14793
C	0.14758	-0.45929	-0.10474
C	-2.13231	0.65262	-0.37799
C	-2.68705	-0.57975	0.38305
C	-2.00365	-1.87534	-0.12005

C	-0.64244	-1.58931	-0.80868
C	-3.14927	1.77683	-0.10633
C	-4.50479	1.03442	-0.16537
C	-4.18473	-0.44825	0.09233
O	-4.99366	-1.35568	0.06012
C	-2.55690	-0.44053	1.92382
H	-2.24799	0.40047	-1.44737
H	0.22497	-0.76255	0.95248
C	1.67418	0.04747	-2.08959
O	4.39652	-0.38373	1.80414
H	-0.67900	2.95108	-0.09997
H	2.02635	0.64241	1.32523
H	4.84657	-1.22398	1.98727
H	4.50868	-2.23182	-0.14477
H	4.21950	-1.13898	-1.48671
H	5.56351	0.04181	0.13942
H	4.04170	1.93161	0.78011
H	3.93191	1.46867	-0.91442
H	2.27369	-1.80153	0.79226
H	2.13689	-2.35586	-0.87381
H	1.65839	2.94955	0.85480
H	1.77817	2.73602	-0.87540
H	-1.84696	-2.55599	0.72518
H	-2.66563	-2.40341	-0.81693
H	-0.05520	-2.51331	-0.81751
H	-0.80880	-1.32347	-1.85960
H	-3.08581	2.58457	-0.84163
H	-2.98401	2.22269	0.88057
H	-5.25028	1.39546	0.55163
H	-4.96708	1.10563	-1.15806
H	-3.05358	0.45579	2.31077
H	-3.01406	-1.31027	2.40806
H	-1.50665	-0.39304	2.22607
H	2.68037	0.32421	-2.42033
H	1.37591	-0.83365	-2.66934
H	1.00060	0.86674	-2.36048

Conformer 2
Energy: -891.31005 Hartree (Rel: 0.0 kcal/mol)
XYZ coordinates for conf 2:

C	3.97566	-1.33144	-0.41313
C	4.50549	-0.14744	0.39786
C	3.70410	1.12407	0.08727
C	2.18942	0.92773	0.26567
C	1.62239	-0.25968	-0.57717
C	2.45945	-1.52310	-0.24396
C	1.42436	2.24070	0.02762
C	-0.06545	2.04719	-0.06983
C	-0.65222	0.84720	-0.15523
C	0.14783	-0.46159	-0.10294
C	-2.13063	0.65103	-0.38344
C	-2.68676	-0.57416	0.38816
C	-2.00513	-1.87494	-0.10397
C	-0.64432	-1.59651	-0.79643
C	-3.14613	1.77883	-0.12155
C	-4.50252	1.03761	-0.17437
C	-4.18442	-0.44309	0.09691
O	-4.99474	-1.34950	0.07369
C	-2.55581	-0.42213	1.92765
H	-2.24640	0.38987	-1.45062
H	0.22607	-0.75651	0.95655
C	1.67397	0.02780	-2.09304
O	4.40375	-0.51123	1.78210
H	-0.67414	2.94980	-0.12101
H	2.03302	0.64509	1.31660
H	4.66859	0.26093	2.30719
H	4.50218	-2.24164	-0.10079
H	4.22846	-1.16729	-1.46815
H	5.56461	0.02119	0.14412
H	4.04625	1.94176	0.73993
H	3.93316	1.44410	-0.93853

H	2.26227	-1.81111	0.79678
H	2.13688	-2.36397	-0.87005
H	1.66358	2.95060	0.83356
H	1.78181	2.72719	-0.89533
H	-1.84820	-2.54807	0.74718
H	-2.66830	-2.40861	-0.79540
H	-0.05801	-2.52105	-0.79760
H	-0.81147	-1.33960	-1.84945
H	-3.08157	2.58017	-0.86374
H	-2.98055	2.23291	0.86157
H	-5.24793	1.40609	0.53890
H	-4.96417	1.10035	-1.16793
H	-3.05010	0.47870	2.30719
H	-3.01494	-1.28660	2.41936
H	-1.50529	-0.37509	2.22908
H	2.68248	0.29110	-2.42776
H	1.36586	-0.85470	-2.66534
H	1.00816	0.85172	-2.36918

Revised structure of 124{7b}, i.e 127{7b-rev} ($CDCl_3$)

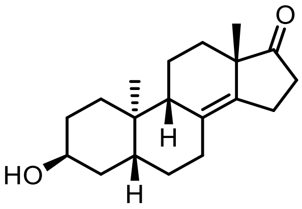

					Conf1	Conf2	Conf3	Conf4
Rel energy (kcal/mol):					0.00	0.01	3.27	3.33

**C-nom**	**iGau**	**Exp**	**Calc**	**diff**	**1**	**2**	**3**	**4**
C	11	134.10	134.56	0.46	[134.52	134.60	134.58	134.66]
C	9	130.70	130.78	0.08	[130.83	130.70	134.67	134.55]
C	2	66.30	66.09	-0.21	[66.05	66.14	65.96	65.98]
C	12	47.60	49.49	1.89	[49.47	49.49	51.11	51.12]
C	10	46.60	47.71	1.11	[47.69	47.73	48.20	48.24]
C	5	39.40	39.78	0.38	[39.78	39.80	36.43	36.44]
C	4	39.10	38.87	-0.23	[38.91	38.85	36.64	36.64]
C	16	36.50	37.40	0.90	[37.41	37.40	36.51	36.50]
C	3	35.70	36.62	0.92	[34.83	38.46	35.10	38.71]
C	6	32.20	34.65	2.45	[34.56	34.73	34.98	35.13]
C	8	30.00	31.74	1.74	[31.76	31.75	28.84	28.83]
C	1	29.20	29.88	0.68	[31.57	28.15	31.38	28.09]
C	7	29.20	30.38	1.18	[30.36	30.42	26.75	26.78]
C	13	27.20	28.51	1.31	[28.50	28.51	29.60	29.61]
C	15	23.00	24.69	1.69	[24.69	24.69	23.99	23.99]
C	19	22.10	21.61	-0.49	[21.60	21.60	25.17	25.18]
C	14	17.50	18.56	1.06	[18.55	18.54	23.16	23.19]
C	20	12.50	13.33	0.83	[13.31	13.35	14.23	14.26]

13C chem shifts: RMSD=1.16ppm (MAE=0.98) N=18 {-0.49 2.45}

Fractions: 0.504 0.492 0.002 0.002

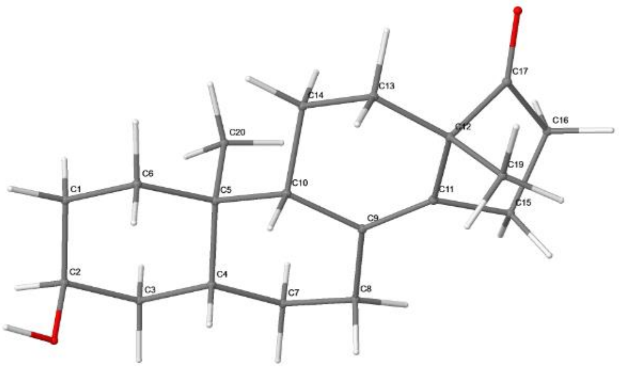

Conformer 1
Energy: -891.31913 Hartree (Rel: 0.0 kcal/mol)
XYZ coordinates for conf 1:

C	-3.76212	-1.44505	0.58694
C	-4.46576	-0.09456	0.40087
C	-3.51260	1.06296	0.70576
C	-2.19145	0.95847	-0.07669
C	-1.43842	-0.39079	0.16842
C	-2.43417	-1.52931	-0.18370
C	-1.29307	2.18027	0.14474
C	-0.05614	2.11963	-0.76657
C	0.68249	0.80387	-0.64438
C	-0.20284	-0.43237	-0.81728
C	2.00619	0.72709	-0.42425
C	2.77706	-0.57923	-0.31759
C	1.86131	-1.74497	0.07344
C	0.60616	-1.75813	-0.81214

C	2.97657	1.86352	-0.12501
C	3.94901	1.25801	0.91173
C	3.84457	-0.25892	0.73840
O	4.50317	-1.08395	1.34237
C	3.55664	-0.88439	-1.62931
C	-0.96769	-0.53934	1.63137
O	-4.91349	0.08354	-0.95108
H	-0.65877	-0.34926	-1.81746
H	-1.85980	3.09725	-0.06562
H	-5.45818	-0.68618	-1.18099
H	-4.43151	-2.25442	0.26073
H	-3.59431	-1.60779	1.65958
H	-5.33250	-0.04007	1.07933
H	-4.01211	2.00610	0.44971
H	-3.32602	1.09154	1.78701
H	-2.65030	-1.48626	-1.25971
H	-1.98363	-2.50972	0.00828
H	-2.46808	0.95717	-1.14178
H	-0.98086	2.24206	1.19615
H	-0.39717	2.22815	-1.80902
H	0.60418	2.96996	-0.57243
H	2.39824	-2.69659	-0.01840
H	1.58214	-1.64262	1.12772
H	0.91113	-1.97628	-1.84300
H	-0.03313	-2.59515	-0.51594
H	2.48616	2.76455	0.24990
H	3.52799	2.15557	-1.02985
H	4.98803	1.59042	0.81662
H	3.63370	1.49194	1.93732
H	4.19517	-0.04592	-1.92894
H	4.19485	-1.76407	-1.48928
H	2.86150	-1.07970	-2.45152
H	-1.77678	-0.36670	2.34781
H	-0.59029	-1.55190	1.81401
H	-0.16151	0.16068	1.87101

Conformer 2
Energy: -891.31911 Hartree (Rel: 0.0 kcal/mol)
XYZ coordinates for conf 2:

C	-3.76594	-1.46083	0.56972
C	-4.46691	-0.11247	0.39721
C	-3.51071	1.04448	0.71950
C	-2.19298	0.95072	-0.06836
C	-1.43773	-0.39884	0.16804
C	-2.43087	-1.53777	-0.19094
C	-1.29685	2.17406	0.15394
C	-0.06158	2.11929	-0.75979
C	0.68032	0.80513	-0.64146
C	-0.20224	-0.43230	-0.81830
C	2.00419	0.73109	-0.42162
C	2.77825	-0.57369	-0.31973
C	1.86536	-1.74333	0.06650
C	0.60998	-1.75605	-0.81886
C	2.97164	1.86888	-0.11819
C	3.94530	1.26202	0.91663
C	3.84478	-0.25458	0.73762
O	4.50549	-1.08004	1.33863
C	3.55878	-0.87177	-1.63252
C	-0.96671	-0.55469	1.63006
O	-4.92459	-0.05270	-0.96170
H	-0.65903	-0.34651	-1.81786
H	-1.86600	3.09083	-0.05182
H	-5.31717	0.82440	-1.09880
H	-4.43623	-2.25621	0.22156
H	-3.60575	-1.63177	1.64153
H	-5.33544	-0.06603	1.07389
H	-4.00496	1.99984	0.48708
H	-3.32135	1.06111	1.80119
H	-2.63850	-1.49768	-1.26827
H	-1.97935	-2.51685	0.00622
H	-2.47533	0.95173	-1.13261

```
H         -0.98252        2.23316        1.20477
H         -0.40442        2.22933       -1.80154
H          0.59655        2.97104       -0.56444
H          2.40466       -2.69318       -0.02947
H          1.58635       -1.64611        1.12128
H          0.91520       -1.96908       -1.85069
H         -0.02739       -2.59567       -0.52616
H          2.47893        2.76737        0.25987
H          3.52258        2.16550       -1.02182
H          4.98349        1.59746        0.82306
H          3.62906        1.49131        1.94298
H          4.19501       -0.03045       -1.92908
H          4.19940       -1.75016       -1.49561
H          2.86419       -1.06612       -2.45541
H         -1.77814       -0.39627        2.34713
H         -0.58082       -1.56545        1.80410
H         -0.16687        0.15022        1.87692

Conformer 3
Energy: -891.31382 Hartree (Rel:  3.3 kcal/mol)
XYZ coordinates for conf 3:
C         -3.88457       -1.42621        0.46224
C         -4.54906       -0.12850       -0.01493
C         -3.69247        1.08446        0.35123
C         -2.23039        0.95566       -0.11596
C         -1.53617       -0.35231        0.37461
C         -2.43755       -1.54735       -0.03840
C         -1.40588        2.18936        0.26171
C         -0.02291        2.13674       -0.39636
C          0.69293        0.79898       -0.31678
C         -0.14271       -0.48852       -0.34891
C          2.03793        0.73369       -0.32923
C          2.84186       -0.53915       -0.55084
C          2.02056       -1.83679       -0.51879
C          0.69451       -1.65761        0.21815
C          3.03585        1.83395        0.00390
C          4.10489        1.08756        0.84538
C          3.91776       -0.40302        0.53432
O          4.52039       -1.31468        1.06760
C          3.61106       -0.45748       -1.90654
C         -1.34735       -0.34372        1.91180
O         -4.70649       -0.11214       -1.44132
H         -0.37921       -0.72116       -1.40177
H         -1.92448        3.10093       -0.06385
H         -5.18511       -0.91903       -1.69045
H         -4.47222       -2.28889        0.11607
H         -3.91871       -1.45708        1.55929
H         -5.53862       -0.02914        0.45994
H         -4.13687        1.98058       -0.10062
H         -3.74011        1.22848        1.43846
H         -2.45669       -1.60948       -1.13444
H         -2.01140       -2.48999        0.32428
H         -2.25756        0.89977       -1.21503
H         -1.31337        2.26193        1.35304
H         -0.16237        2.35651       -1.46788
H          0.61575        2.94133       -0.01579
H          1.81736       -2.15837       -1.54854
H          2.62252       -2.62718       -0.05354
H          0.12675       -2.59299        0.16820
H          0.90166       -1.46754        1.27763
H          2.58650        2.65968        0.56080
H          3.49175        2.26819       -0.89558
H          5.13664        1.39503        0.64174
H          3.93968        1.22781        1.92044
H          4.29565        0.39530       -1.96000
H          4.19895       -1.37112       -2.05010
H          2.89396       -0.36893       -2.72966
H         -2.24310        0.00521        2.43463
H         -1.12905       -1.34906        2.28681
H         -0.51916        0.30515        2.21607
```

Conformer 4
Energy: -891.31392 Hartree (Rel: 3.3 kcal/mol)
XYZ coordinates for conf 4:

C	-3.88816	-1.44317	0.43955
C	-4.54918	-0.14426	-0.02348
C	-3.69379	1.06769	0.36847
C	-2.23292	0.94955	-0.10253
C	-1.53693	-0.36170	0.37680
C	-2.43500	-1.55565	-0.04750
C	-1.40973	2.18227	0.28116
C	-0.02795	2.13657	-0.37975
C	0.69049	0.79985	-0.30866
C	-0.14264	-0.48900	-0.34721
C	2.03557	0.73753	-0.32416
C	2.84152	-0.53251	-0.55429
C	2.02297	-1.83208	-0.52686
C	0.69762	-1.65928	0.21286
C	3.03191	1.83818	0.01239
C	4.10476	1.08973	0.84713
C	3.92013	-0.39963	0.52862
O	4.52647	-1.31262	1.05528
C	3.60697	-0.44218	-1.91158
C	-1.34948	-0.36527	1.91409
O	-4.70553	-0.24212	-1.44670
H	-0.37862	-0.71642	-1.40127
H	-1.93041	3.09556	-0.03677
H	-5.05821	0.60596	-1.76037
H	-4.47206	-2.29375	0.06712
H	-3.93300	-1.48434	1.53520
H	-5.54257	-0.05339	0.44498
H	-4.13648	1.98080	-0.05738
H	-3.74170	1.19478	1.45847
H	-2.44410	-1.61669	-1.14312
H	-2.01037	-2.49803	0.31802
H	-2.26334	0.89994	-1.20239
H	-1.31522	2.24745	1.37269
H	-0.16959	2.36238	-1.44980
H	0.60937	2.94039	0.00459
H	1.81889	-2.14948	-1.55770
H	2.62731	-2.62328	-0.06614
H	0.13145	-2.59532	0.15885
H	0.90591	-1.47405	1.27297
H	2.58223	2.66001	0.57481
H	3.48459	2.27814	-0.88595
H	5.13527	1.40060	0.64243
H	3.94202	1.22391	1.92334
H	4.28942	0.41249	-1.96259
H	4.19659	-1.35371	-2.06121
H	2.88748	-0.35136	-2.73237
H	-2.24876	-0.02930	2.43937
H	-1.12370	-1.37243	2.27941
H	-0.52684	0.28720	2.22583

Originally assigned (incorrect) structure of 125{7c} ($CDCl_3$)

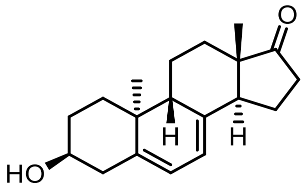

					Conf1	Conf2
		Rel energy (kcal/mol):			0.00	0.00

C-nom	iGau	Exp	Calc	diff	1	2
C	17	220.00	220.47	0.47	[220.44	220.51]
C	4	141.30	140.52	-0.78	[140.42	140.63]
C	9	138.10	137.09	-1.01	[137.42	136.76]
C	7	119.10	121.45	2.35	[121.66	121.24]
C	8	117.30	118.01	0.71	[117.86	118.17]
C	2	70.10	66.20	-3.90	[66.02	66.38]
C	12	49.40	48.46	-0.94	[48.46	48.46]
C	10	47.80	47.54	-0.26	[47.59	47.49]
C	11	46.30	45.09	-1.21	[45.10	45.07]
C	5	40.70	37.86	-2.84	[37.88	37.84]
C	3	38.20	38.23	0.03	[39.93	36.52]
C	16	37.30	36.23	-1.07	[36.22	36.24]
C	6	35.80	34.98	-0.82	[35.19	34.77]
C	13	31.90	30.08	-1.82	[30.07	30.10]
C	1	31.20	28.51	-2.69	[27.19	29.85]
C	15	20.70	21.03	0.33	[21.02	21.04]
C	19	20.40	19.94	-0.46	[19.94	19.95]
C	14	16.30	19.07	2.77	[19.05	19.10]
C	22	13.50	14.12	0.62	[14.18	14.05]

13C chem shifts: RMSD=1.69ppm (MAE=1.32) N=19 {-3.90 2.77}

Fractions: 0.502 0.498

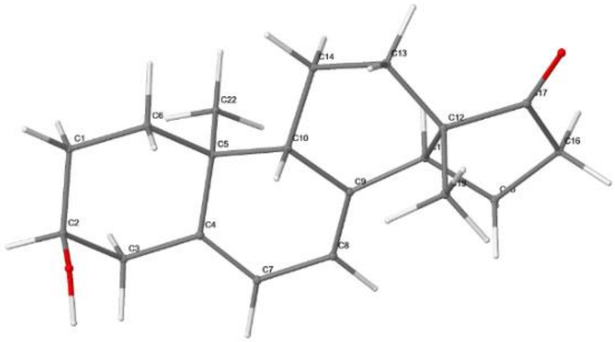

Conformer 1
Energy: -890.08927 Hartree (Rel: 0.0 kcal/mol)
XYZ coordinates for conf 1:

C	4.02135	-1.30572	-0.26509
C	4.48084	-0.05090	0.47591
C	3.75105	1.17673	-0.08813
C	2.24662	1.03229	-0.10491
C	1.65179	-0.28241	-0.63972
C	2.50125	-1.49607	-0.16310
C	1.44368	2.07539	0.19020
C	-0.00334	2.02149	0.00516
C	-0.61846	0.84124	-0.18665
C	0.20363	-0.44312	-0.07225
C	-2.07957	0.63397	-0.44973
C	-2.65002	-0.51311	0.43064
C	-1.95237	-1.85535	0.09875
C	-0.59172	-1.65196	-0.61861
C	-3.10044	1.77840	-0.31838

C	-4.45454	1.03048	-0.32565
C	-4.14049	-0.41646	0.09385
O	-4.94807	-1.32435	0.13702
C	-2.54533	-0.20082	1.94745
H	-2.16826	0.26923	-1.48902
H	0.34659	-0.61498	1.01014
C	1.65642	-0.20097	-2.18864
O	4.19782	-0.25370	1.86359
H	4.52806	-2.18265	0.15573
H	4.34102	-1.22687	-1.31202
H	5.56520	0.08481	0.33478
H	4.03450	2.08137	0.46743
H	4.12256	1.32637	-1.11490
H	2.25978	-1.69808	0.88696
H	2.21278	-2.38676	-0.73366
H	1.88535	3.01902	0.50839
H	-0.56225	2.95493	0.00225
H	-1.79358	-2.41967	1.02513
H	-2.60652	-2.47507	-0.52616
H	-0.76490	-1.51606	-1.69283
H	-0.00424	-2.57033	-0.51924
H	-3.01972	2.50406	-1.13331
H	-2.95704	2.32568	0.61955
H	-5.21406	1.46384	0.33425
H	-4.89684	0.99557	-1.32929
H	-3.04260	0.73490	2.22419
H	-3.01379	-1.00966	2.51857
H	-1.49843	-0.12442	2.25679
H	1.00696	0.60980	-2.53631
H	2.66342	-0.00923	-2.57399
H	1.30741	-1.13773	-2.63761
H	4.34868	0.58773	2.32268

Conformer 2
Energy: -890.08926 Hartree (Rel: 0.0 kcal/mol)
XYZ coordinates for conf 2:

C	4.01855	-1.28931	-0.29919
C	4.48679	-0.04254	0.46018
C	3.74733	1.19118	-0.06225
C	2.24242	1.04013	-0.08575
C	1.64918	-0.26842	-0.63772
C	2.50182	-1.48396	-0.17454
C	1.43663	2.07845	0.21747
C	-0.00983	2.02378	0.02772
C	-0.62323	0.84470	-0.17589
C	0.20122	-0.43927	-0.07328
C	-2.08383	0.63721	-0.44222
C	-2.65253	-0.52019	0.42569
C	-1.95205	-1.85740	0.07995
C	-0.59127	-1.64349	-0.63407
C	-3.10714	1.77803	-0.29885
C	-4.45974	1.02747	-0.31494
C	-4.14294	-0.42319	0.08925
O	-4.94863	-1.33327	0.12233
C	-2.54929	-0.22371	1.94579
H	-2.17193	0.28339	-1.48539
H	0.34308	-0.62238	1.00740
C	1.65344	-0.16972	-2.18560
O	4.21211	-0.13918	1.86212
H	4.53578	-2.17815	0.08919
H	4.32044	-1.19567	-1.35089
H	5.56801	0.09974	0.30506
H	4.02604	2.06934	0.53144
H	4.11680	1.37280	-1.08384
H	2.27483	-1.68315	0.87996
H	2.20426	-2.37535	-0.73899
H	1.87550	3.01799	0.55058
H	-0.57046	2.95624	0.03326
H	-1.79284	-2.43135	1.00037
H	-2.60450	-2.47171	-0.55210
H	-0.76394	-1.49571	-1.70679

H	-0.00262	-2.56224	-0.54490
H	-3.02760	2.51275	-1.10573
H	-2.96516	2.31528	0.64506
H	-5.22070	1.45220	0.34889
H	-4.90118	1.00223	-1.31926
H	-3.04948	0.70750	2.23217
H	-3.01566	-1.03993	2.50810
H	-1.50278	-0.14704	2.25633
H	0.99718	0.63979	-2.52309
H	2.65842	0.03562	-2.56904
H	1.31181	-1.10383	-2.64587
H	4.65574	-0.93722	2.19201

Revised structure of 125{7c}, i.e 128{7c-rev} ($CDCl_3$)

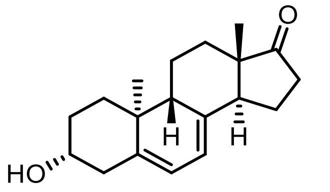

		Conf1	Conf2
Rel energy (kcal/mol):		0.00	0.00

C-nom	iGau	Exp	Calc	diff	1	2
C	17	220.00	220.34	0.34	[220.32	220.36]
C	4	141.30	140.78	-0.52	[140.68	140.89]
C	9	138.10	137.67	-0.43	[137.83	137.50]
C	7	119.10	121.56	2.46	[121.56	121.55]
C	8	117.30	117.93	0.63	[117.85	118.02]
C	2	70.10	68.98	-1.12	[69.01	68.94]
C	12	49.40	48.30	-1.10	[48.30	48.30]
C	10	47.80	47.80	0.00	[47.82	47.78]
C	11	46.30	45.06	-1.24	[45.06	45.05]
C	3	40.70	40.10	-0.60	[41.84	38.35]
C	6	38.20	38.55	0.35	[38.76	38.34]
C	5	37.30	37.83	0.53	[37.83	37.84]
C	16	35.80	36.21	0.41	[36.21	36.22]
C	1	31.90	30.43	-1.47	[28.86	32.01]
C	13	31.20	30.06	-1.14	[30.06	30.07]
C	15	20.70	21.00	0.30	[21.00	21.01]
C	19	20.40	19.92	-0.48	[19.92	19.92]
C	14	16.30	19.31	3.01	[19.28	19.34]
C	22	13.50	14.45	0.95	[14.46	14.44]

13C chem shifts: RMSD=1.16ppm (MAE=0.90) N=19 {-1.47 3.01}

Fractions: 0.501 0.499

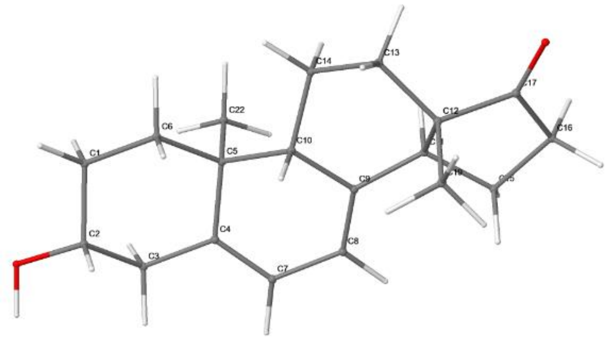

Conformer 1
Energy: -890.09035 Hartree (Rel: 0.0 kcal/mol)
XYZ coordinates for conf 1:

C	-3.92523	-1.35473	0.03471
C	-4.35733	-0.09549	-0.70603
C	-3.67192	1.12713	-0.08012
C	-2.16691	0.99962	-0.01235
C	-1.57615	-0.32388	0.50652
C	-2.40180	-1.52987	-0.02894
C	-1.36390	2.05597	-0.25718
C	0.07692	2.00898	-0.02818
C	0.69629	0.82917	0.15215
C	-0.11054	-0.45838	-0.02144
C	2.15032	0.62649	0.45454
C	2.75768	-0.49180	-0.43812
C	2.06188	-1.84803	-0.16451
C	0.67838	-1.67544	0.51605
C	3.16498	1.78190	0.38649

C	4.52433	1.04398	0.41734
C	4.23607	-0.39290	-0.05184
O	5.05202	-1.29321	-0.09473
C	2.69789	-0.13913	-1.94840
H	2.20974	0.23439	1.48587
H	-0.21813	-0.60150	-1.11239
C	-1.62756	-0.28649	2.05585
O	-5.77963	-0.00326	-0.61760
H	-4.41835	-2.22961	-0.40598
H	-4.27359	-1.28462	1.07381
H	-4.05104	-0.17949	-1.76274
H	-3.94319	2.04242	-0.62304
H	-4.08842	1.22980	0.93483
H	-2.12159	-1.70364	-1.07770
H	-2.12078	-2.43529	0.52112
H	-1.80359	3.00329	-0.56634
H	0.62701	2.94652	0.01562
H	1.93639	-2.38800	-1.11033
H	2.70187	-2.47964	0.46309
H	0.81719	-1.56761	1.59825
H	0.10253	-2.59562	0.37400
H	3.05221	2.48443	1.21766
H	3.04728	2.35344	-0.54051
H	5.30139	1.50114	-0.20494
H	4.93366	0.98445	1.43371
H	3.19641	0.80710	-2.18393
H	3.19013	-0.92884	-2.52621
H	1.66059	-0.06123	-2.28835
H	-0.99377	0.51813	2.44419
H	-2.64669	-0.11121	2.41513
H	-1.28558	-1.23326	2.48887
H	-6.05105	0.80365	-1.08422

Conformer 2
Energy: -890.09036 Hartree (Rel: 0.0 kcal/mol)
XYZ coordinates for conf 2:

C	-3.92517	-1.34457	0.04348
C	-4.35884	-0.08277	-0.70290
C	-3.67055	1.13748	-0.09133
C	-2.16638	1.00188	-0.02269
C	-1.57638	-0.31937	0.50343
C	-2.40254	-1.52700	-0.02630
C	-1.36166	2.05645	-0.26961
C	0.07883	2.00897	-0.03855
C	0.69732	0.82955	0.14675
C	-0.11043	-0.45801	-0.02242
C	2.15088	0.62700	0.45167
C	2.75841	-0.49597	-0.43492
C	2.06105	-1.85025	-0.15586
C	0.67675	-1.67319	0.52195
C	3.16655	1.78126	0.37908
C	4.52530	1.04243	0.41501
C	4.23632	-0.39660	-0.04706
O	5.05138	-1.29803	-0.08390
C	2.70091	-0.15032	-1.94691
H	2.20910	0.23985	1.48496
H	-0.21691	-0.60613	-1.11284
C	-1.62858	-0.27406	2.05255
O	-5.76598	0.14661	-0.61373
H	-4.41713	-2.22628	-0.39074
H	-4.27031	-1.26940	1.08315
H	-4.05916	-0.17232	-1.76122
H	-3.94485	2.04346	-0.64373
H	-4.08473	1.25276	0.92346
H	-2.12675	-1.70267	-1.07601
H	-2.12057	-2.43189	0.52417
H	-1.80000	3.00270	-0.58353
H	0.62964	2.94625	0.00221
H	1.93639	-2.39461	-1.09929
H	2.69964	-2.47944	0.47562
H	0.81423	-1.56019	1.60379

H	0.10045	-2.59371	0.38368
H	3.05345	2.48793	1.20668
H	3.05024	2.34836	-0.55080
H	5.30329	1.49578	-0.20890
H	4.93368	0.98777	1.43203
H	3.20112	0.79403	-2.18628
H	3.19265	-0.94346	-2.52045
H	1.66412	-0.07232	-2.28839
H	-0.99210	0.53039	2.43669
H	-2.64706	-0.09273	2.41061
H	-1.29000	-1.21969	2.49083
H	-6.21230	-0.63050	-0.98688

Originally assigned (incorrect) structure of 122{7a-1} ($CDCl_3$)

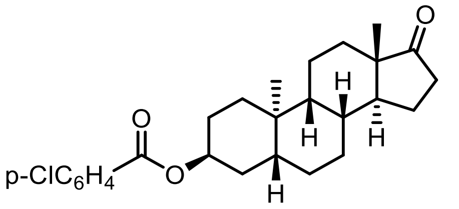

					Conf1	Conf2	Conf3	Conf4
		Rel energy (kcal/mol):			0.00	0.07	1.61	1.61

C-nom	iGau	Exp	Calc	diff	1	2	3	4
C	17	221.00	220.92	-0.08	[220.92	220.88	221.15	221.15]
C	26	165.30	163.47	-1.83	[163.45	163.48	163.53	163.49]
C	31	139.10	139.32	0.22	[139.33	139.32	139.31	139.31]
C	29	130.90	131.35	0.45	[131.35	131.35	131.34	131.34]
C	33	130.90	131.35	0.45	[131.35	131.35	131.34	131.34]
C	27	129.30	130.10	0.80	[130.10	130.09	130.17	130.14]
C	30	128.60	128.02	-0.58	[128.02	128.02	128.03	128.03]
C	32	128.60	128.02	-0.58	[128.02	128.02	128.03	128.03]
C	2	74.50	72.17	-2.33	[72.14	72.21	72.09	72.15]
C	12	54.30	49.27	-5.03	[49.36	49.34	48.16	48.20]
C	11	51.40	44.14	-7.26	[44.01	44.05	45.71	45.82]
C	10	47.80	46.40	-1.40	[46.53	46.43	45.19	45.20]
C	6	44.70	37.03	-7.67	[37.19	36.68	38.46	37.86]
C	16	36.70	35.93	-0.77	[35.89	35.93	36.17	36.21]
C	5	35.90	37.47	1.57	[37.68	37.61	34.99	35.01]
C	3	35.70	33.71	-1.99	[33.51	33.85	34.06	34.52]
C	9	35.10	35.42	0.32	[35.51	35.54	33.82	33.99]
C	4	34.00	40.36	6.36	[40.55	41.18	33.11	33.66]
C	13	31.50	31.30	-0.20	[31.34	31.34	30.70	30.72]
C	1	30.80	27.00	-3.80	[27.14	26.84	27.26	26.81]
C	7	28.30	24.67	-3.63	[24.46	24.69	25.94	26.25]
C	15	27.50	23.33	-4.17	[23.30	23.34	23.50	23.57]
C	8	21.80	27.83	6.03	[28.05	28.26	23.29	23.35]
C	14	20.50	21.33	0.83	[21.41	21.35	20.67	20.57]
C	19	13.80	12.95	-0.85	[12.51	12.44	19.64	19.62]
C	22	12.30	15.69	3.39	[15.75	15.71	15.07	15.08]

13C chem shifts: RMSD=3.35ppm (MAE=2.41) N=26 {-7.67 6.36}

Fractions: 0.495 0.440 0.033 0.033

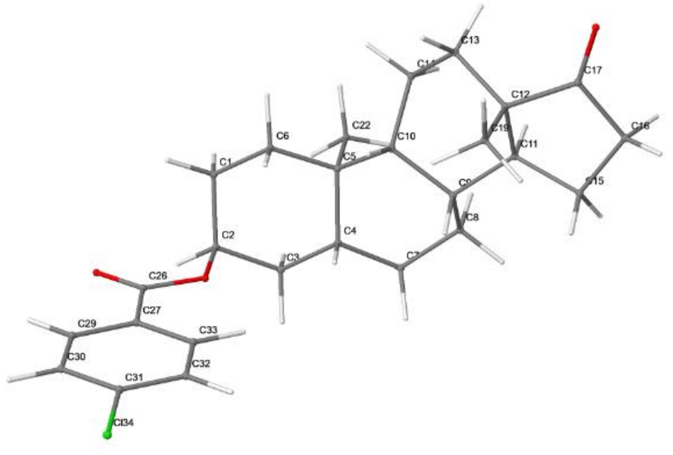

Conformer 1
Energy: -1696.52449 Hartree (Rel: 0.0 kcal/mol)
XYZ coordinates for conf 1:

C	0.58739	2.39048	-1.67795
C	1.35314	2.48872	-0.35990

C	0.44895	2.25384	0.85401
C	-0.38399	0.96780	0.71864
C	-1.26828	0.94726	-0.56956
C	-0.29825	1.13501	-1.77443
C	-1.18649	0.63776	1.98321
C	-1.73155	-0.79096	1.88658
C	-2.59427	-1.04539	0.63259
C	-1.93367	-0.48088	-0.67853
C	-4.05518	-0.58912	0.76545
C	-4.91556	-1.02502	-0.44965
C	-4.37343	-0.35651	-1.71621
C	-2.87274	-0.68261	-1.90891
C	-4.90074	-1.00210	1.99014
C	-6.33881	-0.63637	1.55048
C	-6.30664	-0.58810	0.01273
O	-7.23388	-0.25562	-0.70093
C	-5.02694	-2.56364	-0.63776
H	-4.07504	0.50950	0.75702
H	-1.07301	-1.14214	-0.85914
C	-2.28931	2.10795	-0.58520
O	2.38875	1.45373	-0.40781
H	-2.62392	-2.13696	0.50522
H	0.34213	0.14820	0.59668
C	3.51066	1.66004	0.30573
C	4.49253	0.54567	0.15669
O	3.71039	2.64680	0.99471
C	5.70186	0.63542	0.85950
C	6.65739	-0.37198	0.75955
C	6.39032	-1.47470	-0.05398
C	5.19370	-1.58648	-0.76368
C	4.24594	-0.57090	-0.65452
Cl	7.59007	-2.75319	-0.18815
H	1.30083	2.40856	-2.51030
H	-0.02071	3.29784	-1.77454
H	1.86701	3.44882	-0.27378
H	1.06863	2.20498	1.75687
H	-0.19741	3.13289	0.97370
H	0.35163	0.25226	-1.84206
H	-0.85795	1.18108	-2.71593
H	-1.99899	1.36230	2.13012
H	-0.53737	0.72392	2.86501
H	-0.87184	-1.47567	1.85789
H	-2.29873	-1.05502	2.78753
H	-4.93115	-0.68830	-2.60093
H	-4.53012	0.72535	-1.64121
H	-2.79382	-1.73457	-2.21079
H	-2.49662	-0.11211	-2.76509
H	-4.60506	-0.47540	2.90223
H	-4.80963	-2.07575	2.19308
H	-7.11026	-1.33474	1.89363
H	-6.63513	0.35679	1.91084
H	-5.39567	-3.07244	0.25891
H	-5.73002	-2.77411	-1.45134
H	-4.06658	-3.01531	-0.89963
H	-1.79459	3.08253	-0.54017
H	-2.87481	2.09581	-1.50971
H	-2.98985	2.07430	0.25224
H	5.88305	1.50365	1.48381
H	7.59398	-0.30555	1.30213
H	5.00826	-2.45202	-1.39022
H	3.31319	-0.64511	-1.20138

Conformer 2
Energy: -1696.52450 Hartree (Rel: 0.1 kcal/mol)
XYZ coordinates for conf 2:

C	0.69270	2.61563	-0.80644
C	1.35970	2.39409	0.55381
C	0.36947	1.90744	1.61145
C	-0.46398	0.70756	1.12810
C	-1.24924	1.00364	-0.18976
C	-0.18773	1.43288	-1.24674

C	-1.36126	0.12000	2.22420
C	-1.90942	-1.23506	1.76494
C	-2.68037	-1.17443	0.42955
C	-1.92014	-0.34355	-0.66887
C	-4.14255	-0.72279	0.56367
C	-4.91650	-0.84527	-0.77506
C	-4.27696	0.08190	-1.81222
C	-2.77013	-0.23237	-1.97354
C	-5.07897	-1.38366	1.59891
C	-6.47737	-0.88801	1.15861
C	-6.33283	-0.48863	-0.32057
O	-7.20175	0.02462	-0.99958
C	-5.02903	-2.29625	-1.32018
H	-4.15124	0.34852	0.80794
H	-1.05601	-0.96929	-0.93777
C	-2.25432	2.16482	-0.01229
O	2.38075	1.34965	0.43886
H	-2.71124	-2.20703	0.05368
H	0.26083	-0.08198	0.87346
C	3.60951	1.72131	0.03613
C	4.56037	0.57232	-0.02564
O	3.91918	2.86761	-0.24394
C	5.87578	0.82862	-0.43613
C	6.80889	-0.20153	-0.51315
C	6.41153	-1.49628	-0.17424
C	5.10760	-1.77628	0.23739
C	4.18375	-0.73569	0.31016
Cl	7.58145	-2.80567	-0.26801
H	1.46439	2.81344	-1.55835
H	0.09667	3.53381	-0.73541
H	1.87059	3.30367	0.87775
H	0.92312	1.63142	2.51750
H	-0.27702	2.74992	1.88765
H	0.46135	0.57301	-1.46059
H	-0.67228	1.69868	-2.19345
H	-2.17636	0.81253	2.47479
H	-0.77915	-0.01305	3.14616
H	-1.05554	-1.91620	1.63931
H	-2.54307	-1.68379	2.53971
H	-4.77382	-0.02043	-2.78503
H	-4.42560	1.12092	-1.49802
H	-2.68109	-1.18867	-2.50409
H	-2.32690	0.50896	-2.64727
H	-4.84506	-1.08931	2.62623
H	-5.01395	-2.47722	1.55323
H	-7.27898	-1.62546	1.27761
H	-6.78866	0.00347	1.71757
H	-5.47206	-2.98599	-0.59429
H	-5.66859	-2.29533	-2.20985
H	-4.05592	-2.70318	-1.60742
H	-1.75555	3.08887	0.29404
H	-2.76488	2.38209	-0.95560
H	-3.01917	1.95836	0.73974
H	6.15676	1.84417	-0.69323
H	7.82737	-0.00639	-0.83041
H	4.82165	-2.78987	0.49619
H	3.16829	-0.93986	0.62918

Conformer 3
Energy: -1696.52695 Hartree (Rel: 1.6 kcal/mol)
XYZ coordinates for conf 3:

C	-0.75870	-2.87373	-1.73957
C	-1.56624	-2.85368	-0.44347
C	-0.66088	-2.88086	0.78993
C	0.45377	-1.81901	0.75991
C	1.30705	-1.85082	-0.54314
C	0.33617	-1.79570	-1.75688
C	1.31610	-1.90635	2.03863
C	2.66713	-1.16803	1.91770
C	2.63261	-0.04338	0.87066
C	2.18919	-0.54606	-0.56058

C	3.98698	0.67349	0.77554
C	4.10239	1.60482	-0.45667
C	4.04227	0.77840	-1.76368
C	3.38630	-0.60605	-1.53763
C	4.48177	1.53283	1.95852
C	5.66473	2.31951	1.34885
C	5.43281	2.30828	-0.17082
O	6.19049	2.77577	-0.99953
C	3.04110	2.73930	-0.47161
H	4.75285	-0.10639	0.63204
H	1.52245	0.23422	-0.95239
C	2.15370	-3.14262	-0.63777
O	-2.34946	-1.61650	-0.46153
H	1.88447	0.68834	1.20816
H	-0.04498	-0.83828	0.76340
C	-3.50148	-1.59841	0.23329
C	-4.21404	-0.29170	0.12021
O	-3.92340	-2.54213	0.88113
C	-5.42306	-0.13930	0.81276
C	-6.13414	1.05552	0.74504
C	-5.62210	2.10091	-0.02578
C	-4.42078	1.97287	-0.72475
C	-3.71904	0.77153	-0.64798
Cl	-6.51167	3.61476	-0.11856
H	-1.43518	-2.73875	-2.59183
H	-0.32205	-3.87467	-1.84319
H	-2.27857	-3.68125	-0.40779
H	-1.27220	-2.75721	1.69158
H	-0.22335	-3.88620	0.85460
H	-0.15419	-0.81364	-1.76868
H	0.90301	-1.87050	-2.69367
H	0.73406	-1.49355	2.87272
H	1.49737	-2.95933	2.29162
H	2.95216	-0.75346	2.89234
H	3.46421	-1.87433	1.65248
H	3.47406	1.33181	-2.52110
H	5.05078	0.64904	-2.17475
H	3.06069	-1.00138	-2.50704
H	4.13716	-1.31238	-1.16362
H	4.78650	0.92883	2.81823
H	3.69411	2.21283	2.30406
H	5.76449	3.34760	1.71423
H	6.62599	1.82609	1.54111
H	3.04666	3.34044	0.44397
H	3.24319	3.41139	-1.31294
H	2.03267	2.33455	-0.59935
H	1.53362	-4.03921	-0.53819
H	2.65664	-3.20915	-1.60824
H	2.92495	-3.19954	0.13476
H	-5.79776	-0.96812	1.40360
H	-7.06969	1.17655	1.27995
H	-4.04281	2.79810	-1.31824
H	-2.78559	0.65898	-1.18709

Conformer 4
Energy: -1696.52707 Hartree (Rel: 1.6 kcal/mol)
XYZ coordinates for conf 4:

C	-0.89227	-2.95552	-1.23432
C	-1.57351	-2.85485	0.13262
C	-0.55583	-2.81782	1.27028
C	0.55647	-1.77011	1.07498
C	1.27698	-1.87649	-0.30196
C	0.19089	-1.88186	-1.41554
C	1.54020	-1.79953	2.26522
C	2.87255	-1.07402	1.97525
C	2.73521	-0.00546	0.87886
C	2.15305	-0.57924	-0.47402
C	4.07352	0.69885	0.61392
C	4.06632	1.56508	-0.66988
C	3.87822	0.67245	-1.92011
C	3.24869	-0.69553	-1.55900

C	4.68258	1.61497	1.69651
C	5.80034	2.36199	0.93339
C	5.41785	2.27665	-0.55314
O	6.08836	2.69989	-1.47542
C	3.00815	2.70228	-0.63894
H	4.82163	-0.09100	0.43569
H	1.45096	0.18306	-0.83856
C	2.11065	-3.17565	-0.41002
O	-2.33816	-1.60876	0.21619
H	2.02406	0.74691	1.24953
H	0.06498	-0.78580	1.07652
C	-3.59026	-1.61177	-0.27670
C	-4.26592	-0.28972	-0.12289
O	-4.12024	-2.58377	-0.78905
C	-5.57322	-0.15822	-0.61093
C	-6.25552	1.04976	-0.49657
C	-5.61473	2.12965	0.11364
C	-4.31369	2.02305	0.60763
C	-3.64222	0.80816	0.48635
Cl	-6.46670	3.66042	0.26369
H	-1.64689	-2.87533	-2.02424
H	-0.46355	-3.96210	-1.31825
H	-2.28328	-3.67452	0.26693
H	-1.07725	-2.63126	2.21772
H	-0.11798	-3.82138	1.35222
H	-0.30149	-0.90071	-1.42946
H	0.66304	-2.00563	-2.39844
H	1.04336	-1.34401	3.13166
H	1.74563	-2.83995	2.54991
H	3.25066	-0.61016	2.89443
H	3.64076	-1.79663	1.67155
H	3.23764	1.18843	-2.64550
H	4.84137	0.51751	-2.42141
H	2.83001	-1.13920	-2.46995
H	4.03291	-1.38487	-1.22362
H	5.06975	1.05442	2.55249
H	3.93314	2.31662	2.08159
H	5.93888	3.40609	1.23507
H	6.77460	1.87116	1.05335
H	3.10357	3.34932	0.23974
H	3.12647	3.32955	-1.52936
H	1.99211	2.29642	-0.64656
H	1.50513	-4.06241	-0.19742
H	2.51118	-3.29852	-1.42178
H	2.95749	-3.19314	0.28089
H	-6.04676	-1.01367	-1.08034
H	-7.26689	1.15467	-0.87358
H	-3.83603	2.87527	1.07843
H	-2.63190	0.71222	0.86666

Revised structure of 122{7a-1}, i.e 129{7a-1-rev} ($CDCl_3$)

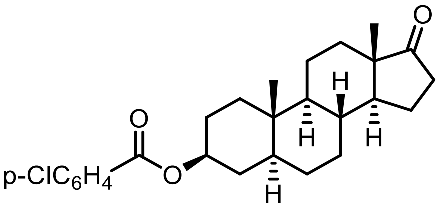

					Conf1	Conf2
		Rel energy (kcal/mol):			0.00	0.02

C-nom	iGau	Exp	Calc	diff	1	2
C	17	221.00	220.45	-0.55	[220.49	220.40]
C	26	165.30	163.59	-1.71	[163.59	163.59]
C	31	139.10	139.30	0.20	[139.27	139.34]
C	29	130.90	131.35	0.45	[131.36	131.33]
C	33	130.90	131.35	0.45	[131.36	131.33]
C	27	129.30	129.89	0.59	[129.89	129.88]
C	30	128.60	127.97	-0.63	[127.97	127.97]
C	32	128.60	127.97	-0.63	[127.97	127.97]
C	2	74.50	74.32	-0.18	[74.36	74.28]
C	10	54.30	53.81	-0.49	[53.81	53.80]
C	11	51.40	50.75	-0.65	[50.74	50.77]
C	12	47.80	48.94	1.14	[48.91	48.98]
C	4	44.70	43.87	-0.83	[43.74	44.00]
C	6	36.70	38.08	1.38	[38.13	38.02]
C	16	35.90	36.26	0.36	[36.29	36.22]
C	5	35.70	36.00	0.30	[35.99	36.02]
C	9	35.10	35.02	-0.08	[35.03	35.00]
C	3	34.00	34.44	0.44	[34.50	34.38]
C	13	31.50	33.51	2.01	[33.51	33.50]
C	8	30.80	31.79	0.99	[31.79	31.79]
C	7	28.30	29.07	0.77	[28.98	29.16]
C	1	27.50	28.01	0.51	[27.89	28.14]
C	15	21.80	22.60	0.80	[22.60	22.61]
C	14	20.50	22.47	1.97	[22.46	22.48]
C	19	13.80	13.45	-0.35	[13.43	13.47]
C	22	12.30	12.47	0.17	[12.50	12.44]

13C chem shifts: RMSD=0.88ppm (MAE=0.72) N=26 {-1.71 2.01}

Fractions: 0.510 0.490

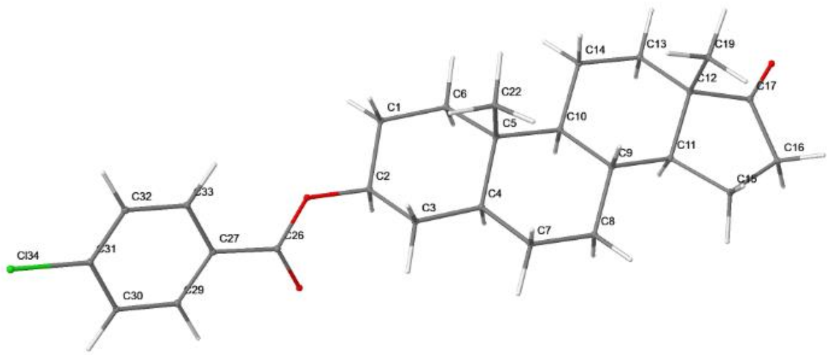

Conformer 1
Energy: -1696.54006 Hartree (Rel: 0.0 kcal/mol)
XYZ coordinates for conf 1:

C	0.89216	-1.57817	-0.65499
C	1.50143	-0.18090	-0.66223
C	0.82090	0.74431	0.34582
C	-0.69652	0.79829	0.08893
C	-1.37803	-0.60898	0.14381

C	-0.63139	-1.51979	-0.86984
C	-1.40275	1.83052	0.97877
C	-2.87510	1.98266	0.57828
C	-3.62499	0.63942	0.56158
C	-2.87110	-0.42102	-0.30287
C	-5.04770	0.78822	0.00822
C	-5.84440	-0.53966	0.01194
C	-5.12857	-1.55574	-0.88353
C	-3.66709	-1.74626	-0.42316
C	-6.02997	1.82261	0.59867
C	-7.37682	1.41811	-0.04714
C	-7.21634	-0.05560	-0.46143
O	-8.04490	-0.71251	-1.06248
C	-6.09443	-1.12416	1.42924
H	-4.93341	1.06115	-1.05608
H	-2.82378	0.00490	-1.31991
C	-1.29403	-1.24122	1.55290
O	2.91522	-0.33158	-0.33962
H	-3.68917	0.27783	1.59749
H	-0.82152	1.14798	-0.94985
C	3.77062	0.58833	-0.82341
C	5.18239	0.31460	-0.42444
O	3.43594	1.53736	-1.51286
C	6.17516	1.20040	-0.86516
C	7.50963	0.99656	-0.52533
C	7.84244	-0.10633	0.26339
C	6.87165	-1.00200	0.71445
C	5.53972	-0.78633	0.36669
Cl	9.52492	-0.37341	0.69922
H	1.35614	-2.18699	-1.44018
H	1.12740	-2.06333	0.30032
H	1.43924	0.25965	-1.66191
H	1.03880	0.39373	1.36257
H	1.24331	1.75180	0.25645
H	-1.02411	-2.54155	-0.82625
H	-0.83267	-1.15258	-1.88707
H	-0.89192	2.79858	0.89066
H	-1.32553	1.53805	2.03498
H	-3.38002	2.68272	1.25589
H	-2.92628	2.43047	-0.42624
H	-5.65310	-2.51912	-0.88282
H	-5.14203	-1.19145	-1.92004
H	-3.66030	-2.27443	0.53771
H	-3.16708	-2.41113	-1.13587
H	-5.74990	2.85235	0.35858
H	-6.07770	1.74618	1.69132
H	-8.24909	1.53874	0.60482
H	-7.58306	1.99920	-0.95494
H	-6.61362	-0.41992	2.08768
H	-6.71874	-2.02058	1.34466
H	-5.16163	-1.40753	1.92383
H	-0.26468	-1.29140	1.91946
H	-1.67727	-2.26731	1.54078
H	-1.87099	-0.68452	2.29689
H	5.88807	2.04905	-1.47660
H	8.28031	1.67986	-0.86470
H	7.15363	-1.85196	1.32611
H	4.77566	-1.47397	0.71008

Conformer 2
Energy: -1696.54002 Hartree (Rel: 0.0 kcal/mol)
XYZ coordinates for conf 2:

C	0.96255	-1.35536	0.37016
C	1.50145	-0.21961	-0.49741
C	0.75491	1.08767	-0.25417
C	-0.75738	0.89313	-0.47024
C	-1.36901	-0.22059	0.44321
C	-0.55744	-1.52050	0.18678
C	-1.53293	2.21514	-0.38708
C	-3.00211	2.01408	-0.77750
C	-3.68450	0.90371	0.04018

C	-2.85995	-0.42229	-0.00403
C	-5.09949	0.60653	-0.47222
C	-5.83086	-0.48099	0.35283
C	-5.04450	-1.79255	0.26126
C	-3.58645	-1.58247	0.72382
C	-6.14479	1.73154	-0.62952
C	-7.45361	0.94483	-0.87968
C	-7.21239	-0.46255	-0.30476
O	-7.99334	-1.39280	-0.36904
C	-6.08761	-0.08698	1.83324
H	-4.97284	0.17600	-1.48162
H	-2.81256	-0.70638	-1.06942
C	-1.28200	0.14568	1.94323
O	2.90585	0.02370	-0.19068
H	-3.75462	1.24862	1.08150
H	-0.87760	0.52739	-1.50406
C	3.82771	-0.70746	-0.84467
C	5.22053	-0.35568	-0.44013
O	3.56110	-1.55883	-1.67656
C	6.27757	-1.04490	-1.05004
C	7.59806	-0.76021	-0.71372
C	7.85164	0.22437	0.24310
C	6.81587	0.92372	0.86454
C	5.49877	0.62903	0.51826
Cl	9.51585	0.59208	0.67588
H	1.47175	-2.28946	0.10868
H	1.20394	-1.14124	1.41861
H	1.44069	-0.50022	-1.55325
H	0.96574	1.44543	0.76159
H	1.12889	1.85563	-0.94285
H	-0.89911	-2.32168	0.85130
H	-0.75593	-1.86288	-0.83961
H	-1.06924	2.95342	-1.05493
H	-1.46375	2.63289	0.62668
H	-3.55503	2.95497	-0.66254
H	-3.05377	1.75153	-1.84552
H	-5.51949	-2.57930	0.86001
H	-5.05192	-2.14260	-0.78021
H	-3.57567	-1.40875	1.80646
H	-3.03803	-2.51699	0.56322
H	-5.90862	2.41216	-1.45259
H	-6.21640	2.33718	0.28154
H	-8.34728	1.39264	-0.43110
H	-7.66357	0.83579	-1.95129
H	-6.65702	0.84367	1.92712
H	-6.66494	-0.87938	2.32240
H	-5.15455	0.04260	2.38778
H	-0.25829	0.37952	2.24941
H	-1.61646	-0.69085	2.56621
H	-1.89806	1.01237	2.19939
H	6.05107	-1.80501	-1.78986
H	8.41833	-1.29150	-1.18372
H	7.03685	1.68436	1.60526
H	4.68479	1.16429	0.99307

Originally assigned (incorrect) structure of 131{26} (DMSO-d_6)

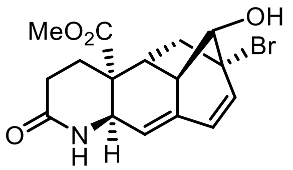

	Conf1	Conf2
Rel energy (kcal/mol):	0.00	0.76

C-nom	iGau	Exp	Calc	diff	1	2
C	19	172.10	174.35	2.25	[174.55	173.62]
C	2	167.60	174.75	7.15	[174.70	174.95]
C	8	136.10	143.45	7.35	[143.61	142.88]
C	12	131.50	135.61	4.11	[135.64	135.49]
C	11	128.00	129.69	1.69	[129.64	129.88]
C	7	107.50	121.69	14.19	[121.52	122.29]
C	14	78.60	78.81	0.21	[78.81	78.83]
C	13	65.80	65.44	-0.36	[65.44	65.42]
C	22	51.90	52.15	0.25	[52.06	52.46]
C	4	47.90	52.65	4.75	[52.69	52.52]
C	5	47.10	50.83	3.73	[50.87	50.67]
C	10	39.90	48.08	8.18	[47.86	48.85]
C	15	39.20	46.76	7.56	[47.08	45.60]
C	9	32.70	41.83	9.13	[41.84	41.79]
C	6	31.50	34.04	2.54	[34.12	33.74]
C	1	29.80	29.91	0.11	[29.89	29.97]

13C chem shifts: RMSD=6.03ppm (MAE=4.60) N=16 {-0.36 14.19}

Fractions: 0.783 0.217

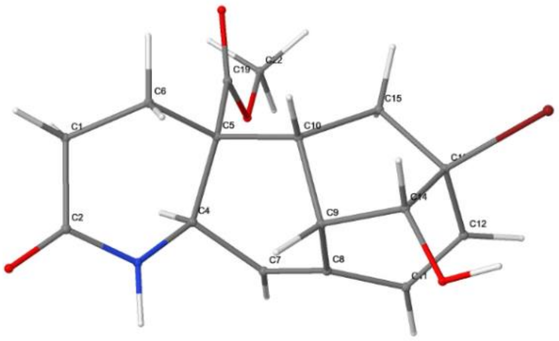

Conformer 1
Energy: -3547.54650 Hartree (Rel: 0.0 kcal/mol)
XYZ coordinates for conf 1:

C	4.01374	0.03140	-1.09427
C	4.00635	-1.40343	-0.59290
N	2.95086	-1.65704	0.23602
C	2.21566	-0.57111	0.90860
C	1.64174	0.46078	-0.15250
C	2.57256	0.46462	-1.40790
C	1.12067	-1.10119	1.79559
C	-0.07223	-1.41731	1.26343
C	-0.19706	-1.34690	-0.26017
C	0.17740	0.11043	-0.61866
C	-1.30772	-1.37886	2.05648
C	-2.34764	-0.71496	1.52695
C	-2.18999	-0.21237	0.10689
C	-1.64708	-1.38719	-0.74667
C	-1.07600	0.88728	-0.06150
O	4.81381	-2.26609	-0.93616
Br	-3.96005	0.40827	-0.57769
O	-2.25635	-2.64052	-0.55591
C	1.63918	1.84636	0.50830

O	1.87352	2.07330	1.68054
O	1.30271	2.80741	-0.36993
C	1.22627	4.14995	0.15227
H	4.64462	0.07995	-1.98525
H	4.46773	0.68463	-0.33575
H	2.92646	-2.57273	0.67030
H	2.91383	-0.01347	1.53933
H	2.16639	-0.23934	-2.14246
H	2.55496	1.45076	-1.87713
H	1.25570	-1.03411	2.87181
H	0.43064	-2.09383	-0.75280
H	0.15866	0.22416	-1.70638
H	-1.31295	-1.73552	3.08317
H	-3.26705	-0.53144	2.07145
H	-1.67391	-1.10644	-1.80841
H	-1.38938	1.66456	-0.75920
H	-0.88881	1.36140	0.90560
H	-3.17850	-2.56725	-0.85582
H	0.94634	4.77466	-0.69504
H	0.47099	4.20908	0.93933
H	2.19533	4.45679	0.55217

Conformer 2
Energy: -3547.54771 Hartree (Rel: 0.8 kcal/mol)
XYZ coordinates for conf 2:

C	3.96568	-0.30749	-1.27851
C	3.98109	-1.52380	-0.36795
N	2.94647	-1.51803	0.52482
C	2.22172	-0.28141	0.87065
C	1.62708	0.40167	-0.43897
C	2.51326	0.01787	-1.66029
C	1.14504	-0.53915	1.89210
C	-0.05753	-0.98851	1.49526
C	-0.21657	-1.34906	0.01679
C	0.14760	-0.05444	-0.74594
C	-1.27560	-0.72002	2.27069
C	-2.32695	-0.22568	1.59747
C	-2.20188	-0.14553	0.09007
C	-1.67712	-1.51669	-0.40790
C	-1.09289	0.85642	-0.40574
O	4.78615	-2.45016	-0.45454
Br	-3.98743	0.26465	-0.70351
O	-2.28230	-2.66128	0.14190
C	1.63030	1.93224	-0.29546
O	1.64717	2.70402	-1.23572
O	1.55229	2.34145	0.98235
C	1.50336	3.76922	1.19162
H	4.56818	-0.53318	-2.16188
H	4.44114	0.54190	-0.76779
H	2.94235	-2.26246	1.21310
H	2.93573	0.42390	1.30714
H	2.08886	-0.87092	-2.13914
H	2.47070	0.82683	-2.39322
H	1.30087	-0.17094	2.90244
H	0.40080	-2.20767	-0.25949
H	0.10442	-0.25118	-1.82113
H	-1.25860	-0.77211	3.35620
H	-3.23398	0.10974	2.08765
H	-1.72712	-1.54739	-1.50486
H	-1.42259	1.40508	-1.28930
H	-0.88506	1.58182	0.38380
H	-3.21037	-2.67061	-0.14857
H	1.44208	3.90098	2.27110
H	2.40657	4.23944	0.79672
H	0.62500	4.19421	0.70097

Revised structure of 131{26}, i.e enamide 132{26-rev} (DMSO-d_6)

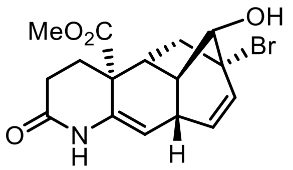

					Conf1	Conf2
Rel energy (kcal/mol):					0.00	0.07

C-nom	iGau	Exp	Calc	diff	1	2
C	19	172.10	172.20	0.10	[172.54	171.81]
C	2	167.60	167.52	-0.08	[167.65	167.38]
C	4	136.10	136.94	0.84	[136.85	137.05]
C	12	131.50	131.67	0.17	[131.46	131.91]
C	11	128.00	129.25	1.25	[129.33	129.17]
C	7	107.50	107.96	0.46	[107.76	108.18]
C	14	78.60	77.07	-1.53	[76.97	77.18]
C	13	65.80	67.07	1.27	[67.14	66.99]
C	22	51.90	52.37	0.47	[52.33	52.42]
C	5	47.90	46.51	-1.39	[46.16	46.91]
C	15	47.10	47.06	-0.04	[46.67	47.51]
C	9	39.90	40.14	0.24	[40.20	40.07]
C	10	39.20	40.37	1.17	[40.73	39.96]
C	8	32.70	32.66	-0.04	[32.69	32.63]
C	6	31.50	32.55	1.05	[32.83	32.24]
C	1	29.80	28.80	-1.00	[28.68	28.94]

13C chem shifts: RMSD=0.87ppm (MAE=0.69) N=16 {-1.53 1.27}

Fractions: 0.531 0.469

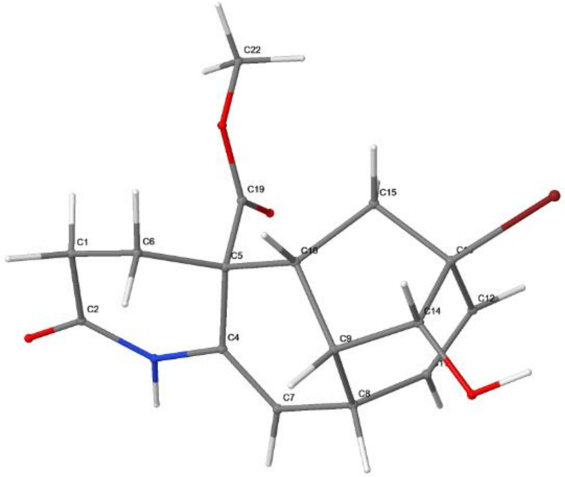


```
Conformer 1
Energy: -3547.57027 Hartree (Rel:  0.0 kcal/mol)
XYZ coordinates for conf 1:
C          3.90472        0.09881       -1.49094
C          4.37775       -0.52769       -0.19037
N          3.40062       -0.94365        0.67945
C          2.00356       -0.97079        0.46474
C          1.50821       -0.00069       -0.59338
C          2.43259       -0.16285       -1.83020
C          1.23039       -1.81858        1.15600
C         -0.25777       -1.93782        0.93671
C         -0.58630       -1.61416       -0.53873
C          0.01101       -0.26104       -1.02086
C         -1.04239       -1.06676        1.89930
C         -1.95000       -0.17873        1.48604
C         -2.19955       -0.01231        0.00687
```

C	-2.09027	-1.36627	-0.72270
C	-1.08483	0.81897	-0.65821
O	5.56810	-0.64226	0.09514
Br	-4.01454	0.78715	-0.27624
O	-2.86381	-2.42096	-0.20205
C	1.63790	1.46192	-0.11424
O	1.75619	2.40548	-0.87263
O	1.56553	1.58839	1.21745
C	1.65494	2.93458	1.73317
H	4.56441	-0.26627	-2.28397
H	4.08909	1.17732	-1.41063
H	3.75289	-1.41934	1.50415
H	1.68931	-2.47427	1.89374
H	2.30527	-1.18252	-2.21145
H	2.10809	0.52685	-2.61368
H	-0.23417	-2.44011	-1.16415
H	0.03154	-0.30199	-2.11323
H	-0.82459	-1.17685	2.95980
H	-2.47581	0.47058	2.17926
H	-2.31271	-1.21113	-1.78894
H	-1.44173	1.30489	-1.56971
H	-0.74565	1.60174	0.02058
H	-3.79765	-2.15827	-0.27275
H	1.59097	2.83364	2.81574
H	2.60593	3.38677	1.44330
H	0.83041	3.54278	1.35435
H	-0.53892	-2.98319	1.12730

Conformer 2
Energy: -3547.57039 Hartree (Rel: 0.1 kcal/mol)
XYZ coordinates for conf 2:

C	3.94064	0.32585	-1.20838
C	4.36357	-0.61443	-0.09150
N	3.35448	-1.23141	0.60346
C	1.96560	-1.17165	0.34891
C	1.51985	0.04929	-0.43782
C	2.48146	0.17265	-1.65284
C	1.15905	-2.15496	0.76846
C	-0.32093	-2.18898	0.47657
C	-0.60081	-1.46574	-0.86145
C	0.02850	-0.04598	-0.93922
C	-1.13106	-1.61292	1.62225
C	-2.01615	-0.62824	1.45027
C	-2.20699	-0.04495	0.07148
C	-2.09431	-1.14175	-1.00688
C	-1.05059	0.90970	-0.29020
O	5.54266	-0.80960	0.19729
Br	-3.99513	0.85161	-0.03129
O	-2.89942	-2.28220	-0.82424
C	1.66508	1.29315	0.46406
O	1.73487	1.27934	1.67622
O	1.66343	2.42301	-0.26485
C	1.74007	3.65861	0.47718
H	4.62821	0.16354	-2.04403
H	4.12810	1.34840	-0.85519
H	3.67376	-1.90554	1.29197
H	1.58443	-2.98931	1.32314
H	2.36437	-0.73008	-2.26370
H	2.19032	1.02584	-2.27020
H	-0.24340	-2.09657	-1.68108
H	0.06218	0.22294	-1.99857
H	-0.95129	-2.02432	2.61351
H	-2.56156	-0.19256	2.28169
H	-2.27998	-0.68858	-1.99199
H	-1.36441	1.66428	-1.01538
H	-0.70967	1.43047	0.60577
H	-3.82659	-1.98878	-0.83889
H	1.73994	4.44765	-0.27368
H	0.87637	3.75784	1.13889
H	2.65825	3.68870	1.06784
H	-0.61113	-3.24281	0.36031

Originally assigned (incorrect) structure 133{3a} ($CDCl_3$)

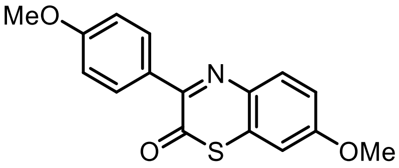

	Conf1	Conf2
Rel energy (kcal/mol):	0.00	0.40

C-nom	iGau	Exp	Calc	diff	1	2
C-C	9	182.26	177.76	-4.50	[177.65	177.96]
C-C	15	164.39	160.34	-4.05	[160.36	160.31]
C-C	4	163.21	159.93	-3.28	[159.94	159.92]
C-C	8	158.55	145.83	-12.72	[145.69	146.10]
C-C	2	147.51	132.36	-15.15	[132.70	131.69]
C-C	1	137.90	128.58	-9.32	[128.60	128.53]
C-C	12	126.93	127.74	0.81	[127.73	127.76]
C-CH	17	132.69	132.08	-0.61	[132.08	132.09]
C-CH	13	132.69	132.08	-0.61	[132.08	132.09]
C-CH	6	125.24	135.66	10.42	[135.40	136.16]
C-CH	5	116.41	114.69	-1.72	[117.14	109.91]
C-CH	14	112.80	113.44	0.64	[113.45	113.42]
C-CH	16	112.80	113.44	0.64	[113.45	113.42]
C-CH	3	102.38	107.82	5.44	[105.61	112.13]
C-CH3	19	54.82	56.16	1.34	[56.17	56.13]
C-CH3	21	54.53	55.42	0.89	[55.41	55.43]

13C chem shifts: RMSD=6.48ppm (MAE=4.51) N=16 {-15.15 10.42}

Fractions: 0.661 0.339

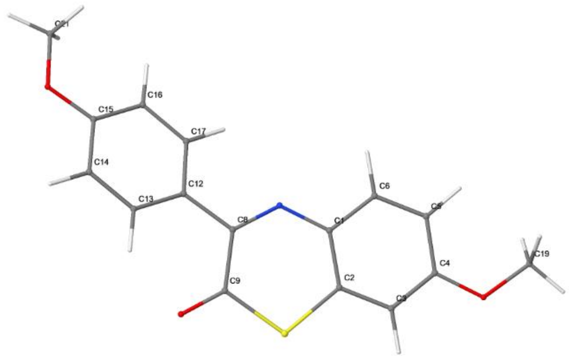

Conformer 1
Energy: -1296.11009 Hartree (Rel: 0.0 kcal/mol)
XYZ coordinates for conf 1:

C	1.58174	-0.67049	-0.06000
C	2.45335	0.43699	0.02918
C	3.84550	0.27625	0.02545
C	4.38786	-1.00811	-0.06317
C	3.53477	-2.13161	-0.15047
C	2.16845	-1.95882	-0.15010
N	0.21024	-0.61091	-0.08421
C	-0.53086	0.45870	-0.00936
C	0.00832	1.84390	0.13929
S	1.79792	2.06257	0.15204
O	-0.65442	2.85905	0.26455
C	-2.00441	0.26686	-0.02936
C	-2.92086	1.25427	-0.44999
C	-4.28327	0.99323	-0.48946
C	-4.78332	-0.25906	-0.09962
C	-3.88875	-1.25315	0.32492
C	-2.52362	-0.98378	0.34987
O	5.71422	-1.28064	-0.07224
C	6.63880	-0.19680	0.01206

O	-6.13305	-0.40924	-0.16582
C	-6.69643	-1.66231	0.21156
H	4.48287	1.14994	0.09432
H	3.98188	-3.11775	-0.21867
H	1.49790	-2.80925	-0.22077
H	-2.56492	2.22942	-0.75248
H	-4.98460	1.75187	-0.82301
H	-4.24342	-2.22801	0.63883
H	-1.83391	-1.75612	0.67214
H	7.62980	-0.65107	-0.01148
H	6.52822	0.48532	-0.83898
H	6.51291	0.35944	0.94832
H	-7.77381	-1.55691	0.07644
H	-6.32678	-2.47465	-0.42584
H	-6.48224	-1.89684	1.26132

Conformer 2
Energy: -1296.11072 Hartree (Rel: 0.4 kcal/mol)
XYZ coordinates for conf 2:

C	1.62982	-0.44470	-0.03730
C	2.42816	0.72334	0.04455
C	3.81883	0.64572	0.04301
C	4.45209	-0.60118	-0.03474
C	3.68070	-1.77854	-0.11264
C	2.29897	-1.68592	-0.11487
N	0.25643	-0.47655	-0.06659
C	-0.55097	0.54371	-0.00232
C	-0.10306	1.96327	0.14159
S	1.66596	2.30461	0.15471
O	-0.83511	2.92996	0.26292
C	-2.00934	0.25939	-0.02870
C	-2.98161	1.17965	-0.47517
C	-4.32447	0.83278	-0.52147
C	-4.74862	-0.44140	-0.11282
C	-3.79772	-1.36956	0.33766
C	-2.45238	-1.01438	0.36906
O	5.80667	-0.57017	-0.02762
C	6.52257	-1.80249	-0.10495
O	-6.08559	-0.67724	-0.18794
C	-6.57315	-1.95563	0.20931
H	4.43205	1.53937	0.10433
H	4.15303	-2.75136	-0.17292
H	1.68622	-2.57945	-0.17920
H	-2.68459	2.16963	-0.79283
H	-5.06881	1.53962	-0.87500
H	-4.09363	-2.35900	0.66655
H	-1.71852	-1.73568	0.71156
H	7.57855	-1.53120	-0.08351
H	6.29395	-2.44920	0.75006
H	6.30043	-2.33322	-1.03799
H	-7.65372	-1.92040	0.06367
H	-6.14774	-2.75528	-0.40912
H	-6.35419	-2.15633	1.26510

Revised structure of 133{3a}, i.e. 140{3a-rev} ($CDCl_3$)

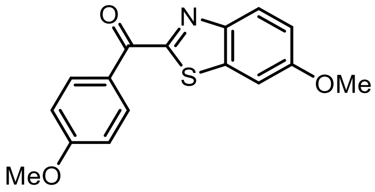

	Conf1	Conf2
Rel energy (kcal/mol):	0.00	0.70

C-nom	iGau	Exp	Calc	diff	1	2
C-C	9	182.26	184.03	1.77	[184.00	184.12]
C-C	8	164.39	165.76	1.37	[165.68	166.02]
C-C	15	163.21	163.88	0.67	[163.89	163.83]
C-C	4	158.55	159.10	0.55	[159.15	158.94]
C-C	1	147.51	148.09	0.58	[148.01	148.35]
C-C	2	137.90	138.87	0.97	[139.06	138.24]
C-C	12	126.93	128.15	1.22	[128.14	128.19]
C-CH	13	132.69	133.51	0.82	[133.51	133.52]
C-CH	17	132.69	133.51	0.82	[133.51	133.52]
C-CH	6	125.24	127.25	2.01	[127.15	127.56]
C-CH	5	116.41	116.30	-0.11	[118.05	110.58]
C-CH	14	112.80	113.20	0.40	[113.20	113.18]
C-CH	16	112.80	113.20	0.40	[113.20	113.18]
C-CH	3	102.38	103.31	0.93	[101.75	108.42]
C-CH3	19	54.82	56.03	1.21	[56.02	56.06]
C-CH3	21	54.53	55.99	1.46	[56.02	55.90]

13C chem shifts: RMSD=1.08ppm (MAE=0.95) N=16 {-0.11 2.01}

Fractions: 0.766 0.234

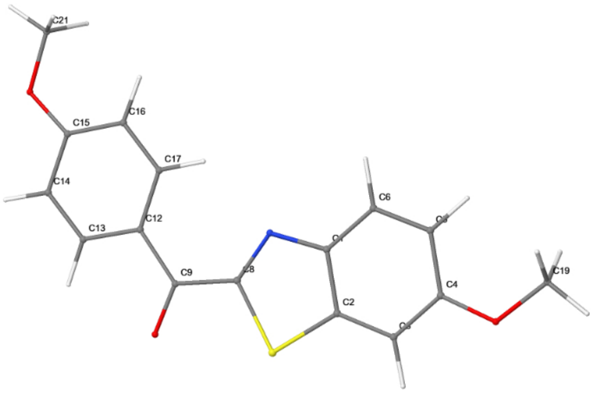

Conformer 1
Energy: -1296.12305 Hartree (Rel: 0.0 kcal/mol)
XYZ coordinates for conf 1:

C	-1.83687	-0.70718	-0.00252
C	-2.66255	0.44545	0.00109
C	-4.05942	0.35385	0.00181
C	-4.62559	-0.92322	-0.00120
C	-3.80923	-2.08372	-0.00485
C	-2.43321	-1.98423	-0.00553
N	-0.48249	-0.45859	-0.00287
C	-0.23212	0.81673	0.00037
C	1.08365	1.52396	0.00034
S	-1.66464	1.87735	0.00393
O	1.03564	2.75802	0.00061
C	2.37936	0.80532	-0.00036
C	3.54855	1.59871	-0.00675
C	4.80408	1.02223	-0.00724
C	4.93520	-0.38035	-0.00093
C	3.78558	-1.18453	0.00569

```
C          2.52426       -0.59227        0.00582
O         -5.96154       -1.16768       -0.00095
C         -6.85747       -0.05907        0.00252
O          6.20520       -0.85095       -0.00183
C          6.41399       -2.26259        0.00483
H         -4.67177        1.24684        0.00460
H         -4.30272       -3.05004       -0.00710
H         -1.80586       -2.86988       -0.00835
H          3.44113        2.67781       -0.01141
H          5.70450        1.62843       -0.01237
H          3.86242       -2.26538        0.01074
H          1.64274       -1.22039        0.01058
H         -7.86036       -0.48770        0.00208
H         -6.72351        0.55735        0.89954
H         -6.72471        0.56198       -0.89149
H          7.49568       -2.40151        0.00265
H          5.98762       -2.72263        0.90381
H          5.98284       -2.73185       -0.88708

Conformer 2
Energy: -1296.12417 Hartree (Rel:  0.7 kcal/mol)
XYZ coordinates for conf 2:
C          1.88113       -0.46439        0.00018
C          2.63578        0.74131       -0.00009
C          4.02733        0.72564       -0.00021
C          4.67821       -0.51458       -0.00001
C          3.93982       -1.72160        0.00026
C          2.55269       -1.69643        0.00036
N          0.51270       -0.29888        0.00025
C          0.18546        0.95782        0.00006
C         -1.17340        1.58082       -0.00007
S          1.54829        2.10949       -0.00028
O         -1.20248        2.81528       -0.00023
C         -2.42109        0.78194        0.00003
C         -3.63842        1.49945        0.00060
C         -4.85480        0.84434        0.00066
C         -4.89665       -0.56378        0.00010
C         -3.69821       -1.29330       -0.00052
C         -2.47717       -0.62211       -0.00055
O          6.03642       -0.45781       -0.00010
C          6.77785       -1.67519        0.00015
O         -6.13406       -1.11415        0.00018
C         -6.25285       -2.53625       -0.00031
H          4.62017        1.63383       -0.00044
H          4.45212       -2.67587        0.00039
H          1.98072       -2.61891        0.00053
H         -3.60020        2.58321        0.00102
H         -5.79188        1.39217        0.00114
H         -3.70613       -2.37685       -0.00100
H         -1.55753       -1.19284       -0.00104
H          7.82808       -1.38063        0.00009
H          6.56722       -2.27154        0.89592
H          6.56723       -2.27191       -0.89537
H         -7.32355       -2.74347       -0.00003
H         -5.79574       -2.97243       -0.89612
H         -5.79516       -2.97310        0.89488
```

Originally assigned (incorrect) structure 134{3b} ($CDCl_3$)

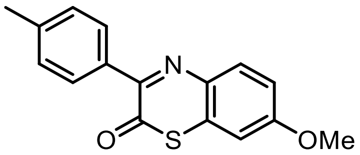

	Conf1	Conf2
Rel energy (kcal/mol):	0.00	0.38

C-nom	iGau	Exp	Calc	diff	1	2
C-C	9	183.73	177.45	-6.28	[177.34	177.66]
C-C	4	163.96	160.33	-3.63	[160.35	160.29]
C-C	8	158.66	146.22	-12.44	[146.11	146.44]
C-C	15	147.52	140.81	-6.71	[140.81	140.81]
C-C	2	143.70	132.69	-11.01	[133.05	132.01]
C-C	12	138.01	131.44	-6.57	[131.44	131.44]
C-C	1	131.58	128.46	-3.12	[128.48	128.42]
C-CH	6	130.28	136.00	5.72	[135.72	136.54]
C-CH	13	130.28	129.85	-0.43	[129.84	129.87]
C-CH	17	128.18	129.85	1.67	[129.84	129.87]
C-CH	14	128.18	127.55	-0.63	[127.56	127.54]
C-CH	16	125.37	127.55	2.18	[127.56	127.54]
C-CH	5	116.49	114.71	-1.78	[117.19	109.98]
C-CH	3	102.39	107.90	5.51	[105.66	112.17]
C-CH3	19	54.82	56.28	1.46	[56.30	56.25]
C-CH3	20	20.81	20.45	-0.36	[20.44	20.46]

13C chem shifts: RMSD=5.61ppm (MAE=4.34) N=16 {-12.44 5.72}

Fractions: 0.656 0.344

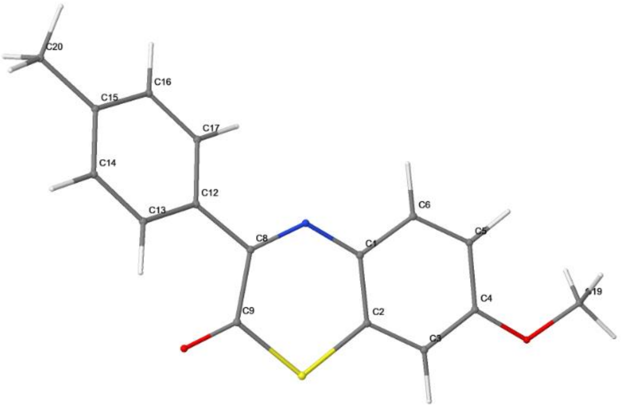

Conformer 1
Energy: -1220.90429 Hartree (Rel: 0.0 kcal/mol)
XYZ coordinates for conf 1:

C	1.27339	-0.69352	-0.04340
C	2.07766	0.46551	0.02425
C	3.47662	0.38756	0.00888
C	4.09330	-0.86380	-0.06983
C	3.30801	-2.03740	-0.13498
C	1.93412	-1.94608	-0.12327
N	-0.09954	-0.71574	-0.05470
C	-0.89989	0.30960	0.01034
C	-0.44533	1.72609	0.13702
S	1.32858	2.05083	0.13536
O	-1.16834	2.69995	0.25397
C	-2.36233	0.02947	0.00087
C	-3.32567	0.94029	-0.46718
C	-4.67633	0.59272	-0.49463
C	-5.12115	-0.65749	-0.04842

C	-4.15625	-1.56547	0.41774
C	-2.80737	-1.23334	0.43889
O	5.43261	-1.05740	-0.08895
C	6.29302	0.07991	-0.02893
C	-6.58815	-1.01457	-0.04559
H	-6.74898	-2.04931	-0.36866
H	4.06156	1.29836	0.06155
H	3.81266	-2.99582	-0.19555
H	1.31437	-2.83536	-0.17669
H	-3.02282	1.91768	-0.81769
H	-5.39863	1.31206	-0.87352
H	-4.46971	-2.54578	0.77000
H	-2.07455	-1.94937	0.79481
H	7.30852	-0.31603	-0.05892
H	6.13267	0.74313	-0.88685
H	6.14511	0.63938	0.90205
H	-7.16178	-0.35674	-0.70614
H	-7.01510	-0.92257	0.96209

Conformer 2
Energy: -1220.90490 Hartree (Rel: 0.4 kcal/mol)
XYZ coordinates for conf 2:

C	1.32025	-0.46635	-0.02620
C	2.03713	0.75489	0.03395
C	3.42965	0.77137	0.02269
C	4.14528	-0.43112	-0.04427
C	3.45534	-1.65936	-0.10175
C	2.07083	-1.66061	-0.09375
N	-0.04797	-0.59210	-0.04118
C	-0.92076	0.37205	0.01402
C	-0.57202	1.82058	0.13276
S	1.17002	2.28209	0.12859
O	-1.36897	2.73551	0.24409
C	-2.35892	-0.01383	0.00158
C	-3.38325	0.81807	-0.48374
C	-4.70447	0.37210	-0.51473
C	-5.05952	-0.90192	-0.05512
C	-4.03398	-1.73076	0.42825
C	-2.71314	-1.30021	0.45309
O	5.49392	-0.30860	-0.04757
C	6.29183	-1.49044	-0.11629
C	-6.49670	-1.36464	-0.05556
H	-6.57774	-2.41964	-0.34038
H	3.98183	1.70487	0.06802
H	3.99252	-2.59836	-0.15391
H	1.51908	-2.59396	-0.14163
H	-3.15050	1.81063	-0.84518
H	-5.47493	1.03173	-0.90721
H	-4.27710	-2.72705	0.79106
H	-1.93217	-1.95605	0.82244
H	7.32681	-1.14723	-0.10825
H	6.11518	-2.13975	0.74893
H	6.09821	-2.04697	-1.04049
H	-7.10703	-0.77533	-0.74723
H	-6.94359	-1.26573	0.94283

Revised structure of 134{3b}, i.e. 141{3b-rev} ($CDCl_3$)

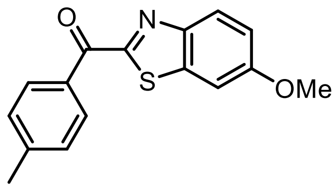

					Conf1	Conf2
	Rel energy (kcal/mol):				0.00	0.70

C-nom	iGau	Exp	Calc	diff	1	2
C-C	9	183.73	185.25	1.52	[185.23	185.30]
C-C	8	163.96	165.29	1.33	[165.21	165.54]
C-C	4	158.66	159.33	0.67	[159.41	159.07]
C-C	1	147.52	148.02	0.50	[147.93	148.31]
C-C	15	143.70	146.11	2.41	[146.10	146.13]
C-C	2	138.01	139.00	0.99	[139.19	138.36]
C-C	12	131.58	131.49	-0.09	[131.48	131.51]
C-CH	13	130.28	132.67	2.39	[132.67	132.69]
C-CH	17	130.28	132.67	2.39	[132.67	132.69]
C-CH	14	128.18	128.61	0.43	[128.61	128.59]
C-CH	16	128.18	128.61	0.43	[128.61	128.59]
C-CH	6	125.37	127.46	2.09	[127.35	127.83]
C-CH	5	116.49	116.44	-0.05	[118.20	110.69]
C-CH	3	102.39	103.28	0.89	[101.72	108.36]
C-CH3	19	54.82	56.11	1.29	[56.11	56.09]
C-CH3	20	20.81	20.81	0.00	[20.81	20.83]

13C chem shifts: RMSD=1.37ppm (MAE=1.09) N=16 {-0.09 2.41}

Fractions: 0.766 0.234

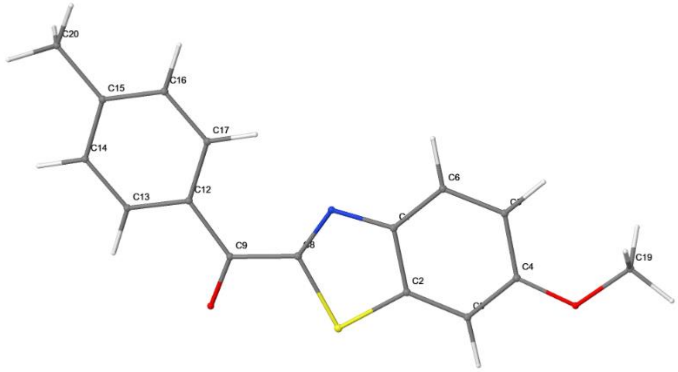

Conformer 1
Energy: -1220.91600 Hartree (Rel: 0.0 kcal/mol)
XYZ coordinates for conf 1:

C	1.55322	-0.72955	0.01217
C	2.30381	0.47370	-0.00265
C	3.70359	0.47110	-0.00615
C	4.34964	-0.76748	0.00569
C	3.60890	-1.97787	0.02091
C	2.22967	-1.96629	0.02423
N	0.18659	-0.56836	0.01350
C	-0.14534	0.68842	0.00011
C	-1.50421	1.30550	-0.00035
S	1.21634	1.83864	-0.01404
O	-1.54276	2.53830	0.00216
C	-2.75517	0.49843	-0.00184
C	-3.96946	1.21251	0.03030
C	-5.18640	0.54573	0.02649
C	-5.24134	-0.85877	-0.00920
C	-4.03168	-1.56487	-0.04486
C	-2.80350	-0.90546	-0.04025

O	5.69794	-0.92648	0.00384
C	6.52198	0.23661	-0.01081
C	-6.56770	-1.57813	0.00692
H	-6.45550	-2.63338	-0.25960
H	4.25842	1.40082	-0.01763
H	4.16317	-2.91060	0.02979
H	1.65977	-2.88984	0.03585
H	-3.93309	2.29616	0.05579
H	-6.11145	1.11699	0.04993
H	-4.05012	-2.65145	-0.07791
H	-1.88282	-1.47361	-0.06778
H	7.54993	-0.12775	-0.00952
H	6.34731	0.83583	-0.91244
H	6.35190	0.85513	0.87857
H	-7.27764	-1.12059	-0.69144
H	-7.02512	-1.53357	1.00406

Conformer 2
Energy: -1220.91712 Hartree (Rel: 0.7 kcal/mol)
XYZ coordinates for conf 2:

C	-1.59533	-0.49041	-0.00171
C	-2.26279	0.76595	0.00135
C	-3.65180	0.84913	0.00141
C	-4.38882	-0.34192	-0.00168
C	-3.73778	-1.59859	-0.00475
C	-2.35266	-1.67206	-0.00478
N	-0.21929	-0.42235	-0.00152
C	0.19607	0.80828	0.00169
C	1.59515	1.33074	0.00138
S	-1.08173	2.05359	0.00431
O	1.71666	2.55786	-0.00136
C	2.78824	0.44085	0.00259
C	4.04816	1.07125	-0.01102
C	5.21709	0.32319	-0.00810
C	5.17641	-1.08193	0.00664
C	3.92123	-1.70491	0.02426
C	2.74088	-0.96369	0.02108
O	-5.73910	-0.18906	-0.00147
C	-6.56549	-1.35069	-0.00505
C	6.45048	-1.89006	-0.01619
H	6.27719	-2.92248	0.30245
H	-4.17868	1.79711	0.00367
H	-4.31666	-2.51404	-0.00709
H	-1.84736	-2.63265	-0.00717
H	4.08565	2.15517	-0.02104
H	6.17897	0.83036	-0.01581
H	3.86568	-2.79062	0.04203
H	1.78342	-1.46799	0.03527
H	-7.59198	-0.98192	-0.00458
H	-6.39729	-1.95807	-0.90227
H	-6.39822	-1.96294	0.88903
H	7.21330	-1.45095	0.63623
H	6.87394	-1.92306	-1.02887

Originally assigned (incorrect) structure 135{3c} ($CDCl_3$)

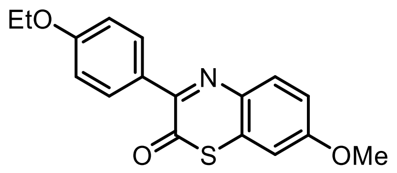

	Conf1	Conf2
Rel energy (kcal/mol):	0.00	0.40

C-nom	iGau	Exp	Calc	diff	1	2
C-C	9	182.20	177.78	-4.42	[177.69	177.96]
C-C	15	164.46	160.37	-4.09	[160.40	160.31]
C-C	4	162.67	159.86	-2.81	[159.87	159.84]
C-C	8	158.53	145.82	-12.71	[145.65	146.14]
C-C	2	147.51	132.35	-15.16	[132.68	131.70]
C-C	1	137.88	128.60	-9.28	[128.62	128.57]
C-C	12	126.72	126.73	0.01	[126.71	126.78]
C-CH	17	132.70	132.08	-0.62	[132.08	132.09]
C-CH	13	132.70	132.08	-0.62	[132.08	132.09]
C-CH	6	125.22	135.64	10.42	[135.37	136.16]
C-CH	5	116.38	114.68	-1.70	[117.12	109.89]
C-CH	14	113.23	113.38	0.15	[113.39	113.37]
C-CH	16	113.23	113.38	0.15	[113.39	113.37]
C-CH	3	102.38	107.82	5.44	[105.62	112.12]
C-CH2	21	62.81	62.13	-0.68	[62.12	62.14]
C-CH3	19	54.81	56.10	1.29	[56.11	56.09]
C-CH3	32	13.66	13.74	0.08	[13.74	13.74]

13C chem shifts: RMSD=6.26ppm (MAE=4.10) N=17 {-15.16 10.42}

Fractions: 0.662 0.338

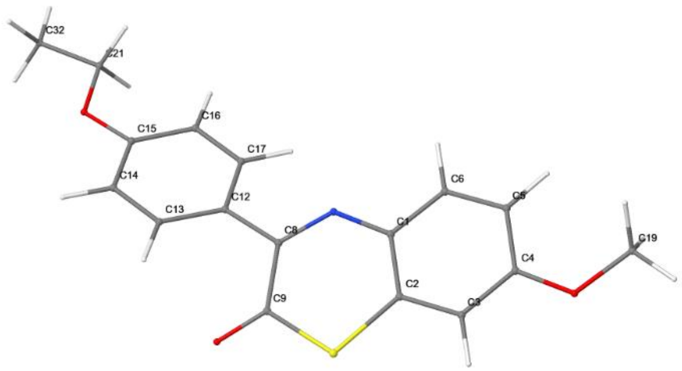

Conformer 1

Energy: -1335.42859 Hartree (Rel: 0.0 kcal/mol)

XYZ coordinates for conf 1:

C	1.96805	-0.67241	-0.05816
C	2.88219	0.40023	0.03017
C	4.26704	0.18540	0.02721
C	4.75897	-1.11915	-0.06003
C	3.86276	-2.20868	-0.14579
C	2.50419	-1.98278	-0.14603
N	0.60000	-0.55936	-0.08337
C	-0.09947	0.53813	-0.00963
C	0.49360	1.90141	0.13848
S	2.29046	2.05015	0.15045
O	-0.12845	2.94207	0.26323
C	-1.57923	0.40346	-0.03073
C	-2.45749	1.42817	-0.44371
C	-3.82890	1.22001	-0.48350
C	-4.37821	-0.01449	-0.10193
C	-3.52111	-1.04528	0.31446
C	-2.14671	-0.82884	0.33993

O	6.07381	-1.44305	-0.06908
C	7.03962	-0.39533	0.00855
O	-5.73175	-0.11102	-0.16751
C	-6.35930	-1.34511	0.20494
H	4.93780	1.03379	0.09567
H	4.27107	-3.21161	-0.21259
H	1.80109	-2.80662	-0.21558
H	-2.06461	2.39102	-0.73968
H	-4.50048	2.00774	-0.81086
H	-3.91226	-2.00818	0.62156
H	-1.48728	-1.62969	0.65594
H	8.01235	-0.88751	-0.01510
H	6.95327	0.28684	-0.84528
H	6.93755	0.16950	0.94256
C	-7.85733	-1.16717	0.03257
H	-6.10856	-1.58550	1.24663
H	-5.98287	-2.15616	-0.43241
H	-8.37514	-2.09191	0.30797
H	-8.10232	-0.92903	-1.00740
H	-8.22784	-0.35890	0.67108

Conformer 2
Energy: -1335.42922 Hartree (Rel: 0.4 kcal/mol)
XYZ coordinates for conf 2:

C	2.01491	-0.45027	-0.04305
C	2.87068	0.67591	0.04519
C	4.25577	0.52836	0.04769
C	4.82565	-0.74856	-0.03292
C	3.99628	-1.88515	-0.11896
C	2.62099	-1.72321	-0.12459
N	0.64170	-0.41331	-0.07404
C	-0.11410	0.64561	-0.00718
C	0.40397	2.04079	0.13967
S	2.18803	2.29306	0.15812
O	-0.27882	3.04292	0.26031
C	-1.58462	0.43406	-0.03264
C	-2.51079	1.40128	-0.47786
C	-3.86923	1.12137	-0.52185
C	-4.35701	-0.12989	-0.11194
C	-3.45146	-1.10390	0.33700
C	-2.09015	-0.81610	0.36575
O	6.18010	-0.78635	-0.02023
C	6.83258	-2.05343	-0.09744
O	-5.70323	-0.29818	-0.18416
C	-6.26916	-1.55355	0.21488
H	4.91321	1.38960	0.11449
H	4.41949	-2.88003	-0.18281
H	1.96427	-2.58450	-0.19475
H	-2.16557	2.37532	-0.79627
H	-4.57794	1.86438	-0.87451
H	-3.79521	-2.07721	0.66697
H	-1.39266	-1.57329	0.70694
H	7.90087	-1.83651	-0.06833
H	6.56561	-2.69056	0.75370
H	6.58974	-2.56906	-1.03378
C	-7.77274	-1.45849	0.02567
H	-6.01630	-1.75408	1.26449
H	-5.84491	-2.35981	-0.39813
H	-8.24454	-2.40152	0.32097
H	-8.01987	-1.26027	-1.02215
H	-8.19062	-0.65441	0.63976

Revised structure of 135{3c}, i.e. 142{3c-rev} ($CDCl_3$)

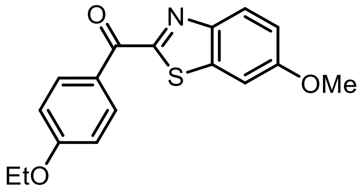

	Conf1	Conf2
Rel energy (kcal/mol):	0.00	0.72

C-nom	iGau	Exp	Calc	diff	1	2
C-C	9	182.20	183.99	1.79	[183.97	184.07]
C-C	8	164.46	165.82	1.36	[165.74	166.07]
C-C	15	162.67	163.83	1.16	[163.83	163.85]
C-C	4	158.53	159.01	0.48	[159.06	158.83]
C-C	1	147.51	148.16	0.65	[148.08	148.42]
C-C	2	137.88	138.88	1.00	[139.07	138.24]
C-C	12	126.72	127.15	0.43	[127.15	127.17]
C-CH	13	132.70	133.52	0.82	[133.52	133.53]
C-CH	17	132.70	133.52	0.82	[133.52	133.53]
C-CH	6	125.22	127.24	2.02	[127.14	127.56]
C-CH	5	116.38	116.29	-0.09	[118.00	110.52]
C-CH	14	113.23	113.14	-0.09	[113.14	113.13]
C-CH	16	113.23	113.14	-0.09	[113.14	113.13]
C-CH	3	102.38	103.28	0.90	[101.76	108.42]
C-CH2	21	62.81	62.81	0.00	[62.82	62.79]
C-CH3	19	54.81	55.93	1.12	[55.91	55.98]
C-CH3	32	13.66	13.69	0.03	[13.69	13.68]

13C chem shifts: RMSD=0.96ppm (MAE=0.76) N=17 {-0.09 2.02}

Fractions: 0.771 0.229

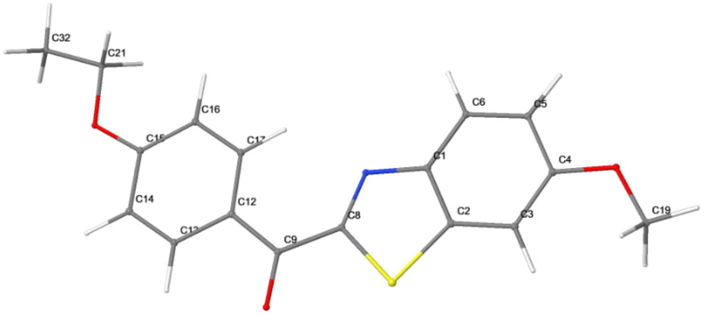

Conformer 1
Energy: -1335.44275 Hartree (Rel: 0.0 kcal/mol)
XYZ coordinates for conf 1:

C	-2.19449	-0.71556	0.00069
C	-3.08744	0.38597	-0.00000
C	-4.47624	0.21083	-0.00026
C	-4.96530	-1.09770	0.00029
C	-4.08136	-2.20743	0.00103
C	-2.71365	-2.02583	0.00123
N	-0.85733	-0.38673	0.00077
C	-0.68320	0.90121	0.00011
C	0.58788	1.68610	0.00018
S	-2.17643	1.87474	-0.00058
O	0.46529	2.91516	0.00027
C	1.92378	1.04649	0.00011
C	3.04426	1.90743	0.00101
C	4.33149	1.40575	0.00091
C	4.54670	0.01306	-0.00018
C	3.44523	-0.85708	-0.00114
C	2.15144	-0.34016	-0.00098
O	-6.28454	-1.42073	0.00022

C	-7.24445	-0.36705	-0.00130
O	5.84119	-0.38115	-0.00026
C	6.14823	-1.78444	-0.00074
H	-5.14137	1.06510	-0.00078
H	-4.51616	-3.20155	0.00142
H	-2.03459	-2.87251	0.00174
H	2.87382	2.97850	0.00183
H	5.19441	2.06427	0.00166
H	3.58500	-1.93143	-0.00207
H	1.30845	-1.01912	-0.00166
H	-8.22022	-0.85426	-0.00175
H	-7.14880	0.25931	0.89381
H	-7.14733	0.25806	-0.89712
C	7.66010	-1.92051	0.00011
H	5.70816	-2.25459	0.88801
H	5.70924	-2.25374	-0.89048
H	7.93607	-2.98013	-0.00030
H	8.09145	-1.45014	0.88936
H	8.09253	-1.44919	-0.88813

Conformer 2
Energy: -1335.44160 Hartree (Rel: 0.7 kcal/mol)
XYZ coordinates for conf 2:

C	2.24220	-0.47904	-0.00051
C	3.07769	0.67206	0.00052
C	4.46490	0.56090	0.00079
C	5.02914	-0.72105	0.00010
C	4.20971	-1.87452	-0.00093
C	2.82755	-1.75425	-0.00123
N	0.88820	-0.22001	-0.00075
C	0.64814	1.05609	0.00004
C	-0.66455	1.77148	-0.00010
S	2.08681	2.11172	0.00111
O	-0.60776	3.00500	-0.00014
C	-1.96430	1.06119	-0.00019
C	-3.12874	1.86175	-0.00159
C	-4.38763	1.29294	-0.00166
C	-4.52902	-0.10917	-0.00026
C	-3.38322	-0.91998	0.00120
C	-2.11843	-0.33555	0.00120
O	6.38808	-0.75778	0.00050
C	7.04361	-2.02349	-0.00051
O	-5.80093	-0.57091	-0.00042
C	-6.03371	-1.98849	0.00097
H	5.11875	1.42623	0.00154
H	4.65545	-2.86165	-0.00148
H	2.19360	-2.63529	-0.00204
H	-3.01478	2.94021	-0.00264
H	-5.28414	1.90495	-0.00277
H	-3.46626	-2.00024	0.00233
H	-1.24094	-0.96928	0.00226
H	8.11170	-1.80230	-0.00018
H	6.79203	-2.60475	0.89459
H	6.79224	-2.60322	-0.89665
C	-7.53635	-2.20355	0.00034
H	-5.56952	-2.43525	-0.88757
H	-5.57054	-2.43335	0.89098
H	-7.75663	-3.27611	0.00135
H	-7.99174	-1.75682	-0.88912
H	-7.99274	-1.75495	0.88835

Originally assigned (incorrect) structure 136{3h} ($CDCl_3$)

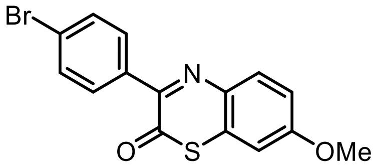

		Conf1	Conf2
Rel energy (kcal/mol):		0.00	0.38

C-nom	iGau	Exp	Calc	diff	1	2
C-C	9	184.17	177.40	-6.77	[177.29	177.61]
C-C	4	164.36	160.72	-3.64	[160.72	160.72]
C-C	8	160.07	144.17	-15.90	[144.01	144.48]
C-C	12	148.62	133.92	-14.70	[133.91	133.94]
C-C	2	139.34	133.05	-6.29	[133.47	132.25]
C-C	1	134.04	128.11	-5.93	[128.14	128.05]
C-C	15	129.34	124.30	-5.04	[124.31	124.28]
C-CH	6	132.81	136.41	3.60	[136.08	137.03]
C-CH	13	132.81	130.57	-2.24	[130.56	130.60]
C-CH	17	131.92	130.57	-1.35	[130.56	130.60]
C-CH	14	131.92	130.10	-1.82	[130.11	130.08]
C-CH	16	126.65	130.10	3.45	[130.11	130.08]
C-CH	3	117.94	107.97	-9.97	[105.71	112.27]
C-CH	5	103.53	115.00	11.47	[117.51	110.22]
C-CH3	20	56.01	56.25	0.24	[56.25	56.24]

13C chem shifts: RMSD=7.72ppm (MAE=6.16) N=15 {-15.90 11.47}

Fractions: 0.655 0.345

NOTICE:
the exp chem shift list does not correspond to the spectrum image -- values are taken from the image

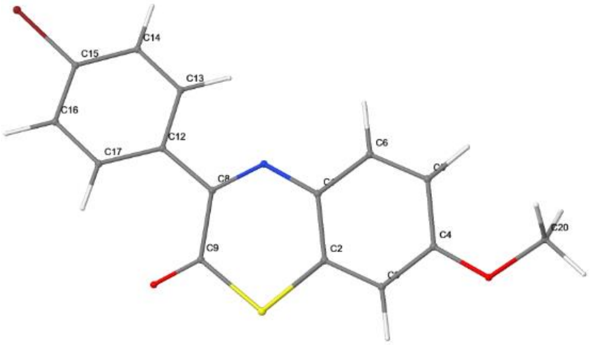

Conformer 1
Energy: -3752.39036 Hartree (Rel: 0.0 kcal/mol)
XYZ coordinates for conf 1:

C	2.27862	-0.66807	-0.03911
C	3.15758	0.43686	0.02014
C	4.54763	0.26600	0.00286
C	5.07960	-1.02442	-0.06998
C	4.21828	-2.14456	-0.12777
C	2.85403	-1.96283	-0.11378
N	0.90903	-0.59802	-0.04339
C	0.17884	0.47842	0.01661
C	0.72418	1.86202	0.12598
S	2.51497	2.06825	0.12214
O	0.06670	2.88319	0.22904
C	-1.30013	0.28715	0.01453
C	-1.81150	-0.95886	0.42520
C	-3.17748	-1.21988	0.41818
C	-4.05476	-0.22489	-0.01483
C	-3.58226	1.01592	-0.43411
C	-2.21062	1.27017	-0.41347
Br	-5.93679	-0.57207	-0.02953

O	6.40173	-1.30600	-0.09001
C	7.33754	-0.22861	-0.03599
H	5.19257	1.13551	0.04899
H	4.65883	-3.13425	-0.18413
H	2.17652	-2.80925	-0.16096
H	-1.11941	-1.72629	0.75271
H	-3.55550	-2.18155	0.74708
H	-4.27225	1.77990	-0.77466
H	-1.85390	2.23859	-0.73529
H	8.32358	-0.69294	-0.06385
H	7.22181	0.43873	-0.89770
H	7.22776	0.34409	0.89207

Conformer 2
Energy: -3752.39097 Hartree (Rel: 0.4 kcal/mol)
XYZ coordinates for conf 2:

C	2.31937	-0.44005	-0.02161
C	3.14064	0.71475	0.03077
C	4.52863	0.60841	0.01550
C	5.13515	-0.65325	-0.04822
C	4.33969	-1.81704	-0.09796
C	2.96106	-1.69688	-0.08575
N	0.94692	-0.44337	-0.02909
C	0.16364	0.59469	0.02065
C	0.63668	2.00705	0.12164
S	2.41179	2.31229	0.12075
O	-0.07764	2.98997	0.21672
C	-1.30381	0.32951	0.01643
C	-1.75469	-0.93075	0.45304
C	-3.10590	-1.26016	0.44427
C	-4.02875	-0.32070	-0.01698
C	-3.61616	0.93237	-0.46240
C	-2.25919	1.25553	-0.43959
Br	-5.89086	-0.76227	-0.03473
O	6.48789	-0.64968	-0.05652
C	7.18087	-1.89694	-0.12153
H	5.16141	1.48948	0.05462
H	4.79249	-2.79958	-0.14729
H	2.32926	-2.57830	-0.12741
H	-1.02725	-1.65466	0.80254
H	-3.43766	-2.23169	0.79348
H	-4.34114	1.65258	-0.82489
H	-1.94910	2.23289	-0.78219
H	8.24142	-1.64396	-0.11569
H	6.94952	-2.52459	0.74655
H	6.93848	-2.43739	-1.04361

Revised structure of 136{3h}, i.e. 143{3h-rev} ($CDCl_3$)

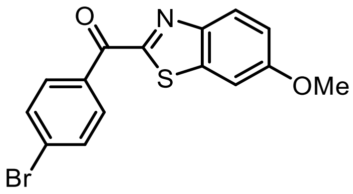

	Conf1	Conf2
Rel energy (kcal/mol):	0.00	0.71

C-nom	iGau	Exp	Calc	diff	1	2
C-C	9	184.17	183.37	-0.80	[183.34	183.49]
C-C	8	164.36	164.78	0.42	[164.70	165.06]
C-C	4	160.07	159.37	-0.70	[159.42	159.21]
C-C	1	148.62	147.30	-1.32	[147.24	147.51]
C-C	2	139.34	139.17	-0.17	[139.39	138.46]
C-C	11	134.04	132.73	-1.31	[132.73	132.72]
C-C	15	129.34	129.51	0.17	[129.51	129.52]
C-CH	13	132.81	133.35	0.54	[133.35	133.35]
C-CH	17	132.81	133.35	0.54	[133.35	133.35]
C-CH	14	131.92	131.37	-0.55	[131.37	131.37]
C-CH	16	131.92	131.37	-0.55	[131.37	131.37]
C-CH	6	126.65	127.62	0.97	[127.50	128.03]
C-CH	5	117.94	116.81	-1.13	[118.56	111.03]
C-CH	3	103.53	103.26	-0.27	[101.70	108.40]
C-CH3	20	56.01	56.02	0.01	[56.00	56.08]

13C chem shifts: RMSD=0.74ppm (MAE=0.63) N=15 {-1.32 0.97}

Fractions: 0.768 0.232

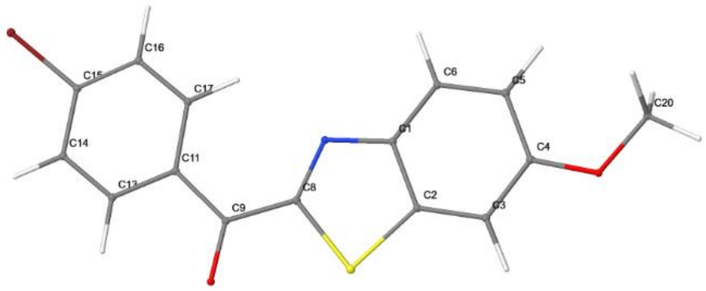

Conformer 1
Energy: -3752.40099 Hartree (Rel: 0.0 kcal/mol)
XYZ coordinates for conf 1:

C	2.52947	-0.69871	0.00052
C	3.39373	0.42644	-0.00008
C	4.78630	0.28835	-0.00049
C	5.30895	-1.00728	-0.00023
C	4.45397	-2.14064	0.00040
C	3.08277	-1.99578	0.00077
N	1.18618	-0.40515	0.00080
C	0.97901	0.87918	0.00046
C	-0.31379	1.61781	0.00065
S	2.44464	1.89067	-0.00023
C	-1.63738	0.92600	0.00042
O	-0.24835	2.84816	0.00086
C	-2.77624	1.75241	-0.00026
C	-4.05601	1.21029	-0.00053
C	-4.19900	-0.17889	-0.00008
C	-3.08938	-1.02274	0.00063
C	-1.81007	-0.46864	0.00086
Br	-5.95257	-0.93745	-0.00044
O	6.63452	-1.29615	-0.00055
C	7.56878	-0.21881	-0.00093
H	5.42875	1.15967	-0.00100
H	4.91543	-3.12258	0.00058

H	2.42598	-2.85973	0.00124
H	-2.63937	2.82805	-0.00058
H	-4.92936	1.85265	-0.00109
H	-3.21977	-2.09903	0.00099
H	-0.94428	-1.11795	0.00139
H	8.55589	-0.68235	-0.00092
H	7.45648	0.40346	-0.89676
H	7.45668	0.40394	0.89458

Conformer 2
Energy: -3752.39198 Hartree (Rel: 0.7 kcal/mol)
XYZ coordinates for conf 2:

C	-2.57130	-0.45941	-0.00053
C	-3.37997	0.71159	0.00017
C	-4.76909	0.63394	0.00049
C	-5.36296	-0.63475	0.00006
C	-4.57051	-1.80805	-0.00069
C	-3.18677	-1.72125	-0.00097
N	-1.21355	-0.23252	-0.00071
C	-0.94406	1.03903	-0.00018
C	0.38571	1.71134	-0.00028
S	-2.35607	2.12717	0.00056
C	1.67298	0.95432	-0.00019
O	0.38172	2.94312	-0.00041
C	2.85148	1.72318	-0.00050
C	4.10277	1.11837	-0.00038
C	4.17682	-0.27619	0.00006
C	3.02667	-1.06395	0.00038
C	1.77637	-0.44714	0.00025
Br	5.89061	-1.12064	0.00023
O	-6.72089	-0.64000	0.00036
C	-7.40774	-1.88982	0.00044
H	-5.40215	1.51454	0.00106
H	-5.03998	-2.78404	-0.00105
H	-2.57373	-2.61686	-0.00153
H	2.76836	2.80425	-0.00083
H	5.00666	1.71698	-0.00063
H	3.10345	-2.14540	0.00072
H	0.87958	-1.05285	0.00048
H	-8.46981	-1.64182	0.00109
H	-7.17095	-2.47547	-0.89564
H	-7.16994	-2.47591	0.89595

Originally assigned (incorrect) structure 137{3p} ($CDCl_3$)

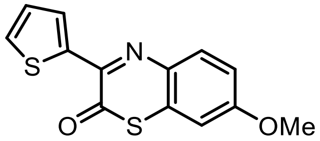

	Conf1	Conf2	Conf3	Conf4
Rel energy (kcal/mol):	0.00	0.36	0.89	1.25

C-nom	iGau	Exp	Calc	diff		1	2	3	4	
C-C	9	177.00	176.70	-0.30	[	176.37	176.73	177.47	177.85	]
C-C	4	164.16	160.13	-4.03	[	160.19	160.05	160.14	159.91	]
C-C	8	159.86	139.92	-19.94	[	139.58	139.79	141.06	141.25	]
C-C	12	148.48	138.23	-10.25	[	139.05	139.07	134.53	134.57	]
C-C	2	139.98	132.05	-7.93	[	132.60	131.53	131.66	130.66	]
C-C	1	139.23	128.12	-11.11	[	128.12	128.04	128.27	128.18	]
C-CH	6	137.15	135.72	-1.43	[	135.46	136.32	135.24	136.08	]
C-CH	17	136.53	133.24	-3.29	[	133.69	133.75	131.16	131.12	]
C-CH	15	128.50	130.81	2.31	[	130.39	130.33	132.77	132.78	]
C-CH	16	126.43	127.13	0.70	[	127.56	127.57	125.22	125.14	]
C-CH	5	117.80	115.07	-2.73	[	117.63	110.38	117.68	110.42	]
C-CH	3	103.63	108.06	4.43	[	105.69	112.23	105.98	112.52	]
C-CH3	20	55.99	56.26	0.27	[	56.29	56.22	56.26	56.12	]

13C chem shifts: RMSD=7.60ppm (MAE=5.28) N=13 {-19.94 4.43}

Fractions: 0.528 0.289 0.118 0.065

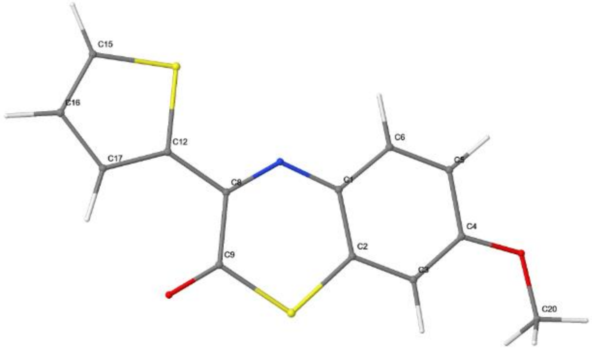


```
Conformer 1
Energy: -1502.34643 Hartree (Rel:  0.0 kcal/mol)
XYZ coordinates for conf 1:
C          0.86133       -0.65682        0.00024
C          1.63668        0.52492        0.00024
C          3.03706        0.48071        0.00020
C          3.68474       -0.75734        0.00023
C          2.92869       -1.95210        0.00034
C          1.55338       -1.89562        0.00033
N         -0.50770       -0.70995        0.00005
C         -1.32705        0.30650       -0.00012
C         -0.91035        1.73896        0.00018
S          0.85285        2.09832        0.00050
O         -1.66188        2.69805        0.00003
C         -2.75699        0.00500       -0.00010
S         -3.24259       -1.68538        0.00020
H         -6.07012        0.58960       -0.00085
C         -4.91733       -1.24692       -0.00053
C         -5.09648        0.11190       -0.00058
C         -3.86729        0.82818       -0.00030
H         -5.67615       -2.01875       -0.00066
O          5.02819       -0.91845        0.00027
C          5.85992        0.24156       -0.00142
H          3.59931        1.40717        0.00027
H          3.45729       -2.89946        0.00041
```

H	0.95636	-2.80175	0.00035
H	-3.79125	1.90580	-0.00032
H	6.88501	-0.12998	-0.00269
H	5.68912	0.84938	-0.89751
H	5.69166	0.85020	0.89460

Conformer 2
Energy: -1502.34586 Hartree (Rel: 0.4 kcal/mol)
XYZ coordinates for conf 2:

C	0.91295	-0.43330	0.00010
C	1.57092	0.82320	0.00018
C	2.96096	0.90513	0.00019
C	3.73336	-0.26357	0.00023
C	3.10256	-1.52486	0.00026
C	1.72017	-1.59291	0.00017
N	-0.44518	-0.61997	-0.00010
C	-1.35780	0.31279	-0.00032
C	-1.08080	1.78046	-0.00004
S	0.63634	2.31427	0.00029
O	-1.92442	2.65948	-0.00030
C	-2.75238	-0.12445	-0.00012
S	-3.07240	-1.85398	0.00040
H	-6.10654	0.13701	-0.00066
C	-4.78177	-1.57954	-0.00013
C	-5.09132	-0.24442	-0.00040
C	-3.93713	0.58738	-0.00028
H	-5.46246	-2.42108	-0.00007
O	5.07438	-0.07685	0.00039
C	5.92868	-1.22082	-0.00096
H	3.46816	1.86482	0.00016
H	3.68426	-2.43845	0.00041
H	1.21427	-2.55302	0.00014
H	-3.96596	1.66730	-0.00042
H	6.94578	-0.82778	-0.00198
H	5.77385	-1.83286	0.89496
H	5.77181	-1.83229	-0.89692

Conformer 3
Energy: -1502.34445 Hartree (Rel: 0.9 kcal/mol)
XYZ coordinates for conf 3:

C	0.98087	0.78587	0.00045
C	1.66006	-0.45426	0.00010
C	3.05944	-0.52319	0.00000
C	3.80532	0.65819	0.00027
C	3.14785	1.90980	0.00064
C	1.77244	1.96397	0.00075
N	-0.37946	0.95388	0.00051
C	-1.27117	0.00097	0.00024
C	-0.96197	-1.45442	0.00017
S	0.75579	-1.96256	-0.00027
O	-1.80558	-2.33716	0.00034
C	-2.68174	0.40201	-0.00012
S	-4.04383	-0.70627	0.00053
H	-5.07179	2.77462	-0.00157
C	-5.16997	0.60555	-0.00035
C	-4.54387	1.82732	-0.00104
C	-3.13131	1.71068	-0.00093
H	-6.23307	0.40119	-0.00036
O	5.15756	0.71023	0.00030
C	5.89331	-0.51299	-0.00121
H	3.54534	-1.49187	-0.00025
H	3.75090	2.81161	0.00078
H	1.25053	2.91541	0.00098
H	-2.44452	2.54798	-0.00141
H	6.94496	-0.22512	-0.00186
H	5.67629	-1.10632	0.89464
H	5.67481	-1.10496	-0.89760

Conformer 4
Energy: -1502.34502 Hartree (Rel: 1.2 kcal/mol)
XYZ coordinates for conf 4:

C	1.02956	-0.55805	0.00035
C	1.59491	0.74330	0.00038
C	2.97503	0.92777	0.00027
C	3.83188	-0.18044	0.00013
C	3.29545	-1.48461	0.00016
C	1.92186	-1.65409	0.00027
N	-0.31067	-0.84884	0.00045
C	-1.28338	0.02026	0.00019
C	-1.10564	1.49909	-0.00024
S	0.55638	2.16488	0.00060
O	-2.02747	2.29957	-0.00081
C	-2.65269	-0.50538	-0.00004
S	-4.10909	0.47574	-0.00043
H	-4.81960	-3.08353	0.00024
C	-5.11264	-0.93215	-0.00020
C	-4.37910	-2.09255	0.00022
C	-2.98276	-1.84921	0.00025
H	-6.18979	-0.82412	-0.00051
O	5.15555	0.10462	0.00006
C	6.09173	-0.97340	-0.00112
H	3.40960	1.92250	0.00018
H	3.94286	-2.35285	0.00014
H	1.48828	-2.64900	0.00027
H	-2.22332	-2.62123	0.00044
H	7.07728	-0.50681	-0.00187
H	5.98216	-1.59525	0.89477
H	5.98053	-1.59470	-0.89718

Revised structure of 137{3p}, i.e. 144{3p-rev} ($CDCl_3$)

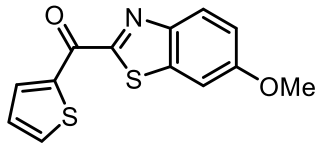

	Conf1	Conf2	Conf3	Conf4
Rel energy (kcal/mol):	0.00	0.54	0.69	1.20

C-nom	iGau	Exp	Calc	diff	1	2	3	4
C-C	9	177.00	176.23	-0.77	[176.12	176.42	176.23	176.53]
C-C	8	164.16	163.60	-0.56	[163.50	163.56	163.86	163.92]
C-C	4	159.86	159.18	-0.68	[159.28	159.30	158.83	158.89]
C-C	1	148.48	147.35	-1.13	[147.31	147.19	147.64	147.48]
C-C	2	139.98	139.19	-0.79	[139.31	139.61	138.46	138.79]
C-C	11	139.23	137.88	-1.35	[138.99	135.18	138.98	135.19]
C-CH	16	137.15	139.48	2.33	[139.27	139.96	139.30	140.05]
C-CH	14	136.53	137.18	0.65	[136.71	138.30	136.76	138.35]
C-CH	15	128.50	127.01	-1.49	[127.60	125.60	127.52	125.62]
C-CH	6	126.43	127.35	0.92	[127.20	127.32	127.66	127.83]
C-CH	5	117.80	116.66	-1.14	[118.46	118.50	110.94	110.95]
C-CH	3	103.63	103.48	-0.15	[101.84	101.95	108.53	108.62]
C-CH3	18	55.99	56.05	0.06	[56.05	56.09	55.97	56.06]

13C chem shifts: RMSD=1.09ppm (MAE=0.92) N=13 {-1.49 2.33}

Fractions: 0.540 0.219 0.169 0.071

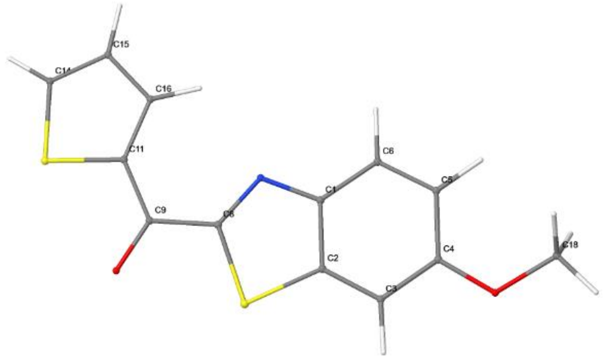

Conformer 1
Energy: -1502.35572 Hartree (Rel: 0.0 kcal/mol)
XYZ coordinates for conf 1:

C	1.27631	0.78058	-0.00017
C	1.91260	-0.48719	-0.00000
C	3.30615	-0.61591	0.00005
C	4.06492	0.55708	-0.00004
C	3.43992	1.83131	-0.00020
C	2.06568	1.94868	-0.00027
N	-0.09953	0.74659	-0.00021
C	-0.54079	-0.47609	-0.00009
C	-1.95474	-0.93788	-0.00007
S	0.70331	-1.74671	0.00007
C	-3.05255	0.02764	0.00004
O	-2.16929	-2.15434	-0.00019
S	-4.68236	-0.61993	-0.00009
C	-5.35035	0.96994	0.00013
C	-4.38287	1.94675	0.00031
C	-3.06966	1.41063	0.00026
O	5.42190	0.58957	0.00001
C	6.13418	-0.64544	0.00032
H	3.77105	-1.59364	0.00016
H	4.07869	2.70838	-0.00027
H	1.58459	2.92151	-0.00041
H	-6.42457	1.10329	0.00014

```
H         -4.60320        3.00832        0.00048
H         -2.16182        1.99893        0.00037
H          7.19151       -0.37824        0.00043
H          5.90569       -1.23532        0.89605
H          5.90595       -1.23562       -0.89528

Conformer 2
Energy: -1502.35681 Hartree (Rel:  0.5 kcal/mol)
XYZ coordinates for conf 2:
C         -1.10848       -0.66895       -0.00044
C         -1.82677        0.55473        0.00010
C         -3.22553        0.59182        0.00037
C         -3.90536       -0.62882       -0.00002
C         -3.19797       -1.85911       -0.00057
C         -1.81892       -1.88641       -0.00077
N          0.26179       -0.54052       -0.00058
C          0.61896        0.70859       -0.00015
C          2.00829        1.22873       -0.00032
S         -0.70211        1.89241        0.00044
C          3.14932        0.30409       -0.00021
O          2.17736        2.45089       -0.00056
S          3.07947       -1.44863        0.00065
C          4.80237       -1.55303        0.00062
C          5.40695       -0.31842       -0.00007
C          4.46380        0.73704       -0.00051
O         -5.25715       -0.75025        0.00012
C         -6.04902        0.43528        0.00035
H         -3.75420        1.53667        0.00085
H         -3.77780       -2.77617       -0.00081
H         -1.27507       -2.82550       -0.00116
H          5.28052       -2.52455        0.00101
H          6.48137       -0.17420       -0.00020
H          4.71379        1.79125       -0.00099
H         -7.08651        0.09919        0.00029
H         -5.85992        1.03889        0.89610
H         -5.85992        1.03922       -0.89519

Conformer 3
Energy: -1502.35596 Hartree (Rel:  0.7 kcal/mol)
XYZ coordinates for conf 3:
C         -1.31412        0.54080        0.00022
C         -1.85062       -0.77712       -0.00004
C         -3.22350       -1.00258       -0.00016
C         -4.07839        0.10701       -0.00006
C         -3.55888        1.42358        0.00019
C         -2.18873        1.63855        0.00033
N          0.06172        0.61332        0.00031
C          0.59519       -0.57081        0.00014
C          2.04231       -0.91976        0.00010
S         -0.54390       -1.93859       -0.00013
C          3.06024        0.12949       -0.00008
O          2.35116       -2.11553        0.00023
S          4.73652       -0.38614        0.00005
C          5.27566        1.25178       -0.00011
C          4.23351        2.14852       -0.00026
C          2.96729        1.50957       -0.00026
O         -5.40576       -0.18262       -0.00020
C         -6.34662        0.88875       -0.00011
H         -3.65031       -1.99954       -0.00033
H         -4.22850        2.27480        0.00027
H         -1.78455        2.64586        0.00053
H          6.33584        1.47025       -0.00012
H          4.36876        3.22425       -0.00038
H          2.01547        2.02361       -0.00036
H         -7.32995        0.41687       -0.00024
H         -6.24209        1.51228        0.89569
H         -6.24196        1.51253       -0.89572

Conformer 4
Energy: -1502.35490 Hartree (Rel:  1.2 kcal/mol)
XYZ coordinates for conf 4:
```

C	1.15020	-0.43238	0.00057
C	1.76219	0.85264	-0.00027
C	3.14557	0.99825	-0.00072
C	3.93454	-0.15937	-0.00028
C	3.33953	-1.44353	0.00058
C	1.95919	-1.57911	0.00100
N	-0.22742	-0.42082	0.00088
C	-0.68853	0.79253	0.00029
C	-2.11893	1.18928	0.00049
S	0.52448	2.08967	-0.00064
C	-3.17461	0.16850	0.00038
O	-2.39375	2.39180	0.00075
S	-2.95251	-1.57150	-0.00090
C	-4.65978	-1.82527	-0.00085
C	-5.36955	-0.64795	0.00015
C	-4.52178	0.48547	0.00080
O	5.27629	0.05308	-0.00085
C	6.15393	-1.07075	0.00042
H	3.62958	1.96865	-0.00137
H	3.95854	-2.33222	0.00088
H	1.49714	-2.56111	0.00162
H	-5.05186	-2.83462	-0.00142
H	-6.45243	-0.59771	0.00035
H	-4.86251	1.51394	0.00148
H	7.16277	-0.65625	0.00056
H	6.01304	-1.68658	0.89651
H	6.01388	-1.68798	-0.89484

Originally assigned (incorrect) structure 138{3q} ($CDCl_3$)

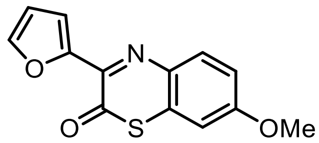

	Conf1	Conf2	Conf3	Conf4
Rel energy (kcal/mol):	0.00	0.34	2.30	2.67

C-nom	iGau	Exp	Calc	diff	1	2	3	4
C-C	9	172.20	176.53	4.33	[176.45	176.79	174.26	174.59]
C-C	4	163.59	159.88	-3.71	[159.96	159.74	159.97	159.73]
C-C	12	159.85	147.67	-12.18	[147.62	147.61	150.26	150.29]
C-C	8	150.05	137.48	-12.57	[137.40	137.60	137.91	138.13]
C-C	2	148.56	131.37	-17.19	[131.68	130.74	132.88	131.85]
C-C	1	139.05	128.43	-10.62	[128.47	128.38	128.17	128.11]
C-CH	15	148.76	145.56	-3.20	[145.58	145.52	145.63	145.60]
C-CH	6	126.37	135.73	9.36	[135.44	136.27	134.91	135.70]
C-CH	5	124.60	114.90	-9.70	[117.50	110.27	117.27	110.02]
C-CH	17	117.89	120.07	2.18	[120.15	120.14	116.32	116.26]
C-CH	16	112.95	111.44	-1.51	[111.44	111.45	111.15	111.12]
C-CH	3	103.59	108.18	4.59	[105.84	112.36	105.91	112.40]
C-CH3	20	56.00	56.19	0.19	[56.24	56.11	56.22	56.06]

13C chem shifts: RMSD=8.63ppm (MAE=7.03) N=13 {-17.19 9.36}

Fractions: 0.628 0.352 0.013 0.007

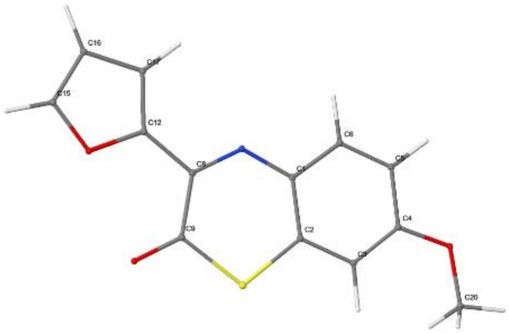

Conformer 1
Energy: -1179.36349 Hartree (Rel: 0.0 kcal/mol)
XYZ coordinates for conf 1:

C	0.66869	-0.73947	-0.00025
C	1.38501	0.47914	0.00033
C	2.78599	0.50543	0.00054
C	3.49549	-0.69810	0.00015
C	2.80044	-1.92901	-0.00042
C	1.42377	-1.94103	-0.00059
N	-0.69659	-0.86745	-0.00053
C	-1.55473	0.11506	-0.00056
C	-1.21321	1.56611	-0.00016
S	0.52655	2.01430	0.00094
O	-2.01903	2.48068	-0.00049
C	-2.97474	-0.21435	-0.00013
O	-3.28972	-1.55185	0.00123
H	-6.27395	-0.18635	-0.00109
C	-4.64263	-1.64720	0.00101
C	-5.21609	-0.40840	-0.00051
C	-4.13814	0.52422	-0.00130
H	-5.04059	-2.65094	0.00215
O	4.84597	-0.79091	0.00044
C	5.61733	0.40987	-0.00070
H	3.30104	1.45898	0.00107
H	3.37564	-2.84886	-0.00072
H	0.87227	-2.87553	-0.00106
H	-4.19839	1.59990	-0.00254

```
H          6.66013        0.09126       -0.00185
H          5.41619        1.00869       -0.89664
H          5.41829        1.00907        0.89548

Conformer 2
Energy: -1179.36291 Hartree (Rel:  0.3 kcal/mol)
XYZ coordinates for conf 2:
C         -0.72348       -0.52312       -0.00001
C         -1.31388        0.76671        0.00012
C         -2.69753        0.92410        0.00015
C         -3.53235       -0.20075        0.00007
C         -2.97069       -1.49405       -0.00000
C         -1.59374       -1.63645       -0.00004
N          0.62251       -0.78884       -0.00019
C          1.57431        0.10228       -0.00030
C          1.38018        1.58197        0.00006
S         -0.30278        2.20831        0.00027
O          2.27645        2.40791        0.00031
C          2.95426       -0.36838       -0.00006
O          3.13224       -1.73113        0.00100
H          6.23944       -0.67499       -0.00144
C          4.46846       -1.96308        0.00063
C          5.16454       -0.78884       -0.00076
C          4.18663        0.24821       -0.00111
H          4.76261       -3.00201        0.00147
O         -4.86181        0.05852        0.00016
C         -5.77605       -1.03789       -0.00054
H         -3.15143        1.91022        0.00021
H         -3.60085       -2.37493        0.00002
H         -1.14011       -2.62238       -0.00013
H          4.35598        1.31214       -0.00193
H         -6.77079       -0.59115       -0.00090
H         -5.65294       -1.65707       -0.89654
H         -5.65380       -1.65752        0.89527

Conformer 3
Energy: -1179.36661 Hartree (Rel:  2.3 kcal/mol)
XYZ coordinates for conf 3:
C          0.70201       -0.76584        0.01001
C          1.39377        0.46686        0.00188
C          2.79388        0.51934       -0.00711
C          3.52647       -0.67046       -0.00444
C          2.85550       -1.91473        0.00768
C          1.47938       -1.95323        0.01361
N         -0.65977       -0.91721        0.00606
C         -1.54604        0.04098        0.00840
C         -1.23937        1.50080        0.02942
S          0.50117        1.98152        0.00459
O         -2.05337        2.40263        0.06313
C         -2.94112       -0.39270        0.00146
O         -3.96236        0.51823       -0.06858
H         -5.62288       -2.31130        0.02434
C         -5.12556       -0.18009       -0.06585
C         -4.88315       -1.52337        0.00476
C         -3.46794       -1.66473        0.04730
H         -6.02610        0.41305       -0.11717
O          4.87837       -0.73753       -0.01257
C          5.62713        0.47745       -0.02993
H          3.29054        1.48252       -0.01476
H          3.44845       -2.82322        0.01041
H          0.94668       -2.89867        0.02026
H         -2.89532       -2.57809        0.10542
H          6.67565        0.17828       -0.03587
H          5.40833        1.06421       -0.92970
H          5.42338        1.08092        0.86228

Conformer 4
Energy: -1179.36716 Hartree (Rel:  2.7 kcal/mol)
XYZ coordinates for conf 4:
C         -0.75523       -0.54838        0.00001
C         -1.32269        0.75170       -0.00003
```

C	-2.70350	0.93174	-0.00010
C	-3.55736	-0.17884	-0.00015
C	-3.01803	-1.48168	-0.00013
C	-1.64372	-1.64730	-0.00005
N	0.58558	-0.83420	0.00003
C	1.56079	0.03246	0.00004
C	1.39842	1.51705	0.00016
S	-0.28326	2.17177	0.00008
O	2.29996	2.33193	0.00033
C	2.90683	-0.53605	0.00003
O	4.01466	0.27046	-0.00047
H	5.38475	-2.71198	0.00034
C	5.10272	-0.53985	-0.00039
C	4.72717	-1.85406	0.00015
C	3.30434	-1.85511	0.00041
H	6.05849	-0.03792	-0.00075
O	-4.88215	0.10295	-0.00031
C	-5.81524	-0.97744	0.00022
H	-3.14071	1.92531	-0.00012
H	-3.66319	-2.35159	-0.00023
H	-1.20741	-2.64106	-0.00005
H	2.64297	-2.70833	0.00083
H	-6.80209	-0.51358	0.00050
H	-5.70364	-1.59905	-0.89563
H	-5.70296	-1.59874	0.89620

Revised structure of 138{3q}, i.e. 145{3q-rev} ($CDCl_3$)

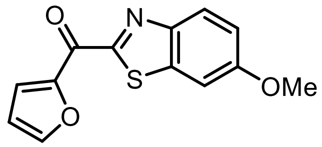

					Conf1	Conf2	Conf3	Conf4
		Rel energy (kcal/mol):			0.00	0.68	1.40	2.05

C-nom	iGau	Exp	Calc	diff	1	2	3	4
C-C	9	172.20	172.94	0.74	[172.81	172.90	174.07	174.15]
C-C	8	163.59	164.05	0.46	[164.02	164.41	163.26	163.60]
C-C	4	159.85	159.01	-0.84	[159.11	158.66	159.26	158.83]
C-C	11	150.05	149.94	-0.11	[149.84	149.89	150.86	150.90]
C-C	1	148.56	147.48	-1.08	[147.30	147.67	148.31	148.64]
C-C	2	139.05	138.96	-0.09	[139.15	138.32	139.25	138.40]
C-CH	14	148.76	149.66	0.90	[149.71	149.74	149.07	149.09]
C-CH	6	126.37	127.17	0.80	[127.01	127.45	127.61	128.10]
C-CH	16	124.60	125.88	1.28	[125.98	125.94	124.92	125.12]
C-CH	5	117.89	116.53	-1.36	[118.37	110.84	118.19	110.68]
C-CH	15	112.95	111.47	-1.48	[111.43	111.48	111.72	111.81]
C-CH	3	103.59	103.52	-0.07	[101.91	108.60	101.74	108.39]
C-CH3	18	56.00	56.01	0.01	[56.03	55.95	56.04	55.96]

13C chem shifts: RMSD=0.87ppm (MAE=0.71) N=13 {-1.48 1.28}

Fractions: 0.692 0.221 0.065 0.022

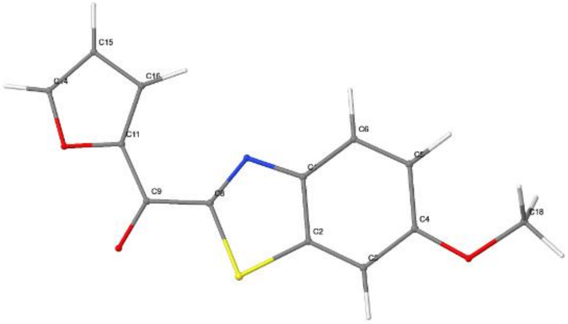

Conformer 1
Energy: -1179.37604 Hartree (Rel: 0.0 kcal/mol)
XYZ coordinates for conf 1:

C	-0.95994	0.76741	-0.00073
C	-1.60881	-0.49386	0.00007
C	-3.00359	-0.60885	0.00046
C	-3.75083	0.57159	-0.00002
C	-3.11327	1.83931	-0.00085
C	-1.73774	1.94301	-0.00120
N	0.41606	0.71952	-0.00098
C	0.84333	-0.50799	-0.00041
C	2.25380	-0.97933	-0.00064
S	-0.41176	-1.76591	0.00044
C	3.32782	0.00825	0.00001
O	2.47736	-2.19132	-0.00125
O	4.60300	-0.50736	-0.00029
C	5.45377	0.54089	0.00053
C	4.77179	1.72785	0.00140
C	3.39239	1.38451	0.00102
O	-5.10769	0.61750	0.00025
C	-5.83199	-0.61034	0.00108
H	-3.47821	-1.58195	0.00109
H	-3.74316	2.72279	-0.00120
H	-1.24719	2.91113	-0.00182
H	6.50492	0.29286	0.00041
H	5.20238	2.71907	0.00216
H	2.54112	2.04705	0.00143

```
H         -6.88668       -0.33280        0.00114
H         -5.60974       -1.20320       -0.89428
H         -5.60938       -1.20220        0.89702

Conformer 2
Energy: -1179.37712 Hartree (Rel:  0.7 kcal/mol)
XYZ coordinates for conf 2:
C          1.00332       -0.53848       -0.00029
C          1.54123        0.77883        0.00017
C          2.91423        1.00337        0.00036
C          3.76837       -0.10677        0.00008
C          3.24779       -1.42267       -0.00040
C          1.87719       -1.63659       -0.00058
N         -0.37334       -0.60927       -0.00043
C         -0.90331        0.57632       -0.00008
C         -2.35004        0.92505       -0.00029
S          0.23583        1.94239        0.00039
C         -3.33621       -0.14984        0.00000
O         -2.67439        2.11378       -0.00067
O         -4.65107        0.25452       -0.00036
C         -5.40842       -0.86306        0.00005
C         -4.62698       -1.98713        0.00064
C         -3.28226       -1.52656        0.00062
O          5.09615        0.18224        0.00028
C          6.03617       -0.88967        0.00001
H          3.34163        2.00009        0.00073
H          3.91664       -2.27452       -0.00063
H          1.47250       -2.64371       -0.00094
H         -6.47702       -0.70656       -0.00017
H         -4.97101       -3.01162        0.00103
H         -2.37686       -2.11300        0.00100
H          7.01988       -0.41853        0.00026
H          5.93112       -1.51363        0.89548
H          5.93128       -1.51305       -0.89588

Conformer 3
Energy: -1179.37489 Hartree (Rel:  1.4 kcal/mol)
XYZ coordinates for conf 3:
C         -0.92363        0.74821       -0.04134
C         -1.58777       -0.50531       -0.00018
C         -2.98370       -0.60323        0.01913
C         -3.71687        0.58577       -0.00445
C         -3.06485        1.84561       -0.04775
C         -1.68846        1.93259       -0.06613
N          0.45085        0.68809       -0.05172
C          0.86546       -0.54293       -0.02104
C          2.26294       -1.04324       -0.02469
S         -0.40649       -1.79014        0.02008
C          3.40146       -0.13032       -0.00051
O          2.45468       -2.26319       -0.04692
O          3.24378        1.23039        0.06687
C          4.48259        1.77104        0.07744
C          5.44438        0.79773        0.01845
C          4.74404       -0.43663       -0.03083
O         -5.07300        0.64778        0.01146
C         -5.81142       -0.57065        0.05975
H         -3.46998       -1.57005        0.05165
H         -3.68478        2.73595       -0.06502
H         -1.18593        2.89396       -0.09838
H          4.52307        2.84894        0.12939
H          6.51426        0.94982        0.01187
H          5.15698       -1.43337       -0.08335
H         -6.86272       -0.28057        0.06684
H         -5.60952       -1.19284       -0.82042
H         -5.58223       -1.13808        0.96975

Conformer 4
Energy: -1179.37385 Hartree (Rel:  2.1 kcal/mol)
XYZ coordinates for conf 4:
C          0.96960       -0.51974       -0.00005
C          1.52067        0.79288        0.00034
```

C	2.89598	1.00321	0.00041
C	3.73885	-0.11559	0.00014
C	3.20560	-1.42666	-0.00022
C	1.83321	-1.62653	-0.00032
N	-0.40624	-0.58152	-0.00015
C	-0.92706	0.60756	0.00018
C	-2.36455	0.98134	-0.00024
S	0.22726	1.96786	0.00052
C	-3.41956	-0.02718	-0.00025
O	-2.66228	2.17982	-0.00070
O	-3.14668	-1.37137	0.00082
C	-4.33534	-2.01501	0.00080
C	-5.37651	-1.12517	-0.00026
C	-4.78354	0.16507	-0.00093
O	5.06944	0.16011	0.00031
C	5.99880	-0.92112	-0.00071
H	3.33396	1.99536	0.00068
H	3.86618	-2.28496	-0.00038
H	1.41742	-2.62904	-0.00061
H	-4.28407	-3.09369	0.00156
H	-6.42973	-1.36710	-0.00058
H	-5.27942	1.12476	-0.00186
H	6.98715	-0.45980	-0.00092
H	5.88798	-1.54421	0.89465
H	5.88712	-1.54324	-0.89664

Table S2: Comparison ^{13}C spectral data between actual compounds (133-139) and revised compounds (140-146) with known compound references.

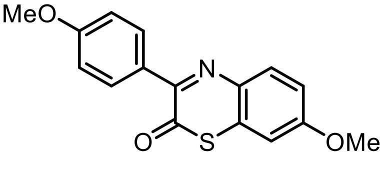 133{3a}	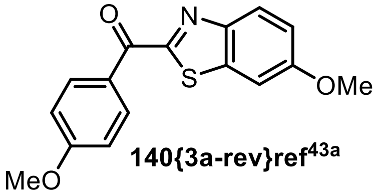 140{3a-rev}ref[43a]			
^{13}C, ppm	^{13}C, ppm	Δ	ppm (offset by -1.02)	Δ
182.26	183.26	1.00	182.24	0.02
164.39	165.43	1.04	164.41	-0.02
163.21	164.23	1.02	163.21	0.00
158.56	159.59	1.03	158.57	-0.01
147.51	148.55	1.04	147.53	-0.02
137.9	138.92	1.02	137.90	0.00
132.69	133.70	1.01	132.68	0.01
126.93	127.99	1.06	126.97	-0.04
125.24	126.25	1.01	125.23	0.01
116.41	117.39	0.98	116.37	0.04
112.8	113.82	1.02	112.80	0.00
102.38	103.44	1.06	102.42	-0.04
54.82	55.84	1.02	54.82	0.00
54.53	55.53	1.00	54.51	0.02
	**rmsd=1.02ppm**		**rmsd=0.02ppm**	

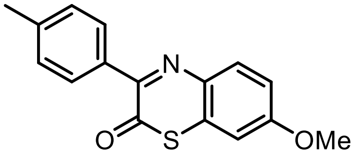 134{3b}	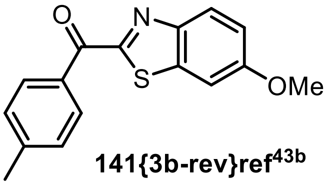 141{3b-rev}ref[43b]			
^{13}C, ppm	^{13}C, ppm	Δ	ppm (offset by -0.88)	Δ
183.73	184.57	0.84	183.69	0.04
163.95	164.83	0.88	163.95	0.00
158.65	159.52	0.87	158.64	0.01
147.52	148.39	0.87	147.51	0.01
143.70	144.58	0.88	143.70	0.00
138.01	138.88	0.87	138.00	0.01
131.58	132.45	0.87	131.57	0.01
130.27	131.16	0.89	130.28	-0.01
128.18	129.07	0.89	128.19	-0.01
125.37	126.25	0.88	125.37	0.00
116.49	117.38	0.89	116.50	-0.01
102.38	103.25	0.87	102.37	0.01
54.82	55.71	0.89	54.83	-0.01
20.81	21.71	0.90	20.83	-0.02
	**rmsd=0.88ppm**		**rmsd=0.01ppm**	

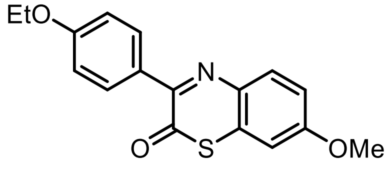 **135{3c}**	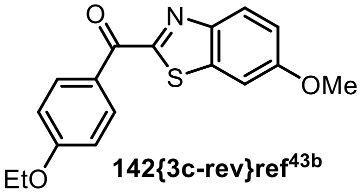 **142{3c-rev}ref[43b]**			
**^{13}C, ppm**	**^{13}C, ppm**	Δ	**ppm** (offset by -0.89)	Δ
182.20	183.11	0.91	182.22	-0.02
164.46	165.35	0.89	164.46	0.00
162.67	163.54	0.87	162.65	0.02
158.53	159.41	0.88	158.52	0.01
147.52	148.40	0.88	147.51	0.01
137.88	138.77	0.89	137.88	0.00
132.70	133.58	0.88	132.69	0.01
126.72	127.60	0.88	126.71	0.01
125.22	126.12	0.90	125.23	-0.01
116.38	117.28	0.90	116.39	-0.01
113.23	114.12	0.89	113.23	0.00
102.38	103.26	0.88	102.37	0.01
62.81	63.71	0.90	62.82	-0.01
54.81	55.72	0.91	54.83	-0.02
13.66	14.56	0.90	13.67	-0.01
	**rmsd=0.89ppm**		**rmsd=0.01ppm**	

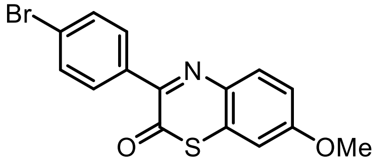 **136{3h}**	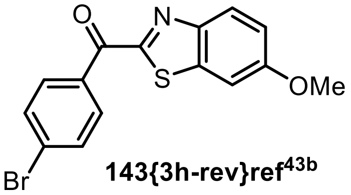 **143{3h-rev}ref[43b]**			
**^{13}C, ppm**	**^{13}C, ppm**	Δ	**ppm** (offset by -0.97)	Δ
183.02	183.96	0.94	182.99	0.03
163.21	164.18	0.97	163.21	0.00
158.92	159.88	0.96	158.91	0.01
147.47	148.43	0.96	147.46	0.01
138.19	139.16	0.97	138.19	0.00
132.89	133.85	0.96	132.88	0.01
131.66	132.63	0.97	131.66	0.00
130.77	131.74	0.97	130.77	0.00
128.19	129.16	0.97	128.19	0.00
125.5	126.47	0.97	125.50	0.00
116.79	117.77	0.98	116.80	-0.01
102.38	103.34	0.96	102.37	0.01
54.86	55.84	0.98	54.87	-0.01
	**rmsd=0.97ppm**		**rmsd=0.01ppm**	

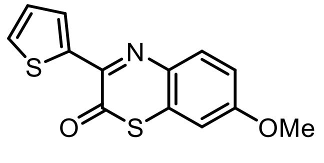 137{3p}	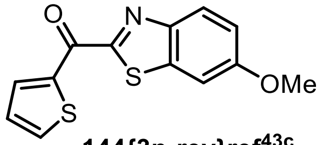 144{3p-rev}ref[43c]			
**^{13}C, ppm**	**^{13}C, ppm**	Δ	**ppm** (offset by -1.01)	Δ
175.85	176.90	1.05	175.89	-0.04
163.02	164.00	0.98	162.99	0.03
158.71	159.70	0.99	158.69	0.02
147.33	148.30	0.97	147.29	0.04
138.83	139.80	0.97	138.79	0.04
138.08	139.10	1.02	138.09	-0.01
136.00	137.00	1.00	135.99	0.01
135.38	136.40	1.02	135.39	-0.01
127.35	128.40	1.05	127.39	-0.04
125.28	126.30	1.02	125.29	-0.01
116.65	117.70	1.05	116.69	-0.04
102.48	103.50	1.02	102.49	-0.01
54.85	55.90	1.05	54.89	-0.04
	**rmsd=1.02ppm**		**rmsd=0.03ppm**	

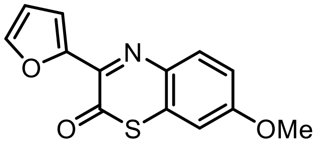 138{3q}	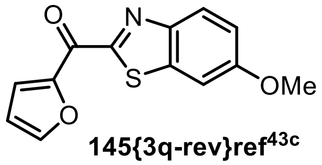 145{3q-rev}ref[43c]			
**^{13}C, ppm**	**^{13}C, ppm**	Δ	**ppm** (offset by -1.00)	Δ
171.05	172.10	1.05	171.10	-0.05
162.44	163.40	0.96	162.40	0.04
158.70	159.70	1.00	158.70	0.00
148.90	149.90	1.00	148.90	0.00
147.61	148.60	0.99	147.60	0.01
147.41	148.40	0.99	147.40	0.01
137.90	138.90	1.00	137.90	0.00
125.22	126.20	0.98	125.20	0.02
123.45	124.50	1.05	123.50	-0.05
116.74	117.70	0.96	116.70	0.04
111.80	112.80	1.00	111.80	0.00
102.44	103.40	0.96	102.40	0.04
54.85	55.90	1.05	54.90	-0.05
	**rmsd=1.0ppm**		**rmsd=0.03ppm**	

Originally assigned (incorrect) structure 139{3r} ($CDCl_3$)

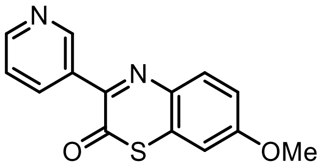

	Conf1	Conf2	Conf3	Conf4
Rel energy (kcal/mol):	0.00	0.21	0.34	0.59

C-nom	iGau	Exp	Calc	diff	1	2	3	4
C-C	9	185.15	177.37	-7.78	[177.34	177.10	177.69	177.45]
C-C	4	164.74	160.87	-3.87	[160.93	160.92	160.78	160.76]
C-C	8	161.28	144.40	-16.88	[144.16	144.48	144.46	144.82]
C-C	2	149.65	132.82	-16.83	[133.25	133.22	132.07	132.04]
C-C	12	140.46	131.13	-9.33	[130.80	131.58	130.83	131.60]
C-C	1	132.18	128.31	-3.87	[128.29	128.41	128.21	128.35]
C-CH	13	154.75	151.11	-3.64	[150.92	151.41	150.81	151.51]
C-CH	15	153.38	150.78	-2.60	[150.73	150.86	150.72	150.85]
C-CH	17	139.53	136.95	-2.58	[137.13	136.66	137.22	136.63]
C-CH	6	127.83	136.53	8.70	[136.27	136.03	137.28	137.02]
C-CH	16	124.52	122.05	-2.47	[121.60	122.72	121.58	122.73]
C-CH	5	119.20	114.99	-4.21	[117.60	117.52	110.34	110.25]
C-CH	3	104.55	108.19	3.64	[105.86	105.87	112.42	112.42]
C-CH3	20	57.06	56.38	-0.68	[56.41	56.42	56.32	56.31]

13C chem shifts: RMSD=7.95ppm (MAE=6.22) N=14 {-16.88 8.70}

Fractions: 0.380 0.265 0.214 0.141

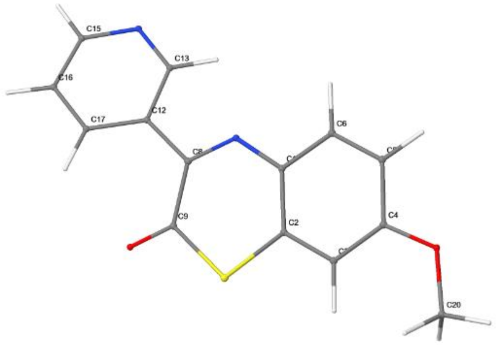

Conformer 1
Energy: -1197.62431 Hartree (Rel: 0.0 kcal/mol)
XYZ coordinates for conf 1:

C	-0.90843	0.71295	-0.06286
C	-1.65499	-0.48390	0.02404
C	-3.05545	-0.47184	0.02090
C	-3.73136	0.74861	-0.06607
C	-3.00368	1.95813	-0.15202
C	-1.62757	1.93250	-0.15130
N	0.46101	0.79957	-0.08112
C	1.30205	-0.19139	-0.01077
C	0.92025	-1.62587	0.11815
S	-0.83420	-2.03334	0.14042
O	1.69812	-2.55922	0.21780
C	2.74962	0.15360	-0.01979
C	3.14392	1.42022	0.45460
N	4.40326	1.86219	0.47139
C	5.35210	1.03872	-0.00203
C	5.08045	-0.23686	-0.49322
C	3.76295	-0.69102	-0.49989
H	6.37169	1.41968	0.01536
O	-5.07678	0.87769	-0.07489
C	-5.88352	-0.29769	0.00998
H	-3.59680	-1.40813	0.08853

```
H         -3.55429        2.89039       -0.21857
H         -1.05065        2.84905       -0.21904
H          2.38592        2.09581        0.84202
H          5.88441       -0.86363       -0.86650
H          3.52570       -1.67805       -0.87389
H         -6.91614        0.05130       -0.01312
H         -5.70215       -0.96366       -0.84144
H         -5.69913       -0.83642        0.94649

Conformer 2
Energy: -1197.62377 Hartree (Rel:  0.2 kcal/mol)
XYZ coordinates for conf 2:
C         -0.91317        0.71908       -0.04167
C         -1.65685       -0.48062        0.02738
C         -3.05729       -0.47251        0.01315
C         -3.73616        0.74679       -0.06651
C         -3.01137        1.95924       -0.13330
C         -1.63520        1.93742       -0.12208
N          0.45620        0.80892       -0.05095
C          1.30029       -0.17982        0.00923
C          0.92188       -1.61571        0.13087
S         -0.83188       -2.02806        0.13870
O          1.70052       -2.54691        0.23810
C          2.74702        0.17477       -0.00148
C          3.75968       -0.67395       -0.48625
N          5.05474       -0.33621       -0.53789
C          5.40201        0.87767       -0.09156
C          4.48262        1.80307        0.40701
C          3.14025        1.44698        0.44699
H          6.46223        1.12141       -0.13509
O         -5.08182        0.87208       -0.08530
C         -5.88565       -0.30669       -0.02214
H         -3.59645       -1.41097        0.06679
H         -3.56417        2.89057       -0.19455
H         -1.06068        2.85642       -0.17635
H          3.51404       -1.66334       -0.85339
H          4.81488        2.77580        0.75646
H          2.38727        2.13299        0.81974
H         -6.91909        0.03938       -0.05138
H         -5.69369       -0.96208       -0.87942
H         -5.70880       -0.85579        0.90980

Conformer 3
Energy: -1197.62337 Hartree (Rel:  0.3 kcal/mol)
XYZ coordinates for conf 3:
C          0.95918       -0.49006       -0.04320
C          1.59490        0.77496        0.03903
C          2.98294        0.87958        0.04107
C          3.77426       -0.27470       -0.03509
C          3.16493       -1.54430       -0.11524
C          1.78400       -1.63407       -0.11969
N         -0.39737       -0.70167       -0.06774
C         -1.32334        0.21017       -0.00763
C         -1.07226        1.67547        0.11612
S          0.63466        2.24561        0.14140
O         -1.93485        2.53175        0.20957
C         -2.73445       -0.26236       -0.02298
C         -3.01792       -1.55550        0.45918
N         -4.23288       -2.10792        0.47104
C         -5.24777       -1.37606       -0.01561
C         -5.08720       -0.08523       -0.51573
C         -3.81542        0.48454       -0.51732
H         -6.22947       -1.84621       -0.00200
O          5.11048       -0.06615       -0.02474
C          5.98583       -1.19259       -0.09892
H          3.47488        1.84511        0.10358
H          3.76181       -2.44596       -0.17471
H          1.29286       -2.59974       -0.18362
H         -2.20567       -2.15776        0.85751
H         -5.94095        0.46435       -0.89990
H         -3.66416        1.48574       -0.89831
```

H	6.99537	-0.78144	-0.07587
H	5.84339	-1.86214	0.75683
H	5.83790	-1.74846	-1.03167

Conformer 4
Energy: -1197.62397 Hartree (Rel: 0.6 kcal/mol)
XYZ coordinates for conf 4:

C	0.96403	-0.49587	-0.02420
C	1.59670	0.77176	0.03858
C	2.98448	0.88014	0.02878
C	3.77851	-0.27266	-0.03983
C	3.17224	-1.54482	-0.09989
C	1.79151	-1.63839	-0.09278
N	-0.39225	-0.71063	-0.03831
C	-1.32086	0.19881	0.01179
C	-1.07319	1.66522	0.12659
S	0.63266	2.24002	0.13511
O	-1.93635	2.51937	0.22752
C	-2.73036	-0.28300	-0.00321
C	-3.81037	0.46685	-0.50524
N	-5.06999	0.01521	-0.56150
C	-5.31189	-1.21945	-0.10274
C	-4.31804	-2.05403	0.41295
C	-3.01278	-1.57992	0.45767
H	-6.34600	-1.55663	-0.15024
O	5.11422	-0.06043	-0.04229
C	5.99196	-1.18524	-0.11183
H	3.47403	1.84775	0.07605
H	3.77129	-2.44542	-0.15324
H	1.30292	-2.60620	-0.14231
H	-3.65016	1.46957	-0.88330
H	-4.56556	-3.04838	0.77173
H	-2.20492	-2.19234	0.84349
H	7.00056	-0.77125	-0.10214
H	5.85893	-1.84580	0.75240
H	5.83736	-1.75160	-1.03717

Revised structure of 139{3r}, i.e. 146{3r-rev} ($CDCl_3$)

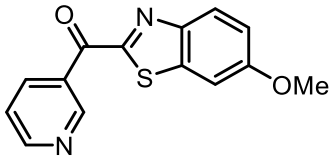

	Conf1	Conf2	Conf3	Conf4
Rel energy (kcal/mol):	0.00	0.03	0.71	0.71

C-nom	iGau	Exp	Calc	diff		1	2	3	4	
C-C	9	185.15	184.05	-1.10	[	183.85	184.18	183.99	184.34	]
C-C	8	164.74	164.55	-0.19	[	164.66	164.27	165.01	164.62	]
C-C	4	161.28	159.57	-1.71	[	159.65	159.71	159.20	159.26	]
C-C	1	149.65	147.32	-2.33	[	147.20	147.30	147.53	147.61	]
C-C	2	140.46	139.20	-1.26	[	139.42	139.40	138.51	138.49	]
C-C	11	132.18	130.18	-2.00	[	129.66	130.76	129.58	130.71	]
C-CH	13	154.75	154.13	-0.62	[	153.58	154.68	153.63	154.70	]
C-CH	15	153.38	154.62	1.24	[	154.66	154.58	154.68	154.55	]
C-CH	17	139.53	139.68	0.15	[	140.19	139.13	140.21	139.19	]
C-CH	6	127.83	127.77	-0.06	[	127.51	127.77	128.07	128.36	]
C-CH	16	124.52	122.54	-1.98	[	122.33	122.77	122.28	122.77	]
C-CH	5	119.20	116.93	-2.27	[	118.70	118.73	111.12	111.16	]
C-CH	3	104.55	103.29	-1.26	[	101.76	101.68	108.44	108.35	]
C-CH3	19	57.06	56.14	-0.92	[	56.16	56.15	56.08	56.09	]

13C chem shifts: RMSD=1.43ppm (MAE=1.22) N=14 {-2.33 1.24}

Fractions: 0.392 0.372 0.118 0.118

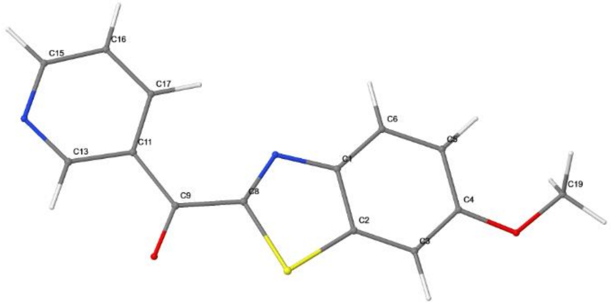

Conformer 1
Energy: -1197.63331 Hartree (Rel: 0.0 kcal/mol)
XYZ coordinates for conf 1:

C	-1.20449	0.74887	0.00010
C	-1.88221	-0.49784	-0.00003
C	-3.27918	-0.57983	-0.00004
C	-3.99813	0.61808	0.00007
C	-3.33099	1.87151	0.00020
C	-1.95422	1.94339	0.00021
N	0.16791	0.66898	0.00010
C	0.57245	-0.56787	-0.00003
C	1.96587	-1.08905	-0.00003
S	-0.71632	-1.79581	-0.00015
C	3.15554	-0.18635	-0.00003
O	2.10599	-2.31238	-0.00003
C	4.42134	-0.80291	0.00036
N	5.57948	-0.13958	0.00038
C	5.51630	1.20211	0.00000
C	4.31945	1.91813	-0.00040
C	3.11619	1.21643	-0.00041
O	-5.35219	0.69627	0.00008
C	-6.10704	-0.51372	-0.00011
H	-3.77680	-1.54139	-0.00014
H	-3.94067	2.76897	0.00029
H	-1.44095	2.89959	0.00031
H	4.47768	-1.88791	0.00066

```
H          6.47080        1.72540        0.00002
H          4.33065        3.00346       -0.00069
H          2.16532        1.73385       -0.00070
H         -7.15432       -0.20974       -0.00013
H         -5.89897       -1.11078       -0.89594
H         -5.89907       -1.11100        0.89560

Conformer 2
Energy: -1197.63444 Hartree (Rel:  0.0 kcal/mol)
XYZ coordinates for conf 2:
C         -1.19915        0.74441        0.00793
C         -1.87978       -0.50084       -0.00215
C         -3.27688       -0.57955       -0.00399
C         -3.99284        0.62017        0.00440
C         -3.32281        1.87206        0.01458
C         -1.94593        1.94081        0.01643
N          0.17271        0.66158        0.00860
C          0.57486       -0.57559       -0.00083
C          1.96627       -1.09867       -0.00143
S         -0.71651       -1.80113       -0.01052
C          3.16417       -0.20474       -0.00083
O          2.10494       -2.32235       -0.00132
C          3.13085        1.20040       -0.02902
N          4.23013        1.96632       -0.03097
C          5.41861        1.34803       -0.00407
C          5.56875       -0.04105        0.02458
C          4.42391       -0.82647        0.02562
O         -5.34677        0.70149        0.00381
C         -6.10438       -0.50662       -0.00707
H         -3.77695       -1.53982       -0.01159
H         -3.93050        2.77085        0.02081
H         -1.42998        2.89549        0.02420
H          2.17997        1.72010       -0.05038
H          6.29451        1.99464       -0.00560
H          6.55800       -0.48711        0.04536
H          4.47660       -1.90964        0.04650
H         -7.15096       -0.20020       -0.00673
H         -5.89602       -1.09734       -0.90704
H         -5.89955       -1.11127        0.88444

Conformer 3
Energy: -1197.63439 Hartree (Rel:  0.7 kcal/mol)
XYZ coordinates for conf 3:
C          1.24542       -0.51148        0.00068
C          1.82357        0.78915        0.00011
C          3.20288        0.97012       -0.00058
C          4.02135       -0.16674       -0.00054
C          3.45973       -1.46667        0.00004
C          2.08402       -1.63766        0.00065
N         -0.13054       -0.53958        0.00111
C         -0.62976        0.66098        0.00093
C         -2.06164        1.06875        0.00097
S          0.55570        1.99124        0.00013
C         -3.17550        0.07443        0.00037
O         -2.29829        2.27688        0.00131
C         -4.48636        0.58852       -0.00164
N         -5.58809       -0.16474       -0.00247
C         -5.41859       -1.49713       -0.00128
C         -4.16864       -2.11585        0.00075
C         -3.02492       -1.32084        0.00158
O          5.35643        0.07952       -0.00117
C          6.26357       -1.02112       -0.00072
H          3.66163        1.95278       -0.00106
H          4.10189       -2.33877        0.00001
H          1.64745       -2.63131        0.00102
H         -4.62886        1.66556       -0.00263
H         -6.32848       -2.09464       -0.00196
H         -4.09375       -3.19865        0.00161
H         -2.03591       -1.76093        0.00313
H          7.26099       -0.57998       -0.00094
H          6.13923       -1.64040        0.89534
```

H	6.13919	-1.64118	-0.89623

Conformer 4
Energy: -1197.63331 Hartree (Rel: 0.7 kcal/mol)
XYZ coordinates for conf 4:

C	-1.24015	-0.50720	-0.00517
C	-1.82106	0.79235	0.00379
C	-3.20075	0.97033	0.00574
C	-4.01662	-0.16843	-0.00135
C	-3.45227	-1.46716	-0.01030
C	-2.07627	-1.63530	-0.01225
N	0.13542	-0.53269	-0.00624
C	0.63265	0.66827	0.00184
C	2.06273	1.07786	0.00207
S	-0.55553	1.99665	0.01066
C	3.18531	0.09165	0.00034
O	2.29815	2.28646	0.00258
C	3.04030	-1.30650	0.02735
N	4.07507	-2.15748	0.02826
C	5.30895	-1.63571	0.00145
C	5.56923	-0.26294	-0.02612
C	4.49059	0.61115	-0.02607
O	-5.35227	0.07488	0.00100
C	-6.25684	-1.02788	-0.00575
H	-3.66179	1.95189	0.01248
H	-4.09255	-2.34061	-0.01576
H	-1.63716	-2.62777	-0.01915
H	2.05102	-1.74869	0.04855
H	6.13066	-2.34989	0.00215
H	6.59078	0.10314	-0.04684
H	4.62945	1.68667	-0.04604
H	-7.25529	-0.58909	-0.00227
H	-6.13148	-1.64132	-0.90567
H	-6.13051	-1.65319	0.88582

Originally assigned correct structure 148{1b} ($CDCl_3$)

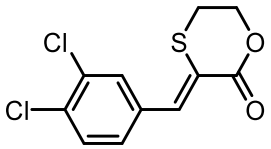

					Conf1	Conf2
	Rel energy (kcal/mol):				0.00	0.01

C-nom	iGau	Exp	Calc	diff	1	2
C	11	163.00	161.53	-1.47	[161.51	161.55]
C	5	134.00	133.31	-0.69	[133.16	133.46]
C	9	133.80	133.29	-0.51	[133.20	133.38]
C	2	133.00	133.28	0.28	[133.28	133.28]
C	6	132.00	132.40	0.40	[130.14	134.70]
C	1	131.00	131.93	0.93	[132.19	131.66]
C	4	130.00	130.79	0.79	[133.28	128.27]
C	3	129.00	129.42	0.42	[129.24	129.61]
C	10	122.00	126.05	4.05	[125.97	126.13]
C	13	68.00	66.88	-1.12	[66.92	66.84]
C	14	26.00	24.72	-1.28	[24.70	24.74]

13C chem shifts: RMSD=1.48ppm (MAE=1.09) N=11 {-1.47 4.05}

Fractions: 0.503 0.497

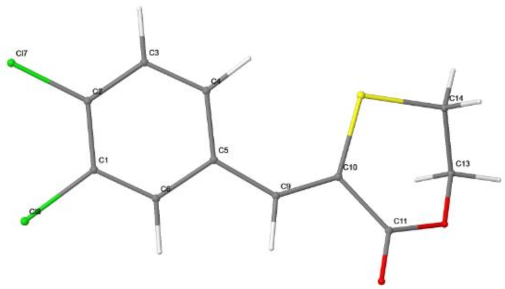

Conformer 1
Energy: -1892.98994 Hartree (Rel: 0.0 kcal/mol)
XYZ coordinates for conf 1:

C	2.38532	-0.50721	0.11476
C	3.20861	0.59752	-0.13787
C	2.63170	1.86414	-0.26978
C	1.25787	2.01904	-0.15700
C	0.40632	0.91752	0.07975
C	1.00452	-0.34937	0.22288
Cl	4.94102	0.43716	-0.28459
Cl	3.05628	-2.11233	0.31038
C	-1.02313	1.19345	0.16744
C	-2.11856	0.38859	0.16877
C	-3.43703	1.10939	0.26459
O	-4.56407	0.50737	-0.17786
C	-4.50382	-0.71982	-0.92788
C	-3.81436	-1.80208	-0.12615
S	-2.04468	-1.38791	0.10024
O	-3.53442	2.22662	0.73155
H	3.26950	2.72070	-0.45838
H	0.82803	3.01082	-0.26005
H	0.41525	-1.22832	0.44512
H	-1.26421	2.25143	0.22445
H	-5.54776	-0.97835	-1.11750
H	-4.00162	-0.54597	-1.88555
H	-3.83469	-2.75501	-0.66156
H	-4.30037	-1.92997	0.84480

Conformer 2
Energy: -1892.98992 Hartree (Rel: 0.0 kcal/mol)
XYZ coordinates for conf 2:

C	2.78112	0.65354	-0.08677
C	3.06305	-0.71211	0.06226
C	2.01438	-1.60462	0.29372
C	0.70063	-1.15445	0.36544
C	0.39361	0.21251	0.20516
C	1.46727	1.10181	-0.00952
Cl	4.69555	-1.32543	-0.02405
Cl	4.05354	1.82157	-0.36667
C	-0.93964	0.80259	0.25365
C	-2.18429	0.25837	0.20919
C	-3.31433	1.25117	0.27659
O	-4.52891	0.91875	-0.21604
C	-4.71089	-0.28222	-0.98836
C	-4.30944	-1.49946	-0.18407
S	-2.50172	-1.49048	0.11281
O	-3.18028	2.35592	0.76346
H	2.23685	-2.65814	0.42334
H	-0.07463	-1.87974	0.57420
H	1.27103	2.16253	-0.12554
H	-0.94441	1.88720	0.31863
H	-5.77763	-0.30118	-1.22126
H	-4.14463	-0.21228	-1.92326
H	-4.51968	-2.41802	-0.73842
H	-4.84926	-1.52715	0.76634

Originally assigned correct structure of 149{1f} ($CDCl_3$)

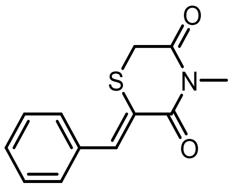

					Conf1	Conf2
		Rel energy (kcal/mol):			0.00	0.00

C-nom	iGau	Exp	Calc	diff	1	2
C-C	6	167.40	168.37	0.97	[168.37	168.36]
C-C	4	163.90	165.41	1.51	[165.41	165.41]
C-C	11	134.00	131.52	-2.48	[131.52	131.52]
C-C	3	121.10	122.16	1.06	[122.16	122.16]
C-CH	10	140.70	142.34	1.64	[142.34	142.34]
C-CH	12	130.60	132.38	1.78	[132.38	132.39]
C-CH	16	130.60	132.38	1.78	[132.38	132.39]
C-CH	14	129.90	130.45	0.55	[130.45	130.45]
C-CH	13	128.50	127.44	-1.06	[127.44	127.44]
C-CH	15	128.50	127.44	-1.06	[127.44	127.44]
C-CH2	1	31.00	29.87	-1.13	[29.87	29.87]
C-CH3	7	28.70	29.01	0.31	[29.01	29.02]

13C chem shifts: RMSD=1.40ppm (MAE=1.28) N=12 {-2.48 1.78}

Fractions: 0.500 0.500

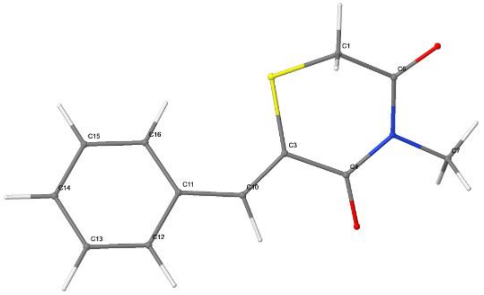

Conformer 1
Energy: -1067.29155 Hartree (Rel: 0.0 kcal/mol)
XYZ coordinates for conf 1:

C	2.05119	-1.66303	-0.74301
S	0.52603	-1.51820	0.24494
C	0.32008	0.23530	0.07253
C	1.53598	1.11420	0.07026
N	2.82315	0.54138	0.10858
C	3.16581	-0.76327	-0.25302
C	3.92168	1.47596	0.40507
O	4.32188	-1.15311	-0.23205
O	1.44538	2.33576	0.08967
C	-0.87613	0.87650	0.01967
C	-2.25319	0.39562	0.03445
C	-3.24405	1.29547	-0.41890
C	-4.58534	0.92958	-0.46766
C	-4.97712	-0.34337	-0.04239
C	-4.01649	-1.23843	0.43536
C	-2.67086	-0.87742	0.47683
H	2.39699	-2.69440	-0.66713
H	1.83479	-1.44280	-1.79443
H	3.66143	2.07127	1.28071
H	4.81643	0.88782	0.59252
H	4.08796	2.14853	-0.44086
H	-0.77876	1.95640	-0.06718
H	-2.94397	2.28793	-0.74518
H	-5.32563	1.63731	-0.82971

H	-6.02432	-0.63101	-0.07151
H	-4.31637	-2.22175	0.78649
H	-1.95448	-1.58172	0.88114

Conformer 2
Energy: -1067.29155 Hartree (Rel: 0.0 kcal/mol)
XYZ coordinates for conf 2:

C	-2.05160	-1.66300	0.74292
S	-0.52634	-1.51836	-0.24500
C	-0.32008	0.23511	-0.07239
C	-1.53587	1.11420	-0.06990
N	-2.82307	0.54146	-0.10896
C	-3.16600	-0.76302	0.25284
C	-3.92158	1.47598	-0.40561
O	-4.32221	-1.15243	0.23185
O	-1.44511	2.33575	-0.08861
C	0.87620	0.87614	-0.01936
C	2.25329	0.39535	-0.03426
C	3.24418	1.29562	0.41819
C	4.58555	0.93000	0.46669
C	4.97739	-0.34315	0.04208
C	4.01671	-1.23869	-0.43470
C	2.67102	-0.87794	-0.47590
H	-2.39759	-2.69430	0.66699
H	-1.83513	-1.44282	1.79433
H	-3.66010	2.07297	-1.27972
H	-4.81571	0.88769	-0.59567
H	-4.08987	2.14694	0.44121
H	0.77889	1.95603	0.06777
H	2.94406	2.28823	0.74399
H	5.32585	1.63808	0.82804
H	6.02464	-0.63059	0.07097
H	4.31662	-2.22221	-0.78526
H	1.95460	-1.58270	-0.87934

Originally assigned correct structure of 152{3b} ($CDCl_3$)

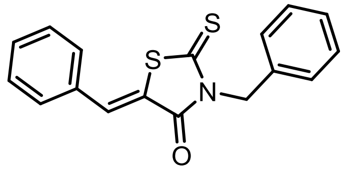

	Conf1
Rel energy (kcal/mol):	0.00

C-nom	iGau	Exp	Calc	diff	1
C-C	5	193.10	191.98	-1.12	[191.98]
C-C	3	167.80	168.33	0.53	[168.33]
C-C	16	133.30	133.26	-0.04	[133.26]
C-C	8	130.70	129.70	-1.00	[129.70]
C-C	2	122.90	121.64	-1.26	[121.64]
C-CH	7	134.80	137.14	2.34	[137.14]
C-CH	11	133.20	132.33	-0.87	[132.33]
C-CH	13	130.60	133.05	2.45	[133.05]
C-CH	9	130.60	133.05	2.45	[133.05]
C-CH	17	129.30	130.38	1.08	[130.38]
C-CH	21	129.30	130.38	1.08	[130.38]
C-CH	10	128.90	128.32	-0.58	[128.32]
C-CH	12	128.90	128.32	-0.58	[128.32]
C-CH	20	128.50	127.31	-1.19	[127.31]
C-CH	18	128.50	127.31	-1.19	[127.31]
C-CH	19	128.10	127.89	-0.21	[127.89]
C-CH2	15	47.50	48.99	1.49	[48.99]

13C chem shifts: RMSD=1.34ppm (MAE=1.14) N=17 {-1.26 2.45}

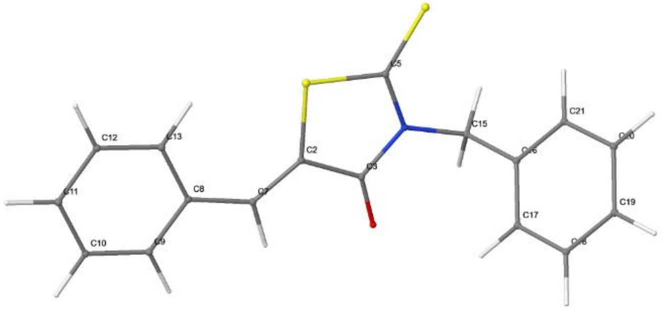

Conformer 1
Energy: -1581.99599 Hartree (Rel: 0.0 kcal/mol)
XYZ coordinates for conf 1:

S	0.89919	1.59622	-0.19984
C	1.13993	-0.02604	0.45692
C	-0.13071	-0.52158	1.03274
N	-1.15256	0.43956	0.88014
C	-0.80961	1.61937	0.27753
S	-1.77351	2.93712	0.00532
C	2.24970	-0.80348	0.49906
C	3.60639	-0.58699	0.02575
C	4.54213	-1.62078	0.25164
C	5.86332	-1.49973	-0.16710
C	6.28261	-0.33988	-0.82466
C	5.37087	0.69472	-1.05857
C	4.04883	0.57654	-0.63997
O	-0.31207	-1.60009	1.57537
C	-2.51052	0.14817	1.39323
C	-3.42591	-0.48664	0.36304
C	-3.28057	-1.83950	0.02053
C	-4.13233	-2.42644	-0.91596
C	-5.14361	-1.67100	-1.51646
C	-5.29891	-0.32612	-1.17508

C	-4.44329	0.26285	-0.24093
H	2.07611	-1.76435	0.98159
H	4.21749	-2.52324	0.76272
H	6.56515	-2.30758	0.01806
H	7.31308	-0.24148	-1.15373
H	5.69188	1.59750	-1.56984
H	3.36681	1.39576	-0.83816
H	-2.35816	-0.52222	2.24133
H	-2.92162	1.09239	1.75250
H	-2.49955	-2.42849	0.49243
H	-4.01083	-3.47557	-1.17190
H	-5.80871	-2.12988	-2.24306
H	-6.08515	0.26668	-1.63461
H	-4.56293	1.31015	0.02068

Originally assigned (incorrect) structure of 150{1g} ($CDCl_3$)

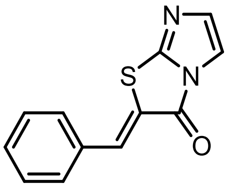

					Conf1
	Rel energy (kcal/mol):				0.00
**C-nom**	**iGau**	**Exp**	**Calc**	**diff**	**1**
C-C	8	158.10	160.36	2.26	[160.36]
C-C	4	152.50	143.61	-8.89	[143.61]
C-C	11	140.30	129.69	-10.61	[129.69]
C-C	7	134.80	119.28	-15.52	[119.28]
C-CH	10	131.70	141.04	9.34	[141.04]
C-CH	1	131.60	135.29	3.69	[135.29]
C-CH	12	129.50	132.55	3.05	[132.55]
C-CH	16	129.50	132.55	3.05	[132.55]
C-CH	14	126.90	132.12	5.22	[132.12]
C-CH	13	126.90	128.24	1.34	[128.24]
C-CH	15	114.70	128.24	13.54	[128.24]
C-CH	2	112.00	113.87	1.87	[113.87]

13C chem shifts: RMSD=8.03ppm (MAE=6.53) N=12 {-15.52 13.54}

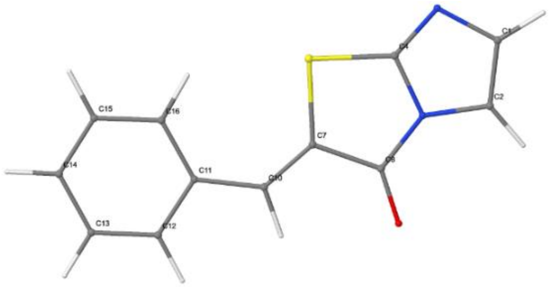

Conformer 1
Energy: -1044.98731 Hartree (Rel: 0.0 kcal/mol)
XYZ coordinates for conf 1:

C	4.58019	-0.54660	-0.00023
C	4.03575	0.70702	0.00050
N	3.59658	-1.54287	-0.00069
C	2.47876	-0.87538	-0.00053
N	2.65981	0.48980	0.00007
S	0.77720	-1.33926	-0.00089
C	0.31546	0.39044	0.00009
C	1.50986	1.29024	0.00019
O	1.51761	2.50701	0.00113
C	-0.92113	0.94121	0.00043
C	-2.25416	0.36004	0.00036
C	-3.34747	1.25425	-0.00122
C	-4.65852	0.78862	-0.00156
C	-4.90980	-0.58623	-0.00019
C	-3.84076	-1.48779	0.00156
C	-2.52807	-1.02449	0.00184
H	5.62848	-0.81174	-0.00030
H	4.45703	1.69944	0.00110
H	-0.89445	2.03035	0.00063
H	-3.15399	2.32365	-0.00225
H	-5.48308	1.49543	-0.00286
H	-5.93176	-0.95427	-0.00043
H	-4.03079	-2.55707	0.00276
H	-1.72297	-1.75018	0.00348

Revised structure of 150{1g}, i.e imidazothiazinone 154{1g-rev} ($CDCl_3$)

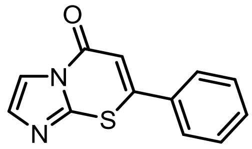

					Conf1
Rel energy (kcal/mol):					0.00
**C-nom**	**iGau**	**Exp**	**Calc**	**diff**	**1**
C-C	8	158.10	156.99	-1.11	[156.99]
C-C	10	152.50	152.11	-0.39	[152.11]
C-C	4	140.30	139.55	-0.75	[139.55]
C-C	11	134.80	134.65	-0.15	[134.65]
C-CH	14	131.70	131.59	-0.11	[131.59]
C-CH	1	131.60	130.05	-1.55	[130.05]
C-CH	13	129.50	128.56	-0.94	[128.56]
C-CH	15	129.50	128.56	-0.94	[128.56]
C-CH	12	126.90	127.34	0.44	[127.34]
C-CH	16	126.90	127.34	0.44	[127.34]
C-CH	2	114.70	115.09	0.39	[115.09]
C-CH	7	112.00	111.08	-0.92	[111.08]

13C chem shifts: RMSD=0.79ppm (MAE=0.68) N=12 {-1.55 0.44}

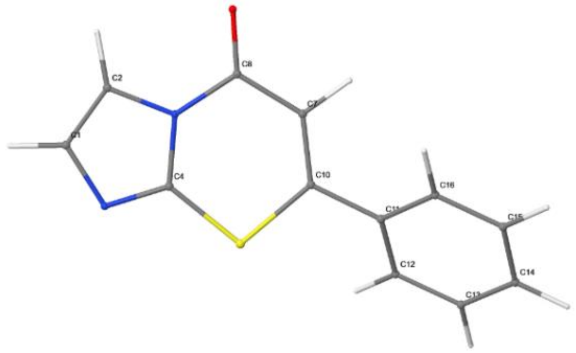

Conformer 1
Energy: -1044.99611 Hartree (Rel: 0.0 kcal/mol)
XYZ coordinates for conf 1:

C	-4.17560	-0.93576	0.11500
C	-3.83572	0.37654	-0.06054
N	-3.05100	-1.73585	0.23609
C	-2.04147	-0.90711	0.13242
N	-2.44656	0.40425	-0.04863
S	-0.36422	-1.38942	0.25564
C	-0.19856	1.32537	-0.21074
C	-1.63271	1.55446	-0.21911
O	-2.15974	2.64743	-0.37564
C	0.45180	0.14403	-0.02641
C	1.93085	0.04757	-0.01775
C	2.59491	-1.03908	-0.61437
C	3.98768	-1.10489	-0.60816
C	4.73681	-0.09308	-0.00361
C	4.08570	0.98781	0.59710
C	2.69419	1.05898	0.59308
H	-5.16753	-1.36375	0.16203
H	-4.40499	1.28113	-0.19611
H	2.02410	-1.81931	-1.10901
H	4.48636	-1.94486	-1.08268
H	5.82159	-0.14736	0.00214
H	4.66130	1.77275	1.07889
H	2.19316	1.88631	1.08573
H	0.38704	2.21838	-0.39982

Originally assigned (incorrect) structure of 151{1h} ($CDCl_3$)

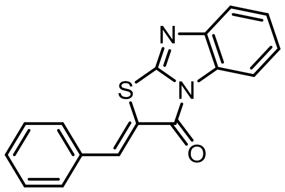

					Conf1
	Rel energy (kcal/mol):				0.00

C-nom	iGau	Exp	Calc	diff	1
C-C	10	159.90	160.54	0.64	[160.54]
C-C	8	150.60	150.06	-0.54	[150.06]
C-C	4	146.70	147.68	0.98	[147.68]
C-C	5	142.50	129.31	-13.19	[129.31]
C-C	15	134.50	129.73	-4.77	[129.73]
C-C	12	130.90	118.45	-12.45	[118.45]
C-CH	14	131.70	139.91	8.21	[139.91]
C-CH	16	129.50	132.38	2.88	[132.38]
C-CH	20	129.50	132.38	2.88	[132.38]
C-CH	18	126.80	131.99	5.19	[131.99]
C-CH	17	126.80	128.27	1.47	[128.27]
C-CH	19	125.90	128.27	2.37	[128.27]
C-CH	2	124.30	125.46	1.16	[125.46]
C-CH	1	118.60	123.99	5.39	[123.99]
C-CH	3	116.10	119.76	3.66	[119.76]
C-CH	6	113.70	113.07	-0.63	[113.07]

13C chem shifts: RMSD=5.68ppm (MAE=4.15) N=16 {-13.19 8.21}

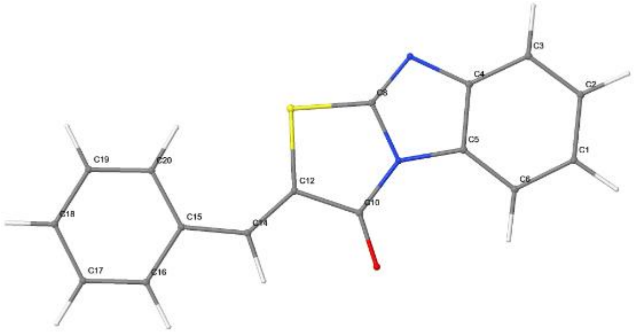

Conformer 1
Energy: -1198.64024 Hartree (Rel: 0.0 kcal/mol)
XYZ coordinates for conf 1:

C	-5.18445	1.22437	0.00032
C	-5.67579	-0.09297	-0.00050
C	-4.81702	-1.19430	-0.00103
C	-3.44278	-0.94859	-0.00072
C	-2.96982	0.38669	0.00011
C	-3.81254	1.49218	0.00064
N	-2.37443	-1.86564	-0.00112
C	-1.32026	-1.11136	-0.00055
N	-1.57677	0.25406	0.00021
C	-0.48143	1.11601	0.00086
O	-0.55887	2.33243	0.00157
C	0.76546	0.29010	0.00060
S	0.40333	-1.46395	-0.00053
C	1.96847	0.90911	0.00085
C	3.33300	0.40487	0.00055
C	4.37319	1.36018	-0.00217
C	5.70886	0.97059	-0.00291
C	6.03892	-0.38749	-0.00075
C	5.02333	-1.34877	0.00222
C	3.68613	-0.96149	0.00286

H	-5.88492	2.05421	0.00071
H	-6.74956	-0.25666	-0.00073
H	-5.19578	-2.21143	-0.00167
H	-3.42147	2.50292	0.00127
H	1.87932	1.99486	0.00106
H	4.11851	2.41674	-0.00380
H	6.49138	1.72370	-0.00511
H	7.08033	-0.69623	-0.00128
H	5.27423	-2.40544	0.00409
H	2.92446	-1.73276	0.00558

Revised structure of 151{1h}, i.e enamide 155{1h-rev} ($CDCl_3$)

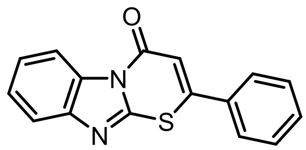

	Conf1
Rel energy (kcal/mol):	0.00

C-nom	iGau	Exp	Calc	diff	1
C-C	10	159.90	159.29	-0.61	[159.29]
C-C	14	150.60	150.18	-0.42	[150.18]
C-C	8	146.70	147.48	0.78	[147.48]
C-C	4	142.50	141.70	-0.80	[141.70]
C-C	15	134.50	134.30	-0.20	[134.30]
C-C	5	130.90	130.50	-0.40	[130.50]
C-CH	18	131.70	131.68	-0.02	[131.68]
C-CH	17	129.50	128.61	-0.89	[128.61]
C-CH	19	129.50	128.61	-0.89	[128.61]
C-CH	16	126.80	127.32	0.52	[127.32]
C-CH	20	126.80	127.32	0.52	[127.32]
C-CH	2	125.90	125.52	-0.38	[125.52]
C-CH	1	124.30	123.78	-0.52	[123.78]
C-CH	3	118.60	118.84	0.24	[118.84]
C-CH	6	116.10	116.54	0.44	[116.54]
C-CH	12	113.70	112.77	-0.93	[112.77]

13C chem shifts: RMSD=0.59ppm (MAE=0.53) N=16 {-0.93 0.78}

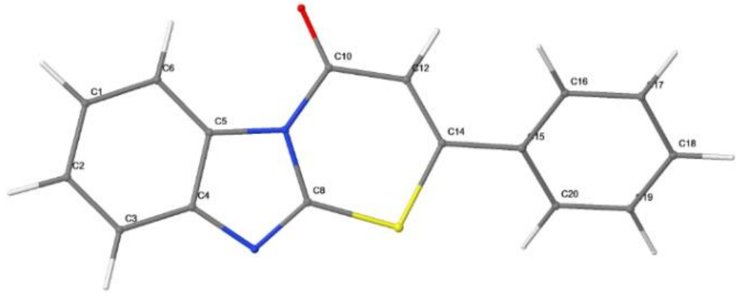

Conformer 1
Energy: -1198.64493 Hartree (Rel: 0.0 kcal/mol)
XYZ coordinates for conf 1:

C	-5.10604	0.87540	-0.11629
C	-5.43271	-0.48707	0.02862
C	-4.44373	-1.46031	0.14422
C	-3.10924	-1.04017	0.11285
C	-2.79802	0.33006	-0.03176
C	-3.78237	1.31331	-0.15041
N	-1.94760	-1.80533	0.20547
C	-0.97650	-0.94199	0.12061
N	-1.39416	0.38420	-0.02446
C	-0.58204	1.53102	-0.16357
O	-1.09497	2.63646	-0.28591
C	0.85815	1.31226	-0.16973
S	0.70740	-1.41039	0.22966
C	1.51891	0.13644	-0.01809
C	2.99772	0.04603	-0.01758
C	3.75906	1.05091	0.60650
C	5.15083	0.98684	0.60181
C	5.80403	-0.08057	-0.02045
C	5.05688	-1.08616	-0.63767
C	3.66378	-1.02761	-0.63519
H	-5.90442	1.60664	-0.20346
H	-6.47797	-0.78198	0.05026
H	-4.68594	-2.51240	0.25550

H	-3.52391	2.35744	-0.26150
H	3.25636	1.86708	1.11575
H	5.72515	1.76638	1.09377
H	6.88907	-0.12954	-0.02109
H	5.55732	-1.91572	-1.12836
H	3.09461	-1.80308	-1.13917
H	1.43117	2.21727	-0.33935

Originally assigned (incorrect) structure of 153{1i} ($CDCl_3$)

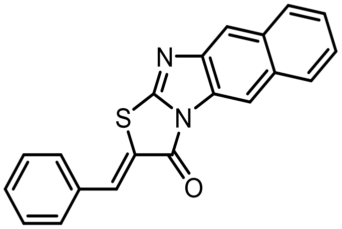

					Conf1
		Rel energy (kcal/mol):			0.00
**C-nom**	**iGau**	**Exp**	**Calc**	**diff**	**1**
C-C	9	159.60	160.00	0.40	[160.00]
C-C	11	150.30	153.22	2.92	[153.22]
C-C	14	150.00	145.73	-4.27	[145.73]
C-C	19	141.00	130.90	-10.10	[130.90]
C-C	13	134.30	130.18	-4.12	[130.18]
C-C	5	130.90	129.97	-0.93	[129.97]
C-C	18	130.30	129.49	-0.81	[129.49]
C-C	8	129.60	118.58	-11.02	[118.58]
C-CH	7	131.90	139.49	7.59	[139.49]
C-CH	4	128.60	132.01	3.41	[132.01]
C-CH	6	128.60	132.01	3.41	[132.01]
C-CH	2	128.60	131.83	3.23	[131.83]
C-CH	24	128.60	128.52	-0.08	[128.52]
C-CH	1	128.20	128.30	0.10	[128.30]
C-CH	3	126.90	128.30	1.40	[128.30]
C-CH	21	125.50	127.97	2.47	[127.97]
C-CH	22	125.30	125.49	0.19	[125.49]
C-CH	23	115.50	125.13	9.63	[125.13]
C-CH	20	114.20	118.07	3.87	[118.07]
C-CH	17	113.80	111.21	-2.59	[111.21]

13C chem shifts: RMSD=4.91ppm (MAE=3.63) N=20 {-11.02 9.63}

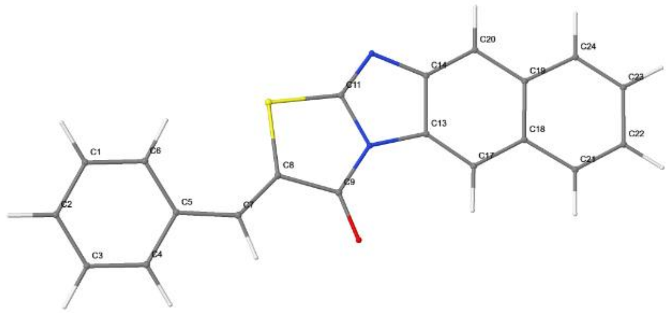

Conformer 1
Energy: -1352.28040 Hartree (Rel: 0.0 kcal/mol)
XYZ coordinates for conf 1:

C	6.22535	-1.11455	0.12978
C	7.17222	-0.10280	-0.05716
C	6.75273	1.22347	-0.19279
C	5.39665	1.53137	-0.14404
C	4.42473	0.52250	0.03205
C	4.86753	-0.80961	0.17340
C	3.02988	0.93711	0.05334
C	1.87245	0.23750	0.04296
C	0.56974	0.97269	0.05626
N	-0.45913	0.03727	0.01901
C	-0.10841	-1.30930	-0.02084
S	1.63453	-1.53680	-0.01367
C	-1.85934	0.07494	0.00878
C	-2.23961	-1.30670	-0.03721

N	-1.10106	-2.13884	-0.05434
O	0.40692	2.18088	0.09140
C	-2.76128	1.10725	0.03387
C	-4.14238	0.76583	0.01264
C	-4.54780	-0.61882	-0.03348
C	-3.57213	-1.65229	-0.05833
C	-5.14834	1.77174	0.03625
C	-6.48473	1.44151	0.01610
C	-6.88439	0.08175	-0.02901
C	-5.93959	-0.91885	-0.05321
H	6.54633	-2.14576	0.24464
H	8.23008	-0.34696	-0.09233
H	7.48189	2.01604	-0.33356
H	5.07296	2.56371	-0.24738
H	4.16056	-1.61475	0.33971
H	2.86811	2.01447	0.05941
H	-2.44422	2.14362	0.06843
H	-3.87732	-2.69381	-0.09318
H	-4.83987	2.81374	0.07085
H	-7.23869	2.22352	0.03472
H	-7.94171	-0.16772	-0.04460
H	-6.24425	-1.96195	-0.08796

Revised structure of 153{1i}, i.e enamide 156{1i-rev} (CDCl$_3$)

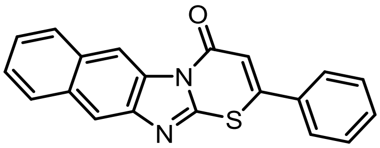

					Conf1
		Rel energy (kcal/mol):			0.00
**C-nom**	**iGau**	**Exp**	**Calc**	**diff**	**1**
C-C	9	159.60	159.18	-0.42	[159.18]
C-C	11	150.30	151.03	0.73	[151.03]
C-C	7	150.00	149.43	-0.57	[149.43]
C-C	14	141.00	139.81	-1.19	[139.81]
C-C	5	134.30	134.19	-0.11	[134.19]
C-C	13	130.90	131.49	0.59	[131.49]
C-C	19	130.30	130.92	0.62	[130.92]
C-C	18	129.60	129.55	-0.05	[129.55]
C-CH	2	131.90	131.72	-0.18	[131.72]
C-CH	1	128.60	128.64	0.04	[128.64]
C-CH	3	128.60	128.64	0.04	[128.64]
C-CH	21	128.60	128.44	-0.16	[128.44]
C-CH	24	128.60	128.13	-0.47	[128.13]
C-CH	4	128.20	127.31	-0.89	[127.31]
C-CH	6	126.90	127.31	0.41	[127.31]
C-CH	23	125.50	125.03	-0.47	[125.03]
C-CH	22	125.30	124.88	-0.42	[124.88]
C-CH	20	115.50	116.70	1.20	[116.70]
C-CH	17	114.20	114.95	0.75	[114.95]
C-CH	8	113.80	113.12	-0.68	[113.12]

13C chem shifts: RMSD=0.60ppm (MAE=0.50) N=20 {-1.19 1.20}

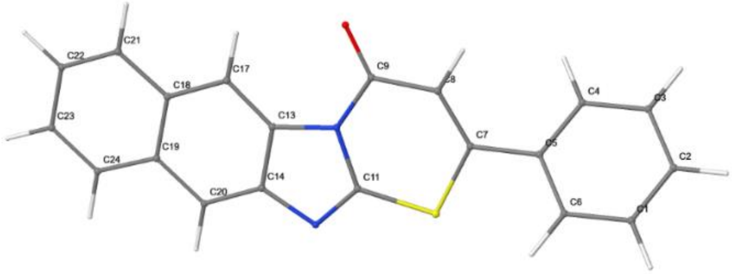

Conformer 1
Energy: -1352.28394 Hartree (Rel: 0.0 kcal/mol)
XYZ coordinates for conf 1:

C	-6.19675	-0.94527	-0.67450
C	-6.91100	0.07577	-0.04392
C	-6.22369	1.10815	0.60042
C	-4.83064	1.12197	0.61384
C	-4.10207	0.10130	-0.02370
C	-4.80248	-0.93701	-0.66340
C	-2.62134	0.13906	-0.01495
C	-1.91684	1.29109	-0.13911
C	-0.46788	1.45412	-0.12004
N	0.29833	0.27764	-0.00079
C	-0.16637	-1.03815	0.11597
S	-1.86741	-1.44391	0.20657
C	1.70288	0.17464	-0.00148
C	1.96364	-1.22450	0.11783
N	0.76576	-1.94079	0.18804
O	0.08562	2.54287	-0.21657
C	2.70516	1.11396	-0.09308
C	4.04515	0.64561	-0.06299
C	4.32549	-0.76629	0.05826

C	3.26019	-1.69619	0.14808
C	5.14002	1.55336	-0.15078
C	6.43981	1.10484	-0.12176
C	6.71582	-0.28300	-0.00262
C	5.68644	-1.19042	0.08463
H	-6.72364	-1.74787	-1.18205
H	-7.99709	0.06583	-0.05110
H	-6.77267	1.89923	1.10279
H	-4.30191	1.91040	1.14017
H	-4.25869	-1.72383	-1.17773
H	-2.45376	2.22063	-0.29379
H	2.48759	2.16995	-0.18366
H	3.46181	-2.75929	0.23884
H	4.92586	2.61540	-0.24203
H	7.26167	1.81215	-0.19010
H	7.74672	-0.62551	0.01923
H	5.89565	-2.25352	0.17592

Originally assigned (incorrect) structure of 158{10c} (Benzene-d_6)

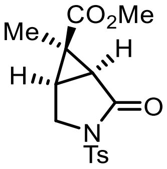

	Conf1	Conf2
Rel energy (kcal/mol):	0.00	0.12

C-nom	iGau	Exp	Calc	diff	1	2
C-C	10	171.40	168.41	-2.99	[167.90	169.04]
C-C	4	168.30	166.17	-2.13	[166.72	165.51]
C-C	18	144.90	145.35	0.45	[145.61	145.04]
C-C	15	136.20	136.17	-0.03	[136.48	135.80]
C-C	6	27.40	28.51	1.11	[29.06	27.84]
C-CH	16	129.60	129.60	0.00	[129.43	129.81]
C-CH	20	129.60	129.60	0.00	[129.43	129.81]
C-CH	17	128.70	129.13	0.43	[129.32	128.90]
C-CH	19	128.70	129.13	0.43	[129.32	128.90]
C-CH	5	34.10	34.36	0.26	[32.14	37.07]
C-CH	1	25.10	25.45	0.35	[22.78	28.69]
C-CH2	2	45.10	42.97	-2.13	[42.81	43.16]
C-CH3	14	51.90	52.14	0.24	[52.00	52.31]
C-CH3	21	21.10	20.64	-0.46	[20.60	20.68]
C-CH3	7	7.60	20.62	13.02	[20.53	20.74]

13C chem shifts: RMSD=3.56ppm (MAE=1.60) N=15 {-2.99 13.02}

Fractions: 0.549 0.451

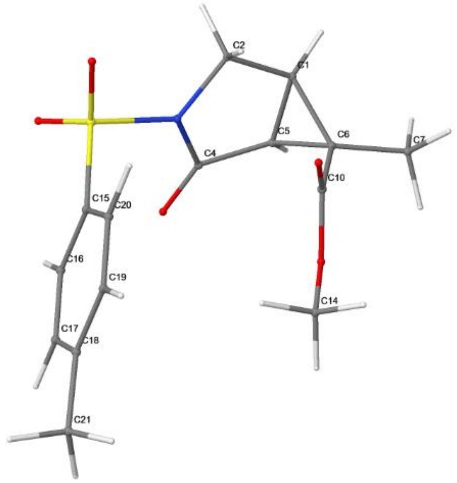

Conformer 1
Energy: -1410.80781 Hartree (Rel: 0.0 kcal/mol)
XYZ coordinates for conf 1:

C	3.22485	-0.53111	-0.29181
C	2.13358	-1.34551	-0.98008
N	1.01854	-1.30097	-0.00830
C	1.35792	-0.76908	1.24323
C	2.74123	-0.20315	1.11207
C	2.95050	0.89642	0.07435
C	4.14628	1.81482	0.30186
H	3.36303	-0.21121	2.00065
O	0.64807	-0.77362	2.23046
C	1.76487	1.62586	-0.51997
O	1.40677	1.55195	-1.67854
O	1.18722	2.42231	0.39577
H	-0.23157	3.81079	0.79109
C	0.08458	3.22439	-0.07126

C	-1.71288	-0.87460	-0.13865
C	-2.31834	-0.67484	1.10403
C	-3.32013	0.28730	1.21392
C	-3.72842	1.04448	0.10554
C	-3.09778	0.82071	-1.12878
C	-2.09225	-0.13491	-1.26196
C	-4.84290	2.05545	0.23011
S	-0.45552	-2.12704	-0.31710
O	-0.64760	-3.14463	0.71460
O	-0.34996	-2.50295	-1.73002
H	2.45988	-2.37826	-1.13434
H	1.82364	-0.91808	-1.93414
H	4.25324	-0.82533	-0.47658
H	3.89149	2.61574	1.00270
H	4.46815	2.26795	-0.64259
H	4.99036	1.25168	0.71318
H	-0.72859	2.58396	-0.42015
H	0.40812	3.87827	-0.88514
H	-2.00101	-1.25499	1.96174
H	-3.79499	0.45036	2.17782
H	-3.40300	1.39567	-1.99930
H	-1.61644	-0.31299	-2.21992
H	-5.81640	1.58723	0.03344
H	-4.88517	2.48305	1.23696
H	-4.72489	2.87376	-0.48750

Conformer 2
Energy: -1410.80762 Hartree (Rel: 0.1 kcal/mol)
XYZ coordinates for conf 2:

C	2.94654	1.04180	0.38210
C	1.69625	1.48973	1.13992
N	0.67060	1.58948	0.08375
C	1.08531	1.12663	-1.16439
C	2.55787	0.82226	-1.05117
C	3.06108	-0.35923	-0.21003
C	4.47762	-0.80924	-0.54535
H	3.16543	1.10624	-1.90441
O	0.39859	1.05385	-2.16540
C	2.10204	-1.43495	0.21504
O	0.94708	-1.26123	0.55669
O	2.68249	-2.65012	0.18745
H	2.46630	-4.63839	0.49388
C	1.84190	-3.74975	0.58389
C	-1.95794	0.62781	0.16186
C	-2.47994	0.38150	-1.11043
C	-3.30739	-0.72429	-1.29205
C	-3.62083	-1.58286	-0.22769
C	-3.08664	-1.30272	1.03889
C	-2.25460	-0.20482	1.24316
C	-4.49830	-2.79232	-0.44508
S	-0.93834	2.06533	0.43509
O	-1.31246	3.09654	-0.53083
O	-0.90238	2.35656	1.87244
H	1.85857	2.46859	1.59669
H	1.38180	0.78506	1.91256
H	3.88122	1.52497	0.64942
H	4.47843	-1.52141	-1.37553
H	4.94777	-1.29353	0.31586
H	5.09132	0.05081	-0.83148
H	0.97215	-3.82012	-0.07362
H	1.50370	-3.61887	1.61479
H	-2.23809	1.04132	-1.93420
H	-3.72162	-0.92015	-2.27770
H	-3.32978	-1.94919	1.87822
H	-1.85535	0.01627	2.22649
H	-5.22438	-2.62151	-1.24642
H	-3.89616	-3.66491	-0.73148
H	-5.04647	-3.05883	0.46432

Revised structure of 158{10c}, i.e. 160{10c-rev} (Benzene-d_6)

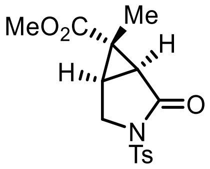

					Conf1
		Rel energy (kcal/mol):			0.00
**C-nom**	**iGau**	**Exp**	**Calc**	**diff**	**1**
C-C	20	171.40	171.29	-0.11	[171.29]
C-C	4	168.30	167.09	-1.21	[167.09]
C-C	13	144.90	146.05	1.15	[146.05]
C-C	10	136.20	136.14	-0.06	[136.14]
C-C	6	27.40	27.64	0.24	[27.64]
C-CH	12	129.60	129.63	0.03	[129.63]
C-CH	14	129.60	129.63	0.03	[129.63]
C-CH	11	128.70	128.66	-0.04	[128.66]
C-CH	15	128.70	128.66	-0.04	[128.66]
C-CH	5	34.10	34.50	0.40	[34.50]
C-CH	1	25.10	25.65	0.55	[25.65]
C-CH2	2	45.10	42.37	-2.73	[42.37]
C-CH3	23	51.90	52.90	1.00	[52.90]
C-CH3	16	21.10	20.67	-0.43	[20.67]
C-CH3	9	7.60	8.39	0.79	[8.39]

13C chem shifts: RMSD=0.92ppm (MAE=0.59) N=15 {-2.73 1.15}

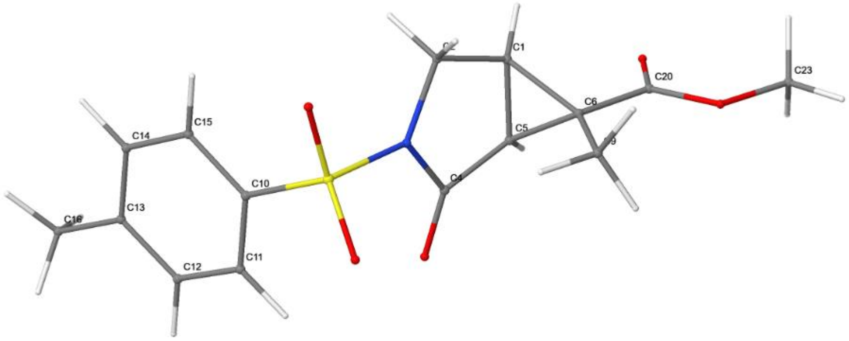

Conformer 1
Energy: -1410.81268 Hartree (Rel: 0.0 kcal/mol)
XYZ coordinates for conf 1:

C	-2.03645	-0.28246	1.22488
C	-0.88631	0.69605	1.43337
N	-0.08649	0.54595	0.20066
C	-0.53478	-0.46602	-0.66083
C	-1.80000	-1.00953	-0.06798
C	-2.98739	-0.03596	0.06385
H	-2.08042	1.75109	-0.78794
O	0.03527	-0.84133	-1.66703
C	-3.06166	1.28391	-0.68460
C	2.67564	0.45989	-0.04374
C	3.07193	-0.25342	-1.17862
C	4.18635	-1.08387	-1.09047
C	4.90846	-1.21303	0.10632
C	4.49026	-0.47560	1.22382
C	3.37797	0.36131	1.16003
C	6.09723	-2.13972	0.19107
S	1.26611	1.55027	-0.12743
O	1.30463	2.46172	1.02064
O	1.12742	2.05799	-1.49012
C	-4.27744	-0.79576	0.23683
O	-4.37261	-1.86572	0.81010

O	-5.32178	-0.15192	-0.31104
C	-6.59627	-0.81118	-0.18573
H	-0.29223	0.41451	2.30857
H	-1.21212	1.73180	1.55754
H	-2.42345	-0.81020	2.08908
H	-3.71979	1.98319	-0.15945
H	-2.01532	-2.06137	-0.21309
H	-3.46832	1.13265	-1.68855
H	2.51315	-0.16174	-2.10161
H	4.50369	-1.63915	-1.96925
H	5.04659	-0.55041	2.15449
H	3.06989	0.94514	2.02047
H	6.77769	-1.84713	0.99671
H	5.77433	-3.16999	0.39100
H	6.66094	-2.15359	-0.74752
H	-7.31165	-0.15446	-0.68012
H	-6.56841	-1.78854	-0.67338
H	-6.85554	-0.94146	0.86772

Originally assigned (incorrect) structure of 159{10h} (Benzene-d_6)

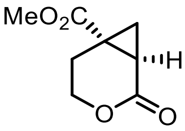

	Conf1	Conf2	Conf3
Rel energy (kcal/mol):	0.00	1.94	2.29

**C-nom**	**iGau**	**Exp**	**Calc**	**diff**	**1**	**2**	**3**
C-C	4	176.30	164.72	-11.58	[164.65	165.82	165.87]
C-C	9	170.60	170.58	-0.02	[170.59	170.40	170.56]
C-C	6	28.00	25.23	-2.77	[25.20	25.84	25.39]
C-CH	5	25.90	25.16	-0.74	[25.38	20.71	22.53]
C-CH2	2	65.40	63.21	-2.19	[62.95	67.68	67.47]
C-CH2	1	26.00	21.24	-4.76	[21.00	25.27	25.30]
C-CH2	8	19.80	16.11	-3.69	[15.59	24.34	26.26]
C-CH3	12	51.40	52.80	1.40	[52.82	52.40	52.73]

13C chem shifts: RMSD=4.82ppm (MAE=3.40) N=8 {-11.58 1.40}

Fractions: 0.944 0.036 0.020

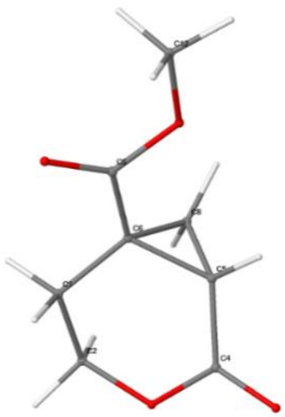

Conformer 1
Energy: -611.74669 Hartree (Rel: 0.0 kcal/mol)
XYZ coordinates for conf 1:

C	-0.20931	-1.49453	0.11704
C	-1.70529	-1.69706	0.33689
O	-2.52106	-0.73833	-0.36953
C	-2.15762	0.56616	-0.46310
C	-0.85068	0.99712	0.11951
C	0.21092	-0.06688	0.42047
O	-2.88973	1.33693	-1.04446
C	-0.45825	0.69011	1.54343
C	1.61712	0.35821	0.11873
O	2.04283	1.48541	0.28456
O	2.35865	-0.65837	-0.35860
C	3.72505	-0.33522	-0.68148
H	0.04113	-1.72951	-0.92294
H	0.33344	-2.20695	0.74897
H	-2.02113	-2.66879	-0.04796
H	-1.96682	-1.66041	1.40008
H	-0.50782	1.94648	-0.27596
H	0.13727	1.45200	2.03472
H	-1.15095	0.16197	2.18979
H	4.16385	-1.26312	-1.04761
H	4.25314	0.01689	0.20793
H	3.76074	0.43888	-1.45171

Conformer 2

Energy: -611.75034 Hartree (Rel: 1.9 kcal/mol)
XYZ coordinates for conf 2:

C	0.59210	1.57929	0.58683
C	1.69579	1.60163	-0.45890
O	2.59413	0.47149	-0.32851
C	2.07642	-0.78060	-0.30413
C	0.61977	-0.93797	0.02015
C	-0.19096	0.27182	0.49711
O	2.79875	-1.73118	-0.50705
C	0.16657	-0.84606	1.45434
C	-1.60600	0.39104	0.01539
O	-2.14174	1.43897	-0.29004
O	-2.22628	-0.80493	-0.05058
C	-3.58703	-0.77948	-0.51951
H	-0.08745	2.41806	0.41257
H	1.02796	1.70330	1.58421
H	1.27604	1.59477	-1.47187
H	2.33914	2.47757	-0.35631
H	0.11979	-1.68412	-0.58750
H	-0.61800	-1.52974	1.75869
H	0.90204	-0.61247	2.21926
H	-3.91637	-1.81829	-0.51151
H	-3.63429	-0.36826	-1.53078
H	-4.20772	-0.17356	0.14503

Conformer 3
Energy: -611.74725 Hartree (Rel: 2.3 kcal/mol)
XYZ coordinates for conf 3:

C	0.20669	1.32254	0.84843
C	1.21761	1.82693	-0.17072
O	2.39691	0.98879	-0.22788
C	2.25008	-0.34728	-0.41136
C	0.90614	-0.95484	-0.14033
C	-0.19363	-0.11572	0.52112
O	3.20373	-1.01023	-0.75303
C	0.48816	-1.23777	1.27691
C	-1.57392	-0.43227	0.02756
O	-1.95178	-1.55449	-0.25313
O	-2.35220	0.66107	-0.08423
C	-3.69021	0.42543	-0.56271
H	-0.67010	1.97371	0.83670
H	0.64313	1.37954	1.85190
H	0.77419	1.87214	-1.17278
H	1.59628	2.81829	0.08583
H	0.60040	-1.69241	-0.87505
H	-0.08035	-2.14811	1.43425
H	1.15249	-0.94907	2.08641
H	-4.16640	1.40538	-0.58775
H	-4.22378	-0.24560	0.11459
H	-3.66468	-0.01709	-1.56139

Revised structure of 159{10h}, i.e. 161{10h-rev} (Benzene-d_6)

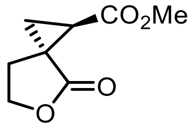

			Rel energy (kcal/mol):		**Conf1** 0.00	**Conf2** 1.63	
**C-nom**	**iGau**	**Exp**	**Calc**	**diff**	**1**	**2**	
C-C	9	176.30	175.77	-0.53	[175.78	175.55]	
C-C	4	170.60	169.95	-0.65	[170.09	167.71]	
C-C	6	28.00	28.52	0.52	[28.58	27.66]	
C-CH	5	25.90	25.40	-0.50	[25.34	26.31]	
C-CH2	2	65.40	65.54	0.14	[65.56	65.19]	
C-CH2	1	26.00	27.78	1.78	[27.74	28.46]	
C-CH2	8	19.80	22.08	2.28	[22.13	21.28]	
C-CH3	12	51.40	52.07	0.67	[52.10	51.68]	

13C chem shifts: RMSD=1.12ppm (MAE=0.89) N=8 {-0.65 2.28}

Fractions: 0.939 0.061

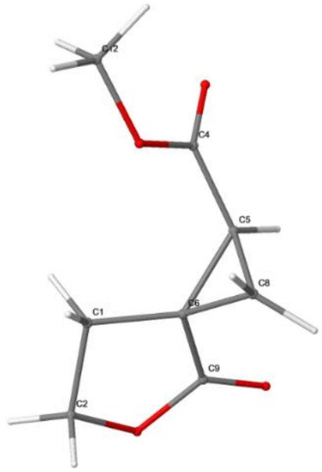

Conformer 1
Energy: -611.75456 Hartree (Rel: 0.0 kcal/mol)
XYZ coordinates for conf 1:

C	1.01728	-1.44150	-0.04338
C	2.52480	-1.47048	-0.35552
O	-2.66667	0.65875	-0.52565
C	-1.76572	-0.03805	0.19262
C	-0.51994	0.75293	0.39600
C	0.81479	0.01630	0.33500
O	-1.95904	-1.16427	0.61234
C	0.27846	0.51029	1.65205
C	1.96857	0.77036	-0.26374
O	2.11167	1.96769	-0.35143
O	2.89320	-0.11415	-0.71406
C	-3.91561	-0.01125	-0.77757
H	0.74394	-2.14173	0.74753
H	0.43399	-1.69754	-0.93377
H	3.12287	-1.75870	0.51523
H	2.78853	-2.11168	-1.19822
H	-0.51862	1.75733	-0.01400
H	0.75908	1.37230	2.10446
H	-0.10336	-0.24333	2.33444
H	-4.51357	0.69523	-1.35288
H	-3.74896	-0.92818	-1.34855
H	-4.41285	-0.25965	0.16340

Conformer 2
Energy: -611.75197 Hartree (Rel: 1.6 kcal/mol)
XYZ coordinates for conf 2:

C	-0.69384	1.41398	-0.06799
C	-2.19523	1.73674	-0.18245
O	2.17024	0.55349	0.33792
C	1.79495	-0.61230	-0.23497
C	0.43313	-1.05230	0.18276
C	-0.72569	-0.06408	0.28543
O	2.49728	-1.23534	-1.00513
C	-0.14222	-0.68530	1.52845
C	-2.07010	-0.56978	-0.15997
O	-2.45132	-1.71494	-0.22432
O	-2.85745	0.48498	-0.49001
C	3.47913	1.02974	-0.02450
H	-0.19541	2.03358	0.67950
H	-0.19358	1.57327	-1.02901
H	-2.61076	2.11221	0.75856
H	-2.43521	2.43723	-0.98403
H	0.16723	-2.02457	-0.21907
H	-0.72833	-1.44714	2.03381
H	0.45228	-0.03992	2.16706
H	3.60798	1.96783	0.51536
H	4.24335	0.30745	0.27270
H	3.54174	1.19423	-1.10303

Structure (correct) of starting 162{9} ($CDCl_3$)

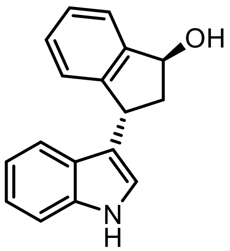

					Conf1	Conf2	Conf3	Conf4
			Rel energy (kcal/mol):		0.00	0.27	0.65	1.20

C-nom	iGau	Exp	Calc	diff	1	2	3	4
C-C	13	145.60	144.20	-1.40	[145.31	143.04	143.90	142.05]
C-C	14	144.90	145.20	0.30	[143.74	146.75	146.02	146.83]
C-C	4	136.70	136.36	-0.34	[136.45	136.79	135.63	135.46]
C-C	5	126.80	126.26	-0.54	[126.80	124.80	127.27	126.56]
C-C	9	119.20	117.85	-1.35	[119.12	115.31	118.04	119.87]
C-CH	17	128.30	127.81	-0.49	[127.31	128.44	128.25	127.40]
C-CH	16	127.10	126.37	-0.73	[126.56	126.19	126.38	125.80]
C-CH	18	125.00	124.62	-0.38	[125.09	124.22	124.26	123.95]
C-CH	15	123.90	124.45	0.55	[123.46	125.53	125.71	123.60]
C-CH	8	122.10	122.60	0.50	[122.14	124.39	121.52	120.38]
C-CH	2	121.60	121.44	-0.16	[121.51	121.27	121.54	121.46]
C-CH	1	119.40	118.80	-0.60	[118.90	118.49	118.96	119.09]
C-CH	6	119.40	118.73	-0.67	[118.48	119.66	118.00	118.09]
C-CH	3	111.30	110.33	-0.97	[110.32	110.39	110.27	110.29]
C-CH	12	75.20	75.82	0.62	[75.66	75.80	75.78	77.17]
C-CH	10	39.20	40.54	1.34	[40.06	42.12	39.56	39.13]
C-CH2	11	45.40	45.78	0.38	[46.15	44.26	47.86	45.03]

13C chem shifts: RMSD=0.76ppm (MAE=0.67) N=17 {-1.40 1.34}

Fractions: 0.476 0.300 0.160 0.063

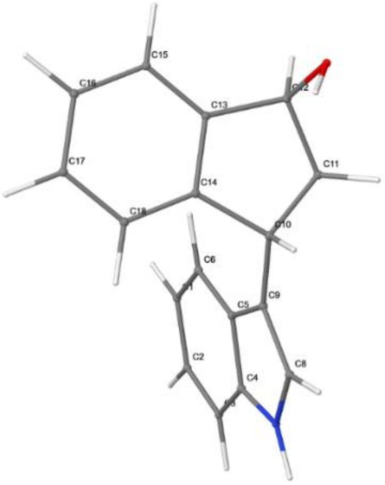

Conformer 1
Energy: -786.80847 Hartree (Rel: 0.0 kcal/mol)
XYZ coordinates for conf 1:

C	4.22840	0.99479	0.94774
C	4.92454	0.14297	0.06367
C	4.24905	-0.77462	-0.73485
C	2.85381	-0.82062	-0.62948
C	2.12898	0.02909	0.25455
C	2.84225	0.94605	1.04870
N	1.93115	-1.61336	-1.27791
C	0.66244	-1.29592	-0.83064
C	0.72822	-0.29361	0.10773

```
C         -0.41465        0.37309        0.84030
C         -0.72704        1.80887        0.30922
C         -1.84243        1.62733       -0.74844
C         -2.55586        0.37933       -0.26093
C         -1.75095       -0.32916        0.63786
C         -3.83088       -0.07449       -0.59520
C         -4.29843       -1.25820       -0.01491
C         -3.49335       -1.97356        0.88034
C         -2.21291       -1.51470        1.21024
O         -2.67799        2.76472       -0.93641
H          4.78763        1.69959        1.55731
H          6.00795        0.20403        0.00544
H          4.78473       -1.43190       -1.41477
H          2.31838        1.60843        1.73344
H          2.14407       -2.32537       -1.95994
H         -0.19902       -1.82159       -1.21708
H         -0.15553        0.41716        1.90696
H         -1.13645        2.41525        1.12878
H          0.15413        2.32343       -0.08291
H         -1.40493        1.45125       -1.73909
H         -4.45174        0.49190       -1.28468
H         -5.29307       -1.62406       -0.25616
H         -3.86898       -2.89004        1.32801
H         -1.59382       -2.07106        1.91006
H         -3.13466        2.92881       -0.09419

Conformer 2
Energy: -786.80657 Hartree (Rel:  0.3 kcal/mol)
XYZ coordinates for conf 2:
C         -2.66475        1.62764       -1.57757
C         -3.96253        1.37228       -1.08561
C         -4.18444        0.40259       -0.11360
C         -3.07481       -0.31142        0.35418
C         -1.75527       -0.07689       -0.12976
C         -1.56534        0.91682       -1.10961
N         -2.99328       -1.31108        1.30057
C         -1.67726       -1.70844        1.42656
C         -0.87703       -0.98886        0.57137
C          0.60636       -1.16920        0.40212
C          1.04953       -1.67698       -1.00551
C          2.46936       -1.11239       -1.25088
C          2.49691        0.12198       -0.36948
C          1.45688        0.08866        0.56821
C          3.42359        1.16593       -0.37076
C          3.29119        2.19001        0.57133
C          2.24790        2.15986        1.50589
C          1.32629        1.10824        1.51171
O          3.52230       -2.03398       -0.92562
H         -2.52461        2.39585       -2.33338
H         -4.80255        1.94423       -1.47047
H         -5.18210        0.20537        0.26960
H         -0.57126        1.13301       -1.49012
H         -3.76814       -1.68817        1.82487
H         -1.40658       -2.49082        2.12302
H          0.92864       -1.89885        1.16043
H          1.03278       -2.76755       -1.09073
H          0.36551       -1.27425       -1.76085
H          2.63688       -0.88500       -2.30827
H          4.23309        1.18531       -1.09655
H          3.99760        3.01589        0.57744
H          2.15177        2.96425        2.23067
H          0.51379        1.09053        2.23364
H          3.53025       -2.13938        0.03996

Conformer 3
Energy: -786.80744 Hartree (Rel:  0.6 kcal/mol)
XYZ coordinates for conf 3:
C         -4.09284        1.31093       -0.97260
C         -4.93995        0.32801       -0.41654
C         -4.42218       -0.75715        0.28303
C         -3.03055       -0.83775        0.41465
```

C	-2.15528	0.13972	-0.13955
C	-2.71161	1.22688	-0.83902
N	-2.24217	-1.77844	1.04190
C	-0.91261	-1.43191	0.89446
C	-0.80501	-0.26262	0.17775
C	0.44826	0.49832	-0.16330
C	0.86102	1.57026	0.89588
C	2.39889	1.70570	0.80369
C	2.82030	0.35458	0.25872
C	1.72694	-0.32299	-0.29709
C	4.10338	-0.19177	0.20599
C	4.28536	-1.43736	-0.40141
C	3.19409	-2.11655	-0.95769
C	1.91057	-1.56268	-0.91245
O	2.83759	2.78621	-0.03501
H	-4.53113	2.14660	-1.51167
H	-6.01604	0.42076	-0.53612
H	-5.07359	-1.51378	0.71231
H	-2.07108	1.99310	-1.26900
H	-2.57613	-2.61043	1.50444
H	-0.13733	-2.05729	1.31412
H	0.27069	1.00576	-1.12447
H	0.36326	2.53420	0.75474
H	0.59210	1.19485	1.88988
H	2.84351	1.93112	1.77801
H	4.94841	0.34234	0.63375
H	5.27614	-1.88209	-0.44183
H	3.34584	-3.08459	-1.42828
H	1.06694	-2.09468	-1.34389
H	2.62010	2.55189	-0.95234

Conformer 4
Energy: -786.80803 Hartree (Rel: 1.2 kcal/mol)
XYZ coordinates for conf 4:

C	-3.80817	1.36732	0.95927
C	-4.65134	0.76846	-0.00143
C	-4.19403	-0.25899	-0.82005
C	-2.86693	-0.67448	-0.65755
C	-1.99526	-0.08419	0.30225
C	-2.49081	0.95176	1.11663
N	-2.14964	-1.65492	-1.30734
C	-0.86957	-1.70367	-0.78834
C	-0.72291	-0.75981	0.20113
C	0.51611	-0.44080	1.00900
C	1.50426	-1.63187	1.18131
C	2.59200	-1.46321	0.09025
C	2.56823	0.02953	-0.17603
C	1.40416	0.61065	0.33779
C	3.52750	0.79962	-0.83227
C	3.30931	2.17373	-0.97237
C	2.14218	2.75796	-0.46497
C	1.18193	1.98032	0.19172
O	3.86839	-1.99024	0.44047
H	-4.19821	2.16601	1.58455
H	-5.67629	1.11490	-0.10271
H	-4.84259	-0.72317	-1.55827
H	-1.85470	1.42056	1.86354
H	-2.50495	-2.26087	-2.03136
H	-0.15929	-2.42176	-1.17426
H	0.18401	-0.08101	1.99120
H	2.00023	-1.54604	2.15665
H	1.01042	-2.60678	1.14877
H	2.32200	-2.01627	-0.81877
H	4.43352	0.33678	-1.21539
H	4.04870	2.79226	-1.47433
H	1.98254	3.82722	-0.57778
H	0.27912	2.44256	0.58292
H	4.19593	-1.47194	1.19487

164{10-rev}(*trans*) matched with exp ^{13}C shifts of the major product ($CDCl_3$)

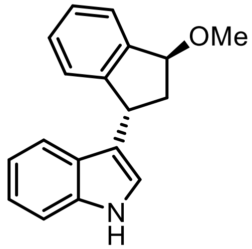

	Conf1	Conf2	Conf3	Conf4	Conf5	Conf6	Conf7	Conf8
Rel energy (kcal/mol):	0.00	0.08	0.23	0.43	0.54	1.49	1.66	3.00

C-nom	iGau	Exp	Calc	diff	1	2	3	4	5	6	7	8
C	14	147.30	146.98	-0.32	[147.48	148.06	143.28	146.90	150.35	146.43	143.77	147.07]
C	13	142.00	142.34	0.34	[142.43	141.27	144.54	143.34	139.13	141.32	146.01	143.05]
C	4	136.70	136.15	-0.55	[136.98	135.53	136.47	135.64	135.62	135.67	136.28	135.64]
C	17	128.80	127.83	-0.97	[128.17	127.97	127.08	127.97	128.08	127.18	126.72	126.90]
C	5	126.60	126.80	0.20	[125.27	127.47	126.80	127.40	128.41	126.82	126.57	126.93]
C	16	126.59	125.50	-1.09	[125.71	124.86	126.35	125.87	124.33	125.63	126.30	125.67]
C	15	125.27	125.15	-0.12	[125.64	125.22	124.23	125.79	125.02	124.34	123.19	123.47]
C	18	124.80	124.25	-0.55	[123.88	124.51	124.83	123.78	124.17	123.70	125.14	124.10]
C	8	121.93	122.63	0.70	[124.68	121.87	122.05	121.66	121.94	120.53	121.85	120.34]
C	2	121.50	121.42	-0.08	[121.24	121.46	121.48	121.49	121.58	121.52	121.44	121.53]
C	1	119.31	118.81	-0.50	[118.43	118.94	118.86	118.91	119.15	119.16	118.82	119.15]
C	6	119.30	118.69	-0.61	[119.79	118.13	118.52	118.11	118.28	118.25	118.55	118.23]
C	9	118.90	117.43	-1.47	[115.43	117.90	119.58	117.92	116.20	120.64	119.18	120.43]
C	3	111.20	110.29	-0.91	[110.47	110.15	110.30	110.20	110.27	110.30	110.21	110.27]
C	12	83.50	83.57	0.07	[84.25	82.76	83.54	84.09	82.76	84.95	83.69	85.43]
C	34	56.10	55.54	-0.56	[56.50	53.81	56.23	56.33	53.98	56.53	59.23	59.61]
C	11	41.30	43.19	1.89	[38.93	49.02	42.36	42.35	43.74	41.65	43.51	42.52]
C	10	40.00	41.06	1.06	[43.15	40.29	40.55	40.48	39.56	39.86	39.56	38.93]

13C chem shifts: RMSD=0.82ppm (MAE=0.67) N=18 {-1.47 1.89}

	1	2	3	4	5	6	7	8
Fractions:	0.278	0.245	0.189	0.136	0.111	0.022	0.017	0.002

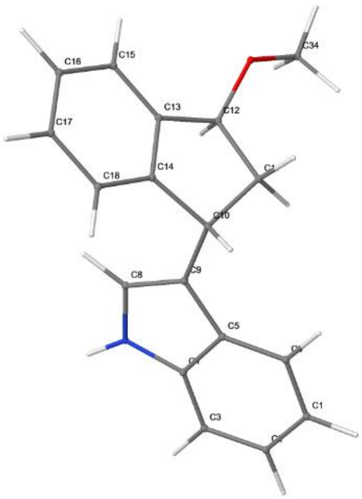

Conformer 1
Energy: -826.11332 Hartree (Rel: 0.0 kcal/mol)
XYZ coordinates for conf 1:

C	-3.02427	1.07574	-1.88440
C	-4.29920	0.71517	-1.39852
C	-4.43786	-0.09313	-0.27514
C	-3.26740	-0.53765	0.35146
C	-1.96840	-0.19143	-0.12139
C	-1.86442	0.63360	-1.25805

N	-3.09965	-1.33268	1.46564
C	-1.74996	-1.49429	1.70715
C	-1.01154	-0.82017	0.76385
C	0.48938	-0.79473	0.68366
C	1.09221	-1.48256	-0.57919
C	2.45240	-0.78887	-0.83117
C	2.24343	0.58351	-0.22700
C	1.13773	0.58510	0.63064
C	3.01482	1.73153	-0.40280
C	2.65918	2.89645	0.28406
C	1.55039	2.90085	1.13959
C	0.78404	1.74474	1.32007
O	3.53920	-1.41086	-0.13327
H	-2.95069	1.71318	-2.76153
H	-5.18794	1.07721	-1.90840
H	-5.41850	-0.36889	0.10385
H	-0.89039	0.92987	-1.63648
H	-3.84197	-1.72701	2.02322
H	-1.41155	-2.09095	2.54375
H	0.87450	-1.31185	1.57459
H	1.18836	-2.56586	-0.45848
H	0.43352	-1.30505	-1.43687
H	2.70204	-0.74885	-1.90281
H	3.87635	1.72387	-1.06620
H	3.24285	3.80361	0.15170
H	1.28018	3.81333	1.66501
H	-0.07974	1.75246	1.97991
C	4.03550	-2.56963	-0.77706
H	4.88318	-2.92540	-0.18493
H	3.28603	-3.37301	-0.83548
H	4.38148	-2.34633	-1.79883

Conformer 2
Energy: -826.11104 Hartree (Rel: 0.1 kcal/mol)
XYZ coordinates for conf 2:

C	4.15472	-1.59747	-0.95641
C	5.11047	-0.68575	-0.45795
C	4.72238	0.47073	0.21053
C	3.34975	0.69589	0.37045
C	2.36660	-0.20758	-0.12535
C	2.79284	-1.36972	-0.79515
N	2.67642	1.73385	0.97808
C	1.31545	1.51774	0.87518
C	1.07195	0.33991	0.20686
C	-0.25933	-0.30292	-0.07289
C	-0.78820	-1.22531	1.06978
C	-2.31302	-1.26862	0.88601
C	-2.60806	0.09812	0.28814
C	-1.44309	0.63505	-0.28091
C	-3.81488	0.79497	0.22908
C	-3.85560	2.03200	-0.42533
C	-2.69912	2.55738	-1.01019
C	-1.48520	1.86307	-0.93895
O	-2.59488	-2.33633	-0.02984
H	4.49275	-2.49181	-1.47308
H	6.16836	-0.89082	-0.59843
H	5.45698	1.17335	0.59479
H	2.06767	-2.08164	-1.18168
H	3.10443	2.54294	1.40231
H	0.61823	2.23337	1.28776
H	-0.15334	-0.91706	-0.97931
H	-0.35221	-2.22819	1.05371
H	-0.55295	-0.76206	2.03444
H	-2.85696	-1.45423	1.82532
H	-4.71158	0.39466	0.69604
H	-4.78786	2.58840	-0.47408
H	-2.73971	3.51922	-1.51503
H	-0.58469	2.28476	-1.37729
C	-3.97265	-2.57755	-0.23891
H	-4.04734	-3.46902	-0.86788
H	-4.49808	-2.76647	0.71127

H	-4.46959	-1.74125	-0.75072

Conformer 3
Energy: -826.11301 Hartree (Rel: 0.2 kcal/mol)
XYZ coordinates for conf 3:

C	-4.29594	0.97165	-1.08573
C	-5.03878	0.34144	-0.06457
C	-4.42407	-0.49302	0.86376
C	-3.04196	-0.68332	0.74791
C	-2.27061	-0.05713	-0.27283
C	-2.92287	0.78064	-1.19644
N	-2.17445	-1.44191	1.50433
C	-0.89581	-1.31773	0.99372
C	-0.89987	-0.47786	-0.09442
C	0.27548	-0.04477	-0.94221
C	0.71829	1.42999	-0.67064
C	1.86197	1.33349	0.37115
C	2.44760	-0.03431	0.09587
C	1.55244	-0.81583	-0.64137
C	3.68835	-0.53628	0.48466
C	4.02917	-1.84337	0.12288
C	3.13365	-2.63191	-0.61040
C	1.88802	-2.12354	-0.99445
O	2.86462	2.33285	0.24908
H	-4.80824	1.61623	-1.79505
H	-6.11053	0.50983	-0.00208
H	-4.99583	-0.97956	1.64946
H	-2.36263	1.27206	-1.98805
H	-2.43021	-2.01576	2.29358
H	-0.07307	-1.85053	1.44863
H	-0.00773	-0.15729	-1.99774
H	1.14873	1.85677	-1.58485
H	-0.11369	2.06324	-0.35170
H	1.45078	1.37182	1.39545
H	4.38135	0.08540	1.04466
H	4.99641	-2.24909	0.40780
H	3.41141	-3.64517	-0.88905
H	1.19738	-2.73730	-1.56778
C	2.45242	3.60688	0.71144
H	3.32079	4.26757	0.64316
H	2.11490	3.56546	1.75908
H	1.63954	4.02682	0.10152

Conformer 4
Energy: -826.11356 Hartree (Rel: 0.4 kcal/mol)
XYZ coordinates for conf 4:

C	-4.10850	1.47190	-1.02714
C	-5.05511	0.60669	-0.43696
C	-4.65521	-0.50156	0.30264
C	-3.28000	-0.72593	0.44006
C	-2.30598	0.13133	-0.14741
C	-2.74399	1.24513	-0.88791
N	-2.59575	-1.72223	1.10272
C	-1.23681	-1.52498	0.94615
C	-1.00568	-0.40068	0.18776
C	0.32050	0.21022	-0.17638
C	0.85485	1.25255	0.85534
C	2.39389	1.24656	0.70281
C	2.67087	-0.15316	0.19724
C	1.50314	-0.74252	-0.30161
C	3.89152	-0.82461	0.14809
C	3.93531	-2.10996	-0.40053
C	2.76954	-2.70217	-0.90095
C	1.54802	-2.02141	-0.85831
O	2.86709	2.16811	-0.28748
H	-4.45570	2.32891	-1.59816
H	-6.11530	0.80968	-0.56225
H	-5.38309	-1.16820	0.75744
H	-2.02569	1.92060	-1.34617
H	-3.01613	-2.49644	1.59426
H	-0.53211	-2.21408	1.38987

H	0.19944	0.71957	-1.14423
H	0.42075	2.24738	0.71705
H	0.59318	0.91353	1.86438
H	2.90203	1.46387	1.65517
H	4.79535	-0.35831	0.53270
H	4.87632	-2.65234	-0.43809
H	2.81341	-3.70177	-1.32589
H	0.64591	-2.48595	-1.24716
C	2.93889	3.50519	0.17190
H	3.35319	4.10137	-0.64575
H	1.95240	3.91252	0.43890
H	3.59801	3.59494	1.05001

Conformer 5
Energy: -826.11131 Hartree (Rel: 0.5 kcal/mol)
XYZ coordinates for conf 5:

C	-3.79765	0.26074	2.00878
C	-4.86197	0.29891	1.08196
C	-4.64597	0.06838	-0.27265
C	-3.33450	-0.20234	-0.68168
C	-2.24416	-0.24698	0.23385
C	-2.49706	-0.00787	1.59806
N	-2.82186	-0.46718	-1.93306
C	-1.46043	-0.68005	-1.83417
C	-1.05485	-0.55719	-0.52521
C	0.34022	-0.67792	0.02882
C	1.25757	-1.72323	-0.66434
C	2.69432	-1.25863	-0.37322
C	2.53915	0.25256	-0.30531
C	1.20035	0.58359	-0.04999
C	3.50340	1.25086	-0.44457
C	3.11955	2.59040	-0.30780
C	1.78643	2.91789	-0.03962
C	0.81664	1.91567	0.08634
O	3.07173	-1.82858	0.88793
H	-4.00185	0.44600	3.05998
H	-5.86810	0.51249	1.43261
H	-5.46436	0.09770	-0.98715
H	-1.68646	-0.03119	2.32225
H	-3.35615	-0.52472	-2.78691
H	-0.88640	-0.90460	-2.72231
H	0.25500	-0.95709	1.08912
H	1.08528	-2.74549	-0.31593
H	1.09502	-1.69853	-1.74755
H	3.41653	-1.58323	-1.13854
H	4.53647	0.99929	-0.67274
H	3.85888	3.37924	-0.41847
H	1.49753	3.96127	0.05838
H	-0.22237	2.17524	0.27192
C	4.40896	-1.56735	1.26719
H	4.59438	-2.12982	2.18646
H	5.12036	-1.90195	0.49476
H	4.58544	-0.50029	1.46341

Conformer 6
Energy: -826.10890 Hartree (Rel: 1.5 kcal/mol)
XYZ coordinates for conf 6:

C	-4.20636	0.93577	1.06139
C	-4.97456	0.28145	0.07436
C	-4.39307	-0.62239	-0.80866
C	-3.01830	-0.85646	-0.68382
C	-2.22065	-0.20673	0.30184
C	-2.84141	0.70054	1.18136
N	-2.18272	-1.68670	-1.39843
C	-0.89946	-1.58547	-0.89485
C	-0.86842	-0.69012	0.14856
C	0.32984	-0.24664	0.95944
C	1.47196	-1.30177	1.06256
C	2.51691	-0.92173	-0.01965
C	2.28386	0.56037	-0.20825
C	1.05461	0.94663	0.33435

C   3.11559   1.48599   -0.83625
C   2.70167   2.81895   -0.91636
C   1.46859   3.20915   -0.37933
C   0.63699   2.27530   0.24762
O   3.86717   -1.16403   0.35228
H   -4.69283   1.63442   1.73682
H   -6.03939   0.48621   0.00344
H   -4.98382   -1.12871   -1.56737
H   -2.26388   1.20988   1.94895
H   -2.46267   -2.29364   -2.15405
H   -0.10193   -2.17134   -1.33062
H   -0.03024   0.01397   1.96284
H   1.96993   -1.19992   2.03383
H   1.10123   -2.32675   0.97753
H   2.31622   -1.46254   -0.96251
H   4.07468   1.17537   -1.24151
H   3.34053   3.55704   -1.39422
H   1.15663   4.24830   -0.44584
H   -0.31886   2.58570   0.66193
C   4.22161   -2.53527   0.33405
H   5.29015   -2.59200   0.55785
H   4.03837   -2.98575   -0.65442
H   3.67122   -3.11653   1.08805

Conformer 7
Energy: -826.11369 Hartree (Rel: 1.7 kcal/mol)
XYZ coordinates for conf 7:
C   -4.36377   0.95611   -1.10769
C   -5.08392   0.42302   -0.01727
C   -4.45132   -0.33619   0.96219
C   -3.07461   -0.55071   0.82635
C   -2.32596   -0.02185   -0.26393
C   -2.99590   0.74159   -1.23794
N   -2.19358   -1.25179   1.62160
C   -0.92829   -1.18581   1.06843
C   -0.95450   -0.44111   -0.08665
C   0.20104   -0.09479   -0.99918
C   0.66080   1.39020   -0.86814
C   1.79848   1.38217   0.17284
C   2.39318   -0.00851   -0.00080
C   1.48289   -0.84291   -0.66108
C   3.64342   -0.49019   0.38804
C   3.97411   -1.82071   0.11096
C   3.06114   -2.65790   -0.54125
C   1.80797   -2.17284   -0.92970
O   2.70069   2.45464   -0.07956
H   -4.88959   1.54392   -1.85538
H   -6.15207   0.60784   0.05892
H   -5.00537   -0.74809   1.80144
H   -2.45311   1.15839   -2.08271
H   -2.43347   -1.75572   2.46190
H   -0.09831   -1.68799   1.54461
H   -0.10795   -0.30039   -2.03318
H   1.09542   1.72576   -1.81730
H   -0.15249   2.07114   -0.60353
H   1.38725   1.49139   1.18891
H   4.36070   0.15664   0.88564
H   4.94848   -2.20620   0.39973
H   3.33113   -3.68900   -0.75411
H   1.10279   -2.82158   -1.44396
C   3.25355   3.04853   1.08198
H   3.87358   3.88432   0.74620
H   3.88346   2.35219   1.65401
H   2.46914   3.43414   1.75110

Conformer 8
Energy: -826.11282 Hartree (Rel: 3.0 kcal/mol)
XYZ coordinates for conf 8:
C   -4.24492   1.07743   0.87862
C   -4.99713   0.35277   -0.07087
C   -4.41059   -0.64138   -0.84701

C	-3.04707	-0.89396	-0.65380
C	-2.26536	-0.17439	0.29546
C	-2.89109	0.82341	1.06681
N	-2.21057	-1.80593	-1.25930
C	-0.94216	-1.68786	-0.72256
C	-0.92177	-0.70050	0.23455
C	0.25784	-0.21491	1.04877
C	1.36974	-1.27575	1.28224
C	2.44917	-1.03109	0.20584
C	2.27231	0.44550	-0.11663
C	1.03636	0.90450	0.35453
C	3.14292	1.31071	-0.78090
C	2.76527	2.64381	-0.96899
C	1.52685	3.10087	-0.50347
C	0.65470	2.23239	0.16013
O	3.72875	-1.38389	0.72561
H	-4.73506	1.84571	1.47064
H	-6.05358	0.57390	-0.19685
H	-4.98939	-1.20237	-1.57595
H	-2.32624	1.38730	1.80519
H	-2.48062	-2.47145	-1.96771
H	-0.14743	-2.33162	-1.07329
H	-0.13161	0.13988	2.01152
H	1.84819	-1.09194	2.25069
H	0.99193	-2.30173	1.28434
H	2.25492	-1.64032	-0.69205
H	4.10828	0.96450	-1.13954
H	3.43817	3.32952	-1.47723
H	1.24313	4.13921	-0.65454
H	-0.30563	2.59210	0.52089
C	4.62643	-1.93178	-0.22333
H	5.52797	-2.22087	0.32383
H	4.90747	-1.21297	-1.00612
H	4.20444	-2.82475	-0.70994

164{10-rev}(*trans*) matched with exp ^{13}C shifts of the minor product ($CDCl_3$)

					Conf1	Conf2	Conf3	Conf4	Conf5	Conf6	Conf7	Conf8
			Rel energy (kcal/mol):		0.00	0.08	0.23	0.43	0.54	1.49	1.66	3.00
**C-nom**	**iGau**	**Exp**	**Calc**	**diff**	**1**	**2**	**3**	**4**	**5**	**6**	**7**	**8**
C	14	145.60	146.98	1.38	[147.48	148.06	143.28	146.90	150.35	146.43	143.77	147.07]
C	13	142.80	142.34	-0.46	[142.43	141.27	144.54	143.34	139.13	141.32	146.01	143.05]
C	4	136.50	136.15	-0.35	[136.98	135.53	136.47	135.64	135.62	135.67	136.28	135.64]
C	17	128.20	127.83	-0.37	[128.17	127.97	127.08	127.97	128.08	127.18	126.72	126.90]
C	5	126.80	126.80	-0.00	[125.27	127.47	126.80	127.40	128.41	126.82	126.57	126.93]
C	16	126.54	125.50	-1.04	[125.71	124.86	126.35	125.87	124.33	125.63	126.30	125.67]
C	15	125.29	125.15	-0.14	[125.64	125.22	124.23	125.79	125.02	124.34	123.19	123.47]
C	18	124.30	124.25	-0.05	[123.88	124.51	124.83	123.78	124.17	123.70	125.14	124.10]
C	8	121.90	122.63	0.73	[124.68	121.87	122.05	121.66	121.94	120.53	121.85	120.34]
C	2	121.70	121.42	-0.28	[121.24	121.46	121.48	121.49	121.58	121.52	121.44	121.53]
C	1	119.18	118.81	-0.37	[118.43	118.94	118.86	118.91	119.15	119.16	118.82	119.15]
C	6	119.13	118.69	-0.44	[119.79	118.13	118.52	118.11	118.28	118.25	118.55	118.23]
C	9	118.70	117.43	-1.27	[115.43	117.90	119.58	117.92	116.20	120.64	119.18	120.43]
C	3	111.10	110.29	-0.81	[110.47	110.15	110.30	110.20	110.27	110.30	110.21	110.27]
C	12	83.30	83.57	0.27	[84.25	82.76	83.54	84.09	82.76	84.95	83.69	85.43]
C	34	56.50	55.54	-0.96	[56.50	53.81	56.23	56.33	53.98	56.53	59.23	59.61]
C	11	41.20	43.19	1.99	[38.93	49.02	42.36	42.35	43.74	41.65	43.51	42.52]
C	10	39.20	41.06	1.86	[43.15	40.29	40.55	40.48	39.56	39.86	39.56	38.93]
					**13C chem shifts: RMSD=0.92ppm (MAE=0.71) N=18 {-1.27 1.99}**							
			Fractions:		0.278	0.245	0.189	0.136	0.111	0.022	0.017	0.002

```
Conformer 1
Energy: -826.11332 Hartree (Rel:  0.0 kcal/mol)
XYZ coordinates for conf 1:
C        -3.02427       1.07574       -1.88440
C        -4.29920       0.71517       -1.39852
C        -4.43786      -0.09313       -0.27514
C        -3.26740      -0.53765        0.35146
C        -1.96840      -0.19143       -0.12139
C        -1.86442       0.63360       -1.25805
N        -3.09965      -1.33268        1.46564
C        -1.74996      -1.49429        1.70715
C        -1.01154      -0.82017        0.76385
C         0.48938      -0.79473        0.68366
C         1.09221      -1.48256       -0.57919
C         2.45240      -0.78887       -0.83117
C         2.24343       0.58351       -0.22700
C         1.13773       0.58510        0.63064
C         3.01482       1.73153       -0.40280
C         2.65918       2.89645        0.28406
C         1.55039       2.90085        1.13959
C         0.78404       1.74474        1.32007
O         3.53920      -1.41086       -0.13327
H        -2.95069       1.71318       -2.76153
H        -5.18794       1.07721       -1.90840
H        -5.41850      -0.36889        0.10385
H        -0.89039       0.92987       -1.63648
H        -3.84197      -1.72701        2.02322
H        -1.41155      -2.09095        2.54375
H         0.87450      -1.31185        1.57459
H         1.18836      -2.56586       -0.45848
H         0.43352      -1.30505       -1.43687
H         2.70204      -0.74885       -1.90281
H         3.87635       1.72387       -1.06620
H         3.24285       3.80361        0.15170
H         1.28018       3.81333        1.66501
H        -0.07974       1.75246        1.97991
C         4.03550      -2.56963       -0.77706
H         4.88318      -2.92540       -0.18493
H         3.28603      -3.37301       -0.83548
H         4.38148      -2.34633       -1.79883

Conformer 2
Energy: -826.11104 Hartree (Rel:  0.1 kcal/mol)
XYZ coordinates for conf 2:
```

C	4.15472	-1.59747	-0.95641
C	5.11047	-0.68575	-0.45795
C	4.72238	0.47073	0.21053
C	3.34975	0.69589	0.37045
C	2.36660	-0.20758	-0.12535
C	2.79284	-1.36972	-0.79515
N	2.67642	1.73385	0.97808
C	1.31545	1.51774	0.87518
C	1.07195	0.33991	0.20686
C	-0.25933	-0.30292	-0.07289
C	-0.78820	-1.22531	1.06978
C	-2.31302	-1.26862	0.88601
C	-2.60806	0.09812	0.28814
C	-1.44309	0.63505	-0.28091
C	-3.81488	0.79497	0.22908
C	-3.85560	2.03200	-0.42533
C	-2.69912	2.55738	-1.01019
C	-1.48520	1.86307	-0.93895
O	-2.59488	-2.33633	-0.02984
H	4.49275	-2.49181	-1.47308
H	6.16836	-0.89082	-0.59843
H	5.45698	1.17335	0.59479
H	2.06767	-2.08164	-1.18168
H	3.10443	2.54294	1.40231
H	0.61823	2.23337	1.28776
H	-0.15334	-0.91706	-0.97931
H	-0.35221	-2.22819	1.05371
H	-0.55295	-0.76206	2.03444
H	-2.85696	-1.45423	1.82532
H	-4.71158	0.39466	0.69604
H	-4.78786	2.58840	-0.47408
H	-2.73971	3.51922	-1.51503
H	-0.58469	2.28476	-1.37729
C	-3.97265	-2.57755	-0.23891
H	-4.04734	-3.46902	-0.86788
H	-4.49808	-2.76647	0.71127
H	-4.46959	-1.74125	-0.75072

Conformer 3
Energy: -826.11301 Hartree (Rel: 0.2 kcal/mol)
XYZ coordinates for conf 3:

C	-4.29594	0.97165	-1.08573
C	-5.03878	0.34144	-0.06457
C	-4.42407	-0.49302	0.86376
C	-3.04196	-0.68332	0.74791
C	-2.27061	-0.05713	-0.27283
C	-2.92287	0.78064	-1.19644
N	-2.17445	-1.44191	1.50433
C	-0.89581	-1.31773	0.99372
C	-0.89987	-0.47786	-0.09442
C	0.27548	-0.04477	-0.94221
C	0.71829	1.42999	-0.67064
C	1.86197	1.33349	0.37115
C	2.44760	-0.03431	0.09587
C	1.55244	-0.81583	-0.64137
C	3.68835	-0.53628	0.48466
C	4.02917	-1.84337	0.12288
C	3.13365	-2.63191	-0.61040
C	1.88802	-2.12354	-0.99445
O	2.86462	2.33285	0.24908
H	-4.80824	1.61623	-1.79505
H	-6.11053	0.50983	-0.00208
H	-4.99583	-0.97956	1.64946
H	-2.36263	1.27206	-1.98805
H	-2.43021	-2.01576	2.29358
H	-0.07307	-1.85053	1.44863
H	-0.00773	-0.15729	-1.99774
H	1.14873	1.85677	-1.58485
H	-0.11369	2.06324	-0.35170
H	1.45078	1.37182	1.39545
H	4.38135	0.08540	1.04466

H	4.99641	-2.24909	0.40780
H	3.41141	-3.64517	-0.88905
H	1.19738	-2.73730	-1.56778
C	2.45242	3.60688	0.71144
H	3.32079	4.26757	0.64316
H	2.11490	3.56546	1.75908
H	1.63954	4.02682	0.10152

Conformer 4
Energy: -826.11356 Hartree (Rel: 0.4 kcal/mol)
XYZ coordinates for conf 4:

C	-4.10850	1.47190	-1.02714
C	-5.05511	0.60669	-0.43696
C	-4.65521	-0.50156	0.30264
C	-3.28000	-0.72593	0.44006
C	-2.30598	0.13133	-0.14741
C	-2.74399	1.24513	-0.88791
N	-2.59575	-1.72223	1.10272
C	-1.23681	-1.52498	0.94615
C	-1.00568	-0.40068	0.18776
C	0.32050	0.21022	-0.17638
C	0.85485	1.25255	0.85534
C	2.39389	1.24656	0.70281
C	2.67087	-0.15316	0.19724
C	1.50314	-0.74252	-0.30161
C	3.89152	-0.82461	0.14809
C	3.93531	-2.10996	-0.40053
C	2.76954	-2.70217	-0.90095
C	1.54802	-2.02141	-0.85831
O	2.86709	2.16811	-0.28748
H	-4.45570	2.32891	-1.59816
H	-6.11530	0.80968	-0.56225
H	-5.38309	-1.16820	0.75744
H	-2.02569	1.92060	-1.34617
H	-3.01613	-2.49644	1.59426
H	-0.53211	-2.21408	1.38987
H	0.19944	0.71957	-1.14423
H	0.42075	2.24738	0.71705
H	0.59318	0.91353	1.86438
H	2.90203	1.46387	1.65517
H	4.79535	-0.35831	0.53270
H	4.87632	-2.65234	-0.43809
H	2.81341	-3.70177	-1.32589
H	0.64591	-2.48595	-1.24716
C	2.93889	3.50519	0.17190
H	3.35319	4.10137	-0.64575
H	1.95240	3.91252	0.43890
H	3.59801	3.59494	1.05001

Conformer 5
Energy: -826.11131 Hartree (Rel: 0.5 kcal/mol)
XYZ coordinates for conf 5:

C	-3.79765	0.26074	2.00878
C	-4.86197	0.29891	1.08196
C	-4.64597	0.06838	-0.27265
C	-3.33450	-0.20234	-0.68168
C	-2.24416	-0.24698	0.23385
C	-2.49706	-0.00787	1.59806
N	-2.82186	-0.46718	-1.93306
C	-1.46043	-0.68005	-1.83417
C	-1.05485	-0.55719	-0.52521
C	0.34022	-0.67792	0.02882
C	1.25757	-1.72323	-0.66434
C	2.69432	-1.25863	-0.37322
C	2.53915	0.25256	-0.30531
C	1.20035	0.58359	-0.04999
C	3.50340	1.25086	-0.44457
C	3.11955	2.59040	-0.30780
C	1.78643	2.91789	-0.03962
C	0.81664	1.91567	0.08634
O	3.07173	-1.82858	0.88793

H	-4.00185	0.44600	3.05998
H	-5.86810	0.51249	1.43261
H	-5.46436	0.09770	-0.98715
H	-1.68646	-0.03119	2.32225
H	-3.35615	-0.52472	-2.78691
H	-0.88640	-0.90460	-2.72231
H	0.25500	-0.95709	1.08912
H	1.08528	-2.74549	-0.31593
H	1.09502	-1.69853	-1.74755
H	3.41653	-1.58323	-1.13854
H	4.53647	0.99929	-0.67274
H	3.85888	3.37924	-0.41847
H	1.49753	3.96127	0.05838
H	-0.22237	2.17524	0.27192
C	4.40896	-1.56735	1.26719
H	4.59438	-2.12982	2.18646
H	5.12036	-1.90195	0.49476
H	4.58544	-0.50029	1.46341

Conformer 6
Energy: -826.10890 Hartree (Rel: 1.5 kcal/mol)
XYZ coordinates for conf 6:

C	-4.20636	0.93577	1.06139
C	-4.97456	0.28145	0.07436
C	-4.39307	-0.62239	-0.80866
C	-3.01830	-0.85646	-0.68382
C	-2.22065	-0.20673	0.30184
C	-2.84141	0.70054	1.18136
N	-2.18272	-1.68670	-1.39843
C	-0.89946	-1.58547	-0.89485
C	-0.86842	-0.69012	0.14856
C	0.32984	-0.24664	0.95944
C	1.47196	-1.30177	1.06256
C	2.51691	-0.92173	-0.01965
C	2.28386	0.56037	-0.20825
C	1.05461	0.94663	0.33435
C	3.11559	1.48599	-0.83625
C	2.70167	2.81895	-0.91636
C	1.46859	3.20915	-0.37933
C	0.63699	2.27530	0.24762
O	3.86717	-1.16403	0.35228
H	-4.69283	1.63442	1.73682
H	-6.03939	0.48621	0.00344
H	-4.98382	-1.12871	-1.56737
H	-2.26388	1.20988	1.94895
H	-2.46267	-2.29364	-2.15405
H	-0.10193	-2.17134	-1.33062
H	-0.03024	0.01397	1.96284
H	1.96993	-1.19992	2.03383
H	1.10123	-2.32675	0.97753
H	2.31622	-1.46254	-0.96251
H	4.07468	1.17537	-1.24151
H	3.34053	3.55704	-1.39422
H	1.15663	4.24830	-0.44584
H	-0.31886	2.58570	0.66193
C	4.22161	-2.53527	0.33405
H	5.29015	-2.59200	0.55785
H	4.03837	-2.98575	-0.65442
H	3.67122	-3.11653	1.08805

Conformer 7
Energy: -826.11369 Hartree (Rel: 1.7 kcal/mol)
XYZ coordinates for conf 7:

C	-4.36377	0.95611	-1.10769
C	-5.08392	0.42302	-0.01727
C	-4.45132	-0.33619	0.96219
C	-3.07461	-0.55071	0.82635
C	-2.32596	-0.02185	-0.26393
C	-2.99590	0.74159	-1.23794
N	-2.19358	-1.25179	1.62160
C	-0.92829	-1.18581	1.06843

C	-0.95450	-0.44111	-0.08665
C	0.20104	-0.09479	-0.99918
C	0.66080	1.39020	-0.86814
C	1.79848	1.38217	0.17284
C	2.39318	-0.00851	-0.00080
C	1.48289	-0.84291	-0.66108
C	3.64342	-0.49019	0.38804
C	3.97411	-1.82071	0.11096
C	3.06114	-2.65790	-0.54125
C	1.80797	-2.17284	-0.92970
O	2.70069	2.45464	-0.07956
H	-4.88959	1.54392	-1.85538
H	-6.15207	0.60784	0.05892
H	-5.00537	-0.74809	1.80144
H	-2.45311	1.15839	-2.08271
H	-2.43347	-1.75572	2.46190
H	-0.09831	-1.68799	1.54461
H	-0.10795	-0.30039	-2.03318
H	1.09542	1.72576	-1.81730
H	-0.15249	2.07114	-0.60353
H	1.38725	1.49139	1.18891
H	4.36070	0.15664	0.88564
H	4.94848	-2.20620	0.39973
H	3.33113	-3.68900	-0.75411
H	1.10279	-2.82158	-1.44396
C	3.25355	3.04853	1.08198
H	3.87358	3.88432	0.74620
H	3.88346	2.35219	1.65401
H	2.46914	3.43414	1.75110

Conformer 8
Energy: -826.11282 Hartree (Rel: 3.0 kcal/mol)
XYZ coordinates for conf 8:

C	-4.24492	1.07743	0.87862
C	-4.99713	0.35277	-0.07087
C	-4.41059	-0.64138	-0.84701
C	-3.04707	-0.89396	-0.65380
C	-2.26536	-0.17439	0.29546
C	-2.89109	0.82341	1.06681
N	-2.21057	-1.80593	-1.25930
C	-0.94216	-1.68786	-0.72256
C	-0.92177	-0.70050	0.23455
C	0.25784	-0.21491	1.04877
C	1.36974	-1.27575	1.28224
C	2.44917	-1.03109	0.20584
C	2.27231	0.44550	-0.11663
C	1.03636	0.90450	0.35453
C	3.14292	1.31071	-0.78090
C	2.76527	2.64381	-0.96899
C	1.52685	3.10087	-0.50347
C	0.65470	2.23239	0.16013
O	3.72875	-1.38389	0.72561
H	-4.73506	1.84571	1.47064
H	-6.05358	0.57390	-0.19685
H	-4.98939	-1.20237	-1.57595
H	-2.32624	1.38730	1.80519
H	-2.48062	-2.47145	-1.96771
H	-0.14743	-2.33162	-1.07329
H	-0.13161	0.13988	2.01152
H	1.84819	-1.09194	2.25069
H	0.99193	-2.30173	1.28434
H	2.25492	-1.64032	-0.69205
H	4.10828	0.96450	-1.13954
H	3.43817	3.32952	-1.47723
H	1.24313	4.13921	-0.65454
H	-0.30563	2.59210	0.52089
C	4.62643	-1.93178	-0.22333
H	5.52797	-2.22087	0.32383
H	4.90747	-1.21297	-1.00612
H	4.20444	-2.82475	-0.70994

164{10-rev}(*cis*) matched with exp ^{13}C shifts of the major product ($CDCl_3$)

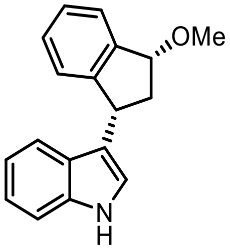

	Conf1	Conf2	Conf3	Conf4	Conf5	Conf6
Rel energy (kcal/mol):	0.00	0.56	0.61	1.04	1.10	1.50

C-nom	iGau	Exp	Calc	diff	1	2	3	4	5	6
C	14	147.30	146.70	-0.60	[143.92	150.64	149.94	146.87	148.23	144.58]
C	13	142.00	142.25	0.25	[143.88	138.51	140.50	143.65	141.89	145.34]
C	4	136.70	135.29	-1.41	[135.43	135.15	134.88	135.51	135.44	135.37]
C	17	128.80	127.65	-1.15	[127.01	128.34	128.35	128.16	128.32	126.69]
C	5	126.60	127.06	0.46	[127.21	127.18	126.82	126.48	126.95	127.26]
C	16	126.59	125.55	-1.04	[125.92	124.58	125.38	126.04	125.20	125.89]
C	15	125.27	124.70	-0.57	[124.02	125.36	125.77	125.50	124.92	123.06]
C	18	124.80	124.15	-0.65	[123.76	124.60	123.84	124.81	125.60	124.08]
C	8	121.93	123.76	1.83	[122.64	125.25	125.36	123.62	124.29	122.66]
C	2	121.50	121.16	-0.34	[121.43	120.84	120.82	121.09	121.01	121.39]
C	1	119.31	118.89	-0.42	[118.96	118.88	118.87	118.69	118.73	118.93]
C	6	119.30	117.67	-1.63	[117.75	117.47	117.45	117.92	117.87	117.71]
C	9	118.90	119.28	0.38	[118.21	119.39	120.67	121.33	120.94	118.12]
C	3	111.20	110.21	-0.99	[110.28	110.14	110.16	110.14	110.15	110.25]
C	12	83.50	83.87	0.37	[82.62	84.51	85.60	85.43	84.97	83.02]
C	34	56.10	55.85	-0.25	[56.14	54.36	56.53	55.89	54.27	59.50]
C	11	41.30	44.10	2.80	[47.15	43.17	38.95	37.76	43.57	48.41]
C	10	40.00	39.87	-0.13	[38.83	40.86	40.78	41.37	41.29	38.00]

13C chem shifts: RMSD=1.09ppm (MAE=0.85) N=18 {-1.63 2.80}

Fractions: 0.464 0.179 0.166 0.081 0.073 0.037

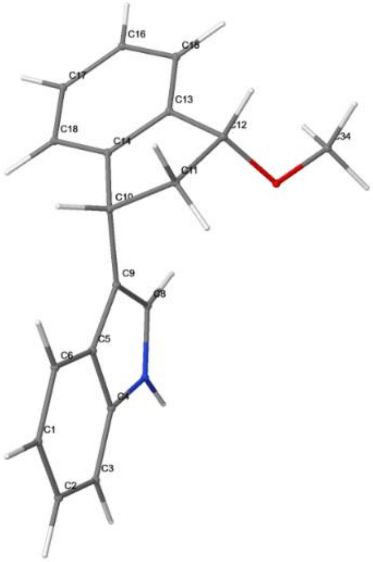

Conformer 1
Energy: -826.11232 Hartree (Rel: 0.0 kcal/mol)
XYZ coordinates for conf 1:

C	-4.26963	1.04925	-1.36707
C	-5.13965	0.46825	-0.41914
C	-4.64912	-0.29218	0.63731
C	-3.26172	-0.45895	0.72514

```
C         -2.36361        0.11540       -0.21976
C         -2.89269        0.88104       -1.27564
N         -2.49789       -1.14619        1.64401
C         -1.16320       -1.02899        1.30788
C         -1.02659       -0.26471        0.17196
C          0.25220        0.15448       -0.50232
C          0.89560        1.45548        0.07458
C          2.40634        1.34435       -0.24657
C          2.62965       -0.15076       -0.29158
C          1.41482       -0.82963       -0.43671
C          3.83804       -0.84225       -0.23082
C          3.82104       -2.23758       -0.32046
C          2.60898       -2.92102       -0.47743
C          1.39895       -2.22128       -0.53818
O          3.26888        1.94599        0.70863
H         -4.68657        1.63823       -2.17970
H         -6.21164        0.61770       -0.51593
H         -5.31782       -0.73976        1.36783
H         -2.23450        1.33665       -2.01153
H         -2.85429       -1.67751        2.42399
H         -0.40522       -1.50946        1.91046
H          0.02272        0.33412       -1.56428
H          0.42511        2.36269       -0.31585
H          0.78640        1.45303        1.16549
H          2.61700        1.78667       -1.23768
H          4.77257       -0.30319       -0.10258
H          4.75233       -2.79531       -0.26589
H          2.60738       -4.00576       -0.54798
H          0.45901       -2.75542       -0.64843
C          3.28353        3.36114        0.64632
H          4.03225        3.70464        1.36507
H          3.56138        3.71741       -0.35826
H          2.31048        3.79843        0.91298

Conformer 2
Energy: -826.11242 Hartree (Rel:  0.6 kcal/mol)
XYZ coordinates for conf 2:
C         -4.24466        1.27903        0.62279
C         -4.92289        0.43992       -0.28739
C         -4.26837       -0.61138       -0.92141
C         -2.91394       -0.80496       -0.62477
C         -2.20678        0.02787        0.29100
C         -2.89948        1.08277        0.91501
N         -2.02038       -1.74656       -1.08719
C         -0.78815       -1.53822       -0.49861
C         -0.85114       -0.46401        0.35795
C          0.27186        0.12426        1.18337
C          1.23924       -0.90755        1.84426
C          2.50265       -0.96907        0.96390
C          2.50071        0.38385        0.27307
C          1.23616        0.98070        0.36618
C          3.54224        1.03137       -0.39576
C          3.30123        2.27298       -0.99301
C          2.03209        2.85810       -0.91595
C          0.99359        2.21684       -0.23300
O          2.34991       -2.06877        0.05161
H         -4.78552        2.09147        1.10097
H         -5.97432        0.61768       -0.49685
H         -4.78935       -1.25848       -1.62230
H         -2.39130        1.73869        1.61797
H         -2.23050       -2.48189       -1.74508
H          0.05786       -2.16898       -0.73190
H         -0.18932        0.73926        1.96595
H          1.52499       -0.54358        2.83726
H          0.79369       -1.89801        1.96417
H          3.41669       -1.13986        1.55330
H          4.53439        0.58904       -0.44096
H          4.10433        2.78886       -1.51253
H          1.85563        3.82529       -1.37962
H          0.01271        2.68114       -0.16582
C          3.48519       -2.32876       -0.75305
```

```
H          3.28358       -3.25622       -1.29593
H          4.38954       -2.46410       -0.13878
H          3.67297       -1.52661       -1.47993

Conformer 3
Energy: -826.11407 Hartree (Rel:  0.6 kcal/mol)
XYZ coordinates for conf 3:
C         -4.28290        1.02664        0.96252
C         -4.94517        0.35955       -0.09081
C         -4.25153       -0.47138       -0.96464
C         -2.87430       -0.62026       -0.76167
C         -2.18258        0.04180        0.29482
C         -2.91527        0.87547        1.16102
N         -1.94151       -1.36334       -1.45242
C         -0.69966       -1.19413       -0.87237
C         -0.79497       -0.34414        0.20509
C          0.33252        0.12220        1.09994
C          1.27617       -1.00683        1.62338
C          2.54824       -0.97752        0.73674
C          2.54712        0.42870        0.18213
C          1.30970        1.04825        0.38178
C          3.58084        1.08975       -0.48291
C          3.36781        2.39341       -0.93768
C          2.13016        3.01835       -0.73342
C          1.09347        2.34910       -0.07725
O          2.50903       -1.89432       -0.36687
H         -4.85468        1.66881        1.62728
H         -6.01514        0.49728       -0.22165
H         -4.75995       -0.98582       -1.77604
H         -2.41992        1.39721        1.97650
H         -2.13155       -1.94353       -2.25553
H          0.17579       -1.69027       -1.26807
H         -0.11911        0.64185        1.95334
H          1.56588       -0.78303        2.65663
H          0.79345       -1.98766        1.62259
H          3.45239       -1.19426        1.32588
H          4.53840        0.59974       -0.64186
H          4.16437        2.92688       -1.44948
H          1.97580        4.03432       -1.08794
H          0.13241        2.83588        0.06972
C          2.81450       -3.22985       -0.00335
H          2.80575       -3.82061       -0.92317
H          2.07815       -3.65315        0.69522
H          3.81144       -3.30026        0.45861

Conformer 4
Energy: -826.11167 Hartree (Rel:  1.0 kcal/mol)
XYZ coordinates for conf 4:
C         -4.32244       -0.03734       -1.37670
C         -4.97766       -0.15466       -0.13209
C         -4.25877       -0.31992        1.04780
C         -2.86272       -0.36450        0.95465
C         -2.17857       -0.24958       -0.28992
C         -2.93525       -0.08226       -1.46452
N         -1.90237       -0.51658        1.93172
C         -0.65010       -0.50503        1.34740
C         -0.76454       -0.34404       -0.01266
C          0.34658       -0.23949       -1.03059
C          0.77298        1.22879       -1.36407
C          2.05712        1.51038       -0.54174
C          2.61645        0.12572       -0.30075
C          1.65630       -0.85588       -0.56440
C          3.89054       -0.22087        0.15128
C          4.20153       -1.57165        0.32652
C          3.24214       -2.55776        0.06000
C          1.96347       -2.20599       -0.38157
O          1.81043        2.11325        0.73311
H         -4.91464        0.09129       -2.27889
H         -6.06299       -0.11499       -0.09390
H         -4.76253       -0.41010        2.00663
H         -2.44334        0.01158       -2.42971
```

H	-2.08065	-0.63383	2.91761
H	0.23730	-0.60743	1.95517
H	0.00252	-0.73162	-1.95009
H	1.01629	1.30249	-2.43080
H	-0.02058	1.95119	-1.15527
H	2.75654	2.15255	-1.09987
H	4.63105	0.54721	0.36068
H	5.19187	-1.86049	0.66846
H	3.49595	-3.60598	0.19574
H	1.22048	-2.97446	-0.58056
C	1.55390	3.50345	0.66219
H	1.43498	3.85875	1.68945
H	0.63511	3.73334	0.10278
H	2.39005	4.04431	0.19080

Conformer 5
Energy: -826.11317 Hartree (Rel: 1.1 kcal/mol)
XYZ coordinates for conf 5:

C	-4.43756	-0.33736	-1.29953
C	-5.07149	-0.08468	-0.06397
C	-4.33160	0.11570	1.09728
C	-2.93675	0.05829	0.99444
C	-2.27375	-0.19678	-0.24078
C	-3.05153	-0.39381	-1.39689
N	-1.95906	0.21286	1.95387
C	-0.71672	0.06096	1.36870
C	-0.85456	-0.19211	0.02499
C	0.23992	-0.38916	-0.99699
C	0.73278	0.92703	-1.68654
C	2.03412	1.33458	-0.96978
C	2.55810	0.00948	-0.44108
C	1.53281	-0.94525	-0.41824
C	3.84328	-0.31944	-0.00334
C	4.08877	-1.60871	0.48030
C	3.05858	-2.55555	0.52134
C	1.77551	-2.22948	0.07020
O	1.68931	2.25009	0.07875
H	-5.04563	-0.48822	-2.18763
H	-6.15653	-0.04521	-0.01833
H	-4.81845	0.31081	2.04917
H	-2.57660	-0.58647	-2.35591
H	-2.12043	0.39413	2.93298
H	0.18226	0.15560	1.96023
H	-0.14642	-1.07349	-1.76374
H	0.96044	0.72170	-2.73882
H	-0.00545	1.73158	-1.64718
H	2.74815	1.83075	-1.64589
H	4.65024	0.40755	-0.05069
H	5.08535	-1.87900	0.81927
H	3.26101	-3.55637	0.89402
H	0.98139	-2.97193	0.09147
C	2.79979	2.83590	0.72971
H	2.40485	3.59259	1.41344
H	3.47793	3.32405	0.01104
H	3.37853	2.10347	1.31051

Conformer 6
Energy: -826.11310 Hartree (Rel: 1.5 kcal/mol)
XYZ coordinates for conf 6:

C	4.31416	-1.19619	-1.31778
C	5.19742	-0.56987	-0.41190
C	4.72223	0.24850	0.60759
C	3.33668	0.42699	0.70142
C	2.42532	-0.19234	-0.20146
C	2.93913	-1.01612	-1.22053
N	2.58648	1.16634	1.59051
C	1.24759	1.03830	1.27584
C	1.09455	0.21510	0.18398
C	-0.19407	-0.23347	-0.45180
C	-0.85335	-1.47640	0.21842
C	-2.35469	-1.38130	-0.11480

C	-2.57008	0.11818	-0.25480
C	-1.34419	0.76855	-0.44659
C	-3.76194	0.84272	-0.22653
C	-3.71780	2.22982	-0.39867
C	-2.49479	2.87894	-0.60446
C	-1.30074	2.15110	-0.62986
O	-3.12650	-2.00521	0.90753
H	4.71920	-1.83020	-2.10205
H	6.26755	-0.73006	-0.51212
H	5.40106	0.73086	1.30593
H	2.27075	-1.50737	-1.92353
H	2.95397	1.73629	2.33737
H	0.49875	1.55400	1.86046
H	0.02503	-0.49041	-1.50001
H	-0.40363	-2.42370	-0.09348
H	-0.75142	-1.39275	1.30645
H	-2.56365	-1.88054	-1.07537
H	-4.71330	0.34456	-0.06120
H	-4.63808	2.80746	-0.37063
H	-2.47223	3.95724	-0.73970
H	-0.35007	2.65729	-0.77481
C	-4.27927	-2.68801	0.44666
H	-4.73742	-3.16138	1.31947
H	-5.01529	-2.01281	-0.01291
H	-4.02132	-3.46806	-0.28602

164{10-rev}(*cis*) matched with exp ^{13}C shifts of the minor product ($CDCl_3$)

	Conf1	Conf2	Conf3	Conf4	Conf5	Conf6
Rel energy (kcal/mol):	0.00	0.56	0.61	1.04	1.10	1.50

C-nom	iGau	Exp	Calc	diff		1	2	3	4	5	6	
C	14	145.60	146.70	1.10	[	143.92	150.64	149.94	146.87	148.23	144.58	]
C	13	142.80	142.25	-0.55	[	143.88	138.51	140.50	143.65	141.89	145.34	]
C	4	136.50	135.29	-1.21	[	135.43	135.15	134.88	135.51	135.44	135.37	]
C	17	128.20	127.65	-0.55	[	127.01	128.34	128.35	128.16	128.32	126.69	]
C	5	126.80	127.06	0.26	[	127.21	127.18	126.82	126.48	126.95	127.26	]
C	16	126.54	125.55	-0.99	[	125.92	124.58	125.38	126.04	125.20	125.89	]
C	15	125.29	124.70	-0.59	[	124.02	125.36	125.77	125.50	124.92	123.06	]
C	18	124.30	124.15	-0.15	[	123.76	124.60	123.84	124.81	125.60	124.08	]
C	8	121.90	123.76	1.86	[	122.64	125.25	125.36	123.62	124.29	122.66	]
C	2	121.70	121.16	-0.54	[	121.43	120.84	120.82	121.09	121.01	121.39	]
C	1	119.18	118.89	-0.29	[	118.96	118.88	118.87	118.69	118.73	118.93	]
C	6	119.13	117.67	-1.46	[	117.75	117.47	117.45	117.92	117.87	117.71	]
C	9	118.70	119.28	0.58	[	118.21	119.39	120.67	121.33	120.94	118.12	]
C	3	111.10	110.21	-0.89	[	110.28	110.14	110.16	110.14	110.15	110.25	]
C	12	83.30	83.87	0.57	[	82.62	84.51	85.60	85.43	84.97	83.02	]
C	34	56.50	55.85	-0.65	[	56.14	54.36	56.53	55.89	54.27	59.50	]
C	11	41.20	44.10	2.90	[	47.15	43.17	38.95	37.76	43.57	48.41	]
C	10	39.20	39.87	0.67	[	38.83	40.86	40.78	41.37	41.29	38.00	]

13C chem shifts: RMSD=1.09ppm (MAE=0.88) N=18 {-1.46 2.90}

Fractions: 0.464 0.179 0.166 0.081 0.073 0.037

Conformer 1
Energy: -826.11232 Hartree (Rel: 0.0 kcal/mol)
XYZ coordinates for conf 1:

C	-4.26963	1.04925	-1.36707
C	-5.13965	0.46825	-0.41914
C	-4.64912	-0.29218	0.63731
C	-3.26172	-0.45895	0.72514
C	-2.36361	0.11540	-0.21976
C	-2.89269	0.88104	-1.27564
N	-2.49789	-1.14619	1.64401
C	-1.16320	-1.02899	1.30788
C	-1.02659	-0.26471	0.17196
C	0.25220	0.15448	-0.50232
C	0.89560	1.45548	0.07458
C	2.40634	1.34435	-0.24657
C	2.62965	-0.15076	-0.29158
C	1.41482	-0.82963	-0.43671
C	3.83804	-0.84225	-0.23082
C	3.82104	-2.23758	-0.32046
C	2.60898	-2.92102	-0.47743
C	1.39895	-2.22128	-0.53818
O	3.26888	1.94599	0.70863
H	-4.68657	1.63823	-2.17970
H	-6.21164	0.61770	-0.51593
H	-5.31782	-0.73976	1.36783
H	-2.23450	1.33665	-2.01153
H	-2.85429	-1.67751	2.42399
H	-0.40522	-1.50946	1.91046
H	0.02272	0.33412	-1.56428
H	0.42511	2.36269	-0.31585
H	0.78640	1.45303	1.16549
H	2.61700	1.78667	-1.23768
H	4.77257	-0.30319	-0.10258
H	4.75233	-2.79531	-0.26589
H	2.60738	-4.00576	-0.54798
H	0.45901	-2.75542	-0.64843
C	3.28353	3.36114	0.64632
H	4.03225	3.70464	1.36507
H	3.56138	3.71741	-0.35826
H	2.31048	3.79843	0.91298

Conformer 2

Energy: -826.11242 Hartree (Rel: 0.6 kcal/mol)
XYZ coordinates for conf 2:

C	-4.24466	1.27903	0.62279
C	-4.92289	0.43992	-0.28739
C	-4.26837	-0.61138	-0.92141
C	-2.91394	-0.80496	-0.62477
C	-2.20678	0.02787	0.29100
C	-2.89948	1.08277	0.91501
N	-2.02038	-1.74656	-1.08719
C	-0.78815	-1.53822	-0.49861
C	-0.85114	-0.46401	0.35795
C	0.27186	0.12426	1.18337
C	1.23924	-0.90755	1.84426
C	2.50265	-0.96907	0.96390
C	2.50071	0.38385	0.27307
C	1.23616	0.98070	0.36618
C	3.54224	1.03137	-0.39576
C	3.30123	2.27298	-0.99301
C	2.03209	2.85810	-0.91595
C	0.99359	2.21684	-0.23300
O	2.34991	-2.06877	0.05161
H	-4.78552	2.09147	1.10097
H	-5.97432	0.61768	-0.49685
H	-4.78935	-1.25848	-1.62230
H	-2.39130	1.73869	1.61797
H	-2.23050	-2.48189	-1.74508
H	0.05786	-2.16898	-0.73190
H	-0.18932	0.73926	1.96595
H	1.52499	-0.54358	2.83726
H	0.79369	-1.89801	1.96417
H	3.41669	-1.13986	1.55330
H	4.53439	0.58904	-0.44096
H	4.10433	2.78886	-1.51253
H	1.85563	3.82529	-1.37962
H	0.01271	2.68114	-0.16582
C	3.48519	-2.32876	-0.75305
H	3.28358	-3.25622	-1.29593
H	4.38954	-2.46410	-0.13878
H	3.67297	-1.52661	-1.47993

Conformer 3
Energy: -826.11407 Hartree (Rel: 0.6 kcal/mol)
XYZ coordinates for conf 3:

C	-4.28290	1.02664	0.96252
C	-4.94517	0.35955	-0.09081
C	-4.25153	-0.47138	-0.96464
C	-2.87430	-0.62026	-0.76167
C	-2.18258	0.04180	0.29482
C	-2.91527	0.87547	1.16102
N	-1.94151	-1.36334	-1.45242
C	-0.69966	-1.19413	-0.87237
C	-0.79497	-0.34414	0.20509
C	0.33252	0.12220	1.09994
C	1.27617	-1.00683	1.62338
C	2.54824	-0.97752	0.73674
C	2.54712	0.42870	0.18213
C	1.30970	1.04825	0.38178
C	3.58084	1.08975	-0.48291
C	3.36781	2.39341	-0.93768
C	2.13016	3.01835	-0.73342
C	1.09347	2.34910	-0.07725
O	2.50903	-1.89432	-0.36687
H	-4.85468	1.66881	1.62728
H	-6.01514	0.49728	-0.22165
H	-4.75995	-0.98582	-1.77604
H	-2.41992	1.39721	1.97650
H	-2.13155	-1.94353	-2.25553
H	0.17579	-1.69027	-1.26807
H	-0.11911	0.64185	1.95334
H	1.56588	-0.78303	2.65663
H	0.79345	-1.98766	1.62259

```
H          3.45239       -1.19426        1.32588
H          4.53840        0.59974       -0.64186
H          4.16437        2.92688       -1.44948
H          1.97580        4.03432       -1.08794
H          0.13241        2.83588        0.06972
C          2.81450       -3.22985       -0.00335
H          2.80575       -3.82061       -0.92317
H          2.07815       -3.65315        0.69522
H          3.81144       -3.30026        0.45861

Conformer 4
Energy: -826.11167 Hartree (Rel:  1.0 kcal/mol)
XYZ coordinates for conf 4:
C         -4.32244       -0.03734       -1.37670
C         -4.97766       -0.15466       -0.13209
C         -4.25877       -0.31992        1.04780
C         -2.86272       -0.36450        0.95465
C         -2.17857       -0.24958       -0.28992
C         -2.93525       -0.08226       -1.46452
N         -1.90237       -0.51658        1.93172
C         -0.65010       -0.50503        1.34740
C         -0.76454       -0.34404       -0.01266
C          0.34658       -0.23949       -1.03059
C          0.77298        1.22879       -1.36407
C          2.05712        1.51038       -0.54174
C          2.61645        0.12572       -0.30075
C          1.65630       -0.85588       -0.56440
C          3.89054       -0.22087        0.15128
C          4.20153       -1.57165        0.32652
C          3.24214       -2.55776        0.06000
C          1.96347       -2.20599       -0.38157
O          1.81043        2.11325        0.73311
H         -4.91464        0.09129       -2.27889
H         -6.06299       -0.11499       -0.09390
H         -4.76253       -0.41010        2.00663
H         -2.44334        0.01158       -2.42971
H         -2.08065       -0.63383        2.91761
H          0.23730       -0.60743        1.95517
H          0.00252       -0.73162       -1.95009
H          1.01629        1.30249       -2.43080
H         -0.02058        1.95119       -1.15527
H          2.75654        2.15255       -1.09987
H          4.63105        0.54721        0.36068
H          5.19187       -1.86049        0.66846
H          3.49595       -3.60598        0.19574
H          1.22048       -2.97446       -0.58056
C          1.55390        3.50345        0.66219
H          1.43498        3.85875        1.68945
H          0.63511        3.73334        0.10278
H          2.39005        4.04431        0.19080

Conformer 5
Energy: -826.11317 Hartree (Rel:  1.1 kcal/mol)
XYZ coordinates for conf 5:
C         -4.43756       -0.33736       -1.29953
C         -5.07149       -0.08468       -0.06397
C         -4.33160        0.11570        1.09728
C         -2.93675        0.05829        0.99444
C         -2.27375       -0.19678       -0.24078
C         -3.05153       -0.39381       -1.39689
N         -1.95906        0.21286        1.95387
C         -0.71672        0.06096        1.36870
C         -0.85456       -0.19211        0.02499
C          0.23992       -0.38916       -0.99699
C          0.73278        0.92703       -1.68654
C          2.03412        1.33458       -0.96978
C          2.55810        0.00948       -0.44108
C          1.53281       -0.94525       -0.41824
C          3.84328       -0.31944       -0.00334
C          4.08877       -1.60871        0.48030
C          3.05858       -2.55555        0.52134
```

C	1.77551	-2.22948	0.07020
O	1.68931	2.25009	0.07875
H	-5.04563	-0.48822	-2.18763
H	-6.15653	-0.04521	-0.01833
H	-4.81845	0.31081	2.04917
H	-2.57660	-0.58647	-2.35591
H	-2.12043	0.39413	2.93298
H	0.18226	0.15560	1.96023
H	-0.14642	-1.07349	-1.76374
H	0.96044	0.72170	-2.73882
H	-0.00545	1.73158	-1.64718
H	2.74815	1.83075	-1.64589
H	4.65024	0.40755	-0.05069
H	5.08535	-1.87900	0.81927
H	3.26101	-3.55637	0.89402
H	0.98139	-2.97193	0.09147
C	2.79979	2.83590	0.72971
H	2.40485	3.59259	1.41344
H	3.47793	3.32405	0.01104
H	3.37853	2.10347	1.31051

Conformer 6
Energy: -826.11310 Hartree (Rel: 1.5 kcal/mol)
XYZ coordinates for conf 6:

C	4.31416	-1.19619	-1.31778
C	5.19742	-0.56987	-0.41190
C	4.72223	0.24850	0.60759
C	3.33668	0.42699	0.70142
C	2.42532	-0.19234	-0.20146
C	2.93913	-1.01612	-1.22053
N	2.58648	1.16634	1.59051
C	1.24759	1.03830	1.27584
C	1.09455	0.21510	0.18398
C	-0.19407	-0.23347	-0.45180
C	-0.85335	-1.47640	0.21842
C	-2.35469	-1.38130	-0.11480
C	-2.57008	0.11818	-0.25480
C	-1.34419	0.76855	-0.44659
C	-3.76194	0.84272	-0.22653
C	-3.71780	2.22982	-0.39867
C	-2.49479	2.87894	-0.60446
C	-1.30074	2.15110	-0.62986
O	-3.12650	-2.00521	0.90753
H	4.71920	-1.83020	-2.10205
H	6.26755	-0.73006	-0.51212
H	5.40106	0.73086	1.30593
H	2.27075	-1.50737	-1.92353
H	2.95397	1.73629	2.33737
H	0.49875	1.55400	1.86046
H	0.02503	-0.49041	-1.50001
H	-0.40363	-2.42370	-0.09348
H	-0.75142	-1.39275	1.30645
H	-2.56365	-1.88054	-1.07537
H	-4.71330	0.34456	-0.06120
H	-4.63808	2.80746	-0.37063
H	-2.47223	3.95724	-0.73970
H	-0.35007	2.65729	-0.77481
C	-4.27927	-2.68801	0.44666
H	-4.73742	-3.16138	1.31947
H	-5.01529	-2.01281	-0.01291
H	-4.02132	-3.46806	-0.28602

Originally assigned (incorrect) structure of 168{12'} ($CDCl_3$)

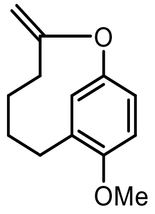

					Conf1
		Rel energy (kcal/mol):			0.00
**C-nom**	**iGau**	**Exp**	**Calc**	**diff**	**1**
C	12	151.80	166.25	14.45	[166.25]
C	6	149.10	152.56	3.46	[152.56]
C	3	143.00	151.33	8.33	[151.33]
C	2	132.20	132.23	0.03	[132.23]
C	1	117.00	130.12	13.12	[130.12]
C	5	112.70	116.90	4.20	[116.90]
C	4	111.90	110.94	-0.96	[110.94]
C	13	111.50	94.41	-17.09	[94.41]
C	15	55.90	55.49	-0.41	[55.49]
C	10	39.00	35.03	-3.97	[35.03]
C	8	29.70	33.52	3.82	[33.52]
C	9	28.70	32.01	3.31	[32.01]
C	11	26.90	30.04	3.14	[30.04]

13C chem shifts: RMSD=7.96ppm (MAE=5.87) N=13 {-17.09 14.45}

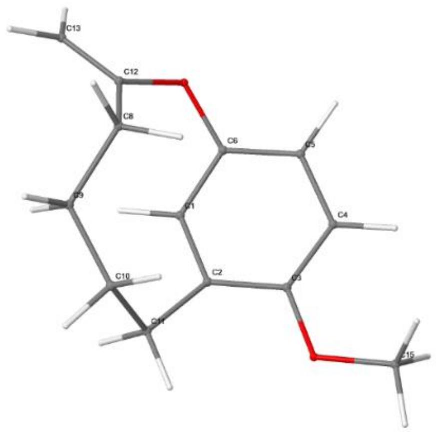

Conformer 1
Energy: -655.38304 Hartree (Rel: 0.0 kcal/mol)
XYZ coordinates for conf 1:

C	-0.32342	-0.21501	1.04124
C	0.87357	0.44756	0.81597
C	1.92816	-0.28158	0.23011
C	1.70981	-1.60727	-0.18199
C	0.41811	-2.15556	-0.16856
C	-0.61054	-1.41076	0.39970
O	-1.94606	-1.68104	0.15600
C	-2.00649	0.54524	-1.06257
C	-1.64827	1.93090	-0.38997
C	-0.16067	2.40852	-0.33744
C	0.75888	1.95282	0.84170
C	-2.68309	-0.54570	-0.21787
C	-3.97801	-0.52758	0.11341
O	3.10778	0.39388	0.05549
C	4.17039	-0.26815	-0.61606
H	-1.13805	0.32195	1.51225

H	2.51513	-2.18547	-0.62028
H	0.21478	-3.10562	-0.65291
H	-2.72007	0.75995	-1.86587
H	-1.12316	0.12632	-1.55360
H	-2.17269	2.69454	-0.97650
H	-2.09777	1.98622	0.60881
H	-0.18151	3.50654	-0.31599
H	0.32885	2.13512	-1.28228
H	1.73263	2.43978	0.73839
H	0.32122	2.28981	1.79062
H	-4.41484	-1.31449	0.71922
H	-4.62465	0.26993	-0.23558
H	4.98700	0.45394	-0.67110
H	4.50840	-1.15318	-0.06169
H	3.88046	-0.56863	-1.63129

Revised structure of 168{12'}, i.e 169{12'-rev} (CDCl$_3$)

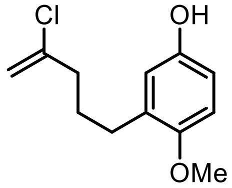


```
                                                  Conf1  Conf2  Conf3  Conf4  Conf5  Conf6  Conf7  Conf8  Conf9
Conf10 Conf11 Conf12 Conf13 Conf14 Conf15 Conf16 Conf17 Conf18 Conf19 Conf20 Conf21 Conf22 Conf23
Conf24 Conf25 Conf26 Conf27 Conf28 Conf29 Conf30 Conf31 Conf32 Conf33 Conf34 Conf35 Conf36 Conf37
Conf38 Conf39 Conf40 Conf41 Conf42 Conf43 Conf44 Conf45 Conf46 Conf47 Conf48 Conf49 Conf50
                Rel energy (kcal/mol):   0.00   0.00   0.01   0.01   0.03   0.03   0.06   0.06   0.23
0.23   0.25   0.25   0.26   0.26   0.33   0.33   0.46   0.51   0.51   0.69   0.70   0.83   0.84   0.84
0.86   0.86   0.88   0.88   0.90   0.90   0.91   0.92   0.92   0.92   0.92   0.92   0.97   0.98   0.98
1.02   1.04   1.04   1.04   1.09   1.09   1.12   1.12   1.18   1.35   1.36

 C-nom     iGau   Exp    Calc   diff        1      2      3      4      5      6      7      8      9
10     11     12     13     14     15     16     17     18     19     20     21     22     23     24
25     26     27     28     29     30     31     32     33     34     35     36     37     38     39
40     41     42     43     44     45     46     47     48     49     50
 C          3  151.80 149.61  -2.19   [ 149.74 149.75 149.66 149.66 149.48 149.48 149.59 149.59 149.47
149.47 149.64 149.64 149.78 149.78 149.60 149.60 149.37 149.54 149.54 149.43 149.60 149.72 149.50
149.50 149.76 149.76 149.32 149.32 149.82 149.82 149.58 149.49 149.48 149.31 149.31 149.59 150.23
149.66 149.66 150.22 149.45 149.76 149.76 149.29 149.29 149.59 149.58 150.22 149.92 150.08 ]
 C          6  149.10 148.94  -0.16   [ 148.98 148.98 149.07 149.07 148.94 148.94 148.84 148.84 148.95
148.95 149.11 149.11 148.98 148.98 148.84 148.84 148.57 148.73 148.73 148.57 148.71 148.65 149.10
149.10 149.08 149.08 148.99 148.99 149.06 149.06 148.50 149.07 149.07 148.95 148.95 148.94 149.12
148.91 148.91 149.14 149.05 148.99 148.99 148.93 148.93 148.84 148.84 149.15 149.18 149.26 ]
 C         12  143.00 141.67  -1.33   [ 142.02 142.01 142.05 142.05 141.97 141.96 141.99 141.98 140.97
140.98 141.01 141.01 141.07 141.07 140.97 140.97 141.89 141.99 141.99 141.24 141.36 141.12 142.30
142.31 142.24 142.24 142.27 142.27 142.33 142.33 140.99 142.41 142.42 142.35 142.35 142.15 142.10
142.24 142.24 142.20 140.70 140.69 140.69 140.60 140.59 140.99 140.99 141.39 141.08 141.01 ]
 C          4  132.20 131.88  -0.32   [ 132.49 132.50 132.44 132.45 131.46 131.45 131.49 131.49 131.64
131.64 132.63 132.63 132.61 132.61 131.62 131.62 130.08 131.07 131.07 130.24 131.22 130.88 132.62
132.61 132.38 132.38 131.59 131.59 132.41 132.41 129.90 132.64 132.64 131.64 131.64 131.38 132.14
131.42 131.42 131.99 132.63 132.48 132.48 131.60 131.60 131.50 131.50 132.26 130.91 132.00 ]
 C          5  117.00 116.99  -0.01   [ 117.23 117.23 117.35 117.35 116.34 116.35 116.28 116.28 116.44
116.44 117.44 117.44 117.15 117.15 116.18 116.18 117.61 118.61 118.61 117.62 118.63 118.38 117.40
117.40 117.19 117.19 116.41 116.41 117.21 117.21 117.40 117.43 117.43 116.45 116.45 116.21 117.05
116.18 116.18 117.12 117.45 117.16 117.16 116.44 116.44 116.16 116.16 117.11 116.14 117.28 ]
 C         13  112.70 112.75   0.05   [ 112.29 112.29 112.24 112.24 112.31 112.31 112.28 112.28 113.54
113.53 113.47 113.47 113.44 113.44 113.49 113.49 112.24 112.13 112.13 113.43 113.33 113.10 112.34
112.34 112.38 112.38 112.35 112.35 112.39 112.39 113.19 112.39 112.39 112.45 112.45 112.42 112.03
112.42 112.41 111.97 114.14 113.84 113.84 114.23 114.23 113.51 113.51 113.33 113.04 113.11 ]
 C          1  111.90 111.72  -0.18   [ 111.11 111.11 111.06 111.07 112.49 112.50 112.48 112.48 112.38
112.38 110.98 110.98 111.04 111.04 112.42 112.42 112.51 111.10 111.10 112.51 111.06 111.21 111.09
111.09 111.10 111.10 112.50 112.50 111.08 111.08 112.62 111.07 111.07 112.49 112.49 112.51 111.17
112.49 112.49 111.30 111.07 111.05 111.05 112.50 112.50 112.45 112.45 111.10 112.63 111.21 ]
 C          2  111.50 110.38  -1.12   [ 109.87 109.87 109.86 109.87 111.00 111.00 111.02 111.02 110.92
110.93 109.79 109.79 109.83 109.83 110.97 110.97 111.07 109.94 109.94 111.07 109.94 110.05 109.87
109.88 109.91 109.91 111.00 111.00 109.92 109.92 111.18 109.82 109.82 110.97 110.98 111.03 109.66
111.04 111.04 109.75 109.83 109.88 109.88 110.96 110.96 111.01 111.01 109.62 110.78 109.65 ]
 C         15   55.90  55.27  -0.63   [  55.26  55.26  55.27  55.27  55.30  55.29  55.29  55.30  55.25
55.25  55.22  55.22  55.30  55.30  55.33  55.33  55.27  55.23  55.23  55.29  55.26  55.24  55.27  55.27
55.31  55.31  55.30  55.30  55.25  55.25  55.27  55.18  55.17  55.20  55.20  55.34  55.19  55.28  55.28
55.19  55.25  55.21  55.21  55.29  55.29  55.25  55.25  55.17  55.23  55.19 ]
 C          8   39.00  39.24   0.24   [  40.11  40.11  40.12  40.11  40.09  40.09  40.07  40.07  39.03
39.02  39.10  39.10  39.17  39.17  39.16  39.15  40.00  40.08  40.08  38.97  39.01  39.11  38.27  38.27
38.33  38.33  38.22  38.22  38.34  38.34  39.10  38.32  38.32  38.24  38.24  38.34  40.28  38.36  38.36
40.31  35.86  35.58  35.58  35.84  35.85  35.56  35.56  39.13  39.18  39.28 ]
 C         11   29.70  32.41   2.71   [  33.23  33.23  33.20  33.20  33.12  33.13  33.17  33.17  32.94
32.94  33.07  33.07  33.18  33.18  33.06  33.06  32.32  32.39  32.39  32.22  32.24  32.46  31.44  31.45
```

31.43 31.43 31.35 31.35 31.01 31.01 32.46 30.90 30.90 30.90 30.91 31.30 28.53 30.94 30.93
28.65 30.66 30.33 30.33 30.59 30.59 30.19 30.19 28.40 28.25 28.50]
C 10 28.70 29.16 0.46 [30.62 30.62 30.62 30.62 30.60 30.60 30.56 30.56 28.33
28.33 28.34 28.34 28.42 28.43 28.46 28.46 28.00 28.04 28.04 25.52 25.58 25.77 29.49 29.49
29.52 29.52 29.47 29.47 29.27 29.27 25.70 29.35 29.35 29.22 29.22 29.55 32.33 29.26 29.26
32.35 25.60 25.56 25.56 25.57 25.57 25.62 25.63 29.91 29.98 30.10]
C 9 26.90 27.39 0.49 [29.16 29.16 29.16 29.17 29.13 29.13 29.13 29.12 26.19
26.19 26.23 26.23 26.24 26.24 26.22 26.22 26.79 26.72 26.72 23.77 23.73 23.96 27.97 27.97
27.82 27.82 27.96 27.96 27.97 27.96 24.01 28.07 28.07 28.08 28.09 27.79 26.16 27.95 27.94
26.26 25.29 24.84 24.84 25.26 25.26 24.79 24.79 23.09 23.06 23.23]

13C chem shifts: RMSD=1.12ppm (MAE=0.76) N=13 {-2.19 2.71}

Fractions: 0.049 0.049 0.048 0.048 0.047 0.047 0.044 0.044
0.033 0.033 0.032 0.032 0.032 0.032 0.028 0.028 0.023 0.021 0.021 0.015 0.015 0.012 0.012
0.012 0.012 0.012 0.011 0.011 0.011 0.011 0.011 0.010 0.010 0.010 0.010 0.010 0.010 0.009
0.009 0.009 0.009 0.008 0.008 0.008 0.008 0.007 0.007 0.007 0.005 0.005

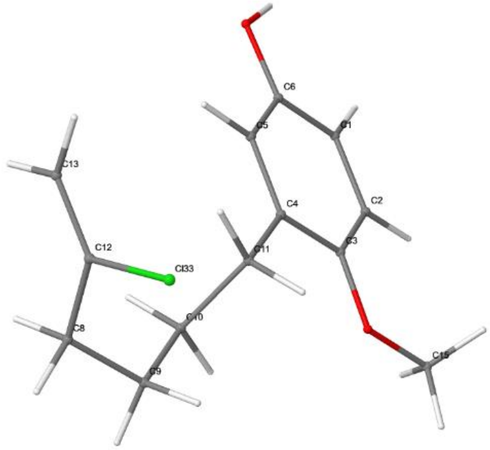

Conformer 1
Energy: -1116.22583 Hartree (Rel: 0.0 kcal/mol)
XYZ coordinates for conf 1:

C	-4.49886	-0.12116	-0.43056
C	-3.70193	1.02976	-0.39972
C	-2.38261	0.96303	0.05248
C	-1.84236	-0.26843	0.48845
C	-2.65374	-1.40134	0.45149
C	-3.97649	-1.34037	-0.00563
O	-4.69992	-2.50757	-0.00593
C	3.07997	-0.52557	-0.84157
C	2.06273	-0.48015	0.31509
C	0.61584	-0.39712	-0.18683
C	-0.40900	-0.35682	0.96528
C	4.51460	-0.61460	-0.40102
C	5.33584	-1.65042	-0.55884
O	-1.53430	2.04184	0.11332
C	-2.03339	3.31057	-0.28239
H	-5.52539	-0.05661	-0.78488
H	-0.18207	0.50226	1.60812
H	-2.26243	-2.35833	0.78648
H	2.87718	-1.39909	-1.47382
H	2.95080	0.36381	-1.47303
H	2.19109	-1.37509	0.93902
H	2.27796	0.38360	0.95671
H	0.39707	-1.25898	-0.83246
H	0.49236	0.50002	-0.80608
H	-0.29240	-1.25784	1.58049
H	6.36137	-1.64489	-0.20684
H	4.97725	-2.54474	-1.06065
H	-2.88408	3.62080	0.33843
H	-2.33867	3.31346	-1.33701
H	-1.21054	4.01428	-0.14403
H	-5.59245	-2.32337	-0.33994
H	-4.12857	1.96877	-0.73287

```
Cl         5.12363        0.84668        0.41917

Conformer 2
Energy: -1116.22583 Hartree (Rel:  0.0 kcal/mol)
XYZ coordinates for conf 2:
C          4.49890       -0.12106       -0.43050
C          3.70194        1.02984       -0.39966
C          2.38261        0.96307        0.05256
C          1.84245       -0.26841        0.48858
C          2.65386       -1.40129        0.45160
C          3.97659       -1.34029       -0.00557
O          4.70004       -2.50748       -0.00588
C         -3.07979       -0.52528       -0.84153
C         -2.06266       -0.48005        0.31520
C         -0.61574       -0.39706       -0.18668
C          0.40908       -0.35681        0.96542
C         -4.51451       -0.61450       -0.40126
C         -5.33585       -1.65004       -0.56037
O          1.53417        2.04180        0.11347
C          2.03263        3.31048       -0.28322
H          5.52543       -0.05648       -0.78483
H          0.29251       -1.25785        1.58060
H          2.26256       -2.35828        0.78659
H         -2.95061        0.36424       -1.47279
H         -2.87693       -1.39865       -1.47394
H         -2.27785        0.38365        0.95690
H         -2.19108       -1.37504        0.93905
H         -0.49224        0.50009       -0.80593
H         -0.39701       -1.25891       -0.83233
H          0.18218        0.50226        1.60830
H         -6.36152       -1.64469       -0.20878
H         -4.97721       -2.54393       -1.06293
H          2.33773        3.31277       -1.33790
H          2.88328        3.62156        0.33723
H          1.20945        4.01386       -0.14519
H          5.59260       -2.32323       -0.33980
H          4.12858        1.96885       -0.73280
Cl        -5.12356        0.84609        0.42003

Conformer 3
Energy: -1116.22581 Hartree (Rel:  0.0 kcal/mol)
XYZ coordinates for conf 3:
C          4.47030       -0.34641        0.46902
C          3.81433        0.87831        0.29450
C          2.49618        0.91441       -0.16409
C          1.81415       -0.28759       -0.46114
C          2.48637       -1.49558       -0.28216
C          3.80754       -1.53743        0.18198
O          4.38707       -2.77390        0.32674
C         -3.10277        0.19381        0.82571
C         -2.08810       -0.01892       -0.31436
C         -0.64123       -0.04921        0.19349
C          0.37993       -0.26241       -0.94271
C         -4.53629        0.24842        0.37595
C         -5.34020        1.30935        0.39751
O          1.78137        2.07125       -0.36094
C          2.42805        3.30935       -0.10738
H          5.49772       -0.35970        0.82596
H          0.25504        0.53743       -1.68261
H          1.98453       -2.43260       -0.50853
H         -2.98782       -0.60732        1.56829
H         -2.88443        1.13902        1.33828
H         -2.31585       -0.95767       -0.83517
H         -2.20559        0.78629       -1.05208
H         -0.52768       -0.85232        0.93479
H         -0.40961        0.89101        0.70930
H          0.15716       -1.20848       -1.45177
H         -6.36609        1.27527        0.04821
H         -4.96617        2.25515        0.77939
H          3.30938        3.44292       -0.74829
H          2.73215        3.39839        0.94384
```

```
H          1.69477       4.08469       -0.33682
H          5.29466      -2.66066        0.65168
H          4.34889       1.79329        0.52196
Cl        -5.16989      -1.29648       -0.24887

Conformer 4
Energy: -1116.22581 Hartree (Rel:  0.0 kcal/mol)
XYZ coordinates for conf 4:
C          4.47027      -0.34629       -0.46918
C          3.81426       0.87842       -0.29460
C          2.49618       0.91447        0.16421
C          1.81430      -0.28759        0.46122
C          2.48656      -1.49559        0.28231
C          3.80771      -1.53736       -0.18189
O          4.38734      -2.77376       -0.32673
C         -3.10269       0.19352       -0.82565
C         -2.08803      -0.01908        0.31449
C         -0.64109      -0.04928       -0.19334
C          0.38003      -0.26259        0.94279
C         -4.53612       0.24843       -0.37584
C         -5.33984       1.30951       -0.39705
O          1.78117       2.07124        0.36108
C          2.42736       3.30953        0.10706
H          5.49759      -0.35944       -0.82639
H          0.15737      -1.20873        1.45176
H          1.98475      -2.43260        0.50872
H         -2.88415       1.13858       -1.33845
H         -2.98780      -0.60779       -1.56805
H         -2.20560       0.78618        1.05212
H         -2.31568      -0.95783        0.83533
H         -0.40956       0.89102       -0.70905
H         -0.52758      -0.85228       -0.93478
H          0.25510       0.53706        1.68290
H         -6.36571       1.27539       -0.04768
H         -4.96586       2.25538       -0.77879
H          2.73093       3.39855       -0.94430
H          3.30885       3.44348        0.74765
H          1.69397       4.08471        0.33675
H          5.29517      -2.66036       -0.65094
H          4.34878       1.79334       -0.52237
Cl        -5.17012      -1.29654        0.24845

Conformer 5
Energy: -1116.22578 Hartree (Rel:  0.0 kcal/mol)
XYZ coordinates for conf 5:
C          4.47207      -0.35616       -0.47813
C          3.82359       0.86923       -0.30273
C          2.50457       0.91450        0.15979
C          1.81676      -0.28071        0.45854
C          2.48414      -1.49440        0.27859
C          3.80323      -1.54401       -0.18802
O          4.47924      -2.72503       -0.37192
C         -3.10135       0.21183       -0.82062
C         -2.08535      -0.00585        0.31731
C         -0.63894      -0.03291       -0.19196
C          0.38359      -0.24916        0.94249
C         -4.53493       0.25483       -0.36985
C         -5.34445       1.31159       -0.38045
O          1.79798       2.07653        0.35705
C          2.45332       3.31078        0.10600
H          5.49603      -0.39311       -0.83686
H          0.15696      -1.19347        1.45356
H          1.96183      -2.42125        0.51203
H         -2.88793       1.16256       -1.32491
H         -2.98266      -0.58212       -1.57033
H         -2.20283       0.79541        1.05926
H         -2.31199      -0.94741        0.83359
H         -0.40803       0.90912       -0.70464
H         -0.52573      -0.83321       -0.93644
H          0.26338       0.55191        1.68174
H         -6.37002       1.26850       -0.03119
```

H	-4.97564	2.26315	-0.75298
H	2.75689	3.40077	-0.94524
H	3.33605	3.43666	0.74646
H	1.72557	4.09052	0.33823
H	3.89691	-3.46275	-0.13001
H	4.36141	1.78199	-0.53177
Cl	-5.16032	-1.29980	0.23957

Conformer 6
Energy: -1116.22578 Hartree (Rel: 0.0 kcal/mol)
XYZ coordinates for conf 6:

C	4.47218	-0.35583	0.47789
C	3.82354	0.86946	0.30240
C	2.50451	0.91451	-0.16007
C	1.81686	-0.28082	-0.45866
C	2.48439	-1.49441	-0.27864
C	3.80353	-1.54381	0.18791
O	4.47970	-2.72473	0.37194
C	-3.10135	0.21225	0.82039
C	-2.08529	-0.00594	-0.31743
C	-0.63885	-0.03272	0.19184
C	0.38363	-0.24951	-0.94251
C	-4.53489	0.25486	0.36945
C	-5.34430	1.31173	0.37879
O	1.79773	2.07641	-0.35752
C	2.45225	3.31075	-0.10472
H	5.49616	-0.39257	0.83660
H	0.26336	0.55119	-1.68217
H	1.96214	-2.42133	-0.51189
H	-2.98257	-0.58128	1.57052
H	-2.88803	1.16329	1.32416
H	-2.31190	-0.94777	-0.83324
H	-2.20281	0.79497	-1.05975
H	-0.52565	-0.83264	0.93673
H	-0.40797	0.90958	0.70404
H	0.15700	-1.19406	-1.45314
H	-6.36978	1.26837	0.02932
H	-4.97552	2.26363	0.75045
H	3.33508	3.43800	-0.74477
H	2.75544	3.39958	0.94671
H	1.72408	4.09032	-0.33622
H	3.89759	-3.46254	0.12979
H	4.36128	1.78229	0.53135
Cl	-5.16027	-1.30033	-0.23845

Conformer 7
Energy: -1116.22573 Hartree (Rel: 0.1 kcal/mol)
XYZ coordinates for conf 7:

C	-4.50435	-0.11508	-0.43631
C	-3.70670	1.03191	-0.40299
C	-2.38534	0.96468	0.05011
C	-1.84620	-0.26530	0.48328
C	-2.66125	-1.39885	0.44408
C	-3.98328	-1.33627	-0.01258
O	-4.80265	-2.43731	-0.05933
C	3.07735	-0.51289	-0.84522
C	2.05907	-0.47930	0.31091
C	0.61260	-0.39161	-0.19145
C	-0.41317	-0.35708	0.96008
C	4.51153	-0.60863	-0.40452
C	5.33251	-1.64240	-0.57659
O	-1.53745	2.04397	0.11239
C	-2.03753	3.31407	-0.27836
H	-5.53034	-0.06458	-0.78751
H	-0.18636	0.49880	1.60721
H	-2.25445	-2.35108	0.78206
H	2.87424	-1.37884	-1.48772
H	2.94972	0.38380	-1.46654
H	2.18721	-1.38022	0.92632
H	2.27349	0.37819	0.96108
H	0.39353	-1.24893	-0.84309

```
H          0.49017        0.50962       -0.80480
H         -0.29630       -1.26081        1.57161
H          6.35785       -1.64218       -0.22404
H          4.97408       -2.52924       -1.09164
H         -2.88795        3.62171        0.34403
H         -2.34312        3.32108       -1.33281
H         -1.21479        4.01746       -0.13762
H         -4.31226       -3.20883        0.26641
H         -4.13103        1.97260       -0.73457
Cl         5.12033        0.84051        0.43673

Conformer 8
Energy: -1116.22573 Hartree (Rel:  0.1 kcal/mol)
XYZ coordinates for conf 8:
C          4.50432       -0.11537       -0.43641
C          3.70675        1.03168       -0.40332
C          2.38538        0.96464        0.04981
C          1.84617       -0.26522        0.48325
C          2.66114       -1.39882        0.44426
C          3.98317       -1.33643       -0.01244
O          4.80248       -2.43753       -0.05893
C         -3.07735       -0.51287       -0.84513
C         -2.05912       -0.47902        0.31101
C         -0.61264       -0.39155       -0.19138
C          0.41314       -0.35676        0.96012
C         -4.51161       -0.60851       -0.40461
C         -5.33270       -1.64207       -0.57744
O          1.53752        2.04398        0.11178
C          2.03797        3.31418       -0.27820
H          5.53032       -0.06501       -0.78763
H          0.29624       -1.26029        1.57192
H          2.25431       -2.35095        0.78248
H         -2.94968        0.38370       -1.46664
H         -2.87422       -1.37895       -1.48745
H         -2.27349        0.37868        0.96094
H         -2.18729       -1.37974        0.92670
H         -0.49016        0.50950       -0.80498
H         -0.39364       -1.24905       -0.84281
H          0.18643        0.49935        1.60697
H         -6.35815       -1.64190       -0.22518
H         -4.97423       -2.52871       -1.09281
H          2.34367        3.32172       -1.33262
H          2.88841        3.62124        0.34443
H          1.21537        4.01767       -0.13715
H          4.31192       -3.20902        0.26662
H          4.13113        1.97228       -0.73512
Cl        -5.12028        0.84027        0.43720

Conformer 9
Energy: -1116.22546 Hartree (Rel:  0.2 kcal/mol)
XYZ coordinates for conf 9:
C          3.92196        0.89543        0.68239
C          3.55738       -0.45156        0.60713
C          2.37841       -0.83260       -0.04142
C          1.54650        0.14263       -0.63114
C          1.93049        1.48277       -0.54619
C          3.10804        1.86856        0.10540
O          3.50533        3.17961        0.19973
C         -3.36767       -1.24107       -0.11433
C         -2.19114       -0.93085       -1.06290
C         -0.89952       -0.52850       -0.34176
C          0.25933       -0.25204       -1.32150
C         -3.84208       -0.07777        0.71315
C         -3.78525        0.04009        2.03799
O          1.95022       -2.13345       -0.15714
C          2.77036       -3.15957        0.38134
H          4.83684        1.19318        1.18528
H         -0.03792        0.54954       -2.00967
H          1.29627        2.24161       -1.00261
H         -4.21150       -1.62138       -0.70484
H         -3.07770       -2.03845        0.58144
```

H	-2.00703	-1.82766	-1.66923
H	-2.48913	-0.14019	-1.76374
H	-0.60004	-1.32655	0.34951
H	-1.07599	0.36625	0.26887
H	0.42970	-1.14885	-1.92954
H	-4.15600	0.91156	2.56600
H	-3.35373	-0.76389	2.62740
H	2.88917	-3.05401	1.46766
H	3.76254	-3.17156	-0.08848
H	2.25796	-4.09892	0.16531
H	2.84783	3.74010	-0.24229
H	4.20433	-1.19365	1.06062
Cl	-4.55770	1.25180	-0.23596

Conformer 10
Energy: -1116.22546 Hartree (Rel: 0.2 kcal/mol)
XYZ coordinates for conf 10:

C	-3.92183	0.89582	0.68224
C	-3.55755	-0.45125	0.60697
C	-2.37862	-0.83255	-0.04151
C	-1.54647	0.14252	-0.63113
C	-1.93018	1.48276	-0.54624
C	-3.10768	1.86878	0.10530
O	-3.50475	3.17990	0.19956
C	3.36769	-1.24125	-0.11396
C	2.19113	-0.93132	-1.06260
C	0.89950	-0.52878	-0.34157
C	-0.25929	-0.25236	-1.32138
C	3.84206	-0.07778	0.71321
C	3.78521	0.04057	2.03801
O	-1.95069	-2.13348	-0.15719
C	-2.77095	-3.15937	0.38154
H	-4.83666	1.19374	1.18511
H	-0.42970	-1.14921	-1.92936
H	-1.29575	2.24145	-1.00262
H	3.07775	-2.03850	0.58197
H	4.21153	-1.62170	-0.70438
H	2.48909	-0.14086	-1.76368
H	2.00701	-1.82831	-1.66864
H	1.07599	0.36603	0.26897
H	0.59994	-1.32674	0.34978
H	0.03807	0.54910	-2.00964
H	4.15597	0.91224	2.56567
H	3.35368	-0.76316	2.62775
H	-3.76313	-3.17135	-0.08826
H	-2.88969	-3.05354	1.46784
H	-2.25871	-4.09888	0.16574
H	-2.84682	3.74032	-0.24192
H	-4.20471	-1.19319	1.06043
Cl	4.55774	1.25156	-0.23630

Conformer 11
Energy: -1116.22542 Hartree (Rel: 0.3 kcal/mol)
XYZ coordinates for conf 11:

C	-3.92179	0.88489	0.67423
C	-3.54902	-0.46318	0.60564
C	-2.37016	-0.83790	-0.04187
C	-1.54420	0.14228	-0.63792
C	-1.93520	1.47794	-0.56009
C	-3.11525	1.85951	0.09130
O	-3.41803	3.19867	0.11537
C	3.37294	-1.22914	-0.11635
C	2.19765	-0.91765	-1.06613
C	0.90413	-0.51921	-0.34619
C	-0.25513	-0.24867	-1.32712
C	3.84538	-0.06742	0.71447
C	3.78390	0.04829	2.03932
O	-1.93278	-2.13584	-0.15355
C	-2.74400	-3.16509	0.39189
H	-4.84289	1.16349	1.18180
H	-0.42138	-1.14698	-1.93424

H	-1.31948	2.24928	-1.01501
H	3.08223	-2.02827	0.57713
H	4.21796	-1.60763	-0.70633
H	2.49562	-0.12464	-1.76425
H	2.01593	-1.81317	-1.67517
H	1.07665	0.37728	0.26292
H	0.60765	-1.31759	0.34607
H	0.03868	0.55378	-2.01542
H	4.15285	0.91889	2.57000
H	3.34974	-0.75637	2.62588
H	-3.73730	-3.18622	-0.07535
H	-2.86090	-3.05472	1.47800
H	-2.22563	-4.10193	0.17929
H	-4.25549	3.32350	0.58968
H	-4.19157	-1.20602	1.06401
Cl	4.56519	1.26275	-0.23004

Conformer 12
Energy: -1116.22542 Hartree (Rel: 0.3 kcal/mol)
XYZ coordinates for conf 12:

C	3.92186	0.88488	0.67409
C	3.54920	-0.46321	0.60529
C	2.37029	-0.83790	-0.04214
C	1.54420	0.14230	-0.63798
C	1.93515	1.47796	-0.56004
C	3.11521	1.85952	0.09133
O	3.41779	3.19871	0.11572
C	-3.37288	-1.22918	-0.11618
C	-2.19763	-0.91769	-1.06603
C	-0.90409	-0.51923	-0.34617
C	0.25512	-0.24866	-1.32716
C	-3.84537	-0.06737	0.71452
C	-3.78369	0.04855	2.03935
O	1.93309	-2.13588	-0.15407
C	2.74376	-3.16497	0.39248
H	4.84295	1.16349	1.18165
H	-0.03874	0.55381	-2.01541
H	1.31939	2.24933	-1.01485
H	-4.21788	-1.60778	-0.70610
H	-3.08209	-2.02820	0.57740
H	-2.01595	-1.81320	-1.67508
H	-2.49567	-0.12470	-1.76414
H	-0.60754	-1.31761	0.34606
H	-1.07658	0.37725	0.26296
H	0.42136	-1.14695	-1.93430
H	-4.15261	0.91919	2.56996
H	-3.34935	-0.75599	2.62593
H	2.86030	-3.05373	1.47854
H	3.73725	-3.18680	-0.07436
H	2.22517	-4.10180	0.18042
H	4.25604	3.32340	0.58868
H	4.19187	-1.20610	1.06341
Cl	-4.56541	1.26257	-0.23013

Conformer 13
Energy: -1116.22542 Hartree (Rel: 0.3 kcal/mol)
XYZ coordinates for conf 13:

C	4.05357	0.45815	0.67283
C	3.04304	1.39024	0.40656
C	1.85893	0.98951	-0.21530
C	1.67246	-0.36217	-0.58501
C	2.69230	-1.27276	-0.31335
C	3.88092	-0.87626	0.31320
O	4.82933	-1.84352	0.53950
C	-3.24701	-1.67320	0.04554
C	-2.07076	-1.44852	-0.92740
C	-0.77830	-0.98759	-0.24381
C	0.38517	-0.81165	-1.24115
C	-3.71973	-0.44083	0.76729
C	-3.65286	-0.20242	2.07540
O	0.82019	1.83743	-0.51220

C	0.96126	3.21152	-0.18499
H	4.97131	0.78283	1.15848
H	0.08697	-0.08151	-2.00346
H	2.57476	-2.31631	-0.59332
H	-4.09160	-2.10349	-0.50838
H	-2.95709	-2.40646	0.80866
H	-1.89039	-2.39464	-1.45520
H	-2.36647	-0.71943	-1.69271
H	-0.48720	-1.71918	0.52320
H	-0.94916	-0.03812	0.27602
H	0.56219	-1.76265	-1.75939
H	-4.02240	0.71270	2.52452
H	-3.21359	-0.94776	2.73229
H	1.81393	3.66500	-0.70730
H	1.08324	3.35853	0.89617
H	0.03899	3.69493	-0.51215
H	5.59801	-1.43487	0.96866
H	3.19811	2.42417	0.69241
Cl	-4.44822	0.79358	-0.29328

Conformer 14
Energy: -1116.22542 Hartree (Rel: 0.3 kcal/mol)
XYZ coordinates for conf 14:

C	-4.05365	0.45810	0.67271
C	-3.04313	1.39022	0.40651
C	-1.85898	0.98951	-0.21527
C	-1.67245	-0.36215	-0.58500
C	-2.69229	-1.27277	-0.31342
C	-3.88094	-0.87631	0.31308
O	-4.82933	-1.84358	0.53938
C	3.24691	-1.67319	0.04613
C	2.07071	-1.44870	-0.92696
C	0.77819	-0.98769	-0.24355
C	-0.38510	-0.81159	-1.24106
C	3.71966	-0.44058	0.76741
C	3.65210	-0.20124	2.07531
O	-0.82023	1.83746	-0.51208
C	-0.96162	3.21163	-0.18532
H	-4.97144	0.78275	1.15830
H	-0.56209	-1.76250	-1.75947
H	-2.57473	-2.31631	-0.59341
H	2.95685	-2.40616	0.80948
H	4.09146	-2.10375	-0.50760
H	2.36649	-0.71974	-1.69236
H	1.89042	-2.39491	-1.45462
H	0.94904	-0.03827	0.27635
H	0.48691	-1.71929	0.52338
H	-0.08677	-0.08135	-2.00321
H	4.02161	0.71409	2.52401
H	3.21224	-0.94602	2.73246
H	-1.08368	3.35895	0.89579
H	-1.81435	3.66476	-0.70783
H	-0.03943	3.69512	-0.51256
H	-5.59829	-1.43484	0.96795
H	-3.19826	2.42415	0.69232
Cl	4.44906	0.79299	-0.29358

Conformer 15
Energy: -1116.22530 Hartree (Rel: 0.3 kcal/mol)
XYZ coordinates for conf 15:

C	-4.05559	0.46714	0.68211
C	-3.04727	1.39646	0.41256
C	-1.86202	0.99633	-0.21263
C	-1.67557	-0.35277	-0.58199
C	-2.69699	-1.26448	-0.30605
C	-3.88326	-0.86849	0.32354
O	-4.90053	-1.74725	0.60515
C	3.24198	-1.67433	0.04045
C	2.06537	-1.44520	-0.93106
C	0.77430	-0.98375	-0.24525
C	-0.39016	-0.80348	-1.24073

C	3.71734	-0.44433	0.76456
C	3.65073	-0.20833	2.07311
O	-0.82465	1.84575	-0.51089
C	-0.96645	3.22000	-0.18381
H	-4.97637	0.77566	1.16747
H	-0.56868	-1.75289	-1.76179
H	-2.56349	-2.30681	-0.59281
H	2.95135	-2.40885	0.80212
H	4.08530	-2.10484	-0.51517
H	2.36169	-0.71462	-1.69468
H	1.88310	-2.38965	-1.46131
H	0.94691	-0.03561	0.27634
H	0.48305	-1.71631	0.52088
H	-0.09167	-0.07200	-2.00153
H	4.02195	0.70519	2.52406
H	3.20983	-0.95400	2.72855
H	-1.08671	3.36741	0.89743
H	-1.82005	3.67288	-0.70503
H	-0.04494	3.70378	-0.51256
H	-4.64486	-2.63279	0.30154
H	-3.20065	2.43101	0.69732
Cl	4.44831	0.79035	-0.29375

Conformer 16
Energy: -1116.22530 Hartree (Rel: 0.3 kcal/mol)
XYZ coordinates for conf 16:

C	4.05531	0.46686	0.68260
C	3.04717	1.39633	0.41285
C	1.86204	0.99639	-0.21267
C	1.67553	-0.35265	-0.58218
C	2.69678	-1.26450	-0.30610
C	3.88294	-0.86872	0.32384
O	4.90007	-1.74760	0.60558
C	-3.24219	-1.67427	0.03895
C	-2.06553	-1.44445	-0.93229
C	-0.77447	-0.98351	-0.24607
C	0.39021	-0.80316	-1.24125
C	-3.71735	-0.44500	0.76442
C	-3.65153	-0.21088	2.07335
O	0.82483	1.84595	-0.51115
C	0.96640	3.22004	-0.18328
H	4.97597	0.77525	1.16826
H	0.09194	-0.07153	-2.00200
H	2.56323	-2.30678	-0.59301
H	-4.08561	-2.10402	-0.51712
H	-2.95176	-2.40967	0.79985
H	-1.88324	-2.38852	-1.46321
H	-2.36175	-0.71330	-1.69539
H	-0.48354	-1.71638	0.51989
H	-0.94700	-0.03551	0.27580
H	0.56877	-1.75249	-1.76244
H	-4.02267	0.70217	2.52533
H	-3.21141	-0.95766	2.72805
H	1.82005	3.67331	-0.70407
H	1.08643	3.36686	0.89807
H	0.04490	3.70390	-0.51197
H	4.64413	-2.63320	0.30237
H	3.20060	2.43083	0.69777
Cl	-4.44707	0.79148	-0.29263

Conformer 17
Energy: -1116.22509 Hartree (Rel: 0.5 kcal/mol)
XYZ coordinates for conf 17:

C	-3.24751	1.49229	0.89799
C	-3.31206	0.09630	0.88550
C	-2.48603	-0.64509	0.03487
C	-1.57743	0.01172	-0.82281
C	-1.53372	1.40783	-0.79790
C	-2.35703	2.15370	0.05448
O	-2.32908	3.52583	0.09499
C	2.73704	-1.15627	0.20088

C	1.54770	-0.44858	-0.47676
C	0.53155	-1.44600	-1.04610
C	-0.67598	-0.78140	-1.74532
C	3.76105	-0.22295	0.78424
C	4.03136	-0.03843	2.07457
O	-2.49336	-2.01724	-0.03983
C	-3.41781	-2.72862	0.76966
H	-3.88811	2.06938	1.55766
H	-1.26399	-1.56864	-2.23127
H	-0.84299	1.92409	-1.46274
H	2.36871	-1.78927	1.01789
H	3.22668	-1.82133	-0.52350
H	1.06054	0.20915	0.25350
H	1.91976	0.19675	-1.28318
H	0.16349	-2.10132	-0.24714
H	1.03535	-2.09501	-1.77611
H	-0.30849	-0.12004	-2.54036
H	4.78311	0.66121	2.42243
H	3.48366	-0.60649	2.82127
H	-4.45429	-2.45163	0.53694
H	-3.23256	-2.55995	1.83856
H	-3.26689	-3.78521	0.54092
H	-1.66655	3.84655	-0.53762
H	-4.01413	-0.39970	1.54577
Cl	4.67468	0.71504	-0.42672

Conformer 18
Energy: -1116.22501 Hartree (Rel: 0.5 kcal/mol)
XYZ coordinates for conf 18:

C	3.25349	1.48555	0.87792
C	3.30962	0.08626	0.87887
C	2.47958	-0.65469	0.03556
C	1.57388	0.00259	-0.82913
C	1.53900	1.39635	-0.81770
C	2.36825	2.14451	0.02833
O	2.26099	3.51192	-0.03202
C	-2.74230	-1.14115	0.21292
C	-1.55462	-0.44211	-0.47670
C	-0.54178	-1.44779	-1.03747
C	0.66736	-0.79382	-1.74400
C	-3.76489	-0.20086	0.78756
C	-4.02981	0.00087	2.07648
O	2.47706	-2.02721	-0.02888
C	3.39522	-2.73852	0.78744
H	3.90592	2.05083	1.54014
H	0.30177	-0.13613	-2.54267
H	0.85903	1.92581	-1.47945
H	-3.23396	-1.81510	-0.50185
H	-2.37177	-1.76416	1.03663
H	-1.92826	0.19443	-1.28920
H	-1.06453	0.22378	0.24405
H	-1.04826	-2.10200	-1.76097
H	-0.17538	-2.09706	-0.23277
H	1.25093	-1.58739	-2.22513
H	-4.78049	0.70473	2.41805
H	-3.47831	-0.55647	2.82847
H	3.21009	-2.55922	1.85470
H	4.43394	-2.47084	0.55355
H	3.23701	-3.79592	0.56760
H	2.88790	3.90490	0.59595
H	4.00881	-0.40718	1.54398
Cl	-4.68452	0.71919	-0.43201

Conformer 19
Energy: -1116.22501 Hartree (Rel: 0.5 kcal/mol)
XYZ coordinates for conf 19:

C	-3.25339	1.48559	0.87799
C	-3.30954	0.08630	0.87892
C	-2.47955	-0.65467	0.03558
C	-1.57388	0.00261	-0.82915
C	-1.53897	1.39636	-0.81770

C	-2.36816	2.14453	0.02838
O	-2.26086	3.51194	-0.03195
C	2.74228	-1.14121	0.21280
C	1.55459	-0.44213	-0.47676
C	0.54175	-1.44779	-1.03756
C	-0.66741	-0.79382	-1.74405
C	3.76485	-0.20095	0.78752
C	4.02982	0.00058	2.07646
O	-2.47703	-2.02719	-0.02889
C	-3.39514	-2.73857	0.78743
H	-3.90576	2.05089	1.54024
H	-1.25101	-1.58739	-2.22514
H	-0.85901	1.92582	-1.47947
H	2.37177	-1.76431	1.03645
H	3.23395	-1.81506	-0.50205
H	1.06451	0.22373	0.24404
H	1.92821	0.19445	-1.28923
H	0.17536	-2.09708	-0.23286
H	1.04822	-2.10198	-1.76107
H	-0.30184	-0.13613	-2.54273
H	4.78048	0.70442	2.41812
H	3.47838	-0.55692	2.82838
H	-4.43388	-2.47091	0.55360
H	-3.20999	-2.55932	1.85469
H	-3.23690	-3.79595	0.56754
H	-2.88785	3.90493	0.59595
H	-4.00871	-0.40713	1.54407
Cl	4.68434	0.71935	-0.43195

Conformer 20
Energy: -1116.22473 Hartree (Rel: 0.7 kcal/mol)
XYZ coordinates for conf 20:

C	3.87241	-0.73306	-0.69516
C	3.47603	0.59748	-0.53552
C	2.30308	0.91062	0.15856
C	1.50623	-0.11739	0.70715
C	1.92433	-1.43999	0.53999
C	3.09690	-1.75785	-0.15647
O	3.52534	-3.05046	-0.33304
C	-2.63618	-0.10439	-1.25609
C	-1.45463	-0.51313	-0.35192
C	-0.95995	0.61197	0.56393
C	0.23350	0.20977	1.45984
C	-3.90040	0.26191	-0.52766
C	-4.49033	1.45490	-0.49304
O	1.85038	2.19180	0.36310
C	2.63332	3.26758	-0.13250
H	4.78311	-0.97776	-1.23303
H	-0.05323	-0.65800	2.06760
H	1.32133	-2.23947	0.96789
H	-2.34904	0.76205	-1.86521
H	-2.85525	-0.92612	-1.95049
H	-1.74466	-1.38374	0.25122
H	-0.63764	-0.84637	-1.00316
H	-1.78047	0.93986	1.21521
H	-0.67466	1.48229	-0.04015
H	0.43068	1.03426	2.15505
H	-5.40918	1.63954	0.05226
H	-4.04464	2.28651	-1.03133
H	2.72645	3.22967	-1.22579
H	3.63662	3.27531	0.31306
H	2.10551	4.17968	0.15207
H	2.89667	-3.65284	0.09563
H	4.09499	1.38067	-0.95781
Cl	-4.65167	-1.09098	0.35940

Conformer 21
Energy: -1116.22472 Hartree (Rel: 0.7 kcal/mol)
XYZ coordinates for conf 21:

C	3.86856	-0.73722	-0.68305
C	3.47376	0.59763	-0.53009

C	2.30138	0.91331	0.15930
C	1.50167	-0.11383	0.71205
C	1.91679	-1.43526	0.55215
C	3.09059	-1.75761	-0.14163
O	3.41504	-3.08760	-0.24753
C	-2.63829	-0.12319	-1.25514
C	-1.45879	-0.52260	-0.34420
C	-0.96452	0.61244	0.55956
C	0.22803	0.21913	1.46073
C	-3.90384	0.25220	-0.53350
C	-4.49285	1.44603	-0.51374
O	1.84810	2.19499	0.35804
C	2.63138	3.26840	-0.14152
H	4.78471	-0.96940	-1.22204
H	-0.05822	-0.64429	2.07452
H	1.32582	-2.24272	0.97573
H	-2.34956	0.73617	-1.87354
H	-2.85666	-0.95270	-1.94043
H	-1.75033	-1.38599	0.26832
H	-0.64107	-0.86462	-0.98987
H	-1.78541	0.94755	1.20670
H	-0.67876	1.47634	-0.05364
H	0.42369	1.04955	2.14926
H	-5.41271	1.63762	0.02745
H	-4.04524	2.27112	-1.06041
H	2.72638	3.22481	-1.23449
H	3.63406	3.27832	0.30556
H	2.10310	4.18190	0.13757
H	4.24525	-3.17044	-0.74318
H	4.09537	1.37726	-0.95491
Cl	-4.65816	-1.08977	0.36693

Conformer 22
Energy: -1116.22451 Hartree (Rel: 0.8 kcal/mol)
XYZ coordinates for conf 22:

C	-3.21186	-0.26683	-1.19034
C	-2.56998	0.95573	-0.95778
C	-1.68031	1.09494	0.10913
C	-1.42037	0.00080	0.96635
C	-2.07479	-1.20535	0.71943
C	-2.96562	-1.35126	-0.35203
O	-3.55966	-2.57856	-0.51609
C	3.02278	-1.20697	0.78029
C	1.51733	-1.18548	1.11797
C	1.03798	0.14182	1.71631
C	-0.45315	0.14168	2.12304
C	3.45289	-0.23048	-0.28017
C	4.26731	0.81166	-0.12725
O	-1.01671	2.25948	0.40982
C	-1.25052	3.39514	-0.40932
H	-3.90308	-0.36154	-2.02507
H	-0.66186	1.07829	2.65340
H	-1.89901	-2.05878	1.36863
H	3.60590	-0.98355	1.68275
H	3.30200	-2.21956	0.46068
H	0.93903	-1.42264	0.21746
H	1.32660	-1.99911	1.83110
H	1.21460	0.95874	1.00789
H	1.63574	0.36930	2.61067
H	-0.63056	-0.67487	2.83463
H	4.53783	1.46974	-0.94547
H	4.68732	1.02199	0.85225
H	-2.30574	3.69773	-0.38799
H	-0.95039	3.21118	-1.44913
H	-0.63728	4.19704	0.00585
H	-4.13969	-2.54575	-1.29358
H	-2.77771	1.78805	-1.62012
Cl	2.77866	-0.56153	-1.89692

Conformer 23
Energy: -1116.22449 Hartree (Rel: 0.8 kcal/mol)

XYZ coordinates for conf 23:

C	4.48532	0.09239	-0.23389
C	3.59427	1.16946	-0.15121
C	2.24075	0.94791	0.11022
C	1.75964	-0.36777	0.29848
C	2.66380	-1.42526	0.21399
C	4.02201	-1.20860	-0.05190
O	4.83617	-2.31248	-0.11547
C	-2.80300	0.13743	0.35431
C	-2.05321	-0.91180	-0.49172
C	-0.56933	-0.57982	-0.71639
C	0.29183	-0.62246	0.56442
C	-4.26954	-0.14816	0.52321
C	-4.89193	-0.49043	1.64920
O	1.30173	1.94625	0.20707
C	1.73359	3.28987	0.05316
H	5.53757	0.27915	-0.43766
H	0.17989	-1.60752	1.03540
H	2.31876	-2.44543	0.35985
H	-2.36948	0.19379	1.35920
H	-2.67794	1.12745	-0.10502
H	-2.14641	-1.89357	-0.00743
H	-2.54619	-0.99695	-1.46753
H	-0.47905	0.41247	-1.17621
H	-0.15687	-1.29739	-1.43741
H	-0.07804	0.12050	1.27976
H	-5.95685	-0.68933	1.69382
H	-4.32145	-0.57699	2.56973
H	2.16023	3.46518	-0.94312
H	2.47503	3.56513	0.81466
H	0.84288	3.90853	0.17709
H	5.74397	-2.02389	-0.30128
H	3.97549	2.17394	-0.29407
Cl	-5.22529	-0.00482	-0.97552

Conformer 24

Energy: -1116.22449 Hartree (Rel: 0.8 kcal/mol)

XYZ coordinates for conf 24:

C	-4.48533	0.09257	-0.23376
C	-3.59423	1.16959	-0.15076
C	-2.24072	0.94791	0.11059
C	-1.75969	-0.36785	0.29843
C	-2.66389	-1.42529	0.21367
C	-4.02210	-1.20849	-0.05212
O	-4.83633	-2.31231	-0.11594
C	2.80300	0.13715	0.35449
C	2.05320	-0.91175	-0.49193
C	0.56931	-0.57966	-0.71649
C	-0.29188	-0.62272	0.56427
C	4.26952	-0.14851	0.52321
C	4.89194	-0.49171	1.64890
O	-1.30167	1.94620	0.20786
C	-1.73328	3.28981	0.05315
H	-5.53756	0.27948	-0.43748
H	0.07803	0.11993	1.27992
H	-2.31886	-2.44552	0.35920
H	2.67791	1.12735	-0.10447
H	2.36949	0.19317	1.35941
H	2.54617	-0.99656	-1.46778
H	2.14638	-1.89369	-0.00800
H	0.15686	-1.29697	-1.43778
H	0.47907	0.41278	-1.17599
H	-0.18001	-1.60796	1.03490
H	5.95686	-0.69068	1.69328
H	4.32154	-0.57897	2.56942
H	-2.47468	3.56567	0.81447
H	-2.15983	3.46462	-0.94325
H	-0.84247	3.90840	0.17677
H	-5.74405	-2.02363	-0.30200
H	-3.97545	2.17412	-0.29331
Cl	5.22526	-0.00405	-0.97544

Conformer 25
Energy: -1116.22447 Hartree (Rel: 0.9 kcal/mol)
XYZ coordinates for conf 25:

C	-4.40661	-0.58449	-0.29402
C	-3.88004	0.70935	-0.19683
C	-2.53161	0.90199	0.10977
C	-1.68720	-0.21016	0.32979
C	-2.23246	-1.48921	0.23076
C	-3.58366	-1.68789	-0.08098
O	-4.03014	-2.98420	-0.15670
C	2.88172	-0.62669	0.25445
C	2.10989	0.58340	-0.31028
C	0.63006	0.29252	-0.60627
C	-0.21999	-0.01529	0.64537
C	4.34655	-0.36847	0.47490
C	4.97659	-0.29141	1.64514
O	-1.93651	2.13498	0.22304
C	-2.74334	3.28819	0.03672
H	-5.45892	-0.72040	-0.53415
H	-0.10376	0.81008	1.35862
H	-1.60448	-2.35986	0.40044
H	2.46039	-0.92751	1.22047
H	2.76441	-1.48019	-0.42738
H	2.18913	1.41921	0.39815
H	2.59585	0.91179	-1.23685
H	0.55384	-0.54813	-1.31004
H	0.19869	1.16403	-1.11193
H	0.15459	-0.92071	1.13681
H	6.03943	-0.09341	1.72731
H	4.41491	-0.43131	2.56449
H	-3.55849	3.33302	0.77080
H	-3.16874	3.32409	-0.97476
H	-2.08188	4.14462	0.17962
H	-4.97559	-2.97901	-0.37590
H	-4.53796	1.55428	-0.36397
Cl	5.28779	-0.14223	-1.02256

Conformer 26
Energy: -1116.22447 Hartree (Rel: 0.9 kcal/mol)
XYZ coordinates for conf 26:

C	4.40673	-0.58439	-0.29384
C	3.88002	0.70942	-0.19711
C	2.53154	0.90204	0.10931
C	1.68725	-0.21013	0.32961
C	2.23263	-1.48916	0.23103
C	3.58388	-1.68782	-0.08052
O	4.03047	-2.98413	-0.15578
C	-2.88184	-0.62641	0.25502
C	-2.10990	0.58306	-0.31089
C	-0.63010	0.29177	-0.60676
C	0.21998	-0.01534	0.64501
C	-4.34664	-0.36782	0.47522
C	-4.97662	-0.28928	1.64539
O	1.93631	2.13504	0.22198
C	2.74335	3.28831	0.03682
H	5.45908	-0.72023	-0.53386
H	-0.15447	-0.92061	1.13685
H	1.60470	-2.35980	0.40093
H	-2.76459	-1.48060	-0.42597
H	-2.46056	-0.92634	1.22134
H	-2.59591	0.91068	-1.23770
H	-2.18896	1.41952	0.39681
H	-0.19869	1.16292	-1.11301
H	-0.55404	-0.54933	-1.31001
H	0.10361	0.81033	1.35791
H	-6.03945	-0.09111	1.72735
H	-4.41493	-0.42816	2.56488
H	3.16908	3.32503	-0.97449
H	3.55827	3.33239	0.77118
H	2.08200	4.14475	0.18024

H	4.97594	-2.97896	-0.37492
H	4.53785	1.55436	-0.36457
Cl	-5.28793	-0.14339	-1.02249

Conformer 27
Energy: -1116.22442 Hartree (Rel: 0.9 kcal/mol)
XYZ coordinates for conf 27:

C	4.49020	0.09918	-0.23749
C	3.59936	1.17275	-0.15507
C	2.24365	0.95154	0.10755
C	1.76337	-0.36153	0.29674
C	2.67071	-1.41993	0.21202
C	4.02788	-1.20284	-0.05446
O	4.93749	-2.22784	-0.14091
C	-2.79964	0.14395	0.35050
C	-2.04996	-0.91072	-0.48884
C	-0.56618	-0.58000	-0.71612
C	0.29621	-0.61794	0.56403
C	-4.26547	-0.14237	0.52412
C	-4.88568	-0.47531	1.65412
O	1.30518	1.95076	0.20330
C	1.73767	3.29447	0.05025
H	5.54293	0.26897	-0.44116
H	0.18477	-1.60156	1.03860
H	2.30922	-2.43648	0.36107
H	-2.36444	0.20882	1.35416
H	-2.67665	1.13048	-0.11678
H	-2.14303	-1.88938	0.00185
H	-2.54327	-1.00221	-1.46389
H	-0.47585	0.41059	-1.17944
H	-0.15456	-1.30001	-1.43530
H	-0.07400	0.12670	1.27737
H	-5.95027	-0.67518	1.70208
H	-4.31387	-0.55237	2.57468
H	2.16406	3.47057	-0.94593
H	2.47905	3.56919	0.81191
H	0.84702	3.91316	0.17468
H	4.47785	-3.06992	0.00585
H	3.97848	2.17798	-0.29877
Cl	-5.22362	-0.01412	-0.97437

Conformer 28
Energy: -1116.22442 Hartree (Rel: 0.9 kcal/mol)
XYZ coordinates for conf 28:

C	-4.49024	0.09934	-0.23713
C	-3.59940	1.17287	-0.15422
C	-2.24368	0.95153	0.10822
C	-1.76340	-0.36164	0.29680
C	-2.67076	-1.41999	0.21167
C	-4.02794	-1.20276	-0.05469
O	-4.93756	-2.22772	-0.14158
C	2.79964	0.14375	0.35071
C	2.04990	-0.91042	-0.48920
C	0.56612	-0.57954	-0.71624
C	-0.29624	-0.61820	0.56391
C	4.26546	-0.14271	0.52414
C	4.88570	-0.47625	1.65395
O	-1.30522	1.95070	0.20460
C	-1.73723	3.29431	0.04937
H	-5.54298	0.26924	-0.44069
H	0.07401	0.12601	1.27768
H	-2.30927	-2.43661	0.36023
H	2.67667	1.13055	-0.11601
H	2.36447	0.20808	1.35441
H	2.54317	-1.00137	-1.46431
H	2.14296	-1.88936	0.00094
H	0.15447	-1.29911	-1.43582
H	0.47580	0.41132	-1.17898
H	-0.18479	-1.60209	1.03791
H	5.95029	-0.67617	1.70177
H	4.31392	-0.55378	2.57448

```
H          -2.47847        3.57058        0.81061
H          -2.16359        3.46890       -0.94708
H          -0.84635        3.91287        0.17280
H          -4.47791       -3.06988        0.00469
H          -3.97854        2.17817       -0.29736
Cl          5.22358       -0.01370       -0.97431

Conformer 29
Energy: -1116.22439 Hartree (Rel:  0.9 kcal/mol)
XYZ coordinates for conf 29:
C           4.40743       -0.51746        0.04655
C           3.83381        0.75843        0.11011
C           2.45634        0.92246       -0.04838
C           1.62917       -0.20038       -0.27942
C           2.22103       -1.46086       -0.34166
C           3.60198       -1.63127       -0.17923
O           4.09284       -2.91143       -0.25716
C          -2.89083       -0.83503        0.24398
C          -2.10987        0.37866        0.78835
C          -0.59707        0.13612        0.91448
C           0.13250       -0.03839       -0.43510
C          -4.37981       -0.63263        0.19777
C          -5.29355       -1.24769        0.94546
O           1.81488        2.13621        0.00067
C           2.60044        3.30258        0.19561
H           5.48220       -0.63068        0.17176
H          -0.07995        0.83577       -1.06275
H           1.60679       -2.33873       -0.52391
H          -2.53660       -1.08999       -0.76262
H          -2.69893       -1.70680        0.88251
H          -2.28871        1.24794        0.14278
H          -2.51369        0.63431        1.77645
H          -0.41470       -0.74843        1.54071
H          -0.15132        0.98835        1.44010
H          -0.26063       -0.91553       -0.96191
H          -6.35505       -1.04392        0.86007
H          -4.97678       -1.98807        1.67465
H           3.33479        3.43336       -0.60996
H           3.12524        3.27963        1.15968
H           1.90173        4.14112        0.18617
H           5.05541       -2.88788       -0.13545
H           4.47857        1.61202        0.28447
Cl         -4.91997        0.57483       -0.99777

Conformer 30
Energy: -1116.22439 Hartree (Rel:  0.9 kcal/mol)
XYZ coordinates for conf 30:
C          -4.40747       -0.51740        0.04670
C          -3.83380        0.75845        0.11056
C          -2.45633        0.92246       -0.04797
C          -1.62921       -0.20036       -0.27930
C          -2.22112       -1.46080       -0.34178
C          -3.60207       -1.63120       -0.17936
O          -4.09299       -2.91132       -0.25758
C           2.89083       -0.83510        0.24339
C           2.10991        0.37828        0.78844
C           0.59712        0.13565        0.91458
C          -0.13254       -0.03839       -0.43501
C           4.37984       -0.63281        0.19738
C           5.29347       -1.24876        0.94449
O          -1.81481        2.13617        0.00148
C          -2.60053        3.30271        0.19482
H          -5.48223       -0.63061        0.17192
H           0.26052       -0.91536       -0.96214
H          -1.60691       -2.33865       -0.52427
H           2.69884       -1.70726        0.88136
H           2.53665       -1.08942       -0.76339
H           2.51378        0.63347        1.77664
H           2.28866        1.24790        0.14330
H           0.15139        0.98768        1.44054
H           0.41481       -0.74912        1.54051
```

H	0.07989	0.83598	-1.06238
H	6.35501	-1.04510	0.85928
H	4.97658	-1.98977	1.67299
H	-3.12567	3.28083	1.15874
H	-3.33462	3.43248	-0.61115
H	-1.90189	4.14129	0.18468
H	-5.05554	-2.88775	-0.13578
H	-4.47851	1.61201	0.28521
Cl	4.92018	0.57566	-0.99699

Conformer 31
Energy: -1116.22438 Hartree (Rel: 0.9 kcal/mol)
XYZ coordinates for conf 31:

C	-3.21642	-0.21361	-1.21218
C	-2.56055	0.99173	-0.94786
C	-1.67101	1.09542	0.12616
C	-1.42590	-0.02016	0.95543
C	-2.09621	-1.21359	0.67588
C	-2.98690	-1.32169	-0.39913
O	-3.65649	-2.48475	-0.69085
C	3.01381	-1.24023	0.75015
C	1.50699	-1.22410	1.08214
C	1.03048	0.08515	1.72098
C	-0.46267	0.07977	2.11968
C	3.45120	-0.23144	-0.27665
C	4.26813	0.80258	-0.08656
O	-0.99614	2.24549	0.45675
C	-1.21648	3.40407	-0.33383
H	-3.90740	-0.29573	-2.04546
H	-0.66935	1.00087	2.67744
H	-1.92016	-2.07724	1.31544
H	3.59370	-1.04792	1.66178
H	3.29118	-2.24291	0.39922
H	0.93184	-1.42901	0.17166
H	1.31028	-2.05975	1.76786
H	1.21447	0.92351	1.04023
H	1.62452	0.28056	2.62534
H	-0.64730	-0.75665	2.80622
H	4.54399	1.48608	-0.88183
H	4.68464	0.97969	0.90096
H	-2.26869	3.71642	-0.30794
H	-0.91451	3.24417	-1.37701
H	-0.59688	4.18892	0.10389
H	-3.40213	-3.16498	-0.04711
H	-2.75475	1.84335	-1.58958
Cl	2.78240	-0.50816	-1.90550

Conformer 32
Energy: -1116.22437 Hartree (Rel: 0.9 kcal/mol)
XYZ coordinates for conf 32:

C	-4.46320	-0.03138	0.04375
C	-3.62829	1.08751	-0.06308
C	-2.24688	0.92814	-0.18772
C	-1.68061	-0.36671	-0.21385
C	-2.52997	-1.46693	-0.10695
C	-3.91609	-1.31213	0.02436
O	-4.67312	-2.45341	0.12435
C	2.82776	0.45910	0.09904
C	2.07203	-0.59352	0.93584
C	0.55540	-0.35220	1.00842
C	-0.18331	-0.55169	-0.33260
C	4.32017	0.27816	0.09425
C	5.22344	1.07637	0.65930
O	-1.35843	1.97089	-0.29493
C	-1.87537	3.29303	-0.30348
H	-5.53800	0.10634	0.13827
H	0.02393	-1.56275	-0.70562
H	-2.11827	-2.47249	-0.12845
H	2.46538	0.44786	-0.93636
H	2.62053	1.45918	0.50034
H	2.26667	-1.59354	0.52746

H 2.48332 -0.58600 1.95345
H 0.35868 0.66189 1.37890
H 0.12803 -1.04291 1.74682
H 0.20787 0.15174 -1.07584
H 6.28852 0.87710 0.62049
H 4.89368 1.96897 1.18355
H -2.40062 3.52732 0.63177
H -2.55781 3.45409 -1.14826
H -1.01227 3.95329 -0.40660
H -5.60681 -2.20456 0.21677
H -4.07476 2.07496 -0.04676
Cl 4.88082 -1.17922 -0.76641

Conformer 33
Energy: -1116.22437 Hartree (Rel: 0.9 kcal/mol)
XYZ coordinates for conf 33:
C 4.46316 -0.03152 0.04373
C 3.62836 1.08745 -0.06334
C 2.24694 0.92821 -0.18800
C 1.68058 -0.36659 -0.21401
C 2.52981 -1.46690 -0.10690
C 3.91593 -1.31221 0.02450
O 4.67285 -2.45354 0.12480
C -2.82779 0.45917 0.09949
C -2.07197 -0.59375 0.93584
C -0.55533 -0.35247 1.00837
C 0.18326 -0.55151 -0.33278
C -4.32014 0.27814 0.09465
C -5.22361 1.07574 0.66024
O 1.35857 1.97100 -0.29542
C 1.87555 3.29311 -0.30317
H 5.53796 0.10617 0.13828
H -0.20797 0.15215 -1.07578
H 2.11802 -2.47243 -0.12831
H -2.62057 1.45908 0.50122
H -2.46542 0.44844 -0.93592
H -2.48321 -0.58660 1.95347
H -2.26662 -1.59363 0.52713
H -0.12787 -1.04341 1.74649
H -0.35857 0.66151 1.37917
H -0.02403 -1.56244 -0.70614
H -6.28864 0.87620 0.62122
H -4.89415 1.96805 1.18515
H 2.55798 3.45469 -1.14786
H 2.40072 3.52685 0.63226
H 1.01249 3.95349 -0.40595
H 5.60661 -2.20479 0.21675
H 4.07497 2.07484 -0.04718
Cl -4.88068 -1.17877 -0.76710

Conformer 34
Energy: -1116.22436 Hartree (Rel: 0.9 kcal/mol)
XYZ coordinates for conf 34:
C 4.46920 -0.04301 0.04887
C 3.64264 1.07875 -0.05653
C 2.25784 0.93049 -0.18324
C 1.68230 -0.35742 -0.21007
C 2.52648 -1.46531 -0.10526
C 3.91299 -1.32071 0.02498
O 4.76278 -2.39435 0.12770
C -2.82894 0.46708 0.08477
C -2.07083 -0.56781 0.94121
C -0.55488 -0.32175 1.01069
C 0.18401 -0.53342 -0.32848
C -4.32077 0.28179 0.08290
C -5.22702 1.08827 0.63112
O 1.37832 1.98096 -0.29129
C 1.90640 3.29878 -0.30744
H 5.54444 0.06996 0.14680
H -0.20211 0.16861 -1.07569
H 2.09052 -2.46318 -0.13076

H	-2.62454	1.47506	0.46725
H	-2.46629	0.43742	-0.95017
H	-2.48282	-0.54300	1.95826
H	-2.26265	-1.57560	0.55088
H	-0.12594	-1.00339	1.75665
H	-0.36001	0.69660	1.36998
H	-0.03049	-1.54490	-0.69685
H	-6.29143	0.88496	0.59524
H	-4.90054	1.99216	1.13776
H	2.58959	3.44950	-1.15347
H	2.43370	3.53442	0.62623
H	1.04863	3.96548	-0.41403
H	4.24247	-3.21281	0.09338
H	4.09452	2.06379	-0.03813
Cl	-4.87661	-1.19426	-0.74916

Conformer 35
Energy: -1116.22436 Hartree (Rel: 0.9 kcal/mol)
XYZ coordinates for conf 35:

C	-4.46916	-0.04307	0.04924
C	-3.64263	1.07875	-0.05563
C	-2.25782	0.93057	-0.18245
C	-1.68225	-0.35732	-0.20982
C	-2.52640	-1.46527	-0.10545
C	-3.91292	-1.32075	0.02482
O	-4.76269	-2.39445	0.12709
C	2.82901	0.46716	0.08532
C	2.07091	-0.56817	0.94124
C	0.55497	-0.32215	1.01091
C	-0.18396	-0.53323	-0.32833
C	4.32082	0.28173	0.08318
C	5.22720	1.08765	0.63199
O	-1.37832	1.98113	-0.28975
C	-1.90676	3.29876	-0.30945
H	-5.54441	0.06983	0.14721
H	0.03055	-1.54455	-0.69715
H	-2.09043	-2.46312	-0.13137
H	2.46623	0.43814	-0.94960
H	2.62472	1.47494	0.46842
H	2.26272	-1.57575	0.55038
H	2.48295	-0.54388	1.95829
H	0.36011	0.69605	1.37066
H	0.12607	-1.00412	1.75658
H	0.20211	0.16915	-1.07523
H	6.29158	0.88423	0.59589
H	4.90087	1.99120	1.13935
H	-2.43430	3.53675	0.62349
H	-2.58982	3.44703	-1.15601
H	-1.04915	3.96543	-0.41757
H	-4.24238	-3.21289	0.09224
H	-4.09451	2.06378	-0.03669
Cl	4.87643	-1.19375	-0.75006

Conformer 36
Energy: -1116.22436 Hartree (Rel: 0.9 kcal/mol)
XYZ coordinates for conf 36:

C	-4.41414	-0.58171	-0.29391
C	-3.88595	0.70846	-0.19774
C	-2.53477	0.90143	0.10700
C	-1.69190	-0.20882	0.32606
C	-2.24058	-1.48947	0.22745
C	-3.59162	-1.68671	-0.08221
O	-4.15217	-2.93608	-0.18412
C	2.87837	-0.61765	0.26379
C	2.10488	0.58413	-0.31626
C	0.62630	0.28699	-0.61198
C	-0.22428	-0.01604	0.64050
C	4.34306	-0.35474	0.47952
C	4.97333	-0.25755	1.64812
O	-1.93964	2.13484	0.21797
C	-2.74653	3.28835	0.03233

H	-5.46269	-0.73340	-0.53071
H	-0.10693	0.81131	1.35120
H	-1.59818	-2.35225	0.39917
H	2.45807	-0.90582	1.23415
H	2.76162	-1.48029	-0.40659
H	2.18080	1.42785	0.38304
H	2.59193	0.90335	-1.24544
H	0.55329	-0.55771	-1.31133
H	0.19277	1.15430	-1.12288
H	0.15003	-0.92005	1.13519
H	6.03593	-0.05685	1.72665
H	4.41204	-0.38249	2.56986
H	-3.56019	3.33435	0.76789
H	-3.17367	3.32403	-0.97837
H	-2.08428	4.14450	0.17347
H	-3.46755	-3.60288	-0.01539
H	-4.54209	1.55506	-0.36383
Cl	5.28333	-0.15254	-1.02193

Conformer 37
Energy: -1116.22429 Hartree (Rel: 1.0 kcal/mol)
XYZ coordinates for conf 37:

C	3.52738	0.27037	-1.25746
C	2.62772	1.27651	-0.88881
C	1.73074	1.07517	0.16407
C	1.72924	-0.14975	0.86872
C	2.63898	-1.13668	0.48780
C	3.53529	-0.94129	-0.56909
O	4.39199	-1.97105	-0.87177
C	-2.64108	-1.39193	0.26560
C	-1.41157	-0.52887	0.60957
C	-0.48322	-1.22836	1.60999
C	0.76380	-0.40718	2.00881
C	-3.58306	-0.75977	-0.72088
C	-3.81645	-1.15618	-1.97015
O	0.82025	2.01467	0.58230
C	0.79318	3.26768	-0.08390
H	4.21965	0.44109	-2.07924
H	1.29335	-0.95786	2.79644
H	2.66353	-2.08344	1.02142
H	-2.31081	-2.34614	-0.16397
H	-3.19039	-1.62886	1.18700
H	-0.86493	-0.30268	-0.31389
H	-1.73950	0.43218	1.02274
H	-0.15792	-2.19278	1.19467
H	-1.04746	-1.45957	2.52453
H	0.44436	0.54484	2.44369
H	-4.50794	-0.64308	-2.62911
H	-3.29969	-2.02934	-2.35846
H	0.54510	3.15460	-1.14735
H	1.75233	3.79436	0.00706
H	0.01304	3.85218	0.40708
H	4.95939	-1.70037	-1.61119
H	2.63988	2.21229	-1.43535
Cl	-4.44562	0.67207	-0.09977

Conformer 38
Energy: -1116.22427 Hartree (Rel: 1.0 kcal/mol)
XYZ coordinates for conf 38:

C	-4.41401	-0.51340	0.05021
C	-3.83858	0.75865	0.11059
C	-2.45895	0.92190	-0.04967
C	-1.63431	-0.19980	-0.27990
C	-2.23023	-1.46157	-0.34004
C	-3.61032	-1.62955	-0.17460
O	-4.21716	-2.86024	-0.22817
C	2.88853	-0.83199	0.24823
C	2.10459	0.38234	0.78678
C	0.59218	0.13759	0.91303
C	-0.13746	-0.04077	-0.43610
C	4.37702	-0.62574	0.20089

C	5.29208	-1.23289	0.95343
O	-1.81711	2.13571	-0.00166
C	-2.60188	3.30293	0.19291
H	-5.48472	-0.64201	0.17504
H	0.25575	-0.91939	-0.96079
H	-1.60211	-2.33230	-0.52397
H	2.69903	-1.70101	0.89130
H	2.53488	-1.09274	-0.75712
H	2.50722	0.64291	1.77405
H	2.28212	1.24928	0.13776
H	0.14493	0.99039	1.43625
H	0.41104	-0.74551	1.54178
H	0.07579	0.83136	-1.06626
H	6.35301	-1.02642	0.86736
H	4.97716	-1.96896	1.68777
H	-3.12576	3.28142	1.15746
H	-3.33671	3.43381	-0.61212
H	-1.90234	4.14077	0.18214
H	-3.54066	-3.53796	-0.38578
H	-4.48069	1.61440	0.28453
Cl	4.91377	0.57483	-1.00267

Conformer 39
Energy: -1116.22427 Hartree (Rel: 1.0 kcal/mol)
XYZ coordinates for conf 39:

C	4.41395	-0.51339	0.04999
C	3.83859	0.75869	0.11021
C	2.45894	0.92198	-0.05001
C	1.63424	-0.19970	-0.28008
C	2.23011	-1.46152	-0.34006
C	3.61017	-1.62953	-0.17460
O	4.21695	-2.86025	-0.22789
C	-2.88845	-0.83219	0.24787
C	-2.10452	0.38180	0.78721
C	-0.59213	0.13696	0.91312
C	0.13739	-0.04063	-0.43621
C	-4.37693	-0.62592	0.20058
C	-5.29211	-1.23371	0.95245
O	1.81718	2.13584	-0.00215
C	2.60182	3.30292	0.19376
H	5.48466	-0.64207	0.17476
H	-0.07593	0.83185	-1.06585
H	1.60196	-2.33224	-0.52388
H	-2.53477	-1.09228	-0.75764
H	-2.69898	-1.70161	0.89039
H	-2.28213	1.24916	0.13877
H	-2.50705	0.64172	1.77468
H	-0.41093	-0.74653	1.54131
H	-0.14476	0.98942	1.43681
H	-0.25582	-0.91898	-0.96133
H	-6.35303	-1.02719	0.86641
H	-4.97726	-1.97036	1.68623
H	3.33681	3.43470	-0.61100
H	3.12553	3.28045	1.15838
H	1.90224	4.14073	0.18371
H	3.54049	-3.53793	-0.38584
H	4.48073	1.61447	0.28389
Cl	-4.91360	0.57573	-1.00205

Conformer 40
Energy: -1116.22421 Hartree (Rel: 1.0 kcal/mol)
XYZ coordinates for conf 40:

C	3.33965	0.29705	1.30268
C	2.53204	1.29918	0.75419
C	1.70419	1.02450	-0.33796
C	1.67968	-0.27182	-0.89976
C	2.49788	-1.25404	-0.34063
C	3.32512	-0.98453	0.75550
O	4.09509	-2.01480	1.23655
C	-2.80892	-1.16699	-0.54402
C	-1.49565	-0.42908	-0.86897

C	-0.53966	-1.30021	-1.69315
C	0.78769	-0.60852	-2.07826
C	-3.79916	-0.35159	0.23997
C	-4.97487	0.10388	-0.18752
O	0.88635	1.95607	-0.93002
C	0.88560	3.27656	-0.40951
H	3.97867	0.52503	2.15309
H	0.56837	0.30073	-2.64653
H	2.50413	-2.25603	-0.76218
H	-3.30208	-1.46893	-1.47670
H	-2.58123	-2.08785	0.01003
H	-1.72592	0.49227	-1.41865
H	-1.00819	-0.12387	0.06370
H	-1.04218	-1.61353	-2.61924
H	-0.31488	-2.22084	-1.13617
H	1.33655	-1.28115	-2.74933
H	-5.63691	0.69488	0.43543
H	-5.29776	-0.11949	-1.20052
H	1.87907	3.73886	-0.47953
H	0.55202	3.29850	0.63614
H	0.18159	3.84124	-1.02350
H	4.62360	-1.69006	1.98299
H	2.56062	2.28970	1.19301
Cl	-3.28992	0.02025	1.90766

Conformer 41
Energy: -1116.22418 Hartree (Rel: 1.0 kcal/mol)
XYZ coordinates for conf 41:

C	-4.04964	0.61246	-0.47892
C	-2.94306	1.45123	-0.29790
C	-1.72180	0.92712	0.13037
C	-1.59407	-0.45658	0.38915
C	-2.70872	-1.27317	0.20496
C	-3.93506	-0.75291	-0.22826
O	-4.97636	-1.63504	-0.38074
C	2.95659	-1.05709	0.97836
C	2.03747	-1.92848	0.09398
C	0.71673	-1.26624	-0.32932
C	-0.27176	-1.04150	0.83564
C	3.47939	0.18574	0.31155
C	3.19295	1.44701	0.62730
O	-0.59044	1.67968	0.33337
C	-0.65985	3.07507	0.08247
H	-4.99548	1.03387	-0.81271
H	0.18222	-0.37741	1.57955
H	-2.63657	-2.33958	0.40185
H	2.42541	-0.74238	1.88405
H	3.80859	-1.66612	1.30714
H	2.59388	-2.22748	-0.80265
H	1.82240	-2.85374	0.64553
H	0.23133	-1.90476	-1.07865
H	0.92000	-0.30697	-0.81974
H	-0.45750	-2.00195	1.33344
H	3.61713	2.29388	0.09936
H	2.50495	1.64969	1.44286
H	-1.39217	3.56717	0.73598
H	-0.91526	3.28430	-0.96451
H	0.33568	3.46871	0.29519
H	-5.75934	-1.14807	-0.68357
H	-3.05399	2.51100	-0.49618
Cl	4.60635	-0.12549	-1.03554

Conformer 42
Energy: -1116.22416 Hartree (Rel: 1.0 kcal/mol)
XYZ coordinates for conf 42:

C	3.80983	1.12487	-0.48984
C	3.62156	-0.26189	-0.44161
C	2.41618	-0.79549	0.01862
C	1.37685	0.06205	0.44598
C	1.58584	1.43909	0.39118
C	2.79118	1.97949	-0.07493

```
O          2.90443        3.34791       -0.09285
C         -3.13884       -0.68298        1.04544
C         -2.18240       -1.58809        0.23824
C         -0.87630       -0.92336       -0.22292
C          0.05955       -0.50322        0.93076
C         -3.62291        0.53897        0.31408
C         -3.38184        1.80972        0.63044
O          2.14966       -2.14112        0.09600
C          3.16761       -3.04948       -0.29648
H          4.75404        1.52785       -0.84998
H          0.24647       -1.37647        1.56780
H          0.80233        2.11848        0.71623
H         -4.00801       -1.27917        1.35291
H         -2.65180       -0.33894        1.96523
H         -1.94763       -2.46165        0.86113
H         -2.71745       -1.97032       -0.63955
H         -1.10282       -0.04435       -0.84033
H         -0.33769       -1.62806       -0.86695
H         -0.43483        0.25032        1.55477
H         -3.77114        2.64178        0.05442
H         -2.77495        2.03771        1.50218
H          4.06966       -2.93493        0.31884
H          3.43583       -2.92333       -1.35360
H          2.75377       -4.04842       -0.14702
H          3.77933        3.58649       -0.43859
H          4.42718       -0.90935       -0.76794
Cl        -4.63017        0.18824       -1.11462
```

Conformer 43
Energy: -1116.22416 Hartree (Rel: 1.0 kcal/mol)
XYZ coordinates for conf 43:

```
C         -3.80984        1.12492       -0.48981
C         -3.62154       -0.26185       -0.44173
C         -2.41617       -0.79549        0.01848
C         -1.37686        0.06203        0.44587
C         -1.58581        1.43910        0.39113
C         -2.79119        1.97951       -0.07490
O         -2.90455        3.34792       -0.09252
C          3.13878       -0.68315        1.04530
C          2.18234       -1.58811        0.23789
C          0.87623       -0.92337       -0.22316
C         -0.05952       -0.50321        0.93059
C          3.62286        0.53890        0.31424
C          3.38178        1.80965        0.63058
O         -2.14961       -2.14110        0.09578
C         -3.16782       -3.04944       -0.29600
H         -4.75410        1.52787       -0.84987
H          0.43488        0.25033        1.55458
H         -0.80227        2.11847        0.71611
H          2.65173       -0.33939        1.96520
H          4.00793       -1.27943        1.35266
H          2.71741       -1.97020       -0.63995
H          1.94758       -2.46175        0.86065
H          0.33757       -1.62810       -0.86713
H          1.10268       -0.04438       -0.84061
H         -0.24639       -1.37644        1.56768
H          3.77119        2.64167        0.05460
H          2.77477        2.03770        1.50223
H         -3.43629       -2.92374       -1.35312
H         -4.06966       -2.93450        0.31953
H         -2.75413       -4.04841       -0.14624
H         -3.77866        3.58653       -0.44023
H         -4.42716       -0.90926       -0.76816
Cl         4.63041        0.18833       -1.11440
```

Conformer 44
Energy: -1116.22409 Hartree (Rel: 1.1 kcal/mol)
XYZ coordinates for conf 44:

```
C         -4.05099        0.62874       -0.48562
C         -2.94357        1.46085       -0.30146
C         -1.72225        0.93245        0.12877
```

```
C          -1.59866       -0.44947        0.38508
C          -2.71846       -1.26294        0.19704
C          -3.94182       -0.73822       -0.23676
O          -5.05591       -1.51815       -0.42726
C           2.94961       -1.05262        0.98246
C           2.03166       -1.92750        0.10025
C           0.71205       -1.26605       -0.32795
C          -0.27975       -1.03993        0.83395
C           3.47654        0.18532        0.30979
C           3.18949        1.44886        0.61569
O          -0.58969        1.68279        0.33466
C          -0.65548        3.07889        0.08559
H          -4.99989        1.03719       -0.81927
H           0.17397       -0.37825        1.58013
H          -2.63264       -2.33016        0.39731
H           2.41618       -0.73167        1.88463
H           3.79952       -1.66115        1.31743
H           2.58953       -2.23043       -0.79414
H           1.81528       -2.85041        0.65529
H           0.22898       -1.90508       -1.07843
H           0.91658       -0.30722       -0.81855
H          -0.46897       -2.00053        1.33059
H           3.61669        2.29186        0.08402
H           2.49750        1.65748        1.42637
H          -1.38763        3.57196        0.73848
H          -0.90825        3.29037       -0.96152
H           0.34066        3.46965        0.30076
H          -4.83513       -2.43983       -0.21869
H          -3.04922        2.52157       -0.49787
Cl          4.60950       -0.13604       -1.02980

Conformer 45
Energy: -1116.22409 Hartree (Rel:  1.1 kcal/mol)
XYZ coordinates for conf 45:
C           4.05108        0.62847       -0.48592
C           2.94379        1.46071       -0.30154
C           1.72249        0.93244        0.12892
C           1.59879       -0.44946        0.38523
C           2.71846       -1.26306        0.19698
C           3.94179       -0.73849       -0.23705
O           5.05577       -1.51853       -0.42774
C          -2.94956       -1.05171        0.98277
C          -2.03171       -1.92716        0.10101
C          -0.71201       -1.26608       -0.32747
C           0.27989       -1.03979        0.83432
C          -3.47665        0.18569        0.30923
C          -3.18915        1.44947        0.61372
O           0.59005        1.68293        0.33502
C           0.65597        3.07902        0.08593
H           4.99996        1.03682       -0.81974
H           0.46911       -2.00033        1.33109
H           2.63254       -2.33027        0.39727
H          -3.79938       -1.66005        1.31831
H          -2.41600       -0.73009        1.88461
H          -1.81545       -2.84982        0.65652
H          -2.58960       -2.23046       -0.79324
H          -0.91640       -0.30737       -0.81834
H          -0.22909       -1.90542       -1.07778
H          -0.17374       -0.37799        1.58044
H          -3.61642        2.29204        0.08143
H          -2.49668        1.65872        1.42383
H           0.90857        3.29047       -0.96122
H           1.38830        3.57199        0.73869
H          -0.34007        3.46992        0.30129
H           4.83490       -2.44021       -0.21928
H           3.04954        2.52141       -0.49797
Cl         -4.61034       -0.13675       -1.02947

Conformer 46
Energy: -1116.22404 Hartree (Rel:  1.1 kcal/mol)
XYZ coordinates for conf 46:
```

C	3.81575	1.12919	-0.49445
C	3.62803	-0.25444	-0.43825
C	2.42014	-0.78874	0.02136
C	1.37976	0.06775	0.43982
C	1.58915	1.44728	0.37805
C	2.79471	1.98597	-0.08732
O	3.02255	3.33835	-0.15770
C	-3.13386	-0.70622	1.03956
C	-2.17162	-1.59755	0.22413
C	-0.87034	-0.92090	-0.23302
C	0.06111	-0.49596	0.92253
C	-3.62236	0.52227	0.32224
C	-3.39499	1.79009	0.66019
O	2.15623	-2.13475	0.10422
C	3.17686	-3.04337	-0.28132
H	4.75228	1.54581	-0.85209
H	0.24704	-1.36699	1.56282
H	0.79214	2.11530	0.70207
H	-4.00103	-1.31014	1.33753
H	-2.65090	-0.37119	1.96485
H	-1.93037	-2.47438	0.83981
H	-2.70453	-1.97645	-0.65636
H	-1.10281	-0.04257	-0.84932
H	-0.32529	-1.61994	-0.87772
H	-0.43677	0.25837	1.54313
H	-3.78835	2.62747	0.09470
H	-2.79775	2.00948	1.54078
H	4.07680	-2.92529	0.33631
H	3.44816	-2.92154	-1.33810
H	2.76369	-4.04215	-0.12885
H	2.22835	3.80764	0.14356
H	4.43448	-0.90414	-0.75817
Cl	-4.61584	0.18528	-1.11873

Conformer 47
Energy: -1116.22404 Hartree (Rel: 1.1 kcal/mol)
XYZ coordinates for conf 47:

C	-3.81556	1.12957	-0.49446
C	-3.62805	-0.25408	-0.43842
C	-2.42024	-0.78863	0.02114
C	-1.37974	0.06765	0.43970
C	-1.58890	1.44723	0.37806
C	-2.79437	1.98614	-0.08725
O	-3.02200	3.33856	-0.15749
C	3.13379	-0.70669	1.03937
C	2.17155	-1.59771	0.22360
C	0.87035	-0.92078	-0.23336
C	-0.06118	-0.49632	0.92232
C	3.62241	0.52198	0.32248
C	3.39515	1.78974	0.66075
O	-2.15650	-2.13469	0.10367
C	-3.17779	-3.04311	-0.28056
H	-4.75202	1.54640	-0.85205
H	0.43668	0.25772	1.54329
H	-0.79180	2.11509	0.70216
H	2.65078	-0.37192	1.96473
H	4.00092	-1.31071	1.33723
H	2.70448	-1.97636	-0.65698
H	1.93021	-2.47474	0.83898
H	0.32528	-1.61948	-0.87841
H	1.10292	-0.04220	-0.84925
H	-0.24721	-1.36762	1.56219
H	3.78863	2.62723	0.09552
H	2.79786	2.00895	1.54135
H	-3.44970	-2.92197	-1.33727
H	-4.07728	-2.92415	0.33755
H	-2.76500	-4.04199	-0.12764
H	-2.22771	3.80769	0.14375
H	-4.43460	-0.90363	-0.75845
Cl	4.61595	0.18534	-1.11861

Conformer 48
Energy: -1116.22396 Hartree (Rel: 1.2 kcal/mol)
XYZ coordinates for conf 48:

C	-3.96197	-0.06208	-0.73141
C	-3.17068	1.07799	-0.55299
C	-1.98012	1.00838	0.17594
C	-1.56837	-0.21794	0.74488
C	-2.37554	-1.34068	0.55844
C	-3.56531	-1.27710	-0.17587
O	-4.29143	-2.43549	-0.30567
C	2.55633	-0.58582	-1.22349
C	1.36537	-0.00757	-0.43117
C	0.91309	-0.89545	0.73359
C	-0.28080	-0.33208	1.53705
C	3.83494	-0.72805	-0.44381
C	4.46562	-1.85940	-0.13558
O	-1.15236	2.08348	0.39122
C	-1.53747	3.34391	-0.13550
H	-4.88620	0.00801	-1.30112
H	-0.01061	0.64760	1.94316
H	-2.08493	-2.29293	0.99487
H	2.29583	-1.58154	-1.60435
H	2.74616	0.05162	-2.09712
H	1.62184	0.99157	-0.05924
H	0.53495	0.12983	-1.13386
H	1.75106	-1.04488	1.42689
H	0.64863	-1.89128	0.34940
H	-0.45642	-0.99596	2.39290
H	5.39235	-1.88004	0.42700
H	4.04693	-2.80876	-0.45731
H	-2.49829	3.67791	0.27777
H	-1.60686	3.32036	-1.23102
H	-0.75482	4.04500	0.16010
H	-5.08910	-2.25058	-0.82664
H	-3.49875	2.01344	-0.99104
Cl	4.54282	0.81953	0.08969

Conformer 49
Energy: -1116.22368 Hartree (Rel: 1.4 kcal/mol)
XYZ coordinates for conf 49:

C	-3.08642	1.19030	-0.87933
C	-2.86855	-0.18560	-0.97334
C	-2.01680	-0.83550	-0.07227
C	-1.37373	-0.10251	0.94687
C	-1.61059	1.27311	1.02582
C	-2.45522	1.92666	0.12252
O	-2.70198	3.27617	0.18042
C	3.00794	-1.21164	0.03360
C	1.49077	-1.29545	0.30186
C	1.05946	-0.61621	1.60656
C	-0.44206	-0.77034	1.93875
C	3.54862	0.18010	-0.15079
C	4.41413	0.81458	0.63742
O	-1.75504	-2.18441	-0.10711
C	-2.39675	-2.96762	-1.10178
H	-3.74588	1.69484	-1.57868
H	-0.61584	-0.33279	2.93018
H	-1.12525	1.84482	1.81618
H	3.55555	-1.66396	0.87025
H	3.24449	-1.80259	-0.86100
H	0.94701	-0.85981	-0.54426
H	1.21339	-2.35636	0.33225
H	1.31117	0.45178	1.56627
H	1.63804	-1.03807	2.44110
H	-0.68749	-1.83430	2.01270
H	4.76451	1.82039	0.43405
H	4.79612	0.31426	1.52277
H	-2.11413	-2.64776	-2.11326
H	-3.48971	-2.92782	-1.00523
H	-2.05854	-3.99306	-0.94135
H	-2.18774	3.65977	0.90852

H	-3.36964	-0.74027	-1.75817
Cl	2.93855	1.01632	-1.60201

Conformer 50
Energy: -1116.22367 Hartree (Rel: 1.4 kcal/mol)
XYZ coordinates for conf 50:

C	-3.09493	1.15894	-0.87649
C	-2.84741	-0.21412	-0.98125
C	-1.99341	-0.85183	-0.07726
C	-1.37413	-0.11194	0.95512
C	-1.63957	1.25493	1.04384
C	-2.49062	1.89854	0.13826
O	-2.69208	3.24633	0.30693
C	3.02918	-1.17445	0.06541
C	1.51217	-1.28118	0.32695
C	1.06331	-0.59593	1.62264
C	-0.43737	-0.76904	1.94962
C	3.54445	0.22316	-0.14519
C	4.38734	0.89340	0.63783
O	-1.70443	-2.19479	-0.11702
C	-2.31963	-2.98401	-1.12332
H	-3.76196	1.64143	-1.58767
H	-0.61991	-0.33225	2.93956
H	-1.18229	1.84365	1.83502
H	3.57990	-1.59878	0.91460
H	3.28242	-1.77931	-0.81521
H	0.96565	-0.86401	-0.52667
H	1.25275	-2.34632	0.36737
H	1.29886	0.47529	1.57412
H	1.64379	-1.00187	2.46376
H	-0.66965	-1.83608	2.02378
H	4.71891	1.90156	0.41564
H	4.76832	0.42168	1.53919
H	-2.03266	-2.65179	-2.12965
H	-3.41419	-2.96615	-1.03830
H	-1.96306	-4.00366	-0.96591
H	-3.29995	3.56190	-0.38070
H	-3.32962	-0.77103	-1.77617
Cl	2.93521	1.01262	-1.62288

Originally assigned (correct) structure of 171{7a} (DMSO-d_6)

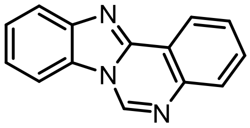

					Conf1
		Rel energy (kcal/mol):			0.00
**C-nom**	**iGau**	**Exp**	**Calc**	**diff**	**1**
C	8	145.97	148.85	2.88	[148.85]
C	5	143.91	143.13	-0.78	[143.13]
C	12	142.79	142.15	-0.64	[142.15]
C	10	139.05	137.79	-1.26	[137.79]
C	15	132.31	131.41	-0.90	[131.41]
C	4	128.95	128.64	-0.31	[128.64]
C	14	128.88	127.79	-1.09	[127.79]
C	16	128.65	127.38	-1.27	[127.38]
C	1	126.20	125.89	-0.31	[125.89]
C	17	124.18	124.98	0.80	[124.98]
C	2	123.37	122.58	-0.79	[122.58]
C	6	119.86	119.54	-0.32	[119.54]
C	13	119.25	119.21	-0.04	[119.21]
C	3	112.51	111.37	-1.14	[111.37]

13C chem shifts: RMSD=1.11ppm (MAE=0.90) N=14 {-1.27 2.88}

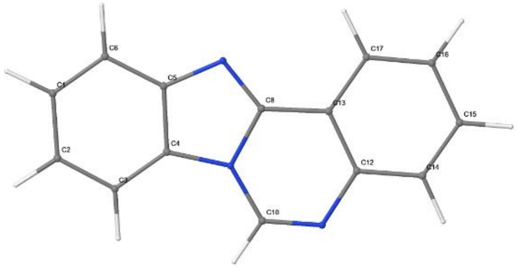

Conformer 1
Energy: -703.18823 Hartree (Rel: 0.0 kcal/mol)
XYZ coordinates for conf 1:

C	4.32205	-0.76837	0.00000
C	4.23190	0.63938	0.00000
C	2.99794	1.28758	0.00000
C	1.86550	0.47341	-0.00000
C	1.93226	-0.94145	-0.00000
C	3.18540	-1.57157	0.00000
N	0.49786	0.75753	-0.00001
C	-0.16442	-0.48179	0.00001
N	0.66270	-1.50445	-0.00001
C	-0.19042	1.95165	-0.00001
N	-1.47830	2.02880	0.00000
C	-2.21415	0.84431	0.00001
C	-1.60311	-0.43946	0.00001
C	-3.61964	0.92918	0.00000
C	-4.39207	-0.22326	0.00000
C	-3.78100	-1.49122	0.00000
C	-2.39849	-1.59991	0.00000
H	5.30380	-1.23327	0.00000
H	5.14194	1.23165	0.00000
H	2.93234	2.37096	0.00000
H	3.25569	-2.65497	-0.00000
H	0.41733	2.85180	0.00000
H	-4.07455	1.91473	0.00000
H	-5.47543	-0.14687	-0.00000
H	-4.39414	-2.38739	-0.00000
H	-1.91328	-2.57066	0.00000

Originally assigned (incorrect) structure 174{9} ($CDCl_3$) Notice that aziridine's C-N bond in this highly strained and unusual structure needs to be fixed in order to prevent rearrangement into quinoxalinium

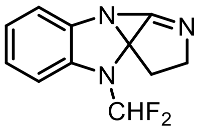

					Conf1
		Rel energy (kcal/mol):			0.00
**C-nom**	**iGau**	**Exp**	**Calc**	**diff**	**1**
C	13	148.90	197.23	48.33	[197.23]
C	8	142.10	66.08	-76.02	[66.08]
C	5	132.50	140.21	7.71	[140.21]
C	1	124.60	124.48	-0.12	[124.48]
C	6	124.10	121.78	-2.32	[121.78]
C	3	120.30	115.66	-4.64	[115.66]
C	4	118.60	143.20	24.60	[143.20]
C	2	110.10	129.38	19.28	[129.38]
C	14	108.40	110.06	1.66	[110.06]
C	11	24.60	79.85	55.25	[79.85]
C	10	14.70	33.14	18.44	[33.14]

13C chem shifts: RMSD=33.81ppm (MAE=23.49) N=11 {-76.02 55.25}

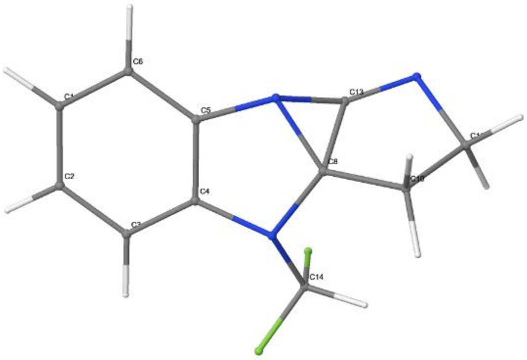

Conformer 1
Energy: -788.35846 Hartree (Rel: 0.0 kcal/mol)
XYZ coordinates for conf 1:

C	-3.38323	-1.32650	0.17279
C	-3.46818	0.06246	0.03497
C	-2.33344	0.85144	-0.20122
C	-1.10576	0.20105	-0.28294
C	-1.02488	-1.18846	-0.12297
C	-2.14441	-1.97685	0.08415
N	0.19061	0.71332	-0.55646
C	1.10490	-0.44042	-0.54517
N	0.34270	-1.60866	-0.25302
C	2.50234	-0.41531	-1.19294
C	3.40290	-0.63765	0.08381
N	2.55959	-1.49286	1.02358
C	1.37306	-1.20496	0.64485
C	0.62433	1.92342	0.03610
F	-0.17512	2.94857	-0.37516
F	0.53433	1.89621	1.41108
H	-4.28271	-1.90812	0.34925
H	-4.43751	0.54771	0.10340
H	-2.41029	1.92590	-0.31208
H	-2.05416	-3.05270	0.19207
H	2.70391	0.52069	-1.72134
H	2.63530	-1.24042	-1.90012
H	4.30835	-1.21084	-0.13563
H	3.70853	0.30106	0.56364
H	1.65964	2.14169	-0.22971

Revised structure of 174{9}, i.e cyanoethylbenzoimidazole 175{9-rev} ($CDCl_3$)

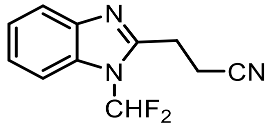

	Conf1	Conf2	Conf3	Conf4
Rel energy (kcal/mol):	0.00	0.62	0.62	0.98

C-nom	iGau	Exp	Calc	diff	1	2	3	4
C	2	148.90	150.36	1.46	[150.31	150.33	150.33	150.70]
C	6	142.10	141.22	-0.88	[141.14	141.29	141.29	141.34]
C	5	132.50	132.54	0.04	[132.42	132.62	132.62	132.86]
C	9	124.60	124.05	-0.55	[124.04	124.01	124.01	124.26]
C	8	124.10	123.73	-0.37	[123.70	123.75	123.75	123.78]
C	7	120.30	120.29	-0.01	[120.16	120.42	120.41	120.49]
C	23	118.60	119.67	1.07	[119.81	119.73	119.73	118.69]
C	4	110.10	112.46	2.36	[112.40	112.40	112.40	112.95]
C-F2	15	108.40	109.09	0.69	[108.91	108.97	108.97	110.47]
C	12	24.60	24.04	-0.56	[23.91	23.81	23.81	25.55]
C	11	14.70	14.08	-0.62	[13.75	13.78	13.78	16.89]

13C chem shifts: RMSD=1.01ppm (MAE=0.78) N=11 {-0.88 2.36}

Fractions: 0.527 0.186 0.186 0.101

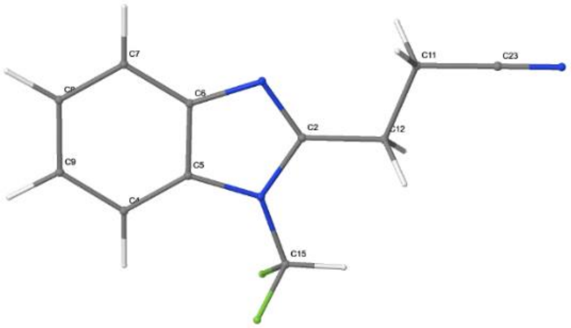

Conformer 1
Energy: -788.51868 Hartree (Rel: 0.0 kcal/mol)
XYZ coordinates for conf 1:

N	-0.26652	0.70603	-0.00137
C	0.77016	-0.22840	-0.00074
N	0.33616	-1.45972	-0.00029
C	-2.80100	0.36375	-0.00056
C	-1.46124	-0.02294	-0.00066
C	-1.05502	-1.37291	-0.00003
C	-2.01496	-2.39140	0.00072
C	-3.35723	-2.01978	0.00090
C	-3.74295	-0.66453	0.00030
H	0.87474	2.44787	-0.00425
C	3.12959	-1.06975	0.00038
C	2.21672	0.17257	-0.00097
H	2.92459	-1.69165	-0.87668
N	5.66682	-0.39459	-0.00036
C	-0.16573	2.12292	-0.00091
F	-0.80427	2.63775	-1.08926
F	-0.79680	2.63686	1.09228
H	-3.09682	1.40682	-0.00114
H	-1.71203	-3.43389	0.00116
H	-4.12533	-2.78775	0.00150
H	-4.79953	-0.41303	0.00044
H	2.92459	-1.68978	0.87877
C	4.54629	-0.69794	-0.00002
H	2.43818	0.79005	0.87850
H	2.43831	0.78814	-0.88176

Conformer 2
Energy: -788.51712 Hartree (Rel: 0.6 kcal/mol)
XYZ coordinates for conf 2:

N	0.25146	0.83766	-0.06641
C	-0.92782	0.16139	-0.38425
N	-0.74860	-1.12537	-0.51278
C	2.62550	-0.04998	0.27688
C	1.26045	-0.12990	0.00288
C	0.60679	-1.34715	-0.27682
C	1.33217	-2.54411	-0.28586
C	2.69693	-2.47904	-0.01550
C	3.33223	-1.25199	0.26054
H	-0.50483	2.77499	0.03160
C	-3.41143	-0.11987	-0.75408
C	-2.24062	0.86616	-0.57473
H	-3.17149	-0.84730	-1.53507
N	-4.02887	-1.41009	1.44911
C	0.43887	2.23489	0.10333
F	1.01336	2.48434	1.31323
F	1.30354	2.71273	-0.83701
H	3.11328	0.89406	0.49188
H	0.83687	-3.48659	-0.49755
H	3.28643	-3.39133	-0.01575
H	4.39840	-1.24023	0.46748
H	-4.30144	0.43348	-1.07212
C	-3.74366	-0.85013	0.47324
H	-2.19518	1.49956	-1.47006
H	-2.44844	1.53266	0.27038

Conformer 3
Energy: -788.51769 Hartree (Rel: 0.6 kcal/mol)
XYZ coordinates for conf 3:

N	-0.25135	0.83763	-0.06657
C	0.92780	0.16123	-0.38454
N	0.74844	-1.12551	-0.51300
C	-2.62544	-0.04973	0.27702
C	-1.26044	-0.12982	0.00285
C	-0.60694	-1.34715	-0.27692
C	-1.33247	-2.54401	-0.28585
C	-2.69720	-2.47878	-0.01532
C	-3.33232	-1.25166	0.26079
H	0.50506	2.77495	0.03086
C	3.41143	-0.12034	-0.75412
C	2.24067	0.86584	-0.57520
H	4.30149	0.43287	-1.07225
N	4.02865	-1.40983	1.44956
C	-0.43862	2.23489	0.10314
F	-1.30377	2.71263	-0.83679
F	-1.01247	2.48449	1.31331
H	-3.11306	0.89439	0.49206
H	-0.83731	-3.48656	-0.49757
H	-3.28681	-3.39099	-0.01547
H	-4.39846	-1.23979	0.46786
H	3.17149	-0.84801	-1.53488
C	3.74349	-0.85022	0.47347
H	2.44848	1.53266	0.26966
H	2.19533	1.49887	-1.47079

Conformer 4
Energy: -788.51769 Hartree (Rel: 1.0 kcal/mol)
XYZ coordinates for conf 4:

N	-0.13814	0.47846	-0.34103
C	0.56486	-0.71915	-0.51277
N	-0.20162	-1.77110	-0.40197
C	-2.61775	0.87246	0.19185
C	-1.46696	0.12597	-0.07089
C	-1.47664	-1.28437	-0.13011
C	-2.66930	-1.98868	0.07555
C	-3.82106	-1.25496	0.34338
C	-3.79269	0.15260	0.40248
H	1.33942	1.84427	-0.88724

C	2.83283	-0.60284	0.59540
C	2.04637	-0.77994	-0.73375
H	2.54386	-1.38962	1.30080
N	5.42557	-0.72095	0.20072
C	0.42512	1.78405	-0.29720
F	-0.47305	2.68569	-0.76471
F	0.71783	2.14323	0.99231
H	-2.60346	1.95445	0.23503
H	-2.68090	-3.07313	0.02762
H	-4.76076	-1.77342	0.51032
H	-4.70986	0.69407	0.61545
H	2.59220	0.35569	1.06840
C	4.28068	-0.66927	0.38525
H	2.37617	-0.03045	-1.46105
H	2.26695	-1.76222	-1.15682

Originally assigned (incorrect) structure of 180{9a} ($CDCl_3$)

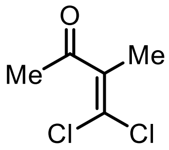

					Conf1	Conf2
		Rel energy (kcal/mol):			0.00	0.57

C-nom	iGau	Exp	Calc	diff	1	2
C	3	194.00	195.68	1.68	[195.57	195.98]
C	1	144.00	135.55	-8.45	[136.53	132.99]
C	6	127.40	131.27	3.87	[132.65	127.64]
C	4	29.80	31.50	1.70	[32.38	29.19]
C	2	25.00	17.33	-7.67	[17.15	17.80]

13C chem shifts: RMSD=5.49ppm (MAE=4.67) N=5 {-8.45 3.87}

Fractions: 0.724 0.276

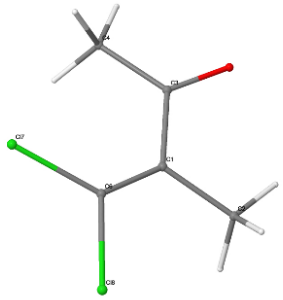

```
Conformer 1
Energy: -1189.72325 Hartree (Rel:  0.0 kcal/mol)
XYZ coordinates for conf 1:
C          0.41337       -0.66004       -0.03732
C          0.31822       -2.17158       -0.08834
C          1.82341       -0.11077        0.02140
C          2.16294        1.32093       -0.33889
O          2.72217       -0.87919        0.33669
C         -0.71740        0.07403       -0.00230
Cl        -0.85347        1.80399        0.13906
Cl        -2.29011       -0.68961       -0.04897
H          1.32214       -2.59442       -0.07097
H         -0.24058       -2.56377        0.76798
H         -0.19807       -2.50172       -0.99604
H          1.61725        1.67016       -1.21959
H          1.92084        1.99694        0.48803
H          3.23861        1.36645       -0.52172

Conformer 2
Energy: -1189.72234 Hartree (Rel:  0.6 kcal/mol)
XYZ coordinates for conf 2:
C         -0.45686       -0.55578       -0.11119
C         -0.63124       -2.05575       -0.19988
C         -1.72442        0.27138       -0.10582
C         -2.96314       -0.38997        0.47319
O         -1.77100        1.40711       -0.54706
C          0.75609        0.02269       -0.01161
Cl         1.05993        1.72610        0.14560
Cl         2.24423       -0.90309        0.02695
H         -1.31406       -2.31400       -1.01687
H          0.31395       -2.56767       -0.37700
H         -1.06284       -2.45508        0.72586
H         -2.76586       -0.79789        1.47128
H         -3.75992        0.35382        0.52899
H         -3.29642       -1.22253       -0.15719
```

Revised structure of 180{9a}, i.e. 181{9a-rev} (CDCl₃)

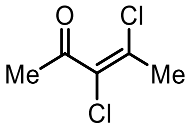

	Conf1	Conf2
Rel energy (kcal/mol):	0.00	0.81

C-nom	iGau	Exp	Calc	diff	1	2
C	3	194.00	193.22	-0.78	[193.72	191.27]
C	2	144.00	144.12	0.12	[143.98	144.68]
C	1	127.40	128.42	1.02	[127.40	132.44]
C	4	29.80	30.72	0.92	[30.16	32.91]
C	6	25.00	25.51	0.51	[25.38	26.04]

13C chem shifts: RMSD=0.74ppm (MAE=0.67) N=5 {-0.78 1.02}

Fractions: 0.797 0.203

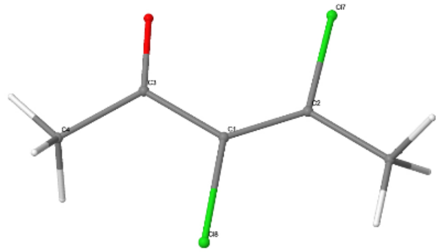

Conformer 1
Energy: -1189.72786 Hartree (Rel: 0.0 kcal/mol)
XYZ coordinates for conf 1:

C	0.31268	0.22867	0.02141
C	-1.02706	0.42139	-0.00389
C	1.05685	-1.08155	0.02701
C	2.56339	-1.05870	-0.14002
O	0.45850	-2.13620	0.16128
C	-1.75161	1.73406	-0.01914
Cl	-2.14154	-0.92862	-0.04054
Cl	1.34489	1.67082	0.04061
H	2.85813	-0.52519	-1.04985
H	3.04518	-0.54885	0.70149
H	2.91141	-2.09196	-0.18627
H	-1.06744	2.58124	-0.00402
H	-2.38053	1.79715	-0.91442
H	-2.41709	1.79644	0.84942

Conformer 2
Energy: -1189.72657 Hartree (Rel: 0.8 kcal/mol)
XYZ coordinates for conf 2:

C	-0.39915	0.23151	0.01982
C	0.91290	0.55956	0.00979
C	-1.09510	-1.11166	-0.02429
C	-0.34475	-2.40170	0.23592
O	-2.29410	-1.14065	-0.24603
C	1.50708	1.93628	0.04540
Cl	2.16951	-0.66805	-0.09168
Cl	-1.56124	1.56161	0.04555
H	0.29089	-2.33861	1.12323
H	-1.09218	-3.18698	0.36599
H	0.29863	-2.65912	-0.61137
H	2.05027	2.12933	-0.88728
H	0.74660	2.70398	0.17803
H	2.23222	2.00210	0.86396

Originally assigned (incorrect) structure of 183{5a} with in config of Me ($CDCl_3$)

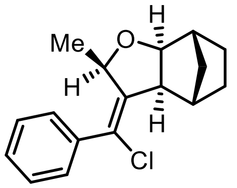

					Conf1
	Rel energy (kcal/mol):				0.00
**C-nom**	**iGau**	**Exp**	**Calc**	**diff**	**1**
C	10	146.00	146.89	0.89	[146.89]
C	14	139.50	138.04	-1.46	[138.04]
C	15	128.40	128.46	0.06	[128.46]
C	19	128.40	128.46	0.06	[128.46]
C	17	128.30	128.31	0.01	[128.31]
C	16	128.30	127.47	-0.83	[127.47]
C	18	128.30	127.47	-0.83	[127.47]
C	12	123.60	124.72	1.12	[124.72]
C	1	86.50	85.75	-0.75	[85.75]
C	9	79.50	78.07	-1.43	[78.07]
C	2	53.60	56.81	3.21	[56.81]
C	6	42.20	42.26	0.06	[42.26]
C	3	41.80	40.01	-1.79	[40.01]
C	7	33.10	33.78	0.68	[33.78]
C	4	28.70	29.71	1.01	[29.71]
C	5	22.10	23.01	0.91	[23.01]
C	11	19.70	18.45	-1.25	[18.45]

13C chem shifts: RMSD=1.23ppm (MAE=0.96) N=17 {-1.79 3.21}

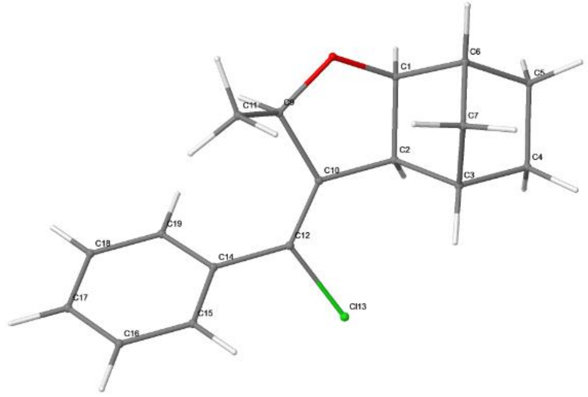

Conformer 1
Energy: -1194.63552 Hartree (Rel: 0.0 kcal/mol)
XYZ coordinates for conf 1:

C	-2.36252	-1.06546	-0.75507
C	-1.60742	0.28547	-0.56384
C	-2.41431	0.96373	0.59019
C	-3.72554	1.48748	-0.04291
C	-4.49672	0.17023	-0.38851
C	-3.57766	-0.94017	0.17980
C	-2.94026	-0.24111	1.39932
O	-1.48849	-2.09237	-0.28154
C	-0.12232	-1.66307	-0.25344
C	-0.18031	-0.13355	-0.29623
C	0.53795	-2.28760	0.97478
C	0.87925	0.68108	-0.20911
Cl	0.61668	2.45224	-0.29678
C	2.31209	0.31315	-0.09599
C	3.08361	0.73620	0.99908
C	4.42756	0.37924	1.09716

C	5.02407	-0.39687	0.09857
C	4.26926	-0.81048	-1.00078
C	2.92277	-0.45460	-1.10036
H	-2.64303	-1.26658	-1.79872
H	-1.63743	0.91839	-1.45761
H	-1.84786	1.71729	1.14015
H	-4.28273	2.08978	0.68314
H	-3.54323	2.11759	-0.92019
H	-4.66484	0.05184	-1.46548
H	-5.47800	0.14378	0.09615
H	-4.07727	-1.89544	0.36178
H	-3.67220	0.05033	2.16103
H	-2.15657	-0.83922	1.87229
H	0.38228	-2.03452	-1.15974
H	1.61261	-2.09197	0.98901
H	0.38282	-3.37127	0.95412
H	0.09533	-1.89145	1.89436
H	2.62381	1.34210	1.77449
H	5.01028	0.70668	1.95372
H	6.07230	-0.67198	0.17530
H	4.72838	-1.40353	-1.78689
H	2.34279	-0.76303	-1.96528

Originally assigned (incorrect) structure of 183{5a} with out config of Me (CDCl₃)

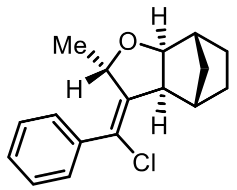

					Conf1
	Rel energy (kcal/mol):				0.00
**C-nom**	**iGau**	**Exp**	**Calc**	**diff**	**1**
C	10	146.00	147.53	1.53	[147.53]
C	14	139.50	137.96	-1.54	[137.96]
C	15	128.40	128.44	0.04	[128.44]
C	19	128.40	128.44	0.04	[128.44]
C	18	128.30	127.66	-0.64	[127.66]
C	16	128.30	127.66	-0.64	[127.66]
C	17	128.30	128.27	-0.03	[128.27]
C	12	123.60	124.35	0.75	[124.35]
C	1	86.50	85.52	-0.98	[85.52]
C	9	79.50	79.13	-0.37	[79.13]
C	2	53.60	54.17	0.57	[54.17]
C	6	42.20	42.13	-0.07	[42.13]
C	3	41.80	39.55	-2.25	[39.55]
C	7	33.10	33.33	0.23	[33.33]
C	4	28.70	29.30	0.60	[29.30]
C	5	22.10	23.30	1.20	[23.30]
C	11	19.70	19.39	-0.31	[19.39]

13C chem shifts: RMSD=0.92ppm (MAE=0.69) N=17 {-2.25 1.53}

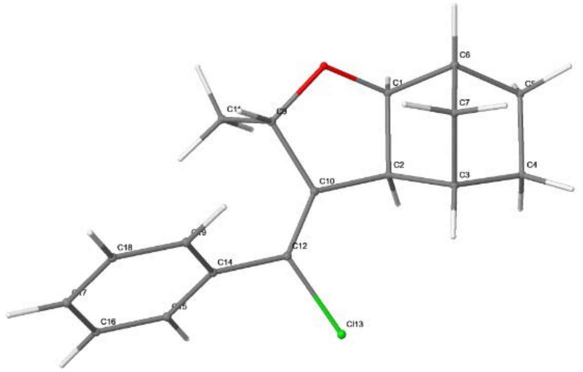

```
Conformer 1
Energy: -1194.63698 Hartree (Rel:  0.0 kcal/mol)
XYZ coordinates for conf 1:
C          2.37477        1.11154       -0.54007
C          1.62267       -0.25915       -0.52535
C          2.35577       -1.03181        0.61695
C          3.72699       -1.46993        0.04997
C          4.49683       -0.11526       -0.09259
C          3.50254        0.92111        0.48728
C          2.77695        0.10225        1.57582
O          1.44487        2.09158       -0.06437
C          0.09889        1.65229       -0.27027
C          0.18130        0.12687       -0.28294
C         -0.47049        2.22015       -1.57779
C         -0.87638       -0.68807       -0.18981
Cl        -0.64846       -2.44765       -0.43187
C         -2.28302       -0.30386        0.08460
C         -3.30653       -0.59549       -0.83278
C         -4.62229       -0.21779       -0.56812
```

C	-4.93775	0.44866	0.61978
C	-3.92986	0.73150	1.54375
C	-2.61168	0.35435	1.28069
H	2.74703	1.39766	-1.53326
H	1.71326	-0.80540	-1.47108
H	1.76722	-1.84439	1.04594
H	4.23326	-2.13733	0.75611
H	3.63275	-2.00851	-0.89928
H	4.76437	0.10959	-1.13175
H	5.42627	-0.12243	0.48592
H	3.95906	1.86452	0.79858
H	3.44427	-0.24389	2.37309
H	1.93630	0.63996	2.02415
H	-0.48480	2.03420	0.57250
H	-1.52070	1.93614	-1.69950
H	-0.40218	3.31310	-1.56314
H	0.09004	1.84925	-2.44307
H	-3.06590	-1.11804	-1.75410
H	-5.40239	-0.44388	-1.28981
H	-5.96411	0.73951	0.82563
H	-4.16871	1.23731	2.47519
H	-1.83237	0.55592	2.00986

Revised structure of 183{5a}, i.e 185{5a-rev} ($CDCl_3$)

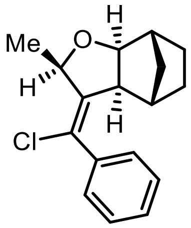

					Conf1
		Rel energy (kcal/mol):			0.00
**C-nom**	**iGau**	**Exp**	**Calc**	**diff**	**1**
C	10	146.00	146.95	0.95	[146.95]
C	14	139.50	138.72	-0.78	[138.72]
C	15	128.40	128.53	0.13	[128.53]
C	19	128.40	128.53	0.13	[128.53]
C	17	128.30	128.14	-0.16	[128.14]
C	16	128.30	127.91	-0.39	[127.91]
C	18	128.30	127.91	-0.39	[127.91]
C	12	123.60	123.48	-0.12	[123.48]
C	1	86.50	86.87	0.37	[86.87]
C	9	79.50	79.67	0.17	[79.67]
C	2	53.60	54.80	1.20	[54.80]
C	6	42.20	42.63	0.43	[42.63]
C	3	41.80	41.09	-0.71	[41.09]
C	7	33.10	33.49	0.39	[33.49]
C	4	28.70	29.43	0.73	[29.43]
C	5	22.10	22.84	0.74	[22.84]
C	11	19.70	19.27	-0.43	[19.27]

13C chem shifts: RMSD=0.57ppm (MAE=0.48) N=17 {-0.78 1.20}

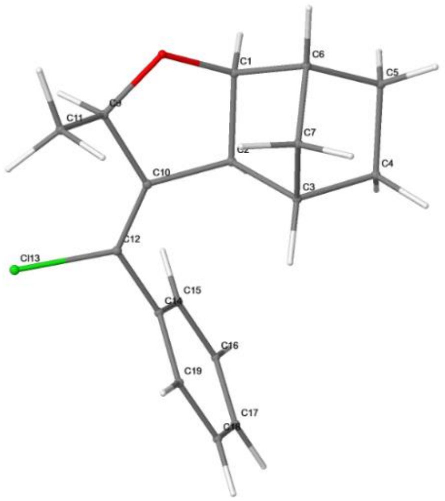

Conformer 1
Energy: -1194.63406 Hartree (Rel: 0.0 kcal/mol)
XYZ coordinates for conf 1:

C	2.42111	-0.11737	-0.91638
C	0.95554	-0.44102	-0.49301
C	1.15274	-1.37148	0.74458
C	1.58001	-2.75233	0.19189
C	3.01570	-2.47541	-0.36645
C	3.26948	-0.99664	0.02072
C	2.48478	-0.87160	1.34401
O	2.64174	1.26718	-0.64012
C	1.42284	1.98764	-0.41161
C	0.33796	0.92786	-0.27487

C	1.64306	2.90812	0.79147
C	-0.97653	1.12035	-0.12167
Cl	-1.64780	2.77750	-0.02060
C	-2.02387	0.06669	-0.06554
C	-2.45805	-0.56152	-1.24294
C	-3.44190	-1.55093	-1.19502
C	-4.00715	-1.91859	0.02820
C	-3.58616	-1.29216	1.20443
C	-2.60241	-0.30365	1.15852
H	2.62159	-0.31451	-1.97864
H	0.40593	-0.97571	-1.27268
H	0.28866	-1.40133	1.41227
H	1.60827	-3.49189	0.99972
H	0.89033	-3.12761	-0.57192
H	3.08326	-2.63334	-1.44930
H	3.75830	-3.12928	0.10204
H	4.32321	-0.70620	0.04089
H	2.86490	-1.52912	2.13397
H	2.44946	0.15213	1.72568
H	1.20323	2.60900	-1.29556
H	0.80404	3.59391	0.92536
H	2.55065	3.49718	0.62387
H	1.77345	2.32796	1.71044
H	-2.02539	-0.26855	-2.19555
H	-3.76879	-2.03055	-2.11353
H	-4.77376	-2.68765	0.06495
H	-4.02332	-1.57339	2.15853
H	-2.27822	0.18616	2.07254

Originally assigned (incorrect) structure of 184{5b} ($CDCl_3$)

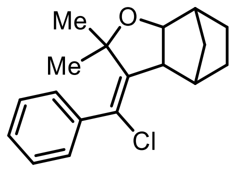

					**Conf1**
	Rel energy (kcal/mol):				0.00

C-nom	iGau	Exp	Calc	diff	1
C	10	148.00	149.88	1.88	[149.88]
C	14	140.40	137.43	-2.97	[137.43]
C	15	128.40	129.83	1.43	[129.83]
C	19	128.40	129.83	1.43	[129.83]
C	18	128.30	127.33	-0.97	[127.33]
C	16	128.30	127.33	-0.97	[127.33]
C	17	128.10	128.36	0.26	[128.36]
C	12	122.10	125.38	3.28	[125.38]
C	9	85.00	85.02	0.02	[85.02]
C	1	82.80	82.86	0.06	[82.86]
C	2	53.70	56.62	2.92	[56.62]
C	6	41.80	41.97	0.17	[41.97]
C	3	39.90	40.00	0.10	[40.00]
C	7	32.60	33.50	0.90	[33.50]
C	4	28.30	29.60	1.30	[29.60]
C	30	25.90	26.40	0.50	[26.40]
C	11	25.50	27.34	1.84	[27.34]
C	5	22.30	23.19	0.89	[23.19]

13C chem shifts: RMSD=1.57ppm (MAE=1.22) N=18 {-2.97 3.28}

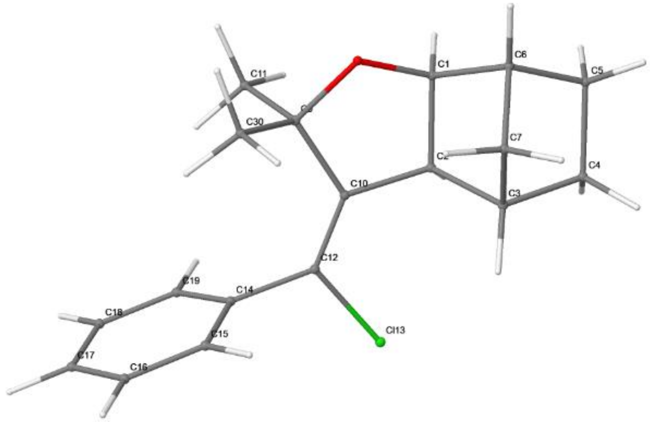

Conformer 1
Energy: -1233.94988 Hartree (Rel: 0.0 kcal/mol)
XYZ coordinates for conf 1:

C	2.43294	1.01734	-0.55209
C	1.64279	-0.32725	-0.54138
C	2.39132	-1.14681	0.55891
C	3.71958	-1.62353	-0.07594
C	4.52896	-0.29358	-0.23450
C	3.60263	0.76504	0.41413
C	2.90028	-0.05095	1.51945
O	1.55633	1.99941	-0.00031
C	0.17293	1.62844	-0.16738
C	0.21364	0.09111	-0.26487
C	-0.35020	2.24601	-1.47884
C	-0.84274	-0.72929	-0.22515
Cl	-0.58539	-2.48825	-0.50695
C	-2.27994	-0.41689	-0.00653

C	-2.82660	-0.45280	1.28602
C	-4.18156	-0.18793	1.48861
C	-5.00920	0.10638	0.40177
C	-4.47709	0.13051	-0.88955
C	-3.12185	-0.13515	-1.09397
H	2.76584	1.32379	-1.55374
H	1.69194	-0.85823	-1.49852
H	1.79243	-1.94567	0.99860
H	4.23354	-2.31805	0.59781
H	3.56454	-2.14437	-1.02706
H	4.75007	-0.05811	-1.28225
H	5.48641	-0.34400	0.29404
H	4.10774	1.68463	0.72179
H	3.59228	-0.44062	2.27461
H	2.10482	0.50740	2.02076
C	-0.56270	2.20570	1.04465
H	-1.41170	2.02534	-1.62435
H	-0.21930	3.33322	-1.44363
H	0.20342	1.86112	-2.34215
H	-2.18364	-0.68398	2.13043
H	-4.59060	-0.21230	2.49487
H	-6.06414	0.31228	0.56039
H	-5.11639	0.35323	-1.73934
H	-2.71177	-0.12931	-2.09982
H	-1.64284	2.07025	0.96081
H	-0.35108	3.27847	1.10796
H	-0.21980	1.73174	1.96956

Revised structure of 184{5b}, i.e 186{5b-rev} ($CDCl_3$)

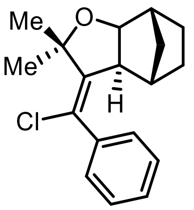

					Conf1	Conf2
	Rel energy (kcal/mol):				0.00	0.00

C-nom	iGau	Exp	Calc	diff	1	2
C	10	148.00	148.96	0.96	[148.96	148.95]
C	14	140.40	139.65	-0.75	[139.65	139.64]
C	15	128.40	128.50	0.10	[128.50	128.50]
C	19	128.40	128.50	0.10	[128.50	128.50]
C	18	128.30	127.69	-0.61	[127.69	127.68]
C	16	128.30	127.69	-0.61	[127.69	127.68]
C	17	128.10	127.92	-0.18	[127.92	127.93]
C	12	122.10	122.82	0.72	[122.82	122.82]
C	9	85.00	86.64	1.64	[86.65	86.63]
C	1	82.80	83.78	0.98	[83.78	83.78]
C	2	53.70	54.82	1.12	[54.82	54.82]
C	6	41.80	42.24	0.44	[42.24	42.24]
C	3	39.90	40.41	0.51	[40.41	40.40]
C	7	32.60	33.04	0.44	[33.04	33.04]
C	4	28.30	29.20	0.90	[29.20	29.20]
C	30	25.90	25.77	-0.13	[25.77	25.77]
C	11	25.50	24.66	-0.84	[24.66	24.66]
C	5	22.30	23.02	0.72	[23.02	23.02]

13C chem shifts: RMSD=0.76ppm (MAE=0.65) N=18 {-0.84 1.64}

Fractions: 0.500 0.500

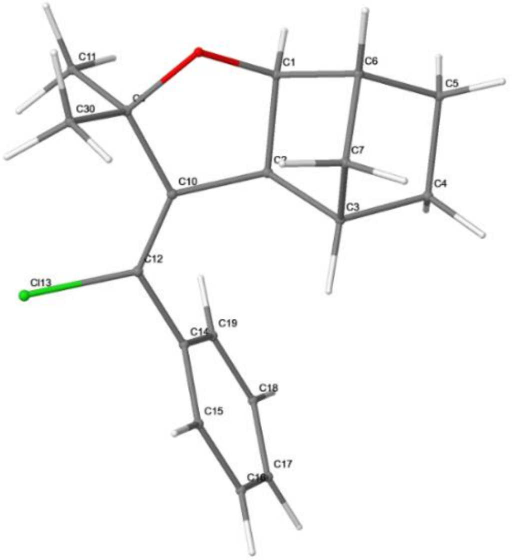

Conformer 1
Energy: -1233.94958 Hartree (Rel: 0.0 kcal/mol)
XYZ coordinates for conf 1:

C	-2.39816	-0.47955	0.67163
C	-0.85690	-0.62297	0.48266
C	-0.75123	-1.69914	-0.64243
C	-1.07671	-3.06194	0.01417
C	-2.60360	-2.94093	0.33447
C	-2.98150	-1.55996	-0.25642

C	-2.03221	-1.45527	-1.46892
O	-2.74768	0.81635	0.19191
C	-1.62044	1.71828	0.18987
C	-0.39742	0.79126	0.15622
C	-1.63885	2.54361	1.49072
C	0.89212	1.10026	-0.03002
Cl	1.41874	2.78117	-0.36182
C	2.04428	0.16195	0.04265
C	2.42107	-0.40160	1.27117
C	3.50463	-1.27919	1.34567
C	4.22809	-1.59901	0.19492
C	3.86641	-1.03380	-1.03144
C	2.78536	-0.15573	-1.10705
H	-2.72315	-0.59723	1.71509
H	-0.36070	-0.98199	1.38876
H	0.19659	-1.67710	-1.18377
H	-0.88663	-3.87778	-0.69200
H	-0.46766	-3.25162	0.90477
H	-2.81395	-2.99881	1.40894
H	-3.17691	-3.73784	-0.15016
H	-4.04896	-1.42425	-0.44989
H	-2.20476	-2.23342	-2.22099
H	-2.06918	-0.47687	-1.95619
C	-1.82222	2.61139	-1.04045
H	-0.79888	3.24389	1.52709
H	-2.57354	3.11233	1.54819
H	-1.58088	1.88865	2.36682
H	1.86838	-0.14252	2.16980
H	3.78603	-1.70688	2.30407
H	5.07178	-2.28111	0.25303
H	4.42693	-1.27647	-1.93003
H	2.50962	0.28749	-2.05988
H	-1.11495	3.44160	-1.06169
H	-2.83792	3.01965	-1.00720
H	-1.71449	2.03351	-1.96366

Conformer 2
Energy: -1233.94958 Hartree (Rel: 0.0 kcal/mol)
XYZ coordinates for conf 2:

C	2.39848	-0.47947	-0.67098
C	0.85719	-0.62296	-0.48278
C	0.75094	-1.69931	0.64199
C	1.07700	-3.06201	-0.01460
C	2.60404	-2.94081	-0.33401
C	2.98145	-1.55989	0.25730
C	2.03141	-1.45545	1.46925
O	2.74770	0.81641	-0.19081
C	1.62057	1.71828	-0.18995
C	0.39753	0.79134	-0.15633
C	1.63927	2.54278	-1.49143
C	-0.89204	1.10001	0.02994
Cl	-1.41873	2.78113	0.36173
C	-2.04431	0.16185	-0.04271
C	-2.78585	-0.15513	1.10686
C	-3.86700	-1.03309	1.03128
C	-4.22828	-1.59887	-0.19491
C	-3.50434	-1.27976	-1.34557
C	-2.42069	-0.40231	-1.27110
H	2.72407	-0.59693	-1.71427
H	0.36149	-0.98171	-1.38925
H	-0.19718	-1.67756	1.18282
H	0.88656	-3.87798	0.69133
H	0.46848	-3.25161	-0.90559
H	2.81506	-2.99855	-1.40836
H	3.17718	-3.73768	0.15088
H	4.04877	-1.42402	0.45141
H	2.20359	-2.23366	2.22135
H	2.06798	-0.47711	1.95667
C	1.82186	2.61234	1.03970
H	0.79927	3.24300	-1.52841
H	2.57398	3.11146	-1.54900

H	1.58154	1.88728	-2.36714
H	-2.51041	0.28852	2.05957
H	-4.42791	-1.27523	1.92977
H	-5.07206	-2.28087	-0.25297
H	-3.78546	-1.70796	-2.30382
H	-1.86764	-0.14372	-2.16965
H	1.11464	3.44261	1.06005
H	2.83759	3.02054	1.00654
H	1.71379	2.03524	1.96338

Originally assigned correct structure of mono-iodide 187{21a} ($CDCl_3$)

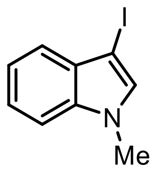

	Conf1
Rel energy (kcal/mol):	0.00

C-nom	iGau	Exp	Calc	diff	1
C	4	136.80	135.09	-1.71	[135.09]
C	8	132.70	132.91	0.21	[132.91]
C	5	130.40	129.06	-1.34	[129.06]
C	2	122.60	121.65	-0.95	[121.65]
C	1	121.10	119.89	-1.21	[119.89]
C	6	120.20	119.09	-1.11	[119.09]
C	3	109.40	109.09	-0.31	[109.09]
C	9	54.70	54.84	0.14	[54.84]
C	10	33.10	30.85	-2.25	[30.85]

13C chem shifts: RMSD=1.23ppm (MAE=1.03) N=9 {-2.25 0.21}

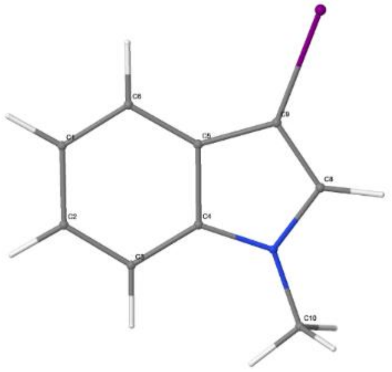

Conformer 1
Energy: -7322.38391 Hartree (Rel: 0.0 kcal/mol)
XYZ coordinates for conf 1:

C	-2.25478	-2.46862	-0.00000
C	-3.40464	-1.65045	0.00000
C	-3.30620	-0.26375	0.00001
C	-2.02309	0.30097	-0.00001
C	-0.85194	-0.51410	-0.00000
C	-0.98230	-1.91295	-0.00000
N	-1.62481	1.62792	-0.00002
C	-0.24720	1.67474	-0.00001
C	0.25366	0.39686	-0.00000
C	-2.48556	2.80222	0.00001
I	2.30401	-0.10687	0.00000
H	-2.36912	-3.54895	-0.00000
H	-4.38835	-2.11153	0.00001
H	-4.19958	0.35321	0.00002
H	-0.09797	-2.54428	-0.00000
H	0.27053	2.62326	-0.00001
H	-3.52852	2.48525	-0.00034
H	-2.30698	3.41104	0.89200
H	-2.30649	3.41139	-0.89163

Originally assigned (incorrect) structure of 188{21a'} ($CDCl_3$)

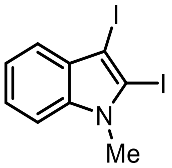

					Conf1
	Rel energy (kcal/mol):				0.00
**C-nom**	**iGau**	**Exp**	**Calc**	**diff**	**1**
C	4	183.30	136.65	-46.65	[136.65]
C	5	158.20	128.87	-29.33	[128.87]
C	2	151.40	122.12	-29.28	[122.12]
C	1	138.40	120.38	-18.02	[120.38]
C	6	125.30	118.93	-6.37	[118.93]
C	3	123.80	109.22	-14.58	[109.22]
C	8	117.40	90.20	-27.20	[90.20]
C	9	109.90	69.82	-40.08	[69.82]
C	10	26.20	32.22	6.02	[32.22]

13C chem shifts: RMSD=27.60ppm (MAE=24.17) N=9 {-46.65 6.02}

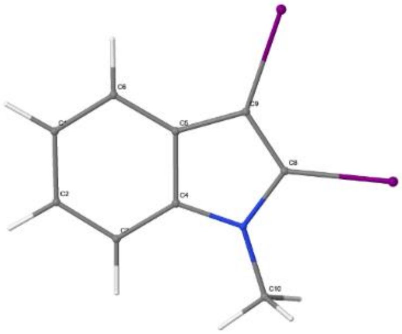

Conformer 1
Energy: -14241.63663 Hartree (Rel: 0.0 kcal/mol)
XYZ coordinates for conf 1:

C	4.28472	0.14552	0.00001
C	4.33459	-1.26517	0.00001
C	3.17532	-2.03098	0.00000
C	1.94646	-1.35478	-0.00001
C	1.88136	0.06677	-0.00000
C	3.07060	0.81665	0.00000
N	0.65150	-1.85350	-0.00002
C	-0.21912	-0.77648	-0.00001
C	0.48962	0.40485	-0.00000
C	0.28091	-3.26189	-0.00000
I	-2.30569	-1.04656	0.00000
I	-0.28651	2.36082	-0.00000
H	5.21202	0.71113	0.00001
H	5.29904	-1.76490	0.00001
H	3.23063	-3.11485	0.00001
H	3.03099	1.90212	0.00001
H	1.19080	-3.86106	-0.00020
H	-0.30181	-3.51030	0.89170
H	-0.30213	-3.51022	-0.89152

Revised 188{21a'} as *N*-methylisatin 189{21a'-rev} ($CDCl_3$)

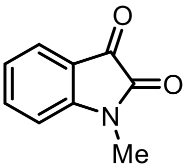

					Conf1
	Rel energy (kcal/mol):				0.00

C-nom	iGau	Exp	Calc	diff	1
C-C	9	183.30	183.20	-0.10	[183.20]
C-C	8	158.20	158.21	0.01	[158.21]
C-C	4	151.40	151.41	0.01	[151.41]
C-C	5	117.40	116.44	-0.96	[116.44]
C-CH	2	138.40	138.49	0.09	[138.49]
C-CH	6	125.30	126.26	0.96	[126.26]
C-CH	1	123.80	123.03	-0.77	[123.03]
C-CH	3	109.90	110.30	0.40	[110.30]
C-CH3	10	26.20	24.84	-1.36	[24.84]

13C chem shifts: RMSD=0.70ppm (MAE=0.52) N=9 {-1.36 0.96}

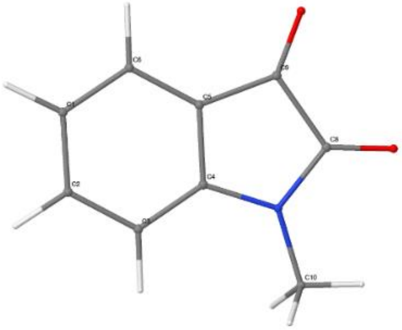

Conformer 1
Energy: -552.37602 Hartree (Rel: 0.0 kcal/mol)
XYZ coordinates for conf 1:

C	2.91907	-0.50830	-0.00016
C	2.74164	0.88094	0.00007
C	1.46763	1.46990	0.00023
C	0.36958	0.61923	-0.00005
C	0.53599	-0.78196	0.00001
C	1.80588	-1.35353	-0.00010
N	-0.99605	0.96385	-0.00057
C	-1.78762	-0.16556	-0.00015
C	-0.80107	-1.38108	0.00040
C	-1.51461	2.31994	0.00014
O	-1.15637	-2.54304	0.00010
O	-3.00379	-0.21185	-0.00001
H	3.92116	-0.92478	-0.00028
H	3.61426	1.52822	0.00014
H	1.35524	2.54865	0.00060
H	1.91512	-2.43407	-0.00006
H	-2.60333	2.25323	-0.00890
H	-1.19120	2.85881	0.89718
H	-1.17651	2.86457	-0.88787

Originally assigned (incorrect) structure of 190{5-4} ($CDCl_3$)

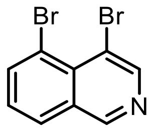

	Conf1
Rel energy (kcal/mol):	0.00

C-nom	iGau	Exp	Calc	diff	1
C-C	5	131.40	133.84	2.44	[133.84]
C-C	4	130.90	131.57	0.67	[131.57]
C-C	6	127.20	119.09	-8.11	[119.09]
C-C	10	117.40	116.74	-0.66	[116.74]
C-CH	7	152.30	153.65	1.35	[153.65]
C-CH	9	145.80	149.32	3.52	[149.32]
C-CH	1	138.90	138.97	0.07	[138.97]
C-CH	3	128.60	130.15	1.55	[130.15]
C-CH	2	128.20	126.87	-1.33	[126.87]

13C chem shifts: RMSD=3.18ppm (MAE=2.19) N=9 {-8.11 3.52}

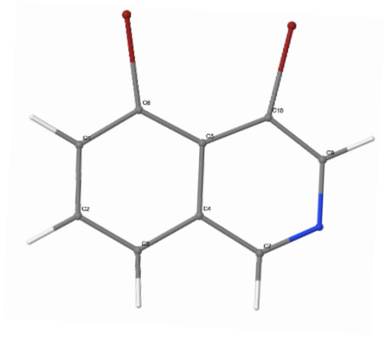

Conformer 1
Energy: -5543.51338 Hartree (Rel: 0.0 kcal/mol)
XYZ coordinates for conf 1:

C	-1.20082	2.37661	0.00002
C	-2.60141	2.22211	0.00001
C	-3.13545	0.95859	-0.00000
C	-2.28868	-0.17709	-0.00001
C	-0.84710	-0.05219	0.00000
C	-0.34712	1.29023	0.00001
C	-2.87975	-1.46805	-0.00001
N	-2.20763	-2.59727	-0.00000
C	-0.85747	-2.49832	0.00000
C	-0.15723	-1.30720	0.00000
Br	1.74322	-1.54854	0.00000
H	-0.77553	3.37358	0.00002
H	-3.23639	3.10220	0.00002
H	-4.21033	0.80289	-0.00001
Br	1.50942	1.77162	-0.00001
H	-3.96707	-1.53813	-0.00002
H	-0.30951	-3.43551	0.00001

Revised structure of 190{5-4}, i.e 193{5-4-rev} ($CDCl_3$)

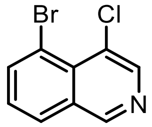

				Rel energy (kcal/mol):	**Conf1** 0.00
**C-nom**	**iGau**	**Exp**	**Calc**	**diff**	**1**
C-C	5	131.40	132.19	0.79	[132.19]
C-C	4	130.90	131.14	0.24	[131.14]
C-C	10	127.20	126.45	-0.75	[126.45]
C-C	6	117.40	118.35	0.95	[118.35]
C-CH	7	152.30	152.94	0.64	[152.94]
C-CH	9	145.80	145.82	0.02	[145.82]
C-CH	1	138.90	138.85	-0.05	[138.85]
C-CH	3	128.60	129.70	1.10	[129.70]
C-CH	2	128.20	127.12	-1.08	[127.12]

13C chem shifts: RMSD=0.74ppm (MAE=0.62) N=9 {-1.08 1.10}

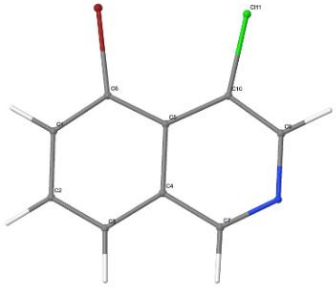

Conformer 1
Energy: -3432.30344 Hartree (Rel: 0.0 kcal/mol)
XYZ coordinates for conf 1:

C	-0.60771	2.23980	0.00002
C	0.58752	2.98717	0.00002
C	1.79152	2.32919	0.00001
C	1.83405	0.91311	-0.00000
C	0.62517	0.12031	0.00000
C	-0.60192	0.85827	0.00001
C	3.09674	0.26293	-0.00002
N	3.26566	-1.04050	-0.00002
C	2.14456	-1.79815	-0.00001
C	0.85859	-1.29277	0.00000
Cl	-0.40443	-2.51306	0.00003
H	-1.55983	2.75783	0.00002
H	0.53934	4.07134	0.00003
H	2.73020	2.87549	0.00001
Br	-2.35281	0.07361	-0.00002
H	3.99471	0.88007	-0.00002
H	2.28835	-2.87484	-0.00001

Originally assigned (incorrect) structure of 191{5-5} ($CDCl_3$)

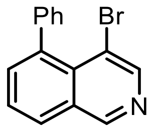

					Conf1
		Rel energy (kcal/mol):			0.00
**C-nom**	**iGau**	**Exp**	**Calc**	**diff**	**1**
C-C	15	141.50	141.54	0.04	[141.54]
C-C	6	138.80	140.61	1.81	[140.61]
C-C	5	131.10	133.50	2.40	[133.50]
C-C	4	130.50	130.52	0.02	[130.52]
C-C	10	127.00	118.17	-8.83	[118.17]
C-CH	7	152.00	152.91	0.91	[152.91]
C-CH	9	144.70	147.63	2.93	[147.63]
C-CH	1	134.70	135.93	1.23	[135.93]
C-CH	18	129.80	129.81	0.01	[129.81]
C-CH	22	129.80	129.81	0.01	[129.81]
C-CH	3	128.10	128.72	0.62	[128.72]
C-CH	20	127.50	126.88	-0.62	[126.88]
C-CH	19	127.50	126.66	-0.84	[126.66]
C-CH	21	127.40	126.66	-0.74	[126.66]
C-CH	2	127.40	126.74	-0.66	[126.74]

13C chem shifts: RMSD=2.59ppm (MAE=1.44) N=15 {-8.83 2.93}

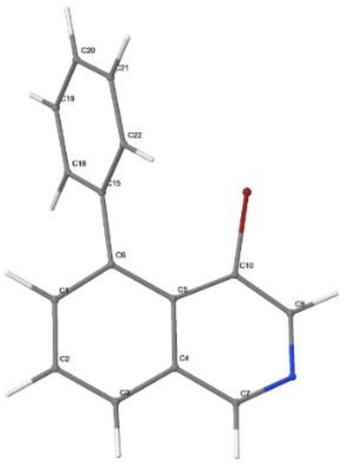

Conformer 1
Energy: -3203.76463 Hartree (Rel: 0.0 kcal/mol)
XYZ coordinates for conf 1:

C	-0.38849	2.64484	-0.15335
C	-1.70499	3.15266	-0.14221
C	-2.75931	2.28446	-0.00502
C	-2.52551	0.88860	0.07250
C	-1.18715	0.35507	0.01421
C	-0.09703	1.29120	-0.05357
C	-3.62730	0.00162	0.19626
N	-3.52859	-1.30948	0.24121
C	-2.28214	-1.83087	0.12900
C	-1.13371	-1.07509	0.00769
Br	0.46245	-2.10299	-0.25508
H	0.44233	3.34206	-0.21001
H	-1.86998	4.22332	-0.21552
H	-3.78357	2.64463	0.03926
C	1.35498	0.94109	0.04260
H	-4.63026	0.42397	0.25599
H	-2.21400	-2.91463	0.12599
C	2.20514	1.12295	-1.05749

C	3.57591	0.88201	-0.94360
C	4.11679	0.47215	0.27691
C	3.27991	0.30859	1.38436
C	1.90989	0.54236	1.26851
H	1.78638	1.44119	-2.00840
H	4.22013	1.01689	-1.80835
H	5.18377	0.28724	0.36694
H	3.69469	0.00068	2.34048
H	1.26298	0.41597	2.13238

Revised structure of 191{5-5}, i.e 194{5-5-rev} (CDCl₃)

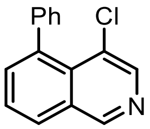

					Conf1
		Rel energy (kcal/mol):			0.00
**C-nom**	**iGau**	**Exp**	**Calc**	**diff**	**1**
C-C	15	141.50	141.87	0.37	[141.87]
C-C	6	138.80	140.02	1.22	[140.02]
C-C	5	131.10	131.68	0.58	[131.68]
C-C	4	130.50	130.13	-0.37	[130.13]
C-C	10	127.00	128.05	1.05	[128.05]
C-CH	7	152.00	152.30	0.30	[152.30]
C-CH	9	144.70	144.11	-0.59	[144.11]
C-CH	1	134.70	135.84	1.14	[135.84]
C-CH	18	129.80	129.33	-0.47	[129.33]
C-CH	22	129.80	129.33	-0.47	[129.33]
C-CH	3	128.10	128.44	0.34	[128.44]
C-CH	2	127.50	127.03	-0.47	[127.03]
C-CH	20	127.50	126.76	-0.74	[126.76]
C-CH	19	127.40	126.67	-0.73	[126.67]
C-CH	21	127.40	126.67	-0.73	[126.67]

13C chem shifts: RMSD=0.70ppm (MAE=0.64) N=15 {-0.74 1.22}

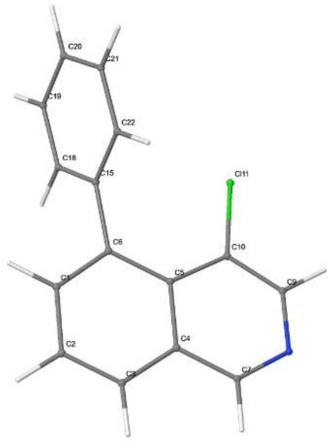

Conformer 1
Energy: -1092.55441 Hartree (Rel: 0.0 kcal/mol)
XYZ coordinates for conf 1:

C	-0.17901	2.34881	-0.05765
C	-1.46646	2.92562	-0.01407
C	-2.56779	2.11072	0.07456
C	-2.41138	0.70183	0.07384
C	-1.10359	0.10328	-0.01247
C	0.03784	0.97792	-0.03447
C	-3.55856	-0.13225	0.14573
N	-3.52824	-1.44742	0.11688
C	-2.31049	-2.02697	-0.01849
C	-1.12431	-1.32545	-0.09458
Cl	0.31290	-2.30281	-0.36676
H	0.68835	3.00213	-0.07977
H	-1.57245	4.00614	-0.02700
H	-3.57051	2.52443	0.13855
C	1.46768	0.53869	0.02650
H	-4.53855	0.33765	0.22715
H	-2.29486	-3.11158	-0.07883
C	2.31804	0.73476	-1.07107
C	3.67297	0.40648	-0.98824

C	4.19839	-0.10848	0.19857
C	3.36196	-0.29032	1.30359
C	2.00774	0.03228	1.21886
H	1.91158	1.13484	-1.99609
H	4.31671	0.55514	-1.85109
H	5.25296	-0.36191	0.26453
H	3.76511	-0.68089	2.23414
H	1.36189	-0.10785	2.08147

Originally assigned (incorrect) structure of 192{5-24} ($CDCl_3$)

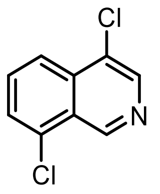

					Conf1
	Rel energy (kcal/mol):				0.00
**C-nom**	**iGau**	**Exp**	**Calc**	**diff**	**1**
C-C	5	134.80	134.07	-0.73	[134.07]
C-C	3	127.90	133.08	5.18	[133.08]
C-C	10	127.50	128.52	1.02	[128.52]
C-C	4	122.50	126.14	3.64	[126.14]
C-CH	7	150.70	147.96	-2.74	[147.96]
C-CH	9	142.70	142.55	-0.15	[142.55]
C-CH	1	132.20	130.88	-1.32	[130.88]
C-CH	2	131.50	128.69	-2.81	[128.69]
C-CH	6	123.20	123.26	0.06	[123.26]

13C chem shifts: RMSD=2.56ppm (MAE=1.96) N=9 {-2.81 5.18}

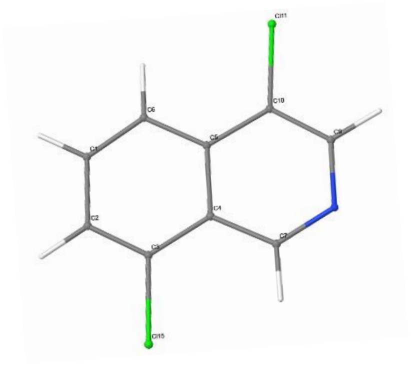

```
Conformer 1
Energy: -1321.10379 Hartree (Rel:  0.0 kcal/mol)
XYZ coordinates for conf 1:
C          0.69286        2.44680       -0.00000
C          1.89349        1.69898       -0.00000
C          1.83742        0.32487        0.00000
C          0.59533       -0.37291       -0.00000
C         -0.60992        0.40682        0.00000
C         -0.53393        1.82303       -0.00000
C          0.47999       -1.78991       -0.00000
N         -0.66807       -2.43738       -0.00000
C         -1.81342       -1.71039        0.00000
C         -1.82486       -0.33448        0.00000
Cl        -3.37239        0.49627        0.00000
H         -1.44776        2.40520       -0.00000
H          0.75074        3.53093       -0.00000
H          2.85264        2.20472       -0.00000
Cl         3.34791       -0.57690        0.00000
H          1.38089       -2.39702       -0.00000
H         -2.74557       -2.26827        0.00000
```

Revised structure of 192{5-24}, i.e 195{5-24-rev} ($CDCl_3$)

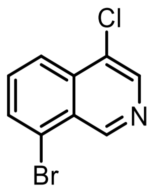

					Conf1
	Rel energy (kcal/mol):				0.00
**C-nom**	**iGau**	**Exp**	**Calc**	**diff**	**1**
C-C	5	134.80	134.27	-0.53	[134.27]
C-C	10	127.90	128.53	0.63	[128.53]
C-C	4	127.50	127.75	0.25	[127.75]
C-C	3	122.50	122.36	-0.14	[122.36]
C-CH	7	150.70	149.35	-1.35	[149.35]
C-CH	9	142.70	142.38	-0.32	[142.38]
C-CH	2	132.20	132.68	0.48	[132.68]
C-CH	1	131.50	130.68	-0.82	[130.68]
C-CH	6	123.20	123.75	0.55	[123.75]

13C chem shifts: RMSD=0.66ppm (MAE=0.56) N=9 {-1.35 0.63}

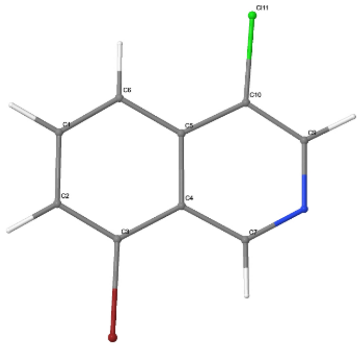

Conformer 1
Energy: -3432.31492 Hartree (Rel: 0.0 kcal/mol)
XYZ coordinates for conf 1:

C	0.05966	2.55686	-0.00000
C	-1.19531	1.90387	0.00000
C	-1.24781	0.53006	0.00000
C	-0.06466	-0.26222	-0.00000
C	1.19849	0.42118	-0.00000
C	1.23353	1.83896	-0.00000
C	-0.05604	-1.68393	-0.00000
N	1.03830	-2.41809	-0.00000
C	2.23635	-1.78196	0.00000
C	2.35297	-0.41108	0.00000
Cl	3.95976	0.29854	0.00000
H	2.19033	2.34745	-0.00000
H	0.08579	3.64226	-0.00000
H	-2.10986	2.48575	0.00000
Br	-2.97070	-0.30475	0.00000
H	-1.00190	-2.21839	-0.00000
H	3.12291	-2.40976	0.00000

Originally assigned (incorrect) structure of 196{2i} (mix DMSO-d_6 & $CDCl_3$)

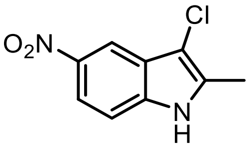

					Conf1
Rel energy (kcal/mol):					0.00
**C-nom**	**iGau**	**Exp**	**Calc**	**diff**	**1**
C-C	8	140.70	140.32	-0.38	[142.80]
C-C	5	137.62	136.14	-1.48	[138.52]
C-C	2	136.58	134.52	-2.06	[136.86]
C-C	6	126.19	122.56	-3.63	[124.60]
C-C	3	89.91	103.79	13.88	[105.36]
C-CH	9	116.07	116.16	0.09	[118.04]
C-CH	7	113.82	113.15	-0.67	[114.96]
C-CH	4	110.49	109.38	-1.11	[111.09]
C-CH3	11	11.51	10.10	-1.41	[9.35]

13C chem shifts: CRMSD=4.90ppm (CMAE=2.74) N=9 {-3.63 13.88}

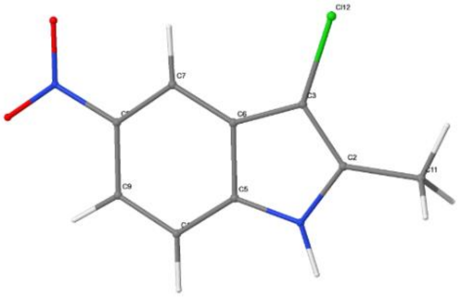

Conformer 1
Energy: -1067.23451 Hartree (Rel: 0.0 kcal/mol)
XYZ coordinates for conf 1:

N	1.77158	-1.73721	0.00001
C	2.57869	-0.60731	0.00001
C	1.74854	0.48955	0.00002
C	-0.70669	-2.17873	-0.00001
C	0.44880	-1.38470	0.00001
C	0.38652	0.04254	0.00001
C	-0.85395	0.68080	-0.00001
C	-1.98966	-0.12609	-0.00002
C	-1.93369	-1.53658	-0.00002
H	2.12282	-2.68528	0.00010
C	4.06608	-0.70970	0.00006
Cl	2.25578	2.15371	0.00002
H	-0.64416	-3.26203	-0.00001
H	-0.94571	1.75956	-0.00001
N	-3.29578	0.51646	-0.00004
H	-2.85657	-2.10131	-0.00003
O	-4.30326	-0.20023	-0.00004
O	-3.34509	1.75195	-0.00004
H	4.42519	-1.24944	-0.88443
H	4.42524	-1.24822	0.88527
H	4.51323	0.28645	-0.00062

Revised structure of 196{2i}, i.e 206{3i} (mix DMSO-d_6 & $CDCl_3$)

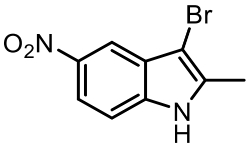

					Conf1
		Rel energy (kcal/mol):			0.00

C-nom	iGau	Exp	Calc	diff	1
C-C	8	140.70	140.80	0.10	[142.73]
C-C	5	137.62	137.75	0.13	[139.61]
C-C	2	136.58	137.68	1.10	[139.53]
C-C	6	126.19	125.20	-0.99	[126.73]
C-C	3	89.91	90.89	0.98	[91.55]
C-CH	9	116.07	116.54	0.47	[117.85]
C-CH	7	113.82	114.24	0.42	[115.49]
C-CH	4	110.49	109.97	-0.52	[111.11]
C-CH3	11	11.51	11.87	0.36	[10.50]

13C chem shifts: CRMSD=0.67ppm (CMAE=0.56) N=9 {-0.99 1.10}

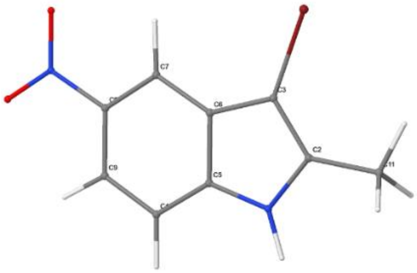

Conformer 1
Energy: -3178.44650 Hartree (Rel: 0.0 kcal/mol)
XYZ coordinates for conf 1:

N	1.05654	2.30183	0.00006
C	2.04542	1.32745	-0.00008
C	1.41795	0.10345	0.00002
C	-1.46111	2.30968	0.00009
C	-0.18592	1.72691	0.00005
C	-0.00002	0.31050	0.00011
C	-1.11109	-0.53235	0.00025
C	-2.36924	0.06572	0.00028
C	-2.55846	1.46465	0.00018
H	1.24007	3.29623	-0.00070
C	3.49155	1.68911	-0.00015
Br	2.26989	-1.58210	0.00015
H	-1.58765	3.38727	0.00003
H	-1.01222	-1.61052	0.00033
N	-3.54449	-0.79382	0.00041
H	-3.56508	1.86135	0.00020
O	-4.66123	-0.26333	-0.00084
O	-3.37943	-2.01908	-0.00070
H	3.75016	2.28271	0.88478
H	3.74998	2.28308	-0.88487
H	4.10508	0.78582	-0.00040

Originally assigned (incorrect) structure of 200{3b} ($CDCl_3$)

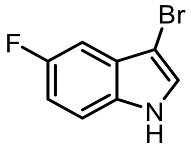

					Conf1
				Rel energy (kcal/mol):	0.00
**C-nom**	**iGau**	**Exp**	**Calc**	**diff**	**1**
C-C	8	134.41	158.40	23.99	[158.40]
C-C	5	131.62	130.86	-0.76	[130.86]
C-C	6	114.12	127.50	13.38	[127.50]
C-C	3	57.00	91.27	34.27	[91.27]
C-CH	2	129.28	126.53	-2.75	[126.53]
C-CH	4	126.13	112.60	-13.53	[112.60]
C-CH	9	123.69	111.69	-12.00	[111.69]
C-CH	7	112.83	103.95	-8.88	[103.95]

13C chem shifts: RMSD=17.11ppm (MAE=13.69) N=8 {-13.53 34.27}

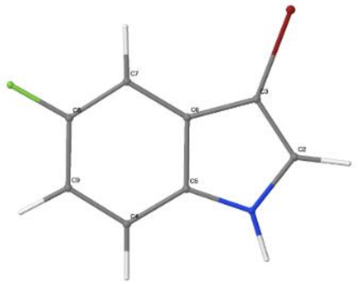

Conformer 1
Energy: -3033.84815 Hartree (Rel: 0.0 kcal/mol)
XYZ coordinates for conf 1:

N	-0.17322	2.40165	0.00000
C	1.10120	1.88184	-0.00003
C	1.01691	0.51200	-0.00000
C	-2.49270	1.39932	0.00001
C	-1.09331	1.37122	0.00000
C	-0.36685	0.14537	-0.00001
C	-1.06048	-1.07678	-0.00002
C	-2.44058	-1.01906	-0.00001
C	-3.16690	0.18422	0.00001
H	-0.39362	3.38650	0.00001
H	1.97033	2.52244	-0.00004
Br	2.47953	-0.68416	0.00001
H	-3.03865	2.33808	0.00002
H	-0.54488	-2.03108	-0.00002
F	-3.14414	-2.17933	-0.00000
H	-4.25080	0.14341	0.00002

Revised structure of 200{3b}, i.e 207{4d} ($CDCl_3$)

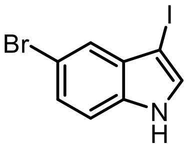

	Conf1
Rel energy (kcal/mol):	0.00

C-nom	iGau	Exp	Calc	diff	1
C-C	5	134.41	134.85	0.44	[134.85]
C-C	6	131.62	130.17	-1.45	[130.17]
C-C	8	114.12	114.88	0.76	[114.88]
C-C	3	57.00	55.10	-1.90	[55.10]
C-CH	2	129.28	130.38	1.10	[130.38]
C-CH	9	126.13	125.19	-0.94	[125.19]
C-CH	7	123.69	121.98	-1.71	[121.98]
C-CH	4	112.83	112.07	-0.76	[112.07]

13C chem shifts: RMSD=1.23ppm (MAE=1.13) N=8 {-1.90 1.10}

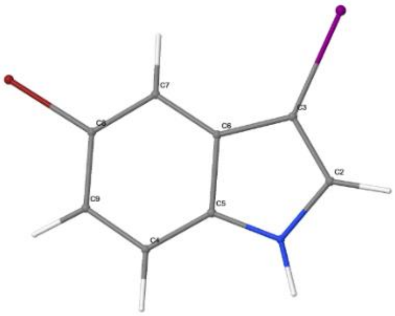

Conformer 1
Energy: -9853.87780 Hartree (Rel: 0.0 kcal/mol)
XYZ coordinates for conf 1:

N	-0.86531	2.90054	-0.00000
C	-1.96013	2.06665	0.00000
C	-1.52630	0.76392	0.00000
C	1.63275	2.53986	-0.00000
C	0.28982	2.14681	-0.00000
C	-0.09167	0.77378	0.00000
C	0.90033	-0.22066	0.00000
C	2.22412	0.18818	0.00000
C	2.60265	1.54514	-0.00000
H	-0.90906	3.90937	0.00000
H	-2.96372	2.46513	0.00000
I	-2.77379	-0.93667	-0.00000
H	1.91594	3.58828	-0.00000
H	0.63750	-1.27272	0.00000
Br	3.60871	-1.14237	0.00000
H	3.65361	1.81037	-0.00000

Originally assigned (incorrect) structure of 201{4d} ($CDCl_3$)

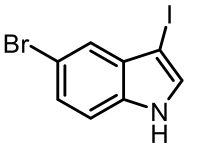

					Conf1
		Rel energy (kcal/mol):			0.00

C-nom	iGau	Exp	Calc	diff	1
C	5	131.46	134.85	3.39	[134.85]
C	2	130.97	130.38	-0.59	[130.38]
C	6	128.87	130.17	1.30	[130.17]
C	9	125.94	125.19	-0.75	[125.19]
C	7	122.59	121.98	-0.61	[121.98]
C	8	114.09	114.88	0.79	[114.88]
C	4	112.34	112.07	-0.27	[112.07]
C	3	103.50	55.10	-48.40	[55.10]

13C chem shifts: RMSD=17.17ppm (MAE=7.01) N=8 {-48.40 3.39}

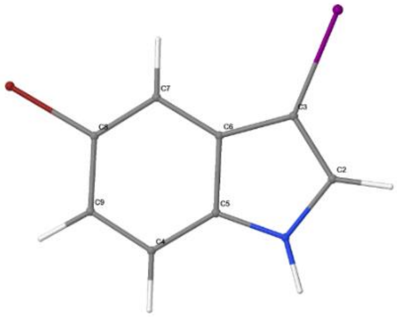

Conformer 1
Energy: -9853.87780 Hartree (Rel: 0.0 kcal/mol)
XYZ coordinates for conf 1:

N	-0.86531	2.90054	-0.00000
C	-1.96013	2.06665	0.00000
C	-1.52630	0.76392	0.00000
C	1.63275	2.53986	-0.00000
C	0.28982	2.14681	-0.00000
C	-0.09167	0.77378	0.00000
C	0.90033	-0.22066	0.00000
C	2.22412	0.18818	0.00000
C	2.60265	1.54514	-0.00000
H	-0.90906	3.90937	0.00000
H	-2.96372	2.46513	0.00000
I	-2.77379	-0.93667	-0.00000
H	1.91594	3.58828	-0.00000
H	0.63750	-1.27272	0.00000
Br	3.60871	-1.14237	0.00000
H	3.65361	1.81037	-0.00000

Revised structure of 197{3c}, i.e 209{3c-rev}, failed bromination? ($CDCl_3$)

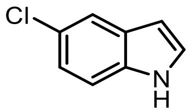

					Conf1
	Rel energy (kcal/mol):				0.00

C-nom	iGau	Exp	Calc	diff	1
C-C	5	134.17	134.24	0.07	[134.24]
C-C	6	128.99	128.39	-0.60	[128.39]
C-C	8	125.49	126.07	0.58	[126.07]
C-CH	2	125.62	126.53	0.91	[126.53]
C-CH	9	122.35	120.95	-1.40	[120.95]
C-CH	7	120.15	119.22	-0.93	[119.22]
C-CH	4	112.07	111.12	-0.95	[111.12]
C-CH	3	102.43	100.88	-1.55	[100.88]

13C chem shifts: RMSD=0.98ppm (MAE=0.87) N=8 {-1.55 0.91}

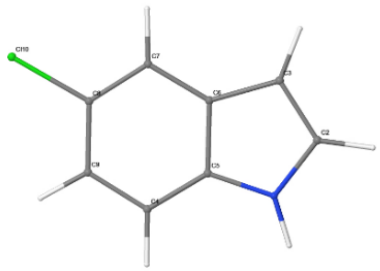

Conformer 1
Energy: -823.40809 Hartree (Rel: 0.0 kcal/mol)
XYZ coordinates for conf 1:

N	-2.61973	0.72809	-0.00000
C	-3.11695	-0.55983	0.00001
C	-2.07937	-1.45737	-0.00001
C	-0.29676	1.71553	-0.00000
C	-1.24214	0.68423	-0.00000
C	-0.86450	-0.69122	-0.00000
C	0.50173	-1.02598	0.00000
C	1.42424	0.00864	0.00000
C	1.04873	1.36698	-0.00000
Cl	3.15149	-0.38104	0.00000
H	-4.18432	-0.73123	0.00001
H	-2.16911	-2.53477	-0.00001
H	-0.59618	2.75971	-0.00000
H	0.82788	-2.06094	0.00000
H	1.81428	2.13502	-0.00000
H	-3.17973	1.56743	-0.00000

Revised structure of 198{3g}, i.e 210{3g-rev}, failed bromination? ($CDCl_3$)

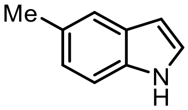

					Conf1
	Rel energy (kcal/mol):				0.00
**C-nom**	**iGau**	**Exp**	**Calc**	**diff**	**1**
C-C	5	134.15	133.78	-0.37	[133.78]
C-C	8	129.03	128.63	-0.40	[128.63]
C-C	6	128.18	127.47	-0.71	[127.47]
C-CH	2	124.27	124.28	0.01	[124.28]
C-CH	9	123.65	122.38	-1.27	[122.38]
C-CH	7	120.38	120.28	-0.10	[120.28]
C-CH	4	110.70	110.38	-0.32	[110.38]
C-CH	3	102.13	100.40	-1.73	[100.40]
C-CH3	10	21.48	20.80	-0.68	[20.80]

13C chem shifts: RMSD=0.82ppm (MAE=0.62) N=9 {-1.73 0.01}

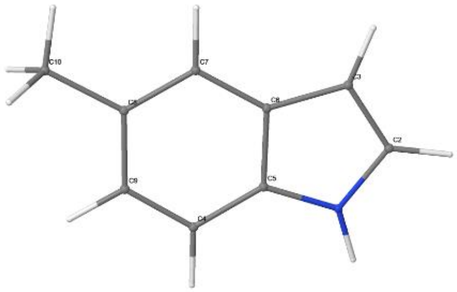

Conformer 1
Energy: -403.13061 Hartree (Rel: 0.0 kcal/mol)
XYZ coordinates for conf 1:

N	-2.21121	0.81740	0.00004
C	-2.78440	-0.43907	0.00002
C	-1.79929	-1.39466	0.00000
C	0.17347	1.66026	-0.00001
C	-0.83614	0.69007	0.00003
C	-0.53979	-0.70185	-0.00002
C	0.80845	-1.10903	-0.00008
C	1.82596	-0.15882	-0.00008
C	1.49078	1.21922	-0.00006
C	3.28094	-0.57539	0.00010
H	-2.71993	1.68854	0.00009
H	-3.86017	-0.54730	0.00003
H	-1.95314	-2.46528	-0.00001
H	-0.06185	2.72138	-0.00002
H	1.05661	-2.16841	-0.00013
H	2.29322	1.95368	-0.00011
H	3.38204	-1.66559	-0.00205
H	3.81152	-0.18977	-0.88014
H	3.81027	-0.19343	0.88270

Revised structure of 199{3h}, i.e 211{3h-rev}, failed bromination? (CDCl3)

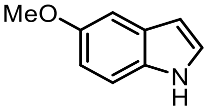

C-nom	iGau	Exp	Calc	diff	1	2
				Rel energy (kcal/mol):	Conf1 0.00	Conf2 0.84
C-C	8	154.24	153.13	-1.11	[153.23	152.72]
C-C	5	131.03	130.99	-0.04	[130.83	131.65]
C-C	6	128.34	128.49	0.15	[128.36	129.04]
C-CH	2	124.95	125.03	0.08	[124.79	125.99]
C-CH	9	112.39	111.35	-1.04	[112.78	105.47]
C-CH	4	111.78	111.45	-0.33	[111.66	110.59]
C-CH	3	102.40	100.38	-2.02	[100.49	99.91]
C-CH	7	102.40	101.08	-1.32	[99.83	106.23]
C-CH3	17	55.54	55.12	-0.42	[55.11	55.18]

13C chem shifts: RMSD=0.97ppm (MAE=0.72) N=9 {-2.02 0.15}

Fractions: 0.804 0.196

NOTICE:
unreacted 5{methoxyindole} starting material isolated as 3{bromo} 3h (assuming overlap at 102.4ppm)

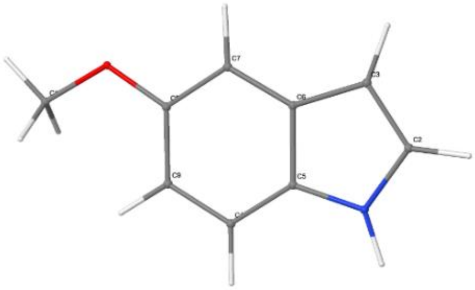

Conformer 1
Energy: -478.33443 Hartree (Rel: 0.0 kcal/mol)
XYZ coordinates for conf 1:

N	2.69926	0.52435	0.00032
C	3.02985	-0.81404	0.00016
C	1.88378	-1.57226	-0.00021
C	0.50776	1.79106	0.00017
C	1.32266	0.65166	0.00010
C	0.77468	-0.65992	-0.00022
C	-0.62692	-0.82810	-0.00051
C	-1.43252	0.30628	-0.00041
C	-0.86595	1.60575	-0.00006
H	3.35940	1.28696	0.00059
H	4.06672	-1.12034	0.00029
H	1.83748	-2.65292	-0.00043
H	0.93225	2.79141	0.00044
H	-1.05205	-1.82540	-0.00086
O	-2.80592	0.28537	-0.00078
H	-1.54151	2.45547	0.00003
C	-3.45016	-0.97879	0.00095
H	-4.52215	-0.77228	0.00178
H	-3.19246	-1.56228	0.89489
H	-3.19425	-1.56388	-0.89248

Conformer 2
Energy: -478.33309 Hartree (Rel: 0.8 kcal/mol)
XYZ coordinates for conf 2:

N	2.59158	0.89008	0.00026
C	3.21807	-0.33888	0.00049
C	2.27871	-1.34000	0.00025
C	0.17031	1.61787	-0.00034
C	1.22126	0.70018	-0.00007

C	0.98799	-0.70645	-0.00009
C	-0.33356	-1.17921	-0.00032
C	-1.38470	-0.26281	-0.00061
C	-1.13512	1.12895	-0.00064
H	3.06069	1.78290	0.00036
H	4.29764	-0.39982	0.00077
H	2.48194	-2.40224	0.00032
H	0.35414	2.68897	-0.00034
H	-0.55939	-2.24152	-0.00028
O	-2.64713	-0.80831	-0.00113
H	-1.95782	1.83398	-0.00094
C	-3.76229	0.06892	0.00148
H	-4.64732	-0.57046	0.00242
H	-3.78053	0.70711	-0.89204
H	-3.77744	0.70562	0.89611

Revised structure of 204{4j}, i.e 212{4j-rev} ($CDCl_3$)

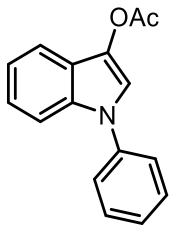

					Conf1
	Rel energy (kcal/mol):				0.00
**C-nom**	**iGau**	**Exp**	**Calc**	**diff**	**1**
C-C	18	168.49	167.12	-1.37	[167.12]
C-C	10	139.44	139.11	-0.33	[139.11]
C-C	3	132.89	133.03	0.14	[133.03]
C-C	5	131.54	130.53	-1.01	[130.53]
C-C	6	122.00	121.65	-0.35	[121.65]
C-CH	12	129.65	129.06	-0.59	[129.06]
C-CH	14	129.65	129.06	-0.59	[129.06]
C-CH	13	126.51	126.01	-0.50	[126.01]
C-CH	11	124.45	124.23	-0.22	[124.23]
C-CH	15	124.45	124.23	-0.22	[124.23]
C-CH	9	123.27	123.01	-0.26	[123.01]
C-CH	8	120.44	120.39	-0.05	[120.39]
C-CH	2	117.85	118.88	1.03	[118.88]
C-CH	7	117.05	118.08	1.03	[118.08]
C-CH	4	110.61	109.96	-0.65	[109.96]
C-CH3	19	21.00	20.58	-0.42	[20.58]

13C chem shifts: RMSD=0.66ppm (MAE=0.55) N=16 {-1.37 1.03}

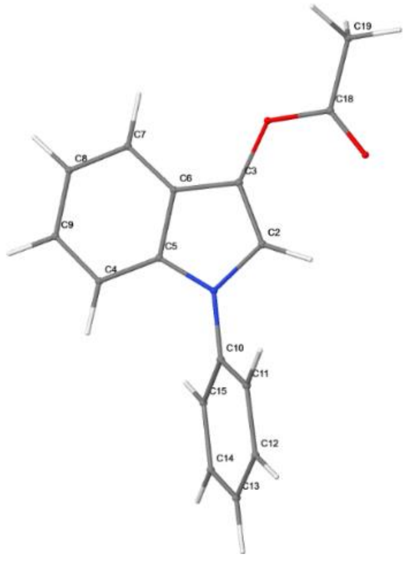

Conformer 1
Energy: -822.73333 Hartree (Rel: 0.0 kcal/mol)
XYZ coordinates for conf 1:

N	-0.64475	-0.04553	-0.02022
C	0.45277	-0.90550	-0.06005
C	1.59964	-0.15433	-0.03473
C	-0.87970	2.47850	0.15774
C	-0.18464	1.26650	0.04449
C	1.23589	1.23013	0.03304
C	1.97016	2.42566	0.10216
C	1.27874	3.62664	0.19426
C	-0.13191	3.64927	0.22839

C	-1.99626	-0.48453	-0.01716
C	-2.37839	-1.54946	0.80959
C	-3.69819	-2.00098	0.79565
C	-4.64747	-1.38542	-0.02396
C	-4.26450	-0.32065	-0.84319
C	-2.94229	0.12500	-0.85226
O	2.93344	-0.51584	-0.07571
O	2.55170	-2.75370	-0.12317
C	3.32288	-1.81898	-0.11527
C	4.82722	-1.91008	-0.14855
H	0.31489	-1.96910	-0.13602
H	-1.96318	2.50665	0.19734
H	3.05591	2.40340	0.09014
H	1.82834	4.56225	0.24797
H	-0.64795	4.60147	0.31440
H	-1.64631	-2.00836	1.46691
H	-3.98586	-2.82800	1.43866
H	-5.67615	-1.73386	-0.02582
H	-4.99227	0.15736	-1.49286
H	-2.63741	0.92598	-1.51748
H	5.12672	-2.95822	-0.17020
H	5.21633	-1.39549	-1.03328
H	5.25529	-1.41967	0.73175

Revised structure of 205{4k}, i.e 213{4k-rev} ($CDCl_3$)

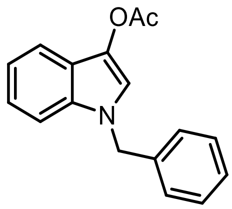

					**Conf1**	**Conf2**	**Conf3**
Rel energy (kcal/mol):					0.00	0.37	1.98
**C-nom**	**iGau**	**Exp**	**Calc**	**diff**	**1**	**2**	**3**
C-C	18	168.58	166.99	-1.59	[167.04	166.82	168.27]
C-C	10	137.25	137.61	0.36	[137.38	138.02	137.75]
C-C	3	133.39	131.03	-2.36	[130.59	131.87	130.60]
C-C	5	129.80	131.06	1.26	[130.27	132.30	134.46]
C-C	6	120.45	120.59	0.14	[121.01	119.71	122.07]
C-CH	12	128.81	127.67	-1.14	[127.72	127.58	127.68]
C-CH	14	128.81	127.67	-1.14	[127.72	127.58	127.68]
C-CH	13	127.73	126.64	-1.09	[126.62	126.68	126.52]
C-CH	11	126.87	125.88	-0.99	[125.69	126.24	125.56]
C-CH	15	126.87	125.88	-0.99	[125.69	126.24	125.56]
C-CH	9	122.59	122.16	-0.43	[122.00	122.45	122.24]
C-CH	8	119.61	119.34	-0.27	[119.36	119.27	119.77]
C-CH	2	117.70	120.05	2.35	[120.81	118.59	120.83]
C-CH	7	117.30	117.76	0.46	[117.69	117.91	117.61]
C-CH	4	109.74	109.49	-0.25	[110.23	108.17	108.69]
C-CH2	17	50.15	51.19	1.04	[52.00	49.80	49.48]
C-CH3	20	21.00	20.59	-0.41	[20.64	20.55	19.78]

13C chem shifts: RMSD=1.16ppm (MAE=0.96) N=17 {-2.36 2.35}

Fractions: 0.635 0.342 0.022

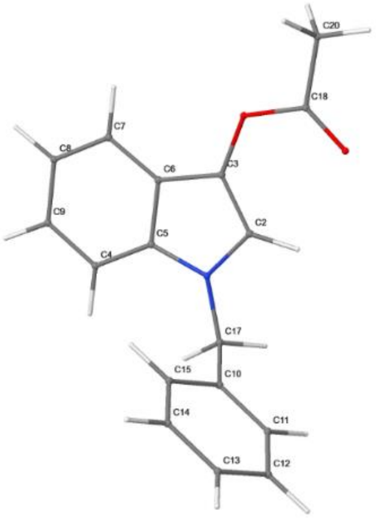

Conformer 1

Energy: -862.04437 Hartree (Rel: 0.0 kcal/mol)

XYZ coordinates for conf 1:

N	0.16031	-0.10625	0.90472
C	-0.98419	-0.86858	0.71212
C	-1.94658	-0.07725	0.13138
C	0.77126	2.29777	0.42154
C	-0.06937	1.17623	0.44368
C	-1.40109	1.23398	-0.05319
C	-1.89859	2.43985	-0.57610
C	-1.06574	3.55153	-0.59303
C	0.25532	3.47966	-0.09901
C	2.55071	-0.75263	0.51523

C	2.34285	-1.25655	-0.77517
C	3.41856	-1.43754	-1.64565
C	4.71690	-1.12042	-1.23612
C	4.93196	-0.61741	0.04838
C	3.85301	-0.43184	0.91636
O	-3.24795	-0.34891	-0.25549
C	1.39840	-0.59360	1.49685
C	-3.80328	-1.57765	-0.09040
O	-3.22199	-2.52245	0.39926
C	-5.22206	-1.58005	-0.60136
H	-1.01681	-1.90197	1.01063
H	1.79177	2.24590	0.78780
H	-2.91408	2.49598	-0.95750
H	-1.43280	4.49232	-0.99401
H	0.88419	4.36518	-0.12973
H	1.33534	-1.50037	-1.10148
H	3.24268	-1.82613	-2.64518
H	5.55337	-1.26067	-1.91531
H	5.93668	-0.36143	0.37393
H	4.02539	-0.03362	1.91405
H	1.69474	0.08230	2.30757
H	1.16421	-1.55684	1.96340
H	-5.65860	-2.56851	-0.45565
H	-5.24043	-1.32055	-1.66498
H	-5.81671	-0.82995	-0.06989

Conformer 2
Energy: -862.04121 Hartree (Rel: 0.4 kcal/mol)
XYZ coordinates for conf 2:

N	0.21948	0.41272	0.89729
C	-0.53887	-0.73155	0.69302
C	-1.73744	-0.36878	0.12774
C	-0.15816	2.87504	0.43507
C	-0.49148	1.51247	0.45194
C	-1.74567	1.05302	-0.04061
C	-2.67807	1.97304	-0.54941
C	-2.34595	3.32156	-0.56102
C	-1.09743	3.76568	-0.07342
C	2.63999	-0.09178	0.49758
C	3.61483	-0.98754	0.95106
C	4.63046	-1.42806	0.09816
C	4.67628	-0.98013	-1.22295
C	3.70265	-0.08937	-1.68530
C	2.69264	0.35311	-0.83060
O	-2.83438	-1.11837	-0.26033
C	1.56751	0.40806	1.45369
C	-2.87812	-2.46653	-0.09343
O	-1.98188	-3.11662	0.40013
C	-4.18596	-3.01146	-0.60969
H	-0.16164	-1.70118	0.96747
H	0.79974	3.23127	0.80216
H	-3.63771	1.63001	-0.92526
H	-3.05374	4.04756	-0.95137
H	-0.86278	4.82630	-0.09702
H	3.57908	-1.34540	1.97769
H	5.37830	-2.12600	0.46497
H	5.46175	-1.32465	-1.89005
H	3.73006	0.26141	-2.71352
H	1.93708	1.04244	-1.19821
H	1.78691	1.43181	1.77375
H	1.56633	-0.20671	2.36027
H	-4.21649	-4.09010	-0.45335
H	-4.29120	-2.78959	-1.67684
H	-5.02526	-2.53692	-0.09108

Conformer 3
Energy: -862.04378 Hartree (Rel: 2.0 kcal/mol)
XYZ coordinates for conf 3:

N	-0.33872	0.55168	-1.01023
C	0.36778	-0.62432	-1.19468
C	1.63776	-0.46540	-0.71012

C	0.24496	2.79472	0.01127
C	0.48995	1.47339	-0.38975
C	1.76094	0.86146	-0.18870
C	2.80000	1.59318	0.41151
C	2.55534	2.90287	0.80249
C	1.28903	3.49539	0.60489
C	-2.73372	0.04614	-0.46843
C	-3.91005	-0.48483	-1.01131
C	-4.86527	-1.08731	-0.18938
C	-4.64887	-1.17224	1.18755
C	-3.47377	-0.65069	1.73548
C	-2.52282	-0.04367	0.91337
O	2.65602	-1.40644	-0.80913
C	-1.73471	0.74192	-1.38180
C	2.94260	-2.16385	0.29893
O	2.34455	-2.07400	1.34415
C	4.09136	-3.09387	0.00238
H	-0.09241	-1.48308	-1.66188
H	-0.72496	3.26205	-0.13014
H	3.77491	1.13888	0.56487
H	3.34683	3.48242	1.26945
H	1.12454	4.52062	0.92466
H	-4.07975	-0.42907	-2.08452
H	-5.77191	-1.49717	-0.62645
H	-5.38729	-1.64586	1.82871
H	-3.29517	-0.71733	2.80531
H	-1.60952	0.35460	1.34649
H	-1.91967	1.82106	-1.39632
H	-1.87120	0.39337	-2.41148
H	4.29849	-3.71020	0.87754
H	4.98209	-2.51488	-0.26292
H	3.84840	-3.73095	-0.85408

Correct structure of the starting material, 214{1} ($CDCl_3$)

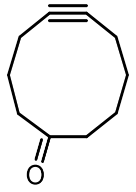

	Conf1	Conf2	Conf3
Rel energy (kcal/mol):	0.00	0.95	1.67

C-nom	iGau	Exp	Calc	diff	1	2	3
C	2	210.74	209.81	-0.93	[210.03	208.44	210.88]
C	6	85.68	86.05	0.37	[86.08	86.61	83.57]
C	7	83.34	83.26	-0.08	[83.44	82.94	81.28]
C	1	42.66	44.68	2.02	[45.93	38.69	44.15]
C	10	42.58	42.02	-0.56	[41.79	43.81	39.81]
C	9	26.86	28.07	1.21	[28.71	26.85	21.46]
C	4	25.64	27.62	1.98	[27.77	28.28	22.96]
C	3	21.74	21.14	-0.60	[21.02	21.03	23.49]
C	8	19.28	18.56	-0.72	[18.64	18.29	18.12]
C	5	18.18	17.98	-0.20	[17.92	17.91	19.24]

13C chem shifts: RMSD=1.08ppm (MAE=0.87) N=10 {-0.93 2.02}

Fractions: 0.792 0.161 0.047

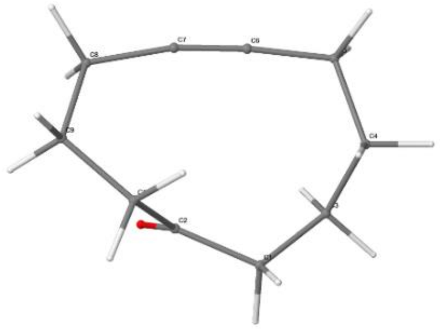

Conformer 1
Energy: -464.65492 Hartree (Rel: 0.0 kcal/mol)
XYZ coordinates for conf 1:

C	0.79499	-1.39525	-0.74391
C	-0.41115	-1.26333	0.18250
C	2.15332	-1.01546	-0.12369
C	2.61096	0.43739	-0.35695
C	1.87591	1.53550	0.44817
C	0.44612	1.63983	0.14708
C	-0.74195	1.64286	-0.08998
C	-2.17177	1.43455	-0.33116
C	-2.62502	0.03262	0.14210
C	-1.79245	-1.11138	-0.46612
O	-0.30916	-1.37597	1.39396
H	0.61188	-0.83930	-1.66989
H	0.80833	-2.45794	-1.03610
H	2.12018	-1.23404	0.95058
H	2.92272	-1.66914	-0.55142
H	2.54189	0.67387	-1.42725
H	3.67360	0.50836	-0.09179
H	2.00130	1.33692	1.52200
H	2.36928	2.49831	0.25721
H	-2.76709	2.20094	0.18252
H	-2.38913	1.54495	-1.40305
H	-3.68019	-0.09841	-0.12447
H	-2.55199	-0.02966	1.23320
H	-2.31742	-2.06013	-0.28947
H	-1.70375	-0.99096	-1.55193

Conformer 2
Energy: -464.65758 Hartree (Rel: 0.9 kcal/mol)
XYZ coordinates for conf 2:

C	0.55818	-1.31427	-0.78989
C	-0.42958	-1.24952	0.37576
C	2.02781	-1.03594	-0.41745
C	2.50837	0.42216	-0.54659
C	1.93744	1.43357	0.47681
C	0.49360	1.62863	0.33578
C	-0.70689	1.67209	0.18212
C	-2.13768	1.47132	-0.05481
C	-2.38257	0.11583	-0.75875
C	-1.92114	-1.10942	0.05525
O	-0.06540	-1.45037	1.52408
H	0.22515	-0.67314	-1.61255
H	0.47203	-2.34605	-1.16708
H	2.19657	-1.39495	0.60465
H	2.66630	-1.64266	-1.07088
H	2.29883	0.78950	-1.56009
H	3.60009	0.42958	-0.43248
H	2.15985	1.08482	1.49501
H	2.46224	2.39119	0.35840
H	-2.68642	1.49707	0.89694
H	-2.55137	2.27964	-0.67195
H	-1.89207	0.12987	-1.73886
H	-3.45763	0.01176	-0.94938
H	-2.45092	-1.14835	1.01337
H	-2.20473	-2.01206	-0.50718

Conformer 3
Energy: -464.65608 Hartree (Rel: 1.7 kcal/mol)
XYZ coordinates for conf 3:

C	0.95198	-1.82566	-0.41797
C	-0.32555	-1.37629	0.28054
C	1.87068	-0.65527	-0.85596
C	2.36787	0.24247	0.30695
C	1.90322	1.71703	0.19754
C	0.44491	1.80258	0.08349
C	-0.75596	1.71497	-0.04799
C	-2.17681	1.42772	-0.25562
C	-2.52078	-0.05434	0.04463
C	-1.53989	-1.05073	-0.59235
O	-0.39137	-1.33634	1.49968
H	0.69432	-2.41979	-1.30430
H	1.49637	-2.46991	0.28103
H	2.72629	-1.09901	-1.37746
H	1.34233	-0.04659	-1.59916
H	3.46334	0.23712	0.34631
H	2.01636	-0.15890	1.26356
H	2.26001	2.27821	1.07122
H	2.37101	2.18518	-0.68015
H	-2.80553	2.07901	0.36550
H	-2.43981	1.65419	-1.29881
H	-3.53825	-0.24433	-0.31476
H	-2.51831	-0.22016	1.12654
H	-2.04267	-2.01775	-0.75042
H	-1.21252	-0.72144	-1.58607

Originally assigned (incorrect) structure of 216{21} as *cis*-isomer ($CDCl_3$)

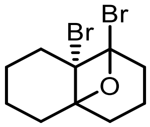

					Conf1	Conf2
Rel energy (kcal/mol):					0.00	0.24

**C-nom**	**iGau**	**Exp**	**Calc**	**diff**	**1**	**2**
C-C	6	75.78	109.14	33.36	[110.34	107.35]
C-C	5	69.54	90.42	20.88	[89.85	91.27]
C-C	4	67.90	90.84	22.94	[91.04	90.54]
C-CH2	1	42.58	38.84	-3.74	[38.95	38.68]
C-CH2	7	32.25	33.65	1.40	[32.76	34.97]
C-CH2	10	28.21	32.89	4.68	[33.56	31.88]
C-CH2	3	27.08	30.85	3.77	[30.97	30.66]
C-CH2	9	21.83	21.65	-0.18	[21.91	21.26]
C-CH2	8	20.09	20.64	0.55	[20.98	20.12]
C-CH2	2	19.43	17.89	-1.54	[17.85	17.95]

13C chem shifts: RMSD=14.59ppm (MAE=9.30) N=10 {-3.74 33.36}

Fractions: 0.599 0.401

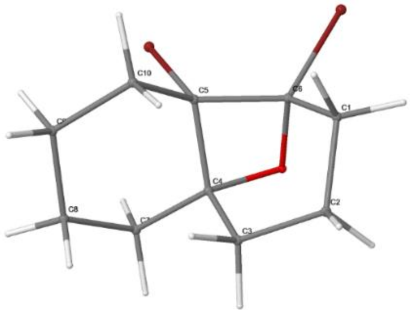

Conformer 1
Energy: -5607.47618 Hartree (Rel: 0.0 kcal/mol)
XYZ coordinates for conf 1:

C	-1.00913	2.17563	0.52526
C	0.34684	2.91708	0.52577
C	1.34692	2.27776	-0.45803
C	1.03906	0.78849	-0.77128
C	0.45094	0.00157	0.41169
C	-0.83937	0.76233	-0.02915
C	2.23875	0.09931	-1.39247
C	3.27446	-0.06533	-0.23060
C	2.73826	-0.57122	1.14458
C	1.36661	0.00354	1.62143
O	-0.31721	0.82821	-1.37517
Br	-2.61966	-0.11223	0.02924
Br	0.17106	-1.95163	-0.07417
H	-1.73153	2.67510	-0.12701
H	-1.44314	2.13937	1.52881
H	0.76438	2.90293	1.53709
H	0.19488	3.97218	0.27820
H	2.36808	2.41453	-0.09479
H	1.29144	2.76779	-1.43717
H	2.68231	0.69993	-2.19501
H	1.95400	-0.86788	-1.81070
H	3.77604	0.89667	-0.08029
H	4.05662	-0.75776	-0.56149
H	3.49221	-0.34675	1.90789
H	2.64607	-1.65952	1.11013
H	1.49121	1.02921	1.98294
H	0.97603	-0.59139	2.45185

Conformer 2
Energy: -5607.47656 Hartree (Rel: 0.2 kcal/mol)
XYZ coordinates for conf 2:

C	-0.97610	2.10302	0.72054
C	0.06891	3.05237	0.10654
C	1.35136	2.29001	-0.26402
C	1.03219	0.83121	-0.71170
C	0.44871	-0.02912	0.42447
C	-0.84269	0.73669	0.02512
C	2.24729	0.18779	-1.35093
C	3.27727	0.02857	-0.18283
C	2.74259	-0.54780	1.16761
C	1.33825	-0.05807	1.65063
O	-0.32098	0.88451	-1.32073
Br	-2.60502	-0.15934	0.04077
Br	0.21597	-1.96197	-0.15134
H	-1.98181	2.50214	0.57525
H	-0.83644	1.96480	1.79882
H	0.28741	3.88022	0.78868
H	-0.35932	3.48652	-0.80054
H	2.06305	2.29628	0.56415
H	1.85288	2.78496	-1.10371
H	2.67831	0.81696	-2.13755
H	1.99229	-0.77846	-1.78932
H	3.73675	1.00541	0.00168
H	4.08983	-0.62231	-0.52467
H	3.47801	-0.32493	1.94911
H	2.69556	-1.63695	1.08769
H	1.40270	0.94774	2.08034
H	0.95864	-0.72054	2.43352

Originally assigned (incorrect) structure of 216{21} as *trans*-isomer ($CDCl_3$)

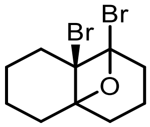

					Conf1	Conf2
		Rel energy (kcal/mol):			0.00	0.14

C-nom	iGau	Exp	Calc	diff	1	2
C-C	6	75.78	107.52	31.74	[106.39	108.95]
C-C	4	69.54	93.50	23.96	[93.10	94.01]
C-C	5	67.90	64.36	-3.54	[66.53	61.62]
C-CH2	10	42.58	40.66	-1.92	[40.55	40.79]
C-CH2	1	32.25	37.46	5.21	[37.59	37.29]
C-CH2	3	28.21	30.31	2.10	[30.27	30.35]
C-CH2	7	27.08	29.93	2.85	[29.93	29.94]
C-CH2	8	21.83	20.87	-0.96	[20.94	20.78]
C-CH2	9	20.09	19.52	-0.57	[19.51	19.53]
C-CH2	2	19.43	17.81	-1.62	[17.94	17.65]

13C chem shifts: RMSD=12.81ppm (MAE=7.45) N=10 {-3.54 31.74}

Fractions: 0.558 0.442

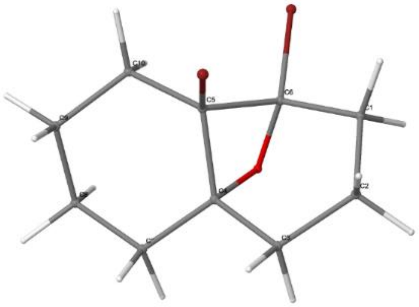

Conformer 1
Energy: -5607.50766 Hartree (Rel: 0.0 kcal/mol)
XYZ coordinates for conf 1:

C	-1.14112	-1.48909	1.09966
C	-0.08638	-1.69843	2.21787
C	1.21901	-0.90823	1.97308
C	0.94444	0.36327	1.15609
C	0.41035	0.07589	-0.28560
C	-0.91749	-0.11383	0.47692
C	2.00797	1.43448	1.34378
C	1.96243	2.58712	0.33246
C	1.80219	2.05601	-1.09730
C	0.47759	1.29574	-1.21862
O	-0.44287	0.81567	1.46448
Br	-2.58920	0.51423	-0.40270
Br	1.16503	-1.50037	-1.24573
H	-2.14594	-1.53938	1.52552
H	-1.07664	-2.24808	0.31503
H	0.12441	-2.76504	2.34158
H	-0.52489	-1.34961	3.15684
H	1.96482	-1.50882	1.44310
H	1.66355	-0.62212	2.93318
H	2.97782	0.92370	1.26760
H	1.93362	1.80591	2.37360
H	2.87712	3.18356	0.42745
H	1.12212	3.25421	0.56455
H	2.64570	1.40262	-1.35965
H	1.80507	2.88398	-1.81536
H	0.28262	0.97622	-2.24535
H	-0.33419	1.97476	-0.93892

Conformer 2
Energy: -5607.50788 Hartree (Rel: 0.1 kcal/mol)
XYZ coordinates for conf 2:

C	-1.12488	-1.46991	1.17214
C	0.17841	-1.92523	1.89268
C	1.23765	-0.79960	2.02897
C	0.93661	0.41990	1.15196
C	0.40098	0.08879	-0.27798
C	-0.91442	-0.12408	0.49933
C	1.96281	1.53292	1.31363
C	1.85888	2.66759	0.28629
C	1.71346	2.10684	-1.13350
C	0.41958	1.29267	-1.23422
O	-0.46002	0.82902	1.46854
Br	-2.61929	0.44825	-0.36461
Br	1.20078	-1.47744	-1.21564
H	-1.94204	-1.33691	1.88699
H	-1.44600	-2.21288	0.43694
H	0.61245	-2.75687	1.33487
H	-0.07810	-2.31165	2.88345
H	2.23386	-1.18539	1.78830
H	1.27959	-0.42887	3.05980
H	2.95161	1.06246	1.23042
H	1.88576	1.91805	2.33814
H	2.74719	3.30470	0.36599
H	0.99157	3.30067	0.51414
H	2.58147	1.48374	-1.38953
H	1.67891	2.92178	-1.86553
H	0.23386	0.94563	-2.25371
H	-0.41674	1.94576	-0.96557

Revised structure of 216{21}, i.e 217{21-rev} ($CDCl_3$)

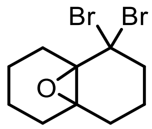

						Conf1	Conf2	Conf3	Conf4
Rel energy (kcal/mol):						0.00	1.17	2.99	3.85
C-nom	iGau	Exp	Calc	diff		1	2	3	4]
CBr2	6	75.78	73.86	-1.92	[	73.99	73.09	71.22	71.91]
C-O	4	69.54	70.84	1.30	[	70.92	70.25	70.55	71.77]
C-O	5	67.90	68.02	0.12	[	67.84	69.34	68.18	67.52]
CH2	1	42.58	42.60	0.02	[	42.43	43.36	50.58	51.59]
CH2	7	32.25	31.59	-0.66	[	31.65	31.31	28.68	30.28]
CH2	10	28.21	27.87	-0.34	[	28.05	26.50	29.07	28.68]
CH2	3	27.08	27.07	-0.01	[	26.77	29.00	30.97	30.56]
CH2	9	21.83	20.91	-0.92	[	21.19	18.98	19.32	21.05]
CH2	2	20.09	20.42	0.33	[	20.45	20.32	17.93	17.79]
CH2	8	19.43	18.98	-0.45	[	18.70	20.93	20.78	18.99]

13C chem shifts: RMSD=0.84ppm (MAE=0.61) N=10 {-1.92 1.30}

Fractions: 0.872 0.121 0.006 0.001

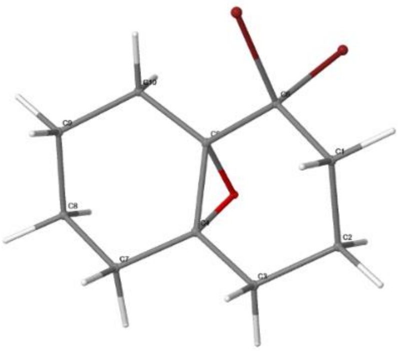

Conformer 1
Energy: -5607.50889 Hartree (Rel: 0.0 kcal/mol)
XYZ coordinates for conf 1:

C	-0.83701	1.40482	-1.22919
C	0.19104	2.50314	-0.93431
C	1.63654	1.99580	-1.05468
C	1.85505	0.55704	-0.59521
C	0.72615	-0.34550	-0.20359
C	-0.68240	0.23075	-0.26765
C	3.25422	0.25704	-0.07971
C	3.45143	-1.19867	0.35677
C	2.34337	-1.61363	1.32982
C	0.95774	-1.58022	0.66697
O	1.32324	-0.43157	-1.51136
Br	-1.21161	0.80421	1.57773
H	-0.69131	1.01219	-2.24105
H	-1.85736	1.79110	-1.16514
H	0.01562	2.89288	0.07408
H	0.02786	3.33607	-1.62678
H	2.30563	2.65650	-0.49219
H	1.96730	2.03616	-2.10083
H	3.44453	0.92550	0.77114
H	3.97474	0.53424	-0.86157
H	4.43569	-1.30458	0.82754
H	3.44299	-1.85515	-0.52216
H	2.35186	-0.93286	2.19279
H	2.52751	-2.62071	1.72173
H	0.17811	-1.64931	1.43038
H	0.83656	-2.45018	0.01062
Br	-1.95760	-1.20943	-0.83096

```
Conformer 2
Energy: -5607.50702 Hartree (Rel:  1.2 kcal/mol)
XYZ coordinates for conf 2:
C         -0.84885        1.34176       -1.27791
C          0.18172        2.45208       -1.05316
C          1.62415        1.93984       -1.17838
C          1.85997        0.53403       -0.62670
C          0.73295       -0.34447       -0.17048
C         -0.68366        0.21914       -0.25710
C          3.28805        0.24431       -0.15824
C          3.40242       -0.79655        0.96691
C          2.44597       -1.97260        0.75634
C          0.99025       -1.49267        0.79554
O          1.30695       -0.51289       -1.47372
Br        -1.23246        0.87864        1.55060
H         -0.71426        0.89750       -2.26960
H         -1.86867        1.73055       -1.22185
H          0.02620        2.88780       -0.06016
H          0.00556        3.25297       -1.77959
H          2.30063        2.64008       -0.67767
H          1.92759        1.91442       -2.23347
H          3.75720        1.18121        0.16090
H          3.84321       -0.09651       -1.04266
H          3.16870       -0.31910        1.92880
H          4.44002       -1.14426        1.03361
H          2.59145       -2.72884        1.53638
H          2.65105       -2.45762       -0.20561
H          0.73849       -1.16091        1.80884
H          0.30792       -2.31364        0.54991
Br        -1.95578       -1.24806       -0.76543

Conformer 3
Energy: -5607.50412 Hartree (Rel:  3.0 kcal/mol)
XYZ coordinates for conf 3:
C          0.80554        1.22555       -1.39822
C         -0.18080        2.38756       -1.25377
C         -1.61157        1.85448       -1.35691
C         -1.88409        0.74644       -0.35396
C         -0.77048       -0.09122        0.20015
C          0.66730        0.18024       -0.28537
C         -3.32466        0.24542       -0.31124
C         -3.49138       -1.20124        0.18291
C         -2.52548       -1.53181        1.32572
C         -1.07468       -1.44181        0.83586
O         -1.30137        1.01495        0.94476
Br         1.53581       -1.49062       -0.96667
H          1.84210        1.56865       -1.44050
H          0.59730        0.71519       -2.34647
H          0.00914        3.11177       -2.05334
H         -0.03337        2.90375       -0.30038
H         -1.80021        1.47039       -2.36868
H         -2.34078        2.65736       -1.18439
H         -3.76687        0.35371       -1.30891
H         -3.87124        0.93561        0.34578
H         -3.30113       -1.89421       -0.64856
H         -4.53073       -1.36230        0.49195
H         -2.71059       -2.54570        1.69890
H         -2.68146       -0.84415        2.16493
H         -0.89234       -2.23418        0.10359
H         -0.37072       -1.60212        1.66234
Br         1.73858        0.75933        1.31280

Conformer 4
Energy: -5607.50276 Hartree (Rel:  3.8 kcal/mol)
XYZ coordinates for conf 4:
C         -0.81985        1.23311        1.38693
C          0.19637        2.37357        1.30497
C          1.60911        1.79827        1.42694
C          1.88059        0.74077        0.36964
C          0.76650       -0.06507       -0.23552
```

C	-0.66828	0.19327	0.26597
C	3.29911	0.19640	0.34613
C	3.54127	-0.84850	-0.74939
C	2.46018	-1.93176	-0.70559
C	1.06310	-1.36191	-0.99526
O	1.31703	1.08264	-0.91250
Br	-1.50544	-1.48785	0.97172
H	-1.84948	1.59868	1.39588
H	-0.65962	0.70829	2.33580
H	0.00213	3.07418	2.12438
H	0.09003	2.92566	0.36659
H	1.75816	1.35997	2.42316
H	2.36150	2.58990	1.31492
H	3.50182	-0.24438	1.33236
H	3.99033	1.04285	0.23573
H	4.53534	-1.29047	-0.61463
H	3.53717	-0.36046	-1.73193
H	2.46306	-2.40363	0.28735
H	2.67747	-2.72455	-1.43104
H	0.30929	-2.11947	-0.77839
H	0.97420	-1.11939	-2.06124
Br	-1.75731	0.75450	-1.32566

Originally assigned (incorrect) structure of 221{10} ($CDCl_3$)

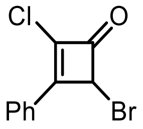

C-nom	iGau	Exp	Calc	diff	Conf1
				Rel energy (kcal/mol):	0.00
					1
C-C	4	178.90	177.72	-1.18	[177.72]
C-C	2	176.30	170.68	-5.62	[170.68]
C-C	7	126.30	124.79	-1.51	[124.79]
C-C	3	76.40	124.34	47.94	[124.34]
C-CH	10	134.10	134.50	0.40	[134.50]
C-CH	8	130.10	131.60	1.50	[131.60]
C-CH	12	130.10	131.60	1.50	[131.60]
C-CH	9	129.70	128.16	-1.54	[128.16]
C-CH	11	129.70	128.16	-1.54	[128.16]
C-CH	1	128.10	53.05	-75.05	[53.05]

13C chem shifts: RMSD=28.24ppm (MAE=13.78) N=10 {-75.05 47.94}

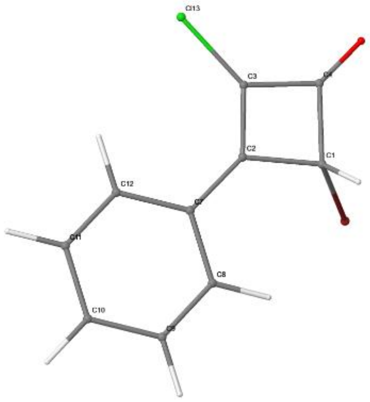

```
Conformer 1
Energy: -3491.45342 Hartree (Rel:  0.0 kcal/mol)
XYZ coordinates for conf 1:
C          1.18747       -0.31638        0.75211
C         -0.04416        0.44589        0.29201
C          0.66763        1.60566        0.11573
C          1.95805        1.00830        0.49628
Br         1.77869       -1.82112       -0.40459
O          3.10923        1.35355        0.58146
C         -1.41931        0.02094        0.17464
C         -1.77296       -1.29704        0.53161
C         -3.09613       -1.71948        0.44456
C         -4.08403       -0.83769       -0.00332
C         -3.74512        0.47076       -0.36553
C         -2.42550        0.90129       -0.27925
Cl         0.26387        3.22025       -0.32084
H          1.19087       -0.70516        1.77076
H         -1.00570       -1.98731        0.86637
H         -3.35705       -2.73619        0.72239
H         -5.11608       -1.16925       -0.07276
H         -4.51247        1.15478       -0.71537
H         -2.16900        1.91623       -0.56116
```

Revised structure of 221{10}, i.e 223{10-rev} ($CDCl_3$)

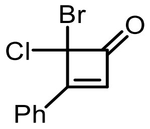

	Conf1
Rel energy (kcal/mol):	0.00

C-nom	iGau	Exp	Calc	diff	1
C-C	2	178.90	180.29	1.39	[180.29]
C-C	4	176.30	178.54	2.24	[178.54]
C-C	7	126.30	123.73	-2.57	[123.73]
C-C	1	76.40	77.47	1.07	[77.47]
C-CH	10	134.10	134.63	0.53	[134.63]
C-CH	8	130.10	131.10	1.00	[131.10]
C-CH	12	130.10	131.10	1.00	[131.10]
C-CH	9	129.70	128.38	-1.32	[128.38]
C-CH	11	129.70	128.38	-1.32	[128.38]
C-CH	3	128.10	128.31	0.21	[128.31]

13C chem shifts: RMSD=1.43ppm (MAE=1.26) N=10 {-2.57 2.24}

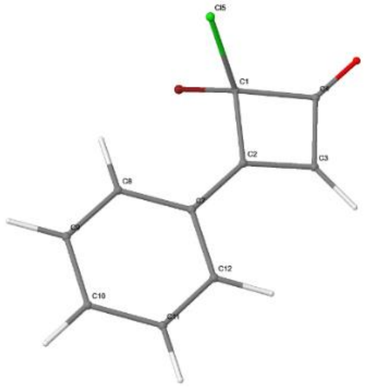

Conformer 1
Energy: -3491.44552 Hartree (Rel: 0.0 kcal/mol)
XYZ coordinates for conf 1:

C	-1.10231	0.27856	0.28948
C	0.21253	0.91669	-0.13196
C	-0.38421	2.07782	-0.54526
C	-1.73581	1.62970	-0.19070
Cl	-1.29354	-0.08418	2.04157
O	-2.86381	2.05319	-0.21478
C	1.57248	0.42570	-0.07588
C	1.85058	-0.87901	0.37695
C	3.16417	-1.33876	0.42638
C	4.21360	-0.50671	0.02724
C	3.94892	0.79173	-0.42409
C	2.63986	1.25644	-0.47608
H	-0.00840	2.99722	-0.97947
Br	-1.66332	-1.28185	-0.78956
H	1.03831	-1.52964	0.68197
H	3.36935	-2.34603	0.77589
H	5.23727	-0.86775	0.06729
H	4.76467	1.43830	-0.73313
H	2.43703	2.26521	-0.82291

Originally assigned (incorrect) structure of 222{9} (CD_3OD); additional linear scaling is used for methanol (crmsd)

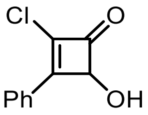

					**Conf1**
	Rel energy (kcal/mol):				0.00

**C-nom**	**iGau**	**Exp**	**Calc**	**diff**	**1**
C-C	4	166.90	174.45	7.55	[184.83]
C-C	2	152.40	162.08	9.68	[170.66]
C-C	7	128.80	124.31	-4.49	[127.41]
C-C	3	119.30	121.64	2.34	[124.35]
C-CH	10	131.00	130.76	-0.24	[134.79]
C-CH	8	131.00	128.25	-2.75	[131.92]
C-CH	12	128.48	128.25	-0.23	[131.92]
C-CH	9	128.46	125.50	-2.96	[128.77]
C-CH	11	128.46	125.50	-2.96	[128.77]
C-CH	1	98.80	84.89	-13.91	[82.26]

13C chem shifts: CRMSD=6.29ppm (CMAE=4.71) N=10 {-13.91 9.68}

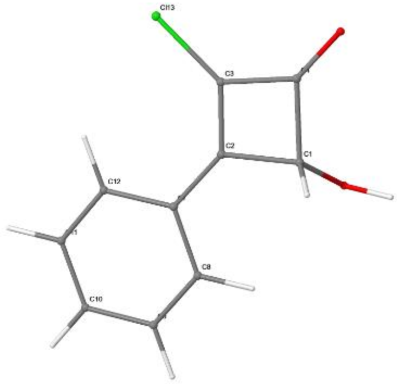

Conformer 1
Energy: -995.86130 Hartree (Rel: 0.0 kcal/mol)
XYZ coordinates for conf 1:

C	-1.21684	-1.50443	-0.33257
C	-0.38375	-0.23617	-0.12279
C	-1.49227	0.56334	-0.04364
C	-2.44670	-0.54648	-0.18096
O	-1.04666	-2.46595	0.68671
O	-3.64860	-0.68968	-0.14786
C	1.05153	-0.07414	-0.06568
C	1.87427	-1.21866	-0.13006
C	3.26030	-1.09287	-0.09682
C	3.84619	0.17286	0.00251
C	3.04068	1.31519	0.07255
C	1.65454	1.19769	0.04111
Cl	-1.74828	2.26708	0.10679
H	-1.11747	-1.94337	-1.33402
H	1.41529	-2.20022	-0.18454
H	3.88429	-1.98033	-0.14512
H	4.92778	0.26986	0.02867
H	3.49608	2.29793	0.15111
H	1.03565	2.08665	0.09230
H	-1.48663	-3.28370	0.40361

Revised structure of 222{9}, i.e 224{9-rev} (CD_3OD); additional linear scaling is used for methanol (crmsd)

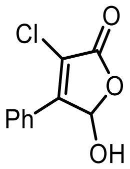

					Conf1
		Rel energy (kcal/mol):			0.00

C-nom	iGau	Exp	Calc	diff	1
C-C	10	166.90	168.66	1.76	[169.97]
C-C	7	152.40	150.22	-2.18	[151.12]
C-C	5	128.80	126.76	-2.04	[127.13]
C-C	11	119.30	119.82	0.52	[120.04]
C-CH	2	131.00	132.72	1.72	[133.22]
C-CH	4	131.00	130.15	-0.85	[130.60]
C-CH	6	128.48	130.15	1.67	[130.60]
C-CH	1	128.46	128.27	-0.19	[128.68]
C-CH	3	128.46	128.27	-0.19	[128.68]
C-CH	8	98.80	98.65	-0.15	[98.40]

13C chem shifts: CRMSD=1.37ppm (CMAE=1.13) N=10 {-2.18 1.76}

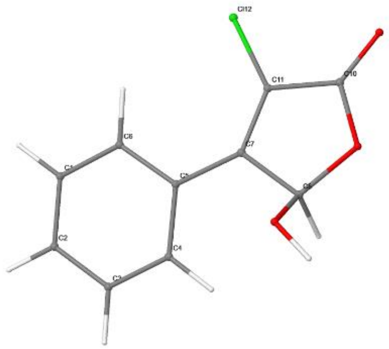

Conformer 1
Energy: -1071.13030 Hartree (Rel: 0.0 kcal/mol)
XYZ coordinates for conf 1:

C	-3.11660	1.36318	0.29682
C	-3.96424	0.30640	-0.04622
C	-3.41841	-0.93320	-0.38888
C	-2.03804	-1.11488	-0.39050
C	-1.16740	-0.05400	-0.05978
C	-1.73494	1.18982	0.28897
C	0.27904	-0.26952	-0.08517
C	0.86395	-1.67672	-0.17451
O	2.30212	-1.50626	-0.18817
C	2.61170	-0.18387	-0.11511
C	1.33716	0.56972	-0.05783
Cl	1.41304	2.29342	-0.00019
O	3.74660	0.23144	-0.11683
O	0.46925	-2.42334	0.92504
H	-3.53196	2.32768	0.57383
H	-5.04130	0.44670	-0.04248
H	-4.06806	-1.76247	-0.65267
H	-1.63875	-2.08903	-0.64768
H	-1.09795	2.01872	0.56832
H	0.61799	-2.16849	-1.12174
H	0.68151	-3.35751	0.75866

Originally assigned correct structure of 225{35b} with correct referencing of the specturm (CDCl$_3$)

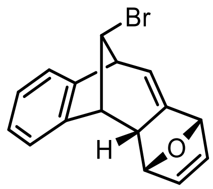

					**Conf1**
		Rel energy (kcal/mol):			0.00
**C-nom**	**iGau**	**Exp**	**Calc**	**diff**	**1**
C	5	146.10	145.36	-0.74	[145.36]
C	4	138.10	137.43	-0.67	[137.43]
C	13	134.10	135.86	1.76	[135.86]
C	15	130.30	132.01	1.71	[132.01]
C	16	130.00	131.88	1.88	[131.88]
C	1	127.60	126.78	-0.82	[126.78]
C	2	125.60	125.24	-0.36	[125.24]
C	3	125.10	124.46	-0.64	[124.46]
C	6	119.40	119.21	-0.19	[119.21]
C	11	116.90	115.43	-1.47	[115.43]
C	17	80.40	81.41	1.01	[81.41]
C	14	79.90	81.26	1.36	[81.26]
C	9	53.00	54.30	1.30	[54.30]
C	8	48.10	49.53	1.43	[49.53]
C	7	45.40	46.59	1.19	[46.59]
C	12	41.20	42.71	1.51	[42.71]

13C chem shifts: RMSD=1.23ppm (MAE=1.13) N=16 {-1.47 1.88}

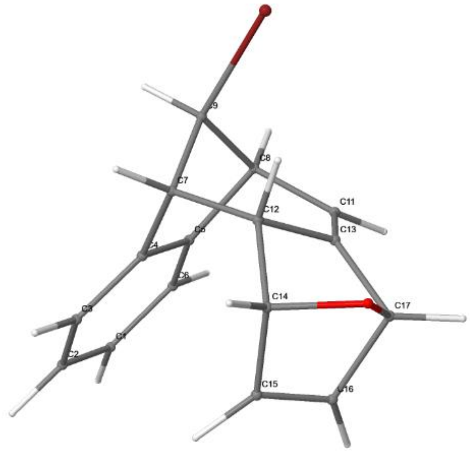

Conformer 1
Energy: -3264.06563 Hartree (Rel: 0.0 kcal/mol)
XYZ coordinates for conf 1:

C	-3.38761	-2.16158	0.44014
C	-3.47987	-1.61873	-0.84512
C	-2.37626	-0.98616	-1.43322
C	-1.18840	-0.89887	-0.71305
C	-1.09495	-1.46111	0.57734
C	-2.18879	-2.08998	1.16250
C	0.15929	-0.31901	-1.11974
C	0.32119	-1.22027	1.10710
C	1.04445	-1.26628	-0.25400
Br	2.98732	-0.84120	-0.17734
C	0.30363	0.18590	1.70623
C	0.43254	1.11775	-0.62301
C	0.28298	1.23822	0.88478
C	-0.35868	2.40315	-1.05589

C	-1.79611	2.34919	-0.55117
C	-1.72179	2.52685	0.77315
C	-0.22657	2.67059	1.08081
O	0.24517	3.32666	-0.11499
H	1.47332	1.34228	-0.88679
H	-4.25022	-2.65602	0.87887
H	-4.41271	-1.69581	-1.39705
H	-2.44910	-0.57862	-2.43857
H	-2.11678	-2.52652	2.15540
H	0.36228	-0.41241	-2.19089
H	0.67598	-1.97746	1.80991
H	1.02948	-2.27705	-0.65977
H	0.10883	0.27817	2.77269
H	-0.18740	2.73708	-2.07945
H	-2.66608	2.11708	-1.15230
H	-2.50686	2.44762	1.51580
H	0.07151	3.21232	1.97775

Originally assigned correct structure of 226{36b} with incorrect referencing of the specturm ($CDCl_3$)

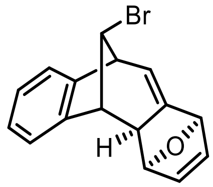

					**Conf1**
	Rel energy (kcal/mol):				0.00
**C-nom**	**iGau**	**Exp**	**Calc**	**diff**	**1**
C	4	146.90	146.94	0.04	[146.94]
C	5	143.30	143.50	0.20	[143.50]
C	13	139.50	142.86	3.36	[142.86]
C	16	134.10	136.35	2.25	[136.35]
C	15	130.90	134.33	3.43	[134.33]
C	2	126.50	126.56	0.06	[126.56]
C	1	126.30	126.33	0.03	[126.33]
C	6	121.50	122.03	0.53	[122.03]
C	3	119.80	120.59	0.79	[120.59]
C	11	115.90	115.95	0.05	[115.95]
C	14	80.50	82.36	1.86	[82.36]
C	17	79.40	81.80	2.40	[81.80]
C	9	48.55	50.87	2.32	[50.87]
C	8	44.60	47.91	3.31	[47.91]
C	12	44.10	46.86	2.76	[46.86]
C	7	43.30	46.34	3.04	[46.34]

13C chem shifts: RMSD=2.11ppm (MAE=1.65) N=16 {0.03 3.43}

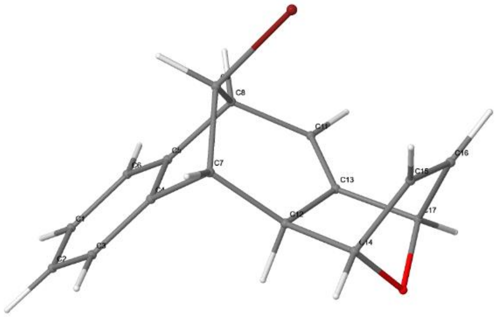

Conformer 1
Energy: -3264.05744 Hartree (Rel: 0.0 kcal/mol)
XYZ coordinates for conf 1:

C	-4.42994	0.15912	0.50164
C	-4.25798	0.62974	-0.80351
C	-2.99824	0.58918	-1.41742
C	-1.92371	0.07352	-0.70058
C	-2.09739	-0.40453	0.61217
C	-3.34788	-0.36864	1.21971
C	-0.44455	-0.05172	-1.08106
C	-0.76367	-0.95184	1.12342
C	-0.18120	-1.34028	-0.26417
Br	1.66260	-2.07666	-0.24819
C	0.13456	0.11352	1.75045
C	0.23150	1.22539	-0.49557
C	0.61381	1.08153	0.96571
C	1.51851	1.97666	-1.01706
C	2.79115	1.18759	-0.74766
C	2.97876	1.23954	0.57558
C	1.79689	2.04765	1.12105
O	1.57053	2.97408	0.03581
H	-0.52697	2.01544	-0.56152
H	-5.41402	0.19411	0.96128

H	-5.10953	1.02602	-1.35000
H	-2.86976	0.95252	-2.43397
H	-3.48905	-0.74566	2.22949
H	-0.26431	-0.17243	-2.15286
H	-0.88024	-1.81004	1.78935
H	-0.75320	-2.15995	-0.69698
H	0.44882	-0.00813	2.78423
H	1.40939	2.45592	-1.99024
H	3.34798	0.62475	-1.48606
H	3.70536	0.71100	1.18064
H	1.91647	2.56834	2.07074

Experimental procedure and spectral data for tribromide compound 227{17}:

(1*R*,2*S*,4*S*,7*R*)-2,7-Dibromobicyclo[2.2.1]heptane (S1): To a solution of norbornene (2.0 g, 21.6 mmol) in 400 ml of CH_2Cl_2 cooled to -78 °C was added bromine (4.0 g, 12.4 mmol) in 8 ml of CH_2Cl_2 for a period of 35min. After completion the reaction was quenched with 5 ml of 10% aqueous sodium bicarbonate, and the organic layer was extracted with CH_2Cl_2. The crude product was proceeded for next reaction. ^{1}H NMR (500 MHz, $CDCl_3$) δ 4.00 – 3.93 (m, 2H), 2.73 – 2.63 (m, 2H), 2.44 (t, J = 4.2 Hz, 1H), 2.28 – 2.19 (m, 1H), 1.76 – 1.60 (m, 2H), 1.39 – 1.23 (m, 2H); $^{13}C\{^1H\}$ NMR (126 MHz, $CDCl_3$) δ 53.84, 50.46, 48.26, 44.62, 42.14, 28.54, 25.30.

^{1}H NMR (500 MHz, $CDCl_3$)

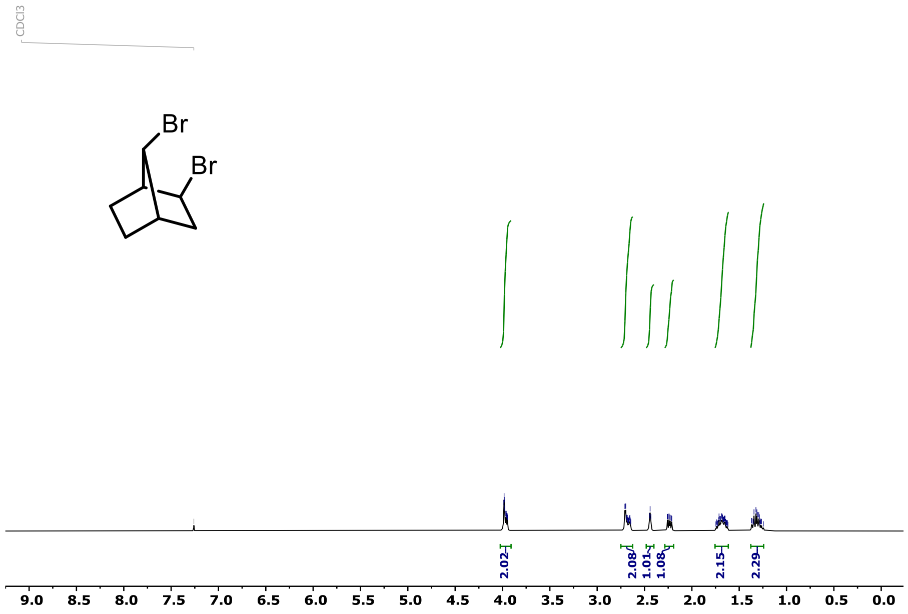

$^{13}C\{^1H\}$ NMR (126 MHz, $CDCl_3$)

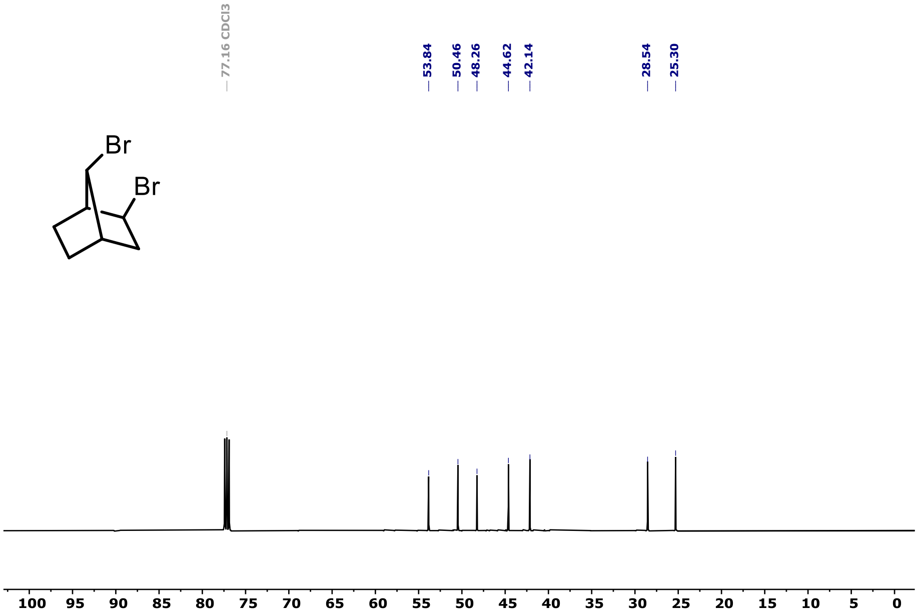

(1*R*,4*S*,7*r*)-7-Bromobicyclo[2.2.1]hept-2-ene (S2): To a stirred solution of (1*R*,2*S*,4*S*,7*R*)-2,7-Dibromobicyclo[2.2.1]heptane (120 mg, 0.473 mmol, 1 equiv.) in 10 mL of dry freshly distilled THF was added potassium *tert*-butoxide (212 mg, 1.89 mmol, 4 equiv.). The reaction mixture was stirred at room temperature for 48 h. After completion the solvent was evaporated, the mixture was diluted with water (20 mL) and the aqueous solution was extracted with ether (3 × 50 mL). The combined organic layers were washed with water (2 × 30 mL), dried over $CaCl_2$, and concentrated. The residue was filtered through a short silica gel column eluting with hexane to give Colorless liquid. ^{1}H NMR (500 MHz, $CDCl_3$) δ 6.03 (d, J = 2.0 Hz, 2H), 3.88 (s, 1H), 3.03 (p, J = 1.8 Hz, 2H), 1.84 – 1.70 (m, 2H), 1.18 – 1.05 (m, 2H); ^{13}C{^{1}H} NMR (126 MHz, $CDCl_3$) δ 133.01, 66.25, 49.48, 22.77.

^{1}H NMR (500 MHz, $CDCl_3$)

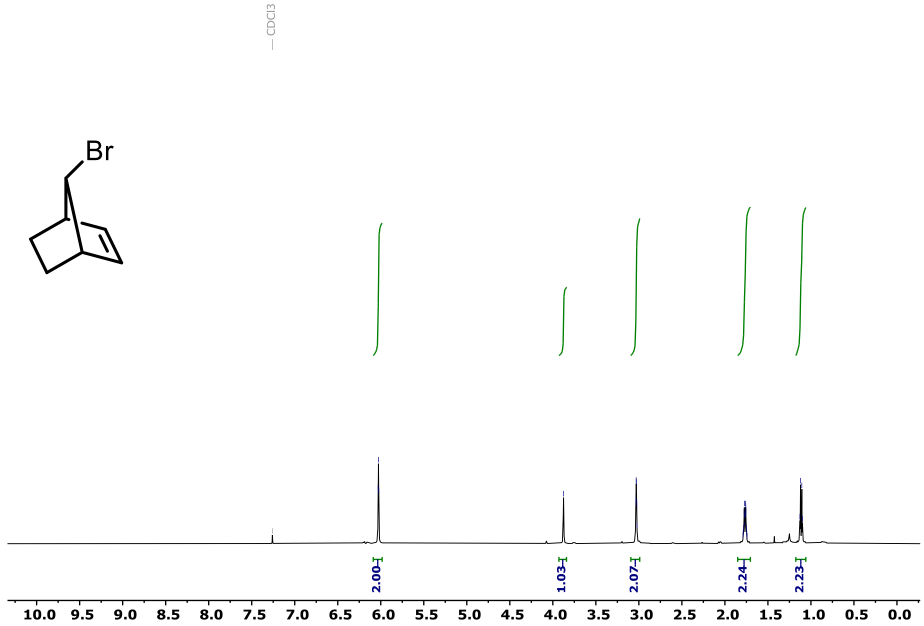

^{13}C{^{1}H} NMR (126 MHz, $CDCl_3$)

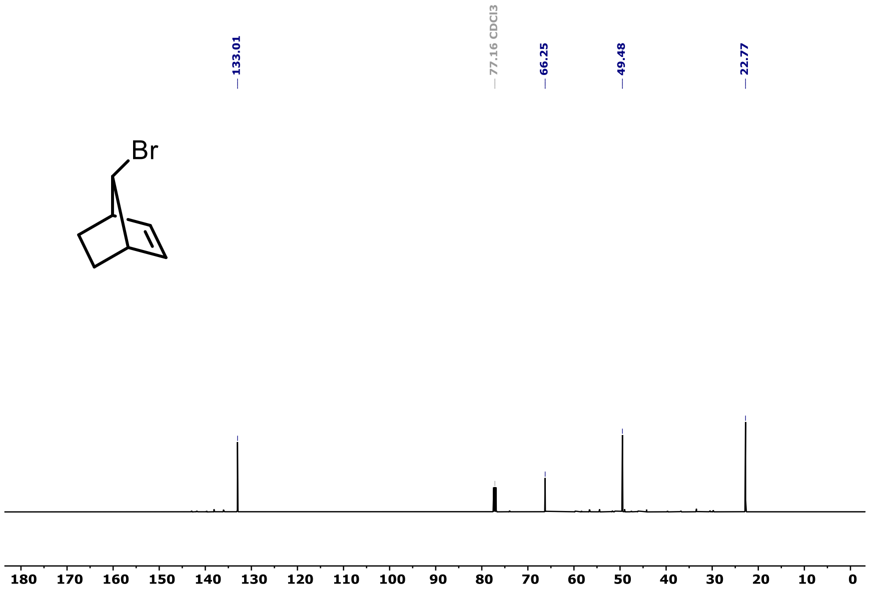

(1*R*,2*R*,3*S*,4*S*,7*s*)-2,3,7-Tribromobicyclo[2.2.1]heptane (227{17}): A solution of (1*R*,4*S*,7*r*)-7-Bromobicyclo[2.2.1]hept-2-ene (318 mg, 1.80 mmol) in 10 mL of carbon tetrachloride was reflux at 77 ºC. To the refluxing solution was added dropwise a hot solution of bromine (318 mg, ~ 0.1 mL, 2.00 mmol) in 3 mL of carbon tetrachloride over a period of 10 min. The resulting reaction mixture was heated for 10 min at reflux at 77 ºC. After completion the reaction mixture was cooled to room temperature and the solvent was evaporated. The oily residue was chromatographed on silica gel to obtain 100 mg eluting with hexane. ^{1}H NMR (500 MHz, $CDCl_3$) δ 4.98 (s, 2H), 4.24 (s, 1H), 2.54 (q, J = 2.5 Hz, 2H), 2.12 (q, J = 5.9 Hz, 2H), 1.68 – 1.61 (m, 2H). ^{13}C{^{1}H} NMR (126 MHz, $CDCl_3$) δ 54.25, 53.99, 49.78, 22.81.

Concentration dependence experiments:

As follows from two concentration experiments (A) and (B) below, tribromide **227{17}** exhibits no concentration dependence of ^{13}C chemical shift values.

(A). (1*R*,2*R*,3*S*,4*S*,7*s*)-2,3,7-Tribromobicyclo[2.2.1]heptane in chloroform-d (c = 0.03M).

^{1}H NMR (500 MHz, $CDCl_3$)

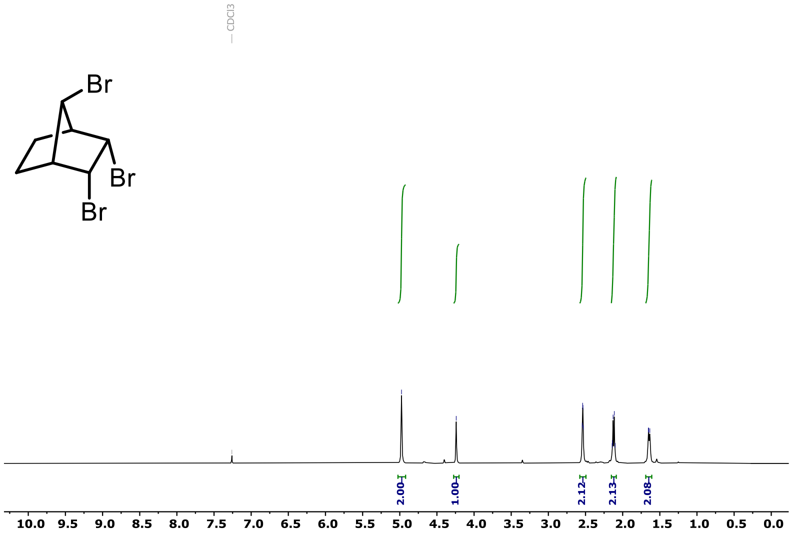

^{13}C{^{1}H} NMR (126 MHz, $CDCl_3$)

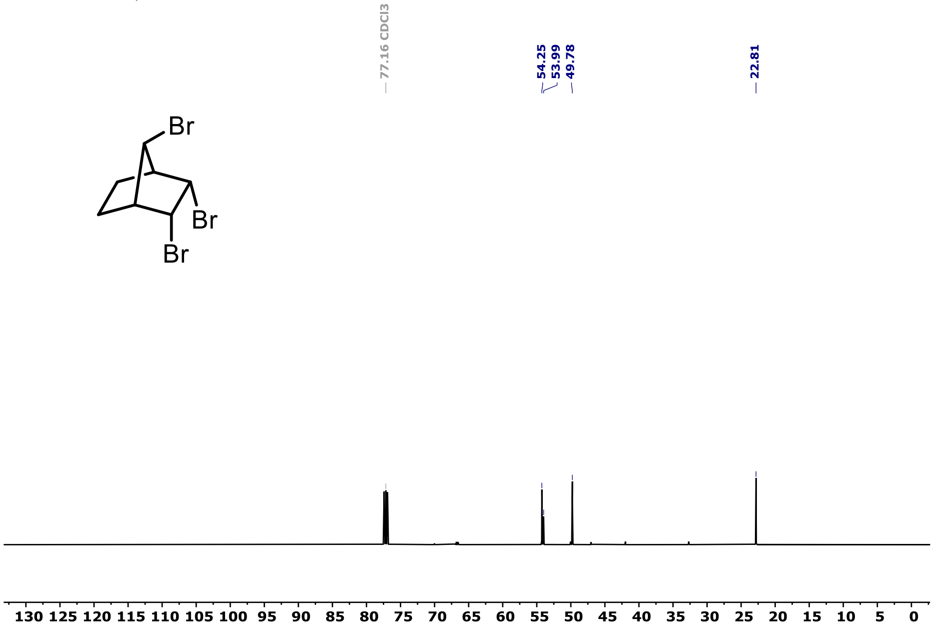

(B). (1*R*,2*R*,3*S*,4*S*,7*s*)-2,3,7-Tribromobicyclo[2.2.1]heptane in chloroform-d (c = 0.015M).

^{1}H NMR (500 MHz, CDCl$_3$)

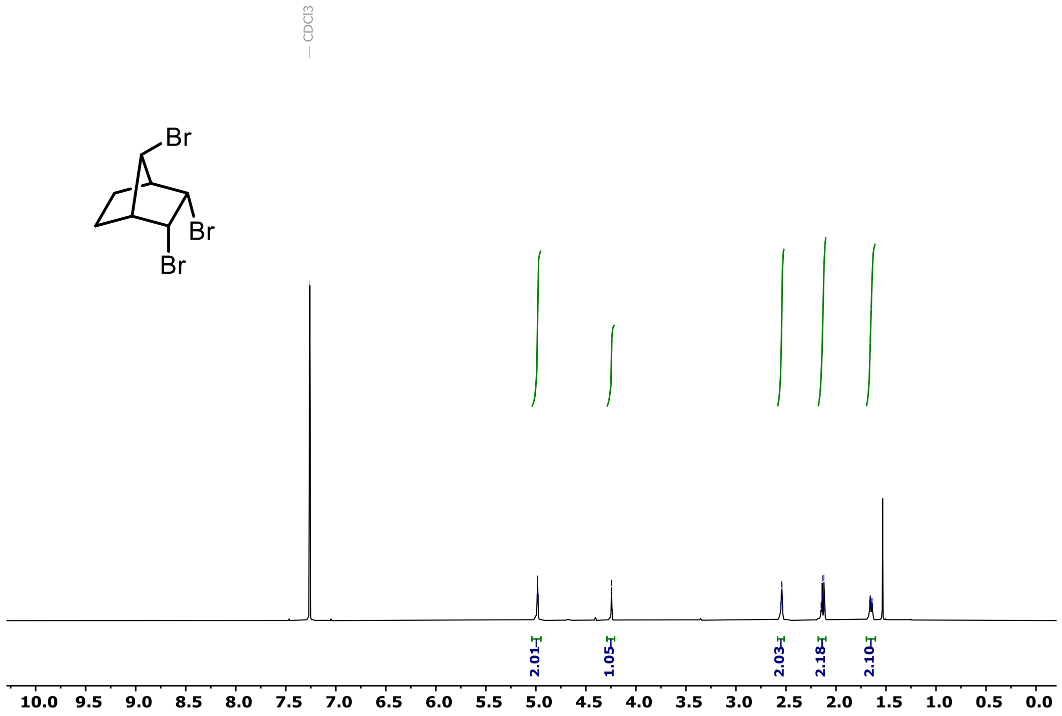

^{13}C{^{1}H} NMR (126 MHz, CDCl$_3$)

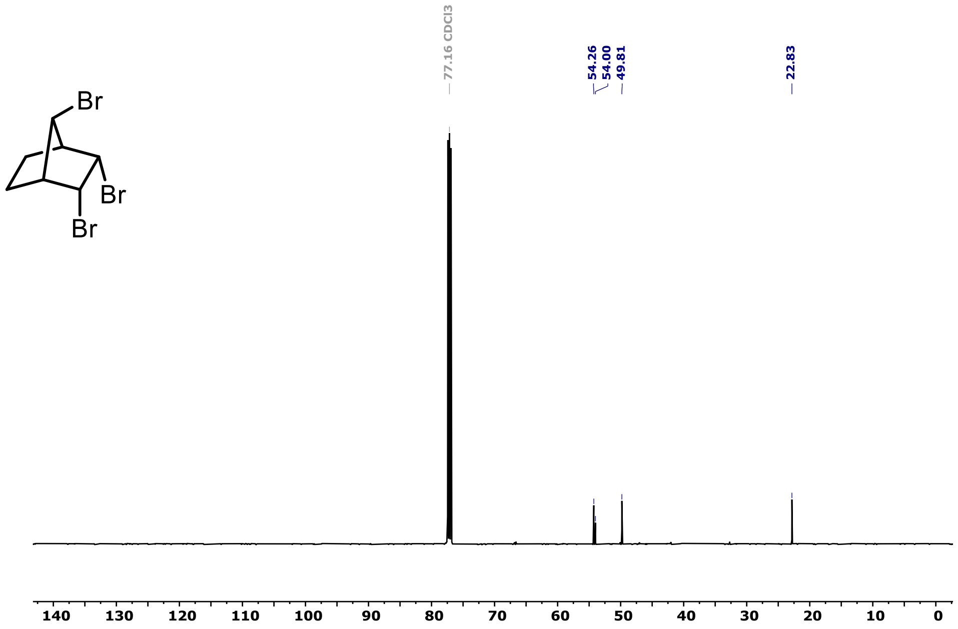

HSQC NMR ($CDCl_3$)

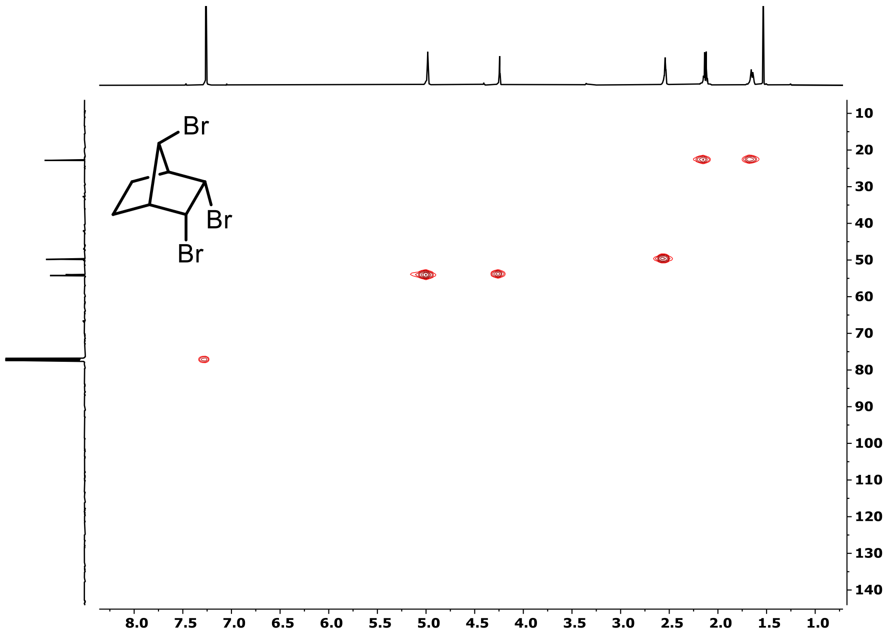

^{13}C peaks-Concentrated (0.03M)	^{13}C peaks-Diluted (0.015M)	Differences
54.25	54.26	0.001
53.99	54.00	0.001
49.78	49.81	0.003
22.81	22.83	0.002
	**rmsd**	**= 0.019365ppm**

Syn-7-bromonorbornene (S2) (synthetic, ^{13}C values, $CDCl_3$)

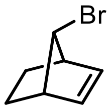

					**Conf1**
	Rel energy (kcal/mol):				0.00
**C-nom**	**iGau**	**Exp**	**Calc**	**diff**	**1**
C	9	133.01	133.64	0.63	[133.64]
C	10	133.01	133.63	0.62	[133.63]
C	2	66.25	65.19	-1.06	[65.19]
C	1	49.48	49.87	0.39	[49.87]
C	5	49.48	49.87	0.39	[49.87]
C	3	22.76	23.42	0.66	[23.42]
C	4	22.76	23.43	0.67	[23.43]

13C chem shifts: RMSD=0.66ppm (MAE=0.63) N=7 {-1.06 0.67}

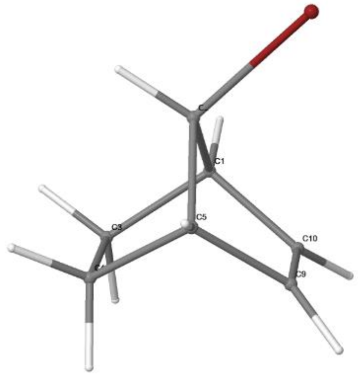

Conformer 1
Energy: -2843.52877 Hartree (Rel: 0.0 kcal/mol)
XYZ coordinates for conf 1:

C	-0.90103	-0.01911	1.13410
C	-0.05557	-0.62787	-0.00012
C	-2.26672	-0.71341	0.78252
C	-2.26676	-0.71304	-0.78277
C	-0.90101	-0.01869	-1.13412
H	-1.20798	2.26563	-1.33126
H	-0.02426	-1.71777	-0.00032
Br	1.85976	-0.09096	-0.00001
C	-1.03240	1.42409	-0.66956
C	-1.03254	1.42383	0.67005
H	-0.55346	-0.18448	2.15461
H	-3.11001	-0.16491	1.21009
H	-2.29391	-1.73375	1.17922
H	-2.29404	-1.73320	-1.17994
H	-3.11004	-0.16430	-1.21006
H	-1.20822	2.26512	1.33205
H	-0.55345	-0.18372	-2.15469

Syn-7-exo-2-dibromonorbornane (S3) (synthetic, ^{13}C values, $CDCl_3$)

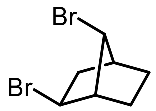

					Conf1
	Rel energy (kcal/mol):				0.00
**C-nom**	**iGau**	**Exp**	**Calc**	**diff**	**1**
C	2	53.84	54.08	0.24	[54.08]
C	1	50.46	51.89	1.43	[51.89]
C	10	48.26	49.72	1.46	[49.72]
C	5	44.62	44.40	-0.22	[44.40]
C	9	42.14	41.97	-0.17	[41.97]
C	3	28.54	28.19	-0.35	[28.19]
C	4	25.30	24.68	-0.62	[24.68]

13C chem shifts: RMSD=0.83ppm (MAE=0.64) N=7 {-0.62 1.46}

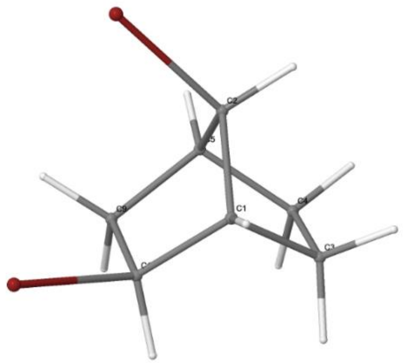

Conformer 1
Energy: -5415.57329 Hartree (Rel: 0.0 kcal/mol)
XYZ coordinates for conf 1:

C	-0.44340	-0.89521	-1.00348
C	-1.43288	0.08676	-0.34906
C	-1.37499	-2.14119	-1.05927
C	-2.16640	-2.05881	0.29237
C	-1.64256	-0.74421	0.92803
H	-0.10813	-1.80869	2.11411
H	-2.34035	0.22150	-0.93664
Br	-0.80195	1.94598	-0.04816
C	-0.19376	-0.96417	1.42359
C	0.58328	-1.21375	0.10492
H	-0.01512	-0.60581	-1.96269
H	-0.80564	-3.06956	-1.16624
H	-2.04765	-2.06770	-1.91956
H	-3.24532	-2.00664	0.11716
H	-1.97918	-2.91924	0.94249
H	0.95264	-2.23322	0.01527
H	-2.30483	-0.30769	1.67800
H	0.17374	-0.07110	1.93365
Br	2.28035	-0.16165	-0.03293

Stereoconfiguration is clarified for bromoladderane 231{S47} ($CDCl_3$)

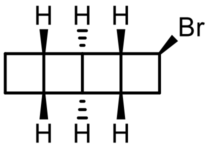

					Conf1	Conf2
	Rel energy (kcal/mol):				0.00	0.00

C-nom	iGau	Exp	Calc	diff	1	2
C	8	53.60	54.58	0.98	[54.58	54.58]
C	13	50.30	50.39	0.09	[50.40	50.37]
C	6	49.70	48.88	-0.82	[48.88	48.89]
C	5	49.00	48.84	-0.16	[48.84	48.83]
C	12	41.30	42.01	0.71	[42.01	42.00]
C	3	41.00	40.06	-0.94	[40.05	40.07]
C	4	40.90	39.94	-0.96	[39.94	39.94]
C	7	39.20	39.08	-0.12	[39.08	39.09]
C	1	26.33	25.58	-0.75	[25.58	25.59]
C	2	26.31	25.50	-0.81	[25.50	25.50]

13C chem shifts: RMSD=0.72ppm (MAE=0.63) N=10 {-0.96 0.98}

Fractions: 0.500 0.500

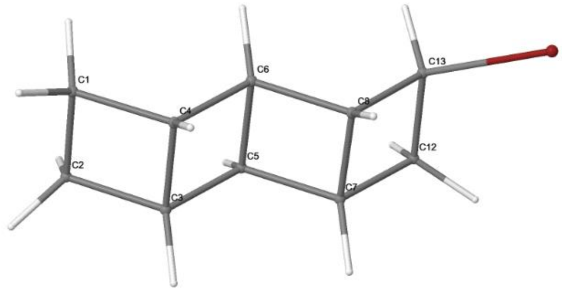

Conformer 1
Energy: -2960.17898 Hartree (Rel: 0.0 kcal/mol)
XYZ coordinates for conf 1:

C	-3.72448	-1.23776	0.32685
C	-4.13076	0.25671	0.15683
C	-2.82076	0.53467	-0.62664
C	-2.40601	-0.97845	-0.44797
C	-1.63805	0.97037	0.26059
C	-1.22607	-0.55485	0.45110
C	-0.33785	1.21533	-0.53253
C	0.07385	-0.29504	-0.33999
H	-2.17517	-1.65500	-1.27722
H	0.32288	-0.98132	-1.15239
H	-1.12881	-1.06377	1.41736
C	0.87750	1.64496	0.33288
C	1.23208	0.16005	0.57316
H	-2.91882	1.03279	-1.59678
H	-0.43268	1.69986	-1.50870
H	-1.85760	1.66494	1.07994
Br	3.04501	-0.36859	-0.06537
H	-3.59793	-1.59113	1.35659
H	-4.39778	-1.92967	-0.18873
H	-5.03426	0.39454	-0.44527
H	-4.24834	0.83253	1.08201
H	1.63740	2.17658	-0.24391
H	0.65713	2.22327	1.23719
H	1.20172	-0.19901	1.60223

Conformer 2
Energy: -2960.17898 Hartree (Rel: 0.0 kcal/mol)
XYZ coordinates for conf 2:

C	3.72406	-1.23808	-0.32688

C	4.13103	0.25624	-0.15757
C	2.82112	0.53531	0.62557
C	2.40621	-0.97809	0.44894
C	1.63801	0.96974	-0.26173
C	1.22565	-0.55566	-0.44984
C	0.33843	1.21601	0.53221
C	-0.07381	-0.29451	0.34131
H	2.17631	-1.65373	1.27918
H	-0.32327	-0.97969	1.15451
H	1.12791	-1.06594	-1.41534
C	-0.87764	1.64533	-0.33221
C	-1.23201	0.16039	-0.57216
H	2.91928	1.03477	1.59501
H	0.43450	1.70127	1.50789
H	1.85695	1.66318	-1.08220
Br	-3.04511	-0.36873	0.06498
H	3.59605	-1.59106	-1.35658
H	4.39756	-1.93053	0.18768
H	5.03425	0.39379	0.44501
H	4.24945	0.83152	-1.08297
H	-1.63711	2.17650	0.24560
H	-0.65847	2.22396	-1.23661
H	-1.20071	-0.19863	-1.60122

Stereoconfiguration is clarified for sulfoxide 232{S42} ($CDCl_3$)

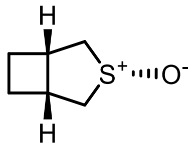

					Conf1	Conf2	Conf3
Rel energy (kcal/mol):					0.00	2.08	2.08
**C-nom**	**iGau**	**Exp**	**Calc**	**diff**	**1**	**2**	**3**
C	5	59.60	59.00	-0.60	[59.05	59.41	57.02]
C	7	59.60	58.99	-0.61	[59.04	57.02	59.41]
C	4	41.70	41.73	0.03	[42.19	34.62	33.53]
C	3	41.70	41.72	0.02	[42.18	33.53	34.62]
C	1	24.60	23.84	-0.76	[23.78	21.13	28.64]
C	2	24.60	23.84	-0.76	[23.78	28.64	21.13]

13C chem shifts: RMSD=0.56ppm (MAE=0.46) N=6 {-0.76 0.03}

Fractions: 0.943 0.028 0.028

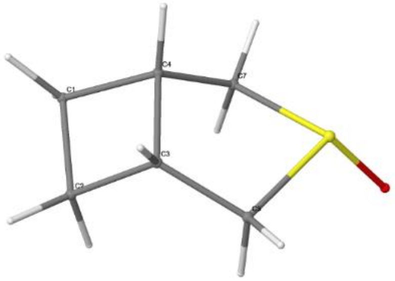

Conformer 1
Energy: -707.99750 Hartree (Rel: 0.0 kcal/mol)
XYZ coordinates for conf 1:

C	-1.70925	-0.77781	0.71024
C	-1.70993	0.77714	0.71031
C	-1.01739	0.78594	-0.68467
C	-1.01712	-0.78583	-0.68497
C	0.42100	1.30158	-0.74652
S	1.54172	0.00018	-0.04460
C	0.42138	-1.30101	-0.74750
O	1.39365	-0.00042	1.47385
H	-2.70286	-1.23596	0.71461
H	-1.11053	-1.22469	1.50742
H	-1.11205	1.22463	1.50778
H	-2.70400	1.23431	0.71425
H	-1.61535	1.24435	-1.47847
H	-1.61526	-1.24407	-1.47874
H	0.75902	1.46261	-1.77746
H	0.59605	2.21730	-0.17380
H	0.59679	-2.21716	-0.17558
H	0.75930	-1.46103	-1.77864

Conformer 2
Energy: -707.99419 Hartree (Rel: 2.1 kcal/mol)
XYZ coordinates for conf 2:

C	-2.34555	-0.74047	0.24686
C	-2.11705	0.75647	0.59515
C	-1.00154	0.83171	-0.49186
C	-1.02844	-0.73967	-0.58012
C	0.38622	1.34189	-0.09345
S	1.56574	-0.07704	-0.35505
C	0.21959	-1.25776	0.12886
O	2.64410	-0.04597	0.71830
H	-3.22260	-0.89378	-0.38894
H	-2.39698	-1.44508	1.08275

H	-1.72351	0.90583	1.60582
H	-2.97051	1.42671	0.45739
H	-1.35417	1.30378	-1.41235
H	-1.10096	-1.15576	-1.58919
H	0.74356	2.18898	-0.68401
H	0.47096	1.59149	0.97052
H	0.15336	-1.20661	1.22236
H	0.51687	-2.26825	-0.16265

Conformer 3
Energy: -707.99419 Hartree (Rel: 2.1 kcal/mol)
XYZ coordinates for conf 3:

C	2.11706	0.75647	0.59515
C	2.34555	-0.74048	0.24686
C	1.02844	-0.73967	-0.58012
C	1.00154	0.83171	-0.49186
C	-0.21959	-1.25776	0.12886
S	-1.56574	-0.07703	-0.35505
C	-0.38622	1.34189	-0.09345
O	-2.64410	-0.04597	0.71830
H	2.97052	1.42671	0.45737
H	1.72352	0.90584	1.60581
H	2.39698	-1.44508	1.08276
H	3.22259	-0.89379	-0.38893
H	1.10096	-1.15577	-1.58919
H	1.35417	1.30378	-1.41235
H	-0.51687	-2.26825	-0.16265
H	-0.15337	-1.20660	1.22236
H	-0.47096	1.59148	0.97053
H	-0.74356	2.18898	-0.68400

Stereoconfiguration is clarified for sulfoxide 233{S55} ($CDCl_3$)

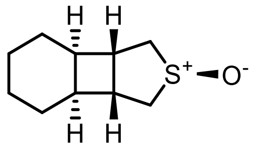

	Conf1	Conf2	Conf3	Conf4
Rel energy (kcal/mol):	0.00	0.00	0.24	0.24

C-nom	iGau	Exp	Calc	diff	1	2	3	4
C	9	58.50	57.09	-1.41	[56.04	59.58	56.87	55.16]
C	11	58.50	57.09	-1.41	[59.58	56.04	55.16	56.87]
C	7	44.10	43.88	-0.22	[49.35	42.08	43.01	39.22]
C	8	44.10	43.88	-0.22	[42.08	49.35	39.22	43.01]
C	4	35.00	34.33	-0.67	[30.30	38.47	34.68	33.79]
C	5	35.00	34.33	-0.67	[38.47	30.30	33.79	34.68]
C	3	27.10	27.16	0.06	[29.48	24.74	30.29	24.19]
C	6	27.10	27.16	0.06	[24.74	29.48	24.19	30.29]
C	2	21.80	21.83	0.03	[22.48	21.30	22.18	21.29]
C	1	21.80	21.83	0.03	[21.30	22.48	21.29	22.18]

13C chem shifts: RMSD=0.71ppm (MAE=0.48) N=10 {-1.41 0.06}

Fractions: 0.300 0.300 0.200 0.200

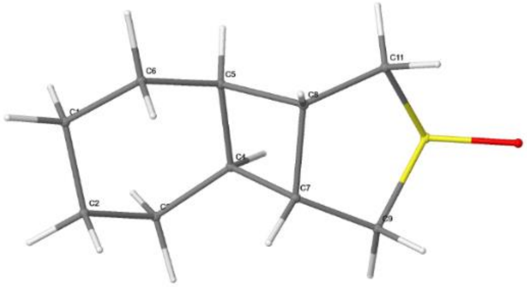

Conformer 1
Energy: -864.04292 Hartree (Rel: 0.0 kcal/mol)
XYZ coordinates for conf 1:

C	3.17657	0.81575	-0.55851
C	3.41560	-0.66749	-0.24063
C	2.10303	-1.45555	-0.36118
C	1.00374	-0.93347	0.58573
C	0.92403	0.62674	0.66184
C	2.20276	1.44396	0.45131
C	-0.40689	-0.92522	-0.07885
C	-0.20941	0.59019	-0.41154
C	-1.61004	-1.00685	0.86178
S	-2.86316	0.18985	0.21326
C	-1.49195	1.39548	-0.15644
H	0.47629	0.92189	1.61946
H	0.16935	0.75039	-1.42483
H	1.07236	-1.44094	1.55542
H	-0.54130	-1.59891	-0.92978
O	-3.35957	-0.35661	-1.11968
H	4.12259	1.37137	-0.54726
H	2.77716	0.90266	-1.57968
H	4.16451	-1.08860	-0.92308
H	3.82494	-0.76495	0.77609
H	2.26807	-2.52428	-0.17482
H	1.76019	-1.37802	-1.40287
H	2.72069	1.52561	1.41790
H	1.94400	2.47003	0.15478
H	-2.08506	-1.99043	0.90596
H	-1.37618	-0.68354	1.88365
H	-1.41226	2.06598	0.70738
H	-1.84271	1.97583	-1.01410

Conformer 2
Energy: -864.04331 Hartree (Rel: 0.0 kcal/mol)
XYZ coordinates for conf 2:

C	-3.41559	-0.66749	-0.24063
C	-3.17657	0.81575	-0.55852
C	-2.20276	1.44396	0.45130
C	-0.92403	0.62674	0.66185
C	-1.00374	-0.93347	0.58573
C	-2.10303	-1.45555	-0.36118
C	0.20941	0.59019	-0.41154
C	0.40689	-0.92522	-0.07885
C	1.49195	1.39548	-0.15644
S	2.86316	0.18985	0.21326
C	1.61004	-1.00685	0.86178
H	-1.07236	-1.44094	1.55542
H	0.54130	-1.59890	-0.92978
H	-0.47629	0.92189	1.61946
H	-0.16934	0.75039	-1.42482
O	3.35957	-0.35661	-1.11968
H	-3.82494	-0.76495	0.77609
H	-4.16451	-1.08860	-0.92308
H	-2.77715	0.90265	-1.57968
H	-4.12259	1.37137	-0.54727
H	-2.72069	1.52561	1.41790
H	-1.94400	2.47003	0.15477
H	-2.26807	-2.52428	-0.17482
H	-1.76019	-1.37803	-1.40287
H	1.84271	1.97583	-1.01409
H	1.41226	2.06598	0.70739
H	1.37618	-0.68354	1.88365
H	2.08506	-1.99044	0.90596

Conformer 3
Energy: -864.04331 Hartree (Rel: 0.2 kcal/mol)
XYZ coordinates for conf 3:

C	3.18169	-0.95197	0.24092
C	3.45812	0.49157	-0.20330
C	2.27952	1.39694	0.18137
C	0.94294	0.94747	-0.44434
C	0.73177	-0.60245	-0.45678
C	1.95705	-1.52374	-0.49036
C	-0.25101	1.07716	0.54874
C	-0.14424	-0.46501	0.82880
C	-1.64102	1.37985	-0.02074
S	-2.48746	-0.21483	-0.49284
C	-1.51545	-1.11774	0.80935
H	0.07885	-0.87332	-1.29630
H	0.40170	-0.70593	1.74648
H	0.80324	1.43215	-1.41842
H	-0.05703	1.73561	1.40132
O	-3.94769	-0.13494	-0.06722
H	4.05241	-1.59100	0.04819
H	3.01997	-0.96621	1.32894
H	4.37863	0.86567	0.26206
H	3.62069	0.51824	-1.29111
H	2.47864	2.44041	-0.09378
H	2.19450	1.38268	1.27769
H	2.23527	-1.68684	-1.54132
H	1.68964	-2.51091	-0.08838
H	-2.30874	1.81445	0.73132
H	-1.63026	2.03077	-0.89977
H	-1.50084	-2.18098	0.55027
H	-2.07206	-0.98045	1.74403

Conformer 4
Energy: -864.04292 Hartree (Rel: 0.2 kcal/mol)
XYZ coordinates for conf 4:

C	-3.45812	0.49156	-0.20328
C	-3.18168	-0.95198	0.24091
C	-1.95704	-1.52374	-0.49038

C	-0.73177	-0.60243	-0.45678
C	-0.94295	0.94748	-0.44434
C	-2.27953	1.39693	0.18140
C	0.14423	-0.46499	0.82879
C	0.25102	1.07717	0.54872
C	1.51544	-1.11773	0.80936
S	2.48747	-0.21484	-0.49283
C	1.64101	1.37985	-0.02079
H	-0.80328	1.43216	-1.41842
H	0.05705	1.73562	1.40130
H	-0.07884	-0.87330	-1.29630
H	-0.40171	-0.70590	1.74647
O	3.94769	-0.13494	-0.06718
H	-3.62070	0.51824	-1.29109
H	-4.37863	0.86565	0.26209
H	-3.01995	-0.96624	1.32893
H	-4.05240	-1.59102	0.04818
H	-2.23526	-1.68682	-1.54134
H	-1.68962	-2.51092	-0.08841
H	-2.47865	2.44040	-0.09373
H	-2.19450	1.38265	1.27771
H	2.07205	-0.98042	1.74404
H	1.50083	-2.18097	0.55030
H	1.63024	2.03073	-0.89985
H	2.30874	1.81448	0.73125

Stereoconfiguration is clarified for acetal 234{19} ($CDCl_3$)

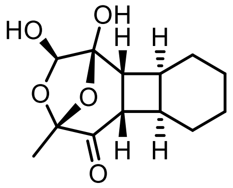

					**Conf1**
		Rel energy (kcal/mol):			0.00
**C-nom**	**iGau**	**Exp**	**Calc**	**diff**	**1**
C	13	202.10	201.53	-0.57	[201.53]
C	12	104.60	105.42	0.82	[105.42]
C	9	103.80	104.53	0.73	[104.53]
C	10	93.70	94.61	0.91	[94.61]
C	1	44.10	43.32	-0.78	[43.32]
C	2	40.90	41.69	0.79	[41.69]
C	3	36.40	38.06	1.66	[38.06]
C	8	31.20	30.88	-0.32	[30.88]
C	4	28.70	30.67	1.97	[30.67]
C	5	26.50	24.61	-1.89	[24.61]
C	7	22.40	22.24	-0.16	[22.24]
C	6	21.80	21.11	-0.69	[21.11]
C	16	18.10	17.77	-0.33	[17.77]

13C chem shifts: RMSD=1.06ppm (MAE=0.89) N=13 {-1.89 1.97}

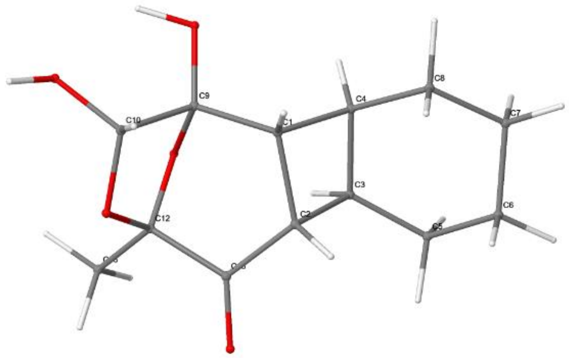

Conformer 1
Energy: -882.29936 Hartree (Rel: 0.0 kcal/mol)
XYZ coordinates for conf 1:

C	0.26813	-0.86367	-0.50272
C	0.56571	0.66516	-0.64901
C	1.54569	0.54981	0.58016
C	1.53143	-1.00203	0.40017
C	2.87975	1.30036	0.57870
C	3.95771	0.64473	-0.29811
C	4.06111	-0.85884	-0.00245
C	2.74620	-1.56190	-0.36892
C	-1.08091	-1.13287	0.16117
C	-2.27188	-1.00130	-0.82797
O	-2.52090	0.38532	-0.88194
C	-1.81817	1.01181	0.23008
C	-0.56510	1.63788	-0.42695
O	-1.06313	-2.32876	0.83905
O	-1.41059	-0.04159	1.07070
C	-2.71717	1.98605	0.95101
O	-3.34124	-1.71994	-0.26292
O	-0.50216	2.82080	-0.71194
H	0.31950	-1.44016	-1.43257
H	1.09377	0.91803	-1.57207
H	0.98904	0.80966	1.48512
H	1.38175	-1.56126	1.32820

H	3.25203	1.32919	1.61276
H	2.71329	2.34492	0.28291
H	3.72326	0.78443	-1.36377
H	4.92012	1.14188	-0.12485
H	4.88758	-1.30470	-0.56978
H	4.29090	-1.01066	1.06265
H	2.81691	-2.64344	-0.19882
H	2.59189	-1.43282	-1.45010
H	-2.05119	-1.35291	-1.83997
H	-1.99746	-2.59096	0.95410
H	-2.18762	2.43315	1.79738
H	-3.01839	2.78450	0.26932
H	-3.60171	1.45744	1.31484
H	-4.03447	-1.84409	-0.92982

Originally assigned (incorrect) structure of 236{6} ($CDCl_3$)

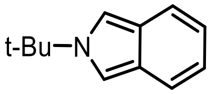

					Conf1
	Rel energy (kcal/mol):				0.00
**C-nom**	**iGau**	**Exp**	**Calc**	**diff**	**1**
C	3	167.99	123.00	-44.99	[123.00]
C	2	139.82	120.85	-18.97	[120.85]
C	12	133.61	119.89	-13.72	[119.89]
C	11	129.98	119.31	-10.67	[119.31]
C	10	126.94	119.24	-7.70	[119.24]
C	13	122.27	118.61	-3.66	[118.61]
C	4	121.42	108.47	-12.95	[108.47]
C	1	53.46	107.56	54.10	[107.56]
C	6	47.60	56.59	8.99	[56.59]
C	7	27.17	31.43	4.26	[31.43]
C	8	27.17	31.43	4.26	[31.43]
C	9	27.17	31.43	4.26	[31.43]

13C chem shifts: RMSD=22.34ppm (MAE=15.71) N=12 {-44.99 54.10}

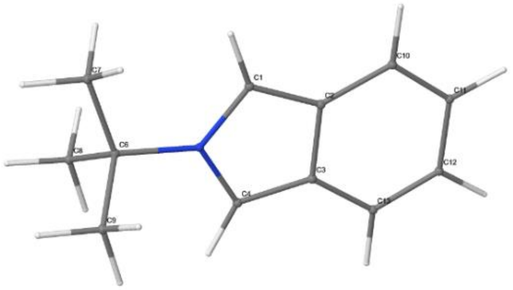

Conformer 1
Energy: -521.05260 Hartree (Rel: 0.0 kcal/mol)
XYZ coordinates for conf 1:

C	0.09673	1.14699	-0.00013
C	-1.23565	0.73256	-0.00007
C	-1.22456	-0.71633	-0.00006
C	0.11375	-1.10714	-0.00013
N	0.89174	0.02664	-0.00020
C	2.38553	-0.00160	0.00003
C	2.94442	1.42834	-0.00088
C	2.86211	-0.73541	1.26827
C	2.86253	-0.73712	-1.26706
C	-2.47458	1.43110	-0.00001
C	-3.64443	0.70707	0.00005
C	-3.63372	-0.72387	0.00005
C	-2.45435	-1.43204	-0.00001
H	0.51423	2.13990	-0.00018
H	0.55446	-2.09210	-0.00010
H	4.03738	1.37741	-0.00088
H	2.63488	1.98432	-0.89167
H	2.63498	1.98542	0.88925
H	3.95677	-0.75652	1.29590
H	2.50368	-1.76882	1.29527
H	2.50320	-0.22396	2.16758
H	2.50382	-0.22698	-2.16719
H	3.95720	-0.75811	-1.29434
H	2.50431	-1.77064	-1.29275
H	-2.49302	2.51857	-0.00002
H	-4.60040	1.22465	0.00010
H	-4.58221	-1.25518	0.00010
H	-2.45825	-2.51961	-0.00001

Revised structure of 236{6}, i.e isoindolinone 237{6-rev} ($CDCl_3$)

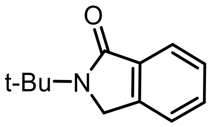

					Conf1
				Rel energy (kcal/mol):	0.00
**C-nom**	**iGau**	**Exp**	**Calc**	**diff**	**1**
C	1	167.99	168.52	0.53	[168.52]
C	3	139.82	140.81	0.99	[140.81]
C	2	133.61	133.55	-0.06	[133.55]
C	13	129.98	130.32	0.34	[130.32]
C	12	126.94	127.33	0.39	[127.33]
C	11	122.27	122.97	0.70	[122.97]
C	14	121.42	122.41	0.99	[122.41]
C	6	53.46	54.42	0.96	[54.42]
C	4	47.60	48.21	0.61	[48.21]
C	7	27.17	27.35	0.18	[27.35]
C	8	27.17	27.35	0.18	[27.35]
C	9	27.17	27.35	0.18	[27.35]

13C chem shifts: RMSD=0.61ppm (MAE=0.51) N=12 {-0.06 0.99}

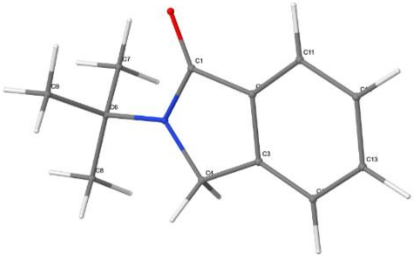

Conformer 1
Energy: -596.31004 Hartree (Rel: 0.0 kcal/mol)
XYZ coordinates for conf 1:

C	-0.11799	0.97841	-0.00055
C	1.30420	0.54410	-0.00040
C	1.35568	-0.84799	-0.00018
C	-0.05145	-1.38647	-0.00006
N	-0.87818	-0.17230	-0.00174
C	-2.36985	-0.17355	0.00003
C	-2.87706	0.54856	-1.26495
C	-2.89450	-1.61944	-0.00472
C	-2.87382	0.53979	1.27126
O	-0.53161	2.13972	0.00041
C	2.45956	1.32287	-0.00027
C	3.69012	0.66380	-0.00006
C	3.74712	-0.73875	0.00010
C	2.58028	-1.51128	0.00006
H	-0.24865	-2.00701	-0.88341
H	-0.24899	-2.00474	0.88487
H	-3.97291	0.56137	-1.27409
H	-2.51409	1.57778	-1.29307
H	-2.53361	0.02967	-2.16732
H	-3.98876	-1.59281	-0.00353
H	-2.57689	-2.17680	0.88274
H	-2.57858	-2.17006	-0.89699
H	-2.51014	1.56862	1.30571
H	-3.96963	0.55336	1.28319
H	-2.52856	0.01443	2.16920
H	2.39255	2.40685	-0.00038
H	4.61283	1.23723	-0.00001
H	4.71470	-1.23325	0.00026
H	2.63710	-2.59673	0.00022

Originally proposed (incorrect) structure of 239{19a} ($CDCl_3$)

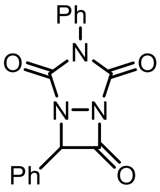

	Conf1	Conf2	Conf3
Rel energy (kcal/mol):	0.00	0.12	1.70

C-nom	iGau	Exp	Calc	diff	1	2	3
C-C	3	171.80	164.08	-7.72	[161.76	161.65	164.08]
C-C	7	154.50	156.46	1.96	[159.22	158.79	156.46]
C-C	5	152.80	148.15	-4.65	[148.25	148.65	148.15]
C-C	8	131.70	131.26	-0.44	[131.28	131.15	131.26]
C-C	17	129.00	125.46	-3.54	[128.45	128.51	125.46]
C-CH	20	130.50	133.43	2.93	[132.13	132.26	133.43]
C-CH	18	129.70	132.06	2.36	[129.73	130.07	132.06]
C-CH	22	129.70	132.06	2.36	[129.73	130.07	132.06]
C-CH	19	129.20	128.72	-0.48	[128.38	128.38	128.72]
C-CH	21	129.20	128.72	-0.48	[128.38	128.38	128.72]
C-CH	11	129.20	128.42	-0.78	[128.76	128.76	128.42]
C-CH	10	129.20	128.16	-1.04	[128.35	128.36	128.16]
C-CH	12	128.60	128.16	-0.44	[128.35	128.36	128.16]
C-CH	9	125.60	125.41	-0.19	[125.57	125.65	125.41]
C-CH	13	125.60	125.41	-0.19	[125.57	125.65	125.41]
C-CH	2	62.15	82.39	20.24	[81.72	82.10	82.39]

13C chem shifts: RMSD=5.75ppm (MAE=3.11) N=16 {-7.72 20.24}

Fractions:	0.000	0.000	1.000
Fractions-dervied rel energies:	>3	>3	0.00
Energy discrepancy:	>3.0	>2.9	1.70

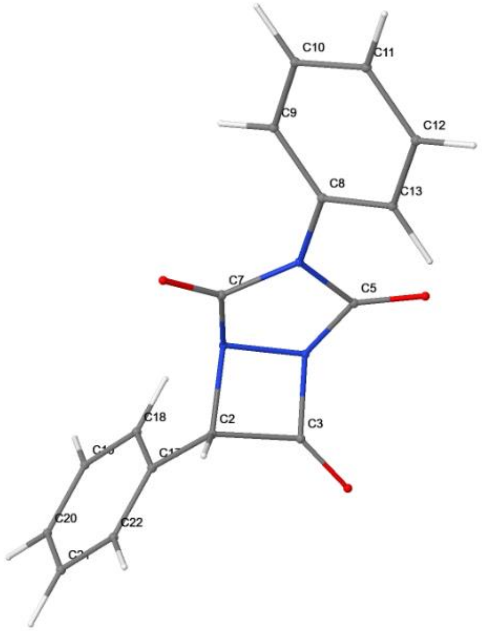

Conformer 1
Energy: -1006.21794 Hartree (Rel: 0.0 kcal/mol)
XYZ coordinates for conf 1:

N	0.60355	-0.20003	-0.32949
C	1.70885	0.26190	0.58480
C	1.29866	1.69990	0.16147
N	0.26916	1.16282	-0.69278

C	-1.13478	1.34300	-0.59824
N	-1.62060	0.08489	-0.20973
C	-0.57818	-0.80616	0.13098
C	-3.00979	-0.22762	-0.05326
C	-3.49463	-1.43452	-0.56618
C	-4.84461	-1.74872	-0.41284
C	-5.70634	-0.85908	0.23356
C	-5.21239	0.34675	0.73601
C	-3.86065	0.66647	0.60287
O	-0.67615	-1.88623	0.66619
O	-1.74815	2.36095	-0.80870
O	1.63480	2.82255	0.38349
C	3.09861	-0.21693	0.27217
C	3.49354	-0.48015	-1.04621
C	4.80176	-0.88054	-1.31561
C	5.72565	-1.01239	-0.27467
C	5.33525	-0.74886	1.03971
C	4.02381	-0.35636	1.31346
H	1.45219	0.09435	1.63647
H	-2.82261	-2.11796	-1.07321
H	-5.22232	-2.68758	-0.80678
H	-6.75839	-1.10479	0.34504
H	-5.87701	1.04191	1.24040
H	-3.47261	1.59901	0.99482
H	2.77416	-0.38597	-1.85422
H	5.10002	-1.08948	-2.33898
H	6.74417	-1.32436	-0.48772
H	6.04613	-0.85679	1.85378
H	3.71901	-0.16042	2.33840

Conformer 2
Energy: -1006.21524 Hartree (Rel: 0.1 kcal/mol)
XYZ coordinates for conf 2:

N	0.60536	-0.07839	-0.38788
C	1.71549	0.05167	0.62528
C	1.29566	1.54444	0.71803
N	0.25895	1.32285	-0.25820
C	-1.14447	1.44576	-0.08981
N	-1.61832	0.12580	-0.15902
C	-0.56827	-0.81807	-0.16285
C	-3.00922	-0.21435	-0.10685
C	-3.46008	-1.17640	0.80113
C	-4.81654	-1.50269	0.82928
C	-5.71502	-0.86390	-0.02814
C	-5.25377	0.10272	-0.92524
C	-3.89819	0.42662	-0.97454
O	-0.65180	-2.01908	-0.04162
O	-1.76914	2.46477	0.07348
O	1.63078	2.52752	1.30470
C	3.10430	-0.28570	0.16264
C	3.50918	-0.02431	-1.15341
C	4.81600	-0.30471	-1.54990
C	5.72905	-0.83919	-0.63571
C	5.32902	-1.09938	0.67629
C	4.01851	-0.82745	1.07363
H	1.46463	-0.46453	1.55860
H	-2.75994	-1.66594	1.46733
H	-5.16945	-2.25389	1.52967
H	-6.77055	-1.11797	0.00250
H	-5.94702	0.60269	-1.59508
H	-3.53268	1.17023	-1.67420
H	2.79858	0.38352	-1.86636
H	5.12207	-0.10554	-2.57288
H	6.74681	-1.05600	-0.94750
H	6.03151	-1.52134	1.38911
H	3.70578	-1.03919	2.09297

Conformer 3
Energy: -1006.21774 Hartree (Rel: 1.7 kcal/mol)
XYZ coordinates for conf 3:

N	-0.70027	0.99018	-1.36081

C	-2.12250	1.06929	-0.81741
C	-1.58398	2.21259	0.07688
N	-0.28812	2.10711	-0.53624
C	0.95040	1.79970	0.08739
N	1.27104	0.52339	-0.39254
C	0.20893	-0.05260	-1.13417
C	2.49074	-0.16494	-0.09392
C	3.17312	-0.81691	-1.12561
C	4.35827	-1.49411	-0.84001
C	4.86652	-1.50819	0.46127
C	4.18037	-0.84772	1.48272
C	2.98536	-0.17946	1.21347
O	0.13516	-1.18768	-1.54474
O	1.56285	2.50082	0.85612
O	-1.99229	2.93235	0.93806
C	-2.70542	-0.14902	-0.15717
C	-3.57686	-0.96496	-0.89175
C	-4.11703	-2.11533	-0.31756
C	-3.79523	-2.45645	0.99823
C	-2.93326	-1.64375	1.73845
C	-2.38916	-0.49460	1.16481
H	-2.77846	1.44168	-1.60796
H	2.77724	-0.79809	-2.13472
H	4.88695	-2.00497	-1.63933
H	5.79308	-2.03184	0.67797
H	4.56949	-0.85568	2.49660
H	2.44783	0.33035	2.00401
H	-3.82912	-0.69865	-1.91474
H	-4.79056	-2.74117	-0.89556
H	-4.21941	-3.34966	1.44781
H	-2.68792	-1.89974	2.76489
H	-1.73503	0.13780	1.75885

Revised structure of 239{19a}, i.e. zwitterion 243{19a-rev} ($CDCl_3$)

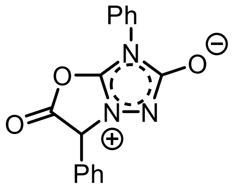

					**Conf1**
	Rel energy (kcal/mol):				0.00

**C-nom**	**iGau**	**Exp**	**Calc**	**diff**	**1**
C-C	21	171.80	172.17	0.37	[172.17]
C-C	3	154.50	154.31	-0.19	[154.31]
C-C	5	152.80	152.92	0.12	[152.92]
C-C	6	131.70	132.00	0.30	[132.00]
C-C	19	129.00	127.86	-1.14	[127.86]
C-CH	16	130.50	131.92	1.42	[131.92]
C-CH	15	129.70	128.67	-1.03	[128.67]
C-CH	17	129.70	128.67	-1.03	[128.67]
C-CH	10	129.20	128.63	-0.57	[128.63]
C-CH	12	129.20	128.63	-0.57	[128.63]
C-CH	14	129.20	128.31	-0.89	[128.31]
C-CH	18	129.20	128.31	-0.89	[128.31]
C-CH	11	128.60	127.76	-0.84	[127.76]
C-CH	9	125.60	124.87	-0.73	[124.87]
C-CH	13	125.60	124.87	-0.73	[124.87]
C-CH	22	62.15	63.77	1.62	[63.77]

13C chem shifts: RMSD=0.88ppm (MAE=0.78) N=16 {-1.14 1.62}

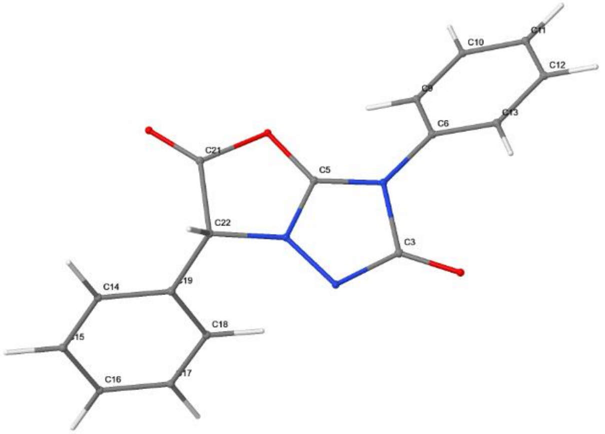

Conformer 1
Energy: -1006.20436 Hartree (Rel: 0.0 kcal/mol)
XYZ coordinates for conf 1:

N	-0.54869	0.51287	-0.70873
N	-0.21298	1.85287	-0.81311
C	1.10682	1.89234	-0.52639
N	1.56875	0.46392	-0.26391
C	0.46878	-0.26098	-0.41353
C	2.87018	0.02964	0.11674
O	0.19715	-1.58164	-0.40865
O	1.88292	2.83476	-0.46040
C	3.01903	-1.05414	0.99081
C	4.30009	-1.47964	1.34080
C	5.42450	-0.81905	0.84027
C	5.26284	0.26987	-0.01952
C	3.98880	0.69926	-0.39139
C	-4.19626	-0.49538	-0.60250
C	-5.34111	-0.27547	0.16305

C	-5.26451	0.47851	1.33722
C	-4.03964	1.01175	1.74254
C	-2.88818	0.78738	0.98456
C	-2.96718	0.03272	-0.18969
O	-1.70939	-2.74238	-0.87788
C	-1.17562	-1.68556	-0.79134
C	-1.73305	-0.25579	-1.02961
H	2.14690	-1.55739	1.39584
H	4.41516	-2.32351	2.01463
H	6.42020	-1.14908	1.12153
H	6.13264	0.78953	-0.41066
H	3.85278	1.54572	-1.05175
H	-4.25892	-1.07749	-1.51783
H	-6.29221	-0.68723	-0.16167
H	-6.15782	0.65499	1.92945
H	-3.97596	1.60655	2.64905
H	-1.94233	1.22005	1.29325
H	-1.96835	-0.16840	-2.09819

Originally proposed (incorrect) structure of 240{19j} ($CDCl_3$)

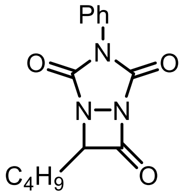

					Conf1	Conf2	Conf3	
Rel energy (kcal/mol):					0.00	0.39	0.59	
**C-nom**	**iGau**	**Exp**	**Calc**	**diff**	**1**	**2**	**3**	
C-C	3	173.80	163.50	-10.30	[163.45	164.07	163.50	]
C-C	7	154.90	159.92	5.02	[159.73	159.64	159.92	]
C-C	5	152.70	148.09	-4.61	[148.20	148.20	148.09	]
C-C	11	130.70	131.42	0.72	[131.32	131.29	131.42	]
C-CH	14	129.20	128.52	-0.68	[128.52	128.61	128.52	]
C-CH	13	129.20	128.27	-0.93	[128.27	128.28	128.27	]
C-CH	15	128.60	128.27	-0.33	[128.27	128.28	128.27	]
C-CH	12	125.70	125.39	-0.31	[125.40	125.56	125.39	]
C-CH	16	125.70	125.39	-0.31	[125.40	125.56	125.39	]
C-CH	2	58.20	79.76	21.56	[80.16	78.59	79.76	]
C-CH2	20	29.50	29.45	-0.05	[30.47	31.85	29.45	]
C-CH2	19	28.00	24.75	-3.25	[28.42	25.92	24.75	]
C-CH2	18	21.90	22.85	0.95	[22.86	23.10	22.85	]
C-CH3	17	13.70	13.60	-0.10	[13.66	13.63	13.60	]
**13C chem shifts: RMSD=6.71ppm (MAE=3.51) N=14 {-10.30 21.56}**								
Fractions:					0.000	0.000	1.000	
Fractions-dervied rel energies:					>3	>3	0.00	
Energy discrepancy:					>3.0	>2.6	0.59	

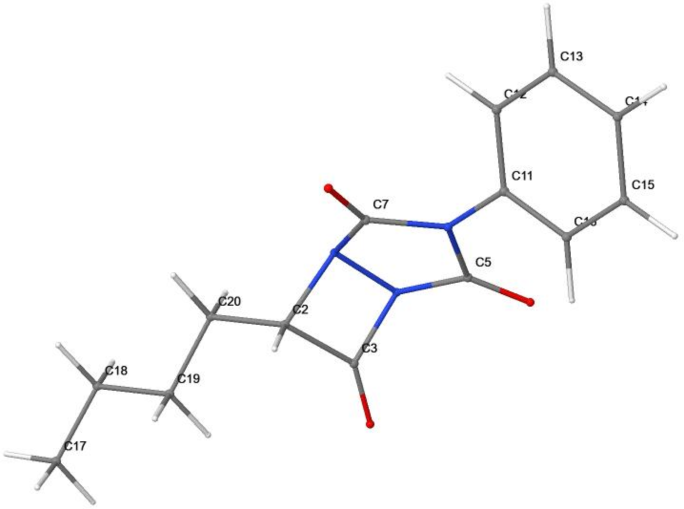

Conformer 1
Energy: -932.42756 Hartree (Rel: 0.0 kcal/mol)
XYZ coordinates for conf 1:

N	0.90594	0.26461	-0.41908
C	1.95304	0.86284	0.48816
C	1.29997	2.22159	0.17019
N	0.34145	1.57075	-0.69338
C	-1.06825	1.51009	-0.55421
N	-1.32889	0.16947	-0.22889
C	-0.14217	-0.55255	0.02977
O	-0.04118	-1.66484	0.49663
O	-1.84759	2.42231	-0.68920
O	1.45670	3.37315	0.44533
C	-2.64155	-0.37801	-0.06199

C	-2.93316	-1.62430	-0.62493
C	-4.20767	-2.16636	-0.46221
C	-5.18778	-1.46400	0.24343
C	-4.88748	-0.21694	0.79576
C	-3.61158	0.32991	0.65365
C	5.72028	-2.53441	0.01929
C	5.27338	-1.08490	-0.19746
C	3.83863	-0.82631	0.28033
C	3.39484	0.62726	0.06442
H	1.78214	0.57519	1.53243
H	-2.17082	-2.16220	-1.17739
H	-4.43447	-3.13613	-0.89518
H	-6.18113	-1.88689	0.36204
H	-5.64462	0.33364	1.34623
H	-3.37373	1.29542	1.08361
H	6.74631	-2.68981	-0.33276
H	5.68666	-2.80484	1.08180
H	5.07111	-3.23351	-0.52188
H	5.34968	-0.83249	-1.26419
H	5.95881	-0.40576	0.32866
H	3.75572	-1.07619	1.34768
H	3.14745	-1.49685	-0.24828
H	3.50684	0.90779	-0.99003
H	4.02423	1.31037	0.64953

Conformer 2
Energy: -932.42818 Hartree (Rel: 0.4 kcal/mol)
XYZ coordinates for conf 2:

N	0.62774	-0.43458	-0.65102
C	1.86292	-0.17650	0.17464
C	1.56535	1.32066	-0.04265
N	0.40946	0.98559	-0.84144
C	-0.95162	1.28891	-0.58431
N	-1.53133	0.04948	-0.27227
C	-0.56042	-0.96762	-0.12834
O	-0.72178	-2.08422	0.30982
O	-1.46976	2.37853	-0.62841
O	2.03720	2.37842	0.25120
C	-2.92493	-0.14301	-0.00519
C	-3.58126	-1.23416	-0.58265
C	-4.93689	-1.43228	-0.32226
C	-5.63532	-0.54014	0.49517
C	-4.97131	0.55045	1.06103
C	-3.61142	0.75131	0.82117
C	6.92628	-0.42625	0.66199
C	5.69728	-0.86382	-0.14133
C	4.38594	-0.29941	0.42161
C	3.15896	-0.74743	-0.38671
H	1.70943	-0.46459	1.22131
H	-3.03598	-1.91927	-1.22229
H	-5.44731	-2.28180	-0.76625
H	-6.69259	-0.69447	0.69012
H	-5.50842	1.24685	1.69817
H	-3.09144	1.59357	1.26172
H	7.84641	-0.84218	0.23641
H	7.02538	0.66608	0.67014
H	6.85851	-0.76162	1.70418
H	5.64036	-1.96109	-0.15963
H	5.80845	-0.54422	-1.18672
H	4.43591	0.79753	0.43618
H	4.27051	-0.61845	1.46724
H	3.07463	-1.84118	-0.37521
H	3.25848	-0.44460	-1.43611

Conformer 3
Energy: -932.42723 Hartree (Rel: 0.6 kcal/mol)
XYZ coordinates for conf 3:

N	-0.85241	-0.33847	-0.18389
C	-1.82944	0.25829	-1.16775
C	-1.57817	1.59536	-0.44046
N	-0.66288	0.94259	0.46635

C	0.72449	1.18083	0.63697
N	1.34143	0.03245	0.11676
C	0.41870	-0.81042	-0.54390
O	0.66352	-1.76909	-1.24099
O	1.23461	2.16140	1.12287
O	-1.94237	2.73087	-0.50878
C	2.75359	-0.20414	0.14036
C	3.22242	-1.47430	0.48980
C	4.59595	-1.71458	0.50899
C	5.49507	-0.69178	0.19705
C	5.01601	0.57512	-0.14331
C	3.64363	0.82469	-0.18150
C	-6.00666	-1.09012	1.49139
C	-5.31112	-1.03140	0.12750
C	-3.91953	-0.38812	0.19528
C	-3.23283	-0.33421	-1.17480
H	-1.40078	0.28073	-2.17624
H	2.52051	-2.26264	0.73790
H	4.96152	-2.70180	0.77570
H	6.56426	-0.88147	0.21945
H	5.70962	1.37432	-0.38744
H	3.26811	1.80522	-0.44838
H	-6.99576	-1.55519	1.41254
H	-5.41883	-1.67365	2.21048
H	-6.14186	-0.08554	1.91052
H	-5.93670	-0.46981	-0.58034
H	-5.21963	-2.04701	-0.28236
H	-3.28945	-0.95142	0.89565
H	-4.01106	0.62890	0.60254
H	-3.82641	0.26726	-1.87597
H	-3.16538	-1.34054	-1.60709

Revised structure of 240{19j}, i.e. zwitterion 244{19j-rev} (CDCl₃)

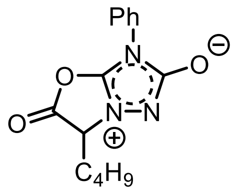

	Conf1	Conf2	Conf3
Rel energy (kcal/mol):	0.00	0.22	0.38

C-nom	iGau	Exp	Calc	diff	1	2	3
C-C	8	173.80	173.56	-0.24	[173.47	173.73	173.52]
C-C	3	154.90	154.34	-0.56	[154.27	154.45	154.34]
C-C	5	152.70	151.81	-0.89	[151.93	152.13	151.16]
C-C	11	130.70	132.34	1.64	[132.38	132.28	132.36]
C-CH	13	129.20	128.54	-0.66	[128.54	128.54	128.54]
C-CH	15	129.20	128.54	-0.66	[128.54	128.54	128.54]
C-CH	14	128.60	127.41	-1.19	[127.41	127.41	127.39]
C-CH	12	125.70	124.29	-1.41	[124.19	124.43	124.28]
C-CH	16	125.70	124.29	-1.41	[124.19	124.43	124.28]
C-CH	9	58.20	61.23	3.03	[61.48	61.37	60.56]
C-CH2	20	29.50	31.93	2.43	[33.75	29.65	31.44]
C-CH2	19	28.00	26.57	-1.43	[28.22	24.47	26.19]
C-CH2	18	21.90	22.87	0.97	[23.07	22.62	22.82]
C-CH3	17	13.70	13.55	-0.15	[13.58	13.47	13.59]

13C chem shifts: RMSD=1.42ppm (MAE=1.19) N=14 {-1.43 3.03}

Fractions: 0.452 0.311 0.237

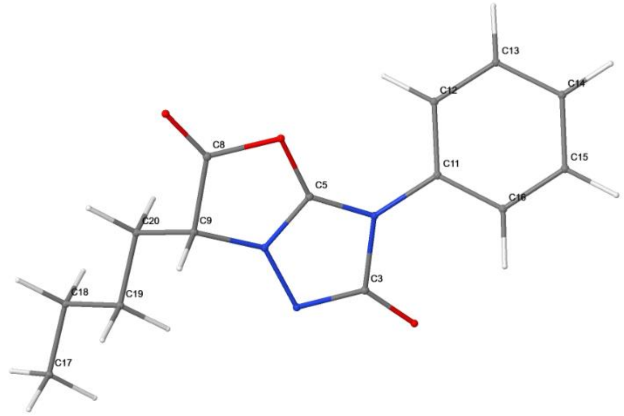

Conformer 1
Energy: -932.41580 Hartree (Rel: 0.0 kcal/mol)
XYZ coordinates for conf 1:

N	0.81846	-0.08790	-0.45724
N	0.74165	-1.46479	-0.58335
C	-0.56602	-1.74984	-0.39702
N	-1.30141	-0.43598	-0.18229
C	-0.34456	0.48192	-0.25267
O	-1.15393	-2.82272	-0.38198
O	-0.32568	1.83090	-0.25783
C	1.02895	2.18676	-0.54512
C	1.85478	0.89728	-0.71895
O	1.36380	3.32619	-0.59562
C	-2.68459	-0.24926	0.09798
C	-3.09713	0.81845	0.90516
C	-4.45634	1.00141	1.15702
C	-5.39841	0.11769	0.62530
C	-4.97387	-0.95135	-0.16696
C	-3.61917	-1.14102	-0.44055
C	6.06845	-1.62283	0.70795

```
C          5.16513      -0.40708       0.94012
C          3.95874      -0.37588      -0.00770
C          3.06861       0.85228       0.22427
H          2.18220       0.83313      -1.76487
H         -2.36734       1.49773       1.33369
H         -4.77479       1.83276       1.77908
H         -6.45544       0.25992       0.83002
H         -5.70005      -1.64408      -0.58230
H         -3.27958      -1.97051      -1.04654
H          6.91850      -1.62246       1.39944
H          5.51661      -2.55899       0.85542
H          6.46800      -1.63004      -0.31355
H          5.74962       0.51543       0.81622
H          4.80852      -0.40811       1.97951
H          3.36345      -1.28827       0.11877
H          4.31051      -0.37468      -1.04929
H          3.63704       1.77477       0.05380
H          2.71114       0.87783       1.26106

Conformer 2
Energy: -932.41544 Hartree (Rel:  0.2 kcal/mol)
XYZ coordinates for conf 2:
N         -0.80857       0.73631      -0.93554
N         -0.45539       2.05737      -0.72912
C          0.82104       1.99905      -0.28844
N          1.23824       0.53526      -0.26408
C          0.15700      -0.11216      -0.68269
O          1.58903       2.88772       0.05371
O         -0.13348      -1.40095      -0.95939
C         -1.46455      -1.38122      -1.48375
C         -1.96007       0.07892      -1.52558
O         -2.01124      -2.38811      -1.80020
C          2.47953      -0.00622       0.17436
C          2.51609      -1.25944       0.79853
C          3.74127      -1.78591       1.20637
C          4.92044      -1.06269       1.01235
C          4.86977       0.19218       0.40112
C          3.65436       0.72738      -0.02592
C         -4.63040      -0.12175       2.86522
C         -4.63502       0.20738       1.36898
C         -3.27626      -0.04377       0.70008
C         -3.29496       0.29774      -0.79427
H         -2.06189       0.36883      -2.57933
H          1.60035      -1.81706       0.96621
H          3.76861      -2.76014       1.68559
H          5.87152      -1.47349       1.33821
H          5.78213       0.76137       0.24842
H          3.60430       1.70143      -0.49441
H         -5.61135       0.06860       3.31468
H         -4.38220      -1.17607       3.03857
H         -3.89272       0.48704       3.40194
H         -4.91737       1.25928       1.22481
H         -5.40465      -0.39113       0.86228
H         -2.99705      -1.09838       0.83976
H         -2.50862       0.55812       1.20373
H         -3.57062       1.34843      -0.93989
H         -4.04440      -0.30559      -1.32045

Conformer 3
Energy: -932.41519 Hartree (Rel:  0.4 kcal/mol)
XYZ coordinates for conf 3:
N         -0.58004       0.79976      -0.35508
N         -0.15917       2.10908      -0.21651
C          1.17608       2.01424      -0.02905
N          1.55014       0.53968      -0.08431
C          0.39084      -0.07607      -0.28844
O          2.01894       2.88132       0.15726
O          0.01318      -1.35436      -0.49748
C         -1.39383      -1.29483      -0.75760
C         -1.84960       0.17679      -0.67977
O         -2.01069      -2.28859      -0.97183
```

C	2.83674	-0.03888	0.10633
C	2.95603	-1.30129	0.70112
C	4.22110	-1.86486	0.86413
C	5.36191	-1.16986	0.45595
C	5.23137	0.09452	-0.12299
C	3.97253	0.66685	-0.30665
C	-6.80598	-0.28796	0.52447
C	-5.42594	0.22098	0.95344
C	-4.33270	-0.08741	-0.07885
C	-2.95878	0.43650	0.35781
H	-2.18324	0.48911	-1.67782
H	2.07360	-1.83749	1.03500
H	4.31139	-2.84612	1.32074
H	6.34564	-1.60965	0.59143
H	6.11376	0.64224	-0.44097
H	3.86134	1.64879	-0.74723
H	-7.56524	-0.05639	1.27975
H	-7.12213	0.17236	-0.41960
H	-6.79937	-1.37505	0.37868
H	-5.15131	-0.22997	1.91706
H	-5.46936	1.30574	1.12266
H	-4.60303	0.36590	-1.04328
H	-4.28238	-1.17026	-0.24664
H	-2.66110	-0.00125	1.31833
H	-2.99521	1.52304	0.50020

Originally proposed (incorrect) structure of 241{19q} ($CDCl_3$)

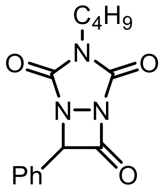

	Conf1
Rel energy (kcal/mol):	0.00

C-nom	iGau	Exp	Calc	diff	1
C-C	3	172.00	161.91	-10.09	[161.91]
C-C	7	156.00	160.02	4.02	[160.02]
C-C	5	154.20	149.38	-4.82	[149.38]
C-C	11	129.50	128.64	-0.86	[128.64]
C-CH	14	131.90	131.98	0.08	[131.98]
C-CH	12	129.00	129.66	0.66	[129.66]
C-CH	16	129.00	129.66	0.66	[129.66]
C-CH	13	128.80	128.29	-0.51	[128.29]
C-CH	15	128.80	128.29	-0.51	[128.29]
C-CH	2	62.10	80.83	18.73	[80.83]
C-CH2	20	39.30	41.54	2.24	[41.54]
C-CH2	19	29.60	30.49	0.89	[30.49]
C-CH2	18	19.50	20.14	0.64	[20.14]
C-CH3	17	13.40	13.43	0.03	[13.43]

13C chem shifts: RMSD=5.98ppm (MAE=3.20) N=14 {-10.09 18.73}

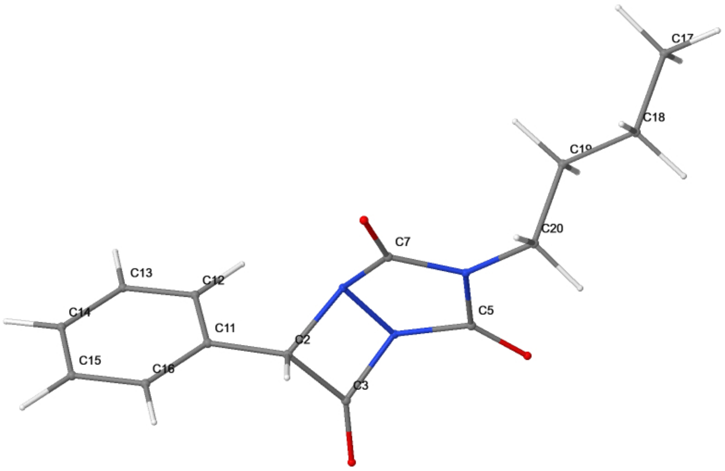

Conformer 1
Energy: -932.43023 Hartree (Rel: 0.0 kcal/mol)
XYZ coordinates for conf 1:

N	0.36124	-0.15283	-0.07755
C	1.60873	0.35139	0.60067
C	1.21946	1.74115	0.02600
N	0.05076	1.17043	-0.59111
C	-1.31492	1.46375	-0.31593
N	-1.79261	0.32156	0.32005
C	-0.77707	-0.59115	0.62836
O	-0.86585	-1.57891	1.32518
O	-1.90967	2.48246	-0.57923
O	1.64581	2.85586	0.03088
C	2.91196	-0.26768	0.17979
C	3.09866	-0.74047	-1.12594
C	4.33126	-1.26963	-1.50689
C	5.38708	-1.32287	-0.59207

C	5.20439	-0.85101	0.70925
C	3.96873	-0.32883	1.09581
C	-6.33643	-1.54779	-0.83098
C	-5.47804	-0.78559	0.18388
C	-4.02712	-0.60890	-0.28295
C	-3.18448	0.15368	0.74507
H	1.49759	0.35334	1.69063
H	2.27617	-0.70676	-1.83416
H	4.46751	-1.64045	-2.51875
H	6.34628	-1.73542	-0.89186
H	6.01831	-0.89646	1.42707
H	3.82543	0.02931	2.11214
H	-7.36581	-1.65990	-0.47279
H	-6.37191	-1.02229	-1.79296
H	-5.93436	-2.55159	-1.01423
H	-5.48766	-1.31716	1.14555
H	-5.92193	0.20173	0.37247
H	-4.00561	-0.06733	-1.23763
H	-3.57120	-1.59156	-0.46082
H	-3.15914	-0.37756	1.70047
H	-3.58486	1.15641	0.91595

Revised structure of 241{19q}, i.e. zwitterion 245{19q-rev} ($CDCl_3$)

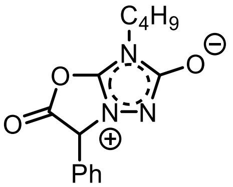

					Conf1	Conf2	Conf3
Rel energy (kcal/mol):					0.00	0.01	0.01

C-nom	iGau	Exp	Calc	diff	1	2	3
C-C	15	172.00	172.68	0.68	[172.69	172.68	172.68]
C-C	3	156.00	155.30	-0.70	[155.41	155.24	155.24]
C-C	5	154.20	153.55	-0.65	[153.46	153.61	153.59]
C-C	13	129.50	128.19	-1.31	[128.21	128.18	128.18]
C-CH	10	131.90	131.74	-0.16	[131.69	131.77	131.77]
C-CH	9	129.00	128.52	-0.48	[128.51	128.53	128.53]
C-CH	11	129.00	128.52	-0.48	[128.51	128.53	128.53]
C-CH	8	128.80	128.36	-0.44	[128.29	128.40	128.40]
C-CH	12	128.80	128.36	-0.44	[128.29	128.40	128.40]
C-CH	16	62.10	63.70	1.60	[63.67	63.71	63.71]
C-CH2	20	39.30	41.08	1.78	[41.34	40.95	40.95]
C-CH2	19	29.60	32.05	2.45	[32.19	31.98	31.98]
C-CH2	18	19.50	20.19	0.69	[20.21	20.18	20.18]
C-CH3	17	13.40	13.38	-0.02	[13.36	13.39	13.39]

13C chem shifts: RMSD=1.07ppm (MAE=0.85) N=14 {-1.31 2.45}

Fractions: 0.337 0.332 0.332

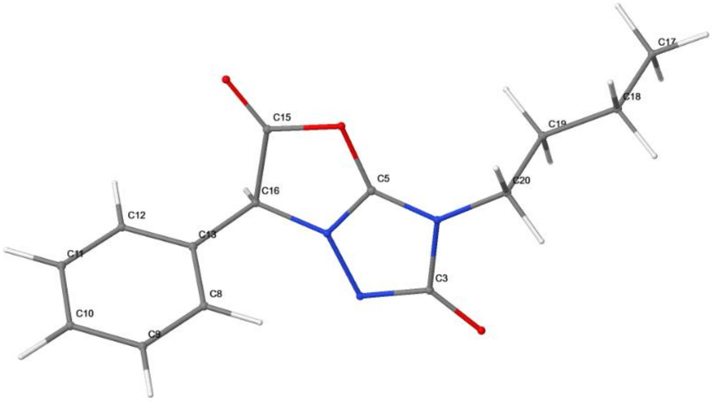

Conformer 1
Energy: -932.41422 Hartree (Rel: 0.0 kcal/mol)
XYZ coordinates for conf 1:

N	-0.39991	0.33230	1.03100
N	-0.04037	1.63927	1.33490
C	1.31329	1.62844	1.25001
N	1.75201	0.24551	0.90067
C	0.63169	-0.44492	0.79413
O	0.33193	-1.73965	0.56255
O	2.13559	2.52517	1.41178
C	-3.99317	-0.47966	0.15146
C	-4.97840	-0.10273	-0.76092
C	-4.67872	0.80505	-1.78038
C	-3.39116	1.33464	-1.88342
C	-2.39829	0.95432	-0.97761
C	-2.69996	0.04535	0.04087
O	-1.65619	-2.85581	0.54731
C	-1.08640	-1.82411	0.69439
C	-1.64123	-0.41147	1.03205
C	5.47141	-0.44722	-2.43808
C	5.03129	-0.49659	-0.97121

```
C          3.56001       -0.10560       -0.78128
C          3.13993       -0.15851        0.69211
H         -4.22910       -1.18203        0.94629
H         -5.97999       -0.51306       -0.67091
H         -5.44818        1.10305       -2.48684
H         -3.15525        2.04799       -2.66775
H         -1.40460        1.38399       -1.04791
H         -2.06250       -0.45863        2.04468
H          6.52466       -0.72984       -2.54399
H          4.87710       -1.13368       -3.05340
H          5.35174        0.56089       -2.85283
H          5.66398        0.17510       -0.37506
H          5.19356       -1.50792       -0.57291
H          2.91785       -0.77726       -1.36722
H          3.39042        0.91095       -1.15797
H          3.73135        0.54327        1.28609
H          3.27432       -1.16212        1.10787

Conformer 2
Energy: -932.41421 Hartree (Rel:  0.0 kcal/mol)
XYZ coordinates for conf 2:
N          0.33531        0.35075       -0.63994
N          0.06467        1.65824       -1.02355
C         -1.16948        1.89405       -0.51331
N         -1.62762        0.66417        0.19935
C         -0.63946       -0.19817        0.04718
O         -0.42783       -1.47733        0.41948
O         -1.87923        2.89432       -0.55799
C          3.92184       -0.85410       -0.68981
C          5.17016       -0.51299       -0.16953
C          5.28445        0.53770        0.74474
C          4.14662        1.24553        1.13645
C          2.89298        0.90268        0.62524
C          2.78071       -0.14863       -0.28973
O          1.32007       -2.89424        0.04835
C          0.85476       -1.81295       -0.10932
C          1.43076       -0.57159       -0.84776
C         -6.34151       -1.10282       -0.16267
C         -5.30979       -0.34880        0.68278
C         -3.94376       -0.24229       -0.00745
C         -2.92561        0.51571        0.85247
H          3.83552       -1.66799       -1.40468
H          6.05239       -1.06302       -0.48380
H          6.25784        0.80745        1.14438
H          4.23090        2.06927        1.83932
H          2.01460        1.46933        0.91568
H          1.52300       -0.83381       -1.90936
H         -7.30614       -1.16385        0.35324
H         -6.50696       -0.60179       -1.12404
H         -6.00901       -2.12664       -0.37313
H         -5.18973       -0.85320        1.65171
H         -5.68291        0.66044        0.90425
H         -4.04911        0.27679       -0.96857
H         -3.55963       -1.24801       -0.22613
H         -2.77582        0.02141        1.81777
H         -3.26147        1.53949        1.03781

Conformer 3
Energy: -932.41421 Hartree (Rel:  0.0 kcal/mol)
XYZ coordinates for conf 3:
N         -0.33504       -0.35022       -0.64044
N         -0.06455       -1.65763       -1.02389
C          1.16946       -1.89367       -0.51311
N          1.62734       -0.66400        0.19989
C          0.63936        0.19851        0.04730
O          0.42758        1.47766        0.41979
O          1.87908       -2.89400       -0.55768
C         -2.89284       -0.90423        0.62336
C         -4.14640       -1.24746        1.13457
C         -5.28413       -0.53851        0.74460
C         -5.16982        0.51373       -0.16791
```

C	-3.92161	0.85525	-0.68817
C	-2.78057	0.14861	-0.28984
O	-1.32028	2.89452	0.04835
C	-0.85494	1.81326	-0.10932
C	-1.43073	0.57191	-0.84792
C	6.34149	1.10217	-0.16295
C	5.30968	0.34860	0.68281
C	3.94366	0.24193	-0.00740
C	2.92537	-0.51547	0.85291
H	-2.01458	-1.47176	0.91239
H	-4.23067	-2.07240	1.83602
H	-6.25745	-0.80853	1.14423
H	-6.05197	1.06468	-0.48080
H	-3.83528	1.67035	-1.40165
H	-1.52312	0.83433	-1.90947
H	7.30612	1.16331	0.35294
H	6.50688	0.60074	-1.12412
H	6.00909	2.12594	-0.37381
H	5.18967	0.85342	1.65152
H	5.68271	-0.66058	0.90471
H	4.04893	-0.27765	-0.96825
H	3.55966	1.24758	-0.22659
H	2.77567	-0.02064	1.81794
H	3.26105	-1.53920	1.03880

Originally proposed (incorrect) structure of 242{19r} ($CDCl_3$)

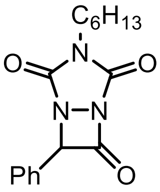

					Conf1
	Rel energy (kcal/mol):				0.00
**C-nom**	**iGau**	**Exp**	**Calc**	**diff**	**1**
C-C	3	172.10	161.92	-10.18	[161.92]
C-C	7	155.90	160.10	4.20	[160.10]
C-C	5	154.30	149.42	-4.88	[149.42]
C-C	11	129.40	128.60	-0.80	[128.60]
C-CH	14	132.00	131.89	-0.11	[131.89]
C-CH	12	128.90	129.47	0.57	[129.47]
C-CH	16	128.90	129.47	0.57	[129.47]
C-CH	13	128.80	128.28	-0.52	[128.28]
C-CH	15	128.80	128.28	-0.52	[128.28]
C-CH	2	62.10	80.87	18.77	[80.87]
C-CH2	22	39.60	41.60	2.00	[41.60]
C-CH2	19	31.10	31.76	0.66	[31.76]
C-CH2	21	27.50	29.55	2.05	[29.55]
C-CH2	20	25.90	27.61	1.71	[27.61]
C-CH2	18	22.40	23.53	1.13	[23.53]
C-CH3	17	13.90	13.91	0.01	[13.91]

13C chem shifts: RMSD=5.66ppm (MAE=3.04) N=16 {-10.18 18.77}

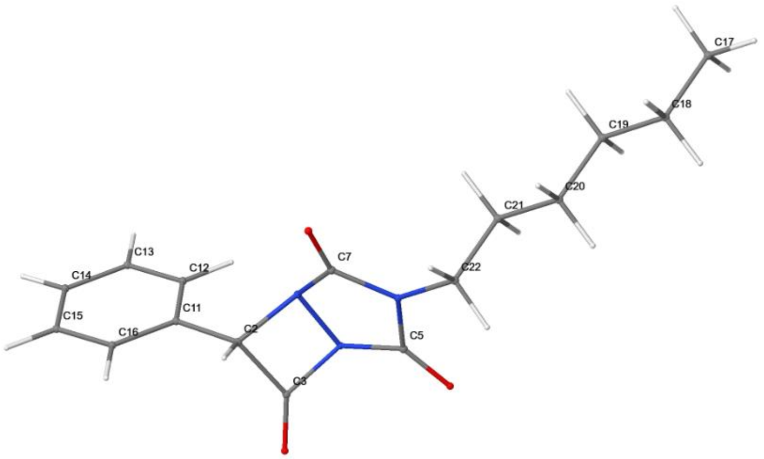

Conformer 1
Energy: -1011.05643 Hartree (Rel: 0.0 kcal/mol)
XYZ coordinates for conf 1:

N	1.20073	-0.07429	-0.03988
C	2.52053	0.35980	0.54213
C	2.25668	1.71536	-0.16955
N	1.00896	1.21403	-0.68388
C	-0.30463	1.68223	-0.39728
N	-0.87208	0.67133	0.37306
C	0.05373	-0.31047	0.74448
O	-0.10795	-1.20536	1.54577
O	-0.79944	2.72735	-0.74897
O	2.79822	2.77083	-0.29999
C	3.73380	-0.43909	0.15567
C	3.80225	-1.10048	-1.07753
C	4.95649	-1.79837	-1.43192
C	6.05183	-1.83374	-0.56412

C	5.98714	-1.17360	0.66461
C	4.82964	-0.48203	1.02596
C	-8.08149	-1.49114	-0.77097
C	-7.09391	-0.72937	0.11939
C	-5.65281	-0.77130	-0.40587
C	-4.65958	-0.01129	0.48303
C	-3.22110	-0.05683	-0.04868
C	-2.25279	0.70583	0.86194
H	2.45371	0.49042	1.62803
H	2.94840	-1.08006	-1.74799
H	5.00041	-2.31507	-2.38642
H	6.94956	-2.37821	-0.84297
H	6.83183	-1.20334	1.34683
H	4.77794	0.02308	1.98722
H	-9.10046	-1.44246	-0.36999
H	-8.10190	-1.07487	-1.78584
H	-7.80526	-2.54976	-0.85294
H	-7.12044	-1.14634	1.13619
H	-7.41523	0.31795	0.21045
H	-5.62472	-0.35242	-1.42255
H	-5.33021	-1.81889	-0.49746
H	-4.68561	-0.43145	1.49899
H	-4.98102	1.03639	0.57455
H	-3.18084	0.37573	-1.05676
H	-2.88668	-1.09895	-0.13264
H	-2.24104	0.27551	1.86711
H	-2.53309	1.75963	0.93884

Revised structure of 242{19r}, i.e. zwitterion 246{19r-rev} ($CDCl_3$)

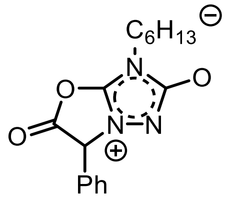

	Conf1
Rel energy (kcal/mol):	0.00

C-nom	iGau	Exp	Calc	diff	1
C-C	15	172.10	172.75	0.65	[172.75]
C-C	3	155.90	155.23	-0.67	[155.23]
C-C	5	154.30	153.66	-0.64	[153.66]
C-C	13	129.40	128.20	-1.20	[128.20]
C-CH	10	132.00	131.73	-0.27	[131.73]
C-CH	9	128.90	128.51	-0.39	[128.51]
C-CH	11	128.90	128.51	-0.39	[128.51]
C-CH	8	128.80	128.32	-0.48	[128.32]
C-CH	12	128.80	128.32	-0.48	[128.32]
C-CH	16	62.10	63.58	1.48	[63.58]
C-CH2	22	39.60	40.80	1.20	[40.80]
C-CH2	19	31.10	31.77	0.67	[31.77]
C-CH2	21	27.50	30.89	3.39	[30.89]
C-CH2	20	25.90	27.70	1.80	[27.70]
C-CH2	18	22.40	23.50	1.10	[23.50]
C-CH3	17	13.90	13.86	-0.04	[13.86]

13C chem shifts: RMSD=1.21ppm (MAE=0.93) N=16 {-1.20 3.39}

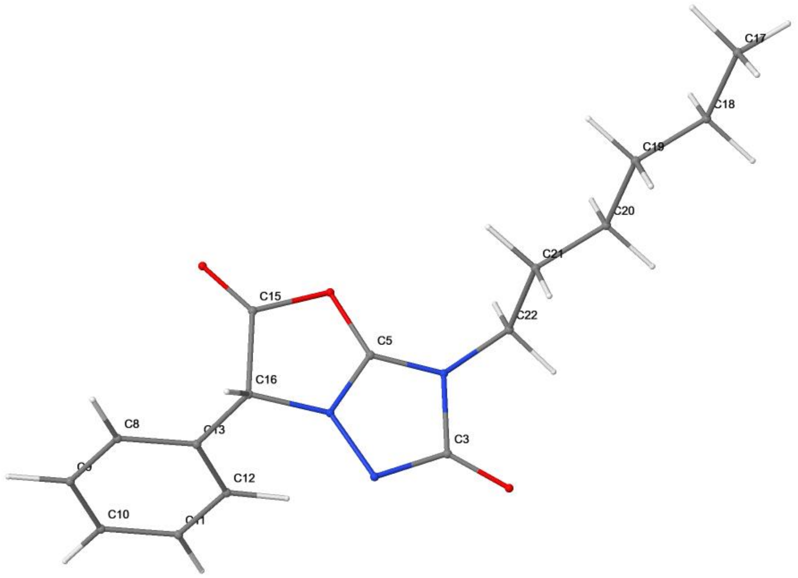


```
Conformer 1
Energy: -1011.04038 Hartree (Rel:  0.0 kcal/mol)
XYZ coordinates for conf 1:
N          1.22003       -0.42459       -0.66598
N          1.08139       -1.75417       -1.04405
C         -0.12933       -2.10602       -0.54465
N         -0.71264       -0.92210        0.15502
C          0.18954        0.03071        0.00834
O          0.27415        1.32533        0.37774
O         -0.73732       -3.17117       -0.58978
C          4.66984        1.13497       -0.64235
C          5.93668        0.91762       -0.10120
```

C	6.14369	-0.13316	0.79666
C	5.08000	-0.96517	1.15047
C	3.80706	-0.74664	0.61867
C	3.60210	0.30518	-0.27944
O	1.88250	2.90169	0.01990
C	1.52512	1.77994	-0.13711
C	2.22696	0.59615	-0.86085
C	-8.02259	1.16419	-0.29457
C	-6.93508	0.45114	0.51611
C	-5.55740	0.48987	-0.15845
C	-4.46470	-0.22020	0.65160
C	-3.09131	-0.18451	-0.03133
C	-2.02187	-0.90007	0.80234
H	4.51111	1.94936	-1.34398
H	6.76151	1.56431	-0.38594
H	7.13205	-0.30621	1.21277
H	5.23739	-1.78942	1.84004
H	2.98847	-1.40896	0.87973
H	2.31112	0.86338	-1.92186
H	-8.99345	1.11758	0.21205
H	-7.77491	2.22242	-0.44473
H	-8.14089	0.70806	-1.28531
H	-7.22751	-0.59564	0.68034
H	-6.86262	0.90796	1.51339
H	-5.26419	1.53696	-0.32523
H	-5.62815	0.02932	-1.15473
H	-4.75831	-1.26621	0.82031
H	-4.39047	0.24335	1.64617
H	-2.78623	0.85738	-0.19904
H	-3.14941	-0.66398	-1.01666
H	-2.27713	-1.95426	0.93899
H	-1.91658	-0.44006	1.79044

Originally proposed correct structure of 247{3} ($CDCl_3$)

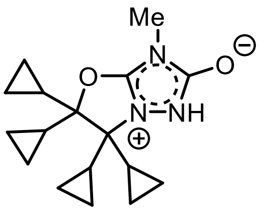

	Conf1	Conf2	Conf3
Rel energy (kcal/mol):	0.00	0.00	0.95

C-nom	iGau	Exp	Calc	diff	1	2	3
C-C	3	159.18	158.98	-0.20	[159.00	159.00	158.81]
C-C	5	150.90	151.57	0.67	[151.47	151.47	152.59]
C-C	8	107.34	107.67	0.33	[108.02	108.03	104.20]
C-C	9	73.34	75.79	2.45	[75.75	75.75	76.17]
C-CH	13	14.89	15.22	0.33	[12.67	16.37	22.14]
C-CH	12	14.89	14.99	0.10	[11.51	17.37	20.46]
C-CH	10	13.86	14.92	1.06	[16.37	12.67	18.92]
C-CH	11	13.86	14.85	0.99	[17.37	11.51	18.87]
C-CH2	14	2.79	3.21	0.42	[3.43	3.18	2.25]
C-CH2	19	2.79	3.21	0.42	[3.18	3.43	2.23]
C-CH2	20	2.45	2.70	0.25	[4.20	1.10	3.24]
C-CH2	17	2.45	2.62	0.17	[1.10	4.20	2.31]
C-CH2	18	2.31	2.43	0.12	[4.20	0.31	4.21]
C-CH2	15	2.31	2.31	0.00	[0.31	4.20	2.89]
C-CH2	16	2.22	2.23	0.01	[3.93	0.06	4.52]
C-CH2	21	2.22	2.03	-0.19	[0.05	3.93	2.38]
C-CH3	22	26.35	24.21	-2.14	[24.21	24.21	24.20]

13C chem shifts: RMSD=0.90ppm (MAE=0.58) N=17 {-2.14 2.45}

Fractions: 0.454 0.454 0.092

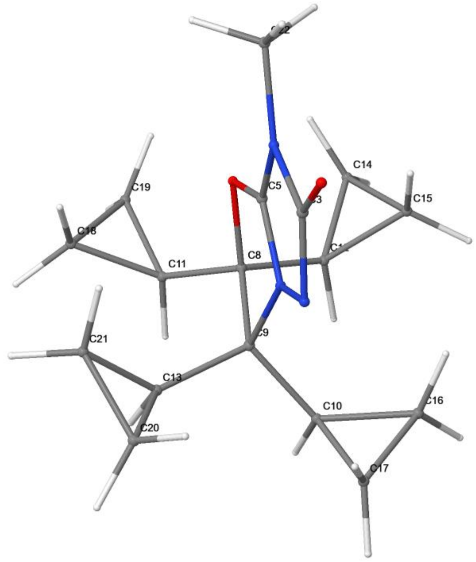

Conformer 1
Energy: -976.17898 Hartree (Rel: 0.0 kcal/mol)
XYZ coordinates for conf 1:

N	1.02282	0.39375	0.26891
N	2.20602	1.05533	0.60924
C	3.17909	0.17005	0.29636
N	2.53972	-1.04485	-0.26506
C	1.23474	-0.79426	-0.24496
O	4.40302	0.24405	0.41729

O	0.17261	-1.47258	-0.64665
C	-1.01910	-0.65345	-0.18243
C	-0.38119	0.81508	0.14316
C	-0.88245	1.37177	1.47378
C	-1.97378	-0.63874	-1.35574
C	-1.56551	-1.35362	1.05401
C	-0.55706	1.79860	-1.01906
C	-1.56743	-2.85858	1.19290
C	-0.69218	-2.02376	2.09064
C	-0.87361	2.84230	1.81612
C	-2.18547	2.13048	1.60973
C	-3.00746	-1.71230	-1.60761
C	-3.44955	-0.33323	-1.19742
C	0.42294	2.91606	-1.29546
C	0.30336	1.76677	-2.26226
C	3.25314	-2.23266	-0.69454
H	-0.64714	0.71410	2.30396
H	-1.51653	-0.22774	-2.24922
H	-2.43682	-0.84517	1.45624
H	-1.60114	2.04170	-1.19512
H	-2.45365	-3.32630	1.61288
H	-1.07306	-3.43742	0.41916
H	0.38038	-2.07034	1.93084
H	-0.97680	-1.90129	3.13192
H	-0.57990	3.10874	2.82736
H	-0.56019	3.54932	1.05692
H	-2.75011	2.36611	0.71232
H	-2.80487	1.90757	2.47437
H	-3.15307	-2.01717	-2.64005
H	-3.11655	-2.51087	-0.88299
H	-3.85207	-0.21015	-0.19646
H	-3.90322	0.31766	-1.93953
H	0.01321	3.88126	-1.58157
H	1.32110	2.95539	-0.68802
H	1.13440	1.06815	-2.30595
H	-0.18870	1.93019	-3.21734
H	4.31167	-2.01710	-0.53733
H	2.96376	-3.10337	-0.09872
H	3.07341	-2.43773	-1.75375

Conformer 2
Energy: -976.18049 Hartree (Rel: 0.0 kcal/mol)
XYZ coordinates for conf 2:

N	-1.02280	0.39380	0.26882
N	-2.20593	1.05539	0.60932
C	-3.17909	0.17017	0.29649
N	-2.53975	-1.04473	-0.26523
C	-1.23476	-0.79415	-0.24516
O	-4.40299	0.24419	0.41758
O	-0.17264	-1.47251	-0.64678
C	1.01909	-0.65345	-0.18242
C	0.38121	0.81509	0.14313
C	0.55717	1.79858	-1.01909
C	1.56531	-1.35369	1.05408
C	1.97390	-0.63878	-1.35562
C	0.88243	1.37178	1.47377
C	3.44965	-0.33333	-1.19704
C	3.00758	-1.71238	-1.60732
C	-0.30311	1.76663	-2.26239
C	-0.42290	2.91594	-1.29563
C	0.69175	-2.02383	2.09054
C	1.56710	-2.85866	1.19290
C	2.18552	2.13038	1.60979
C	0.87371	2.84234	1.81600
C	-3.25312	-2.23252	-0.69479
H	1.60126	2.04175	-1.19501
H	2.43657	-0.84529	1.45646
H	1.51679	-0.22774	-2.24916
H	0.64697	0.71420	2.30396
H	3.90351	0.31755	-1.93905
H	3.85199	-0.21028	-0.19601

H	3.11652	-2.51098	-0.88272
H	3.15338	-2.01721	-2.63976
H	0.18905	1.93011	-3.21741
H	-1.13407	1.06793	-2.30619
H	-1.32112	2.95523	-0.68826
H	-0.01322	3.88116	-1.58174
H	0.97620	-1.90144	3.13187
H	-0.38079	-2.07031	1.93053
H	1.07285	-3.43744	0.41904
H	2.45322	-3.32646	1.61300
H	2.80481	1.90748	2.47452
H	2.75028	2.36587	0.71242
H	0.56042	3.54935	1.05674
H	0.57994	3.10887	2.82720
H	-2.96389	-3.10326	-0.09894
H	-4.31168	-2.01693	-0.53776
H	-3.07327	-2.43758	-1.75399

Conformer 3
Energy: -976.18049 Hartree (Rel: 0.9 kcal/mol)
XYZ coordinates for conf 3:

N	0.94111	0.21069	0.19819
N	2.31709	0.46623	0.26902
C	2.89059	-0.74547	0.09240
N	1.81224	-1.74041	-0.13007
C	0.68131	-1.04948	-0.06680
O	4.07390	-1.08421	0.10211
O	-0.58656	-1.37034	-0.26999
C	-1.35905	-0.12073	0.04433
C	-0.23947	1.05952	-0.01591
C	-0.43525	2.11570	1.07052
C	-2.46420	0.01318	-0.98794
C	-1.96673	-0.30567	1.43120
C	-0.19118	1.76046	-1.38035
C	-2.54248	-1.62782	1.87913
C	-1.31625	-1.06852	2.55751
C	0.07209	1.98883	2.48402
C	0.70021	2.99167	1.54956
C	-2.34178	-0.43273	-2.42173
C	-3.24792	-1.18218	-1.47749
C	1.03650	2.48507	-1.87856
C	0.55816	1.23764	-2.58053
C	2.02742	-3.15966	-0.34283
H	-1.38808	2.63019	0.95470
H	-3.05322	0.91282	-0.81758
H	-2.53480	0.57023	1.73343
H	-1.13365	2.25462	-1.60317
H	-3.49051	-1.61268	2.40951
H	-2.40049	-2.49155	1.23776
H	-0.37220	-1.57371	2.37690
H	-1.40895	-0.65561	3.55780
H	-0.57176	2.33998	3.28610
H	0.66409	1.11417	2.73392
H	1.69885	2.76475	1.19242
H	0.49145	4.04845	1.69304
H	-2.78391	0.20016	-3.18617
H	-1.42279	-0.92423	-2.72295
H	-2.92667	-2.16900	-1.16027
H	-4.32381	-1.07190	-1.57983
H	0.88866	3.44728	-2.36179
H	1.94850	2.39586	-1.29893
H	1.17107	0.34742	-2.46658
H	0.07876	1.32382	-3.55164
H	3.10896	-3.30448	-0.31300
H	1.55578	-3.75127	0.44702
H	1.64112	-3.47354	-1.31670

Originally proposed correct structure of 248{4} ($CDCl_3$)

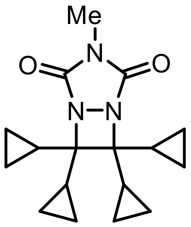

	Conf1	Conf2	Conf3	Conf4	Conf5	Conf6
Rel energy (kcal/mol):	0.00	0.00	0.00	0.03	1.86	1.89

C-nom	iGau	Exp	Calc	diff	1	2	3	4	5	6
C-C	3	160.67	160.73	0.06	[161.31	159.95	161.31	160.45	159.44	159.42]
C-C	5	160.67	160.48	-0.19	[159.95	161.31	159.95	160.84	159.07	159.43]
C-C	21	79.98	80.06	0.08	[80.95	79.14	80.95	79.10	80.58	80.70]
C-C	20	79.98	80.06	0.08	[79.14	80.95	79.14	80.98	80.69	80.70]
C-CH	17	12.07	12.86	0.79	[17.82	14.15	10.60	8.21	17.10	18.97]
C-CH	16	12.07	12.84	0.77	[14.15	17.82	8.19	10.65	19.04	17.05]
C-CH	18	12.07	12.78	0.71	[10.60	8.19	17.82	14.14	18.90	17.05]
C-CH	19	12.07	12.73	0.66	[8.19	10.60	14.15	17.79	16.98	18.97]
C-CH2	9	3.86	3.34	-0.52	[4.39	3.38	2.71	2.67	5.41	5.07]
C-CH2	11	3.86	2.84	-1.02	[3.86	2.98	2.48	2.03	3.25	2.12]
C-CH2	15	3.86	3.33	-0.53	[3.38	4.39	2.67	2.68	5.09	5.42]
C-CH2	13	3.86	2.84	-1.02	[2.98	3.86	2.03	2.48	2.14	3.23]
C-CH2	12	2.49	3.32	0.83	[2.71	2.67	4.39	3.37	5.07	5.42]
C-CH2	10	2.49	3.32	0.83	[2.67	2.71	3.38	4.39	5.41	5.07]
C-CH2	14	2.49	2.83	0.34	[2.48	2.03	3.86	2.97	2.15	3.23]
C-CH2	8	2.49	2.83	0.34	[2.03	2.48	2.98	3.88	3.27	2.12]
C-CH3	22	25.84	25.42	-0.42	[25.41	25.41	25.41	25.44	25.25	25.76]

13C chem shifts: RMSD=0.62ppm (MAE=0.54) N=17 {-1.02 0.83}

	1	2	3	4	5	6
Fractions:	0.248	0.248	0.247	0.236	0.011	0.010

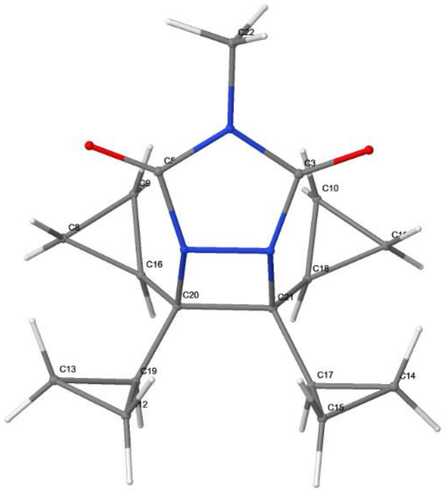

Conformer 1
Energy: -976.18926 Hartree (Rel: 0.0 kcal/mol)
XYZ coordinates for conf 1:

N	0.52821	-0.85131	-0.67272
N	0.68027	0.55937	-0.85314
C	2.02168	0.88030	-0.66916
N	2.66218	-0.34909	-0.47943
C	1.75076	-1.40175	-0.31771
O	2.56272	1.97410	-0.72339

O	2.00092	-2.55389	-0.00317
C	-1.89680	-1.38971	2.39454
C	-1.10093	-2.59419	1.96724
C	0.76127	1.69469	2.11392
C	0.31557	2.93624	1.38673
C	-1.84891	-1.55516	-2.20234
C	-1.72008	-2.74954	-1.29707
C	-2.91952	2.10591	-0.57660
C	-1.85023	3.00745	-1.13892
C	-0.67378	-1.21648	1.51625
C	-1.60305	1.51898	-1.03826
C	-0.45619	1.64444	1.22047
C	-1.90221	-1.36866	-0.70446
C	-0.76968	-0.72568	0.08159
C	-0.59943	0.88302	-0.09020
C	4.10242	-0.49383	-0.36183
H	-1.82314	-1.04611	3.42268
H	-2.87936	-1.25260	1.95239
H	-1.54937	-3.26193	1.24113
H	-0.46806	-3.08629	2.70022
H	0.58704	1.61965	3.18398
H	1.68948	1.23450	1.79056
H	0.93975	3.26310	0.56191
H	-0.15410	3.73623	1.95304
H	-2.77487	-1.41715	-2.75402
H	-0.95485	-1.23057	-2.72583
H	-0.73497	-3.19996	-1.20575
H	-2.54711	-3.45098	-1.22774
H	-3.79871	1.89270	-1.17844
H	-3.12052	2.17178	0.48874
H	-1.33974	3.67012	-0.45006
H	-1.98768	3.41552	-2.13650
H	0.19402	-0.82609	2.03670
H	-1.65289	0.99597	-1.98612
H	-1.39249	1.60329	1.77259
H	-2.88524	-1.14657	-0.29724
H	4.47422	0.04320	0.51555
H	4.31631	-1.55836	-0.25773
H	4.59328	-0.10010	-1.25564

Conformer 2
Energy: -976.19222 Hartree (Rel: 0.0 kcal/mol)
XYZ coordinates for conf 2:

N	0.68014	0.55959	-0.85309
N	0.52840	-0.85114	-0.67283
C	1.75111	-1.40132	-0.31795
N	2.66225	-0.34843	-0.47953
C	2.02144	0.88084	-0.66907
O	2.00156	-2.55344	-0.00356
O	2.56222	1.97477	-0.72320
C	-2.92010	2.10511	-0.57678
C	-1.85103	3.00689	-1.13912
C	-1.84877	-1.55582	-2.20210
C	-1.71950	-2.75010	-1.29677
C	0.76064	1.69516	2.11393
C	0.31453	2.93651	1.38665
C	-1.89604	-1.39023	2.39473
C	-1.09974	-2.59443	1.96742
C	-1.60345	1.51850	-1.03835
C	-0.67319	-1.21658	1.51629
C	-1.90190	-1.36922	-0.70422
C	-0.45675	1.64441	1.22041
C	-0.59969	0.88288	-0.09021
C	-0.76945	-0.72588	0.08162
C	4.10253	-0.49283	-0.36195
H	-3.79920	1.89161	-1.17865
H	-3.12116	2.17100	0.48855
H	-1.34074	3.66974	-0.45027
H	-1.98855	3.41486	-2.13672
H	-2.77485	-1.41810	-2.75366
H	-0.95488	-1.23102	-2.72574

H	-0.73426	-3.20026	-1.20556
H	-2.54634	-3.45173	-1.22727
H	0.58638	1.62009	3.18399
H	1.68905	1.23534	1.79063
H	0.93868	3.26353	0.56187
H	-0.15548	3.73637	1.95286
H	-1.82240	-1.04655	3.42285
H	-2.87871	-1.25354	1.95269
H	-1.54800	-3.26239	1.24140
H	-0.46660	-3.08623	2.70035
H	-1.65312	0.99543	-1.98619
H	0.19453	-0.82583	2.03662
H	-2.88494	-1.14736	-0.29688
H	-1.39306	1.60298	1.77249
H	4.31669	-1.55737	-0.25855
H	4.59330	-0.09837	-1.25548
H	4.47415	0.04371	0.51581

Conformer 3
Energy: -976.19218 Hartree (Rel: 0.0 kcal/mol)
XYZ coordinates for conf 3:

N	-0.52837	-0.85117	-0.67274
N	-0.68021	0.55953	-0.85307
C	-2.02155	0.88068	-0.66906
N	-2.66225	-0.34863	-0.47940
C	-1.75102	-1.40144	-0.31777
O	-2.56242	1.97456	-0.72323
O	-2.00135	-2.55356	-0.00331
C	1.71955	-2.74993	-1.29693
C	1.84866	-1.55561	-2.20224
C	1.85084	3.00702	-1.13909
C	2.91995	2.10525	-0.57681
C	1.10028	-2.59431	1.96737
C	1.89640	-1.38998	2.39464
C	-0.31487	2.93641	1.38664
C	-0.76079	1.69500	2.11393
C	1.90195	-1.36905	-0.70436
C	0.45661	1.64444	1.22043
C	1.60331	1.51862	-1.03837
C	0.67346	-1.21654	1.51628
C	0.76952	-0.72581	0.08160
C	0.59962	0.88293	-0.09020
C	-4.10253	-0.49316	-0.36194
H	2.54642	-3.45154	-1.22754
H	0.73433	-3.20012	-1.20560
H	0.95471	-1.23085	-2.72580
H	2.77468	-1.41783	-2.75388
H	1.98835	3.41501	-2.13669
H	1.34055	3.66985	-0.45023
H	3.12103	2.17109	0.48853
H	3.79906	1.89180	-1.17870
H	0.46728	-3.08624	2.70033
H	1.54861	-3.26219	1.24131
H	2.87901	-1.25311	1.95253
H	1.82277	-1.04632	3.42276
H	0.15499	3.73635	1.95287
H	-0.93902	3.26334	0.56183
H	-1.68912	1.23499	1.79066
H	-0.58651	1.62001	3.18399
H	2.88500	-1.14714	-0.29712
H	1.39293	1.60312	1.77250
H	1.65297	0.99556	-1.98621
H	-0.19429	-0.82593	2.03668
H	-4.59324	-0.09961	-1.25591
H	-4.31657	-1.55764	-0.25760
H	-4.47435	0.04416	0.51525

Conformer 4
Energy: -976.18922 Hartree (Rel: 0.0 kcal/mol)
XYZ coordinates for conf 4:

N	0.67795	-0.56081	-0.85342

N	0.53090	0.85031	-0.67357
C	1.75747	1.39305	-0.31800
N	2.66341	0.33858	-0.48067
C	2.01681	-0.89012	-0.67048
O	2.01925	2.54225	-0.00277
O	2.54646	-1.98950	-0.72650
C	0.30401	-2.93507	1.39325
C	0.75670	-1.69325	2.11567
C	-1.09014	2.60165	1.96371
C	-1.89142	1.40146	2.39326
C	-1.85722	-3.00305	-1.13649
C	-2.92455	-2.09966	-0.57329
C	-1.71711	2.75421	-1.29827
C	-1.84462	1.55968	-2.20354
C	-0.46097	-1.63978	1.22275
C	-1.89875	1.37322	-0.70564
C	-0.66944	1.22131	1.51479
C	-1.60764	-1.51493	-1.03641
C	-0.60179	-0.88068	-0.08967
C	-0.76726	0.72908	0.08095
C	4.10310	0.48929	-0.36273
H	-0.17004	-3.72999	1.96305
H	0.92597	-3.26894	0.56965
H	1.68700	-1.23913	1.78981
H	0.58385	-1.61353	3.18562
H	-0.45471	3.09212	2.69556
H	-1.53605	3.27016	1.23676
H	-2.87480	1.26832	1.95167
H	-1.81890	1.05912	3.42190
H	-1.99622	-3.41103	-2.13388
H	-1.34711	-3.66643	-0.44802
H	-3.12436	-2.16491	0.49234
H	-3.80402	-1.88515	-1.17424
H	-2.54499	3.45470	-1.22927
H	-0.73280	3.20641	-1.20690
H	-0.94962	1.23556	-2.72574
H	-2.76980	1.42092	-2.75632
H	-1.39684	-1.59199	1.77510
H	-2.88214	1.15120	-0.29912
H	0.19670	0.82825	2.03594
H	-1.65750	-0.99207	-1.98436
H	4.38776	0.71811	0.66877
H	4.55808	-0.45484	-0.66466
H	4.44594	1.29618	-1.01499

Conformer 5
Energy: -976.19222 Hartree (Rel: 1.9 kcal/mol)
XYZ coordinates for conf 5:

N	-0.71216	-0.19546	-0.69144
N	0.71164	-0.20744	-0.68894
C	1.15227	-1.50664	-0.88055
N	-0.01688	-2.23462	-1.13246
C	-1.17626	-1.48559	-0.88683
O	2.29669	-1.93483	-0.90530
O	-2.33060	-1.88631	-0.91651
C	-2.82772	-0.17304	1.84117
C	-1.61621	-0.96090	2.27484
C	1.62246	-0.95649	2.28152
C	2.84092	-0.19300	1.82452
C	-1.60295	2.50018	-1.54411
C	-2.82193	2.02638	-0.79583
C	2.83738	2.00077	-0.81587
C	1.61917	2.46763	-1.56978
C	-1.44633	0.43009	1.72037
C	1.50081	2.09523	-0.11543
C	1.46770	0.43044	1.71280
C	-1.48477	2.10899	-0.09471
C	-0.80466	0.83898	0.39839
C	0.81742	0.83272	0.39354
C	-0.02141	-3.63076	-1.53411
H	-3.49211	0.23469	2.59840

H	-3.32064	-0.50778	0.93517
H	-1.34119	-1.81612	1.66638
H	-1.42357	-1.09883	3.33520
H	1.44039	-1.07939	3.34561
H	1.32647	-1.81374	1.68585
H	3.31752	-0.54627	0.91677
H	3.52053	0.21317	2.56897
H	-1.51222	3.55508	-1.78817
H	-1.18959	1.82733	-2.28782
H	-3.19591	1.03631	-1.04292
H	-3.59139	2.74254	-0.52095
H	3.60859	2.71868	-0.55060
H	3.20921	1.00687	-1.05042
H	1.20415	1.78602	-2.30464
H	1.53057	3.51948	-1.82732
H	-1.22777	1.18662	2.47334
H	1.39329	2.92481	0.58126
H	1.27029	1.19979	2.45866
H	-1.37583	2.92910	0.61303
H	0.51026	-4.23877	-0.79749
H	-1.06271	-3.94959	-1.59417
H	0.45941	-3.74801	-2.50932

Conformer 6
Energy: -976.19222 Hartree (Rel: 1.9 kcal/mol)
XYZ coordinates for conf 6:

N	0.71192	-0.19872	-0.69337
N	-0.71209	-0.19823	-0.69339
C	-1.16450	-1.49354	-0.88688
N	-0.00080	-2.23134	-1.13873
C	1.16335	-1.49440	-0.88679
O	-2.31394	-1.90763	-0.91696
O	2.31240	-1.90959	-0.91674
C	2.83010	2.01599	-0.80095
C	1.61172	2.48894	-1.55079
C	-1.61052	2.49032	-1.55009
C	-2.82914	2.01738	-0.80063
C	1.61994	-0.96559	2.27400
C	2.83485	-0.18828	1.83094
C	-2.83442	-0.18715	1.83161
C	-1.61975	-0.96512	2.27417
C	1.49316	2.10255	-0.10013
C	-1.45664	0.42530	1.71629
C	-1.49227	2.10331	-0.09957
C	1.45732	0.42483	1.71599
C	0.81129	0.83465	0.39668
C	-0.81081	0.83504	0.39684
C	-0.00106	-3.63109	-1.52822
H	3.60061	2.73199	-0.52858
H	3.20273	1.02463	-1.04484
H	1.19758	1.81408	-2.29228
H	1.52232	3.54313	-1.79829
H	-1.52082	3.54459	-1.79716
H	-1.19644	1.81567	-2.29180
H	-3.20198	1.02621	-1.04500
H	-3.59950	2.73346	-0.52809
H	1.43221	-1.09888	3.33584
H	1.33539	-1.82066	1.66967
H	3.32003	-0.52989	0.92329
H	3.50663	0.21703	2.58293
H	-3.50576	0.21838	2.58388
H	-3.32007	-0.52841	0.92407
H	-1.33583	-1.82026	1.66966
H	-1.43173	-1.09862	3.33593
H	1.38478	2.92522	0.60465
H	-1.24777	1.18570	2.46817
H	-1.38380	2.92567	0.60557
H	1.24910	1.18547	2.46780
H	-0.90273	-3.82793	-2.11049
H	0.88790	-3.82410	-2.13107
H	0.01056	-4.28543	-0.65057

Originally proposed correct structure of 249{5a} ($CDCl_3$)

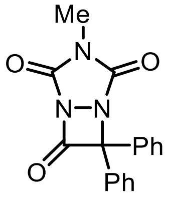

	Conf1	Conf2
Rel energy (kcal/mol):	0.00	0.00

C-nom	iGau	Exp	Calc	diff	1	2
C-C	8	164.50	165.19	0.69	[165.19	165.19]
C-C	3	158.30	157.51	-0.79	[157.51	157.51]
C-C	5	149.80	149.49	-0.31	[149.49	149.49]
C-C	12	133.00	133.19	0.19	[135.33	131.03]
C-C	18	133.00	133.17	0.17	[131.03	135.33]
C-C	9	94.50	96.35	1.85	[96.35	96.35]
C-CH	21	130.10	131.44	1.34	[132.22	130.65]
C-CH	15	130.10	131.43	1.33	[130.65	132.22]
C-CH	19	129.00	129.39	0.39	[130.73	128.05]
C-CH	23	129.00	129.39	0.39	[130.73	128.05]
C-CH	13	129.00	129.39	0.39	[128.05	130.73]
C-CH	17	129.00	129.39	0.39	[128.05	130.73]
C-CH	20	127.30	128.07	0.77	[128.10	128.04]
C-CH	22	127.30	128.07	0.77	[128.10	128.04]
C-CH	14	127.30	128.07	0.77	[128.04	128.10]
C-CH	16	127.30	128.07	0.77	[128.04	128.10]
C-CH3	11	26.60	26.18	-0.42	[26.18	26.18]

13C chem shifts: RMSD=0.82ppm (MAE=0.69) N=17 {-0.79 1.85}

Fractions: 0.501 0.499

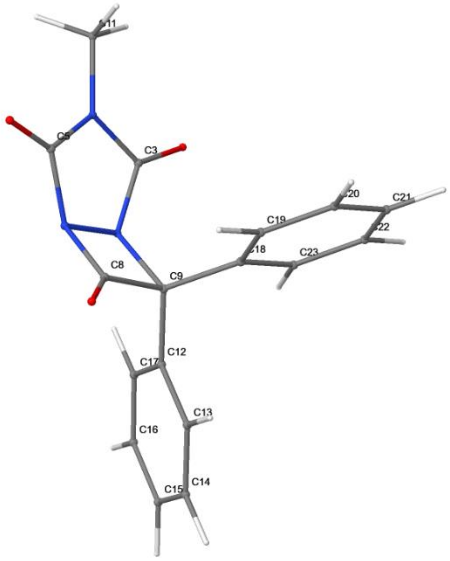


```
Conformer 1
Energy: -1045.53216 Hartree (Rel:  0.0 kcal/mol)
XYZ coordinates for conf 1:
N         -1.08172       -1.64488        0.58869
N         -0.56480       -0.99965       -0.59963
C         -1.66942       -0.48220       -1.30036
```

N	-2.80375	-0.98536	-0.64609
C	-2.50486	-1.59509	0.56469
O	-1.64817	0.19279	-2.30641
O	-3.26741	-2.00303	1.40886
C	-0.19106	-0.92959	1.44761
C	0.43777	-0.18983	0.22978
O	-0.03705	-0.96450	2.63290
C	-4.16657	-0.77305	-1.11445
C	1.87780	-0.59280	-0.03969
C	2.87003	-0.15529	0.85021
C	4.20124	-0.51366	0.64494
C	4.55516	-1.30284	-0.45376
C	3.56920	-1.73606	-1.34092
C	2.23121	-1.38935	-1.13299
C	0.19908	1.31291	0.21106
C	-0.46900	1.97014	1.25279
C	-0.69012	3.34870	1.18663
C	-0.25361	4.07773	0.08088
C	0.41304	3.42582	-0.96126
C	0.64332	2.05373	-0.89559
H	-4.10410	-0.34808	-2.11651
H	-4.69830	-1.72665	-1.14336
H	-4.69497	-0.08181	-0.45268
H	2.59978	0.46308	1.70151
H	4.96227	-0.17433	1.34185
H	5.59379	-1.57733	-0.61530
H	3.83604	-2.34888	-2.19734
H	1.46228	-1.73460	-1.81536
H	-0.80083	1.42292	2.12889
H	-1.20072	3.84791	2.00503
H	-0.42633	5.14905	0.03104
H	0.75772	3.98716	-1.82485
H	1.16703	1.55325	-1.70379

Conformer 2
Energy: -1045.53215 Hartree (Rel: 0.0 kcal/mol)
XYZ coordinates for conf 2:

N	1.08195	-1.64465	0.58892
N	0.56500	-0.99962	-0.59949
C	1.66958	-0.48220	-1.30029
N	2.80393	-0.98519	-0.64593
C	2.50509	-1.59478	0.56493
O	1.64829	0.19263	-2.30645
O	3.26766	-2.00254	1.40916
C	0.19117	-0.92938	1.44774
C	-0.43771	-0.18983	0.22978
O	0.03712	-0.96419	2.63302
C	4.16674	-0.77291	-1.11437
C	-0.19925	1.31295	0.21092
C	0.46810	1.97049	1.25291
C	0.68895	3.34908	1.18668
C	0.25287	4.07786	0.08059
C	-0.41307	3.42564	-0.96181
C	-0.64307	2.05351	-0.89608
C	-1.87769	-0.59299	-0.03972
C	-2.87005	-0.15517	0.84988
C	-4.20122	-0.51373	0.64460
C	-4.55495	-1.30342	-0.45379
C	-3.56885	-1.73695	-1.34064
C	-2.23091	-1.39006	-1.13270
H	4.69515	-0.08153	-0.45274
H	4.69849	-1.72650	-1.14313
H	4.10421	-0.34812	-2.11649
H	0.79960	1.42345	2.12926
H	1.19900	3.84852	2.00528
H	0.42538	5.14921	0.03068
H	-0.75741	3.98678	-1.82568
H	-1.16622	1.55280	-1.70450
H	-2.59996	0.46360	1.70093
H	-4.96235	-0.17415	1.34127
H	-5.59354	-1.57805	-0.61534

H	-3.83554	-2.35017	-2.19683
H	-1.46188	-1.73554	-1.81484
